# Pathogen recognition in compatible plant-microbe interactions

**DOI:** 10.1038/s41598-017-04792-5

**Published:** 2017-07-25

**Authors:** Fabio Rezzonico, Oliver Rupp, Johannes Fahrentrapp

**Affiliations:** 1 Research Group Environmental Genomics and Systems Biology, Zurich University of Applied Sciences, Wädenswil, Switzerland; 2 Bioinformatics and Systems Biology, Justus-Liebig-University Giessen, Giessen, Germany; 3 Research Group for Viticulture, Zurich University of Applied Sciences, Wädenswil, Switzerland

## Abstract

Microbial infections in plant leaves remain a major challenge in agriculture. Hence an understanding of disease mechanisms at the molecular level is of paramount importance for identifying possible intervention points for their control. Whole-transcriptome changes during early disease stages in susceptible plant species are less well-documented than those of resistant ones. This study focuses on the differential transcriptional changes at 24 hours post inoculation (hpi) in tomato leaflets affected by three pathogens: (1) *Phytophthora infestans*, (2) *Botrytis cinerea*, and (3) *Oidium neolycopersici*. Grey mould (*B. cinerea*) was the disease that had progressed the most by 24 hpi, both in terms of visible symptoms as well as differential gene expression. By means of RNA-seq, we identified 50 differentially expressed tomato genes specifically induced by *B. cinerea* infection and 18 specifically induced by *P. infestans* infection at 24 hpi. Additionally, a set of 63 genes were differentially expressed during all three diseases when compared by a Bayesian approach to their respective mock infections. And Gene expression patterns were found to also depend on the inoculation technique. These findings suggest a specific and distinct transcriptional response in plant leaf tissue in reaction to *B. cinerea* and *P. infestans* invasion at 24 hpi, indicating that plants may recognize the attacking pathogen.

## Introduction

Plant-microbe interaction starts with the arrival of the pathogen’s dispersal and infection units on the host. Visual disease symptoms of fungal/oomycetal attack in plant leaves only appear after a certain incubation period, by which time the application of plant protection products (PPP) may already be ineffective. Research work is mostly focused on the incompatible plant-pathogen interaction and aims to understand tolerance or resistance mechanisms and to identify the responsible genes. Results of compatible interaction in crops are not often analysed and reported. Analysis of the very first physiological reactions and the underlying changes at gene transcriptional level during disease attack could lead to a better understanding of disease mechanisms and help to develop new approaches for early disease detection, which in turn could contribute to improved PPP application techniques.

The increasing market for fresh and processed tomatoes (*Solanum lycopersicum*) makes this fruit crop one of the most important grown worldwide^1^. Like all plant species tomatoes are challenged by a wide variety of pathogenic organisms. Grey mould, late blight and oidium are among the tomato leaf diseases with the greatest economic impact. These three diseases are distinct in terms of the pathogen’s mechanism for host tissue invasion, time when the first symptoms develop and the response triggered in/by the host tissue. Grey mould, caused by the generalist *Botrytis cinerea*, is a destructive disease attacking more than 1400 plant species^2^. The necrotroph invades leaf tissue through the stomata or directly through the cuticle by forming appressoria and penetration pegs^3^. The symptoms may even become visible within the first 24–48 hours post inoculation (hpi)^4^. The hemibiotroph *Phytophthora infestans*, which causes late blight, is a relatively specific pathogen that mainly attacks tomato and potato leaves and harvested organs^5^. *P. infestans* invades leaf tissue through the stomata or the cuticle and haustoria that grow into the cells^6^. The first stage of late blight in tomato leaves is characterized by a biotrophic life style followed by a necrotrophic phase^7^. The growth of oomycetes, including *P. infestans*, is characterized during the biotrophic phase by nutrient uptake via haustoria–plant-derived nutrient and molecule exchange interphases located at the plant cell plasma membrane^6^. The biotrophic phase (48 hpi) was estimated by macro and microscopical assessments, and comparative gene expression studies with so-called transition (96 hpi) and necrotrophic phases (144 hpi)^8^. Depending on the experimental conditions, first symptoms appeared on the potato and tomato leaves at approximately 48–72 hpi^7, 9^. Oidium in tomato is caused by the obligate biotrophic fungus *Oidium neolycopersici*, which invades the epidermal cells by means of appressoria formation^10^. After invading the host tissue with appressoria formation at 6–8 hpi^11^, the first symptoms (conidia) become visible after seven days^12^. An up-regulation of the mildew locus O *SlMLO1*, *4* and *14* has been reported as early as 10 hpi for *O. neolycopersici* in the tomato cultivar Moneymaker^13^.

Plants have evolved different defence layers that mainly involve three signalling molecules: (1) salicylic acid (SA), (2) jasmonic acid (JA), and (3) ethylene (ET). The SA-dependent defence pathway is mostly activated by biotrophic pathogens such as during the early phase of *P. infestans* infection. In contrast JA and ET are mainly involved in defence reactions triggered by necrotrophic pathogens such as *B. cinerea* ^14, 15^. The genes *NPR1* and *PDF1.2*, *THI2.1*, *HEL*, and *CHIB* ^14^ are major components of the SA and JA/ET defence pathways. The cross-talk between the SA-JA-ET pathways is highly complex and the role of ET is to a certain extent inconsistent in terms of presence/absence, influence on resistance and virulence, and cross-communication with the JA and SA pathways^10, 16^.

Changes in gene expression are one of the first reaction levels that follow plant-environment interactions, including pathogen attack. These changes are known to occur at the infection site and in surrounding tissue. For instance, in *B. cinerea*-*Arabidopsis thaliana* interactions, differentially expressed (DE) genes have been reported at 12 and 24 hpi and at 0–6 and 6–12 mm distant from the infection site^17^ in high resolution with two-hours sampling intervals during a 48 hours experiment^18^. Comparable studies have been published for *P. infestans* ^8, 19, 20^ and in-depth information is available for host-*O. neolycopersici* interactions e.g.^13, 21^. To our knowledge, studies comparing more than one pathogen using same plant material, grown in tomato under equivalent greenhouse and laboratory conditions are not available. Knowing early and disease-specific reactions in susceptible plant may allow the establishment of reduced pesticide regimes and potentially site specific application techniques. A first attempt to identify such potential marker genes could either be based on comparable disease development stages or common time points. Since comparable stages of *B. cinerea*, *P. infestans* and *O. neolycopersici* invasion are not obvious due to their different strategies, we decided to use a fixed time point of 24 hpi as a base for comparison. Using an RNA-seq approach we aimed to identify *S. lycopersicum* genes that were differentially expressed at 24 hpi, specifically the following three diseases: grey mould, late blight and powdery mildew. The identified genes were characterized *in silico* using the commonly available databases.

## Results

### Infection with *B. cinerea* is evident at 24 hpi

**Figure 1:**
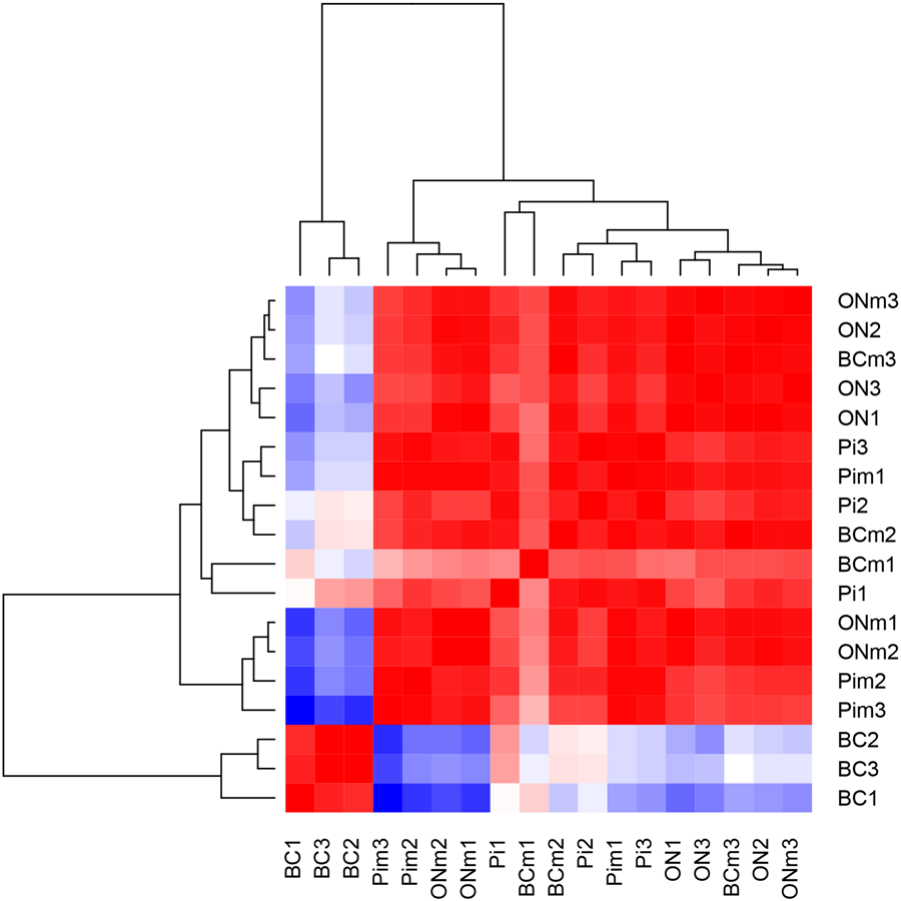
Pairwise comparison of read counts from the sequenced tomato leaf samples inoculated with either *B. cinerea* (BC), *P. infestans* (Pi) or *O. neolycopersici* (ON) and their corresponding mock (m) inoculations in three repetitions (1–3). Red, high similarity; blue, low similarity.

Nine pathogen-infected samples (PI: *P. infestans*, BC: *B. cinerea* and ON: *O. neolycopersici*) and their respective mock-inoculated variants (PIm, BCm and ONm) were subjected to Illumina HiSeq sequencing of the transcribed RNA at 24 hpi, which resulted in more than 1.13 billion total read pairs with a Q30 of 96% (project available at http://www.ebi.ac.uk/ena/data/view/PRJEB21223). Approximately 63 million read pairs were generated per sample. The reads were mapped against the available genomes of *S. lycopersicum* Heinz 1706, *B. cinerea* T4 and *P. infestans*. For *O. neolycopersici, there was* no genome sequence available. On average 80% of the reads per sample mapped to the host *S. lycopersicum*. 28 million read pairs (13.7%) of the *B. cinerea* and 1.4 (0.7%) of the *P. infestans* inoculated samples mapped to their respective genomes. One mock-inoculated sample of *P. infestans* infections yielded only 35% reads for *S. lycopersicum* and a *Penicillium spp* contamination was identified. Nevertheless the *S. lycopersicon*-derived reads clustered with the other mock-infections and therefore the sample was considered healthy. Pairwise comparison using edgeR^22^ on all the samples suggests similar patterns for all the mock-inoculated samples as well as for those inoculated with *P. infestans* and *O. neolycopersici*. In contrast, samples inoculated with *B. cinerea* clustered separately (Fig. 1), indicating a more advanced progression of the disease within the first 24 hpi.

Eleven genes that were differentially expressed in pairwise comparison of the pathogen and its corresponding mock inoculation were used to validate the RNA-seq expression. Trends in up- and down-regulated RNA-seq-logFC (log2 fold change) correspond to the mock sample-normalized expression measured by means of qPCR (quantitative real-time PCR; Supplementary Table S1). Three genes with no indicated differential expression in RNA-seq and a FDR > 0.69 were up-regulated in a qPCR evaluation. The R^2^ of the linear correlation in the RNA-seq and qPCR data was 0.92 (Supplementary Figure S1). Time-course experiments with sampling in three-hours intervals from 0–24 hpi and then at 48 hpi lead to a correlation at 24 hpi with the RNA-seq expressions of R^2^ = 0.8 (Supplementary Figure S2 and S3).

### Inoculation techniques alter gene expression

Nine patterns of cluster type [PI&PIm] vs. [BC&BCm&ON&ONm] comprising 668 genes were identified by summarizing genes that were differentially regulated between abaxial (PI and PIm) and adaxial (BC, BCm, ON and ONm) drop inoculation (all patterns can be found in Supplementary Data S1 with an explanatory file in Supplementary Data S2). Since *B. cinerea* was inoculated in half-strength grape juice, we tested if genes were clustering together in the pattern types [BC&BCm] vs. [PI&PIm&ON&ONm] and were able identify four patterns comprising 609 genes. Each sequenced gene can only be present in one pattern, hence there is no overlap between the genes expressed by different inoculation positions, inoculation media, and the ones identified as disease specific candidate genes that are described below. This suggests that both the location of the droplet deposition and the medium used for inoculation may impact gene expression.

### Genes are regulated by disease in general

**Figure 2:**
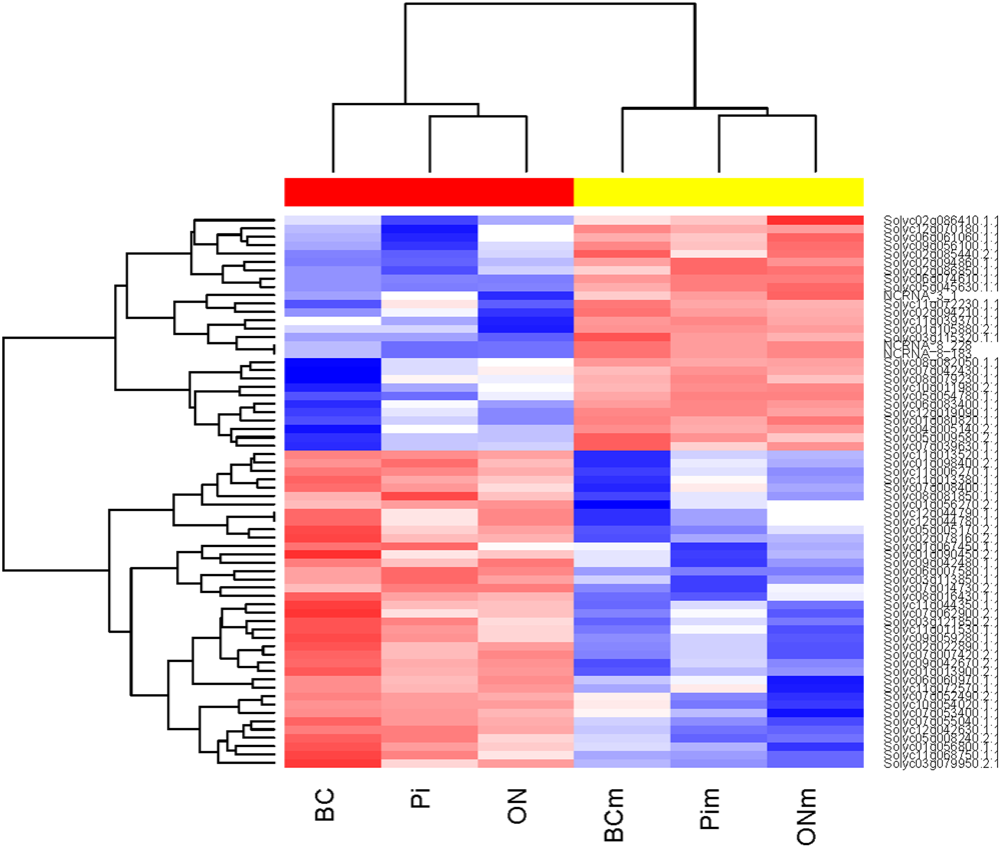
EBSeq pattern “healthy vs. diseased” ([PIm&BCm&ONm] vs. [PI&BC&ON]) identified DE genes from pathogen- and mock-inoculated tissues, comparing the overall similarity between the treatments. Of a total 63 genes were differentially expressed in the healthy and infected samples, independent of the inoculum source. Data is given for *B. cinerea* (BC), *P. infestans* (Pi), *O. neolycopersici* (ON), and their corresponding mock (m) inoculations. Blue represents up-regulated genes; red, down-regulated genes.

Using the R package EBSeq^23^ we identified one gene expression pattern (Fig. 2) that separates 63 genes by their differential regulation when diseased or healthy (Supplementary Table S2).

### The most DE genes were found in *B. cinerea* inoculated tissues

Gene expression following infection with each disease and their corresponding mock inoculations were analysed by pairwise differential expression analysis using edgeR^22^. Pairwise comparison of *B. cinerea* and the corresponding mock infected samples at 24 hpi revealed 2476 up-regulated and 2264 down-regulated differentially expressed (DE) genes (Supplementary Table S3). At 24 hpi, 661 genes were up-regulated and 49 were down-regulated in *P. infestans* compared to the mock infections (Supplementary Table S3). Following *O. neolycopersici* leaf tissue infection, one gene was up-regulated and 22 were down-regulated compared to the water control (Supplementary Table S3). Comparing the differential expression between the diseases, we found 511 up-regulated genes in both the *B. cinerea* and the *P. infestans* infected tissues, and 24 and 3 down-regulated genes in *B. cinerea*-*P. infestans* and *B. cinerea*-*O. neolycopersici* infected leaf tissue, respectively (Supplementary Figure S4).

### Grey mould and late blight-specific regulated genes were identified

In an attempt to identify disease-specific genes, the data were analysed with EBseq, and genes of informative patterns (i.e. sample combinations clustering for their gene expression against other samples which indicate whether they are “healthy” (BCm&PIm&ONm) or “diseased” (BC&PI&ON)) were extracted and compared to a pairwise comparison build with edgeR using a false discovery rate (FDR) < 1% and a logFC > 0.9. Genes belonging to a disease-specific pattern (only expressed for one disease and not for any other tested pathogen or mock inoculated samples) were extracted and analysed for their putative function using (1) Gene Ontology (GO) enrichment analysis, (2) an ortholog gene search of *A. thaliana* and *Solanum tuberosum* derived genes, and (3) protein database comparison.

EBseq resulted in 46 patterns for *B. cinerea*, 3 for *P. infestans* and 3 for *O. neolycopersici*, include gene candidates that are potentially disease-specifically regulated (Table 1). The 46 *B. cinerea*-specific patterns comprised 10009 genes, while the three patterns specific for *P. infestans* and *O. neolycopersici* included 178 and 80 genes, respectively (Table 1). These genes selected with EBseq were compared with the results of the DE of the pairwise comparison of the following pairs: BC vs. BCm, PI vs. PIm, ON vs. ONm, BC vs. PI, BC vs. ON and PI vs. ON. If the pattern-identified disease-specific genes were ‘DE’ for the specific disease-mock comparison and ‘not DE’ for the other pairs, they were selected for further characterization. This allowed 28 up- and 22 down-regulated genes to be identified that were specific for *B. cinerea* infections at 24 hpi, 18 up-regulated and no down-regulated genes with late blight specificity, and no DE genes for powdery mildew (Table 2). The *B. cinerea* infection specific up-regulated genes had a maximum logFC of 8.2 (*Solyc04g028460.1.1*, unknown protein) and the down-regulated genes a maximum of 5.3 (*Solyc12g096490.1.1*, *GDU1*). The strongest up-regulated gene during *P. infestans* infection was measured with a logFC of 4.4 (*Solyc02g068670.1.1*, *Ankyrin repeat-containing protein*).

**Table 1:** Overview on informative patterns and their numbers of genes.

Pattern associated genes	Pattern type	#Patterns
10009	[BC] vs. [all others]	46
178	[PI] vs. [all others]	3
80	[ON] vs. [all others]	3
193	[BCm] vs. [all others]	4
23	[PIm] vs. [all others]	3
71	[ONm] vs. [all others]	2
609	[BC] vs. [BCm] vs. [all others]	4
668	[PI] vs. [PIm] vs. [all others]	9
337	[ON] vs. [ONm] vs. [all others]	1
89	[BC&BCm] vs. [PI&PIm] vs. [ON&ONm]	2
63	[Disease] vs. [healthy]	1
655	[BC&PI] vs. [all others]	3
26	[PI&ON] vs. [all others]	1
54	[BCm&ONm] vs. [all others]	1
19	[BC&PI] vs. [ON&BCm] vs. [ONm&PIm]	1
12	[PI] vs. [BCm] vs. [all others]	1
8	[BC] vs. [PIm] vs. [all others]	1

For pattern type, the single clade that is separated from the others (i.e. BC: BC vs “BCm, PI, PIm, ON, ONm”) is provided. BC, *B. cinerea* inoculated, BCm, inoculation with BC-corresponding mock solution; PI, *P. infestans*; ON, *O. neolycopersici*. Possibilities: BC, BCm, PI, PIm, ON, ONm, and their given combination.

**Table 2:** Disease-specific genes that are differentially regulated in tomato leaves at 24 hpi.

Gene ID	Functional description	Putative disease specificity	DE compared to mock treatment	logFC	PValue	FDR	Gene size
Solyc04g028460.1.1	Unknown Protein	BCspec	up	−8.2	0.000459735	0.00329139	336
Solyc05g013650.2.1	Lysine ketoglutarate reductase trans-splicing related 1	BCspec	up	−7.7	4.07E-05	0.000417875	1215
Solyc06g066040.1.1	Unknown Protein	BCspec	up	−6.9	0.000356986	0.002655293	558
Solyc09g074220.1.1	Unknown Protein	BCspec	up	−6.7	0.000516971	0.003618668	666
Solyc09g090390.1.1	Unknown Protein	BCspec	up	−6.3	0.001655282	0.009693517	852
Solyc01g016380.2.1	Os06g0207500 protein (Fragment)	BCspec	up	−5.1	0.001600352	0.009431806	738
Solyc02g088840.2.1	Unknown Protein	BCspec	up	−5.1	0.000590923	0.004061215	1248
Solyc04g074360.1.1	UDP-glucuronosyltransferase	BCspec	up	−5.0	8.24E-05	0.000767071	1467
Solyc09g056290.1.1	RNA-dependent RNA polymerase	BCspec	up	−4.8	0.000872922	0.005660068	399
Solyc10g079900.1.1	Unknown Protein	BCspec	up	−4.6	0.000631817	0.004307987	1494
Solyc03g113580.1.1	Germin-like protein	BCspec	up	−4.5	0.000121579	0.001068314	654
Solyc01g099210.2.1	Lipoxygenase	BCspec	up	−4.4	0.000468489	0.003341085	2592
Solyc10g005440.1.1	Serine/threonine-protein kinase receptor	BCspec	up	−4.1	0.001589537	0.009380068	2505
Solyc09g011350.1.1	Plant-specific domain TIGR01570 family protein	BCspec	up	−3.9	0.001126102	0.007018453	747
Solyc04g005240.1.1	Unknown Protein	BCspec	up	−3.8	4.85E-05	0.000485408	276
Solyc06g053440.2.1	Unknown Protein	BCspec	up	−3.7	0.000586649	0.004035862	579
Solyc07g008360.1.1	p-coumarate CoA-ligase 2	BCspec	up	−2.6	0.000875671	0.005673897	1707
Solyc11g017390.1.1	Unknown Protein	BCspec	up	−2.5	0.000248335	0.001958543	459
Solyc08g063030.2.1	ADP,ATP carrier protein 1, mitochondrial	BCspec	up	−2.2	0.001274919	0.007787885	750
Solyc08g075230.1.1	Genomic DNA chromosome 5 P1 clone MDA7	BCspec	up	−2.2	0.00034842	0.002607255	597
Solyc03g097230.1.1	Protein containing AIG2-like domain	BCspec	up	−2.1	0.000771587	0.005104815	585
Solyc10g080560.1.1	DNA-3-methyladenine glycosylase	BCspec	up	−1.9	0.000871383	0.005652742	867
Solyc02g071520.2.1	RAG1-activating protein 1 homolog	BCspec	up	−1.5	0.000604591	0.004142788	708
Solyc01g087570.2.1	Unknown Protein	BCspec	up	−1.5	0.000438104	0.003162748	270
Solyc01g007770.2.1	Genomic DNA chromosome 5 P1 clone MHF15	BCspec	up	−1.4	0.000594061	0.004078728	525
Solyc06g068960.1.1	Calmodulin	BCspec	up	−1.3	0.001619588	0.009518784	465
Solyc07g048030.2.1	Heterogeneous nuclear ribonucleoprotein A3	BCspec	up	−1.3	0.001003225	0.006355831	1314
Solyc12g095790.1.1	Integral membrane protein like	BCspec	up	−1.2	0.001201251	0.007411601	1029
Solyc05g006900.1.1	Unknown Protein	BCspec	down	1.4	0.001087962	0.006805303	990
Solyc11g065180.1.1	THUMP domain-containing protein	BCspec	down	1.4	0.001054798	0.006623311	1152
Solyc01g111600.2.1	Metal ion binding protein	BCspec	down	1.4	0.000815055	0.005341251	462
Solyc05g048810.2.1	tRNA-specific adenosine deaminase	BCspec	down	1.4	0.00165971	0.00971739	1296
Solyc06g076850.2.1	Binding protein	BCspec	down	1.5	0.00115087	0.007150384	1656
Solyc06g063300.2.1	Kelch-domain-containing protein	BCspec	down	1.6	0.000487788	0.003460853	1845
Solyc06g053840.2.1	Auxin responsive protein	BCspec	down	1.7	8.40E-05	0.000779433	573
Solyc11g005640.1.1	Ubiquitin	BCspec	down	2.0	0.000607185	0.004158502	669
Solyc09g005020.1.1	Unknown Protein	BCspec	down	2.2	0.000987737	0.00627497	753
Solyc06g030540.2.1	Unknown Protein	BCspec	down	2.4	0.001068037	0.00669279	258
Solyc11g007530.1.1	Ring H2 finger protein	BCspec	down	2.4	0.000724629	0.004842834	672
Solyc08g007430.1.1	Nitrate transporter	BCspec	down	2.4	0.001056343	0.006627024	1773
Solyc04g028470.1.1	GDSL esterase/lipase 2	BCspec	down	2.5	0.000325126	0.002467012	963
Solyc06g075090.2.1	Lysine decarboxylase-like protein	BCspec	down	2.8	0.000124617	0.001090846	576
Solyc08g066450.1.1	Unknown Protein	BCspec	down	2.8	0.000395385	0.002898268	564
Solyc06g060830.2.1	Homeobox-leucine zipper protein	BCspec	down	3.0	5.86E-06	7.72E-05	897
Solyc01g010970.2.1	ARGONAUTE 1	BCspec	down	3.2	0.001442447	0.008661893	3003
Solyc10g076790.1.1	Auxin transporter-like protein 1	BCspec	down	3.3	0.001119952	0.006989584	1458
Solyc01g066640.2.1	Os04g0405500 protein	BCspec	down	3.5	0.00011117	0.000990706	1152
Solyc06g053210.2.1	Ubiquitin	BCspec	down	3.9	0.000567333	0.003921524	567
Solyc11g010340.1.1	BHLH transcription factor	BCspec	down	5.0	0.000959904	0.00612349	897
Solyc12g096490.1.1	GDU1	BCspec	down	5.3	4.39E-06	5.97E-05	495
Solyc02g068670.1.1	Ankyrin repeat-containing protein At3g12360	PIspec	up	−4.4	7.93E-18	1.54E-14	1704
Solyc07g056210.2.1	Unknown Protein	PIspec	up	−4.4	1.02E-17	1.64E-14	456
Solyc08g062490.2.1	WRKY transcription factor 16	PIspec	up	−3.0	3.83E-10	1.13E-07	546
Solyc01g079140.2.1	Unknown Protein	PIspec	up	−2.7	1.82E-07	2.28E-05	423
Solyc06g069740.1.1	Calmodulin-like protein	PIspec	up	−2.3	0.000194837	0.007839798	558
Solyc11g017280.1.1	Receptor like kinase, RLK	PIspec	up	−2.3	3.00E-10	9.30E-08	2796
Solyc01g009930.1.1	LRR receptor-like serine/threonine-protein kinase, RLP	PIspec	up	−2.3	0.000192504	0.007768893	1758
Solyc07g056200.2.1	NBS-LRR class disease resistance protein	PIspec	up	−2.2	2.92E-07	3.44E-05	390
Solyc02g077040.2.1	Cathepsin B-like cysteine proteinase 5	PIspec	up	−2.2	1.07E-07	1.42E-05	1038
Solyc03g115930.1.1	Calmodulin-like protein	PIspec	up	−2.2	1.65E-06	0.000152033	609
Solyc03g122350.2.1	Cytochrome P450	PIspec	up	−2.1	1.20E-10	4.37E-08	1527
Solyc03g095770.2.1	WRKY transcription factor 6	PIspec	up	−1.9	2.56E-06	0.000214804	822
Solyc04g074000.2.1	Receptor like kinase, RLK	PIspec	up	−1.9	8.80E-08	1.20E-05	3114
Solyc03g033840.2.1	26S protease regulatory subunit 6B homolog	PIspec	up	−1.8	6.24E-07	6.59E-05	1518
Solyc11g005630.1.1	Receptor-like protein kinase	PIspec	up	−1.8	3.56E-06	0.000282714	2334
Solyc02g081360.2.1	Long-chain-fatty-acid–CoA ligase	PIspec	up	−1.8	8.06E-06	0.000574741	1728
Solyc02g081350.2.1	Acyl-CoA synthetase/AMP-acid ligase II	PIspec	up	−1.7	0.000135705	0.005812497	1782
Solyc07g049660.2.1	Acetyl coenzyme A cis-3-hexen-1-ol acetyl transferase	PIspec	up	−1.6	1.43E-05	0.000924682	1404

The genes have been selected using R package EBseq and pairwise comparison (edgeR). Full description of the selected genes is provided in Supplementary Table S4.

It was possible to identify several disease-related genes. Taking into account (1) the ITAG2.4 *S. lycopersicum* gene sequence list and characterization, (2) *A. thaliana* and *S. tuberosum* orthologs, (3) UniProt characterization and (4) Blast2GO annotation, the analysis revealed two of the *B. cinerea* specific (*Solyc01g099210.2.1*, lipoxygenase; *Solyc10g005440.1.1*, serine/threonine protein kinase receptor) and two of the *P. infestans* infection specific up-regulated genes (*Solyc08g062490.2.1*, WRKY transcription factor; and *Solyc07g056200.2.1*, NBS-LRR class disease resistance protein) known to be related to disease response.

18 of *B. cinerea*-up-regulated genes, 18 of the *B. cinerea*-down-regulated genes and 16 of the *P. infestans*-up-regulated genes were characterized by an ITAG functional description. Among these 52 genes, we identified one carrier protein, one glycosylase, one ion binding protein, three kinases, three ligases, one lipase, one oxygenase, one proteinase, one receptor protein, one reductase, one RNA polymerase, two transferases, one nitrate transporter, one leucine zipper and three transcriptions factors (Table 2).

According to KEGG pathway information generated using STRING^24^, two up-regulated genes are involved in plant-pathogen interaction (*Solyc06g068960.1.1*, calmodulin; *Solyc06g069740.1.1*, calmodulin-like protein) and two down-regulated genes are involved in plant hormone signal transduction (*Solyc10g076790.1.1*, ARGONAUTE; *Solyc06g053840.2.1*, auxin-responsive protein).

### GO classification and enrichment analysis

The DE genes in the *B. cinerea* infection were classified with Blast2GO (v4.0.7) into 33 GO terms, and the ones related to the *P. infestans* infection into 14. The DE genes from the *B. cinerea*- and *P. infestans*-inoculated leaf samples shared three GO terms. GO enrichment analysis using the R package GOstat^25, 26^ identified ten GO terms related to biotic stress and defence that were overrepresented in the *P. infestans* inoculations at 24 hpi (P < 0.05; Supplementary Table S5). No such relation could be identified for the up-regulated genes from the *B. infestans*-inoculated samples (P < 0.05; Supplementary Table S6). The GO term “response to endogenous stimulus” was overrepresented in the down-regulated genes from the *B. cinerea* infection (P < 0.05; Supplementary Table S7).

### Ortholog genes in A. thaliana and S. tuberosum

**Figure 3:**
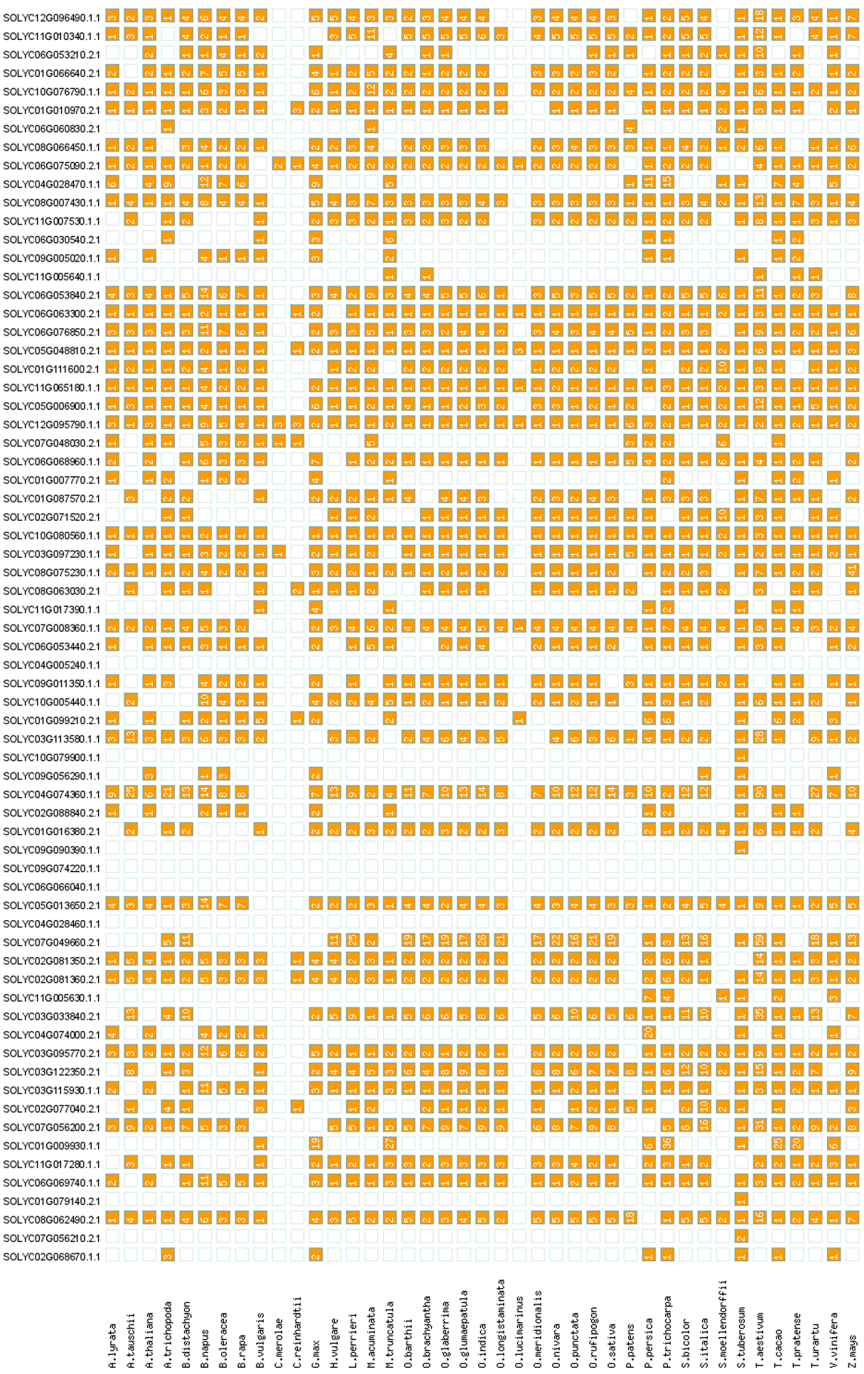
Gene orthologues of *B. cinerea*- and *P. infestans*-specific DE genes. Ortholog search performed with g:profiler^64^.

Comparing a total of 68 DE genes in tomato leaves infected with *B. cinerea* or *P. infestans* at 24 hpi with 41 different plant species, 38 ortholog *A. thaliana* genes and 43 ortholog *S. tuberosum* genes (Fig. 3) were identified. 26 DE genes were not represented by any ortholog *A. thaliana* genes and 11 were not represented by any *S. tuberosum* genes. Additionally, nine and 14 *A. thaliana* and *S. tuberosum* orthologues were described as “unknown protein”, “Conserved gene of unknown function” or “gene of unknown function” (Supplementary Table S4). For the *Solyc04G028460.1.1*, *Solyc06G066040.1.1*, *Solyc09G074220.1.1* and *Solyc04G005240.1.1* genes, all coding for unknown proteins and all being up-regulated after *B. cinerea* infection, no single ortholog could be identified. Orthologues of the *Solyc01g079140.2.1* (*P. infestans* up-regulated), *Solyc07g056210.2.1* (*B. cinerea* up-regulated), *Solyc09g090390.1.1* (*B. cinerea* up-regulated) and *Solyc10g079900.1.1* (*B. cinerea* up-regulated) genes–all coding for unknown proteins –were only found in *S. tuberosum*.

Performing species co-occurrence analysis (Supplementary Figures S5–S7) using STRING, *Solyc01g079140.2.1* (unknown protein), which was up-regulated during the *P. infestans* infection, was identified as being restricted to the *Solanaceae* family. *Solyc04g005240.1.1* (unknown protein) and *Solyc09g074220.1.1* (unknown protein), which were both up-regulated during *B. cinerea* infection, occurred only in *S. lycopersicum*, whereas *Solyc06g066040.1.1*, *Solyc09g090390.1.1* and *Solyc11g017390.1.1* (all coding for unknown proteins) were only found in the *Solanaceae* family. Since the orthologues have, to our knowledge, not yet been well characterized, the orthologue and co-occurrence analyses only provided information on the genes to be compared in future work.

## Discussion

According to our knowledge, this paper represents the first differential gene expression study of *S. lycopersicum* affected by different diseases. Pairwise comparison of pathogen- and mock-treated samples at 24 hpi revealed that the greatest number of DE genes in the tomato specimens occurred when the plants were infected with *B. cinerea*. This number was less for *P. infestans* and even lower for *O. neolycopersici*. 75% of the DE genes induced by *P. infestans* were also found to be DE in the tomato-*B. cinerea* interaction. This is in contrast to a study into the compatible interaction of tomato with *Cladosporium fulvum* and *Verticillium dahlia*, which shared 454 DE genes out of a total of 4693 seven days after inoculation^27^. Furthermore, we identified genes that are specifically expressed at 24 hpi for both *B. cinerea* and *P. infestans* infected leaves. No such specific DE genes could be found for the infection with *O. neolycopersici*. Both the pairwise comparison and the disease-specific differential expression underline the limitations of the selected experimental setup, which used 24 hpi as a fixed time point for comparison, since each of the diseases has differing invasion and colonization strategies, as well as different development strategies over time and space. Due to its necrotrophic lifestyle *B. cinerea* is the fastest of the three pathogens in terms of invasion and destruction of host tissue, leading to visible symptoms within 24 hours^4^. An intermediate development speed was reported for *P. infestans* ^7^, and this was also visible in our experiments. Powdery mildew was the last of the three diseases to display evident symptoms^12^. This is also true for other *Erysiphaceae*, whose symptoms develop in a range from 5.1 to 8 days, as has been shown for powdery mildew species screening in grapevines^28^. Additionally, the water-based inoculation method used may not provide optimal infection conditions for *O. neolicopersici*, since powdery mildew does not need complete wetness during infection^29^. Spores were often transferred by touching healthy tissue with sporulating lesions^12^, but the water-based method allows control and standardisation of the experimental setup in terms of experiment replication comparability with *O. neolycopersici* and other pathogen inoculations. And liquid conidia suspensions were used successfully in experiments with *O. neolycopersici* ^30, 31^.

We identified 68 specific differentially expressed genes for grey mould and late blight at a relatively early disease progression state at 24 hpi. These genes were either up- or down-regulated and never lead to resistance, since the Heinz 1706 cultivar is highly susceptible to both diseases. Nevertheless, many of the up- and down-regulated candidate genes are related to environmental stress. All 68 identified gene accession numbers were checked using google scholar for citings in other publications. Referenced publications could only be found for eight genes. The disease-specific genes include six genes that are related to plant-pathogen interactions and two that are related to hormone signalling. One of these, the lipoxygenase (*Solyc01g099210.2.1*, up-regulated during *B. cinerea* infection) was reported to be up-regulated during root nematode infection^32^. *SlWRKY80* (*Solyc03g095770.2.1*), which is up-regulated in *P. infestans* infected tissue, was also reported to be up-regulated during *Pseudomonas syringae* infiltration at 12 hpi^33^ and 6 days after infection with *Xanthomonas perforans* race T3^34^. *Solyc03g113580.1.1* (up-regulated during *B. cinerea* infection) coding for a Germin-like protein, was found to be expressed in tomato radicles grown under enhanced aluminium conditions^35^. *Solyc06g053840.2.1*, which is down-regulated during *B. cinerea* infection and coding for an auxin responsive protein (SlIAA4), is involved in hormone signal transduction and was up-regulated in young and old leaves and cotyledons compared to root^36^. *Solyc06g075090.2.1* was down-regulated in *B. cinerea* infected leaf tissue and was reported to be involved in cytokinin related synthesis and signalling^37^. *Solyc08g062490.2.1* (up-regulated in *P. infestans* infected leaf tissue) was annotated in the tomato genome (release ITAG2.4) as WRKY transcription factor 16, but is reported in literature as SlWRKY50^38^, a well-studied protein that mediates signalling of JA- and SA-pathways when the JA-pathway is repressed^39^.

When taking into account the gene description provided in the ITAG2.4 release, we could identify the following genes that potentially belonging to the major resistance (R) gene classes^40^ from the 68 candidates: (1) three genes coding for receptor like kinases (RLKs; *Solyc04g074000.2.1*, *Solyc11g017280.1.1*, *Solyc11g005630.1.1*), (2) two genes coding for lysine rich repeat proteins ((LRR; *Solyc07g056200.2.1*, *Solyc01g009930.1.1*), (1) one for serine/threonine-protein kinase receptor (*Solyc10g005440.1.1*), and (4) additionally three for transcriptions factors (TF, *Solyc11g010340.1.1*, *Solyc03g095770.2.1*, *Solyc08g062490.2.1*). RLKs can be involved in multiple processes including biotic and abiotic stress^41^. The RLKs, LRRs and two of the TFs were up-regulated during *P. infestans* infection, indicating a response to the pathogen invasion in the susceptible tomato tissue. However, this response did not lead to resistance. Therefore, this may suggest a disruption to downstream processes in disease resistance response. The serine/threonine-protein kinase was up-regulated during *B. cinerea* infection, which also failed to lead to a resistance reaction. *Solyc08g075230.1.1* includes a harpin-induced 1 interpro domain. The bacterial harpins induce disease resistance through the systemically acquired resistance pathways^42^.

Additionally, the *B. cinerea* inoculation down-regulated the Argonaute 1 gene (*AGO1*, *Solyc01g010970.2.1*), which may also be involved in biotic plant-environment interaction since an interaction of AGO1 with AGO2 in response to virus infection was demonstrated in *A. thaliana* ^43^. The argonaute Piwi subfamily to which AGO1 belongs supports the silencing of mobile genetic elements^44^ and antiviral RNA^45^. The down-regulated *Solyc11g010340.1.1* gene is one of 152 bHLH transcription factors identified in tomato. One of these TFs (*SlybHLH131, Solyc10g008270.2.1*) was reported to be involved in the tomato reaction to tomato yellow leaf curl virus infection^46^. *Solyc02g068670.1.1* was characterized to code for an “Ankyrin repeat-containing protein”. These proteins were reported to be related to resistance as a potential negative regulator of pathogen-induced protein PR1 and antioxidation metabolism^47^. Furthermore, a number of DE genes may be involved in abiotic interaction: *Solyc06g060830.2.1* down-regulated during *B. cinerea* infection codes for a putative homeobox-leucine zipper protein which is known to be involved in response to abiotic stresses^48^. The two genes coding for the homeobox-leucine zipper proteins *ATHB12* and *ATHB7* were up-regulated during drought stress^49^. The homologue gene *AtRNP1* of *Solyc07g048030.2.1* (Heterogeneous nuclear ribonucleoprotein A3) was reported to be involved in the abiotic stress response in *A. thaliana* ^50^. In our experimental setup, the only remarkable abiotic difference of the disease infection setup of the *B. cinerea* infections was the media that was used for inoculation (half strength grape juice), which might have exerted some stress due to its higher sugar level and osmotic potential but *Solyc07g048030.2.1* did not match the media-specific expression pattern. *Solyc06g068960.1.1*, *Solyc03g115930.1.1* and Solyc06g069740.1.1 potentially code for calmodulin and calmodulin-like proteins which are Ca^2+^ sensor proteins known to be involve in environmental stress responses^51, 52^.

In summary, the genes mentioned in the previous sections are related to biotic and abiotic stresses. The Heinz 1706 tomato cultivar we used for our experiments is obviously highly susceptible to all three diseases, hence no single related stress pathway which might be induced by these genes leads to a resistant phenotype. Nevertheless, some of the 68 genes we identified as late blight and grey mould-specific could be of use as indicators for pre-symptomatic disease identification if they are expressed systemically.

The presented multi-disease comparison at 24 hpi revealed several major findings: First, we identified genes that are differentially regulated in tomato leaves both during *B. cinerea* and *P. infestans* infections by comparing grey mould, late blight and powdery mildew leaf diseases with their mock infections. The identified candidate genes may be of use in identifying one of these two diseases before symptoms development. Therefore, the regulation of candidates will be evaluated in future works for their temporal and spatial expression patterns. During *O. neolycopersici* infection, no disease-specific differentially expressed genes were identified when compared to late blight and grey mould disease. Nevertheless, a pairwise comparison of *O. neolycopersici* inoculated leaf tissue with corresponding mock-inoculated tissues identified some DE genes. Second, most of the late blight and grey mould specific DE tomato genes are apparently not directly related to plant-pathogen interactions. Third, the results suggest that inoculation location (abaxial and adaxial) and inoculum solution solvent (water and half-strength grape juice) both have an impact on gene expression.

In future studies we will analyse the expression of identified disease-specific candidate genes over time and space within the whole plant and assess the potential use of one or a combination of these genes for early pre-symptomatic disease detection.

## Methods

### Plant material

Tomato plants (*S. lycopersicum*, Heinz 1706 cultivar) were grown in standard soil in a semi-regulated greenhouse with open windows. The temperature was set to 20–26 °C with maxima during sunny summer days of up to 40 °C. On cloudy days, artificial light was used to achieve minimal constant lighting of 80 kW per square meter for 16 h per day. Once a week, cuttings were produced from the tomato plants, which were treated once with sulphur (Stulln WG, Andermatt Biocontrol, Grossdietwil, Switzerland) and then placed in approximately 100% rel. humidity for one week. Afterwards the cuttings were acclimatised to the same greenhouse conditions mentioned above. Young and fully unfolded leaflets were harvested from two-week old cuttings for inoculation trials. 15 leaflets were placed in miniature grow boxes (30 × 60 cm and 14 cm in height with a clear plastic cover) on paper towels wetted with distilled sterile water. A separate box was used for each pathogen and mock inoculation. All inoculations were repeated three times.

### Inoculum preparation, inoculation and sampling for transcriptome analysis

A *P. infestans* strain K5276 that was isolated in Switzerland and kindly provided by Syngenta (Basel, Switzerland) was grown for 3–8 weeks on V8 medium (200 ml V8 Jus de Legume, Globus, Switzerland; 30 mM CaCO_3_, 1.5% (w/v) Agar, Sigma-Aldrich, Buchs, Switzerland). Sporangia were collected with 10 ml tap water, diluted to 4 × 10^5^ sporangia per ml and stored in darkness for 2 h at 5 °C before inoculation. Slight shaking of the inoculum solution hindered sporangia and zoospore sedimentation. 10 μl of the suspension was applied to the abaxial leaf surface for inoculation. The inoculated leaves were stored in darkness at 16 °C for 24 h, followed by a 16/8 hour day/night regime. Mock inoculations were performed under the same conditions using tap water for inoculation.

A *B. cinerea* strain T4 that was kindly provided by Philippe Nicot, INRA Centre de Recherche PACA, Montfavet, France, was grown on 15 g/l malt agar (Fluka, Sigma-Aldrich, Buchs, Switzerland) plates for 3–8 weeks. Spores were harvested with 20 ml half-strength grape juice (Farmer, Landi, Dotzingen, Switzerland) and diluted to 1.2 × 10^6^ spores per ml. The spore suspension was used directly for inoculation, with 10 μl drops placed on the adaxial leaf surface. The inoculated leaves were stored without light at 18 °C. Mock inoculations were performed under the same conditions using half-strength grape juice for inoculation.

*O. neolycopersici* that was isolated on tomato plants in the greenhouse at our institute was maintained on *S. lycopersicum* cv. Heinz 1706. Spores were harvested with a wet paint brush and diluted in water to a concentration of 4 × 10^4^ spores per ml, which was used to directly inoculate the adaxial leaf surface with 10 μl drops. The inoculated leaves were stored at 22 °C with a 16/8 hours day/night regime. Mock inoculations were performed under the same conditions using tap water for inoculation.

*Inoculation and Sampling:* Each inoculum was applied as approximately eight 10-μl-drops to the abaxial or adaxial surface of 15 leaflets (folioles^53^). Leaf disks (LD) of the inoculated sites were cut at 24 hours past inoculation (hpi) with a 5 mm-diameter cork borer for RNA-seq. LDs of eight leaflets were pooled, the remaining inoculum removed with a paper towel and frozen in liquid nitrogen. Samples were stored at −80 °C until further processing.

### RNA extraction, transcriptome sequencing and quantitative real time PCR

*Total RNA* was extracted using NucleoSpin® RNA Plant (Macherey-Nagel, Düren, Germany) following the manufacturer’s instructions, including DNase treatment. The RNA quality and quantity was estimated using the Standard Sensitivity RNA Analysis Kit in a Fragment Analyzer (Advanced Analytical Technologies, Ames, USA) and analysed with the associated PROSize® 2.0 v.1.3 software. Total RNA with an RNA quality number (RQN) >= 6 was sequenced with Illumina Highseq2500 v4 chemistry by GATC (Constance, Germany) using a strand-specific cDNA library from purified poly-A containing mRNA molecules.

Validating the RNA-seq-derived differential expressed genes primers for 11 DE genes (Supplementary Table S1) were designed using Primer3^54^. For qPCR total RNA was transcribed into first strand cDNA using an iScript cDNA synthesis kit (Bio-Rad, Hercules, CA, USA). Primers LSM7 and SlCBL1 that amplify genes that code for U6 snRNA-associated Sm-like protein LSm7 and Calcineurin B-like protein, respectively, were used as reference genes^55, 56^. The qPCR was conducted on a LifeCycler 480 (Roche, Basel, Switzerland) with a Fast EvaGreen® qPCR Master Mix (Biotium, Haywardm, CA, USA). For all primer pairs used in this study (Supplementary Table S1) the fast amplification protocol suggested by the master-mix provider was used, consisting of initial denaturation at 95 °C for 2’ followed by 40 cycles at 95 °C for 5” and 60 °C for 30”. A melting curve analysis was performed from 60–100 °C in 0.1 °C steps at the end of the run. All samples were analysed in duplicates. Primer efficiency was estimated by 1:10 dilution series run in triplicates (Fahrentrapp *et al*. under review). The same primers were applied to an additional time-course experiment (Fahrentrapp *et al*., unpublished, Supplementary Figure S2) conducted under the same conditions as described above. Leaf disc samples were taken at 3 hours intervals from 0–24 hpi and then at 48 hpi.

### Data processing and statistics

To estimate the percentage of RNA-seq reads coming from the infection, the reads from all samples were aligned to the ENSEMBL^57^ (release 30) genome of *S. lycopersicum* and to the pathogen genomes. The *B. cinerea* T4^58^ and *P. infestans* genomes^59^ were downloaded from the Broad Institute’s website. Since no reference genome was available for *O. neolycopersici*, reads from the infected and corresponding mock samples were assembled using Oases^60^ with a k-mer of 31. Resulting contigs were blasted against GenBank nt database using only contigs that matched fungal species for further analysis. The reads from the *O. neolycopersici* infected and mock-inoculated samples were mapped to the fungal contigs. For each sample, the percentage of reads that mapped the pathogen genome/contigs was calculated.

### Differential gene expression analysis

Expression levels for each sample were estimated using the RSEM package (version 1.2.25)^61^ in paired-end and strand-specific mode with bowtie2^62^ to the ENSEMBL (release 30) annotation of the *S. lycopersicum* genome. The RSEM “rsem-run-ebseq” tool was used to calculate the expression pattern based on the estimated expression levels for each gene based on the EBSeq method^23^. Significant patterns were assigned with a PPDE (posterior probability of being DE) >= 99%, which corresponds to a False Discovery Rate (FDR) of 1%. Additionally a pairwise comparison of all samples was performed using the R package edgeR^22^.

The resulting genes of interest were linked to their GO terms with Blast2GO (v4.0.7) and further analysed using g:Profiler^63, 64^, STRING^24, 65^ GOstat^25^ for GO enrichment analysis.

### Availability of materials and data

RNA-seq data are available at European Nucleotide Archive http://www.ebi.ac.uk/ena/data/view/PRJEB21223.

## Electronic supplementary material

Supplementary Materials

Supplementary Data 1

Supplentary Data 2
