# Supplementary material for: Pathogen recognition in compatible plant-microbe interactions
Source: Sci Rep. 2017 Jul 25;7:6383. doi: 10.1038/s41598-017-04792-5 (PMC5526865; doi:10.1038/s41598-017-04792-5)
Supplement: Supplementary file 1 — Supplementary Materials [file 41598_2017_4792_MOESM1_ESM.pdf]

Supplementary Materials
Pathogen recognition in compatible plant-microbe interactions

Fabio Rezzonico[1], Oliver Rupp[2], Johannes Fahrentrapp[3]*

[1] Research group Environmental Genomics and Systems Biology, Zurich University of Applied Sciences, Wädenswil, Switzerland

[2] Bioinformatics and Systems Biology, University of Giessen, Giessen, Germany

[3] Research Group for Viticulture, Zurich University of Applied Sciences, Wädenswil, Switzerland

* Corresponding author: johannes.fahrentrapp@zhaw.ch

Abstract

Microbial infections in plant leaves remain a major challenge in agriculture. Hence an understanding of disease mechanisms at the molecular level is of paramount importance for identifying possible intervention points for their control. Whole-transcriptome changes during early disease stages in susceptible plant species are less well-documented than those of resistant ones. This study focuses on the differential transcriptional changes at 24 hours post inoculation (hpi) in tomato leaflets affected by three pathogens: (1) Phytophthora infestans, (2) Botrytis cinerea, and (3) Oidium neolycopersici. Grey mould (B. cinerea) was the disease that had progressed the most by 24 hpi, both in terms of visible symptoms as well as differential gene expression. By means of RNA-seq, we identified 50 differentially expressed tomato genes induced by B. cinerea infection and 18 by P. infestans infection at 24 hpi. Additionally, a set of 63 genes were differentially expressed during all three diseases when compared by a Bayesian approach to their respective mock infections. And Gene expression patterns were found to also depend on the inoculation technique. These findings suggest a specific and distinct transcriptional response in plant leaf tissue in reaction to B. cinerea and P. infestans invasion at 24 hpi, indicating that plants may recognize the attacking pathogen.

Please navigate the supplemental material using provided bookmarks.

All tables can be provided as .xlsx. Please contact the corresponding author.

Supplementary Figure S1

Correlation of RNA-seq expression to qPCR of *B. cinerea* infected leaf samples at 24 hpi of genes summarized in Table S1.

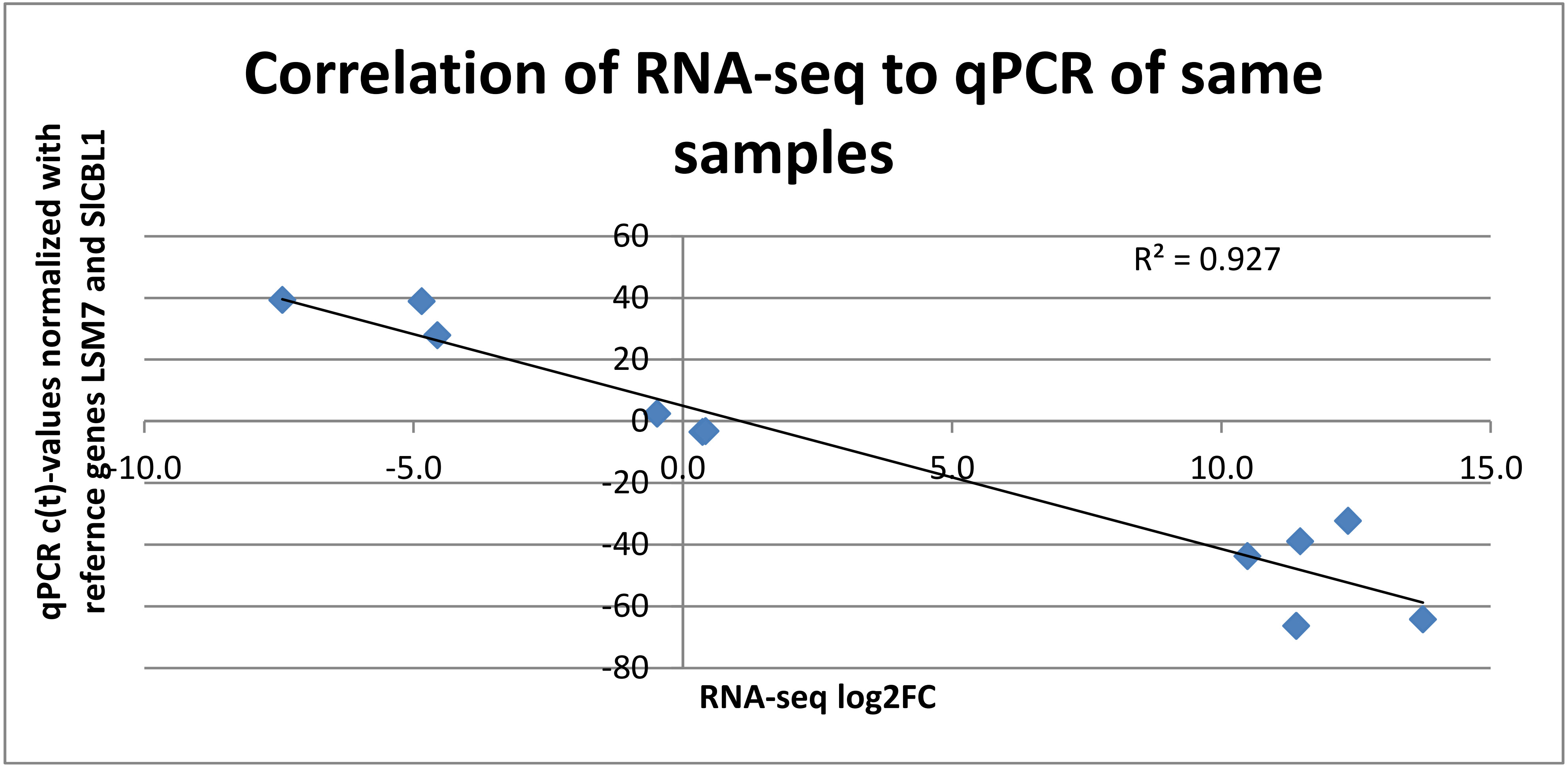

Supplementary Figure S2

Time course experiment. qPCR measured differential gene expression during *B. cinerea* disease development in *S. lycopersicum* leaflets in 3 hour intervals from 0-24 hpi and at 48 hpi.

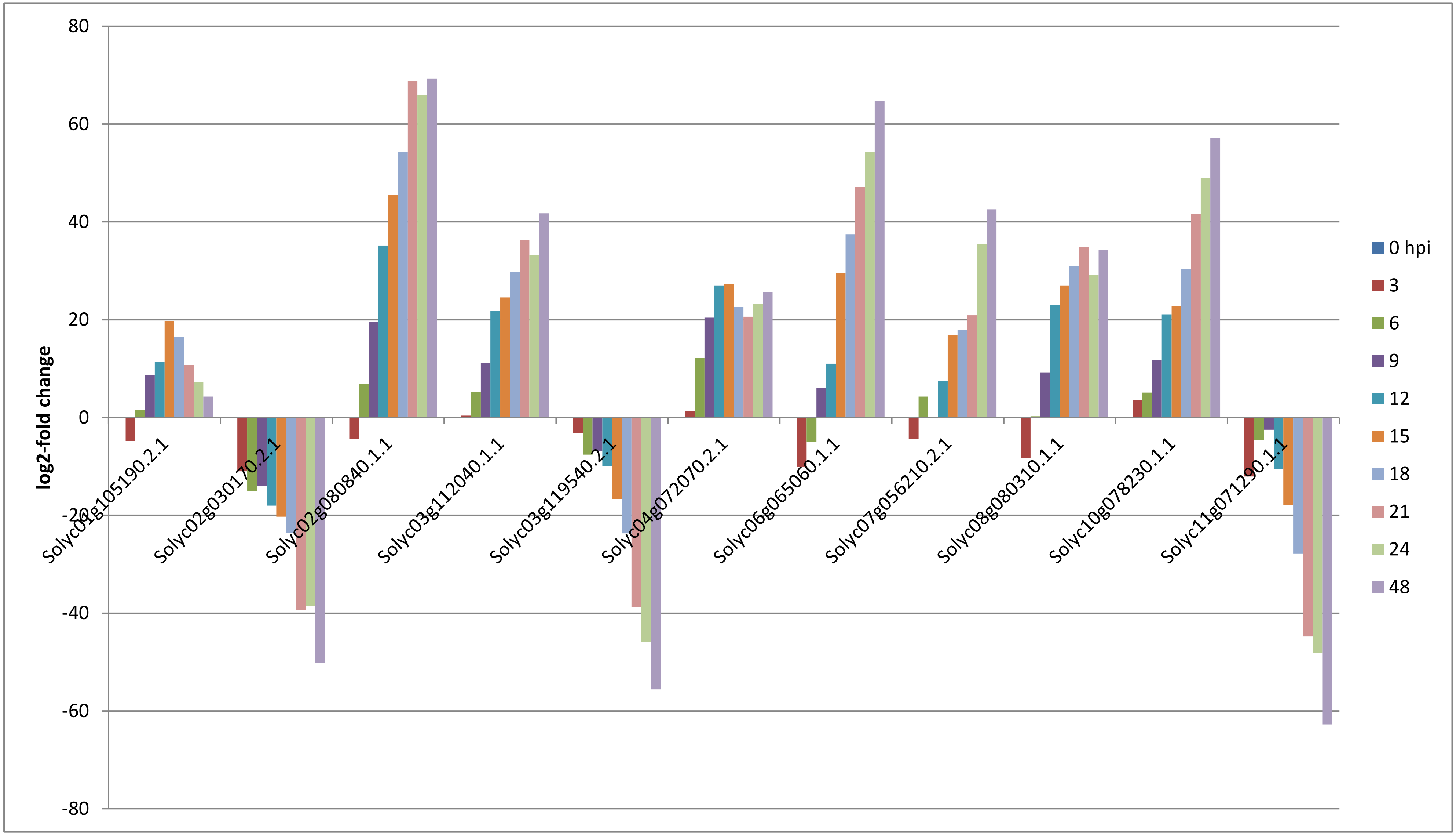

Supplementary Figure S3

Correlation of RNA-seq expression to qPCR of *B. cinerea* infected leaf samples at 24 hpi of genes summarized in Table S1. In contrast to Supplementary Figure S1 the samples analyzed with qPCR were taken from a seperate time course experiment at 24 hpi.

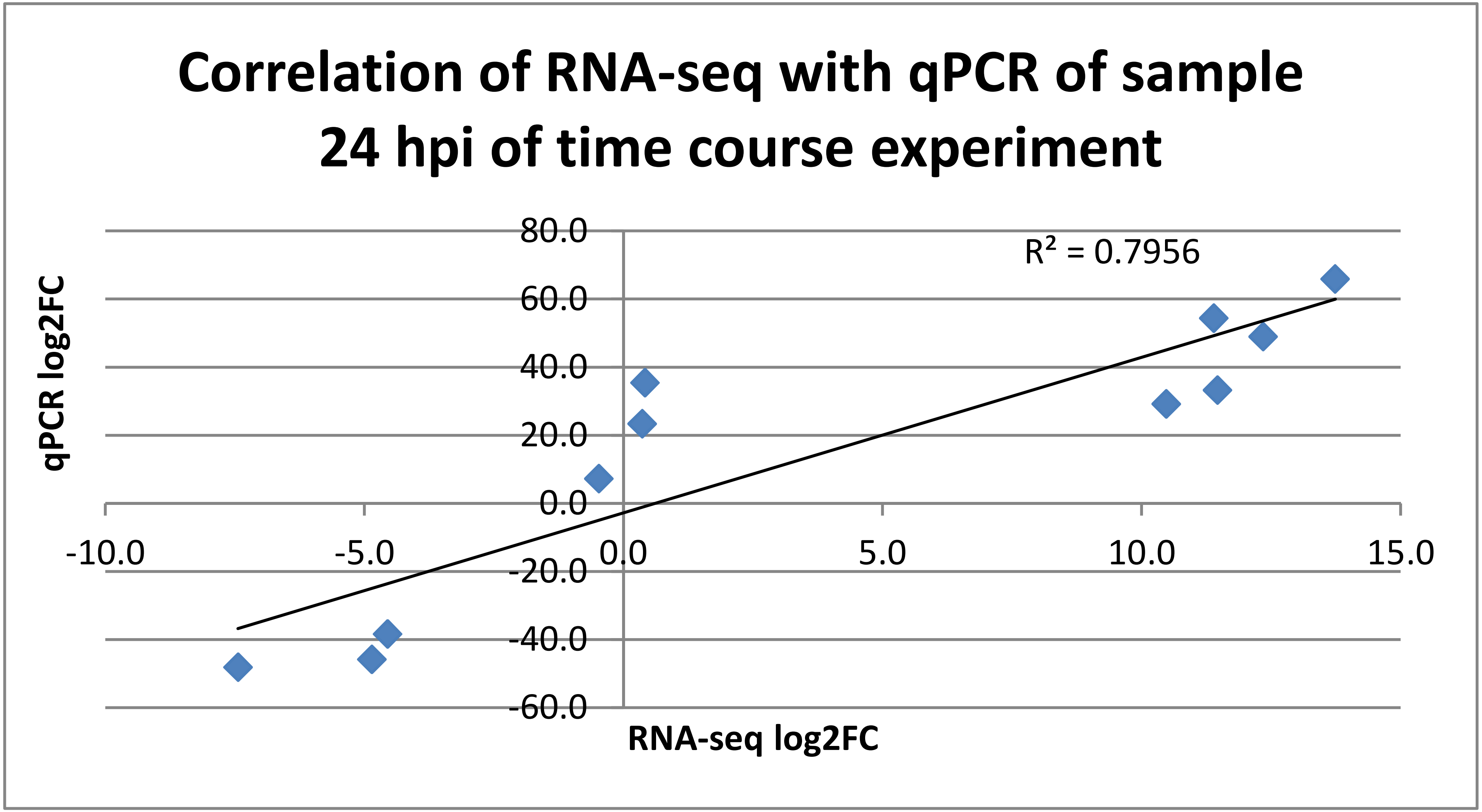

Supplementary Figure S4.1

Venn diagram of differntial expressed genes at 24 hpi identified by pairwise comparison of pathogen and mock.treaded tomato leaf samples. BC, B. cinerea; PI, P. infestans; ON, O. neolycopersici.

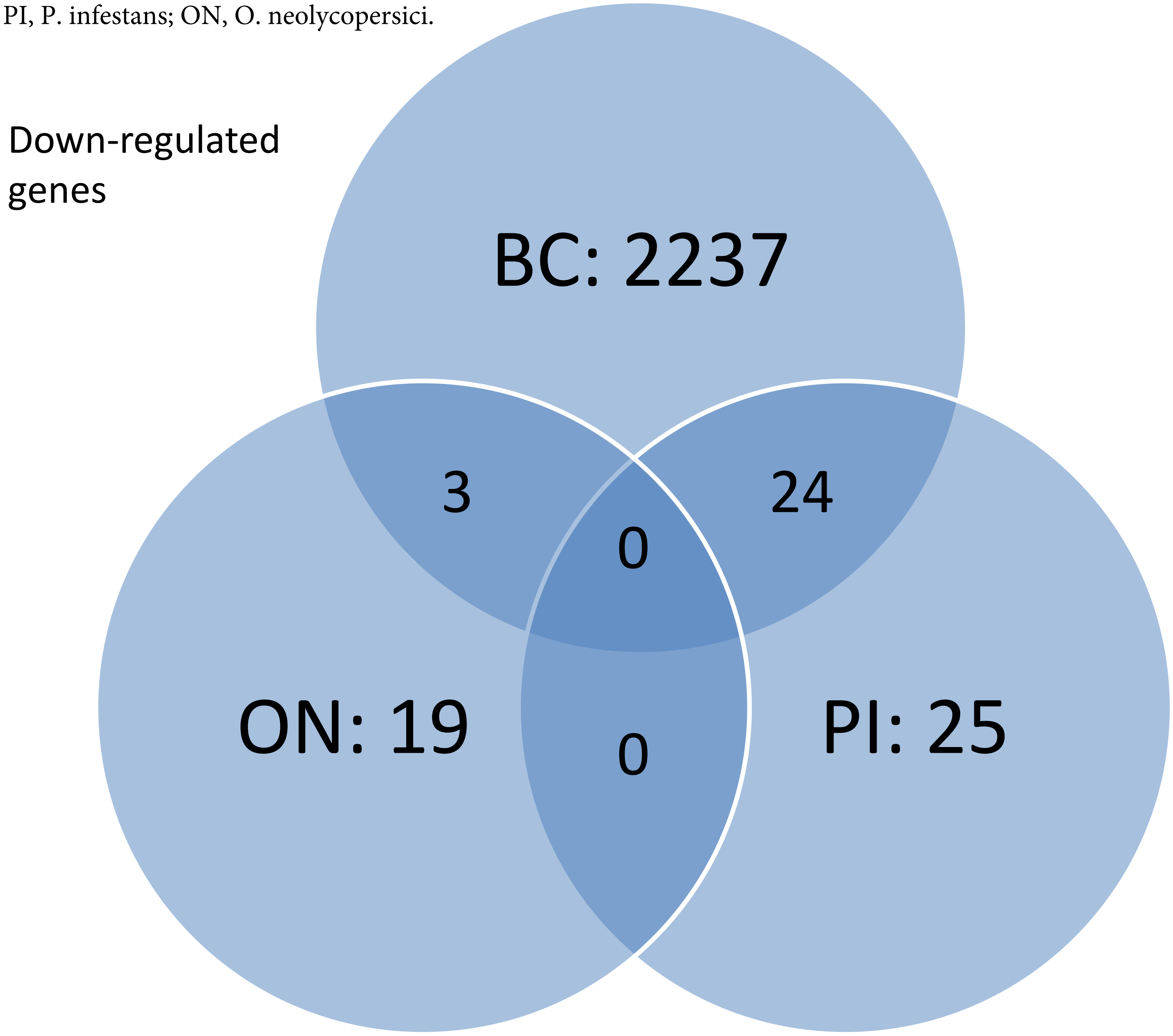

Supplementary Figure S4.2

Venn diagram of differntial expressed genes at 24 hpi identified by pairwise comparison of pathogen and mock.treaded tomato leaf samples. BC, B. cinerea; PI, P. infestans; ON, O. neolycopersici.

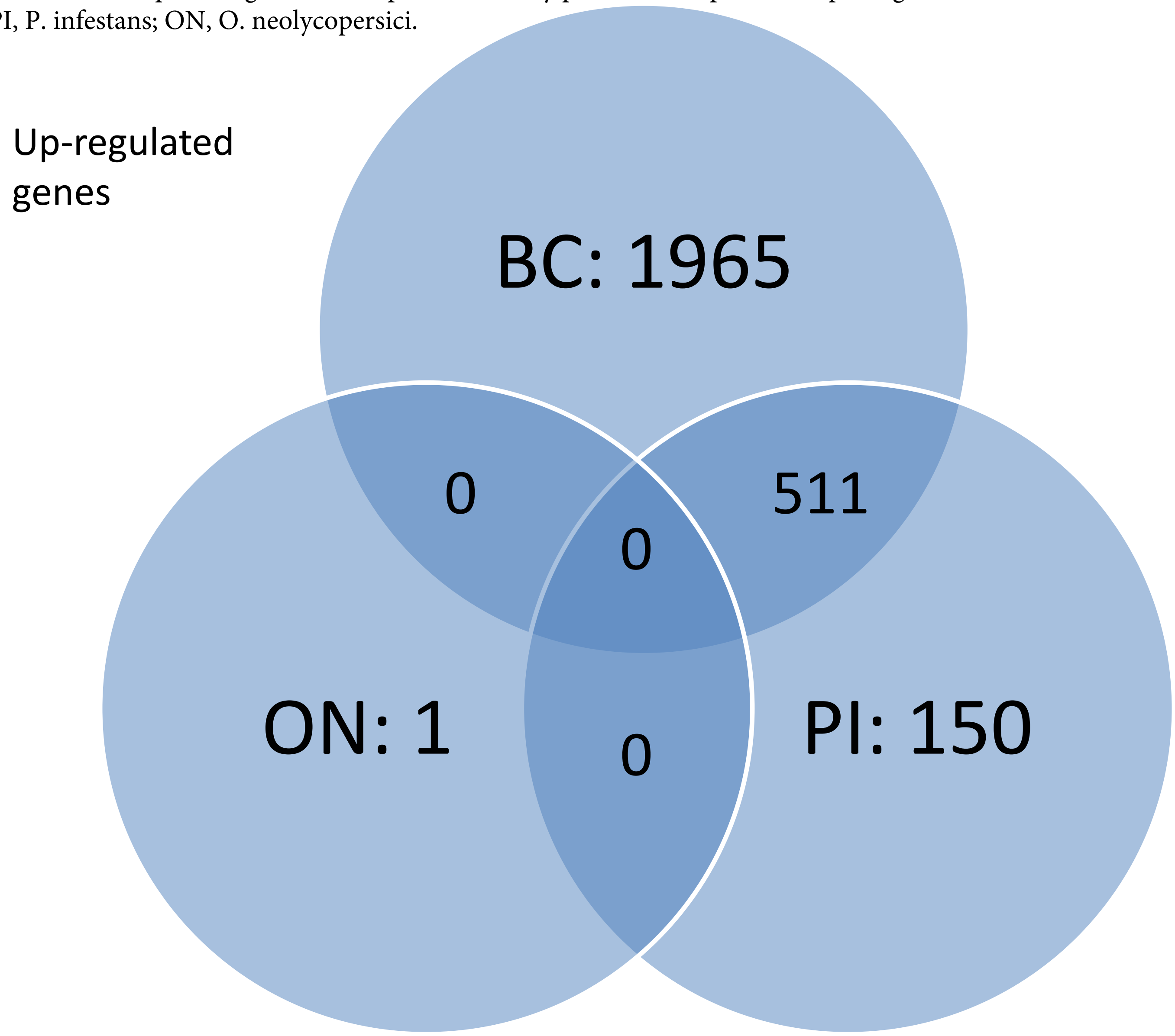

Supplementary Figure S5

Co-occurrence of DE tomato genes down regulated during *B. cinerea* infection at 24 hpi.

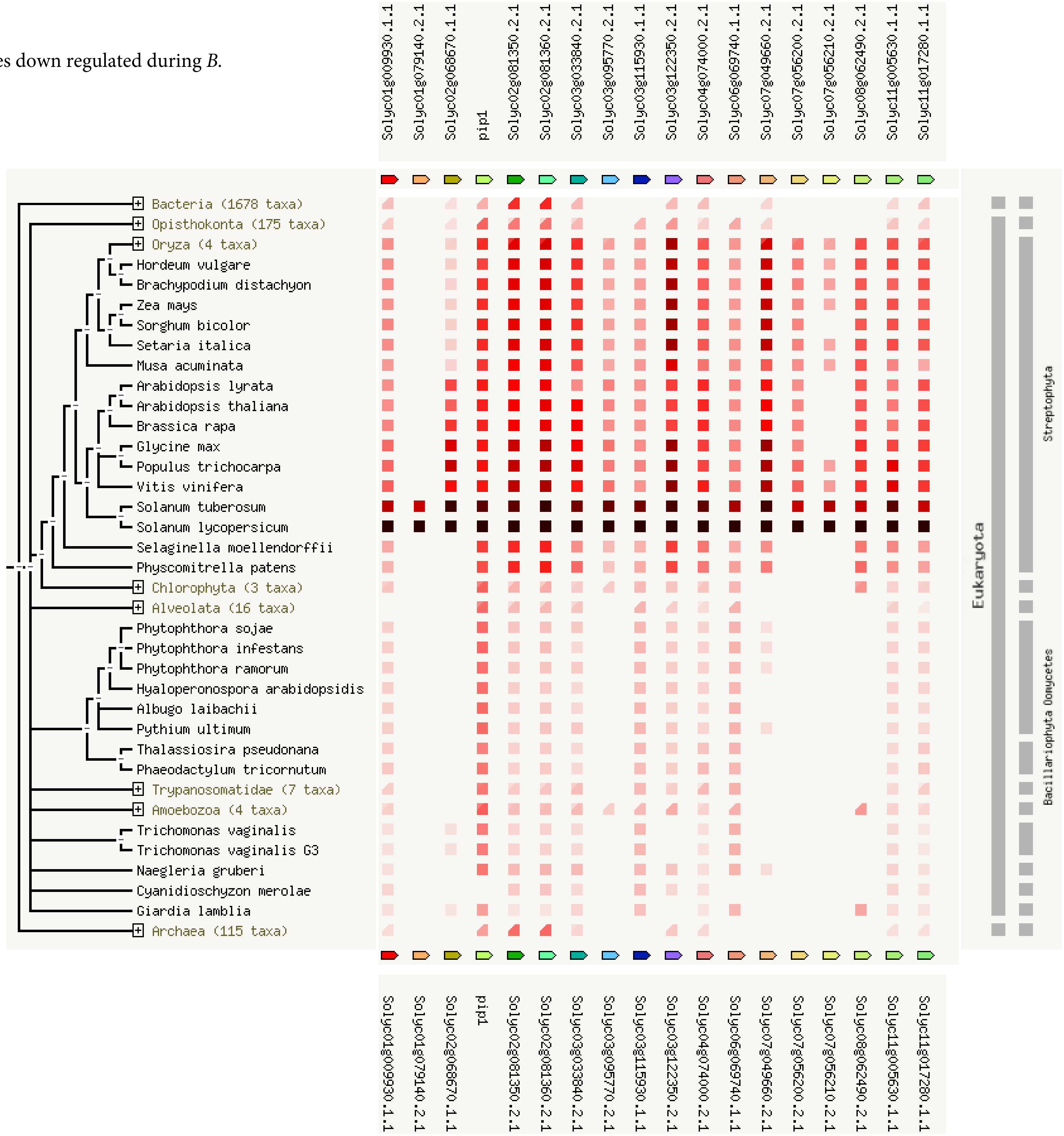

Supplementary Figure S6

Co-occurrence of DE tomato genes up regulated during *B. cinerea* infection at 24 hpi.

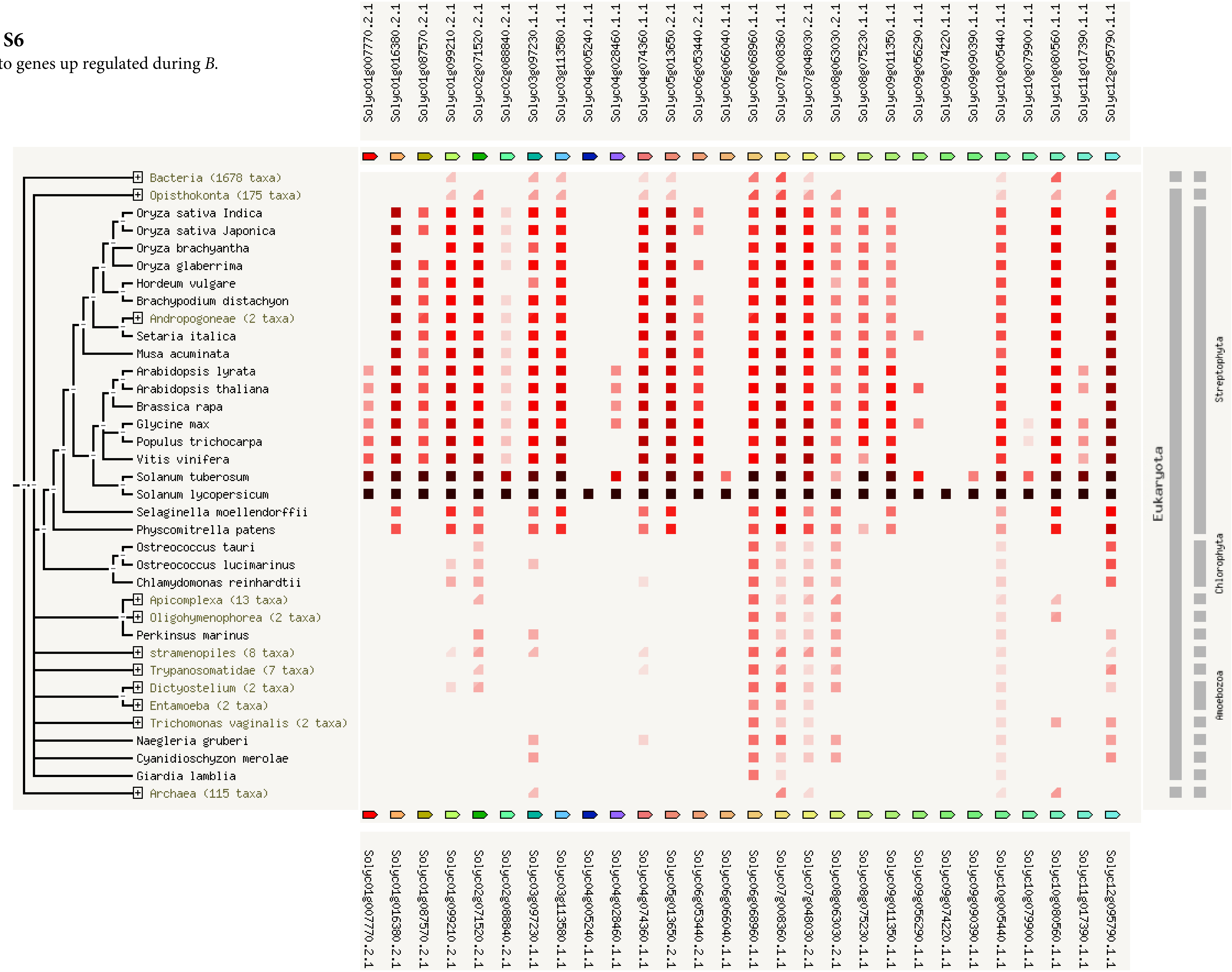

Supplementary Figure S7

Co-occurrence of DE tomato genes up regulated during *P. infestans* infection at 24 hpi.

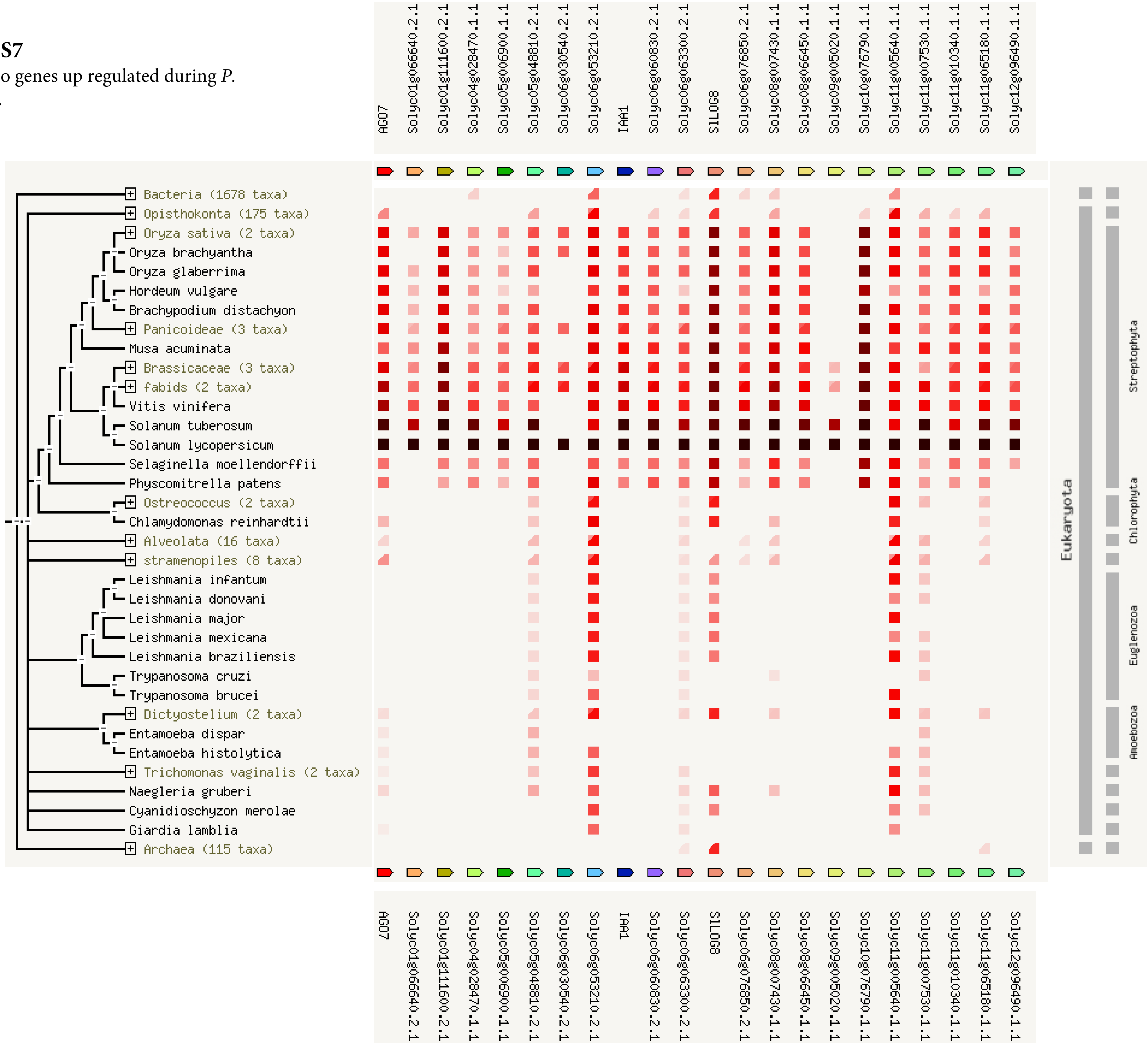

Supplementary Table S1

Primers designed in this study and their target gene for differential expression studies. DE was measured for *B. cinerea* (BC) inoculated and corresponding mock inoculated (BCm) samples at 24hpi and normalized to 0 hpi. As reference, genes coding for U6 snRNA-associated Sm-like protein LSm7 and Calcineurin B-like protein were used (Pombo, Zheng et al. 2014, Müller, Grau et al. 2015).

Gene ID	Protein Name	Primer sequence forward	Primer sequence reverse	Efficiency	Amplicon length	RNAseq DE BC vs BCm	log2FC BC vs BCm	FDR BC vs BCm	Reference gene- and mock infections-normalized qPCR of same pamples as uses for RNA-seq	Fold change log2 of qPCR in BC time course	SD
Solyc01g105190.2.1	Unknown Protein	TGGTCCGTGCTATTGAATTCTCT	CAGTACTGGTGCTCAAGTCTGT	93	153	not DE	-0.5	0.8	2.4	7.2	16.9
Solyc02g030170.2.1	FAD linked oxidase domain protein	TAGAAGTTCTGTTTGGCCTCTCT	CGAGAGAGGAAGGACGACGA	102	143	down	-4.6	4.20E-18	27.9	-38.5	12.7
Solyc02g080840.1.1	F-box-like	CGCGATCAAGGAAGCAGTTG	TACTGGTAGCTGCAATGCCA	97	146	up	13.7	1.24E-59	-64.3	65.9	6.2
Solyc03g112040.1.1	Cytochrome P450	GGCTGCTGGAACTCAAGTCT	GACAACCTCTTCTGCCTGCA	97	167	up	11.5	2.81E-18	-38.9	33.2	3.7
Solyc03g119540.2.1	CONSTANS-like zinc finger protein	TCAATCCTGATGACTACTGGCC	GCGATACCCTTGCTTCTCTTC	105	105	down	-4.8	4.01E-22	38.8	-45.9	9.5
Solyc04g072070.2.1	WRKY transcription factor 16	TGATGCCACACCAACACATG	TGCAATCTCCACTTGAACACTTG	100	215	not DE	0.4	0.7	-3.5	23.3	11.6
Solyc06g065060.1.1	FAD-binding domain-containing protein	ACTTTGCAGGGTTCCCAACA	TCCATCGAGACCTCCCTCTG	98	128	up	11.4	8.61E-46	-66.4	54.3	2.5
Solyc07g056210.2.1	Unknown Protein	AGCAGCAACTTTACCAGGAGT	TCTCAACTGGCTCTGGACCT	113	161	not DE	0.4	0.7	-3.2	35.4	11.1
Solyc08g080310.1.1	Multidrug resistance protein mdtK	AGGGGAACTTTCTTTAGCTGGT	GCACCAAAAGCTTGTCCACA	100	120	up	10.5	3.99E-51	-43.9	29.2	6.1
Solyc10g078230.1.1	Cytochrome P450	TGGGAAGGATTTGATATGGGCC	ACATTGCCAGGCTTAGTACAGT	117	174	up	12.3	1.37E-23	-32.4	48.9	3.5
Solyc11g071290.1.1	Alcohol dehydrogenase	TGCTACTGGTGGACTTGGTG	AACACAAGTCCAGCTCCAAG	105	132	down	-7.4	1.50E-21	39.2	-48.2	18.7

Supplementary Table S2

DE genes of EBSeq pattern “healthy vs. diseased” ([PIm&BCm&ONm] vs [PI&BC&ON]).

gene ID	functional_description
Solyc01g013900.2.1	Aldehyde dehydrogenase (AHRD V1 ***- Q71LY7_ORYSI);
Solyc01g056270.2.1	Unknown Protein (AHRD V1)
Solyc01g056800.1.1	Exostosin-like (AHRD V1 ***- Q2HVN7_MEDTR);
Solyc01g067450.1.1	Mutator-like transposase-like (AHRD V1 *--- Q9FH11_ARATH);
Solyc01g080820.1.1	Unknown Protein (AHRD V1)
Solyc01g090450.2.1	Actin-related protein 2/3 complex subunit 5 (AHRD V1 **** A5AQ06_VITVI);
Solyc01g098400.2.1	Histidine phosphotransfer protein (AHRD V1 **** B9I823_POPTR);
Solyc01g105880.2.1	(E)-beta-ocimene synthase (AHRD V1 **** Q5CD81_CITUN);
Solyc02g022890.1.1	Fatty acid hydroxylase family protein expressed (AHRD V1 ***- Q9AY73_ORYSJ);
Solyc02g078160.2.1	Plant-specific domain TIGR01615 family protein (AHRD V1 ***- B6UDN7_MAIZE);
Solyc02g085440.2.1	F-box family protein (AHRD V1 ***- D7MPC5_ARALY)
Solyc02g086410.1.1	Unknown Protein (AHRD V1)
Solyc02g086850.1.1	RNA exonuclease 4 (AHRD V1 ***- B6T4V3_MAIZE);
Solyc02g094210.1.1	Os02g0508100 protein (Fragment) (AHRD V1 ***- Q0E0Z9_ORYSJ)
Solyc02g094860.1.1	cytochrome P450
Solyc03g079950.2.1	DNA-binding protein BIN4 (AHRD V1 *-*- BIN4_ARATH)
Solyc03g113850.1.1	Guanine nucleotide-binding protein subunit beta (AHRD V1 *--* GBB_DICDI);
Solyc03g115320.1.1	Expansin (AHRD V1 ***- Q7X9Q3_SAMNI);
Solyc03g121850.2.1	Aleurone layer morphogenesis protein (AHRD V1 *-*- Q7XZV4_ORYSJ)
Solyc04g005140.2.1	Uridine kinase (AHRD V1 *-*- C3SA58_BRADI);
Solyc05g005170.2.1	Polygalacturonase 2 (AHRD V1 ***- A8D0T9_ONCHC);
Solyc05g008240.2.1	Unknown Protein (AHRD V1)
Solyc05g009580.2.1	Aluminum-activated malate transporter-like (AHRD V1 *--- Q5Z6M4_ORYSJ);
Solyc05g045630.1.1	Unknown Protein (AHRD V1);
Solyc05g054780.1.1	Disease resistance response/ dirigent-like protein (AHRD V1 ***- Q9M3C8_ARATH);
Solyc06g007580.1.1	Plant-specific domain TIGR01615 family protein (AHRD V1 *-*- B6SS18_MAIZE);
Solyc06g060970.1.1	Expansin-like protein (AHRD V1 ***- Q0WRS3_ARATH);
Solyc06g061060.1.1	Unknown Protein (AHRD V1)
Solyc06g074610.1.1	Unknown Protein (AHRD V1)
Solyc06g083400.1.1	Xyloglucan endotransglucosylase/hydrolase 2 (AHRD V1 **** Q2MK81_9ROSA);
Solyc07g007420.2.1	Leucine-rich repeat family protein (AHRD V1 **-- D7KDI8_ARALY)
Solyc07g008400.1.1	Pto-like, Serine/threonine kinase protein, resistance protein
Solyc07g014730.2.1	Phospholipase A2 (AHRD V1 **** Q5CCT8_TOBAC);
Solyc07g039630.1.1	Unknown Protein (AHRD V1)
Solyc07g042430.1.1	Dimethylaniline monooxygenase (AHRD V1 **** C1GPZ2_PARBA);
Solyc07g052490.2.1	Myb family transcription factor (AHRD V1 **** D7M4Z5_ARALY);
Solyc07g053400.1.1	Unknown Protein (AHRD V1)
Solyc07g055040.1.1	Agenet domain-containing protein (AHRD V1 ***- D7KG24_ARALY);
Solyc07g062900.2.1	Yippee family protein (AHRD V1 ***- D7MTM3_ARALY);
Solyc08g016430.1.1	Unknown Protein (AHRD V1)
Solyc08g079230.1.1	Cortical cell-delineating protein (AHRD V1 *--- B6T836_MAIZE);
Solyc08g081850.1.1	Unknown Protein (AHRD V1);
Solyc08g082050.1.1	UBX domain-containing protein (AHRD V1 ***- D7KBM2_ARALY);
Solyc09g042480.1.1	Chromatin remodeling complex subunit (AHRD V1 ***- B9N1U4_POPTR)
Solyc09g042670.2.1	Unknown Protein (AHRD V1)
Solyc09g056100.1.1	PG1 protein (AHRD V1 *-*- C4VMD4_9LACO)
Solyc09g059280.1.1	Histone H3 (AHRD V1 ***- D6PYM8_ORYSI);
Solyc10g011980.2.1	2-hydroxyacid dehydrongenase (Fragment) (AHRD V1 *-*- C6F725_PSEMZ)
Solyc10g054020.1.1	Unknown Protein (AHRD V1)
Solyc11g006270.1.1	3-oxo-5-alpha-steroid 4-dehydrogenase family protein (AHRD V1 ***- D7M7I5_ARALY);
Solyc11g011530.1.1	Unknown Protein (AHRD V1)
Solyc11g013380.1.1	GrpE protein homolog (AHRD V1 ***- B9RJC6_RICCO);
Solyc11g013520.1.1	Histone acetyltransferase (AHRD V1 **** B9H794_POPTR);
Solyc11g039370.1.1	Unknown Protein (AHRD V1)
Solyc11g044350.1.1	WW domain containing adaptor with coiled-coil (AHRD V1 *-*- Q5U4Q0_XENTR);
Solyc11g068750.1.1	NAC domain protein
Solyc11g072230.1.1	Glucan endo-1 3-beta-glucosidase 3 (AHRD V1 ***- B6TLN1_MAIZE);
Solyc11g072570.1.1	Proton-dependent oligopeptide transport family protein (AHRD V1 **-- D7LMP9_ARALY);
Solyc12g019090.1.1	Serine/threonine-protein phosphatase 7 long form homolog (AHRD V1 *--- PPP7L_ARATH);
Solyc12g042630.1.1	Mutator-like transposase-like (AHRD V1 *--- Q9FH11_ARATH);
Solyc12g044780.1.1	F-box family protein (AHRD V1 *-*- D7LXD8_ARALY);
Solyc12g044790.1.1	F-box family protein (AHRD V1 *-*- D7LXD8_ARALY);
Solyc12g070180.1.1	Ulp1 protease family C-terminal catalytic domain containing protein (AHRD V1 ***- Q60D46_SOLDE);

Supplementary Table S3

DE genes identified with pairwise correlation of *B. cinerea* - and mock-infected tomato leaf tissue.

Gene ID	logFC	logCPM	PValue	FDR	BCm1	BCm2	BCm3	BC1	BC2	BC3		
Solyc01g101190.2.1	-16.4747	6.764242	3.52E-48	1.21E-44	0	0	0	13145	6111	4394	BC up	2476
Solyc05g046340.1.1	-15.2992	5.588955	9.20E-42	1.82E-38	0	0	0	6217	2157	1939	BC down	2264
Solyc06g066230.2.1	-13.7785	6.429372	9.74E-60	1.35E-55	0	1	1	7324	6035	5317		
Solyc02g080840.1.1	-13.7404	6.873349	4.49E-64	1.24E-59	1	2	0	8046	11358	6974		
Solyc01g101210.2.1	-13.6818	3.972293	2.47E-20	4.21E-18	0	0	0	2577	225	460		
Solyc08g078920.1.1	-13.3721	3.662473	2.81E-20	4.77E-18	0	0	0	1633	134	762		
Solyc04g054380.1.1	-13.1697	3.461212	2.49E-32	1.57E-29	0	0	0	1271	942	305		
Solyc04g071590.1.1	-12.5505	5.173118	2.18E-55	2.01E-51	0	2	0	1548	4105	2547		
Solyc06g083960.1.1	-12.489	4.416728	1.21E-46	3.73E-43	0	0	1	809	2487	1561		
Solyc01g101050.2.1	-12.4606	2.753024	7.28E-28	3.10E-25	0	0	0	256	686	548		
Solyc06g011440.1.1	-12.3929	2.685667	3.33E-29	1.56E-26	0	0	0	628	489	306		
Solyc10g078230.1.1	-12.349	2.641256	3.86E-26	1.37E-23	0	0	0	518	294	476		
Solyc12g019620.1.1	-12.2345	2.527708	5.38E-29	2.44E-26	0	0	0	488	502	298		
Solyc01g094160.1.1	-12.1415	2.434674	2.32E-26	8.45E-24	0	0	0	266	472	435		
Solyc01g101180.2.1	-12.0011	8.530847	4.35E-26	1.52E-23	23	0	5	42241	21891	16329		
Solyc12g088850.1.1	-11.9878	2.28157	1.28E-25	4.12E-23	0	0	0	434	352	274		
Solyc05g008170.1.1	-11.9708	2.264806	7.32E-26	2.44E-23	0	0	0	422	407	242		
Solyc03g112040.1.1	-11.4626	1.758643	1.58E-20	2.81E-18	0	0	0	352	250	146		
Solyc02g094270.1.1	-11.4379	6.02639	2.20E-48	8.69E-45	0	10	0	5599	4762	3855		
Solyc01g008640.2.1	-11.4352	1.731347	6.64E-21	1.26E-18	0	0	0	324	250	158		
Solyc06g065060.1.1	-11.3937	9.271168	1.87E-49	8.61E-46	22	65	12	31507	68434	40645		
Solyc12g057070.1.1	-10.9825	1.281322	8.59E-15	7.22E-13	0	0	0	326	132	72		
Solyc09g091130.2.1	-10.969	1.265559	2.52E-16	2.59E-14	0	0	0	105	171	226		
Solyc02g078680.1.1	-10.96	1.257146	2.45E-15	2.24E-13	0	0	0	69	217	226		
Solyc09g064760.1.1	-10.8936	1.192809	6.38E-16	6.17E-14	0	0	0	264	142	92		
Solyc02g080920.1.1	-10.8636	1.164004	1.17E-15	1.10E-13	0	0	0	257	174	71		
Solyc06g082420.2.1	-10.846	1.148952	3.18E-15	2.85E-13	0	0	0	198	283	51		
Solyc02g070110.1.1	-10.823	8.049644	3.27E-41	5.64E-38	12	38	12	8045	40761	15271		
Solyc04g077240.2.1	-10.7872	1.087392	7.71E-17	8.56E-15	0	0	0	109	219	146		
Solyc09g066350.1.1	-10.7841	5.370428	9.83E-44	2.47E-40	0	10	0	4326	2615	2051		
Solyc07g042220.1.1	-10.7451	1.045871	1.87E-16	1.97E-14	0	0	0	194	166	97		
Solyc05g050870.2.1	-10.7439	1.045931	7.38E-12	3.94E-10	0	0	0	305	130	27		
Solyc05g039950.1.1	-10.7152	6.151324	3.14E-35	2.96E-32	1	4	11	8658	4383	2590		
Solyc04g015380.1.1	-10.657	0.95814	3.08E-09	9.43E-08	0	0	0	351	49	16		
Solyc01g005500.2.1	-10.6041	5.334685	3.28E-30	1.74E-27	7	2	0	5370	1826	1482		
Solyc07g052260.2.1	-10.4811	0.777287	5.69E-11	2.52E-09	0	0	0	29	104	209		
Solyc08g080310.1.1	-10.481	5.535837	5.77E-55	3.99E-51	4	9	0	3497	4849	2293		
Solyc04g072280.2.1	-10.4093	10.35195	4.85E-18	5.98E-16	243	22	29	65015	181447	66004		
Solyc08g076630.1.1	-10.3982	0.702106	6.03E-13	3.81E-11	0	0	0	201	99	55		
Solyc09g066160.1.1	-10.2991	2.180438	1.72E-20	3.02E-18	0	1	0	115	523	378		
Solyc04g054940.1.1	-10.2986	0.600465	2.94E-12	1.67E-10	0	0	0	171	58	85		
Solyc08g075980.1.1	-10.2643	0.566308	4.62E-11	2.09E-09	0	0	0	194	41	70		
Solyc02g076860.2.1	-10.2383	0.544255	2.34E-13	1.59E-11	0	0	0	149	110	62		
Solyc02g080850.1.1	-10.2363	4.89113	1.11E-54	6.13E-51	0	8	2	2404	2506	1674		
Solyc11g069880.1.1	-10.2314	2.835581	9.32E-27	3.58E-24	0	2	0	370	395	692		
Solyc06g083970.1.1	-10.0981	0.404032	3.77E-13	2.46E-11	0	0	0	106	95	83		
Solyc02g086040.1.1	-10.0722	0.386404	1.64E-10	6.62E-09	0	0	0	24	225	70		
Solyc08g082520.1.1	-10.0021	3.088071	4.57E-33	3.32E-30	0	3	0	604	629	599		
Solyc04g071600.2.1	-9.96564	7.8815	4.67E-43	1.08E-39	19	72	13	13861	23352	15590		
Solyc12g009130.1.1	-9.96382	0.286779	4.94E-07	8.88E-06	0	0	0	2	263	50		
Solyc10g078220.1.1	-9.94739	9.861819	3.42E-29	1.58E-26	108	285	20	60416	86584	60072		
Solyc02g078340.2.1	-9.86691	2.954287	7.42E-33	5.00E-30	0	3	0	527	671	508		
Solyc12g005440.1.1	-9.81122	0.115654	3.29E-10	1.26E-08	0	0	0	98	35	84		
Solyc09g014820.2.1	-9.80565	2.886644	1.89E-29	9.16E-27	0	3	0	314	901	487		
Solyc06g008540.1.1	-9.79485	0.10764	6.62E-11	2.89E-09	0	0	0	35	128	79		
Solyc06g082560.1.1	-9.78601	0.095699	1.64E-11	8.23E-10	0	0	0	56	90	83		
Solyc03g112710.1.1	-9.75581	0.067842	5.63E-11	2.50E-09	0	0	0	113	67	46		
Solyc09g066190.1.1	-9.7541	4.135315	1.00E-40	1.63E-37	6	0	1	1045	1565	1250		
Solyc03g098670.1.1	-9.74573	6.109675	1.90E-19	2.89E-17	0	40	1	3127	7883	4717		
Solyc06g068900.2.1	-9.72316	4.706936	1.50E-26	5.60E-24	10	0	0	833	2864	2134		
Solyc12g049040.1.1	-9.71249	0.02481	4.94E-10	1.79E-08	0	0	0	129	51	37		
Solyc07g062200.1.1	-9.69847	0.012814	1.64E-11	8.23E-10	0	0	0	58	103	63		
Solyc08g074260.2.1	-9.68879	7.32419	7.22E-39	1.05E-35	25	35	16	17873	8318	8071		
Solyc07g041650.1.1	-9.68784	0.017625	2.21E-08	5.64E-07	0	0	0	15	214	32		
Solyc09g005830.1.1	-9.64643	5.83165	7.23E-37	7.99E-34	6	24	1	2659	5282	4529		
Solyc07g061710.1.1	-9.63762	-0.0437	3.65E-11	1.69E-09	0	0	0	68	111	43		
Solyc12g005380.1.1	-9.62476	2.232832	1.18E-24	3.35E-22	0	2	0	395	529	174		
Solyc03g044850.1.1	-9.59433	2.298212	1.07E-20	1.97E-18	0	0	2	579	241	229		
Solyc06g075740.1.1	-9.5798	-0.09565	4.04E-10	1.50E-08	0	0	0	38	147	40		
Solyc05g016690.2.1	-9.55365	5.138333	7.64E-33	5.03E-30	0	22	0	2803	2941	2045		
Solyc06g008550.1.1	-9.53833	3.623904	7.48E-38	8.99E-35	3	2	1	655	1108	934		
Solyc10g080460.1.1	-9.51905	-0.17058	6.44E-10	2.28E-08	0	0	0	70	42	69		
Solyc01g080920.2.1	-9.47601	-0.20832	3.24E-10	1.24E-08	0	0	0	40	78	68		
Solyc05g047530.2.1	-9.43878	4.39578	1.43E-41	2.63E-38	0	12	1	1102	2150	1461		
Solyc12g019640.1.1	-9.43475	3.578013	2.80E-38	3.69E-35	0	7	0	835	1271	627		
Solyc03g083690.1.1	-9.42172	5.152333	5.62E-44	1.55E-40	11	6	2	2944	2655	2152		
Solyc07g052370.2.1	-9.41925	10.23029	4.86E-28	2.13E-25	283	357	48	112660	89713	61997		
Solyc01g090310.2.1	-9.3796	-0.30684	2.12E-08	5.43E-07	0	0	0	99	22	43		
Solyc09g011620.1.1	-9.36146	1.338041	2.48E-14	1.97E-12	1	0	0	169	90	242		

Solyc08g013930.2.1	-9.35307	-0.31741	2.70E-08	6.70E-07	0	0	0	92	82	11
Solyc11g045690.1.1	-9.33276	-0.34653	4.47E-10	1.63E-08	0	0	0	58	65	47
Solyc09g075700.1.1	-9.32479	8.549994	1.43E-08	3.83E-07	166	5	0	19109	45382	22343
Solyc08g007090.1.1	-9.32152	6.253816	3.86E-12	2.14E-10	35	0	0	6190	8598	3045
Solyc02g070090.1.1	-9.27395	8.495828	4.23E-22	8.92E-20	133	43	17	21957	46932	16761
Solyc02g005340.2.1	-9.2664	-0.41495	2.52E-08	6.31E-07	0	0	0	95	27	33
Solyc02g085800.1.1	-9.24913	3.309878	5.12E-33	3.63E-30	3	3	0	880	983	409
Solyc01g009370.1.1	-9.21828	-0.4408	2.42E-08	6.11E-07	0	0	0	43	123	15
Solyc07g005920.1.1	-9.18908	-0.48462	1.97E-07	3.92E-06	0	0	0	107	33	13
Solyc08g081820.2.1	-9.18862	5.044036	7.41E-23	1.66E-20	0	14	10	575	5916	1753
Solyc11g069680.1.1	-9.18553	4.559023	3.18E-29	1.52E-26	7	1	6	2986	1170	923
Solyc04g045280.1.1	-9.18089	-0.48192	1.29E-08	3.50E-07	0	0	0	58	95	16
Solyc02g080830.1.1	-9.17412	3.610893	1.82E-29	8.97E-27	0	9	0	432	1530	852
Solyc06g065420.1.1	-9.17179	-0.51085	1.53E-08	4.06E-07	0	0	0	65	28	49
Solyc02g080930.1.1	-9.17007	3.829943	2.20E-22	4.69E-20	8	0	0	1755	1015	414
Solyc07g054720.1.1	-9.16143	8.227168	5.37E-21	1.04E-18	120	44	11	23240	23050	19021
Solyc07g017360.1.1	-9.15631	-0.51807	3.19E-09	9.72E-08	0	0	0	64	51	35
Solyc06g061210.2.1	-9.15629	8.811003	5.62E-11	2.50E-09	195	62	0	43510	30072	24034
Solyc03g025640.1.1	-9.12795	5.925853	1.28E-12	7.71E-11	32	0	0	3224	7396	3497
Solyc03g098570.1.1	-9.12423	-0.54563	3.40E-08	8.19E-07	0	0	0	16	88	50
Solyc05g051350.1.1	-8.98834	5.085702	8.43E-42	1.79E-38	12	5	7	2147	4647	1370
Solyc01g087960.1.1	-8.97944	5.296101	6.99E-38	8.79E-35	16	4	7	3663	3202	1884
Solyc06g036440.1.1	-8.9175	1.674045	2.05E-17	2.40E-15	2	0	0	368	203	126
Solyc01g102380.2.1	-8.91721	-0.74115	4.04E-08	9.54E-07	0	0	0	21	82	34
Solyc04g083140.1.1	-8.90658	9.43843	2.03E-14	1.64E-12	358	92	14	74755	45970	31232
Solyc03g111300.1.1	-8.89134	3.800459	3.21E-35	2.96E-32	6	5	0	1196	960	852
Solyc07g009070.2.1	-8.88975	-0.76612	1.73E-06	2.67E-05	0	0	0	5	91	40
Solyc06g005100.2.1	-8.82835	-0.82371	9.08E-08	1.96E-06	0	0	0	51	64	14
Solyc01g006530.1.1	-8.79943	-0.86643	3.45E-08	8.27E-07	0	0	0	40	39	36
Solyc07g015910.2.1	-8.7856	-0.87363	5.74E-06	7.58E-05	0	0	0	3	72	46
Solyc04g051620.1.1	-8.77309	1.378463	2.88E-19	4.26E-17	0	2	0	182	237	157
Solyc09g082270.2.1	-8.76591	9.374882	5.22E-24	1.36E-21	277	262	34	44478	62003	41474
Solyc02g036240.1.1	-8.74292	3.90758	8.96E-25	2.63E-22	9	2	1	739	2559	480
Solyc02g065250.1.1	-8.71517	1.501971	0.000332	0.002514	0	0	2	0	825	0
Solyc01g067040.1.1	-8.71447	-0.94251	6.14E-08	1.39E-06	0	0	0	46	42	24
Solyc09g090210.2.1	-8.64367	3.75159	5.03E-35	4.34E-32	0	10	4	874	948	1042
Solyc06g075260.2.1	-8.50695	-1.13843	1.46E-06	2.30E-05	0	0	0	57	29	11
Solyc12g087800.1.1	-8.48469	3.357558	8.85E-18	1.08E-15	7	3	0	1425	677	197
Solyc08g048260.1.1	-8.48031	-1.16352	1.99E-07	3.96E-06	0	0	0	28	45	24
Solyc07g056100.1.1	-8.47291	2.277309	2.23E-23	5.17E-21	0	2	3	381	403	283
Solyc01g090980.1.1	-8.46793	-1.21432	1.73E-05	0.000198	0	0	0	18	7	54
Solyc02g036220.1.1	-8.46652	5.739531	6.33E-10	2.25E-08	44	0	0	3717	6472	2360
Solyc06g060450.2.1	-8.42886	-1.20878	8.38E-07	1.42E-05	0	0	0	14	55	27
Solyc10g055270.1.1	-8.40649	-1.2445	5.78E-07	1.02E-05	0	0	0	22	32	33
Solyc12g049170.1.1	-8.40167	-1.2593	0.000494	0.003484	0	0	0	64	0	17
Solyc01g110080.1.1	-8.39701	-1.21501	6.99E-06	8.95E-05	0	0	0	9	83	13
Solyc10g050550.1.1	-8.35215	-1.28474	4.43E-06	6.04E-05	0	0	0	53	25	9
Solyc07g018080.1.1	-8.34655	-1.29116	5.76E-07	1.02E-05	0	0	0	21	42	25
Solyc12g096740.1.1	-8.322	-1.31455	1.94E-06	2.95E-05	0	0	0	45	27	13
Solyc02g062300.2.1	-8.31046	9.82857	1.71E-31	1.03E-28	441	437	196	90695	56561	48816
Solyc01g099620.2.1	-8.30838	3.194182	1.17E-29	5.97E-27	2	10	0	569	1086	470
Solyc09g066200.1.1	-8.30587	2.110415	1.35E-19	2.10E-17	0	2	3	153	542	297
Solyc03g005500.1.1	-8.29776	5.681011	2.48E-12	1.42E-10	45	5	0	4758	3997	2578
Solyc12g096840.1.1	-8.29734	-1.34392	7.57E-07	1.29E-05	0	0	0	25	31	26
Solyc12g042850.1.1	-8.28592	-1.34867	1.77E-05	0.000201	0	0	0	58	18	6
Solyc01g098970.1.1	-8.26902	-1.34831	3.10E-06	4.40E-05	0	0	0	33	48	8
Solyc11g044500.1.1	-8.26462	-1.35318	1.61E-06	2.50E-05	0	0	0	25	52	12
Solyc09g013150.2.1	-8.24204	9.430915	3.34E-15	2.97E-13	557	128	41	56738	70901	30727
Solyc01g044520.1.1	-8.22624	-1.40613	1.38E-05	0.000161	0	0	0	5	45	31
Solyc04g025530.2.1	-8.21778	9.496654	8.19E-26	2.70E-23	444	334	102	37718	92768	39060
Solyc02g070130.1.1	-8.20526	-1.41412	2.07E-05	0.00023	0	0	0	4	55	25
Solyc03g098540.1.1	-8.1977	-1.42286	0.000343	0.002576	0	0	0	0	55	28
Solyc02g062310.1.1	-8.18164	0.982233	1.98E-10	7.85E-09	2	0	0	287	115	34
Solyc06g076160.2.1	-8.17832	8.406129	1.32E-29	6.63E-27	160	266	49	29476	24315	19886
Solyc04g028460.1.1	-8.16558	-1.52066	0.00046	0.003291	0	0	0	2	4	55
Solyc10g011920.1.1	-8.14071	0.142526	2.16E-10	8.50E-09	1	0	0	117	70	50
Solyc06g060600.1.1	-8.13968	1.73761	1.52E-15	1.41E-13	0	0	4	148	546	129
Solyc01g107080.2.1	-8.11624	8.585139	2.23E-30	1.23E-27	240	161	94	32793	31188	20856
Solyc07g064660.1.1	-8.11037	6.210495	7.86E-37	8.36E-34	25	59	26	2953	10699	4054
Solyc09g059410.1.1	-8.07595	-1.55733	0.000138	0.001187	0	0	0	55	4	8
Solyc04g078170.1.1	-8.07427	-1.53117	4.52E-06	6.15E-05	0	0	0	27	41	9
Solyc03g097510.1.1	-8.04764	1.25907	4.00E-15	3.52E-13	2	1	0	93	292	161
Solyc07g065150.1.1	-8.03967	-1.56392	2.43E-05	0.000265	0	0	0	38	32	4
Solyc07g052130.1.1	-8.01446	5.139007	7.08E-24	1.78E-21	26	26	0	2302	4116	1759
Solyc12g088330.1.1	-7.99908	3.334798	5.35E-24	1.38E-21	0	4	11	1008	717	485
Solyc09g059160.1.1	-7.99459	-1.59981	1.23E-05	0.000146	0	0	0	27	41	6
Solyc11g069870.1.1	-7.98965	-1.62549	3.29E-06	4.64E-05	0	0	0	19	30	19
Solyc07g042880.1.1	-7.98179	-1.64462	5.16E-06	6.90E-05	0	0	0	23	19	22
Solyc04g052960.1.1	-7.97852	-1.62591	4.72E-06	6.39E-05	0	0	0	26	33	11
Solyc10g009530.2.1	-7.97679	-0.01746	3.13E-10	1.20E-08	1	0	0	91	82	44
Solyc02g036180.1.1	-7.96686	0.568739	1.45E-12	8.69E-11	0	2	0	84	206	62
Solyc05g041220.1.1	-7.96598	-1.63106	9.95E-06	0.000121	0	0	0	10	46	16
Solyc12g006750.1.1	-7.96225	-1.6496	0.000988	0.006278	0	0	0	56	9	0
Solyc07g005380.2.1	-7.95535	7.524748	1.57E-24	4.35E-22	128	93	47	22287	10300	7334

Solyc05g050730.1.1	-7.95016	-0.07413	7.84E-10	2.73E-08	0	0	1	26	96	83
Solyc03g096420.1.1	-7.93708	1.339569	2.10E-15	1.92E-13	0	4	0	110	243	200
Solyc03g006440.1.1	-7.93459	-1.69884	3.61E-05	0.000377	0	0	0	32	7	20
Solyc07g062480.1.1	-7.91828	6.298948	2.80E-18	3.57E-16	60	50	10	10723	2766	3108
Solyc02g065150.1.1	-7.91252	2.61779	8.31E-17	9.19E-15	6	3	0	163	550	595
Solyc02g087090.2.1	-7.90551	-0.08737	8.45E-10	2.92E-08	1	0	0	89	63	49
Solyc01g079970.2.1	-7.90127	0.751746	6.74E-07	1.17E-05	2	0	0	313	29	13
Solyc08g078170.1.1	-7.88864	3.719819	5.29E-33	3.65E-30	10	9	2	1060	1312	624
Solyc02g065090.2.1	-7.88632	8.695896	1.77E-09	5.70E-08	464	41	23	62974	13770	11545
Solyc12g098790.1.1	-7.8781	-1.77285	0.001198	0.007392	0	0	0	26	0	27
Solyc11g043160.1.1	-7.87583	-1.73015	1.43E-05	0.000166	0	0	0	33	19	10
Solyc07g008120.2.1	-7.84426	4.467588	2.85E-14	2.23E-12	27	3	0	1670	1528	1530
Solyc02g038740.2.1	-7.81206	9.8873	3.96E-35	3.53E-32	423	930	382	94512	64879	47146
Solyc07g007740.1.1	-7.80463	-1.79912	1.04E-05	0.000127	0	0	0	13	26	20
Solyc11g006050.1.1	-7.78814	1.821823	1.00E-11	5.23E-10	5	0	0	493	130	124
Solyc09g082480.1.1	-7.77268	-1.86581	0.000112	0.000999	0	0	0	16	5	29
Solyc08g066100.2.1	-7.77106	1.424137	3.02E-13	2.02E-11	0	5	0	63	273	247
Solyc05g006380.1.1	-7.7707	-1.83348	1.77E-05	0.000201	0	0	0	10	25	22
Solyc01g099040.2.1	-7.76271	-1.77167	0.000461	0.003298	0	0	0	2	66	4
Solyc04g051360.2.1	-7.76264	-0.35691	8.43E-09	2.38E-07	0	1	0	24	57	79
Solyc03g005520.1.1	-7.75113	3.940826	6.16E-39	9.46E-36	9	14	5	1030	1394	973
Solyc02g078930.1.1	-7.72794	3.778183	6.80E-24	1.72E-21	16	4	2	1187	1322	606
Solyc05g006310.1.1	-7.72594	-1.86131	1.74E-05	0.000199	0	0	0	10	31	17
Solyc05g013650.2.1	-7.71179	-1.84834	4.07E-05	0.000418	0	0	0	9	45	9
Solyc08g078180.1.1	-7.69944	6.336483	1.80E-33	1.35E-30	20	121	34	6228	6918	4713
Solyc04g081620.1.1	-7.69124	-1.9089	0.000127	0.001108	0	0	0	37	7	8
Solyc12g088800.1.1	-7.66929	5.754625	1.03E-38	1.43E-35	36	28	33	3825	5642	2829
Solyc01g095080.2.1	-7.66806	9.21447	1.09E-08	2.99E-07	780	104	6	41296	71591	26310
Solyc01g006840.2.1	-7.65806	3.168963	9.27E-26	3.01E-23	6	12	0	539	751	659
Solyc03g078270.1.1	-7.65517	4.348022	1.95E-34	1.63E-31	17	19	3	1463	2036	1102
Solyc04g008530.1.1	-7.63863	-1.96929	0.000777	0.005138	0	0	0	37	1	10
Solyc02g078650.2.1	-7.63407	10.1228	5.75E-32	3.53E-29	542	1316	485	56454	168117	46992
Solyc04g080320.1.1	-7.63256	-1.93985	1.87E-05	0.000211	0	0	0	18	27	10
Solyc11g068570.1.1	-7.62615	-1.95533	0.000107	0.000958	0	0	0	34	13	5
Solyc05g012660.1.1	-7.60183	6.515922	2.38E-37	2.74E-34	48	116	33	6992	9100	4649
Solyc08g059710.2.1	-7.60042	3.921741	3.20E-30	1.74E-27	16	8	4	1182	1345	841
Solyc10g050730.1.1	-7.57602	2.919311	6.82E-15	5.76E-13	0	19	0	873	581	241
Solyc07g005080.1.1	-7.56237	0.164894	6.77E-11	2.95E-09	0	2	0	97	114	43
Solyc01g106530.1.1	-7.55447	-0.46348	1.90E-06	2.91E-05	0	0	1	63	9	66
Solyc05g053610.2.1	-7.54746	6.199215	8.48E-09	2.39E-07	0	204	8	2945	9964	4366
Solyc02g071480.2.1	-7.52205	-2.03804	0.000277	0.002145	0	0	0	32	16	2
Solyc03g044180.1.1	-7.49737	7.340985	1.72E-10	6.86E-09	195	120	1	12204	16784	7993
Solyc10g085110.1.1	-7.49559	-2.06329	0.000124	0.001082	0	0	0	28	17	4
NCRNA_1_340	-7.48039	-2.07409	7.69E-05	0.000722	0	0	0	6	30	14
Solyc02g021350.2.1	-7.4775	2.252718	2.28E-19	3.44E-17	5	5	0	236	569	288
Solyc11g071990.1.1	-7.47015	4.021055	8.94E-37	9.15E-34	8	20	9	796	1986	968
Solyc10g011930.1.1	-7.46251	7.187878	4.92E-14	3.70E-12	125	211	4	12242	13279	7203
Solyc06g048800.1.1	-7.46164	-0.64992	2.27E-08	5.78E-07	0	1	0	39	33	56
Solyc11g069560.1.1	-7.44281	6.245413	9.15E-09	2.56E-07	118	13	1	8966	5700	2331
Solyc11g018800.1.1	-7.4123	9.624571	8.71E-22	1.81E-19	836	609	223	86730	50648	34613
Solyc03g112030.1.1	-7.41102	6.472884	4.35E-25	1.35E-22	92	68	29	9487	6615	3584
Solyc09g097950.1.1	-7.40557	-2.11606	0.000115	0.001019	0	0	0	9	36	6
Solyc01g102900.2.1	-7.39265	2.004317	2.53E-12	1.44E-10	0	3	6	57	542	321
Solyc06g075730.1.1	-7.38768	-0.01095	3.67E-10	1.37E-08	0	2	0	45	110	66
Solyc03g078280.1.1	-7.38481	4.334743	7.85E-28	3.29E-25	22	22	2	1432	1927	1155
Solyc07g052120.2.1	-7.37654	7.595074	4.52E-18	5.60E-16	234	157	24	12509	20997	10615
Solyc08g006750.2.1	-7.37341	9.392492	1.44E-19	2.22E-17	854	310	185	49808	69791	33600
Solyc07g049670.2.1	-7.36499	2.494697	1.67E-12	9.86E-11	6	7	0	855	152	174
Solyc08g079080.2.1	-7.35403	1.004438	3.35E-11	1.56E-09	0	5	0	42	310	125
Solyc12g013470.1.1	-7.34905	1.025211	3.67E-14	2.82E-12	0	5	0	169	181	102
TRNA_1_54	-7.3455	-2.23352	0.000439	0.003168	0	0	0	24	3	12
Solyc01g087950.2.1	-7.3426	1.360858	1.41E-12	8.45E-11	5	0	0	125	359	124
Solyc10g018340.1.1	-7.33974	5.636573	2.77E-27	1.09E-24	19	110	13	4075	4487	2561
Solyc10g018320.1.1	-7.32959	-2.23351	0.000827	0.005407	0	0	0	32	3	5
Solyc05g010170.1.1	-7.32058	2.174326	1.27E-10	5.23E-09	8	1	0	665	203	107
Solyc04g072000.2.1	-7.31674	9.305471	8.54E-19	1.19E-16	767	569	97	48187	61166	33178
Solyc02g077580.1.1	-7.30857	1.808764	1.64E-13	1.15E-11	7	0	0	166	398	228
Solyc08g068710.1.1	-7.30147	9.309687	1.01E-22	2.26E-20	700	621	166	60773	50469	29424
Solyc10g084910.1.1	-7.28924	-2.27485	0.000137	0.001181	0	0	0	8	13	18
Solyc03g006230.1.1	-7.28379	-2.30351	0.000371	0.002745	0	0	0	10	5	21
Solyc12g096750.1.1	-7.27835	-0.6647	9.87E-07	1.63E-05	1	0	0	75	56	10
Solyc06g076060.1.1	-7.26812	0.671596	1.54E-12	9.11E-11	0	4	0	127	139	85
Solyc10g012100.1.1	-7.26488	-2.26725	0.000993	0.006304	0	0	0	30	10	1
Solyc11g005860.1.1	-7.25286	2.908265	8.74E-15	7.30E-13	0	24	0	504	634	500
Solyc01g008870.1.1	-7.24291	3.364463	3.21E-26	1.15E-23	3	16	9	1066	645	511
NCRNA_3_252	-7.23152	0.289558	1.98E-10	7.85E-09	0	3	0	64	195	39
Solyc05g052420.1.1	-7.22633	2.113191	1.05E-10	4.37E-09	8	0	1	550	105	226
Solyc07g039570.2.1	-7.21673	6.025605	2.19E-13	1.50E-11	44	155	1	4268	6995	3570
Solyc04g079260.2.1	-7.21411	7.563418	1.88E-27	7.51E-25	151	178	152	5733	25108	13017
Solyc05g021390.2.1	-7.20337	2.2342	1.06E-12	6.45E-11	0	15	0	558	362	135
Solyc03g098740.1.1	-7.19608	10.29519	5.86E-15	5.02E-13	1894	839	160	79244	136986	70186
Solyc12g040760.1.1	-7.17364	-2.35389	0.000128	0.001119	0	0	0	17	13	8
Solyc10g076240.1.1	-7.16793	10.31329	2.95E-24	7.85E-22	299	2509	1170	72411	112114	92799
Solyc06g075100.2.1	-7.15512	5.809542	3.81E-11	1.76E-09	97	30	1	3929	6112	2810

Solyc10g076190.1.1	-7.1493	1.193628	2.54E-10	9.93E-09	5	0	0	57	305	163
NCRNA_11_202	-7.12675	-2.36638	0.000291	0.002243	0	0	0	16	21	3
Solyc11g051150.1.1	-7.10916	3.904838	1.03E-18	1.40E-16	19	19	3	1952	973	394
Solyc10g084550.1.1	-7.09992	-2.38526	0.000215	0.001726	0	0	0	10	25	5
Solyc03g123690.1.1	-7.09047	3.49923	9.68E-25	2.82E-22	15	13	3	1016	758	656
Solyc06g065070.1.1	-7.08966	0.425792	1.00E-09	3.39E-08	3	0	0	57	197	64
Solyc10g078840.1.1	-7.08959	-0.35571	1.08E-05	0.000131	0	2	0	139	19	6
Solyc02g068210.1.1	-7.07523	-2.43217	0.000201	0.001636	0	0	0	17	13	6
Solyc06g083950.1.1	-7.0718	-2.44627	0.000157	0.001324	0	0	0	9	14	12
Solyc01g087280.1.1	-7.05069	4.267887	1.27E-15	1.19E-13	25	32	0	2038	1938	494
Solyc08g068680.2.1	-7.04195	9.063393	7.77E-21	1.45E-18	595	922	115	44060	51686	25252
Solyc08g067640.1.1	-7.03628	3.078134	1.19E-24	3.35E-22	6	7	11	701	539	552
Solyc01g009480.2.1	-7.02415	3.389704	8.44E-24	2.08E-21	4	29	3	868	831	594
Solyc07g040670.1.1	-7.02321	-2.47565	0.000167	0.001395	0	0	0	10	16	9
Solyc06g063430.1.1	-7.01128	-2.45108	0.000965	0.006152	0	0	0	15	22	1
Solyc12g016070.1.1	-7.00602	2.903801	7.35E-16	7.00E-14	0	23	4	754	363	448
Solyc02g094110.1.1	-6.99356	-2.50209	0.000189	0.001557	0	0	0	12	14	8
Solyc06g051090.1.1	-6.99166	0.980396	5.48E-14	4.07E-12	0	4	2	132	154	137
Solyc08g006740.2.1	-6.98406	9.409157	6.04E-26	2.06E-23	901	538	446	49887	68610	35334
Solyc03g036470.1.1	-6.97566	3.980282	6.88E-34	5.28E-31	16	27	7	1179	1450	883
Solyc07g063700.1.1	-6.9547	2.737609	1.18E-18	1.60E-16	0	19	5	328	838	380
Solyc06g069130.2.1	-6.94969	6.56206	2.22E-24	6.00E-22	42	263	49	4518	10973	6041
Solyc03g065120.1.1	-6.94182	-2.51298	0.000466	0.003328	0	0	0	11	22	3
Solyc01g101170.2.1	-6.93656	5.499872	1.52E-15	1.41E-13	27	52	64	7379	1130	1030
Solyc01g109140.2.1	-6.92377	10.61781	4.37E-21	8.57E-19	2331	1860	514	126582	141363	82344
Solyc11g072870.1.1	-6.90886	-2.6108	0.00103	0.006497	0	0	0	3	9	17
Solyc03g113120.2.1	-6.89458	4.29846	4.49E-13	2.91E-11	43	4	4	973	2136	1314
Solyc06g050140.1.1	-6.85621	3.469383	1.65E-27	6.73E-25	5	28	8	601	1064	776
Solyc06g066040.1.1	-6.85471	-2.62504	0.000357	0.002655	0	0	0	12	10	8
Solyc05g007330.1.1	-6.8459	-2.61922	0.000345	0.002589	0	0	0	12	13	6
Solyc07g056600.1.1	-6.83972	3.690712	8.11E-18	9.96E-16	11	39	0	608	1201	1003
Solyc07g040710.2.1	-6.83842	7.070617	3.82E-17	4.32E-15	196	239	21	11528	10741	7242
Solyc12g057080.1.1	-6.82405	7.000414	2.14E-16	2.22E-14	206	164	41	16773	7368	3835
Solyc04g011750.2.1	-6.80389	6.677358	5.08E-12	2.75E-10	210	69	21	13723	4301	3725
Solyc08g082780.1.1	-6.78035	-2.68279	0.000702	0.004711	0	0	0	4	14	11
Solyc12g043030.1.1	-6.77356	0.758393	1.03E-10	4.34E-09	0	4	2	48	289	77
Solyc01g106990.1.1	-6.76264	-2.65225	0.001614	0.009495	0	0	0	12	19	1
Solyc08g065710.1.1	-6.7588	2.459352	9.18E-08	1.98E-06	15	0	0	810	159	180
Solyc02g077290.1.1	-6.73973	8.121786	1.63E-22	3.56E-20	448	396	116	18025	31295	14529
Solyc01g009870.2.1	-6.73833	2.048729	8.41E-19	1.17E-16	2	11	3	252	352	292
Solyc05g010000.1.1	-6.73385	4.495446	2.58E-24	6.92E-22	5	71	21	1140	2574	1412
Solyc11g008950.1.1	-6.73231	2.877819	1.07E-20	1.97E-18	12	4	8	425	618	542
Solyc07g052070.1.1	-6.71892	-2.73983	0.000545	0.003793	0	0	0	7	10	10
Solyc07g041940.2.1	-6.71355	5.185533	3.57E-34	2.82E-31	43	65	28	1755	3294	2843
Solyc07g056610.1.1	-6.71179	1.612322	5.63E-15	4.84E-13	0	8	4	173	238	239
Solyc03g042560.1.1	-6.70945	5.594186	2.54E-17	2.93E-15	63	125	5	3488	4273	2889
Solyc09g074220.1.1	-6.703	-2.73477	0.000517	0.003619	0	0	0	10	12	6
Solyc04g024780.1.1	-6.70189	5.549358	2.16E-35	2.13E-32	61	68	41	2836	4088	3275
Solyc09g091670.2.1	-6.67596	10.05829	4.87E-26	1.68E-23	1328	2327	562	68911	101903	65958
Solyc11g018590.1.1	-6.66957	-2.7635	0.000835	0.005447	0	0	0	14	9	4
Solyc05g050880.2.1	-6.66587	-1.41429	2.42E-05	0.000264	0	1	0	55	18	5
Solyc03g098560.1.1	-6.65931	-0.73955	3.51E-06	4.91E-05	0	2	0	8	97	35
Solyc08g005040.1.1	-6.63737	-0.76204	6.16E-07	1.08E-05	0	2	0	70	30	23
Solyc08g061200.2.1	-6.63438	0.382349	5.54E-11	2.47E-09	1	4	0	78	123	84
Solyc03g118000.1.1	-6.62783	7.049852	1.50E-10	6.07E-09	267	217	5	11765	11481	6264
Solyc05g041190.1.1	-6.62551	-1.42356	2.05E-06	3.08E-05	0	1	0	27	45	11
Solyc10g007580.1.1	-6.62265	1.263769	4.38E-11	2.00E-09	0	11	0	91	287	165
Solyc01g102910.2.1	-6.6222	2.172999	3.04E-08	7.44E-07	0	20	2	33	484	457
Solyc06g082430.1.1	-6.61524	-0.77667	3.72E-07	6.91E-06	0	2	0	19	65	43
Solyc03g005890.1.1	-6.60234	1.536608	5.24E-06	6.99E-05	7	0	2	10	741	49
Solyc10g076220.1.1	-6.5913	2.025037	1.86E-16	1.96E-14	3	6	7	151	388	341
NCRNA_4_197	-6.58618	-2.80808	0.001372	0.008298	0	0	0	12	13	2
Solyc03g031450.2.1	-6.58332	0.637738	4.88E-09	1.44E-07	5	0	0	152	89	86
Solyc09g090900.2.1	-6.57957	5.540785	2.03E-18	2.66E-16	85	85	13	4694	2355	2711
Solyc12g013610.1.1	-6.57335	-0.65977	1.35E-07	2.80E-06	0	0	2	45	37	47
Solyc05g055370.1.1	-6.56432	4.528356	2.54E-07	4.93E-06	68	4	0	2498	1450	1039
Solyc04g025520.1.1	-6.56202	4.018588	1.08E-08	2.98E-07	44	9	1	2426	512	475
Solyc02g079840.1.1	-6.53893	-2.83361	0.001485	0.008868	0	0	0	9	16	2
Solyc05g054650.1.1	-6.52612	5.84724	4.80E-11	2.17E-09	154	39	6	4378	4634	3515
Solyc08g069170.1.1	-6.52537	-0.91002	4.75E-05	0.000477	0	2	0	80	5	20
Solyc01g105070.2.1	-6.50033	9.535575	2.84E-23	6.50E-21	1021	1302	803	86100	37087	34272
Solyc12g009370.1.1	-6.49571	-2.91352	0.001049	0.006592	0	0	0	9	7	7
Solyc11g010210.1.1	-6.48997	5.977595	7.10E-28	3.07E-25	102	67	80	3454	7183	3713
Solyc04g072800.2.1	-6.48176	7.261363	2.48E-32	1.57E-29	146	339	209	13145	13967	7226
Solyc02g076910.2.1	-6.45575	2.763033	1.28E-05	0.000151	8	26	0	71	1859	11
Solyc02g077570.1.1	-6.45029	4.556603	2.39E-09	7.45E-08	68	18	0	977	2835	1550
Solyc03g044830.2.1	-6.43789	2.256473	3.10E-17	3.54E-15	1	18	5	320	443	291
Solyc09g011890.1.1	-6.43508	-1.63302	6.84E-06	8.79E-05	0	1	0	25	15	23
Solyc12g099730.1.1	-6.43164	6.554411	6.23E-11	2.74E-09	263	73	13	8815	7315	4516
Solyc05g052870.2.1	-6.43141	1.117155	6.14E-09	1.78E-07	5	4	0	317	86	57
Solyc12g019630.1.1	-6.42919	2.227765	1.28E-11	6.53E-10	14	4	0	438	416	193
Solyc06g059880.2.1	-6.42883	-0.71833	1.64E-06	2.54E-05	2	4	0	62	29	33
Solyc09g007890.1.1	-6.42437	6.315286	7.35E-29	3.28E-26	131	161	63	6064	7266	4351
Solyc07g056510.2.1	-6.42063	7.658055	7.86E-12	4.17E-10	318	658	19	8164	24888	13181

Solyc03g111280.1.1	-6.3967	1.309936	1.44E-09	4.69E-08	9	0	0	210	207	126
Solyc11g006240.1.1	-6.39013	3.517893	1.62E-08	4.28E-07	38	3	0	597	1326	684
Solyc12g006280.1.1	-6.38914	5.310091	2.57E-34	2.09E-31	54	100	36	3522	3203	1998
Solyc09g066400.1.1	-6.37678	8.794655	8.54E-05	0.00079	1511	17	3	51450	26819	17813
Solyc07g008110.2.1	-6.3735	6.507859	8.71E-30	4.54E-27	74	298	104	6641	9192	4689
Solyc08g068730.1.1	-6.36614	9.511734	1.59E-25	5.04E-23	1161	1786	548	59574	70987	34076
Solyc01g102890.2.1	-6.35975	-0.07239	1.15E-07	2.41E-06	2	2	0	27	68	98
Solyc07g005070.1.1	-6.35792	1.289106	5.76E-12	3.11E-10	5	1	4	162	350	75
Solyc01g095830.2.1	-6.32792	-1.70065	8.40E-06	0.000105	0	1	0	12	40	15
Solyc07g062370.1.1	-6.3204	4.878799	0.000108	0.000969	105	0	0	4637	980	636
Solyc09g090390.1.1	-6.31797	-3.03454	0.001655	0.009694	0	0	0	8	8	5
Solyc04g079240.2.1	-6.31526	6.021939	1.65E-21	3.35E-19	127	152	34	6181	4403	3419
Solyc06g072000.1.1	-6.31083	0.806906	6.05E-11	2.66E-09	0	2	6	95	205	97
Solyc08g066890.2.1	-6.28258	6.199425	7.26E-25	2.18E-22	68	91	191	4571	7829	4185
Solyc10g084110.1.1	-6.28207	2.082097	2.25E-11	1.10E-09	14	4	0	230	453	264
Solyc03g005040.1.1	-6.26916	2.040435	3.32E-14	2.57E-12	11	3	4	241	551	177
Solyc03g019690.1.1	-6.26609	3.06714	9.20E-24	2.25E-21	10	28	7	541	853	482
Solyc06g075570.1.1	-6.25982	-0.38376	7.69E-06	9.74E-05	0	4	0	119	18	20
Solyc06g068830.1.1	-6.25603	1.786839	4.10E-15	3.60E-13	7	8	2	204	257	267
Solyc10g047810.1.1	-6.25034	-0.30412	2.07E-06	3.10E-05	0	3	1	7	111	64
RRNA_11_20	-6.23974	5.159426	6.68E-25	2.03E-22	66	82	33	1188	3936	2800
Solyc07g066550.2.1	-6.23443	8.059151	5.01E-13	3.21E-11	781	322	77	22094	21170	14885
Solyc08g066880.2.1	-6.227	7.928569	1.62E-26	5.97E-24	270	387	566	14065	24792	15421
Solyc09g092600.2.1	-6.22475	2.010843	2.65E-07	5.10E-06	10	7	2	30	1020	61
Solyc03g096430.1.1	-6.22282	0.72637	1.49E-08	3.95E-07	0	10	0	73	101	161
Solyc09g098080.2.1	-6.21724	6.302097	4.27E-08	1.00E-06	75	422	11	492	14502	4557
Solyc04g011490.2.1	-6.21687	2.312225	3.75E-09	1.13E-07	18	3	0	527	455	144
Solyc07g052450.1.1	-6.21651	1.487234	1.22E-09	4.05E-08	0	18	0	172	365	120
Solyc04g080560.2.1	-6.21224	-1.18056	0.000267	0.002084	0	2	0	1	83	21
Solyc04g076730.1.1	-6.21194	6.662418	3.29E-07	6.17E-06	352	69	2	6942	10559	5371
Solyc01g097060.2.1	-6.21101	2.871765	3.00E-20	5.06E-18	10	9	17	659	702	292
Solyc03g020010.1.1	-6.19921	10.49919	1.65E-06	2.55E-05	5350	382	69	156514	104634	56821
Solyc08g080930.1.1	-6.19538	0.822656	2.27E-11	1.11E-09	4	5	0	100	166	119
Solyc09g090480.2.1	-6.18479	-1.74242	0.000308	0.002348	0	0	1	41	7	10
Solyc12g010990.1.1	-6.16763	-1.22318	8.04E-06	0.000101	0	2	0	42	17	26
Solyc06g068890.1.1	-6.15404	3.345023	1.01E-18	1.39E-16	3	49	13	599	895	711
Solyc11g005840.1.1	-6.15243	5.829243	4.74E-31	2.73E-28	102	142	65	3574	5646	3433
Solyc08g066930.1.1	-6.15075	3.064057	1.73E-23	4.13E-21	13	15	16	491	787	556
Solyc11g072920.1.1	-6.14461	4.17618	1.40E-11	7.11E-10	58	28	1	1020	1890	1129
Solyc05g008210.2.1	-6.14102	6.964343	1.10E-27	4.54E-25	233	317	133	9737	10247	7250
Solyc11g072140.1.1	-6.13041	2.180225	9.42E-12	4.92E-10	14	9	0	184	594	269
Solyc01g005160.2.1	-6.11798	3.446524	4.14E-12	2.28E-10	32	25	0	486	1275	706
Solyc04g074780.2.1	-6.11111	5.756031	2.07E-31	1.22E-28	94	100	96	4070	4926	2966
Solyc04g071070.2.1	-6.10909	7.663986	2.83E-11	1.34E-09	645	134	151	25149	7623	9380
Solyc06g048870.1.1	-6.1048	-0.99947	2.67E-05	0.000288	2	0	0	9	60	39
Solyc02g087070.2.1	-6.10037	10.26948	7.87E-09	2.24E-07	4397	964	272	163513	55687	43198
Solyc05g053960.2.1	-6.09663	6.767888	3.07E-18	3.89E-16	290	147	109	8904	5844	7779
Solyc02g085260.1.1	-6.09193	0.706912	3.71E-05	0.000386	7	0	0	150	8	148
Solyc09g011510.2.1	-6.08572	3.257381	2.75E-07	5.27E-06	39	3	0	394	739	865
Solyc01g097380.1.1	-6.08545	4.230892	5.37E-13	3.44E-11	64	10	13	832	1618	1556
Solyc11g007460.1.1	-6.08507	1.711658	3.19E-12	1.80E-10	0	19	3	232	327	173
Solyc08g014090.1.1	-6.07438	1.60869	2.26E-14	1.81E-12	5	11	2	178	239	230
Solyc10g076210.1.1	-6.05886	-0.05025	2.96E-08	7.27E-07	0	6	0	54	96	61
Solyc10g006720.2.1	-6.04156	3.116177	3.57E-10	1.34E-08	0	51	12	212	1188	602
Solyc12g015700.1.1	-6.03707	-0.36092	2.03E-05	0.000227	0	5	0	6	51	96
Solyc11g066140.1.1	-6.03165	3.034239	2.42E-06	3.55E-05	36	0	0	827	804	241
Solyc02g014560.2.1	-6.03051	2.183643	2.57E-13	1.74E-11	0	16	13	342	299	314
Solyc02g068460.1.1	-6.0277	3.782435	1.13E-10	4.72E-09	35	48	0	455	1141	1291
Solyc09g098160.2.1	-6.01006	7.24755	5.75E-16	5.61E-14	483	167	126	10597	16042	7742
Solyc07g056080.1.1	-5.97781	4.42315	6.73E-31	3.79E-28	35	79	25	1196	2334	1294
Solyc02g071130.2.1	-5.96865	4.785741	1.51E-20	2.71E-18	81	62	16	2059	2708	1408
Solyc04g016000.2.1	-5.96404	7.576892	1.35E-18	1.81E-16	555	228	244	14098	16625	11116
Solyc03g097410.1.1	-5.96071	5.520352	1.50E-10	6.07E-09	161	54	26	6813	1534	1306
Solyc05g009120.2.1	-5.9449	7.348629	1.59E-20	2.82E-18	415	456	126	13155	15319	7920
Solyc03g119240.2.1	-5.92919	6.926185	6.74E-20	1.09E-17	284	431	75	8119	12020	6946
Solyc09g005560.2.1	-5.91603	4.466391	3.36E-24	8.83E-22	64	42	24	1446	2168	1293
Solyc02g081890.2.1	-5.90955	3.370289	1.19E-16	1.28E-14	13	28	26	209	1398	762
Solyc11g067000.1.1	-5.90336	3.549847	2.24E-12	1.30E-10	23	57	3	1486	859	265
Solyc03g059050.2.1	-5.89664	6.052692	9.90E-06	0.000121	301	28	1	7694	5188	1923
Solyc06g050950.2.1	-5.88845	1.413946	4.93E-12	2.68E-10	6	12	0	183	238	160
Solyc10g050690.1.1	-5.86321	-0.01369	6.31E-06	8.22E-05	5	0	0	122	79	18
Solyc03g083840.2.1	-5.86194	-1.23576	2.77E-05	0.000298	2	0	0	44	28	16
Solyc02g067600.1.1	-5.83306	4.177107	4.49E-19	6.53E-17	39	79	12	1933	1002	892
Solyc06g068530.1.1	-5.82351	0.84866	1.94E-09	6.20E-08	0	12	2	182	163	58
Solyc06g076170.2.1	-5.81309	0.890992	1.49E-07	3.04E-06	9	0	1	205	91	90
Solyc08g014010.2.1	-5.81244	7.260672	8.75E-25	2.60E-22	245	719	195	9704	16322	8485
Solyc09g074350.1.1	-5.7908	3.355126	1.86E-22	4.02E-20	13	46	18	847	660	650
Solyc12g010670.1.1	-5.78773	2.531746	7.08E-16	6.77E-14	3	26	15	260	538	449
Solyc07g051950.2.1	-5.78504	3.25274	6.87E-14	5.01E-12	37	8	11	571	759	698
Solyc04g078660.1.1	-5.7834	7.63099	2.95E-27	1.15E-24	337	800	335	15945	17616	10201
Solyc08g068240.1.1	-5.78316	0.167205	1.93E-08	4.99E-07	0	5	3	117	68	52
Solyc09g065750.2.1	-5.78069	1.851344	1.49E-10	6.07E-09	15	2	4	331	225	205
Solyc10g083690.2.1	-5.77324	10.64824	3.71E-17	4.22E-15	5349	3472	1457	130075	132717	87501
Solyc04g054950.2.1	-5.77242	8.366088	1.69E-10	6.75E-09	1346	508	127	37560	18326	14359

Solyc11g068940.1.1	-5.74734	6.13281	1.19E-16	1.28E-14	212	246	39	6471	5378	3427
Solyc11g006030.1.1	-5.74388	6.733828	2.27E-13	1.55E-11	400	246	45	7956	10964	5089
Solyc03g036480.1.1	-5.7302	-1.19542	4.96E-06	6.67E-05	0	3	0	40	23	25
Solyc09g008510.1.1	-5.72402	-0.95582	2.31E-05	0.000253	0	0	3	64	33	12
Solyc08g077910.2.1	-5.7239	-0.86796	3.04E-06	4.33E-05	0	4	0	22	56	39
Solyc06g005510.2.1	-5.71522	0.703584	1.46E-10	5.97E-09	1	6	5	89	132	123
Solyc01g106630.2.1	-5.70612	1.417029	0.000956	0.006103	13	1	1	9	748	2
Solyc07g055990.2.1	-5.68949	1.271488	1.14E-11	5.87E-10	8	7	2	123	291	134
Solyc04g025920.2.1	-5.67961	-0.26379	8.59E-08	1.87E-06	2	4	0	71	76	35
Solyc06g073830.1.1	-5.66191	3.77586	1.18E-21	2.41E-19	37	59	11	907	1344	784
Solyc12g005720.1.1	-5.66131	7.751914	2.15E-17	2.51E-15	354	1335	202	20866	13542	11411
Solyc02g092890.1.1	-5.65421	6.398206	2.96E-07	5.61E-06	387	164	3	5050	8355	5230
Solyc01g094260.2.1	-5.65128	4.004037	3.16E-15	2.84E-13	57	27	25	2018	898	531
Solyc01g073860.2.1	-5.64122	2.121425	6.03E-08	1.37E-06	22	6	0	458	202	239
Solyc11g069220.1.1	-5.64059	2.9153	4.71E-13	3.03E-11	7	51	10	949	513	196
Solyc04g017740.1.1	-5.62825	2.084354	9.56E-05	0.000869	24	1	0	65	120	590
Solyc10g086710.1.1	-5.62018	3.979584	1.24E-17	1.48E-15	43	55	24	367	1910	1244
Solyc07g042400.2.1	-5.61678	0.012498	2.79E-07	5.34E-06	0	5	3	42	174	30
Solyc04g064880.2.1	-5.60674	10.92138	6.99E-08	1.56E-06	10001	1801	391	181233	163270	83779
Solyc01g057080.1.1	-5.60525	-0.94789	8.52E-05	0.000788	3	0	0	9	61	41
Solyc01g109320.2.1	-5.59634	5.546862	3.31E-14	2.57E-12	62	312	61	831	5051	4252
Solyc09g065740.1.1	-5.5851	1.086824	1.13E-07	2.38E-06	7	10	0	285	63	86
Solyc05g046110.2.1	-5.57233	2.849018	7.99E-11	3.43E-09	34	12	4	750	647	221
Solyc08g068600.2.1	-5.55747	8.42705	3.04E-17	3.49E-15	908	1844	257	23462	35615	18072
Solyc03g082470.2.1	-5.54842	4.703019	1.06E-23	2.57E-21	37	152	50	2587	1621	1326
Solyc04g072620.1.1	-5.54717	3.809567	1.29E-13	9.08E-12	60	23	17	508	1081	1312
Solyc07g063730.1.1	-5.54578	5.965901	3.31E-21	6.57E-19	53	363	169	3490	6335	3983
Solyc10g083890.1.1	-5.54401	3.002834	4.35E-07	7.91E-06	40	17	0	1135	365	207
Solyc05g025670.1.1	-5.54067	-2.26487	0.00095	0.006076	1	0	0	21	6	11
Solyc02g067770.2.1	-5.53076	0.779278	1.98E-09	6.29E-08	2	2	9	92	210	88
Solyc05g005160.2.1	-5.52167	10.16393	7.15E-21	1.34E-18	3559	3616	2020	105615	89371	54785
Solyc12g100260.1.1	-5.52145	6.876539	1.00E-09	3.39E-08	336	720	19	9363	6721	8200
Solyc11g007450.1.1	-5.51139	2.91257	9.71E-18	1.17E-15	12	30	23	269	836	558
Solyc05g056080.2.1	-5.50576	6.06919	2.55E-21	5.11E-19	151	396	76	5401	6270	3236
Solyc10g050880.1.1	-5.4898	6.388186	4.28E-10	1.57E-08	311	394	15	3916	9203	5532
Solyc11g011330.1.1	-5.4723	5.55341	9.54E-08	2.05E-06	275	23	15	3491	4202	2597
Solyc09g075820.2.1	-5.47185	9.94707	2.12E-23	4.96E-21	2340	4950	1720	73709	98892	48276
Solyc12g100250.1.1	-5.4602	7.438901	5.98E-09	1.74E-07	554	1052	20	11826	14560	11031
Solyc10g007880.2.1	-5.4589	7.043469	1.39E-19	2.16E-17	231	665	337	3860	15815	9819
Solyc12g005710.1.1	-5.44633	-1.87725	7.76E-05	0.000728	0	2	0	15	21	18
Solyc12g006130.1.1	-5.44538	-0.82698	0.000215	0.001731	0	0	4	6	108	22
Solyc10g085860.1.1	-5.44346	4.26385	6.32E-20	1.03E-17	69	77	20	953	2182	1178
Solyc05g054380.1.1	-5.43934	8.813065	5.39E-22	1.13E-19	1468	1380	923	34057	44892	21670
Solyc05g008220.2.1	-5.43126	7.348198	2.49E-19	3.74E-17	594	561	226	12743	12840	9494
Solyc08g065540.2.1	-5.42847	0.412298	1.15E-07	2.42E-06	4	8	0	42	207	65
Solyc09g014910.2.1	-5.4235	4.889315	1.01E-05	0.000123	182	25	0	1550	3811	1495
Solyc11g068370.1.1	-5.41334	3.365186	2.08E-08	5.34E-07	61	12	2	750	863	613
Solyc03g114500.2.1	-5.41308	8.870095	8.26E-24	2.06E-21	1317	1889	999	38533	39788	23983
Solyc03g007890.2.1	-5.41171	4.134575	7.45E-15	6.27E-13	70	67	15	1439	742	1339
Solyc10g012090.1.1	-5.40859	-0.20302	7.62E-06	9.65E-05	6	0	0	75	79	35
Solyc01g007960.2.1	-5.40738	5.22176	4.76E-20	7.83E-18	57	284	53	1685	4300	2389
Solyc06g074140.1.1	-5.37992	7.874475	3.20E-18	4.04E-16	906	864	294	16863	22775	12379
Solyc10g054350.1.1	-5.37973	0.316444	9.26E-08	1.99E-06	0	11	2	56	107	99
Solyc01g005390.2.1	-5.37483	3.232096	2.25E-09	7.06E-08	54	8	8	922	514	522
Solyc07g056690.1.1	-5.36474	-0.95787	9.54E-06	0.000117	0	5	0	53	33	21
Solyc01g006580.2.1	-5.3619	-0.64951	9.91E-06	0.000121	0	0	5	47	62	29
Solyc03g026370.1.1	-5.3595	6.261404	3.20E-19	4.71E-17	214	416	121	8410	5105	3035
Solyc01g098140.2.1	-5.35	6.03534	3.31E-16	3.32E-14	284	224	67	5904	5005	3269
Solyc01g098850.2.1	-5.34794	3.310303	3.94E-18	4.95E-16	29	51	16	950	562	559
Solyc12g100270.1.1	-5.34542	8.324696	9.37E-10	3.19E-08	1203	1867	70	25665	23226	19308
Solyc06g076070.1.1	-5.33944	4.123267	5.67E-21	1.09E-18	59	68	34	1744	992	919
Solyc12g056590.1.1	-5.31916	8.592944	1.81E-23	4.28E-21	1219	1525	890	25145	38847	21631
Solyc10g008150.1.1	-5.31852	2.694697	4.48E-09	1.33E-07	39	11	2	425	544	419
Solyc04g080530.2.1	-5.31811	1.673774	6.71E-08	1.51E-06	0	14	18	385	254	70
Solyc12g099130.1.1	-5.31405	0.939225	4.76E-06	6.43E-05	14	0	0	116	173	123
Solyc08g078650.2.1	-5.30819	7.241886	8.31E-12	4.38E-10	730	553	75	9593	16317	8058
Solyc09g011550.2.1	-5.30436	6.614583	2.14E-18	2.77E-16	407	348	139	6570	8126	6331
Solyc04g040090.2.1	-5.29405	2.351169	4.64E-15	4.04E-13	18	19	11	234	523	357
Solyc03g116700.2.1	-5.29354	9.025648	1.26E-18	1.69E-16	1660	3005	659	37553	44056	31062
Solyc05g056170.2.1	-5.29003	9.593734	2.60E-23	5.99E-21	2414	3030	1960	54085	78843	39518
Solyc05g005700.2.1	-5.27734	6.873962	6.02E-25	1.85E-22	362	487	295	8673	10771	6307
Solyc01g108350.2.1	-5.26527	3.029563	1.06E-09	3.58E-08	3	91	10	840	592	336
Solyc09g014610.2.1	-5.2634	0.27305	5.82E-06	7.67E-05	0	5	7	179	44	28
Solyc04g011740.2.1	-5.24288	1.537405	5.35E-10	1.93E-08	10	4	12	329	205	95
Solyc03g116510.1.1	-5.24085	7.026655	2.67E-16	2.73E-14	566	522	167	12223	9112	6550
Solyc06g063350.1.1	-5.23225	1.388241	3.98E-12	2.20E-10	8	11	7	235	236	106
Solyc09g014220.2.1	-5.21179	-0.38713	1.92E-05	0.000215	6	0	0	53	74	38
Solyc07g052140.2.1	-5.20849	8.706351	1.40E-19	2.16E-17	1144	2180	1124	17051	33495	36459
Solyc01g097560.1.1	-5.20275	-1.04937	1.54E-05	0.000178	1	0	3	36	57	14
Solyc01g057470.1.1	-5.20193	1.148895	0.000129	0.001123	16	2	0	355	103	18
Solyc07g040900.1.1	-5.19891	-0.40816	4.99E-06	6.70E-05	4	3	0	87	38	30
Solyc07g007070.1.1	-5.18195	-0.39595	4.49E-05	0.000455	6	0	0	43	109	24
Solyc06g076300.2.1	-5.17265	5.534657	2.11E-25	6.63E-23	79	246	191	2554	4470	3065
Solyc01g049830.2.1	-5.16977	-0.65552	0.000126	0.0011	5	0	0	66	62	12

Solyc03g096540.2.1	-5.16441	7.090802	0.00073	0.004871	1042	42	3	23069	2597	2445
Solyc06g060240.1.1	-5.16161	-1.39861	4.03E-05	0.000415	0	4	0	26	31	21
Solyc02g083730.2.1	-5.15621	-1.03704	4.70E-05	0.000473	3	0	1	16	53	34
Solyc08g068610.2.1	-5.14808	7.269495	1.55E-12	9.14E-11	766	724	102	11832	13515	8270
Solyc01g016380.2.1	-5.12618	-2.6311	0.0016	0.009432	0	0	1	11	13	6
Solyc02g088840.2.1	-5.11957	-2.75833	0.000591	0.004061	0	1	0	8	9	9
Solyc11g073080.1.1	-5.11855	4.174674	1.38E-26	5.24E-24	56	91	53	1000	1895	1096
Solyc07g006670.1.1	-5.11636	-0.20933	4.17E-07	7.62E-06	0	7	3	46	96	48
Solyc01g073720.2.1	-5.11257	5.091975	7.70E-20	1.23E-17	157	93	90	2066	2547	2524
Solyc02g062030.1.1	-5.10599	-1.96969	0.000233	0.001853	0	0	2	12	33	9
Solyc04g078450.2.1	-5.08588	2.79	1.22E-17	1.46E-15	20	44	17	440	801	327
Solyc01g073670.2.1	-5.08345	2.769954	2.04E-12	1.19E-10	37	14	16	650	445	346
Solyc01g108360.2.1	-5.07971	3.239522	6.03E-12	3.24E-10	10	107	14	933	800	348
Solyc11g007970.1.1	-5.07667	1.509521	5.47E-06	7.26E-05	0	40	2	75	413	171
Solyc10g076510.1.1	-5.07643	10.05887	4.08E-12	2.25E-10	6387	2900	1208	80585	102772	52532
Solyc09g007910.2.1	-5.07401	10.11887	2.18E-18	2.79E-16	3000	7634	2876	118094	77722	44009
Solyc03g097840.2.1	-5.07331	9.282612	4.11E-21	8.12E-19	2368	3104	1508	39229	64828	34246
Solyc01g099660.2.1	-5.06897	6.269428	3.61E-10	1.35E-08	456	107	165	8898	6798	1661
Solyc11g007470.1.1	-5.05347	3.993948	1.65E-20	2.91E-18	42	92	55	547	2037	1025
Solyc05g055270.1.1	-5.05055	1.497594	5.10E-07	9.15E-06	1	3	26	125	291	197
Solyc04g079910.2.1	-5.04413	3.153678	3.50E-12	1.96E-10	39	14	37	371	1421	352
Solyc07g052150.2.1	-5.03918	6.454431	4.76E-21	9.27E-19	264	562	239	4163	7171	7050
NCRNA_6_228	-5.0324	-0.08674	3.67E-06	5.11E-05	0	9	3	115	54	29
Solyc04g015430.2.1	-5.03224	3.809988	3.22E-10	1.23E-08	90	16	28	1514	730	675
Solyc09g014720.1.1	-5.02957	3.38725	1.90E-14	1.54E-12	34	92	9	787	841	611
Solyc02g093310.2.1	-5.02701	5.885034	6.19E-26	2.09E-23	143	369	215	4047	5994	3065
Solyc03g116070.1.1	-5.0263	-2.23137	0.000339	0.00255	0	2	0	14	19	9
Solyc01g005800.2.1	-5.01618	3.775345	2.28E-08	5.79E-07	92	35	11	260	1105	1424
Solyc03g111510.2.1	-5.01405	4.869866	3.35E-20	5.58E-18	146	99	71	1884	3368	1376
Solyc08g080290.2.1	-5.00408	0.967073	3.65E-09	1.10E-07	4	20	2	152	147	114
Solyc12g100240.1.1	-4.99507	3.982476	3.58E-07	6.68E-06	87	100	0	1063	985	1190
Solyc02g062320.2.1	-4.99203	-0.85021	0.000257	0.002016	0	2	4	57	66	3
Solyc01g106510.2.1	-4.99135	1.19856	7.10E-08	1.58E-06	11	7	7	142	60	230
Solyc04g074360.1.1	-4.99131	-0.81076	8.24E-05	0.000767	3	3	0	16	111	12
Solyc07g065160.2.1	-4.98897	2.676243	1.49E-15	1.39E-13	20	47	14	554	621	249
Solyc07g008690.2.1	-4.98574	4.736118	1.02E-24	2.95E-22	98	155	71	1443	2302	1920
Solyc05g005090.2.1	-4.97464	-1.50911	0.000372	0.002752	0	3	1	48	20	4
Solyc03g058890.2.1	-4.97306	2.408267	1.66E-14	1.35E-12	21	36	9	380	511	274
Solyc10g045100.1.1	-4.97234	-0.81906	6.75E-05	0.000645	0	5	2	69	47	7
Solyc04g007620.1.1	-4.97183	2.678558	3.03E-17	3.48E-15	18	43	20	406	690	331
Solyc04g078460.2.1	-4.94299	8.374887	2.50E-12	1.43E-10	2149	740	601	24332	28959	18551
Solyc08g007230.1.1	-4.94181	3.556119	1.47E-08	3.94E-07	89	22	9	1266	638	550
Solyc06g050540.1.1	-4.9359	-1.54974	9.48E-05	0.000863	0	3	1	11	26	30
Solyc01g049820.2.1	-4.93548	1.244256	2.38E-09	7.44E-08	9	10	9	240	247	51
Solyc02g076830.1.1	-4.92324	1.603139	2.79E-08	6.90E-07	0	24	18	278	234	140
Solyc09g011520.2.1	-4.92314	5.115381	4.06E-06	5.58E-05	286	60	3	1862	3585	2160
Solyc08g068630.2.1	-4.9227	-0.54036	1.34E-06	2.14E-05	1	3	4	45	87	23
Solyc11g010860.1.1	-4.91834	0.371363	0.00037	0.002742	12	0	0	17	235	60
Solyc09g089910.1.1	-4.91006	5.852703	2.21E-24	6.00E-22	199	390	169	4207	5281	3105
Solyc07g048070.2.1	-4.90835	7.376216	4.65E-10	1.70E-08	868	1170	92	6306	20591	10516
Solyc09g075870.1.1	-4.90017	2.809779	3.09E-09	9.44E-08	30	57	7	973	327	179
Solyc12g062940.1.1	-4.89715	3.654546	1.57E-12	9.29E-11	86	28	23	758	1186	782
Solyc08g036660.2.1	-4.87545	4.331288	1.17E-18	1.59E-16	82	57	100	1481	1294	1380
Solyc03g094020.2.1	-4.86914	1.141645	4.78E-08	1.11E-06	12	1	11	134	230	120
Solyc08g007790.2.1	-4.86889	9.578479	2.18E-18	2.79E-16	2629	4028	3217	79421	52402	31749
Solyc03g115610.2.1	-4.86277	7.470911	8.01E-12	4.22E-10	1181	707	193	11677	18204	9362
Solyc02g094180.2.1	-4.85353	2.439526	1.69E-11	8.45E-10	26	35	10	160	639	396
Solyc06g072880.1.1	-4.84857	-2.95866	0.001585	0.009356	0	1	0	4	10	8
Solyc10g055740.1.1	-4.848	3.627345	4.93E-19	7.13E-17	32	96	46	501	1409	822
Solyc02g090490.2.1	-4.84565	9.073128	1.43E-18	1.91E-16	1500	4695	1619	48090	36959	28694
Solyc01g006950.2.1	-4.84476	8.246153	4.51E-18	5.60E-16	1245	2351	608	20585	30823	15709
Solyc12g008960.1.1	-4.84142	7.988749	3.22E-20	5.40E-18	752	2189	732	15549	26928	13840
Solyc04g058170.1.1	-4.83532	3.864772	2.25E-17	2.62E-15	71	99	25	738	1587	891
Solyc11g072930.1.1	-4.83396	2.195444	1.30E-09	4.27E-08	8	62	4	305	446	250
Solyc01g080010.2.1	-4.83214	8.0899	9.59E-07	1.59E-05	2299	494	138	34346	13685	9238
Solyc10g038080.1.1	-4.83016	7.325631	5.39E-19	7.67E-17	326	1175	793	14094	13925	7033
Solyc01g009320.1.1	-4.82748	3.491462	3.39E-06	4.76E-05	102	14	2	819	993	618
Solyc02g080090.1.1	-4.82609	3.264404	1.47E-06	2.31E-05	72	36	5	1477	252	240
Solyc03g096650.1.1	-4.81928	1.934882	5.00E-11	2.25E-09	7	18	26	164	329	307
Solyc02g079440.1.1	-4.81665	8.056783	2.94E-07	5.59E-06	2291	368	187	27108	20660	10058
NCRNA_9_219	-4.80198	-1.68333	0.000559	0.00387	3	0	0	17	29	17
Solyc08g007780.2.1	-4.79996	1.754323	1.28E-08	3.47E-07	23	9	8	390	227	106
Solyc10g081970.1.1	-4.79681	7.486229	1.33E-18	1.78E-16	480	1778	496	11518	17980	9890
Solyc05g054890.2.1	-4.79406	4.520356	1.97E-22	4.23E-20	95	165	62	1667	2584	942
Solyc02g068470.1.1	-4.7925	4.649227	2.25E-05	0.000247	211	80	0	1116	2340	1887
Solyc11g066890.1.1	-4.78222	7.262984	4.22E-20	6.98E-18	433	899	815	13399	11650	7756
Solyc12g096920.1.1	-4.78065	4.273071	1.19E-23	2.86E-21	53	168	68	1219	1766	1182
Solyc09g056290.1.1	-4.77204	-2.43714	0.000873	0.00566	0	2	0	10	19	7
Solyc09g011540.2.1	-4.77126	3.919892	2.05E-06	3.08E-05	137	25	6	1386	483	1093
Solyc03g025490.2.1	-4.7681	-2.04562	0.000452	0.003251	0	3	0	16	30	5
Solyc09g091000.2.1	-4.75454	5.727216	1.50E-07	3.05E-06	249	554	14	6373	2267	2254
Solyc08g074710.1.1	-4.75255	-0.48783	0.000249	0.00196	5	4	0	6	78	65
Solyc03g116440.1.1	-4.74612	2.100518	4.21E-12	2.31E-10	10	28	24	395	225	255
Solyc03g096660.1.1	-4.74447	3.040545	6.42E-13	4.05E-11	11	76	42	320	734	673

Solyc08g068390.2.1	-4.74426	9.584049	1.82E-19	2.78E-17	2034	6723	3062	61850	64524	39158
Solyc04g007390.2.1	-4.73678	5.615632	1.07E-13	7.63E-12	346	191	84	4071	3989	2476
Solyc04g011480.2.1	-4.73316	2.555975	8.11E-09	2.30E-07	43	30	4	590	487	206
Solyc08g023510.1.1	-4.7248	6.977975	6.08E-10	2.17E-08	947	586	95	8668	12258	6672
Solyc06g065550.1.1	-4.71874	-0.76083	4.12E-05	0.000422	0	3	5	67	39	17
Solyc09g090970.2.1	-4.71846	5.531908	7.50E-07	1.28E-05	374	188	22	6700	1559	1281
Solyc11g011500.1.1	-4.71587	2.741248	3.51E-12	1.96E-10	45	32	12	492	518	401
Solyc02g092120.2.1	-4.71306	4.423453	1.08E-20	1.97E-18	117	103	72	1645	1680	1229
Solyc12g056960.1.1	-4.71055	7.265834	1.43E-14	1.17E-12	894	703	468	16164	8717	7205
Solyc10g079380.1.1	-4.70109	-2.32695	0.000787	0.005192	0	0	2	16	15	7
Solyc11g007390.1.1	-4.69821	5.446152	8.08E-19	1.13E-16	225	255	137	1670	5407	2706
Solyc07g053420.2.1	-4.69684	4.777888	6.65E-19	9.43E-17	115	261	54	1667	3235	1297
Solyc01g102680.2.1	-4.68978	4.196804	1.17E-20	2.11E-18	101	99	57	1192	1816	1002
Solyc05g053620.2.1	-4.68222	-0.21795	2.16E-05	0.000239	7	0	3	27	104	56
Solyc04g005050.1.1	-4.682	8.752775	1.11E-16	1.20E-14	1965	3814	915	28615	41317	23977
Solyc09g011590.2.1	-4.68117	9.831865	9.34E-14	6.74E-12	4944	7487	1322	62219	85780	50093
Solyc10g076680.1.1	-4.67951	3.395776	1.35E-05	0.000159	107	5	6	974	1004	359
Solyc09g008740.1.1	-4.66666	4.192401	9.80E-22	2.02E-19	49	142	95	1549	1740	745
Solyc10g076670.1.1	-4.66639	5.10991	3.56E-07	6.64E-06	333	48	25	3274	3159	1199
Solyc12g006840.1.1	-4.65955	7.000313	4.46E-16	4.42E-14	513	1346	247	8502	13087	6618
Solyc03g006700.2.1	-4.65564	7.012621	9.60E-14	6.91E-12	780	593	417	13840	6086	6498
Solyc08g007160.2.1	-4.64859	5.904516	3.59E-23	8.12E-21	236	450	241	3621	6428	3206
Solyc09g009980.1.1	-4.64368	1.384072	7.59E-09	2.17E-07	2	28	14	109	314	157
Solyc06g010030.2.1	-4.62957	6.75567	2.87E-08	7.07E-07	979	271	114	11070	8093	4107
Solyc02g069540.1.1	-4.62659	0.603555	8.43E-07	1.42E-05	0	14	11	119	99	94
Solyc03g119370.1.1	-4.62556	4.978385	6.87E-07	1.18E-05	299	95	8	2507	2839	1507
Solyc02g080890.2.1	-4.62095	7.881585	1.48E-17	1.74E-15	1202	1853	621	14432	24207	13094
Solyc01g073830.1.1	-4.62008	0.978403	9.94E-07	1.64E-05	0	28	8	145	124	133
Solyc08g005100.2.1	-4.61947	3.980264	1.55E-22	3.40E-20	63	122	64	1244	1454	743
Solyc01g059960.1.1	-4.61677	-0.42189	0.000146	0.001243	6	0	3	102	37	15
Solyc04g074830.1.1	-4.61674	4.333055	1.70E-18	2.25E-16	62	237	52	1126	2078	1199
Solyc09g090990.2.1	-4.60709	6.426526	9.22E-10	3.15E-08	255	1295	69	7018	5783	5103
Solyc01g105080.2.1	-4.5958	0.126342	1.88E-05	0.000212	10	2	2	120	47	52
Solyc03g120570.2.1	-4.5956	4.258625	2.88E-09	8.85E-08	140	36	76	2461	1294	395
Solyc05g005070.2.1	-4.59071	6.703043	9.50E-18	1.15E-15	561	812	271	7723	9213	5529
Solyc07g008520.2.1	-4.58528	3.410531	2.61E-16	2.67E-14	36	96	45	485	808	886
Solyc01g086680.2.1	-4.58434	6.327423	1.03E-07	2.21E-06	777	204	56	5030	8454	4227
Solyc10g079900.1.1	-4.58319	-1.91557	0.000632	0.004308	0	4	0	21	25	8
Solyc10g008170.2.1	-4.58291	-0.24078	0.00076	0.00504	9	0	1	127	32	14
Solyc02g077350.1.1	-4.57893	-0.07182	3.98E-06	5.48E-05	0	10	6	74	100	35
Solyc08g062810.1.1	-4.57443	0.794501	1.29E-07	2.69E-06	3	13	12	49	252	96
Solyc09g090130.2.1	-4.57165	7.230461	9.23E-20	1.47E-17	482	1474	628	9477	14082	8841
Solyc02g068220.1.1	-4.5714	-0.31311	2.98E-05	0.000318	0	3	9	45	69	53
Solyc05g056420.2.1	-4.56544	3.837268	1.89E-17	2.22E-15	87	57	64	959	1389	763
Solyc03g111540.1.1	-4.55052	4.365522	2.32E-20	4.01E-18	81	222	58	1416	1943	1110
Solyc02g079530.2.1	-4.54551	5.803076	1.02E-10	4.30E-09	489	206	83	3777	5734	2783
Solyc08g074960.2.1	-4.54481	4.354402	3.80E-11	1.76E-09	13	290	97	1464	1951	1040
Solyc04g008250.1.1	-4.52952	1.047484	3.44E-05	0.000362	8	8	15	37	493	18
Solyc08g081700.1.1	-4.52462	4.618641	2.37E-21	4.79E-19	77	227	124	1208	3108	1252
Solyc02g082950.2.1	-4.51011	6.297331	1.16E-20	2.11E-18	372	661	299	5900	7192	3949
Solyc01g111250.2.1	-4.50432	5.479944	5.12E-14	3.83E-12	349	216	104	2458	5316	2248
Solyc05g053600.2.1	-4.49711	10.07618	1.73E-16	1.84E-14	4733	10667	3699	100322	87628	44843
Solyc11g007980.1.1	-4.49674	5.532517	4.59E-07	8.29E-06	48	917	56	979	5538	3596
Solyc08g013960.1.1	-4.49331	2.744194	7.09E-13	4.41E-11	46	38	21	470	742	284
Solyc02g062550.2.1	-4.49329	5.979174	1.11E-13	7.84E-12	458	432	116	3532	6140	3924
Solyc08g029000.2.1	-4.49268	12.31868	2.30E-06	3.40E-05	54231	12472	2464	466367	360435	250270
Solyc02g062870.2.1	-4.4883	0.087194	0.000659	0.004462	0	23	0	138	54	28
Solyc01g073660.2.1	-4.48792	8.794417	7.28E-17	8.12E-15	2514	3320	1539	38685	36678	20151
Solyc03g094120.2.1	-4.48546	4.323907	2.43E-11	1.18E-09	113	198	39	434	3051	1165
Solyc05g055030.1.1	-4.48516	3.273483	1.08E-08	2.98E-07	93	18	22	803	667	544
Solyc01g096430.2.1	-4.47862	9.00806	6.10E-09	1.77E-07	4698	2010	795	55770	31237	20992
Solyc02g076930.1.1	-4.47708	4.229615	1.70E-10	6.78E-09	164	92	27	1889	1180	854
Solyc04g040130.1.1	-4.47394	7.930083	6.03E-11	2.66E-09	598	4050	431	14297	19636	17008
Solyc11g012120.1.1	-4.47204	0.48517	3.88E-05	0.0004	6	6	10	12	84	170
Solyc09g065560.2.1	-4.46772	8.503376	3.62E-16	3.61E-14	2237	2250	1402	32541	28852	16332
Solyc12g011100.1.1	-4.46689	1.845256	1.65E-08	4.33E-07	2	52	18	212	326	226
Solyc02g069060.2.1	-4.46525	3.720747	1.28E-19	2.01E-17	43	117	75	798	1524	628
Solyc12g009160.1.1	-4.45722	3.943389	1.02E-18	1.40E-16	78	117	68	1463	1107	688
Solyc03g113580.1.1	-4.4566	-1.1825	0.000122	0.001068	0	7	1	20	57	19
Solyc02g069300.1.1	-4.4459	-0.67825	0.000491	0.003472	0	13	0	21	68	43
Solyc12g013640.1.1	-4.44147	0.724669	1.39E-05	0.000163	17	2	4	175	82	78
Solyc05g055730.2.1	-4.4399	4.296709	2.15E-18	2.77E-16	125	95	95	1545	1767	937
Solyc01g099210.2.1	-4.43345	-1.45084	0.000468	0.003341	0	7	0	17	25	29
Solyc10g008730.2.1	-4.42471	5.455714	2.14E-20	3.72E-18	248	388	143	3235	4166	2142
Solyc07g053270.2.1	-4.42382	7.700828	8.40E-16	7.95E-14	1304	1601	674	15032	23443	8278
Solyc10g079690.1.1	-4.41345	1.090235	1.72E-08	4.49E-07	13	18	6	97	295	95
Solyc12g017700.1.1	-4.41002	2.629744	4.51E-09	1.34E-07	56	20	18	595	337	331
Solyc02g088160.2.1	-4.40981	3.149071	1.67E-10	6.71E-09	17	94	60	1066	651	205
Solyc02g089920.1.1	-4.40609	0.88017	1.15E-08	3.13E-07	11	15	6	119	133	125
Solyc11g066800.1.1	-4.398	6.190066	5.52E-20	9.03E-18	385	522	372	3955	7917	4147
Solyc08g074970.1.1	-4.39625	6.243377	5.73E-17	6.43E-15	501	579	238	5263	7649	3686
Solyc03g005480.2.1	-4.39588	3.68691	2.05E-08	5.25E-07	131	52	14	978	729	887
Solyc11g007480.1.1	-4.39435	1.23799	1.28E-09	4.20E-08	11	16	14	106	250	152
Solyc02g092820.2.1	-4.38956	5.231718	9.74E-11	4.15E-09	304	220	95	831	5667	2190

Solyc10g024490.1.1	-4.38376	2.047644	2.16E-06	3.22E-05	43	3	13	218	308	318
Solyc06g066590.2.1	-4.38363	6.092389	6.80E-07	1.17E-05	729	285	30	4860	6184	3625
NCRNA_8_259	-4.38224	0.38092	1.22E-07	2.55E-06	8	8	6	76	111	84
Solyc12g056420.1.1	-4.37432	1.090277	1.81E-08	4.71E-07	12	8	15	148	245	82
Solyc10g005620.2.1	-4.37	5.052188	3.07E-15	2.77E-13	272	179	97	2044	2859	2104
Solyc04g074680.1.1	-4.36816	6.203279	1.86E-13	1.28E-11	409	864	158	3270	9770	3732
Solyc08g080530.1.1	-4.36628	1.597667	3.19E-06	4.51E-05	2	62	6	100	356	205
Solyc04g077300.2.1	-4.36522	5.376756	7.58E-19	1.07E-16	96	474	264	2573	4241	2243
Solyc01g095860.2.1	-4.36389	0.219994	2.50E-06	3.65E-05	3	19	2	109	73	57
Solyc08g021880.1.1	-4.35251	1.894989	1.46E-09	4.74E-08	23	42	6	284	275	215
Solyc01g010420.2.1	-4.35079	-1.7535	0.000896	0.005782	3	0	1	22	22	14
Solyc01g111570.2.1	-4.33802	6.368102	4.38E-18	5.48E-16	457	538	482	7277	6839	3609
Solyc05g008360.1.1	-4.33743	0.001872	1.24E-05	0.000147	0	12	8	82	53	64
Solyc02g078530.2.1	-4.33675	4.935237	1.88E-18	2.47E-16	200	282	88	1921	2957	1720
Solyc12g005680.1.1	-4.32165	1.168312	7.19E-09	2.06E-07	16	19	6	142	196	135
Solyc01g090890.2.1	-4.31865	0.649885	0.000451	0.003244	11	7	8	22	390	6
Solyc07g063710.1.1	-4.31386	4.454116	1.28E-07	2.66E-06	6	323	160	921	2435	1417
Solyc02g078660.2.1	-4.3126	5.569135	5.29E-19	7.62E-17	189	534	237	2143	6050	2502
Solyc03g121190.2.1	-4.30993	2.953834	5.86E-10	2.09E-08	65	65	11	566	517	502
Solyc09g011980.1.1	-4.30962	0.452082	6.81E-07	1.18E-05	9	15	2	70	167	66
Solyc11g071470.1.1	-4.3083	1.717226	9.53E-09	2.65E-07	24	17	16	100	392	225
Solyc02g086270.2.1	-4.30448	5.764942	7.50E-20	1.20E-17	277	579	223	3635	5108	2956
Solyc03g043880.2.1	-4.30358	5.852638	2.97E-09	9.12E-08	607	207	112	5307	4794	2331
Solyc07g055690.1.1	-4.30114	6.187608	2.62E-07	5.07E-06	835	256	60	5377	6401	3808
Solyc02g067780.2.1	-4.30008	2.991692	1.69E-10	6.75E-09	75	41	19	467	838	429
Solyc09g008560.2.1	-4.29712	6.446048	3.94E-13	2.57E-11	680	529	329	9159	5655	3491
Solyc07g005090.2.1	-4.29095	8.04658	5.93E-13	3.76E-11	2170	1873	710	14496	29899	14024
Solyc03g071850.1.1	-4.28251	3.48768	2.46E-07	4.80E-06	123	58	7	977	839	545
Solyc10g083290.1.1	-4.28213	9.093727	3.20E-11	1.49E-09	4316	5152	914	34280	60407	26233
Solyc07g049460.2.1	-4.28076	1.41344	1.40E-05	0.000164	31	10	2	77	354	163
Solyc12g099260.1.1	-4.28027	10.25323	3.73E-12	2.08E-10	9986	6017	5137	120483	82236	53312
Solyc06g035960.2.1	-4.27854	10.02501	2.60E-11	1.24E-09	9361	6809	2305	79710	98603	47926
Solyc01g006050.2.1	-4.2645	1.376602	5.72E-06	7.56E-05	2	48	11	51	386	155
Solyc06g069650.2.1	-4.26378	3.326616	1.48E-08	3.95E-07	7	192	44	549	923	652
Solyc10g085010.1.1	-4.26315	9.039297	1.17E-12	7.10E-11	2901	7190	1132	34414	56666	24934
Solyc01g106610.2.1	-4.26291	6.766907	0.000283	0.002187	1473	94	41	14909	3634	3500
Solyc08g078700.2.1	-4.26033	3.959797	3.31E-08	8.01E-07	153	33	66	1285	499	1198
Solyc08g028780.1.1	-4.26031	6.563394	1.26E-11	6.46E-10	844	654	214	8568	7144	4297
Solyc06g065010.2.1	-4.25934	4.783254	1.27E-19	2.01E-17	172	242	121	2344	1910	1470
Solyc06g061170.2.1	-4.2549	7.195272	6.47E-14	4.73E-12	1042	1145	586	14965	9188	6370
Solyc09g011870.1.1	-4.25158	9.50961	2.58E-16	2.65E-14	2762	8958	3864	60724	64236	32126
Solyc07g008810.1.1	-4.24404	1.288253	0.000109	0.000971	1	59	3	51	378	134
Solyc03g005510.1.1	-4.24336	-0.12142	0.000956	0.006103	11	4	0	9	147	53
Solyc10g055200.1.1	-4.2286	8.005825	1.80E-13	1.24E-11	1742	2787	717	23164	18715	12143
Solyc08g075550.2.1	-4.22541	7.393634	5.36E-09	1.57E-07	1768	894	289	6990	19191	10569
Solyc12g099140.1.1	-4.21716	3.266518	8.48E-08	1.85E-06	108	15	33	729	772	522
Solyc02g079500.2.1	-4.21499	4.882135	7.87E-11	3.39E-09	71	498	131	698	4490	1629
Solyc03g111730.2.1	-4.21184	4.271987	1.78E-16	1.88E-14	92	211	104	779	2819	874
Solyc05g015850.2.1	-4.21143	7.38809	1.45E-11	7.33E-10	1566	1145	396	11728	15938	8474
Solyc09g074890.1.1	-4.2103	0.913543	8.51E-07	1.43E-05	16	9	9	201	83	92
Solyc11g007490.1.1	-4.20612	6.345956	1.17E-08	3.19E-07	876	494	94	5411	8390	3966
Solyc01g097240.2.1	-4.20013	9.69502	7.25E-13	4.49E-11	4012	12486	2359	39486	90145	50472
Solyc02g085010.1.1	-4.19653	6.807569	9.14E-17	9.99E-15	702	1171	454	8852	9103	5734
Solyc03g020050.2.1	-4.19347	9.908582	0.001193	0.007369	13658	291	681	153504	18843	20328
Solyc03g096190.1.1	-4.18975	2.979297	8.31E-11	3.56E-09	41	121	17	343	785	546
Solyc02g036480.1.1	-4.18831	8.299215	6.50E-13	4.09E-11	2222	3668	763	23298	30758	14430
Solyc07g045000.2.1	-4.18709	2.103296	5.52E-08	1.26E-06	4	63	33	190	607	189
Solyc11g008820.1.1	-4.18689	2.317407	1.14E-09	3.81E-08	43	43	12	237	532	306
Solyc04g072920.2.1	-4.18421	4.705289	2.71E-16	2.75E-14	128	370	90	1268	3281	1306
Solyc11g063530.1.1	-4.18283	-1.32489	0.001174	0.007269	0	1	6	13	52	20
Solyc06g008620.1.1	-4.17759	8.415602	0.000658	0.004457	4901	355	88	50193	10802	8350
Solyc06g076320.1.1	-4.17695	2.533386	6.88E-07	1.19E-05	64	35	7	637	460	152
Solyc09g007520.2.1	-4.1733	7.777837	8.15E-09	2.31E-07	2462	817	547	20037	17357	9289
Solyc02g094190.2.1	-4.16303	7.447828	4.13E-14	3.14E-12	1453	1062	822	11477	18187	8480
Solyc03g020030.2.1	-4.16118	5.272683	4.22E-05	0.000431	543	56	23	3905	1547	2199
Solyc10g017570.2.1	-4.16101	6.065396	8.71E-17	9.55E-15	499	579	280	4100	6732	3637
Solyc12g088040.1.1	-4.15711	6.116084	2.77E-11	1.32E-09	643	584	147	6195	5078	3275
Solyc05g016310.1.1	-4.15666	7.147525	1.48E-12	8.85E-11	1170	1383	359	8492	16274	6685
Solyc02g083850.2.1	-4.15602	5.807962	3.53E-13	2.32E-11	510	355	206	4937	4012	2750
Solyc01g073890.2.1	-4.15538	5.925626	3.36E-14	2.59E-12	174	1055	264	3936	5871	3264
Solyc01g105630.2.1	-4.15451	3.039271	5.08E-12	2.75E-10	59	98	20	557	745	447
Solyc05g009410.1.1	-4.15348	-0.71158	0.000216	0.001732	6	0	4	31	85	20
Solyc01g007000.2.1	-4.1514	4.723159	5.96E-12	3.21E-10	138	449	44	1890	2523	1286
Solyc10g079810.1.1	-4.1468	5.411528	1.04E-16	1.13E-14	352	313	178	3220	3911	2019
Solyc10g005440.1.1	-4.1459	0.143357	0.00159	0.00938	17	0	0	80	136	31
Solyc08g068770.1.1	-4.13833	8.929279	1.48E-11	7.49E-10	3763	5428	1240	47245	38740	18204
Solyc06g005520.2.1	-4.1278	7.64011	1.58E-15	1.46E-13	1509	1738	880	13912	18046	10578
Solyc09g030370.2.1	-4.12229	1.655757	1.02E-06	1.69E-05	36	11	10	310	160	163
Solyc04g008100.1.1	-4.12093	7.231857	2.93E-06	4.19E-05	1833	902	73	8775	17570	7058
Solyc12g057140.1.1	-4.11476	6.372857	5.60E-16	5.49E-14	517	596	596	3964	10650	4041
Solyc02g065190.2.1	-4.11324	5.16643	7.78E-10	2.71E-08	347	194	148	712	4395	2695
Solyc08g013970.1.1	-4.11132	3.95464	3.37E-15	3.00E-13	82	92	143	891	1959	650
Solyc02g070910.1.1	-4.10739	5.04219	4.35E-19	6.35E-17	132	463	185	1665	3430	1997
Solyc11g008840.1.1	-4.10721	-1.55667	0.000372	0.002752	2	5	0	29	21	16

Solyc03g082530.1.1	-4.1003	5.485005	1.75E-11	8.68E-10	446	379	88	3193	3993	2342
Solyc01g096560.1.1	-4.08648	3.168292	7.29E-10	2.56E-08	96	37	37	473	797	606
Solyc05g010020.2.1	-4.08549	5.475672	9.66E-11	4.12E-09	383	544	63	2768	4319	2449
Solyc03g123380.1.1	-4.0793	2.584367	4.61E-11	2.09E-09	17	68	54	277	529	437
Solyc09g097960.2.1	-4.07851	5.16415	1.16E-10	4.79E-09	114	743	99	1325	4765	1966
Solyc06g066310.2.1	-4.07452	6.510632	1.76E-13	1.22E-11	565	851	583	10054	4413	4013
Solyc06g070990.2.1	-4.07158	3.851365	1.50E-10	6.08E-09	140	140	27	1244	964	762
Solyc06g062540.2.1	-4.07059	2.152561	0.000503	0.003536	8	109	8	34	1106	46
Solyc08g022240.1.1	-4.06638	1.668915	6.90E-09	1.98E-07	28	27	12	274	278	127
Solyc02g021680.2.1	-4.06587	0.019481	4.72E-05	0.000474	0	20	6	48	112	57
Solyc01g080570.2.1	-4.06175	7.446584	8.94E-07	1.50E-05	2376	468	279	12407	16378	8636
Solyc07g006420.1.1	-4.06136	5.035313	3.51E-10	1.32E-08	245	494	43	1483	3428	2111
Solyc02g077370.1.1	-4.05926	6.818644	5.92E-08	1.35E-06	1251	977	110	7315	10504	6221
Solyc05g041920.2.1	-4.05723	6.034051	2.81E-13	1.88E-11	565	695	184	4803	6039	3190
Solyc08g005280.1.1	-4.0527	2.035089	2.21E-09	6.95E-08	26	49	19	129	456	291
Solyc05g052280.2.1	-4.05217	5.864791	6.48E-12	3.48E-10	210	1009	279	1700	6699	4161
Solyc12g057060.1.1	-4.05142	8.959768	5.24E-09	1.54E-07	5358	3515	1241	54532	30583	18407
Solyc06g035710.1.1	-4.04927	-1.51062	0.000813	0.00533	3	4	0	14	48	13
Solyc12g056600.2.1	-4.04829	7.322317	2.04E-07	4.05E-06	2058	349	487	9724	18492	7256
Solyc05g008910.2.1	-4.04284	1.198479	2.71E-05	0.000292	12	30	12	369	60	33
Solyc01g107390.2.1	-4.04009	6.45696	9.36E-07	1.56E-05	1171	350	125	9482	5916	3051
Solyc10g081560.1.1	-4.03722	5.774317	3.93E-12	2.18E-10	517	539	139	3402	6161	2507
Solyc10g085030.1.1	-4.03444	4.483335	2.55E-14	2.01E-12	128	357	72	1992	1804	946
Solyc04g055000.2.1	-4.0208	1.195883	1.39E-05	0.000163	0	33	25	132	258	110
NCRNA_12_259	-4.01332	0.104612	3.02E-06	4.30E-05	9	12	3	80	82	58
NCRNA_12_273	-4.01332	0.104612	3.02E-06	4.30E-05	9	12	3	80	82	58
Solyc02g062670.2.1	-4.01132	5.97847	2.42E-11	1.17E-09	661	482	190	5622	4664	2899
Solyc06g074990.1.1	-4.01102	2.437192	1.91E-11	9.42E-10	37	53	33	295	690	231
Solyc09g074490.2.1	-4.00551	-1.10009	0.000625	0.004265	3	8	0	18	25	44
Solyc03g119970.2.1	-4.00061	8.795504	7.95E-12	4.20E-10	4309	3019	2073	20790	56157	22284
Solyc01g097390.2.1	-3.9983	3.276217	7.13E-11	3.10E-09	97	74	37	392	1069	622
Solyc09g014460.1.1	-3.99716	-0.41258	0.000113	0.001004	0	16	4	62	60	33
Solyc06g036420.1.1	-3.98471	2.210702	3.31E-10	1.26E-08	38	47	21	258	354	329
Solyc12g007040.1.1	-3.98469	3.23233	1.11E-13	7.84E-12	71	110	41	705	770	493
Solyc02g082450.2.1	-3.98303	8.197409	1.85E-07	3.70E-06	3970	1225	538	20078	29117	14136
Solyc01g103580.2.1	-3.97921	5.106449	4.08E-16	4.06E-14	260	342	194	3432	2201	1483
Solyc09g005840.1.1	-3.97804	2.709443	2.93E-11	1.38E-09	57	61	30	553	460	335
Solyc03g043900.1.1	-3.9762	2.583472	4.13E-10	1.52E-08	26	99	31	203	613	444
Solyc04g079730.1.1	-3.97605	5.525576	1.05E-11	5.44E-10	443	397	182	5065	2608	1789
Solyc08g081550.2.1	-3.97251	8.245354	1.61E-10	6.50E-09	2751	3626	775	28839	24998	10963
Solyc01g103870.1.1	-3.97189	2.308985	1.64E-08	4.32E-07	52	39	17	489	310	218
Solyc11g068440.1.1	-3.97177	5.265136	2.91E-16	2.93E-14	344	362	167	2386	3930	1978
Solyc06g073760.2.1	-3.97008	5.055541	5.81E-16	5.65E-14	231	464	130	1511	3755	1954
Solyc06g076020.2.1	-3.95992	7.988101	1.11E-08	3.04E-07	1831	4499	343	20911	23378	10347
Solyc08g067390.2.1	-3.95967	6.022561	6.43E-16	6.19E-14	510	753	291	4367	5473	3747
Solyc02g064830.2.1	-3.95636	5.491764	7.53E-06	9.56E-05	686	134	38	2305	4735	2628
Solyc09g014590.2.1	-3.95153	0.407038	2.37E-05	0.000259	8	4	16	150	78	43
Solyc04g074540.2.1	-3.95149	0.237899	3.55E-05	0.000372	0	19	12	83	111	55
Solyc05g050350.1.1	-3.95124	6.996689	1.39E-09	4.55E-08	1333	1344	263	11979	9601	5253
Solyc05g005150.1.1	-3.9326	7.819165	4.87E-15	4.21E-13	1152	3582	1250	14563	21785	11876
Solyc04g077180.1.1	-3.93213	3.74936	2.82E-13	1.89E-11	43	229	94	621	1202	961
Solyc03g080190.2.1	-3.92899	8.533189	5.48E-13	3.49E-11	3398	3934	1439	23515	35827	19724
Solyc02g079150.1.1	-3.92213	0.543021	2.78E-07	5.32E-06	9	19	8	95	153	65
Solyc01g008690.2.1	-3.91719	3.791879	1.11E-07	2.34E-06	195	51	34	1143	1187	606
Solyc12g010410.1.1	-3.91693	4.050502	2.49E-16	2.57E-14	93	257	87	834	1792	946
Solyc09g011350.1.1	-3.91613	-1.25334	0.001126	0.007018	4	0	4	45	26	12
Solyc07g018200.1.1	-3.91381	5.795508	3.19E-10	1.22E-08	636	393	197	5656	3676	2194
Solyc08g021870.1.1	-3.90025	2.21147	3.91E-10	1.45E-08	17	74	34	338	323	280
Solyc04g073950.1.1	-3.89984	6.275788	4.14E-14	3.14E-12	729	607	449	6063	6287	3872
Solyc01g109120.2.1	-3.89082	6.477488	4.43E-11	2.02E-09	354	1991	302	5539	8667	4786
Solyc05g041910.2.1	-3.8848	9.489903	3.69E-14	2.82E-12	4936	10626	3406	47825	71864	34940
Solyc07g062490.1.1	-3.87793	9.61753	1.56E-08	4.14E-07	9699	3631	3542	78943	42294	37338
Solyc01g081340.2.1	-3.87757	2.344916	1.70E-09	5.48E-08	33	90	16	296	422	328
Solyc05g008310.2.1	-3.87722	6.396879	4.72E-15	4.09E-13	353	1346	622	4494	9590	4317
Solyc11g008600.1.1	-3.87708	3.292616	2.45E-13	1.66E-11	46	159	63	469	976	624
Solyc05g021080.1.1	-3.87596	2.200268	1.08E-07	2.29E-06	31	39	41	560	332	86
Solyc10g005300.2.1	-3.87437	0.127159	5.93E-05	0.00058	9	17	2	130	66	28
Solyc03g098100.2.1	-3.87076	8.726665	3.92E-08	9.32E-07	5727	2197	1188	23069	52072	19074
Solyc03g113130.2.1	-3.86845	2.016196	1.04E-08	2.87E-07	9	67	37	203	410	244
Solyc04g082960.1.1	-3.86102	6.266389	1.04E-14	8.68E-13	576	1078	370	3940	6950	5116
Solyc02g077520.2.1	-3.86025	2.240409	5.06E-10	1.83E-08	23	63	39	218	367	366
Solyc02g087450.2.1	-3.85986	1.294246	8.70E-08	1.89E-06	8	23	31	178	215	121
Solyc11g068780.1.1	-3.85872	2.338993	1.59E-07	3.22E-06	65	23	22	436	334	260
Solyc10g080010.1.1	-3.85725	7.457132	3.66E-13	2.40E-11	1514	2318	700	11696	16473	9125
Solyc02g068680.1.1	-3.85145	6.327868	1.15E-08	3.15E-07	786	1206	117	4017	9369	4186
Solyc06g072650.1.1	-3.8505	6.151981	1.03E-15	9.69E-14	359	1080	513	5946	5085	3615
Solyc07g042230.1.1	-3.84605	1.701016	3.98E-06	5.48E-05	20	71	4	338	242	103
Solyc02g087100.1.1	-3.8457	0.749929	2.33E-06	3.43E-05	12	24	8	189	98	56
Solyc08g078970.1.1	-3.84321	2.440135	2.08E-09	6.59E-08	54	60	21	488	422	225
Solyc02g072070.2.1	-3.8427	4.32305	8.15E-08	1.78E-06	260	190	23	1149	2074	1069
Solyc05g012180.2.1	-3.84092	5.399818	2.69E-19	3.99E-17	247	621	281	2790	3994	2181
Solyc04g005240.1.1	-3.84089	0.015483	4.85E-05	0.000485	11	13	1	46	109	58
Solyc03g007080.2.1	-3.83752	2.278419	2.83E-08	6.96E-07	53	45	19	459	425	154
Solyc06g065430.2.1	-3.83706	-1.94721	0.001592	0.009388	0	3	3	19	18	12

Solyc05g053150.1.1	-3.83539	5.546277	9.50E-07	1.58E-05	699	178	114	1416	6749	2426
Solyc02g068540.1.1	-3.83435	4.306594	5.82E-16	5.65E-14	155	175	160	1739	1464	904
Solyc02g067790.2.1	-3.83429	6.916219	1.04E-08	2.89E-07	1475	987	371	3766	16693	6624
Solyc09g011740.2.1	-3.82811	8.770147	1.90E-12	1.11E-10	4293	4540	2050	35521	37994	19021
NCRNA_5_231	-3.82443	-0.06383	0.000303	0.002317	13	8	1	24	106	69
Solyc08g078550.1.1	-3.82396	2.901362	4.25E-10	1.56E-08	30	109	68	223	971	450
Solyc03g119810.1.1	-3.82352	5.260564	8.64E-15	7.24E-13	287	609	159	3275	3070	1693
Solyc02g079540.1.1	-3.82322	4.426455	1.35E-14	1.10E-12	174	318	88	1779	1861	915
Solyc10g076780.1.1	-3.81838	5.881759	1.77E-15	1.63E-13	273	1021	404	4524	5028	2845
Solyc06g051970.2.1	-3.81602	3.106157	1.66E-09	5.36E-08	107	49	41	571	805	444
Solyc05g007110.2.1	-3.80874	2.802045	3.63E-05	0.000379	114	33	4	546	562	343
Solyc07g042550.2.1	-3.80719	9.811123	2.46E-14	1.97E-12	7325	10974	5336	59162	91676	42538
Solyc06g084370.1.1	-3.80523	3.947696	5.11E-16	5.03E-14	104	220	98	778	1779	803
Solyc08g074680.2.1	-3.80479	9.806091	1.11E-05	0.000133	13524	2012	3315	113342	40362	27578
Solyc08g007460.2.1	-3.80239	9.109928	4.42E-11	2.02E-09	5911	6251	1898	37184	54291	26706
Solyc10g086180.1.1	-3.802	9.814525	2.96E-11	1.39E-09	2844	13143	9632	84677	71561	33700
Solyc06g009290.2.1	-3.7998	9.657347	4.54E-08	1.06E-06	11446	4521	2264	50450	73344	45654
Solyc03g043700.2.1	-3.79377	4.33937	1.20E-12	7.28E-11	191	263	82	1937	1574	746
Solyc06g053270.1.1	-3.79067	0.284061	1.65E-05	0.000189	2	21	12	41	132	82
Solyc04g077810.1.1	-3.78979	4.420542	4.29E-10	1.57E-08	227	274	60	1021	1295	1841
Solyc05g056500.1.1	-3.77684	5.975221	1.88E-16	1.97E-14	515	771	399	4010	5576	3558
Solyc12g049030.1.1	-3.75727	7.089496	1.08E-07	2.29E-06	958	3107	184	7080	12433	8781
Solyc10g055820.1.1	-3.75312	5.091862	1.25E-14	1.03E-12	139	514	340	1616	2583	2631
Solyc09g007730.2.1	-3.75192	4.798842	2.51E-19	3.74E-17	178	384	221	1789	2382	1605
Solyc07g056470.2.1	-3.7501	6.275143	6.59E-06	8.52E-05	1289	261	151	7981	4876	3036
Solyc02g088510.2.1	-3.74843	4.170274	1.40E-17	1.66E-15	107	260	146	1319	1682	823
Solyc06g053440.2.1	-3.74558	-1.06749	0.000587	0.004036	0	7	6	26	60	16
Solyc05g052880.2.1	-3.74164	0.64404	8.95E-05	0.000821	21	6	9	194	66	50
Solyc12g088940.1.1	-3.7375	6.172283	4.42E-14	3.34E-12	291	1301	606	4053	6787	4273
Solyc12g057150.1.1	-3.73721	4.262624	8.05E-16	7.65E-14	179	221	116	1088	1896	1075
Solyc07g006800.1.1	-3.73628	1.245187	0.000176	0.001455	33	13	10	250	27	157
Solyc05g051210.2.1	-3.73434	1.640166	4.83E-07	8.70E-06	36	21	20	109	279	244
Solyc04g074480.2.1	-3.73248	11.37724	3.43E-14	2.64E-12	14750	35622	25546	205503	224532	126905
Solyc12g015860.1.1	-3.72961	8.3198	3.04E-13	2.03E-11	1587	4584	3117	28438	21134	15554
Solyc02g062390.2.1	-3.72899	6.213928	1.57E-06	2.45E-05	1117	590	100	3910	10385	2639
Solyc02g092870.1.1	-3.7245	3.265338	2.34E-08	5.92E-07	137	56	37	727	709	527
Solyc02g085130.2.1	-3.72377	3.609786	2.16E-14	1.74E-12	109	141	82	653	1256	684
Solyc03g006250.2.1	-3.72239	7.735536	2.73E-13	1.84E-11	935	3317	2091	16522	20352	8736
Solyc09g010780.2.1	-3.71741	5.729574	1.62E-16	1.73E-14	342	786	414	2794	6118	2637
Solyc11g006540.1.1	-3.71732	5.346928	1.62E-13	1.13E-11	462	325	249	2215	3907	2417
Solyc07g056000.2.1	-3.71714	6.062593	2.80E-09	8.64E-08	481	1487	143	5048	6498	2777
Solyc05g052670.1.1	-3.71577	9.267127	1.03E-10	4.34E-09	6270	5921	4030	65454	39138	22218
Solyc02g070920.2.1	-3.71147	1.760454	2.42E-08	6.11E-07	14	46	38	136	363	216
Solyc01g079980.2.1	-3.70926	0.603551	0.000651	0.004418	19	15	5	244	30	23
Solyc11g044840.1.1	-3.70898	5.782116	6.84E-08	1.53E-06	108	1314	424	1571	8599	2508
Solyc07g048060.1.1	-3.70866	5.251194	1.65E-07	3.32E-06	533	398	53	1936	4784	1718
Solyc04g057830.1.1	-3.70224	3.616263	1.53E-12	9.09E-11	103	204	54	974	1105	517
Solyc05g016060.2.1	-3.69905	8.268029	1.87E-08	4.84E-07	4297	2728	812	23232	28073	13911
Solyc02g071560.2.1	-3.69808	4.622621	0.000123	0.001077	5	678	190	483	2703	1962
Solyc08g005790.2.1	-3.69705	1.203457	2.30E-06	3.40E-05	5	56	15	199	188	93
Solyc01g102390.2.1	-3.69686	6.80256	3.86E-10	1.44E-08	1412	759	623	4950	12212	6669
Solyc01g097450.2.1	-3.69652	4.44477	1.14E-12	6.92E-11	60	426	209	1394	2069	1132
Solyc03g115060.2.1	-3.69442	7.883038	2.54E-09	7.88E-08	2919	2823	633	14844	21388	13106
Solyc02g079750.2.1	-3.6876	8.971787	1.02E-10	4.30E-09	5994	4581	2575	40624	34703	26718
Solyc03g044210.2.1	-3.68609	-0.17802	0.000471	0.003355	13	1	6	38	72	65
Solyc10g055420.1.1	-3.68443	1.480372	4.58E-05	0.000463	47	8	9	239	168	149
Solyc01g068630.2.1	-3.6843	3.514628	5.92E-08	1.35E-06	142	102	54	1428	596	307
Solyc10g076480.1.1	-3.68301	7.484669	1.19E-14	9.82E-13	1079	2953	1404	12164	16657	8945
Solyc03g083960.2.1	-3.68213	6.016945	4.55E-15	3.98E-13	534	953	403	4454	6774	2751
Solyc05g052650.2.1	-3.67396	2.689759	7.31E-08	1.62E-06	24	141	46	760	338	209
Solyc05g055080.1.1	-3.66534	2.634875	1.11E-06	1.81E-05	15	190	20	310	640	362
Solyc09g098010.2.1	-3.66463	0.117411	7.31E-05	0.000694	13	15	2	104	80	38
Solyc03g006610.2.1	-3.66289	5.340451	6.13E-17	6.85E-15	363	564	250	2619	3926	2031
Solyc03g111170.2.1	-3.65841	8.713964	2.67E-13	1.80E-11	4133	4551	2991	29987	38161	20212
Solyc04g081510.2.1	-3.65638	7.528654	1.41E-14	1.15E-12	1509	2578	1336	12339	17109	9383
Solyc12g100170.1.1	-3.65293	1.5424	9.15E-08	1.97E-06	19	59	14	137	296	177
Solyc07g056500.2.1	-3.6519	6.422742	1.88E-13	1.29E-11	880	1103	466	5317	7999	4664
Solyc09g014470.1.1	-3.65002	-0.57866	0.000429	0.003107	0	10	10	51	48	34
Solyc09g007930.2.1	-3.64956	2.262518	8.14E-05	0.000759	76	30	13	117	968	101
Solyc01g107400.2.1	-3.6474	3.944787	0.001163	0.00722	312	5	17	893	1015	1128
Solyc06g062920.2.1	-3.64564	5.400059	2.34E-05	0.000256	695	369	20	2902	3940	2058
Solyc08g006730.1.1	-3.64535	3.413862	4.55E-13	2.94E-11	63	167	96	912	910	420
Solyc03g119660.1.1	-3.64385	2.831498	3.01E-11	1.41E-09	67	85	51	366	648	454
Solyc02g079460.1.1	-3.64358	1.881757	1.45E-06	2.29E-05	53	36	9	290	293	176
Solyc02g093250.2.1	-3.63791	5.806885	1.74E-11	8.65E-10	454	1092	228	5051	3788	2577
Solyc02g083370.1.1	-3.63657	1.710499	2.88E-08	7.07E-07	31	47	18	153	321	206
Solyc09g007860.2.1	-3.63398	5.021973	1.03E-12	6.29E-11	377	423	129	1982	3099	1745
Solyc08g075320.2.1	-3.63108	4.041561	3.17E-11	1.48E-09	126	306	87	759	2497	546
Solyc01g056860.1.1	-3.63021	2.318968	1.10E-07	2.34E-06	47	65	33	582	334	121
Solyc11g007950.1.1	-3.62511	0.920154	0.000248	0.001955	0	50	17	177	152	65
Solyc03g005980.2.1	-3.6231	5.307821	1.83E-13	1.26E-11	283	807	196	2978	4134	1462
Solyc08g081540.2.1	-3.61787	3.978928	9.19E-13	5.66E-11	130	295	74	1304	1300	683
Solyc09g090980.2.1	-3.61411	9.789611	2.00E-09	6.35E-08	5475	21576	3354	76045	55715	46117
Solyc03g098660.2.1	-3.61363	5.733804	1.05E-10	4.37E-09	444	1089	191	2371	4521	3886

Solyc07g065090.1.1	-3.60746	7.427848	2.32E-12	1.34E-10	1770	1898	1263	14989	12799	7669
Solyc07g005110.2.1	-3.60665	2.879779	4.72E-08	1.10E-06	57	173	17	570	652	322
Solyc10g054010.1.1	-3.60435	4.381945	6.39E-15	5.45E-13	102	311	240	1038	2002	1297
Solyc11g066150.1.1	-3.59652	5.348153	2.83E-16	2.86E-14	386	512	307	2435	4572	1819
Solyc05g052660.1.1	-3.594	2.44338	2.54E-08	6.36E-07	23	115	45	565	321	213
Solyc10g084240.1.1	-3.59097	3.819461	3.46E-13	2.27E-11	133	132	138	1126	1340	539
Solyc12g096960.1.1	-3.58931	5.473707	5.97E-07	1.05E-05	551	739	64	4824	2603	1597
Solyc11g006910.1.1	-3.58704	4.574707	6.23E-14	4.57E-12	224	257	213	1132	1758	1830
Solyc01g105620.2.1	-3.5857	4.211109	2.47E-15	2.25E-13	169	290	114	1229	1617	992
Solyc03g083680.1.1	-3.5845	5.142951	2.20E-12	1.28E-10	358	614	136	2760	2937	1642
Solyc11g015900.1.1	-3.58313	5.483283	4.07E-10	1.50E-08	446	875	120	2264	4576	2585
Solyc11g005470.1.1	-3.58192	2.557947	1.29E-10	5.29E-09	37	111	44	409	510	301
Solyc03g113070.2.1	-3.58127	4.433705	1.77E-11	8.78E-10	209	317	134	2377	1346	702
Solyc04g079660.2.1	-3.5722	5.527167	9.04E-13	5.57E-11	200	895	458	1838	4873	2975
Solyc02g067610.1.1	-3.57123	2.185927	2.10E-07	4.15E-06	28	88	34	542	224	146
Solyc01g105920.2.1	-3.56958	4.148349	8.89E-07	1.49E-05	311	98	45	907	2182	799
Solyc02g065590.2.1	-3.56837	3.376584	2.51E-11	1.21E-09	95	151	74	1012	757	377
Solyc08g075970.2.1	-3.55924	6.950746	2.48E-14	1.97E-12	739	1923	1315	8482	11878	5769
Solyc11g069640.1.1	-3.5547	2.355763	2.95E-07	5.61E-06	48	19	72	292	490	284
Solyc03g082450.2.1	-3.55412	2.582616	1.10E-08	3.02E-07	21	127	59	523	362	309
Solyc05g025820.2.1	-3.55207	6.432016	3.27E-12	1.84E-10	901	1317	468	7199	6456	4040
Solyc08g005090.1.1	-3.54749	3.30255	1.03E-09	3.48E-08	29	165	135	529	794	666
Solyc05g006390.1.1	-3.54723	6.468143	1.60E-11	8.07E-10	476	2176	515	5956	7174	4980
Solyc02g064570.1.1	-3.54427	4.088106	5.13E-06	6.86E-05	301	118	32	1837	929	649
Solyc10g074800.1.1	-3.54256	0.506287	4.65E-05	0.000469	17	21	5	156	73	52
Solyc10g084170.1.1	-3.5387	4.622641	2.34E-09	7.30E-08	287	418	75	2448	1503	1023
Solyc09g011990.1.1	-3.53849	3.977089	5.29E-14	3.94E-12	143	214	132	1340	1011	807
Solyc11g005700.1.1	-3.53747	5.191187	2.00E-15	1.83E-13	374	519	241	2078	3795	1876
Solyc06g008100.2.1	-3.53123	4.050466	1.07E-07	2.28E-06	266	151	46	1557	1124	693
Solyc01g107770.1.1	-3.53092	2.099796	2.13E-07	4.20E-06	32	100	17	168	403	304
Solyc01g007050.1.1	-3.51866	3.434632	9.10E-08	1.97E-06	144	173	21	670	1070	537
Solyc01g106410.1.1	-3.51737	2.79523	2.14E-10	8.44E-09	34	117	81	347	690	410
Solyc07g006100.2.1	-3.51643	2.511161	7.99E-09	2.27E-07	61	52	55	512	486	197
Solyc01g089990.1.1	-3.51079	0.553641	7.48E-06	9.52E-05	15	19	11	131	71	81
Solyc06g074530.1.1	-3.50409	7.622647	2.97E-12	1.68E-10	1377	4234	1300	14446	17298	9279
Solyc12g007050.1.1	-3.50336	6.731319	2.88E-11	1.36E-09	1102	1927	483	7698	8659	5459
Solyc02g032170.2.1	-3.50239	-0.60534	0.001269	0.007759	0	18	6	26	45	54
Solyc06g062340.2.1	-3.50035	3.794643	5.70E-13	3.62E-11	98	280	101	962	893	884
Solyc01g107760.2.1	-3.4998	1.331858	5.49E-05	0.000541	26	24	25	25	333	180
Solyc12g006050.1.1	-3.49976	5.964549	2.54E-08	6.36E-07	975	660	184	4012	6541	2782
Solyc05g014300.1.1	-3.4996	1.548053	0.000335	0.002528	55	14	8	55	195	301
Solyc04g005160.1.1	-3.49207	10.08407	6.97E-13	4.35E-11	8338	19744	8752	82614	91434	50735
Solyc11g067190.1.1	-3.48979	6.128325	1.15E-09	3.82E-08	598	1040	639	2725	12019	1838
Solyc08g068690.1.1	-3.48716	9.308695	1.31E-09	4.30E-08	2574	17521	4207	44181	58623	29930
Solyc02g064560.1.1	-3.48546	0.298639	0.000796	0.005241	18	19	1	141	40	53
Solyc02g071970.1.1	-3.48225	5.164712	7.52E-10	2.63E-08	104	1008	279	1905	4002	1776
Solyc04g074990.2.1	-3.48142	3.12322	9.36E-12	4.90E-10	64	122	98	474	737	556
Solyc08g068720.1.1	-3.47881	2.011766	5.11E-05	0.000509	73	21	15	468	199	135
Solyc03g034240.2.1	-3.47444	1.806147	0.000222	0.001776	69	9	12	101	497	176
Solyc07g007590.1.1	-3.47388	6.61589	6.86E-13	4.29E-11	870	1752	699	7145	10159	3729
Solyc10g084960.1.1	-3.4737	4.783567	9.70E-14	6.96E-12	158	582	244	1355	2136	1991
Solyc08g082900.2.1	-3.4732	5.421194	2.46E-12	1.41E-10	439	824	195	3263	3781	1892
Solyc05g007930.2.1	-3.47083	5.267201	3.22E-15	2.88E-13	220	599	483	2282	4230	1748
Solyc02g092880.2.1	-3.47054	2.929924	1.27E-07	2.64E-06	122	70	30	508	561	457
Solyc02g093520.2.1	-3.46943	7.705686	4.67E-13	3.01E-11	2088	3076	1630	12390	19534	11434
Solyc09g010980.1.1	-3.46796	3.335213	8.92E-10	3.07E-08	88	228	43	534	1170	472
Solyc09g056430.2.1	-3.46291	4.02119	2.70E-08	6.71E-07	197	220	98	546	755	1669
Solyc10g078550.1.1	-3.45165	10.29306	3.82E-07	7.07E-06	20347	8361	6999	133686	70272	47565
Solyc05g005050.2.1	-3.44978	2.827975	2.09E-09	6.61E-08	87	100	41	578	552	322
Solyc02g070580.1.1	-3.44058	6.324341	8.67E-10	3.00E-08	1201	771	436	5339	7581	3843
Solyc03g082520.1.1	-3.43836	4.977008	1.66E-05	0.000191	613	248	33	1970	2522	1877
Solyc04g012050.2.1	-3.43641	1.092528	9.77E-06	0.00012	11	61	10	200	164	74
Solyc08g080540.2.1	-3.4353	4.286494	2.76E-08	6.82E-07	265	341	44	1481	1767	831
Solyc01g111350.2.1	-3.43504	6.303346	4.93E-10	1.79E-08	308	959	1395	4991	8173	3599
Solyc12g062510.1.1	-3.43427	5.194119	7.04E-16	6.76E-14	254	713	337	2251	3881	1664
Solyc03g114000.2.1	-3.42601	5.257361	2.13E-16	2.22E-14	267	644	421	2132	3676	2134
Solyc11g007500.1.1	-3.42183	6.465857	2.01E-05	0.000225	1646	869	102	3914	11049	4279
Solyc02g090110.2.1	-3.42167	7.859407	5.20E-11	2.33E-09	2786	3635	1315	16226	20837	11146
Solyc02g087330.2.1	-3.4124	7.520996	6.53E-13	4.10E-11	1902	2686	1567	11773	16012	9933
Solyc03g112680.1.1	-3.41125	0.659147	1.40E-05	0.000163	5	25	25	124	142	61
Solyc07g039290.1.1	-3.41097	3.491033	1.25E-08	3.41E-07	129	78	136	343	1250	756
Solyc08g016290.1.1	-3.40831	1.443755	6.60E-06	8.54E-05	25	54	17	74	440	107
Solyc04g011540.2.1	-3.4019	5.888327	9.85E-14	7.05E-12	603	979	458	4467	4558	3000
Solyc03g098090.1.1	-3.40077	2.560017	7.39E-06	9.41E-05	35	95	77	103	1149	170
Solyc08g082460.2.1	-3.39699	1.171108	3.50E-06	4.91E-05	6	48	30	136	204	121
Solyc12g055710.1.1	-3.3963	4.716094	4.98E-14	3.73E-12	192	613	184	1732	2269	1390
Solyc10g045420.1.1	-3.39194	6.831577	4.11E-10	1.51E-08	1306	1746	809	11832	7162	4047
Solyc05g051540.1.1	-3.39154	2.427612	2.28E-05	0.00025	64	116	13	670	322	107
Solyc01g095100.2.1	-3.3909	5.043988	3.03E-14	2.36E-12	217	772	263	1790	3386	1745
Solyc01g086730.2.1	-3.38742	6.722048	3.48E-12	1.95E-10	1053	2050	679	7597	8979	5137
Solyc01g096930.2.1	-3.38421	7.994285	3.34E-13	2.21E-11	2310	4300	2362	17605	21437	13155
Solyc11g006730.1.1	-3.38284	2.009327	7.57E-07	1.29E-05	57	64	14	250	351	220
Solyc03g113080.2.1	-3.37531	4.423443	1.66E-10	6.68E-09	292	241	159	2098	1527	760
Solyc12g006850.1.1	-3.3753	6.429719	1.69E-13	1.18E-11	783	1555	746	6002	8531	3655

Solyc03g117990.1.1	-3.37463	1.138763	5.75E-06	7.59E-05	8	39	33	208	147	87
Solyc06g062330.1.1	-3.37143	6.253433	2.33E-12	1.35E-10	770	1471	510	5349	5470	4404
Solyc03g025670.2.1	-3.37135	8.568004	5.84E-11	2.58E-09	2196	8702	3686	17162	42386	20836
Solyc07g064870.2.1	-3.3694	3.801461	9.16E-10	3.14E-08	203	153	91	1228	875	670
Solyc07g045100.1.1	-3.36802	7.249528	3.49E-09	1.06E-07	1808	3062	610	12007	14100	5848
Solyc09g007790.1.1	-3.36734	5.812634	1.80E-07	3.61E-06	964	369	345	5739	3583	2035
Solyc11g010430.1.1	-3.36652	5.345538	2.84E-16	2.87E-14	314	753	414	2451	3551	2302
Solyc04g075000.1.1	-3.36367	5.291743	3.87E-15	3.43E-13	399	557	390	2994	2731	2100
Solyc05g013710.2.1	-3.35902	5.065161	2.34E-08	5.92E-07	575	349	128	2859	2702	1335
Solyc05g025680.1.1	-3.35556	2.429175	9.60E-06	0.000118	76	41	51	631	143	242
Solyc02g092860.2.1	-3.35231	6.942897	4.74E-07	8.54E-06	2227	1077	475	6285	8387	9238
Solyc03g034360.2.1	-3.35185	2.653025	2.17E-05	0.000239	19	244	19	242	706	374
Solyc08g067610.2.1	-3.3447	7.09728	2.92E-07	5.55E-06	1639	3234	293	9987	10047	7424
Solyc06g071070.1.1	-3.33273	7.948429	8.37E-09	2.36E-07	3937	2555	1463	19703	18433	11813
Solyc01g099910.2.1	-3.33137	6.670158	2.66E-05	0.000287	2154	653	203	9177	6687	4537
Solyc05g051710.2.1	-3.3305	3.77464	6.77E-13	4.24E-11	148	245	102	881	1275	676
Solyc06g076090.2.1	-3.3305	1.037608	1.91E-06	2.92E-05	23	36	14	159	170	89
Solyc03g034250.2.1	-3.32865	5.834809	9.96E-14	7.11E-12	569	896	570	3569	6197	2405
Solyc04g054690.2.1	-3.32433	5.134071	1.45E-08	3.87E-07	499	633	117	3225	2910	1161
Solyc04g009900.2.1	-3.32354	5.95721	2.09E-11	1.03E-09	341	1173	845	2538	6460	3878
Solyc01g066940.2.1	-3.32341	6.185332	3.18E-13	2.10E-11	652	1411	654	5739	5722	3357
Solyc05g025690.1.1	-3.31846	0.853196	5.70E-05	0.00056	20	12	26	201	91	62
Solyc01g058250.1.1	-3.31632	5.378265	3.22E-10	1.23E-08	171	712	689	1578	4435	2627
Solyc06g065530.2.1	-3.316	3.115906	6.61E-07	1.15E-05	141	130	25	620	541	530
Solyc12g088390.1.1	-3.31423	6.896575	2.73E-12	1.55E-10	1176	2388	910	8192	9170	6582
Solyc06g007930.2.1	-3.31283	7.617806	1.02E-12	6.24E-11	1470	3702	2237	11806	17846	10656
Solyc01g098960.1.1	-3.3109	-1.11571	0.001021	0.006454	2	7	7	41	20	24
Solyc08g068700.1.1	-3.31081	7.950516	3.67E-06	5.11E-05	4408	2725	893	28592	11286	8711
Solyc07g006770.2.1	-3.30563	4.766871	1.07E-13	7.64E-12	231	613	215	1318	2840	1520
Solyc08g079420.2.1	-3.30221	8.731884	2.48E-11	1.20E-09	4943	7576	3033	25727	37919	23294
Solyc11g068380.1.1	-3.30139	3.530718	2.87E-09	8.82E-08	157	155	87	1130	880	366
Solyc05g052890.2.1	-3.30108	-0.27693	0.000811	0.00532	15	8	3	85	53	25
Solyc05g044490.2.1	-3.30072	4.449985	5.19E-10	1.87E-08	337	188	183	1002	2149	1339
Solyc05g056410.1.1	-3.30009	0.687747	2.62E-05	0.000284	20	14	19	150	84	77
Solyc08g005950.2.1	-3.29893	1.838799	2.54E-07	4.93E-06	39	32	49	259	257	194
Solyc06g053150.1.1	-3.29623	5.445518	6.23E-14	4.57E-12	514	737	332	2635	4138	2238
Solyc02g093270.2.1	-3.2951	9.206563	2.46E-12	1.41E-10	5899	10052	5807	34500	52499	33459
Solyc03g078490.2.1	-3.29319	9.945507	1.23E-08	3.36E-07	11362	18559	6716	27305	131106	55331
Solyc01g107090.2.1	-3.29283	6.966296	2.13E-11	1.04E-09	1168	2885	851	6741	11698	7194
Solyc10g009140.2.1	-3.29103	0.517394	8.92E-05	0.00082	10	13	26	42	129	111
Solyc02g031990.1.1	-3.28609	5.622338	7.33E-07	1.26E-05	886	673	110	3899	3842	2259
Solyc10g081980.1.1	-3.28516	7.982563	9.61E-10	3.27E-08	1595	7441	1692	15369	24464	12659
Solyc08g068660.1.1	-3.27831	-0.42083	0.000168	0.001406	6	16	6	67	49	30
Solyc02g088220.2.1	-3.27539	5.832552	2.17E-10	8.54E-09	832	845	337	4285	4556	2727
Solyc05g051530.2.1	-3.27436	5.649222	3.32E-08	8.04E-07	615	1072	234	5588	3232	1375
Solyc11g045460.1.1	-3.27072	3.109973	2.92E-09	8.97E-08	91	194	52	517	591	576
Solyc01g107100.2.1	-3.26567	6.427695	1.47E-12	8.79E-11	792	1775	784	5671	6069	5237
Solyc02g090400.2.1	-3.26244	2.684521	1.08E-06	1.76E-05	107	91	26	322	836	248
Solyc05g005060.2.1	-3.2592	3.267243	1.57E-08	4.15E-07	149	147	47	611	762	553
Solyc03g121420.2.1	-3.25872	3.610939	1.23E-09	4.08E-08	87	249	145	671	1753	332
Solyc12g014500.1.1	-3.2503	-0.46098	0.000451	0.003243	1	17	11	47	56	39
Solyc03g025870.2.1	-3.24867	-0.11802	0.000679	0.004576	16	11	4	96	41	38
Solyc05g056400.2.1	-3.24707	2.22826	0.000687	0.004624	84	38	31	711	52	115
Solyc02g078370.1.1	-3.24352	0.113811	0.000243	0.001923	17	19	3	86	72	53
Solyc04g040180.2.1	-3.23875	10.29216	1.46E-09	4.75E-08	18761	16817	8954	92500	102237	59905
Solyc04g050790.2.1	-3.23809	2.332908	2.62E-07	5.07E-06	58	106	32	185	651	250
Solyc11g005480.1.1	-3.22908	6.917551	4.18E-11	1.93E-09	1178	2434	1152	11052	8705	4690
Solyc05g010470.2.1	-3.22594	6.354522	5.27E-11	2.36E-09	1164	1154	646	5571	7373	3869
Solyc07g043320.2.1	-3.22447	9.714616	9.36E-10	3.19E-08	11964	13740	5439	51754	83464	39701
Solyc12g040570.1.1	-3.22446	5.707006	4.23E-13	2.75E-11	464	1172	481	4092	4348	2240
Solyc03g117980.2.1	-3.22337	10.22715	1.61E-11	8.10E-10	9611	26381	12714	85827	98200	58919
Solyc04g079250.2.1	-3.22165	4.199666	8.53E-07	1.44E-05	81	648	120	434	2591	990
Solyc02g067800.2.1	-3.22104	1.479632	4.73E-05	0.000476	16	80	24	48	272	233
Solyc10g081320.1.1	-3.2161	-0.2707	0.000442	0.003185	2	20	12	70	41	45
Solyc11g008640.1.1	-3.21583	1.573826	8.46E-07	1.43E-05	17	67	38	163	202	209
Solyc01g107860.2.1	-3.21352	7.574879	2.41E-12	1.39E-10	1691	3369	2388	12071	17808	9373
Solyc02g069600.2.1	-3.2111	0.839463	1.20E-05	0.000143	14	49	12	129	177	66
Solyc06g036290.2.1	-3.20073	3.513673	1.15E-06	1.87E-05	156	144	115	1498	477	267
Solyc08g008150.1.1	-3.19921	3.479083	3.47E-09	1.05E-07	87	319	78	458	1054	739
Solyc01g098840.2.1	-3.19075	8.056798	7.73E-09	2.21E-07	3705	3990	2259	27026	14335	10841
Solyc07g062630.1.1	-3.18655	0.928726	3.67E-05	0.000382	10	64	12	146	183	66
Solyc12g005940.1.1	-3.18589	7.442794	4.82E-05	0.000483	4073	921	520	13660	16494	6207
Solyc03g026230.1.1	-3.1781	4.784423	6.55E-11	2.87E-09	324	625	179	1339	3488	1143
Solyc03g118450.1.1	-3.17796	4.118972	3.99E-10	1.48E-08	185	469	88	1042	1608	901
Solyc12g014010.1.1	-3.17488	7.120043	2.08E-05	0.000231	3105	1149	375	11781	8299	7073
Solyc09g083090.2.1	-3.17466	4.48157	6.09E-08	1.38E-06	430	238	112	1074	2278	1252
Solyc04g071090.2.1	-3.1741	1.287591	0.000689	0.004635	6	77	29	160	32	227
Solyc05g008820.2.1	-3.17176	5.173304	4.79E-14	3.61E-12	359	811	325	2023	3273	2025
Solyc01g096510.2.1	-3.1715	6.874367	3.07E-10	1.18E-08	827	3452	963	7029	10154	6505
Solyc09g011860.2.1	-3.16799	5.323295	8.84E-10	3.05E-08	460	1058	187	2305	4446	1725
Solyc03g119230.1.1	-3.16739	2.141883	2.83E-07	5.40E-06	48	113	25	305	376	212
Solyc01g104740.2.1	-3.16268	5.388804	9.67E-05	0.000878	998	228	122	1062	3460	3592
Solyc02g093040.2.1	-3.16227	2.249948	5.19E-05	0.000516	73	41	54	610	133	157
Solyc06g005480.2.1	-3.16161	5.716069	3.60E-10	1.35E-08	808	907	309	3177	4978	2636

Solyc09g011330.1.1	-3.15547	6.049518	9.61E-08	2.06E-06	1166	1027	289	5602	4108	3268
Solyc01g087020.2.1	-3.15289	4.811799	1.24E-14	1.03E-12	263	609	299	1770	2616	1371
Solyc11g073060.1.1	-3.15025	2.242173	0.000245	0.001936	80	95	11	159	156	508
Solyc03g097570.2.1	-3.14412	2.233457	0.001032	0.006505	115	14	19	156	828	113
Solyc02g091070.2.1	-3.14379	5.61756	8.12E-05	0.000758	1221	165	165	3475	3854	2499
Solyc06g069040.2.1	-3.14217	4.752475	1.16E-09	3.87E-08	179	844	218	2385	1502	1338
Solyc12g010940.1.1	-3.13864	1.673179	0.000133	0.001154	3	101	46	149	345	167
Solyc11g008200.1.1	-3.13804	3.717079	2.11E-08	5.39E-07	149	355	67	1224	949	476
Solyc02g067440.2.1	-3.13689	-0.0436	0.000233	0.001859	4	19	17	47	60	74
Solyc02g072400.1.1	-3.1367	1.941643	1.49E-05	0.000173	35	87	40	484	187	86
Solyc05g008640.1.1	-3.13624	3.859905	8.01E-11	3.43E-09	146	287	165	546	1820	734
Solyc01g057910.2.1	-3.13516	4.40346	2.12E-11	1.04E-09	280	351	202	1899	1524	826
Solyc03g082640.1.1	-3.13459	4.113086	1.47E-10	5.99E-09	101	495	189	843	1947	848
Solyc12g057130.1.1	-3.13457	0.304396	0.001512	0.009012	30	6	4	74	86	77
Solyc04g005670.1.1	-3.13334	8.533269	1.04E-10	4.35E-09	4496	7654	3097	23515	36656	16664
Solyc02g094520.2.1	-3.13187	4.056454	2.12E-10	8.35E-09	203	262	190	1647	1013	630
Solyc04g009440.2.1	-3.13084	8.914883	5.45E-08	1.25E-06	8629	6840	2621	27711	44344	26050
Solyc10g011910.2.1	-3.12436	4.78236	4.34E-09	1.29E-07	187	972	158	1488	2582	1525
Solyc02g080290.2.1	-3.12416	4.90748	4.29E-07	7.83E-06	635	267	147	2414	2179	1386
Solyc01g106390.2.1	-3.12311	6.888778	8.94E-10	3.07E-08	1120	3417	794	8382	10451	5351
Solyc11g012240.1.1	-3.12119	2.296543	9.56E-06	0.000117	16	109	90	514	258	185
Solyc03g112340.1.1	-3.12093	7.505955	1.12E-09	3.74E-08	1757	5142	1243	10412	16422	9969
Solyc01g105280.2.1	-3.1189	7.936178	4.26E-11	1.96E-09	2575	5035	2636	19488	20012	10207
Solyc11g068920.1.1	-3.11759	0.911771	0.00086	0.005591	2	78	15	52	236	107
Solyc10g086280.1.1	-3.11426	9.379525	1.97E-09	6.29E-08	9704	12672	4543	47175	56714	31145
Solyc08g079480.2.1	-3.10894	3.136533	1.83E-06	2.81E-05	173	104	46	457	550	658
Solyc02g071220.2.1	-3.1037	6.21285	2.22E-11	1.09E-09	881	1649	619	4039	7161	3957
Solyc05g054820.1.1	-3.10323	2.021667	0.000193	0.001581	42	142	7	441	260	111
Solyc04g071770.2.1	-3.1013	6.491935	1.11E-08	3.04E-07	1441	1439	654	8472	6052	3417
Solyc02g078380.2.1	-3.09643	7.227945	5.68E-10	2.04E-08	1725	3126	1478	10558	7886	9847
Solyc04g077630.2.1	-3.09633	3.963558	1.27E-08	3.44E-07	91	498	151	529	1650	1007
Solyc12g087790.1.1	-3.09382	2.561486	1.47E-07	3.00E-06	60	168	38	277	604	328
Solyc09g014990.2.1	-3.09052	8.351708	2.46E-08	6.19E-07	4282	8476	1559	21280	30404	15203
Solyc08g068790.1.1	-3.09032	2.616451	1.51E-07	3.07E-06	87	122	44	530	374	290
Solyc02g079510.2.1	-3.08924	2.236223	3.06E-07	5.78E-06	36	85	77	190	573	233
Solyc03g115860.2.1	-3.0885	7.18234	8.30E-10	2.88E-08	1954	2289	1580	13114	9418	6151
Solyc05g010720.2.1	-3.08649	3.959491	9.50E-10	3.23E-08	199	367	104	720	1221	1091
Solyc03g097100.1.1	-3.07389	-0.28465	0.000549	0.003816	10	21	4	28	89	48
Solyc09g090470.2.1	-3.07045	5.152942	7.84E-12	4.17E-10	459	808	268	2515	2598	1897
Solyc05g014980.2.1	-3.06536	7.65646	4.87E-11	2.20E-09	2015	4154	2550	9816	18953	11938
Solyc03g020000.2.1	-3.06512	2.958116	2.27E-05	0.000249	154	56	70	904	364	240
Solyc04g006930.2.1	-3.06436	2.103816	2.23E-07	4.38E-06	50	65	60	359	290	195
Solyc01g094980.1.1	-3.06392	-0.4691	0.000449	0.00323	3	19	10	49	66	29
Solyc10g074570.1.1	-3.06226	7.415133	6.38E-10	2.26E-08	2515	2950	1525	12343	15492	7117
Solyc03g119980.2.1	-3.06195	6.397887	1.23E-11	6.28E-10	674	1708	1298	5581	7752	3879
Solyc03g122340.2.1	-3.05649	8.17637	1.32E-10	5.38E-09	2334	6251	4218	14617	31385	14215
Solyc12g009220.1.1	-3.05549	8.809393	1.04E-10	4.37E-09	5008	10283	4457	28174	44476	19985
Solyc03g006770.2.1	-3.05433	3.401343	2.75E-06	3.97E-05	214	125	61	1081	569	404
Solyc02g071780.2.1	-3.05136	6.393776	1.01E-11	5.27E-10	939	1791	929	6124	7456	3561
Solyc01g106620.2.1	-3.0509	11.78187	1.90E-05	0.000214	82552	31144	13314	278742	221244	183697
Solyc01g079780.2.1	-3.04706	6.525733	8.63E-12	4.54E-10	1072	1618	1194	5737	7429	5119
Solyc06g005820.2.1	-3.04435	1.22511	0.00136	0.00824	23	2	60	160	156	134
Solyc04g074950.2.1	-3.04409	5.315449	4.80E-12	2.62E-10	515	905	323	2950	3166	1852
Solyc05g053790.1.1	-3.04364	1.854402	0.000627	0.004277	86	33	12	442	138	110
Solyc02g091650.2.1	-3.04209	6.078904	4.68E-09	1.39E-07	1151	1100	488	5408	5283	2871
Solyc04g074110.2.1	-3.03796	2.996779	2.47E-07	4.82E-06	138	86	84	685	494	373
Solyc02g087210.2.1	-3.03673	6.39954	2.69E-07	5.19E-06	1481	1752	325	5733	5944	4791
Solyc04g077310.2.1	-3.0316	5.954026	4.89E-12	2.66E-10	614	1522	682	3487	5827	3257
Solyc08g077900.2.1	-3.02589	6.773036	0.000305	0.002333	2281	2088	116	12193	5109	3812
Solyc09g098000.2.1	-3.02454	0.577313	0.000347	0.002601	30	17	10	137	68	74
Solyc10g055680.1.1	-3.02289	6.405488	9.09E-10	3.12E-08	1139	1886	705	3611	9749	4358
Solyc09g011390.2.1	-3.02167	3.783516	4.88E-09	1.44E-07	237	232	98	886	979	811
Solyc01g010430.2.1	-3.02114	6.774453	1.05E-10	4.37E-09	1290	2612	1027	8022	9435	4717
Solyc06g069720.2.1	-3.02014	0.681513	3.58E-05	0.000374	23	20	21	106	160	62
Solyc02g079720.1.1	-3.01179	1.253991	5.42E-05	0.000536	47	35	13	145	257	93
Solyc03g083480.2.1	-3.01034	6.920217	1.08E-05	0.00013	2570	2020	306	9248	8438	6056
Solyc03g121340.1.1	-3.00552	3.456339	2.78E-06	4.00E-05	58	444	77	374	861	869
Solyc11g007020.1.1	-3.00188	8.112927	2.54E-08	6.36E-07	1648	5667	5327	24612	15920	12940
Solyc09g083360.2.1	-3.00052	4.318537	8.97E-05	0.000822	400	248	138	2843	435	471
Solyc02g014220.2.1	-3.0005	0.482355	0.000485	0.003439	24	31	5	140	74	53
Solyc03g005570.2.1	-2.98919	6.441531	1.03E-09	3.47E-08	1069	2369	639	6123	6623	4414
Solyc05g009550.2.1	-2.98904	4.920763	9.87E-11	4.19E-09	242	916	325	1331	3548	1473
Solyc01g103650.2.1	-2.98815	8.519487	3.20E-09	9.74E-08	5498	7966	2789	24624	28893	19102
Solyc03g112700.1.1	-2.98549	6.843096	2.91E-11	1.38E-09	1234	2564	1436	8276	8955	5554
Solyc10g012370.2.1	-2.98538	10.13679	1.54E-07	3.12E-06	20109	15915	10044	106301	64063	47748
Solyc03g115870.2.1	-2.98408	5.599338	2.21E-08	5.63E-07	786	753	490	5137	2751	1601
Solyc12g045020.1.1	-2.98371	5.101678	1.99E-05	0.000223	282	1511	63	2143	3247	1600
Solyc09g011630.2.1	-2.98049	7.003789	0.000583	0.004015	3472	1096	155	11946	7635	5380
Solyc06g050790.2.1	-2.97143	4.352588	3.38E-12	1.90E-10	171	522	279	1389	1578	1060
Solyc02g081830.2.1	-2.96775	4.386305	7.94E-12	4.20E-10	303	371	235	1499	1654	998
Solyc10g074730.1.1	-2.96751	0.89473	0.000142	0.001219	5	43	40	98	169	102
Solyc01g009780.2.1	-2.96362	6.555335	5.10E-11	2.29E-09	1215	2057	1002	5480	8592	4856
Solyc06g072330.2.1	-2.95728	4.970415	3.86E-05	0.000399	792	216	159	3081	1325	1483
Solyc02g062380.1.1	-2.95625	2.625723	4.06E-05	0.000417	149	49	36	331	708	254

Solyc06g065560.1.1	-2.95588	7.940912	1.02E-09	3.44E-08	3432	5511	2261	18042	20064	11017
Solyc01g100010.2.1	-2.95509	6.715139	6.70E-09	1.93E-07	1722	2157	780	8003	8000	4807
Solyc07g020870.1.1	-2.94734	5.139069	2.23E-09	7.00E-08	207	680	754	2929	2339	1607
Solyc02g071090.2.1	-2.94271	3.200127	0.000117	0.001033	13	337	145	269	821	698
Solyc07g008550.2.1	-2.93881	3.157819	1.53E-06	2.40E-05	170	84	104	774	460	454
Solyc10g008960.1.1	-2.93806	1.735655	6.08E-06	7.97E-05	55	62	29	132	368	181
Solyc08g066020.1.1	-2.93451	6.74178	1.63E-08	4.29E-07	1844	2068	821	8730	7747	4632
Solyc02g083360.1.1	-2.93314	5.645022	2.49E-11	1.20E-09	724	1101	471	2978	4511	2548
Solyc02g079170.2.1	-2.93012	9.018588	5.24E-08	1.21E-06	9728	8523	4010	35954	45666	22907
Solyc01g073820.2.1	-2.92633	3.206485	5.64E-05	0.000554	184	199	28	1025	499	272
Solyc03g112060.2.1	-2.92608	6.792387	8.24E-10	2.86E-08	1778	1981	1140	6998	10192	5196
Solyc03g006620.2.1	-2.92474	6.402951	1.87E-10	7.42E-09	1047	2159	845	6013	7551	3552
Solyc02g086900.2.1	-2.91873	6.475388	2.12E-09	6.71E-08	840	2255	1235	4643	5181	6692
Solyc03g098730.1.1	-2.91851	9.112784	3.11E-06	4.42E-05	11555	10370	2183	41501	44022	24565
Solyc08g066420.1.1	-2.91499	4.123161	1.14E-10	4.73E-09	150	488	230	1371	1134	863
Solyc12g007100.1.1	-2.91438	5.998008	1.81E-10	7.18E-09	958	1270	678	4608	5622	2666
Solyc10g006450.2.1	-2.90987	2.390333	8.99E-08	1.94E-06	60	126	64	323	508	233
Solyc11g010730.1.1	-2.90736	5.115654	1.90E-11	9.41E-10	402	880	397	1429	3838	1858
Solyc04g051500.2.1	-2.90617	7.733859	1.03E-09	3.48E-08	1649	5702	3277	13420	19591	9937
Solyc01g067750.2.1	-2.90113	8.602538	2.11E-08	5.40E-07	7108	5941	3805	27388	33249	17234
Solyc10g080050.1.1	-2.89973	-0.96749	0.001562	0.009251	6	12	5	33	54	15
Solyc12g005850.1.1	-2.89945	6.334061	4.26E-10	1.56E-08	1228	1634	844	4084	7583	4481
Solyc12g057160.1.1	-2.89812	3.559077	1.49E-06	2.35E-05	123	431	69	353	1485	632
Solyc11g010710.1.1	-2.89268	2.74315	7.01E-08	1.56E-06	89	150	77	343	789	272
Solyc04g011690.2.1	-2.88984	5.909806	8.62E-07	1.45E-05	1387	688	397	4220	5184	2643
Solyc07g005100.2.1	-2.88825	7.452664	4.04E-10	1.50E-08	2403	3748	2083	9614	16452	9110
Solyc07g008930.1.1	-2.88733	1.549212	0.000106	0.000949	26	121	13	255	215	107
Solyc03g116900.2.1	-2.88535	8.28212	1.09E-07	2.31E-06	4905	7470	2423	10773	36445	17150
Solyc01g080890.2.1	-2.88469	5.650968	4.20E-11	1.94E-09	777	914	596	3516	4179	2303
Solyc07g045030.2.1	-2.88079	3.398821	1.03E-06	1.69E-05	55	448	94	788	878	440
Solyc09g011560.2.1	-2.88038	6.527211	5.89E-09	1.72E-07	1245	2703	675	5470	8261	4686
Solyc04g074470.1.1	-2.88012	5.886985	4.05E-09	1.22E-07	1039	1121	527	4438	4019	2990
Solyc01g096790.2.1	-2.87951	3.510521	3.20E-08	7.79E-07	187	212	120	405	1232	674
Solyc01g087850.2.1	-2.87866	9.013419	2.20E-09	6.92E-08	5169	11441	8411	39064	31515	27947
Solyc08g078050.1.1	-2.87843	6.124829	2.98E-10	1.15E-08	662	1914	944	3217	7536	3550
Solyc02g090150.1.1	-2.87373	1.024641	0.000186	0.001528	39	15	29	148	201	66
Solyc12g011160.1.1	-2.8722	8.523279	2.45E-10	9.61E-09	4305	8498	5051	21995	32781	18622
Solyc11g016930.1.1	-2.86985	3.454396	2.84E-08	7.00E-07	201	198	96	578	968	619
Solyc09g056390.1.1	-2.86807	6.169219	4.70E-09	1.39E-07	1103	1464	798	6577	4592	2836
Solyc04g014640.1.1	-2.86444	-0.22667	0.000495	0.003488	6	29	9	42	85	42
Solyc04g054710.2.1	-2.85982	9.625476	7.84E-10	2.73E-08	8293	18073	12387	56901	62306	36641
Solyc01g099860.2.1	-2.85905	5.895465	2.57E-11	1.23E-09	708	1349	839	3846	5717	2527
Solyc10g008700.1.1	-2.85759	0.805323	5.58E-05	0.000549	19	27	34	129	106	94
Solyc05g007950.2.1	-2.85603	3.43296	2.40E-06	3.53E-05	237	105	108	495	1342	443
Solyc04g045600.2.1	-2.85222	6.689461	5.24E-08	1.21E-06	1625	1843	1298	10348	5733	3796
Solyc01g102530.2.1	-2.85089	6.314401	4.83E-11	2.18E-09	949	1843	1111	4698	7000	4105
Solyc10g076600.1.1	-2.85072	7.434117	3.01E-10	1.16E-08	2219	3985	2235	10688	15527	8351
Solyc10g082020.1.1	-2.85032	0.238084	0.000331	0.002504	8	31	20	38	115	77
Solyc08g080130.2.1	-2.84849	9.251662	8.21E-09	2.33E-07	9289	15122	5413	42593	49849	28243
Solyc12g008670.1.1	-2.84597	0.755635	0.00023	0.001833	33	36	9	81	135	107
Solyc05g018180.2.1	-2.84566	6.497384	1.15E-10	4.77E-09	1187	2127	1116	5873	7976	4198
Solyc05g014320.2.1	-2.84336	4.85824	1.20E-12	7.25E-11	361	697	375	1712	2528	1505
Solyc08g080860.2.1	-2.83521	3.936561	3.01E-07	5.70E-06	298	231	156	1535	847	559
Solyc02g086590.2.1	-2.83394	6.704952	3.50E-10	1.32E-08	1262	2809	1231	5663	9386	5642
Solyc09g014600.1.1	-2.83266	1.686552	0.000204	0.001658	14	44	95	289	152	158
Solyc02g092580.2.1	-2.8313	10.21673	8.69E-06	0.000108	28086	14036	8632	107786	61399	55580
Solyc04g082500.2.1	-2.83128	5.267447	3.17E-11	1.48E-09	550	989	383	2597	2889	2001
Solyc02g071690.1.1	-2.82898	0.308396	0.000736	0.004905	20	25	13	34	73	114
Solyc05g052750.2.1	-2.82878	6.391582	1.48E-08	3.94E-07	1562	1684	693	5545	7212	3927
Solyc04g081960.1.1	-2.82466	4.362743	1.43E-11	7.28E-10	262	542	238	1309	1846	954
Solyc08g006330.2.1	-2.82214	7.10441	3.96E-08	9.37E-07	2671	2484	1199	8519	12750	6353
Solyc04g016420.2.1	-2.82118	3.142254	7.99E-07	1.35E-05	54	346	101	429	689	576
Solyc12g098780.1.1	-2.82041	6.892672	1.48E-09	4.79E-08	1443	2720	1744	10091	8048	4969
Solyc09g064510.2.1	-2.81762	6.95759	4.22E-10	1.55E-08	1676	3103	1463	7847	11520	5605
Solyc09g098510.2.1	-2.81382	11.47045	6.30E-09	1.82E-07	34665	58707	45904	224967	173298	142449
Solyc08g007980.2.1	-2.81291	2.210291	9.64E-07	1.59E-05	67	79	72	262	334	285
Solyc05g008990.2.1	-2.8125	4.310187	2.65E-11	1.26E-09	195	566	279	1144	1670	1077
Solyc07g056440.2.1	-2.80996	3.805787	1.67E-10	6.71E-09	146	344	222	735	1148	833
Solyc08g008110.2.1	-2.80863	5.411609	1.29E-10	5.31E-09	642	1043	439	3237	3552	1692
Solyc11g072630.1.1	-2.80607	4.763044	5.63E-06	7.45E-05	699	204	218	1341	2700	1415
Solyc07g045090.2.1	-2.80116	0.286785	0.000216	0.001732	18	33	10	64	103	68
Solyc07g043510.2.1	-2.8004	5.204338	3.31E-10	1.26E-08	667	638	421	2160	3519	1729
Solyc05g007940.2.1	-2.80006	4.360944	5.55E-06	7.35E-05	372	316	259	904	3465	335
Solyc03g025680.2.1	-2.79565	5.427695	3.22E-10	1.23E-08	545	1294	435	1958	4631	2174
Solyc08g076390.2.1	-2.79516	6.41184	3.72E-09	1.12E-07	1488	1658	931	5938	7579	3536
Solyc03g123680.1.1	-2.79266	6.638725	4.79E-09	1.42E-07	1286	3119	909	6015	8071	5359
Solyc06g007180.2.1	-2.79191	7.453388	0.001122	0.006997	4934	2105	445	22780	6916	3702
Solyc01g058720.2.1	-2.79142	6.415813	2.10E-05	0.000233	2280	998	389	4671	7950	4384
Solyc08g082790.2.1	-2.78619	-0.87745	0.001464	0.008766	6	12	8	29	60	19
Solyc08g008100.2.1	-2.78602	7.931154	1.44E-08	3.86E-07	3894	6047	2366	18507	19887	9968
Solyc08g078070.2.1	-2.78205	8.483671	1.57E-09	5.07E-08	3550	9778	5609	23374	31617	16255
Solyc09g007900.2.1	-2.78158	9.002301	1.36E-06	2.16E-05	6171	19128	3438	47719	34996	17651
RRNA_11_11	-2.78074	0.376686	0.000384	0.002827	13	29	23	35	114	97
Solyc04g051860.2.1	-2.77876	7.267827	2.57E-09	7.99E-08	1437	4134	2589	11290	12942	6378

Solyc02g088270.2.1	-2.77871	2.857534	0.000123	0.001079	106	127	116	84	1223	304
Solyc07g019460.2.1	-2.77752	9.082191	2.39E-09	7.45E-08	7101	14203	6806	37563	47898	22800
Solyc07g006500.2.1	-2.77473	7.024041	0.000152	0.001289	3540	1737	435	13016	7324	4629
Solyc11g042460.1.1	-2.77466	6.125996	5.10E-10	1.84E-08	1063	1559	871	3522	6743	3675
Solyc06g050630.2.1	-2.77415	4.227401	1.41E-07	2.90E-06	399	355	133	1449	1392	813
Solyc10g007900.2.1	-2.77317	5.485339	2.42E-05	0.000263	419	1278	708	443	6716	2451
Solyc08g081610.2.1	-2.77243	1.679	8.26E-06	0.000103	58	59	36	194	250	173
Solyc05g052680.1.1	-2.77207	6.690671	5.22E-09	1.53E-07	896	3458	1399	7379	8370	4595
Solyc02g093860.2.1	-2.77048	8.126022	4.59E-09	1.36E-07	3022	9081	3231	16470	25632	13532
Solyc09g057960.1.1	-2.77031	4.496903	6.37E-05	0.000616	646	226	87	1707	1486	1123
Solyc08g082510.1.1	-2.76992	-0.10874	0.00131	0.007969	10	32	8	66	34	63
Solyc05g014380.2.1	-2.76787	8.655384	1.76E-07	3.54E-06	8081	7582	3601	29774	26718	20571
Solyc02g069740.2.1	-2.76758	2.568642	2.28E-07	4.48E-06	79	131	89	273	624	300
Solyc06g062420.2.1	-2.76293	3.774663	0.000106	0.000951	186	488	85	1845	494	254
Solyc10g081430.1.1	-2.76169	1.496352	1.00E-05	0.000122	39	72	34	217	234	106
Solyc01g090210.2.1	-2.75938	4.129294	9.73E-10	3.30E-08	228	497	204	1384	1521	604
Solyc11g011120.1.1	-2.75918	7.125291	1.12E-09	3.75E-08	1659	3110	2383	6991	12502	7927
Solyc12g098760.1.1	-2.75771	7.284369	6.29E-09	1.82E-07	1989	3369	2564	13042	9773	7006
Solyc05g014390.2.1	-2.75505	7.460991	2.08E-07	4.11E-06	3653	3037	1661	11706	11597	10064
Solyc02g079180.1.1	-2.74986	1.123412	0.000401	0.002929	45	55	9	171	116	116
Solyc07g005150.1.1	-2.74554	0.886509	0.000169	0.001407	25	34	32	52	187	122
Solyc09g009130.2.1	-2.74315	5.853315	8.36E-08	1.83E-06	1130	1033	626	2288	7068	2668
Solyc03g120260.2.1	-2.73682	7.433388	0.000253	0.001991	4799	2076	786	19697	8265	4944
Solyc08g065480.2.1	-2.73591	7.759221	2.28E-09	7.13E-08	3044	5491	2834	13308	20443	9630
Solyc01g102400.2.1	-2.73309	5.50929	5.25E-09	1.54E-07	865	752	577	2039	4665	2425
Solyc02g094260.1.1	-2.73239	6.138471	1.14E-09	3.81E-08	1007	1554	1054	4094	4624	4486
Solyc10g055800.1.1	-2.72988	11.414	7.27E-06	9.29E-05	66974	35349	22194	233434	181389	111510
Solyc01g105300.2.1	-2.72836	0.554454	0.000112	0.000999	22	32	20	79	123	80
Solyc01g095620.2.1	-2.72659	9.155641	8.51E-08	1.85E-06	10211	12903	5876	47111	39136	23938
Solyc10g076660.1.1	-2.72561	5.36391	3.84E-05	0.000397	1156	270	328	3165	3730	1394
Solyc05g007220.2.1	-2.72329	7.013986	8.98E-10	3.08E-08	1813	2957	1943	7378	12338	6096
Solyc02g072210.1.1	-2.72309	2.388167	9.32E-07	1.55E-05	49	176	71	300	450	272
Solyc06g053670.1.1	-2.72273	6.856702	1.27E-09	4.19E-08	1469	3226	1572	8108	8431	5773
Solyc09g059400.2.1	-2.72085	-0.03977	0.001003	0.006356	5	37	14	60	56	61
Solyc07g066330.2.1	-2.71928	5.27119	2.18E-06	3.26E-05	349	1823	223	1533	4052	2153
Solyc12g014510.1.1	-2.71927	1.251382	2.40E-05	0.000262	24	67	38	106	233	130
Solyc12g010710.1.1	-2.71838	5.69262	4.75E-10	1.73E-08	659	1533	643	4013	4039	2090
Solyc02g082550.2.1	-2.71731	5.475378	5.36E-11	2.40E-09	600	1197	591	3176	3755	1859
Solyc01g109710.2.1	-2.71649	8.452489	1.33E-08	3.60E-07	5864	6496	5125	25496	25497	16612
Solyc03g117870.2.1	-2.71424	8.948483	3.14E-09	9.60E-08	6315	12721	7416	34470	41644	21338
Solyc12g036390.1.1	-2.71259	4.85545	1.47E-09	4.79E-08	212	1020	454	1909	2310	1413
Solyc08g078040.2.1	-2.7103	7.831274	2.72E-08	6.74E-07	2108	7495	3400	10506	24409	11193
Solyc04g011350.2.1	-2.7075	7.354249	7.53E-09	2.16E-07	2324	4214	2198	12393	10288	8281
Solyc08g007130.2.1	-2.70426	4.754504	0.000474	0.003374	843	281	59	1359	3204	1048
Solyc11g065990.1.1	-2.70348	2.966063	5.66E-08	1.29E-06	92	215	119	466	801	315
Solyc01g073840.1.1	-2.70264	1.0019	0.001351	0.008194	28	86	4	138	122	112
Solyc04g016230.2.1	-2.70083	5.442626	9.68E-06	0.000119	1116	679	256	2245	2304	3298
Solyc04g082810.2.1	-2.70065	1.973437	4.51E-05	0.000457	22	156	58	345	235	164
Solyc09g064530.2.1	-2.69983	5.210537	4.15E-09	1.24E-07	733	715	401	2676	3128	1509
RRNA_9_2	-2.6946	5.281812	1.76E-08	4.59E-07	764	749	436	1461	3891	2333
Solyc12g007030.1.1	-2.69353	8.868246	1.97E-07	3.93E-06	9787	8931	4539	29720	41078	21424
Solyc07g049190.2.1	-2.69304	5.06851	2.92E-11	1.38E-09	508	875	418	2197	2932	1494
Solyc06g062490.2.1	-2.69266	2.218109	3.17E-06	4.49E-05	77	78	77	218	425	264
Solyc03g025720.2.1	-2.69245	9.848103	0.000895	0.005779	29645	7852	2373	79440	65371	34336
Solyc03g007710.2.1	-2.68614	0.821305	0.00032	0.002433	17	52	29	42	183	119
Solyc01g060020.2.1	-2.68479	12.54448	2.19E-06	3.26E-05	136760	107505	49335	448207	360289	312904
Solyc01g103670.2.1	-2.67971	6.755902	1.67E-09	5.38E-08	1446	2824	1622	6822	7599	6073
Solyc08g079000.2.1	-2.67598	1.72345	0.000129	0.001121	84	49	24	186	298	162
Solyc03g032090.1.1	-2.67553	5.795799	4.12E-08	9.71E-07	415	1433	1258	2478	6146	2659
Solyc01g087990.2.1	-2.67523	1.883144	2.04E-05	0.000228	27	139	54	166	417	167
Solyc12g099790.1.1	-2.67442	3.051145	0.000558	0.003866	163	120	122	1172	180	167
Solyc02g093230.2.1	-2.6721	5.497458	2.83E-07	5.40E-06	874	1142	378	4257	3123	1418
Solyc07g063640.1.1	-2.66882	0.98815	0.000202	0.001642	30	36	35	74	275	70
Solyc03g114180.2.1	-2.66655	5.116848	0.000546	0.003796	849	823	110	4547	1116	788
Solyc09g008020.2.1	-2.66584	6.254987	1.94E-08	4.99E-07	1321	1726	978	6464	5224	3022
Solyc11g073110.1.1	-2.66508	3.508906	1.38E-07	2.86E-06	73	383	217	697	1050	508
Solyc11g042420.1.1	-2.66484	0.584755	0.000161	0.001351	16	34	30	92	146	59
Solyc02g092110.2.1	-2.66469	5.137988	1.07E-09	3.59E-08	552	1095	331	2061	2939	1833
Solyc03g113710.2.1	-2.66331	3.992175	2.49E-08	6.24E-07	310	387	144	851	1512	785
Solyc01g009160.2.1	-2.66224	4.98048	9.46E-09	2.63E-07	563	900	267	2377	2467	1308
Solyc01g073970.2.1	-2.66133	4.403844	5.37E-08	1.23E-06	313	241	491	1234	2064	931
Solyc01g104770.2.1	-2.66132	7.703227	7.81E-09	2.23E-07	2132	6511	3375	12951	18138	9811
Solyc01g107170.2.1	-2.66069	7.177619	1.32E-07	2.73E-06	1968	5316	1238	7518	13494	7500
Solyc12g094390.1.1	-2.66007	3.93369	3.36E-09	1.02E-07	199	439	218	1250	984	672
Solyc10g050220.1.1	-2.65953	2.758686	8.81E-05	0.00081	187	78	49	548	482	273
Solyc02g061770.2.1	-2.65793	6.917669	0.000673	0.004545	3941	1026	370	9944	8950	4614
Solyc09g009260.2.1	-2.65609	8.029469	1.70E-08	4.44E-07	2347	7557	5062	17405	21857	11842
Solyc11g008250.1.1	-2.65246	8.60742	6.88E-08	1.54E-06	6876	9411	4609	30964	31134	14464
Solyc11g011380.1.1	-2.65124	8.387823	6.00E-07	1.05E-05	2286	11314	6398	13438	39417	15795
Solyc03g034100.2.1	-2.64733	1.634154	0.00069	0.004637	71	38	40	349	93	118
Solyc06g083710.1.1	-2.64527	1.834547	3.04E-05	0.000324	26	129	59	298	209	161
Solyc04g005040.1.1	-2.63927	4.829207	7.97E-08	1.74E-06	471	976	198	2126	2208	1188
Solyc06g053260.1.1	-2.63789	7.965298	9.59E-06	0.000118	5970	5200	1650	21353	20711	7544
Solyc05g010140.2.1	-2.63725	5.81267	7.69E-07	1.31E-05	1344	877	557	4392	4155	2318

Solyc10g086410.2.1	-2.63495	7.764861	2.56E-07	4.96E-06	3553	5725	2727	20498	13829	7423
Solyc12g011090.1.1	-2.62938	0.875741	0.000162	0.001358	28	55	20	77	149	119
Solyc02g079570.2.1	-2.62845	7.571229	6.66E-08	1.50E-06	3422	5143	1943	11968	17826	8006
Solyc09g075670.1.1	-2.61524	4.249406	8.55E-08	1.86E-06	360	561	149	1300	1351	960
Solyc04g009600.2.1	-2.61416	3.943255	6.17E-10	2.19E-08	193	416	266	1036	1202	725
Solyc09g009690.2.1	-2.61333	7.330952	5.56E-05	0.000547	4676	1820	1126	13099	10858	6774
Solyc01g081540.2.1	-2.60969	6.578238	6.84E-09	1.97E-07	1475	2314	1504	7345	7211	3920
Solyc02g065550.2.1	-2.60838	6.229823	3.95E-07	7.27E-06	1591	1898	597	5088	6814	2966
Solyc05g009420.1.1	-2.60621	5.739366	7.50E-10	2.62E-08	653	1357	1018	3620	4560	2278
Solyc07g008360.1.1	-2.60466	0.102374	0.000876	0.005674	9	27	24	64	119	33
Solyc10g084400.1.1	-2.60215	8.755439	0.000172	0.001431	12293	5061	3375	46782	19768	14079
Solyc12g010480.1.1	-2.60101	2.011441	1.73E-05	0.000198	75	68	67	293	233	217
Solyc09g014860.2.1	-2.60031	7.627658	4.42E-08	1.04E-06	3606	5097	2288	12161	18060	8753
Solyc09g018230.1.1	-2.59758	5.318948	2.81E-09	8.67E-08	781	875	497	2904	3087	1719
Solyc09g010940.2.1	-2.59741	7.499556	4.20E-08	9.89E-07	2728	5464	2308	14070	13776	7196
Solyc09g089580.2.1	-2.59604	5.444634	5.89E-05	0.000576	309	1676	756	974	7902	1039
Solyc02g032930.2.1	-2.5939	6.92492	1.78E-08	4.64E-07	2173	2596	1823	8508	10054	5124
Solyc11g005420.1.1	-2.59085	3.125123	2.58E-07	5.00E-06	124	232	141	340	869	500
Solyc02g087580.2.1	-2.58972	3.943948	1.06E-07	2.26E-06	313	386	149	1179	1129	646
Solyc02g078480.2.1	-2.58937	5.292707	1.14E-07	2.40E-06	893	799	380	3019	2969	1583
Solyc10g078470.1.1	-2.58914	4.294	2.28E-07	4.48E-06	117	749	374	934	1649	1167
Solyc02g085300.2.1	-2.5883	4.165376	4.45E-07	8.08E-06	413	366	169	1446	1418	636
Solyc01g066760.2.1	-2.58457	3.7959	9.62E-09	2.67E-07	210	286	266	970	885	736
Solyc03g044190.2.1	-2.58299	6.796815	1.62E-08	4.28E-07	1743	2684	1812	8307	6893	5580
Solyc11g062400.1.1	-2.58274	6.077843	7.78E-09	2.22E-07	1151	1941	789	4309	5606	3169
Solyc09g065940.2.1	-2.5824	1.9026	1.39E-05	0.000163	72	71	56	204	309	201
Solyc09g061620.2.1	-2.58021	6.628024	5.62E-09	1.64E-07	1250	2790	1736	4877	9761	4907
Solyc01g109310.2.1	-2.57983	1.813295	9.15E-06	0.000113	49	103	52	174	339	175
Solyc05g041540.2.1	-2.57857	7.346246	1.18E-08	3.21E-07	2677	3914	2527	9070	14956	7856
Solyc04g051310.2.1	-2.57631	7.463031	1.06E-08	2.92E-07	2405	5043	2860	11488	15880	7362
Solyc11g072470.1.1	-2.57402	5.335413	4.82E-08	1.12E-06	918	838	403	2319	3493	2014
Solyc08g061480.2.1	-2.57219	6.802963	2.05E-07	4.05E-06	843	3354	2561	5808	11782	4840
Solyc04g077620.1.1	-2.57004	7.507025	4.79E-08	1.11E-06	3169	5210	2122	11824	14852	8468
Solyc02g082910.2.1	-2.56908	6.576065	9.89E-07	1.63E-05	2271	1325	1258	5002	8345	5094
Solyc08g079230.1.1	-2.56679	1.602315	0.000177	0.001465	43	91	44	220	92	211
Solyc10g018120.1.1	-2.56594	5.748409	1.58E-05	0.000182	316	2563	749	1466	6739	2790
Solyc06g069570.2.1	-2.56112	7.584858	4.96E-08	1.15E-06	3605	4666	2500	12774	15650	8687
Solyc01g109460.2.1	-2.55953	8.723244	3.96E-08	9.37E-07	4979	14620	6421	23336	33181	23747
Solyc02g092240.2.1	-2.55861	5.050432	1.30E-05	0.000153	859	497	337	3452	2002	884
Solyc02g032950.2.1	-2.55363	7.342191	3.29E-06	4.63E-05	3795	3956	1215	12124	12277	6792
Solyc01g109720.2.1	-2.5533	5.454875	2.40E-05	0.000262	1277	499	381	3772	2952	1642
Solyc08g080670.1.1	-2.54978	10.52075	0.001142	0.007106	48961	16652	5278	147347	67919	55844
Solyc06g024210.1.1	-2.54836	4.895977	3.68E-08	8.78E-07	506	499	608	1161	2459	1988
Solyc02g088090.1.1	-2.54814	7.750172	1.54E-07	3.12E-06	2516	8548	2670	11930	20996	9659
Solyc02g087740.2.1	-2.54804	3.938117	8.32E-09	2.35E-07	274	418	193	980	1358	657
Solyc04g011770.2.1	-2.54578	4.077485	5.22E-08	1.20E-06	364	342	213	1205	1221	780
Solyc11g017390.1.1	-2.54416	0.841498	0.000248	0.001959	24	40	37	87	213	64
Solyc10g084000.1.1	-2.54307	1.293825	0.000831	0.005425	19	135	16	120	274	99
Solyc01g091190.2.1	-2.53994	9.644137	5.71E-08	1.30E-06	8599	23107	16580	54177	66063	34719
Solyc10g017580.2.1	-2.53944	6.232533	4.00E-09	1.20E-07	938	2362	1275	4670	6710	3312
Solyc03g093130.2.1	-2.53886	2.961599	0.000802	0.005276	103	387	32	259	1298	173
Solyc04g064870.2.1	-2.53635	7.567166	7.98E-05	0.000746	5684	2993	1012	10435	18330	8629
Solyc07g054140.2.1	-2.53601	2.308628	3.76E-06	5.22E-05	71	104	105	291	427	237
Solyc08g076160.2.1	-2.53366	7.589882	1.41E-08	3.79E-07	2780	5571	3144	12022	16984	8548
Solyc04g054820.2.1	-2.53251	5.063179	5.05E-08	1.17E-06	687	786	408	2853	1942	1437
Solyc06g048810.2.1	-2.53094	5.962682	1.58E-09	5.09E-08	883	1750	1077	3644	5441	2997
Solyc12g010980.1.1	-2.52942	7.291271	1.79E-07	3.60E-06	1837	3597	3695	12339	9359	7424
Solyc03g113040.2.1	-2.52677	4.283437	2.58E-07	5.00E-06	406	515	197	1559	1645	620
Solyc04g080740.1.1	-2.52116	6.250708	3.09E-08	7.54E-07	762	2085	1763	5341	5820	3399
Solyc10g012170.2.1	-2.52074	5.212967	1.50E-09	4.87E-08	466	1308	554	1840	3777	1729
Solyc12g017530.1.1	-2.52073	5.856802	6.57E-09	1.90E-07	716	1862	998	4311	4418	2392
Solyc04g079350.1.1	-2.51988	6.527868	2.74E-08	6.77E-07	1429	2891	1282	4152	8531	5152
Solyc01g005220.2.1	-2.51867	4.376991	1.24E-09	4.08E-08	239	564	446	1127	1755	1097
Solyc02g070020.1.1	-2.51339	6.609219	5.13E-07	9.20E-06	2181	2256	1059	7408	6963	4179
Solyc02g078900.2.1	-2.51326	6.858903	2.04E-08	5.25E-07	1441	3536	2143	5460	10306	6523
Solyc03g031830.1.1	-2.51276	4.75432	2.14E-09	6.76E-08	519	705	345	1653	2276	1271
Solyc10g055560.1.1	-2.51057	1.540558	7.60E-05	0.000714	52	50	58	226	224	107
Solyc01g104880.2.1	-2.50813	6.65349	1.53E-08	4.06E-07	1532	2993	1572	7205	7372	4544
Solyc01g095970.2.1	-2.50745	6.44653	1.65E-08	4.33E-07	1504	2235	1373	5759	8032	3377
Solyc06g069670.2.1	-2.50332	1.991981	0.000178	0.001472	24	140	92	120	360	271
Solyc03g082370.1.1	-2.50257	7.071237	1.57E-06	2.44E-05	2485	4880	1019	9005	10452	6215
Solyc01g097040.2.1	-2.49733	6.764512	1.55E-08	4.12E-07	1448	3433	1863	7259	9382	4494
Solyc08g082280.2.1	-2.49647	8.982893	3.96E-08	9.37E-07	7521	15326	8135	34071	40750	22366
Solyc04g056340.2.1	-2.49027	6.329256	1.18E-06	1.91E-05	1825	1871	887	6824	4989	3256
Solyc03g112080.1.1	-2.4871	4.235683	2.58E-09	7.99E-08	290	514	331	1240	1747	717
Solyc01g079600.2.1	-2.48217	8.492683	1.90E-05	0.000214	10023	5435	3654	28336	26797	13801
Solyc01g088400.2.1	-2.47983	5.075985	6.14E-06	8.02E-05	469	852	707	634	4978	1504
Solyc04g016160.2.1	-2.47965	4.097921	3.70E-08	8.81E-07	361	440	202	991	1419	858
Solyc06g082440.1.1	-2.47799	5.50186	1.05E-05	0.000127	1213	1079	273	3432	3371	1838
Solyc08g005120.2.1	-2.47784	4.648954	1.17E-07	2.46E-06	505	645	327	2048	2154	729
Solyc08g077020.1.1	-2.47721	3.805798	1.67E-06	2.58E-05	175	655	124	966	1248	521
Solyc02g084970.2.1	-2.47679	3.383774	6.47E-07	1.13E-05	194	330	116	461	1034	540
Solyc08g067630.2.1	-2.47558	8.305255	1.06E-06	1.73E-05	2448	11660	6766	15712	33653	13704
RRNA_6_4	-2.47248	3.557984	1.76E-07	3.53E-06	213	265	209	497	999	725

Solyc06g076080.1.1	-2.47196	3.43915	8.10E-06	0.000102	127	538	89	517	1109	510
Solyc03g083130.1.1	-2.46686	4.685006	1.83E-09	5.85E-08	373	832	414	1902	2027	1010
Solyc12g042210.1.1	-2.46493	0.849386	0.001063	0.006663	23	45	40	194	92	47
Solyc04g077230.1.1	-2.46084	5.840419	3.33E-08	8.04E-07	896	2032	741	3814	3856	2974
Solyc08g014150.2.1	-2.4595	7.198511	6.70E-08	1.51E-06	2754	3865	2199	9742	11837	6524
Solyc01g009810.2.1	-2.45828	3.812329	0.000213	0.001715	310	523	54	361	1615	806
Solyc07g056390.2.1	-2.45821	6.456372	1.91E-08	4.93E-07	1437	2269	1604	6215	6382	3981
Solyc08g078850.2.1	-2.45299	4.140393	5.30E-07	9.47E-06	329	470	265	1059	748	1259
Solyc09g089780.2.1	-2.45284	2.081903	0.000492	0.003477	15	214	76	191	333	263
Solyc04g079820.2.1	-2.45214	3.557442	0.000759	0.005034	420	90	72	770	1059	463
Solyc02g078170.1.1	-2.45034	5.65839	4.50E-08	1.05E-06	1044	1408	600	3470	4039	2154
NCRNA_11_155	-2.44904	1.474841	0.000204	0.00166	57	69	38	89	261	176
Solyc08g014170.1.1	-2.44815	2.387169	5.01E-06	6.73E-05	77	170	84	365	390	232
Solyc11g056620.1.1	-2.44682	6.099673	1.43E-08	3.84E-07	1224	1864	1093	3860	6407	3082
Solyc10g062160.1.1	-2.44663	4.826156	1.19E-08	3.24E-07	305	1100	490	1367	2974	1273
Solyc01g112070.2.1	-2.4455	3.144635	0.001286	0.007851	48	321	224	80	1207	514
Solyc07g005330.2.1	-2.4428	6.56386	1.18E-07	2.47E-06	1672	2355	1679	7626	6905	3492
Solyc08g081230.1.1	-2.4419	7.010715	9.06E-07	1.51E-05	2108	5264	1196	7804	11005	5948
Solyc01g086650.2.1	-2.44153	5.510934	4.81E-08	1.12E-06	1021	893	699	2656	3538	2389
Solyc10g006710.2.1	-2.43722	5.855093	3.94E-07	7.26E-06	1366	1097	832	3940	4019	2840
Solyc02g089900.1.1	-2.43721	6.300372	2.32E-06	3.42E-05	821	3903	884	3705	8193	3647
Solyc04g050440.2.1	-2.43579	1.441318	0.001219	0.007499	73	52	27	280	112	97
Solyc03g026360.1.1	-2.43486	0.482496	0.001259	0.007705	34	25	19	88	82	80
Solyc12g036330.1.1	-2.43346	4.132401	2.05E-06	3.08E-05	467	308	204	824	1589	943
Solyc03g119480.2.1	-2.43324	5.585041	1.11E-08	3.05E-07	856	1552	629	3204	3438	2343
Solyc02g082250.2.1	-2.43043	7.698726	7.11E-08	1.58E-06	3018	6379	3813	14250	17452	8342
Solyc09g089850.2.1	-2.42943	4.013763	8.04E-09	2.29E-07	242	442	321	783	1588	776
Solyc03g111720.2.1	-2.42916	8.1046	6.31E-07	1.10E-05	6108	5509	4323	17002	25905	10967
RRNA_6_3	-2.42891	5.312051	1.66E-07	3.34E-06	840	749	705	1495	3449	2528
Solyc07g056410.2.1	-2.42782	3.89843	2.75E-06	3.97E-05	378	288	183	869	1642	494
Solyc02g086880.2.1	-2.42368	11.80687	2.03E-06	3.05E-05	82216	72231	53223	267758	229550	166902
Solyc05g006660.2.1	-2.4218	7.056149	3.60E-08	8.62E-07	2270	3713	2300	7908	11526	6117
Solyc09g008190.2.1	-2.41957	3.84634	1.54E-08	4.08E-07	205	397	301	734	1313	717
Solyc04g048950.2.1	-2.41926	6.194291	1.83E-06	2.81E-05	1845	1511	872	5454	4871	3314
Solyc12g006270.1.1	-2.41837	1.107441	0.000261	0.002046	37	49	43	177	131	88
Solyc04g008450.2.1	-2.4139	3.963203	6.42E-08	1.45E-06	322	386	235	1017	1039	819
Solyc03g118720.2.1	-2.4135	6.127174	1.45E-08	3.87E-07	1215	1990	1173	4279	5834	3227
Solyc11g030730.1.1	-2.41319	3.624058	2.85E-05	0.000306	121	696	100	555	1303	571
Solyc03g082500.2.1	-2.41307	6.382658	3.64E-08	8.69E-07	1500	2481	1273	5178	7499	3486
Solyc03g005470.2.1	-2.41235	8.504594	6.76E-06	8.71E-05	9715	6392	4411	24750	27892	16127
Solyc07g008140.2.1	-2.41188	4.741576	0.000301	0.002305	80	1669	347	1167	3211	1050
Solyc05g056140.2.1	-2.41006	6.199797	1.93E-07	3.85E-06	1600	2003	900	4206	6021	3695
Solyc12g019890.1.1	-2.40918	5.494081	6.51E-07	1.13E-05	1166	800	597	2831	3859	1961
Solyc09g074250.2.1	-2.40614	5.454122	3.66E-09	1.10E-07	625	1363	842	2026	4261	2205
Solyc01g014840.2.1	-2.39894	4.982362	1.48E-06	2.33E-05	195	1444	636	1515	2658	1782
Solyc03g119440.2.1	-2.3956	7.827739	1.46E-07	2.99E-06	2798	7904	4462	14402	19514	9711
Solyc01g096420.2.1	-2.39461	7.553226	0.00047	0.003349	6103	3867	696	9848	14376	10716
Solyc03g044300.2.1	-2.38922	3.477048	1.86E-06	2.86E-05	211	283	195	1010	702	371
Solyc06g065910.2.1	-2.38919	1.05614	0.000252	0.001984	34	48	45	149	170	76
Solyc02g072190.2.1	-2.38815	4.530066	5.31E-08	1.22E-06	218	914	469	1157	2012	1217
Solyc09g011490.2.1	-2.38363	4.75866	6.71E-08	1.51E-06	452	1101	292	1542	2377	1261
Solyc01g087560.2.1	-2.38353	8.629028	7.79E-06	9.84E-05	10765	7636	4545	24513	33503	17615
Solyc07g043550.2.1	-2.38344	5.53742	1.00E-05	0.000122	332	2087	926	1295	4539	2999
Solyc01g067640.2.1	-2.38334	5.888986	1.72E-08	4.49E-07	1027	1600	1113	3987	4397	2732
Solyc03g033680.1.1	-2.38051	0.128546	0.001179	0.007298	16	35	19	49	95	59
Solyc03g053090.1.1	-2.38041	1.841525	7.79E-05	0.00073	78	78	59	233	340	125
Solyc08g067410.1.1	-2.37836	6.914821	1.22E-07	2.55E-06	2319	3346	1949	7920	10450	4852
Solyc01g066720.2.1	-2.37739	7.385119	1.17E-07	2.46E-06	3025	5109	2637	10480	13275	7817
Solyc06g076820.1.1	-2.37713	4.773397	2.53E-09	7.86E-08	497	806	447	1573	2086	1441
Solyc11g012790.1.1	-2.37655	6.117287	2.15E-08	5.49E-07	1151	2063	1264	4481	5837	2951
Solyc01g086850.2.1	-2.37627	5.084209	5.66E-09	1.65E-07	649	960	541	2446	2464	1456
Solyc07g007170.2.1	-2.37611	6.019765	1.07E-07	2.27E-06	1166	2288	841	4223	5594	2649
Solyc01g005470.2.1	-2.37526	9.601392	1.94E-06	2.95E-05	16338	19787	12004	64673	46115	32309
Solyc12g005980.1.1	-2.37264	7.395449	9.52E-07	1.58E-05	3832	4412	2177	11525	13067	7246
Solyc01g067070.2.1	-2.37218	6.753038	5.44E-08	1.25E-06	1907	3200	1837	6793	8487	5054
Solyc09g091990.2.1	-2.37088	5.51834	2.68E-09	8.28E-08	683	1434	892	2717	3952	2083
Solyc01g008130.2.1	-2.36977	4.69168	7.34E-08	1.62E-06	310	1057	437	1033	2659	1330
Solyc03g096390.2.1	-2.36823	7.443853	1.45E-07	2.96E-06	2823	5912	2830	9427	16442	7816
Solyc04g071580.2.1	-2.36627	2.76168	8.22E-06	0.000103	119	185	127	254	766	320
Solyc01g081310.2.1	-2.36579	5.87935	4.81E-06	6.49E-05	695	2436	1003	1514	6734	3321
Solyc02g078840.2.1	-2.36392	6.320509	3.34E-08	8.05E-07	1295	2569	1403	4320	7316	3720
Solyc03g044380.2.1	-2.36367	6.339136	6.68E-08	1.50E-06	1470	2204	1480	5753	5960	3486
Solyc09g091660.2.1	-2.35942	10.85698	1.43E-06	2.27E-05	27306	78997	23948	126112	153331	74729
Solyc08g079010.1.1	-2.35718	1.84308	0.00024	0.001903	97	79	38	238	306	140
Solyc12g098910.1.1	-2.35645	6.451559	0.000164	0.001378	2930	1437	562	6003	6428	3943
Solyc03g112090.2.1	-2.35534	0.83201	0.000538	0.003754	21	58	39	75	191	80
Solyc08g068780.1.1	-2.35476	5.013915	2.43E-06	3.57E-05	767	802	426	3084	1671	1152
Solyc06g005810.2.1	-2.35345	4.618803	2.65E-09	8.22E-08	386	741	485	1345	2227	1136
Solyc06g053550.2.1	-2.35322	3.206182	3.25E-06	4.60E-05	100	343	195	398	1028	401
Solyc02g071870.2.1	-2.35209	6.989556	5.45E-07	9.69E-06	2898	3261	1768	8029	9906	5946
Solyc04g016260.2.1	-2.35164	7.537864	2.19E-07	4.31E-06	3467	5554	3044	10431	17654	7931
Solyc06g053710.2.1	-2.3515	8.077388	4.14E-07	7.59E-06	3287	10703	4923	13102	25684	13192
Solyc08g078190.1.1	-2.34674	5.119891	1.53E-07	3.10E-06	415	1486	578	1319	3064	2133
Solyc08g062360.2.1	-2.34629	5.879181	2.01E-05	0.000225	1388	2110	391	2422	6078	2941

Solyc12g010440.1.1	-2.34173	4.838338	2.46E-07	4.80E-06	657	842	333	2124	2163	1112
Solyc05g006510.1.1	-2.34088	5.382402	3.26E-06	4.61E-05	1182	652	577	2630	3564	1765
Solyc04g071890.2.1	-2.3388	9.622194	2.30E-06	3.40E-05	13936	20886	15906	68877	42668	32034
Solyc11g013120.1.1	-2.33783	5.679074	2.29E-06	3.38E-05	334	2093	1274	3044	4102	2479
Solyc11g012260.1.1	-2.33401	5.585492	7.85E-08	1.72E-06	908	1597	696	3714	3264	1954
Solyc06g036260.2.1	-2.33392	6.064735	2.32E-05	0.000254	1844	1890	469	4138	6158	2641
Solyc10g079860.1.1	-2.33305	7.452327	0.000368	0.00273	1729	10667	1427	4268	14122	13405
Solyc01g091330.2.1	-2.33222	2.02931	6.38E-05	0.000616	44	129	103	276	241	217
Solyc06g076450.2.1	-2.33163	4.438109	1.33E-08	3.60E-07	368	777	331	1204	2022	947
Solyc01g059880.2.1	-2.32452	7.383609	3.27E-07	6.14E-06	2702	5498	3079	12258	13328	6170
Solyc02g090330.2.1	-2.32089	2.906055	3.83E-05	0.000396	162	182	129	686	320	324
Solyc04g015820.1.1	-2.31976	0.72657	0.001489	0.008889	25	64	25	153	69	69
Solyc04g008110.2.1	-2.31801	1.907554	5.39E-05	0.000533	45	135	80	241	332	144
Solyc08g082440.2.1	-2.317	7.514865	5.81E-07	1.02E-05	3616	5736	2788	12864	14425	7292
Solyc06g063280.1.1	-2.31441	1.308994	0.000448	0.003223	23	98	55	100	191	159
Solyc03g097030.2.1	-2.31422	4.744753	2.28E-08	5.79E-07	430	1014	423	1926	1813	1207
Solyc01g096230.2.1	-2.3105	5.34252	4.22E-09	1.26E-07	700	1304	733	2497	3498	1729
Solyc12g056360.1.1	-2.30931	3.996165	9.00E-07	1.50E-05	223	771	187	979	1356	692
Solyc04g078630.2.1	-2.30815	5.46455	5.57E-08	1.27E-06	897	1402	645	3170	3417	1737
Solyc02g087350.1.1	-2.30444	7.048898	4.10E-05	0.00042	1571	7390	1311	5061	15553	5759
Solyc01g008140.2.1	-2.30339	6.866884	3.27E-07	6.14E-06	2308	3449	1968	8428	8647	4771
Solyc10g049240.1.1	-2.30187	2.393798	3.78E-05	0.000392	117	122	93	357	315	273
Solyc09g009840.1.1	-2.30066	6.628533	2.51E-07	4.88E-06	1681	3424	1700	4347	9055	5320
Solyc02g093990.2.1	-2.30052	1.506082	0.000703	0.004717	76	83	23	201	200	122
Solyc04g016190.1.1	-2.2999	2.405779	0.000651	0.004418	91	257	41	523	217	201
Solyc06g051620.2.1	-2.2991	4.600986	1.75E-08	4.57E-07	433	812	409	1617	1589	1219
Solyc09g008250.2.1	-2.29752	4.457381	1.27E-08	3.45E-07	329	746	442	1317	1472	1203
Solyc12g009520.1.1	-2.29709	5.120734	3.26E-08	7.91E-07	715	1094	518	1590	3088	1872
Solyc01g067380.2.1	-2.29493	6.280414	6.12E-06	8.00E-05	2097	1822	942	5783	5818	2992
Solyc06g065310.2.1	-2.29277	3.117172	1.68E-05	0.000193	145	289	160	817	441	312
Solyc01g094960.2.1	-2.28925	6.764193	1.33E-07	2.75E-06	1673	3922	2024	6081	9210	5188
Solyc04g074730.1.1	-2.2891	7.332766	3.12E-06	4.43E-05	2679	4811	3399	5003	17765	8590
Solyc10g085810.1.1	-2.28795	5.213353	4.66E-08	1.09E-06	764	1059	631	2745	2855	1389
Solyc04g012010.2.1	-2.28713	1.769914	9.69E-05	0.000879	45	123	71	147	310	183
Solyc10g008890.2.1	-2.28609	4.382486	4.01E-05	0.000413	602	453	233	1973	960	802
Solyc02g080080.2.1	-2.2854	6.546257	7.00E-06	8.96E-05	1718	3746	1168	2888	9573	5403
Solyc10g049890.1.1	-2.2853	3.484701	4.17E-07	7.62E-06	149	374	256	569	1049	519
Solyc06g051540.2.1	-2.2851	5.072455	1.73E-08	4.50E-07	615	1195	522	2344	2658	1337
Solyc01g009820.2.1	-2.28504	6.528422	1.32E-07	2.73E-06	1750	2906	1595	5579	7572	4205
Solyc11g068430.1.1	-2.2837	6.69975	1.63E-07	3.29E-06	1431	3625	2245	6456	8313	4716
Solyc07g056110.2.1	-2.28106	6.141682	1.04E-07	2.21E-06	1265	2294	1275	3638	6853	3109
Solyc01g087070.2.1	-2.28036	2.888191	4.47E-05	0.000453	85	309	147	209	701	474
Solyc07g005520.1.1	-2.27961	4.313905	1.43E-05	0.000166	604	344	246	922	1932	955
Solyc04g078290.2.1	-2.27955	7.454273	2.27E-06	3.37E-05	3320	7152	2032	10432	14072	8293
Solyc11g066860.1.1	-2.27942	6.590778	8.35E-05	0.000776	1059	3903	2083	1669	11566	5650
Solyc06g076440.1.1	-2.27782	4.572099	4.34E-07	7.91E-06	555	643	355	1870	1524	973
Solyc10g005630.2.1	-2.27526	6.396666	2.74E-07	5.26E-06	1050	3362	1710	4249	7203	4342
Solyc04g072930.1.1	-2.27404	5.608126	1.47E-07	3.00E-06	1125	1324	748	3211	3750	2142
Solyc08g008330.2.1	-2.2726	5.56402	1.66E-08	4.35E-07	836	1329	1015	2603	3713	2449
Solyc06g008890.2.1	-2.27159	4.10193	1.43E-07	2.94E-06	257	572	368	1369	1139	662
Solyc12g096570.1.1	-2.27095	5.380115	3.15E-05	0.000334	197	2051	1001	1956	3848	2093
Solyc06g076050.2.1	-2.27031	5.145409	5.56E-07	9.86E-06	904	921	464	2479	2380	1627
Solyc11g015890.1.1	-2.26751	3.55489	3.91E-06	5.41E-05	289	344	144	777	992	458
Solyc06g075580.2.1	-2.26619	4.567127	8.76E-05	0.000807	664	282	455	2193	1024	983
Solyc04g064860.1.1	-2.26572	3.280213	6.12E-06	8.00E-05	226	316	115	601	795	426
Solyc08g079520.2.1	-2.26546	7.050685	2.85E-07	5.43E-06	2433	4312	2394	7159	12587	5702
Solyc06g050810.2.1	-2.26377	4.842051	2.02E-06	3.05E-05	786	714	337	2045	1998	1245
Solyc08g069060.2.1	-2.26271	7.820694	2.67E-06	3.86E-05	5305	6580	3165	14599	19885	8682
Solyc08g081480.2.1	-2.26175	5.531829	3.17E-07	5.96E-06	830	914	1241	2402	4566	1962
Solyc03g007290.2.1	-2.26075	3.401761	7.81E-06	9.87E-05	230	365	133	850	758	365
Solyc08g081370.1.1	-2.26073	5.200464	1.37E-08	3.70E-07	491	1273	847	1997	3121	1781
Solyc08g077550.2.1	-2.26018	3.634076	5.26E-07	9.40E-06	255	316	248	828	1037	483
Solyc09g074480.1.1	-2.25779	1.182536	0.000576	0.003973	50	75	29	127	146	131
Solyc06g064660.2.1	-2.25768	3.396351	8.43E-07	1.42E-05	157	296	266	606	782	544
Solyc03g123560.2.1	-2.25598	6.285533	1.72E-05	0.000196	2222	1986	806	4417	4951	4594
Solyc02g084650.2.1	-2.2535	5.643989	7.07E-08	1.57E-06	1064	1371	905	3287	3835	2188
Solyc02g092990.1.1	-2.25041	6.724441	1.90E-06	2.91E-05	2170	4000	1324	5121	10297	4851
Solyc12g005340.1.1	-2.24923	7.032534	6.95E-06	8.91E-05	2551	5783	1284	7221	11564	5972
Solyc03g115770.2.1	-2.24817	3.736994	0.000152	0.001288	142	173	541	545	1531	555
Solyc12g017480.1.1	-2.247	3.092941	4.67E-05	0.00047	191	291	107	766	438	328
Solyc07g040890.1.1	-2.24693	4.564546	8.63E-05	0.000797	623	901	124	1763	1718	918
Solyc02g089110.2.1	-2.24631	5.996912	2.29E-07	4.49E-06	1344	1945	1067	4266	5009	2668
Solyc09g092000.2.1	-2.24577	5.326411	8.47E-09	2.39E-07	702	1319	785	2131	3296	2036
Solyc05g012850.2.1	-2.2437	5.010961	1.27E-05	0.00015	375	1511	579	770	3926	1663
Solyc06g071990.2.1	-2.24325	5.472498	8.64E-08	1.88E-06	606	1239	1225	2172	3953	2226
Solyc04g080820.2.1	-2.24058	8.964626	3.51E-06	4.91E-05	5344	17562	13372	34466	40688	19089
RRNA_11_2	-2.2392	7.099589	6.06E-06	7.94E-05	3019	2979	2926	5171	10627	9041
Solyc01g007910.2.1	-2.23916	7.745704	7.76E-07	1.32E-05	3154	7847	4498	9900	19848	10819
Solyc04g079840.2.1	-2.23768	5.130583	4.56E-08	1.06E-06	775	1059	554	1954	3152	1537
Solyc01g073740.2.1	-2.23742	8.948351	5.52E-07	9.81E-06	8777	15408	10194	31120	37315	22943
Solyc09g089640.2.1	-2.23703	6.118981	5.74E-07	1.01E-05	1387	2501	1033	3121	6411	3577
Solyc11g008720.1.1	-2.23684	4.079072	3.88E-07	7.15E-06	380	370	350	990	1551	683
Solyc01g095490.2.1	-2.23657	5.633252	7.55E-08	1.66E-06	974	1428	987	3460	3991	1890
Solyc03g044900.2.1	-2.23405	6.56025	6.19E-07	1.08E-05	1907	2291	2088	6432	6926	4095

Solyc09g098070.2.1	-2.23355	6.596947	2.80E-07	5.35E-06	1883	3229	1674	5981	7888	4249
Solyc06g060700.1.1	-2.22868	1.470521	0.000375	0.002772	40	132	38	140	229	142
Solyc03g116890.2.1	-2.22815	7.138836	3.26E-06	4.61E-05	2798	5869	1651	8313	11771	6330
Solyc09g082470.2.1	-2.22758	7.41826	5.37E-07	9.57E-06	3305	5069	3429	10294	14828	7202
RRNA_11_14	-2.22652	7.18451	4.00E-06	5.51E-05	3186	3187	3172	5993	11786	8846
Solyc07g062620.2.1	-2.2254	7.065821	3.39E-07	6.34E-06	2038	4360	3130	7571	10637	6601
Solyc02g083380.2.1	-2.22395	3.879694	2.87E-05	0.000308	462	282	175	826	1069	784
Solyc06g065570.2.1	-2.2231	6.717799	8.25E-07	1.40E-05	2390	3035	1739	5700	9194	4899
Solyc02g088460.2.1	-2.21698	8.215108	2.61E-06	3.79E-05	7051	7672	5171	20362	21749	12762
Solyc07g043310.2.1	-2.21663	9.369961	2.33E-06	3.43E-05	14894	19780	10805	37950	61830	26683
Solyc10g083230.1.1	-2.21586	0.714498	0.001166	0.007235	38	46	26	103	128	70
Solyc12g005220.1.1	-2.21522	4.802254	3.18E-08	7.74E-07	497	889	583	1230	2534	1465
Solyc10g080770.1.1	-2.21518	8.221569	1.56E-06	2.43E-05	6554	9484	4737	18331	26010	12131
Solyc11g010420.1.1	-2.21491	1.98472	0.00013	0.001132	95	111	61	231	285	202
Solyc03g097050.2.1	-2.21084	3.029614	1.04E-05	0.000126	134	249	192	611	676	259
Solyc03g083590.2.1	-2.20921	2.443425	2.55E-05	0.000276	77	187	129	278	496	250
Solyc08g062860.2.1	-2.20814	5.508623	1.02E-07	2.18E-06	889	1531	816	3349	3243	1810
Solyc09g090040.2.1	-2.20808	7.571796	7.87E-06	9.92E-05	5049	4772	2798	12919	15019	7603
Solyc05g051050.2.1	-2.20593	4.82519	4.37E-07	7.94E-06	688	790	448	1362	3003	1144
Solyc08g078960.2.1	-2.20589	5.660036	3.02E-07	5.72E-06	1122	1448	905	3658	3831	1926
Solyc08g063030.2.1	-2.20577	1.19352	0.001275	0.007788	50	40	57	191	137	84
Solyc01g112220.2.1	-2.20574	5.732684	5.99E-06	7.86E-05	1354	1757	587	3993	3750	2068
Solyc04g009920.2.1	-2.20446	6.673868	3.68E-07	6.85E-06	1757	3512	2089	6693	8340	4106
Solyc09g008200.2.1	-2.20299	8.946347	8.28E-07	1.40E-05	7542	17112	11209	29926	39866	22050
Solyc10g054930.1.1	-2.20054	4.313447	4.66E-08	1.09E-06	343	698	398	1294	1519	864
Solyc06g068650.2.1	-2.19981	6.8496	1.61E-05	0.000186	910	4882	3128	6125	11846	4395
Solyc02g093830.2.1	-2.19971	8.893677	9.29E-07	1.55E-05	7378	17700	9967	30176	36308	21386
Solyc09g061280.2.1	-2.19897	3.867798	8.60E-07	1.45E-05	260	529	272	612	1620	616
Solyc05g005190.2.1	-2.19882	7.926293	1.13E-06	1.83E-05	3813	8104	5659	17191	16265	10833
Solyc04g054150.1.1	-2.19831	2.097286	9.71E-05	0.00088	99	111	79	211	363	217
Solyc03g117350.1.1	-2.1973	4.609047	3.71E-08	8.83E-07	432	804	512	1669	1885	982
Solyc05g051570.2.1	-2.19605	6.45677	4.44E-07	8.06E-06	1849	2817	1536	5141	7533	3814
Solyc11g068460.1.1	-2.19207	5.669724	3.53E-07	6.60E-06	1207	1365	900	3256	4111	2127
Solyc09g075450.2.1	-2.1894	8.306106	3.79E-06	5.25E-05	7913	8610	5046	18771	24491	15049
Solyc05g054800.1.1	-2.18913	8.017726	1.92E-06	2.93E-05	5121	9834	4045	16809	21741	10174
RRNA_11_1	-2.18866	6.148259	2.24E-06	3.32E-05	1582	1854	1417	2669	6300	4151
Solyc03g113380.2.1	-2.18776	6.1434	6.18E-07	1.08E-05	1565	2295	1144	4449	5790	2962
Solyc08g062820.2.1	-2.18742	6.543827	2.06E-06	3.09E-05	2260	2208	1794	4762	8593	4264
Solyc02g062500.2.1	-2.1867	4.171118	0.000506	0.003555	88	966	432	486	2558	653
Solyc04g016270.1.1	-2.18647	4.828467	2.74E-07	5.25E-06	709	781	452	1462	2528	1337
Solyc01g102700.2.1	-2.18554	5.831993	7.62E-07	1.30E-05	1272	1962	841	3635	3960	2752
Solyc10g074740.1.1	-2.18492	7.54778	5.08E-06	6.80E-05	3149	8556	2701	13446	13745	7393
RRNA_SL2.40sc04870_1	-2.18444	7.130703	7.82E-06	9.87E-05	3024	3012	3363	5595	10794	8889
Solyc12g014430.1.1	-2.18433	6.407033	4.22E-05	0.000431	619	2606	3040	5571	5166	4397
Solyc09g074320.2.1	-2.18393	8.287203	1.05E-06	1.72E-05	4613	12040	6750	19433	25950	13058
Solyc12g005200.1.1	-2.18385	6.447837	3.39E-05	0.000357	2494	2021	1248	7491	4819	3375
Solyc01g107900.2.1	-2.18342	9.483844	4.33E-05	0.000441	21342	18541	7903	47368	53680	30978
Solyc08g080680.2.1	-2.18138	6.826188	1.20E-06	1.94E-05	2564	2900	2299	7042	8814	5103
Solyc03g114600.2.1	-2.18076	5.689168	7.08E-07	1.22E-05	964	2162	758	3666	4119	1876
Solyc07g065250.2.1	-2.18035	5.266058	5.30E-07	9.47E-06	466	1577	901	1617	4197	1657
Solyc12g087950.1.1	-2.17822	6.158207	9.43E-07	1.57E-05	1691	2086	1193	4427	5170	3429
Solyc06g069150.1.1	-2.17821	5.394143	0.00013	0.001128	202	2147	1099	1474	3708	2557
Solyc03g123370.2.1	-2.17761	9.970095	4.19E-06	5.75E-05	20783	36850	15657	71697	74951	39084
Solyc08g080640.1.1	-2.17661	9.456131	2.33E-05	0.000255	17419	15788	13816	57012	35832	31447
Solyc04g015570.2.1	-2.17606	4.809344	5.68E-06	7.51E-05	824	742	321	1813	2171	1200
Solyc10g079130.1.1	-2.17568	8.075096	1.54E-06	2.41E-05	5311	9385	4904	18830	20874	10444
Solyc04g074710.2.1	-2.17403	4.030442	4.35E-07	7.92E-06	255	720	282	777	1486	798
Solyc11g007890.1.1	-2.17305	3.375118	5.20E-05	0.000517	209	508	88	541	858	516
Solyc07g063180.2.1	-2.17048	2.267395	0.000106	0.000949	55	184	125	271	318	268
Solyc11g069600.1.1	-2.16872	7.083034	6.75E-07	1.17E-05	2470	4358	2977	6835	12074	6462
Solyc08g081680.1.1	-2.16724	2.25016	0.001011	0.006399	144	54	96	235	497	184
Solyc07g054830.2.1	-2.16555	6.788757	5.72E-07	1.01E-05	1684	4289	2381	6444	8941	5078
Solyc04g049090.2.1	-2.16472	9.791152	3.73E-05	0.000387	25381	25452	10065	60233	69070	35192
Solyc12g099340.1.1	-2.16471	6.614398	2.93E-06	4.19E-05	2458	2527	1728	6459	7719	4006
Solyc01g104620.2.1	-2.16439	2.284222	0.000138	0.001192	109	146	88	206	327	323
Solyc08g075230.1.1	-2.16105	1.653175	0.000348	0.002607	36	100	95	158	247	166
Solyc09g074340.2.1	-2.15991	3.310115	7.44E-06	9.47E-05	184	352	194	796	607	387
Solyc02g088630.2.1	-2.15963	5.491279	7.58E-08	1.67E-06	886	1340	987	2518	4089	1882
Solyc11g068770.1.1	-2.15836	6.163649	1.94E-06	2.95E-05	780	3197	1683	4315	5517	3333
Solyc06g074960.2.1	-2.15829	5.810198	3.04E-07	5.75E-06	1138	1920	1035	3652	4019	2557
Solyc03g115850.2.1	-2.1573	4.722562	7.41E-07	1.27E-05	297	1285	538	1272	2401	1270
Solyc01g097330.2.1	-2.15682	6.601228	1.09E-05	0.000131	2609	2546	1499	7446	6168	3961
Solyc04g016460.2.1	-2.15661	2.108664	0.000284	0.002191	119	122	56	204	355	229
Solyc07g066080.2.1	-2.15409	6.688717	3.21E-06	4.55E-05	1174	4294	2571	4878	9674	4870
Solyc07g049700.1.1	-2.15252	3.66923	1.20E-06	1.94E-05	267	451	215	633	1216	558
Solyc04g083160.1.1	-2.15119	3.015952	2.04E-05	0.000228	183	203	174	371	606	476
Solyc08g014570.2.1	-2.15035	7.356367	1.42E-06	2.24E-05	2930	5988	3319	11173	12836	6402
Solyc03g097230.1.1	-2.14905	3.315511	0.000772	0.005105	42	269	429	570	746	494
Solyc03g112970.1.1	-2.14806	3.174225	3.71E-05	0.000386	241	295	106	519	665	452
Solyc12g044940.1.1	-2.14752	6.948114	4.32E-05	0.00044	3563	3875	1240	8825	8817	4981
Solyc05g053330.2.1	-2.14614	5.58099	4.86E-07	8.75E-06	737	2277	786	2531	4267	2162
Solyc05g018050.1.1	-2.14555	1.681979	0.000972	0.006188	38	138	74	303	147	114
Solyc01g097270.2.1	-2.14366	10.9863	0.000127	0.00111	64249	43487	26441	154923	110681	93416
Solyc01g097220.1.1	-2.14182	2.169939	0.00024	0.001905	91	102	118	162	450	233

Solyc06g062990.1.1	-2.14148	5.8767	2.96E-07	5.61E-06	1022	2319	1134	3178	5138	2649
Solyc06g075190.1.1	-2.14078	6.49842	9.44E-07	1.57E-05	1898	3266	1538	5351	6701	4398
Solyc02g086770.2.1	-2.13934	2.423327	0.000832	0.005432	166	138	60	453	236	241
Solyc01g080800.2.1	-2.13891	4.457554	1.12E-05	0.000134	518	917	225	945	2390	914
Solyc07g007820.2.1	-2.1378	7.350344	1.15E-06	1.86E-05	3052	5924	3240	10025	12894	7171
Solyc05g056200.1.1	-2.13645	4.5325	2.85E-07	5.43E-06	529	739	405	1190	2231	969
Solyc07g062670.1.1	-2.12957	1.855143	0.000518	0.003625	79	136	54	166	438	112
Solyc06g008600.2.1	-2.12548	6.213309	4.88E-06	6.58E-05	1758	2177	1377	5941	4518	2894
Solyc09g059040.2.1	-2.12453	8.558671	2.20E-06	3.28E-05	6963	15192	6830	21333	29273	18277
Solyc08g081240.2.1	-2.12386	7.109536	1.59E-06	2.47E-05	2579	5168	2720	9333	10893	5353
Solyc11g065940.1.1	-2.12192	7.411379	2.76E-06	3.98E-05	2244	7058	4087	9139	13686	8371
Solyc05g013880.1.1	-2.12149	4.900995	1.94E-05	0.000218	900	868	326	2251	1873	1245
Solyc01g105370.2.1	-2.11907	7.982626	8.66E-05	0.0008	8229	5951	2922	15907	20101	10665
Solyc10g054030.1.1	-2.11858	6.216959	1.89E-06	2.90E-05	1631	2626	1280	5360	5324	2918
Solyc07g049580.2.1	-2.11535	4.153631	1.91E-06	2.91E-05	433	390	407	1185	1373	716
Solyc11g007590.1.1	-2.11495	6.644227	2.06E-06	3.09E-05	2167	3046	2077	7102	6620	4316
Solyc07g045350.2.1	-2.11336	9.256262	2.17E-05	0.000239	7369	20885	18589	47623	34339	26510
Solyc01g008300.1.1	-2.11171	4.697559	2.45E-06	3.58E-05	476	730	686	2242	1688	807
Solyc01g106500.2.1	-2.11075	6.913173	1.47E-06	2.32E-05	2014	5001	2414	5963	10539	5823
Solyc01g010060.2.1	-2.10944	3.39923	0.000116	0.001026	127	640	141	387	1074	529
Solyc08g077920.2.1	-2.10839	6.837543	2.59E-06	3.76E-05	1505	4449	2993	5521	9763	5770
Solyc03g114360.2.1	-2.10768	5.950263	1.54E-06	2.41E-05	1446	2199	967	3700	4470	3002
Solyc06g072610.2.1	-2.10676	6.148136	6.96E-07	1.20E-05	1359	2599	1434	4645	5684	2794
Solyc10g085000.1.1	-2.1062	6.411473	6.42E-05	0.000619	2777	1816	1134	5893	6352	3366
Solyc07g064740.2.1	-2.10462	5.346828	3.20E-05	0.000339	1143	1066	647	3827	2164	1289
Solyc08g076050.2.1	-2.1025	4.236435	2.97E-05	0.000318	210	917	425	517	2021	1028
Solyc01g091910.2.1	-2.10096	5.152151	7.72E-08	1.70E-06	572	1222	913	1969	3086	1552
Solyc04g006940.2.1	-2.10062	5.913969	2.65E-06	3.84E-05	775	2818	1316	2646	5475	3058
RRNA_11_6	-2.10039	7.110309	1.86E-05	0.00021	3311	3038	3297	5749	9854	8823
Solyc01g097230.2.1	-2.09989	4.695674	1.11E-07	2.34E-06	528	907	518	1292	2342	1192
Solyc08g066970.2.1	-2.09614	4.783943	2.80E-07	5.36E-06	640	921	485	1845	1993	1169
Solyc11g008420.1.1	-2.09611	3.489488	3.15E-06	4.46E-05	162	414	294	652	774	579
Solyc05g011920.2.1	-2.09557	5.465936	4.10E-07	7.51E-06	820	1916	756	2064	4057	2110
Solyc04g016250.2.1	-2.09542	1.037569	0.001055	0.006623	32	85	43	143	151	78
Solyc02g087260.1.1	-2.09491	4.768231	1.31E-07	2.73E-06	443	1094	599	1761	2262	1040
Solyc12g094660.1.1	-2.094	7.482628	2.31E-06	3.41E-05	3000	7442	3670	10421	14839	7771
RRNA_SL2.40sc06748_1	-2.09346	6.214612	1.13E-05	0.000135	1623	1668	1963	2752	6219	4473
Solyc05g053320.1.1	-2.09324	3.50482	2.39E-06	3.51E-05	195	356	300	723	860	487
Solyc11g006500.1.1	-2.09223	5.972583	1.07E-06	1.74E-05	1453	2069	1143	3823	5103	2652
Solyc08g066210.2.1	-2.08934	6.870635	2.08E-06	3.12E-05	2370	4635	2066	6337	10382	5076
Solyc02g024050.2.1	-2.08798	7.202447	3.50E-05	0.000367	3425	4305	3034	13499	7942	4852
Solyc11g008390.1.1	-2.08314	7.123961	1.72E-06	2.65E-05	2432	5581	2947	8474	11112	6016
Solyc11g068610.1.1	-2.08211	5.805366	1.22E-06	1.96E-05	1349	1694	1066	3399	4369	2457
Solyc10g085350.1.1	-2.0816	1.554059	0.000551	0.003827	41	86	93	177	214	136
Solyc03g007430.2.1	-2.07765	6.235024	3.38E-06	4.74E-05	1852	2656	1167	4389	6114	3326
Solyc02g078180.2.1	-2.07717	5.605913	2.28E-07	4.48E-06	838	2015	1010	2612	4083	2258
Solyc05g006220.2.1	-2.07565	5.069325	3.14E-07	5.91E-06	636	939	913	2318	2628	1241
Solyc06g063190.2.1	-2.07317	5.579397	1.19E-05	0.000142	1048	2129	624	1646	4657	2586
Solyc04g009640.2.1	-2.07175	2.089029	0.00017	0.001415	59	169	109	245	367	174
Solyc07g055080.2.1	-2.07147	6.160386	0.001352	0.008195	2404	1483	987	8144	2540	1801
Solyc09g082570.2.1	-2.06824	4.112389	6.86E-06	8.81E-05	410	741	208	974	1200	904
Solyc08g067290.2.1	-2.06663	5.819381	0.000192	0.001572	1629	1480	926	5783	2589	1605
Solyc02g088620.2.1	-2.06549	7.073693	1.24E-05	0.000147	3666	4035	2168	8618	10678	5443
Solyc06g075610.1.1	-2.06495	7.167868	6.84E-06	8.79E-05	3256	5621	2295	9613	10844	5793
Solyc02g083820.1.1	-2.06362	5.423611	1.91E-07	3.82E-06	862	1659	834	2301	3853	1832
Solyc07g008620.1.1	-2.06347	5.987681	2.22E-06	3.30E-05	1412	2567	991	3629	4917	2976
Solyc07g006890.1.1	-2.0628	8.127283	0.000217	0.00174	9287	8241	2536	18633	20552	11614
Solyc03g006080.2.1	-2.06086	5.688685	4.88E-06	6.57E-05	1391	1704	753	3366	3441	2398
Solyc03g005260.2.1	-2.06059	8.082814	4.30E-05	0.000438	7967	6284	4856	18131	16108	13407
Solyc12g008950.1.1	-2.06021	3.713522	0.000423	0.003066	500	241	133	738	1098	574
Solyc02g087460.1.1	-2.05488	6.752378	1.95E-06	2.96E-05	1983	4150	2371	7081	8076	4457
Solyc01g067740.2.1	-2.05469	10.05576	5.04E-05	0.000503	28827	37501	14175	77049	81094	37421
Solyc07g065340.1.1	-2.05366	6.452946	2.13E-05	0.000236	2123	3139	1445	2842	8208	5038
Solyc08g014130.2.1	-2.05278	6.628971	2.81E-05	0.000302	2841	2521	1741	7111	5733	4562
Solyc02g089060.2.1	-2.0509	4.734693	9.02E-07	1.51E-05	616	984	458	1142	2430	1338
Solyc02g090970.1.1	-2.04975	6.038349	1.22E-05	0.000145	754	3625	1323	3236	5499	3257
Solyc09g090710.2.1	-2.04963	4.543811	1.26E-06	2.02E-05	306	948	636	1019	2185	1124
NCRNA_2_182	-2.04921	2.561168	0.00035	0.002614	99	143	195	184	580	326
Solyc06g073020.2.1	-2.04833	4.870954	6.08E-07	1.07E-05	726	906	558	1376	2470	1487
Solyc08g066980.2.1	-2.04635	5.218917	3.28E-07	6.15E-06	891	1177	739	2134	2965	1684
Solyc04g054260.2.1	-2.04523	6.78517	2.80E-06	4.02E-05	2533	3426	2364	6475	8513	5020
Solyc12g008770.1.1	-2.04354	6.361688	6.64E-06	8.58E-05	2203	2387	1485	5211	6063	3592
Solyc02g071820.2.1	-2.03864	7.253985	0.0001	0.000905	1268	8955	3401	8481	10642	7981
Solyc03g083140.2.1	-2.03843	4.43557	1.45E-06	2.29E-05	552	745	354	1132	1924	945
Solyc02g092230.2.1	-2.03833	4.834257	2.78E-06	4.00E-05	641	1014	554	2360	1891	914
Solyc01g098730.2.1	-2.03806	6.967057	3.67E-06	5.11E-05	2258	5326	2562	6383	8832	6939
Solyc03g063690.1.1	-2.03701	3.070976	6.02E-05	0.000587	101	393	210	389	593	496
Solyc12g099810.1.1	-2.03687	5.223555	0.000106	0.000952	1306	752	518	2888	2344	1439
Solyc08g082040.2.1	-2.03551	4.849467	6.59E-07	1.14E-05	567	1186	560	1198	2853	1344
Solyc02g063520.2.1	-2.03424	5.217097	2.44E-06	3.57E-05	725	1864	520	1936	2896	1871
Solyc06g051030.2.1	-2.03415	6.630067	3.48E-05	0.000366	3006	2930	1353	5998	7912	4184
Solyc03g033370.2.1	-2.03344	2.869178	3.42E-05	0.00036	111	265	204	367	570	375
Solyc01g079810.2.1	-2.03065	5.852095	1.54E-06	2.40E-05	1352	1984	1118	3449	4287	2664
Solyc03g117640.1.1	-2.02966	4.389108	1.14E-06	1.86E-05	328	995	430	1039	2023	866

Solyc08g080940.2.1	-2.02735	8.297499	4.18E-05	0.000428	9238	8830	5072	22003	21854	12752
Solyc01g079610.2.1	-2.02653	7.662362	2.18E-05	0.00024	3449	6002	6168	15511	11447	8630
Solyc02g091100.2.1	-2.02482	8.730792	1.96E-05	0.00022	11702	12631	7398	24074	34914	18665
Solyc02g085830.2.1	-2.02394	5.3957	3.61E-06	5.03E-05	1078	1502	675	1661	3898	2182
Solyc05g055470.2.1	-2.02038	7.194323	5.02E-06	6.74E-05	2929	5517	3204	9962	11178	5562
Solyc10g083610.1.1	-2.01958	7.12998	3.46E-06	4.85E-05	2871	5253	2997	8591	11186	5793
Solyc05g006980.2.1	-2.0189	3.771089	0.000231	0.001842	259	436	365	280	1893	550
Solyc10g054440.1.1	-2.01863	11.04528	1.86E-05	0.00021	50766	72054	40673	151628	135906	88494
Solyc01g109680.2.1	-2.01767	4.484316	0.000401	0.002929	747	561	300	2231	932	718
Solyc03g112870.2.1	-2.01713	8.243298	7.79E-06	9.84E-05	5612	11446	7206	12570	26576	16047
Solyc04g007650.2.1	-2.01388	3.257073	0.000205	0.00166	267	337	131	823	531	333
Solyc06g072740.1.1	-2.01225	1.915017	0.000964	0.006142	84	150	66	317	277	96
Solyc05g050120.2.1	-2.01203	12.14567	1.93E-05	0.000217	109401	154789	87513	304806	273251	216201
Solyc05g006860.2.1	-2.01203	3.006969	0.00012	0.001055	169	263	192	347	986	247
Solyc12g099100.1.1	-2.0111	7.317909	4.98E-06	6.70E-05	2956	6342	3629	7484	13274	8141
Solyc01g060240.2.1	-2.00786	4.576066	0.000249	0.00196	906	380	332	1308	2062	1013
Solyc01g095070.2.1	-2.00694	7.146603	3.53E-06	4.94E-05	2724	4866	3611	8305	10581	6565
Solyc09g075120.2.1	-2.00651	8.433369	6.47E-06	8.40E-05	5678	13310	9103	21597	26013	14819
Solyc01g058500.2.1	-2.00538	4.917323	1.02E-05	0.000125	747	665	803	1127	2443	1813
Solyc06g074550.2.1	-2.00455	2.286671	0.000167	0.001395	87	199	111	287	318	249
Solyc10g011880.1.1	-2.00436	4.089067	0.000343	0.002576	571	336	291	1585	763	600
Solyc09g075110.1.1	-2.00425	4.301129	2.03E-06	3.05E-05	279	898	485	1348	1405	792
Solyc12g005080.1.1	-2.00277	7.924888	5.86E-06	7.72E-05	5082	9260	5144	12520	20334	11382
Solyc05g006780.2.1	-2.00171	5.642897	9.46E-07	1.57E-05	939	2066	1086	3404	3697	1941
Solyc10g007290.2.1	-1.99883	4.877171	2.26E-06	3.35E-05	621	1001	693	2267	2253	877
Solyc03g115410.2.1	-1.9964	4.749524	1.71E-06	2.64E-05	700	885	494	1794	2019	1055
Solyc06g053700.1.1	-1.99518	3.233948	3.32E-05	0.00035	243	329	162	577	678	424
Solyc03g116030.2.1	-1.99494	5.731088	1.12E-05	0.000134	1552	1566	885	3395	3965	2218
Solyc03g093890.2.1	-1.99467	3.289778	6.99E-05	0.000666	232	409	151	375	757	594
Solyc06g009020.2.1	-1.9942	9.20673	1.08E-05	0.00013	13606	20786	12264	38751	41676	25309
Solyc06g066050.2.1	-1.99396	5.223279	4.64E-06	6.28E-05	946	1184	751	2937	2123	1496
Solyc08g078510.2.1	-1.99363	5.645542	1.53E-05	0.000177	1021	2684	613	2679	4028	2345
Solyc09g015530.2.1	-1.99121	5.575731	5.81E-07	1.02E-05	861	1864	1169	2445	4270	2069
Solyc01g007020.2.1	-1.98892	5.319579	9.56E-05	0.000869	700	2544	381	1876	3907	1678
RRNA_11_4	-1.98865	6.880824	4.74E-05	0.000476	2941	2681	3122	4655	8553	7396
Solyc02g065540.1.1	-1.98857	4.586604	6.20E-06	8.11E-05	556	908	459	1975	1472	827
Solyc02g062460.2.1	-1.98812	3.646852	1.82E-05	0.000206	157	596	324	625	1246	482
Solyc09g015820.2.1	-1.9873	6.560161	2.87E-06	4.12E-05	1733	3624	2334	5680	6989	4248
Solyc10g011740.2.1	-1.98715	5.594069	1.63E-06	2.52E-05	730	2301	1104	3007	3958	1872
Solyc10g054320.1.1	-1.98688	5.823956	4.62E-06	6.27E-05	1337	2017	1135	4007	4235	2039
Solyc02g078400.2.1	-1.98651	8.196619	0.000676	0.004562	5364	13383	5953	4431	29021	19763
Solyc02g088520.2.1	-1.98644	3.711799	7.51E-06	9.54E-05	281	478	289	975	908	471
Solyc09g098310.2.1	-1.98639	7.08776	4.31E-06	5.89E-05	2374	5559	3311	7593	11014	6064
Solyc01g059980.2.1	-1.98606	4.394663	2.53E-06	3.69E-05	332	679	669	1351	1295	1027
Solyc08g082310.2.1	-1.98445	4.187569	1.55E-06	2.42E-05	285	711	505	962	1249	985
Solyc07g032230.2.1	-1.98344	7.067931	4.82E-06	6.51E-05	2602	5541	2922	7900	11158	5469
Solyc11g072550.1.1	-1.9834	6.639204	4.37E-06	5.95E-05	1774	3965	2464	6581	7531	3921
Solyc06g005500.2.1	-1.98301	8.160285	8.93E-06	0.000111	6477	10496	5926	18800	20696	11860
Solyc03g070380.2.1	-1.98147	1.327994	0.001453	0.008719	67	81	45	151	190	107
Solyc07g049180.2.1	-1.98081	7.881589	1.79E-05	0.000203	5927	8806	4079	14781	18654	9428
Solyc07g063620.2.1	-1.97909	3.260035	7.52E-05	0.000709	269	222	217	582	811	363
Solyc03g007220.2.1	-1.97898	4.750677	4.25E-05	0.000434	638	1193	394	2394	1293	981
Solyc08g063010.2.1	-1.97855	2.806175	0.000114	0.001016	137	242	180	548	413	268
Solyc02g067530.2.1	-1.97747	4.75727	7.03E-07	1.21E-05	437	1276	600	1407	2311	1212
Solyc03g097830.2.1	-1.97705	5.612812	1.32E-06	2.11E-05	746	2148	1247	2517	4314	2142
Solyc10g009180.2.1	-1.97653	6.96127	9.88E-06	0.000121	3003	4806	2238	8097	9276	5072
Solyc06g009110.2.1	-1.97603	5.491275	5.60E-07	9.92E-06	781	1910	1071	2440	3642	2052
Solyc03g114890.2.1	-1.97554	3.591398	1.35E-05	0.000159	187	588	264	534	942	665
Solyc06g082080.2.1	-1.97461	4.39255	1.11E-05	0.000133	513	957	275	1189	1901	799
Solyc07g008280.2.1	-1.97414	4.764275	1.14E-06	1.85E-05	633	897	613	1217	2039	1540
Solyc08g005200.2.1	-1.9738	4.814493	5.72E-07	1.01E-05	598	901	724	1853	1998	1177
Solyc12g013690.1.1	-1.97037	7.278191	0.000125	0.001096	5083	4600	2046	9959	10714	6890
Solyc05g051200.1.1	-1.96965	6.586277	0.001026	0.006477	3600	2519	916	8336	4754	3581
Solyc10g080900.1.1	-1.96897	4.756712	2.51E-05	0.000272	728	798	554	1271	3273	759
Solyc02g089290.2.1	-1.96753	5.775341	3.09E-06	4.40E-05	1109	2195	1211	3835	3630	2252
Solyc04g076830.2.1	-1.9654	7.505347	1.10E-05	0.000132	4399	6015	3952	9760	15507	7878
Solyc10g074930.1.1	-1.96525	7.149807	2.14E-05	0.000236	3611	5163	2581	6354	14132	6030
RRNA_SL2.40sc06396_1	-1.96517	7.750314	6.90E-05	0.000658	5498	4960	5713	8896	15332	13270
Solyc07g064800.2.1	-1.96204	7.475467	1.74E-05	0.000199	2619	8081	4595	7914	15356	8949
Solyc10g012080.2.1	-1.96139	6.783551	1.31E-05	0.000154	2529	4449	2074	7768	8053	4041
Solyc09g014280.1.1	-1.96106	8.150857	9.90E-05	0.000897	6299	10300	6387	25642	12627	10580
Solyc04g071030.1.1	-1.95577	7.197581	4.92E-05	0.000492	3198	7498	2010	8551	12965	5542
Solyc06g071060.1.1	-1.95319	5.09709	3.54E-05	0.000371	1142	969	477	2196	2309	1536
Solyc03g007900.2.1	-1.95281	5.555341	5.06E-06	6.78E-05	1289	1700	768	2546	3710	2184
RRNA_6_1	-1.95259	7.996325	7.81E-05	0.000731	6660	5960	6682	10884	17006	16084
Solyc07g043250.1.1	-1.95242	7.014435	9.51E-06	0.000117	2832	5349	2568	8405	8613	5784
Solyc10g049630.1.1	-1.95237	5.315171	2.76E-06	3.97E-05	919	1817	623	2462	3096	1625
Solyc03g118390.2.1	-1.95185	7.003614	6.88E-06	8.82E-05	2900	4735	2807	6536	11303	5617
Solyc07g005440.1.1	-1.95003	9.127692	2.08E-05	0.000231	14235	20607	10330	32739	43310	24468
Solyc04g078640.1.1	-1.94897	5.609613	6.74E-05	0.000645	1636	1474	622	3193	3461	2043
Solyc03g083470.2.1	-1.94877	5.5994	0.000867	0.005632	1689	1846	294	3544	3083	1929
Solyc09g009390.2.1	-1.94842	9.265759	3.54E-05	0.000371	12764	21746	15596	48636	35974	23397
Solyc02g067470.2.1	-1.94724	3.931538	6.81E-06	8.76E-05	248	678	381	688	1548	618
Solyc08g080900.2.1	-1.94421	7.569122	4.42E-05	0.000448	5622	5966	3228	10782	15117	8314

Solyc04g010310.2.1	-1.94248	7.466567	1.36E-05	0.000159	3714	7415	3722	11852	12271	7310
Solyc11g005380.1.1	-1.94182	8.143361	1.60E-05	0.000184	7135	9991	5660	15244	24492	11879
Solyc06g069780.2.1	-1.94117	5.639814	9.23E-06	0.000114	1453	1514	915	2618	3638	2542
Solyc07g005130.2.1	-1.9402	5.933837	1.17E-05	0.000139	1739	1883	1160	3422	4743	2780
Solyc01g103030.2.1	-1.93959	5.448347	7.72E-07	1.31E-05	820	1616	1179	2208	3707	1992
Solyc07g008940.2.1	-1.93919	6.625446	6.58E-06	8.52E-05	1670	4383	2434	5589	8266	4090
Solyc04g007580.1.1	-1.93705	6.191287	0.000186	0.00153	1593	4206	752	2945	7016	3474
Solyc06g035610.2.1	-1.93694	2.21215	0.00077	0.005094	134	176	63	268	370	194
Solyc05g006640.2.1	-1.93682	6.7004	7.35E-06	9.37E-05	2313	3968	2301	5687	9255	4149
Solyc06g073840.2.1	-1.93525	7.366881	4.10E-05	0.000421	4743	5358	2934	9932	13899	6302
Solyc11g012780.1.1	-1.93331	4.9918	7.61E-07	1.30E-05	762	1150	681	1949	2452	1314
Solyc09g075750.1.1	-1.93249	4.885214	1.91E-05	0.000215	882	1044	433	2076	2179	1060
Solyc02g082350.2.1	-1.9295	6.359294	3.42E-05	0.00036	2189	2939	1514	6339	4932	3130
Solyc01g005920.2.1	-1.92904	4.786472	3.55E-05	0.000371	865	718	521	2081	1991	897
Solyc08g060970.2.1	-1.92746	5.753399	7.80E-06	9.85E-05	738	2560	1465	3100	4292	2313
Solyc01g010020.2.1	-1.927	4.150376	5.09E-06	6.81E-05	397	821	304	891	1374	887
Solyc01g099520.2.1	-1.92697	6.184995	9.36E-06	0.000115	1024	3390	1983	3861	5966	3278
Solyc06g073190.2.1	-1.92673	7.85468	2.54E-05	0.000275	5533	8379	5030	15700	13855	10616
Solyc04g058110.2.1	-1.92293	4.514052	3.19E-05	0.000339	266	1281	555	863	2111	1173
Solyc11g008370.1.1	-1.91957	3.794382	1.01E-05	0.000123	197	618	396	838	1106	556
Solyc04g007870.2.1	-1.91837	6.575414	7.26E-06	9.27E-05	2225	3513	2133	5468	7726	4004
Solyc08g023470.2.1	-1.91777	6.772711	7.83E-06	9.87E-05	2489	4220	2403	6192	8822	4674
Solyc06g074940.2.1	-1.91755	4.913737	0.000359	0.002665	263	1745	814	848	3755	1220
Solyc03g120140.2.1	-1.91748	5.359327	8.72E-07	1.46E-05	897	1534	980	2439	3390	1614
Solyc02g090820.2.1	-1.91647	6.690054	0.00011	0.000978	997	4953	3335	4598	8387	5396
Solyc10g080560.1.1	-1.91631	1.59004	0.000871	0.005653	58	115	78	181	220	130
Solyc10g086680.1.1	-1.91598	5.082375	8.66E-05	0.0008	467	714	1501	1894	2985	1319
Solyc08g078090.1.1	-1.91505	5.956181	8.24E-05	0.000767	590	2963	2029	2703	5335	3123
Solyc09g082230.1.1	-1.91493	5.495037	0.000142	0.001219	1568	1497	480	2561	3703	1880
Solyc12g087960.1.1	-1.9149	4.061465	0.000147	0.001255	578	373	296	1231	1048	650
Solyc03g083150.2.1	-1.91477	4.313855	8.33E-06	0.000104	505	751	384	801	1917	930
Solyc06g009270.2.1	-1.91311	6.957115	9.02E-06	0.000112	2532	4996	2994	7968	8375	5496
Solyc07g045160.2.1	-1.91097	6.044904	8.93E-06	0.000111	1262	3356	1252	3675	5236	2917
Solyc07g043560.2.1	-1.90917	7.451986	4.28E-05	0.000437	2604	7146	5376	12604	10662	7272
Solyc07g064610.2.1	-1.90882	7.227597	1.27E-05	0.000151	3372	5267	3728	9211	11653	6046
Solyc09g059430.2.1	-1.90832	6.704573	1.58E-05	0.000182	2289	4603	2070	4627	9400	4911
Solyc09g008840.2.1	-1.9083	2.903532	9.10E-05	0.000834	147	272	205	524	508	289
Solyc04g079300.2.1	-1.90799	7.071744	1.56E-05	0.00018	2132	6248	3491	8241	10459	5447
Solyc11g066970.1.1	-1.90702	2.368655	0.001648	0.009661	46	165	224	260	432	234
Solyc01g091840.2.1	-1.90678	5.527201	2.46E-06	3.60E-05	915	2167	953	2209	3991	2131
Solyc12g017430.1.1	-1.90646	2.312037	0.000414	0.00301	127	172	105	295	314	247
Solyc11g061760.1.1	-1.90541	3.993279	5.51E-06	7.31E-05	398	511	379	1009	1125	679
Solyc02g078820.2.1	-1.90309	5.807429	1.02E-05	0.000125	1404	2120	1130	3879	3823	2202
Solyc01g068450.2.1	-1.90119	4.883922	6.79E-07	1.17E-05	608	1213	700	1664	2359	1270
Solyc02g077470.2.1	-1.89532	5.363339	0.000126	0.001099	1188	1342	788	3830	1884	1350
Solyc05g013750.2.1	-1.89127	8.088897	2.17E-05	0.000239	6662	11041	5369	14714	21450	12374
Solyc09g091840.2.1	-1.89123	8.989041	2.19E-05	0.000241	9623	19430	14254	24153	42131	24574
Solyc12g005640.1.1	-1.89076	5.71629	3.43E-06	4.82E-05	1219	1818	1320	2536	4205	2598
Solyc11g017380.1.1	-1.88986	3.008054	0.000152	0.00129	206	312	155	575	571	284
Solyc03g007040.2.1	-1.88883	5.908848	1.06E-05	0.000128	1605	2062	1261	3300	5206	2408
Solyc12g005140.1.1	-1.88873	5.158372	1.27E-05	0.000151	1011	1330	612	2536	2524	1292
Solyc05g009340.1.1	-1.88848	2.759621	0.000354	0.002635	98	352	166	462	578	200
Solyc06g036430.2.1	-1.88788	2.312915	0.000353	0.002634	115	167	127	251	404	229
Solyc03g025380.2.1	-1.88679	3.774778	0.000278	0.002157	418	486	212	1098	1089	319
Solyc06g005490.2.1	-1.88509	4.869137	1.25E-06	2.01E-05	674	1055	716	1699	1995	1402
Solyc09g089670.2.1	-1.88475	6.737998	1.12E-05	0.000134	2278	3836	2860	6561	7538	4694
Solyc02g071470.2.1	-1.88469	6.656205	6.24E-05	0.000604	2272	5143	1587	7110	6140	4262
Solyc02g080070.2.1	-1.88468	8.562688	8.74E-05	0.000805	11009	14468	5933	18301	32900	17074
Solyc01g096730.2.1	-1.88233	6.920027	5.23E-05	0.000519	3524	3772	2462	5604	11649	5026
Solyc01g098580.2.1	-1.88197	3.156009	0.000473	0.003372	285	297	136	684	480	364
Solyc06g063090.2.1	-1.88082	6.815312	6.50E-05	0.000625	1364	5493	3403	5012	10456	5052
Solyc09g074050.2.1	-1.87994	7.722932	7.21E-05	0.000685	3233	11909	4537	9175	19462	9750
Solyc12g009180.1.1	-1.87912	6.492095	7.65E-06	9.69E-05	1797	3502	2416	5092	6754	4077
Solyc02g080040.2.1	-1.87911	3.142279	0.000594	0.004079	131	546	161	302	1026	347
Solyc06g066370.2.1	-1.87845	9.293667	5.49E-05	0.000541	14671	30104	10598	35014	52827	25516
Solyc02g093050.2.1	-1.87745	7.696446	4.52E-05	0.000457	5471	8321	3776	10091	18648	8946
Solyc06g063180.1.1	-1.8748	6.379908	5.71E-05	0.00056	2395	3412	1233	5140	5865	3637
Solyc09g090250.2.1	-1.87416	5.651651	2.93E-06	4.19E-05	1007	2176	1214	3060	4056	1930
Solyc09g090440.1.1	-1.874	4.471624	3.36E-05	0.000355	695	733	370	1541	1445	894
Solyc05g006560.1.1	-1.87342	5.269748	2.41E-06	3.53E-05	684	1822	945	2532	2671	1586
Solyc01g104690.2.1	-1.87065	7.484575	0.000792	0.005222	6700	4537	2547	13268	8848	8184
Solyc04g039960.2.1	-1.86943	4.056708	0.000106	0.000951	187	857	497	1078	781	908
Solyc12g043120.1.1	-1.8688	8.230146	3.31E-05	0.000349	4862	14444	7839	19256	21832	12102
Solyc11g010450.1.1	-1.86862	3.73594	1.51E-05	0.000175	233	525	398	587	1108	671
Solyc02g072080.1.1	-1.86591	7.138491	0.000158	0.001328	3682	7060	1778	7841	12556	5304
Solyc11g013880.1.1	-1.86407	6.396742	1.13E-05	0.000135	1633	3920	1966	4838	7042	3316
Solyc12g006500.1.1	-1.864	5.593016	5.91E-06	7.77E-05	1019	1794	1310	2075	4843	1997
Solyc08g016610.1.1	-1.86399	1.803184	0.001458	0.008744	46	140	120	188	302	138
Solyc02g063020.1.1	-1.86195	4.491854	6.97E-06	8.93E-05	589	945	401	1137	1820	1043
Solyc06g082290.2.1	-1.86084	5.175353	4.24E-06	5.80E-05	589	1702	971	1639	3483	1505
Solyc06g005170.2.1	-1.85942	7.864177	0.000159	0.001337	5639	11838	3459	17074	15833	8250
Solyc12g005290.1.1	-1.85925	4.368273	1.17E-05	0.000139	376	1014	480	1547	1343	736
Solyc03g098650.2.1	-1.85916	1.695844	0.001309	0.007962	79	129	68	208	207	143
Solyc02g064680.2.1	-1.8589	7.032054	2.68E-05	0.000289	2490	5780	3338	7065	12626	4529

Solyc11g018610.1.1	-1.85875	5.48242	1.05E-05	0.000127	971	1894	1041	3454	2932	1496
Solyc01g007010.2.1	-1.85859	5.004246	7.62E-05	0.000716	707	1942	363	1677	2699	1383
Solyc10g050060.1.1	-1.85834	6.610898	0.000196	0.001603	3072	3207	1699	7867	5654	3454
Solyc09g075480.2.1	-1.85766	3.290082	0.000213	0.001718	279	209	276	492	776	461
Solyc05g013060.2.1	-1.85756	5.434098	8.19E-05	0.000763	1508	1222	693	2017	3972	1865
Solyc01g107820.2.1	-1.85533	9.69436	0.000929	0.00597	30409	28783	8081	52809	60372	33355
Solyc06g009140.2.1	-1.85492	9.960668	3.77E-05	0.000392	21381	44820	22077	48826	88655	42793
Solyc02g090350.2.1	-1.85423	6.68413	0.000246	0.001946	2225	4032	2634	9765	5286	2797
Solyc09g011670.2.1	-1.85376	7.930749	7.22E-05	0.000686	4474	8748	7778	18243	14105	9748
Solyc04g074430.1.1	-1.85323	3.962246	0.000491	0.003472	534	603	160	1067	1295	471
Solyc04g012040.2.1	-1.85301	5.013347	0.001241	0.007613	983	974	662	3583	1033	818
Solyc01g007780.2.1	-1.85246	3.038886	0.000201	0.001636	149	289	266	399	487	494
Solyc09g059510.2.1	-1.85246	6.029545	6.49E-05	0.000625	1883	2542	1108	4599	3696	2884
Solyc02g020890.2.1	-1.85124	4.711583	2.23E-06	3.31E-05	515	1245	598	1541	2016	1093
Solyc11g066320.1.1	-1.84824	4.298057	9.39E-05	0.000856	663	608	318	1375	1338	742
Solyc01g103220.2.1	-1.84724	7.915827	4.37E-05	0.000444	4440	9940	6933	9959	20239	12577
Solyc03g063680.1.1	-1.84308	2.418097	0.000535	0.003735	78	282	137	293	370	261
Solyc09g083100.1.1	-1.84175	3.269425	5.41E-05	0.000535	214	396	232	444	779	477
Solyc12g042480.1.1	-1.84109	7.9662	3.49E-05	0.000366	5007	12071	5605	11737	19316	12783
Solyc12g100010.1.1	-1.84037	5.311161	4.34E-06	5.91E-05	897	1870	792	2578	2801	1598
Solyc09g089680.2.1	-1.84011	4.544001	0.001578	0.009331	906	769	206	2034	1228	756
Solyc05g050800.2.1	-1.83489	6.852728	7.47E-05	0.000705	2842	5391	2085	8491	7347	4309
Solyc05g005260.1.1	-1.83471	8.06457	5.13E-05	0.00051	5379	14248	5206	16074	19835	11282
Solyc06g076100.2.1	-1.83367	6.534784	1.65E-05	0.000189	2018	4090	2151	5723	6916	3749
Solyc06g061150.2.1	-1.83051	6.914955	1.97E-05	0.000221	2780	4653	3053	6696	10127	4834
Solyc11g070110.1.1	-1.83028	4.522013	4.18E-06	5.74E-05	418	1076	596	1238	2012	899
Solyc01g007220.2.1	-1.82907	7.311696	6.05E-05	0.000589	2506	8173	4156	10664	11093	6151
Solyc03g119340.2.1	-1.82821	3.555081	0.000389	0.002861	121	637	364	401	1061	629
Solyc03g007530.2.1	-1.82696	8.289096	8.52E-05	0.000788	4499	14049	10054	13729	28348	14255
Solyc01g111970.2.1	-1.82658	2.242846	0.000842	0.005488	110	138	146	299	349	184
Solyc11g069550.1.1	-1.82646	4.451012	4.65E-06	6.29E-05	407	908	634	1271	1892	792
Solyc09g064780.2.1	-1.82602	6.373247	3.46E-05	0.000363	2280	3400	1524	4696	6607	3436
Solyc01g105230.2.1	-1.82586	6.080162	4.36E-05	0.000443	2008	2277	1379	4268	5276	2516
Solyc12g056210.1.1	-1.8253	6.956897	0.000106	0.000949	3536	4293	2652	9524	7430	4554
Solyc04g014820.2.1	-1.82424	5.411982	1.51E-05	0.000175	1261	1517	820	2698	3214	1632
Solyc01g014230.2.1	-1.82355	5.99868	2.21E-05	0.000243	1551	2370	1600	4533	4283	2375
Solyc12g010570.1.1	-1.82252	6.177526	8.97E-05	0.000822	2209	2962	1075	4181	5483	3094
Solyc12g007110.1.1	-1.82208	7.621195	6.75E-05	0.000645	5675	6817	4134	11861	14814	8081
Solyc12g008970.1.1	-1.82196	6.915266	5.85E-05	0.000572	3480	4382	2404	7688	8736	4813
Solyc08g080520.2.1	-1.82173	5.145008	3.92E-06	5.41E-05	636	1707	910	1677	3227	1493
Solyc04g007660.1.1	-1.82109	4.818028	1.20E-05	0.000143	746	1068	617	2069	1701	1097
Solyc10g084690.1.1	-1.8184	3.191048	0.000542	0.003774	223	365	210	814	547	237
Solyc11g072660.1.1	-1.81456	4.92982	9.38E-06	0.000116	809	1287	587	1437	2892	1222
Solyc11g072830.1.1	-1.81399	2.499145	0.001291	0.007873	166	179	126	440	263	241
Solyc03g121610.2.1	-1.81355	3.160361	0.000601	0.004121	120	360	327	413	459	589
Solyc06g050440.2.1	-1.81124	3.433007	0.000181	0.001493	143	644	265	506	997	449
Solyc04g073960.2.1	-1.80895	2.54265	0.001527	0.009089	187	221	88	207	582	268
Solyc09g092480.1.1	-1.80754	4.455417	0.000244	0.00193	358	1316	457	552	2247	1173
Solyc09g090730.1.1	-1.80434	7.456136	0.000106	0.000952	2473	10053	4498	9016	13557	8217
Solyc06g072310.2.1	-1.80372	2.697806	0.001058	0.006638	186	307	88	457	410	252
Solyc07g066570.2.1	-1.80211	4.615902	4.75E-06	6.42E-05	565	1115	540	1432	1911	992
Solyc01g065530.2.1	-1.8021	5.694588	9.96E-05	0.0009	1110	3057	799	2663	5173	1736
Solyc02g071350.2.1	-1.80142	3.852816	4.76E-05	0.000478	356	662	286	772	1411	476
Solyc01g106310.2.1	-1.79909	3.268233	0.000121	0.001067	183	435	262	619	531	469
Solyc04g078680.2.1	-1.79691	7.254947	3.44E-05	0.000362	3589	6271	3754	9056	11559	6270
Solyc05g005250.2.1	-1.79492	6.238067	2.14E-05	0.000236	1762	3077	1889	4820	5650	2849
Solyc09g047910.2.1	-1.79376	4.36821	5.28E-05	0.000523	660	700	387	1374	1501	757
Solyc01g111620.2.1	-1.79355	5.133688	4.90E-06	6.60E-05	706	1633	891	1537	2691	1860
Solyc09g074970.2.1	-1.79254	2.951908	0.000181	0.001496	177	362	167	370	658	344
Solyc12g038550.1.1	-1.79118	3.582563	9.55E-05	0.000868	337	462	246	844	810	434
Solyc09g007130.2.1	-1.78997	5.919846	0.000467	0.003335	2108	1912	1021	4959	3017	2264
Solyc03g034180.2.1	-1.78869	7.090225	7.50E-05	0.000707	3701	5670	2730	8683	10630	4894
Solyc02g084710.2.1	-1.78828	4.32624	0.000398	0.002914	622	589	462	1849	952	607
Solyc12g055930.1.1	-1.78821	4.570056	6.73E-06	8.68E-05	460	1082	644	1507	1809	881
Solyc01g080220.2.1	-1.78816	7.864953	6.33E-05	0.000612	4668	10845	6011	9923	18818	12024
Solyc01g088530.2.1	-1.78705	3.539921	6.56E-05	0.00063	200	584	311	497	1027	544
Solyc10g081440.1.1	-1.78586	7.982832	7.92E-05	0.000741	4262	10702	8223	16713	16893	10235
Solyc05g014340.2.1	-1.7844	6.140987	3.32E-05	0.00035	1923	2628	1615	4274	5635	2633
Solyc10g084940.1.1	-1.78367	5.250302	2.72E-05	0.000293	1148	1136	914	1958	3565	1396
Solyc10g080810.1.1	-1.78363	5.443527	8.53E-06	0.000106	1141	1665	1024	2651	3511	1588
Solyc09g008260.2.1	-1.7831	4.560692	5.04E-05	0.000502	765	651	535	1451	1683	976
Solyc03g097680.2.1	-1.78299	7.995374	3.90E-05	0.000403	5289	11299	6779	14306	19437	11002
Solyc02g087000.2.1	-1.78261	6.985393	6.70E-05	0.000642	2472	5504	3626	4996	12618	5461
Solyc09g011660.2.1	-1.78149	6.019058	2.74E-05	0.000295	1483	2208	1976	4161	4237	2770
RRNA_3_1	-1.78145	8.766058	0.000211	0.001706	11548	13014	12454	16850	30201	26622
Solyc02g062930.2.1	-1.78131	5.550717	5.43E-06	7.22E-05	967	2035	1278	2403	3817	2057
Solyc05g006520.2.1	-1.78117	8.274089	4.62E-05	0.000466	7515	11556	8256	17447	23101	13537
Solyc04g016180.2.1	-1.77985	7.988998	0.000151	0.001284	7116	10365	5053	18461	16144	9357
Solyc08g066430.1.1	-1.77719	4.174525	7.47E-05	0.000705	295	904	513	613	1353	1112
Solyc01g109050.2.1	-1.77703	3.066139	0.000211	0.001702	167	320	263	534	669	286
Solyc10g074500.1.1	-1.77654	7.574626	5.59E-05	0.00055	4882	7657	4462	11608	13762	7881
Solyc02g083390.2.1	-1.77652	5.875512	0.000101	0.000911	1833	1953	1223	4190	3828	2161
Solyc11g065660.1.1	-1.77594	5.689734	1.47E-05	0.000171	1339	2023	1219	3074	4234	1893
Solyc06g065200.2.1	-1.77529	3.042365	0.000167	0.001394	164	364	228	417	719	334

Solyc12g014080.1.1	-1.77359	5.294747	4.78E-06	6.46E-05	723	1805	1126	2042	3265	1652
Solyc05g007150.2.1	-1.77319	6.320221	5.11E-05	0.000508	1542	2837	2743	5032	4905	3660
Solyc01g006870.2.1	-1.77202	5.983202	3.93E-05	0.000406	1100	2784	1981	2574	5381	3177
Solyc02g064750.2.1	-1.77181	5.763525	8.45E-05	0.000783	1687	2069	987	3581	3736	2123
Solyc09g090560.1.1	-1.77112	3.114928	0.000175	0.00145	214	338	219	507	733	308
Solyc07g043590.2.1	-1.77013	6.831023	6.23E-05	0.000604	2153	5672	2941	4864	10789	4889
Solyc11g006720.1.1	-1.76981	4.157942	6.89E-05	0.000657	533	796	277	1070	1320	728
Solyc01g102500.2.1	-1.76899	6.086908	1.84E-05	0.000208	1394	3078	1820	4003	4989	2861
Solyc01g097280.2.1	-1.76659	4.611893	6.12E-05	0.000595	387	1206	734	881	1699	1537
Solyc12g042460.1.1	-1.76633	2.524692	0.001168	0.007249	188	180	118	376	414	213
Solyc06g071620.2.1	-1.76607	4.365505	8.37E-05	0.000778	263	976	723	822	1609	1128
Solyc11g010780.1.1	-1.76547	4.753831	0.000469	0.003342	1016	658	512	2122	1453	1002
Solyc03g118340.2.1	-1.76482	7.905252	0.000262	0.002051	4655	10461	7044	7623	26620	10147
Solyc08g079700.1.1	-1.76339	6.863344	0.000473	0.003366	2722	7396	1381	6372	8992	5026
Solyc11g044880.1.1	-1.76148	7.074492	4.35E-05	0.000442	3146	5664	3462	8050	10531	5194
Solyc06g083790.2.1	-1.76138	8.507902	5.20E-05	0.000517	7957	16100	9514	19084	28253	16307
Solyc01g081470.2.1	-1.76135	4.498476	5.34E-05	0.000528	283	1135	763	1376	1499	1002
Solyc06g005160.2.1	-1.76096	9.078238	0.000124	0.001088	8081	26657	17016	28232	44136	23034
Solyc06g035620.2.1	-1.76077	4.556426	0.000159	0.001337	764	1046	301	1317	1851	968
Solyc06g051750.2.1	-1.75877	5.338155	3.18E-05	0.000338	1255	1425	833	1850	3739	1680
Solyc01g094040.2.1	-1.75744	6.019685	2.78E-05	0.000298	1562	2668	1655	2822	5488	3108
Solyc05g012890.1.1	-1.75715	5.649412	1.53E-05	0.000177	1286	2242	1071	2981	3794	2015
Solyc07g006280.2.1	-1.75707	8.047918	0.000241	0.001911	6041	14907	4609	14925	24924	8440
Solyc01g067020.2.1	-1.75549	6.492611	8.46E-05	0.000784	1620	5372	1921	4469	7465	3950
Solyc05g013660.2.1	-1.75524	5.869771	6.66E-05	0.000638	1120	3190	1353	2381	5838	2415
Solyc11g051200.1.1	-1.75462	5.216096	4.60E-05	0.000464	880	1021	1294	1811	2290	2113
Solyc10g006310.2.1	-1.75417	4.67295	5.90E-06	7.77E-05	525	1051	762	1477	1826	1111
Solyc04g050140.2.1	-1.75096	6.205291	7.11E-05	0.000676	2091	2902	1598	4996	5094	2734
Solyc02g083590.2.1	-1.74994	8.317128	0.000143	0.001225	4667	15166	10676	18853	24624	12549
Solyc05g056510.2.1	-1.74477	6.581912	3.63E-05	0.000378	2179	4251	2457	5751	6657	4112
Solyc01g006650.1.1	-1.74419	2.816867	0.000961	0.006128	224	224	157	496	389	298
Solyc03g123700.2.1	-1.74409	5.547778	0.000326	0.00247	1743	1213	909	3192	2664	2032
Solyc01g111980.2.1	-1.74387	8.608357	0.000283	0.002188	7024	23443	8431	28554	25063	13773
Solyc11g066770.1.1	-1.74249	5.736991	1.67E-05	0.000191	1261	2329	1337	2868	4313	2206
Solyc04g076960.2.1	-1.74241	4.232833	6.92E-05	0.000659	266	786	713	853	1566	878
Solyc08g066440.2.1	-1.74196	5.342835	1.06E-05	0.000128	849	2085	955	2516	3114	1503
Solyc02g063160.2.1	-1.74077	5.191069	1.21E-05	0.000144	1027	1341	878	2186	2598	1534
Solyc04g077980.1.1	-1.7385	8.72465	0.000284	0.002194	7622	27496	7901	23839	32800	17415
Solyc03g083160.1.1	-1.73846	1.913461	0.001524	0.009073	76	169	106	211	271	165
Solyc06g075370.2.1	-1.73789	5.223552	8.13E-06	0.000102	718	1823	1026	2165	2930	1467
Solyc09g090110.2.1	-1.7354	6.526892	6.73E-05	0.000644	1567	4414	2855	4172	7498	4420
Solyc04g081550.2.1	-1.73165	7.643153	0.000235	0.001868	2792	11131	5981	8722	15871	10054
Solyc06g083750.2.1	-1.73162	4.289931	2.19E-05	0.000241	395	755	645	897	1419	1023
Solyc10g085300.1.1	-1.73136	5.594543	7.99E-05	0.000746	1615	1703	909	2860	3535	1989
Solyc03g095730.2.1	-1.73066	6.466226	3.97E-05	0.00041	2144	3485	2437	4317	6677	4267
Solyc10g007280.2.1	-1.73042	7.248585	0.000247	0.001952	4691	6589	2572	9531	10870	5981
Solyc02g083860.2.1	-1.72999	4.869181	5.36E-05	0.00053	436	1870	684	1530	1994	1448
Solyc01g005560.2.1	-1.7298	9.765368	0.000136	0.001179	24599	30309	22250	46543	69173	36725
Solyc04g049350.2.1	-1.72946	9.87425	0.000104	0.000938	21050	41263	25338	59837	66174	36614
Solyc11g010500.1.1	-1.72624	7.876009	0.000408	0.002974	4291	14217	5110	9464	25575	8425
Solyc12g008500.1.1	-1.72604	7.781251	0.000116	0.001027	5531	10621	4626	11838	18391	8679
Solyc07g026660.2.1	-1.72529	3.771069	0.000438	0.003163	415	341	400	632	718	864
Solyc01g097350.2.1	-1.7242	5.816854	3.87E-05	0.0004	1497	2352	1325	3580	3817	2292
Solyc10g009060.1.1	-1.72398	5.093228	9.02E-06	0.000112	759	1715	799	2022	2677	1294
Solyc05g010260.2.1	-1.72279	6.138695	4.82E-05	0.000483	1269	3081	2317	4243	5230	2787
Solyc03g117020.2.1	-1.72214	7.055824	0.000131	0.001136	2524	4911	4801	8634	8314	5661
Solyc04g077270.2.1	-1.72156	7.408456	0.00021	0.001697	4494	8243	3304	11744	10100	6940
Solyc12g019320.1.1	-1.72049	7.543634	0.000122	0.001072	5203	6826	4724	10950	14963	6965
Solyc08g076530.2.1	-1.72049	4.675886	2.08E-05	0.000231	644	924	738	1717	1541	1069
Solyc05g015470.1.1	-1.71996	5.419547	5.05E-05	0.000503	1397	1515	864	2222	3590	1739
Solyc08g077290.1.1	-1.71904	4.685734	1.28E-05	0.000151	557	1060	775	1093	2145	1250
Solyc04g079120.2.1	-1.71677	5.676779	0.000206	0.001667	1861	1478	1015	2793	4128	2058
Solyc06g060230.2.1	-1.71636	7.958479	0.000101	0.000912	5074	11373	7205	11264	18178	13003
Solyc09g011920.2.1	-1.71611	6.616071	6.08E-05	0.000592	2660	3831	2441	5399	7212	4321
Solyc02g083460.2.1	-1.71424	6.885048	0.000144	0.001228	2859	5840	2601	5155	11928	4430
Solyc04g011520.2.1	-1.71021	7.082991	0.000141	0.001211	4114	5389	2808	7383	9996	6005
Solyc07g017860.2.1	-1.70747	5.256935	7.50E-05	0.000707	848	1733	1046	3122	2011	1310
Solyc04g080170.2.1	-1.70717	5.834651	0.000151	0.001278	1901	2019	1138	3028	4914	2178
Solyc07g064270.2.1	-1.70616	6.988521	0.000139	0.001193	2127	5754	4342	5410	10125	6398
Solyc11g072270.1.1	-1.70568	5.15649	1.14E-05	0.000137	858	1365	1056	2072	2873	1327
Solyc11g044610.1.1	-1.70392	5.003762	0.000216	0.001734	999	823	929	1887	1474	1816
RRNA_SL2.40sc03714_1	-1.70252	5.422889	0.000126	0.001101	1133	1167	1454	1990	2450	2585
Solyc07g053980.2.1	-1.70204	7.133313	7.52E-05	0.000709	3188	6570	3657	7999	11013	5523
Solyc06g061190.1.1	-1.7011	4.950087	1.54E-05	0.000178	780	1434	716	1902	2442	1088
Solyc01g107780.2.1	-1.70006	6.843848	0.000754	0.00501	2894	6652	1835	3439	10305	6294
Solyc06g051730.2.1	-1.69984	5.606286	0.000141	0.001208	1595	1724	1021	3468	3252	1677
Solyc01g108120.2.1	-1.69914	4.793986	8.09E-06	0.000101	617	1264	762	1426	2200	1199
Solyc06g076130.2.1	-1.69777	6.378111	0.00012	0.001056	2126	4328	1610	5178	6151	3122
Solyc07g062500.2.1	-1.69736	7.537769	0.000731	0.004873	3984	8550	5265	4601	19972	9047
Solyc11g021170.1.1	-1.69616	4.398211	0.000275	0.002137	672	532	604	1187	1040	1191
Solyc04g010300.2.1	-1.69599	3.34322	0.000358	0.002659	242	538	210	728	649	358
Solyc05g008510.2.1	-1.69312	5.846157	0.000116	0.001024	1660	2461	1229	3989	3461	2279
Solyc02g086120.2.1	-1.69284	2.327208	0.001552	0.009207	91	217	170	220	462	207
Solyc12g010690.1.1	-1.69164	4.593321	1.31E-05	0.000154	538	1160	631	1235	1964	1015

Solyc11g066760.1.1	-1.69012	5.367167	1.18E-05	0.000141	964	1761	1171	2100	3503	1662
Solyc01g106890.2.1	-1.68886	4.290818	0.000468	0.003341	736	696	299	1195	1130	931
Solyc10g083720.1.1	-1.68701	8.411531	0.000148	0.001255	8807	14332	8790	21817	24247	12748
Solyc04g015970.2.1	-1.68595	8.843785	0.000119	0.001052	10827	19796	12902	26096	36150	17929
Solyc05g053400.1.1	-1.68428	2.845988	0.001529	0.009101	103	383	235	231	653	371
Solyc11g068400.1.1	-1.68289	6.242918	6.58E-05	0.000632	1562	3461	2285	3286	6240	3612
Solyc08g007100.2.1	-1.68247	3.444572	0.000125	0.001093	284	471	270	600	816	459
Solyc04g063280.2.1	-1.68164	7.22084	0.000103	0.000929	3267	7223	4017	7502	12799	5981
Solyc02g068740.2.1	-1.68096	4.324585	3.67E-05	0.000382	365	859	700	1108	1321	949
Solyc07g005860.2.1	-1.67975	5.362079	1.59E-05	0.000182	788	1892	1312	2322	3125	1672
Solyc01g095990.2.1	-1.67902	8.101943	0.000476	0.003386	9240	10294	5354	18491	19238	9704
Solyc04g012160.2.1	-1.67728	7.324379	0.000259	0.002025	2987	9112	4135	11230	10119	5931
Solyc11g008380.1.1	-1.67728	4.167153	3.00E-05	0.00032	406	847	484	1128	1330	655
Solyc02g093750.2.1	-1.67575	5.529154	8.52E-05	0.000788	1467	1873	921	2078	3459	2333
Solyc11g045210.1.1	-1.6747	4.628131	2.59E-05	0.00028	698	1028	586	1444	1663	1087
Solyc07g065650.2.1	-1.67461	7.068842	9.31E-05	0.00085	2999	5668	4115	6469	10254	6324
Solyc10g081850.1.1	-1.67368	6.440555	0.000163	0.001365	2637	3649	1813	5502	5992	3387
Solyc07g006790.2.1	-1.67333	8.339997	0.00012	0.001055	7205	14175	9680	18739	22267	14149
Solyc03g083440.2.1	-1.67304	7.948209	0.000263	0.002057	7544	8661	6201	12208	22329	9373
Solyc12g013620.1.1	-1.673	8.187734	0.000157	0.001322	5481	14654	8748	16880	23406	10767
Solyc02g094030.2.1	-1.67286	6.961293	0.000651	0.004418	1608	7492	3865	4352	10637	6563
Solyc10g005060.2.1	-1.67163	4.447644	0.000211	0.001704	744	543	594	1252	1785	791
Solyc12g096270.1.1	-1.67036	5.197603	2.02E-05	0.000225	1033	1457	925	2031	2586	1634
Solyc03g082870.2.1	-1.66962	5.067836	1.20E-05	0.000142	846	1521	847	1964	2412	1377
Solyc06g061180.1.1	-1.66907	5.443959	3.57E-05	0.000373	1050	2365	911	2496	2937	1943
Solyc02g078540.2.1	-1.666	7.496611	0.000136	0.001174	4286	7893	5123	10017	15098	6649
Solyc05g050970.2.1	-1.664	7.535341	0.000241	0.001911	4321	7857	5540	13185	10529	7344
Solyc12g042360.1.1	-1.66349	5.390422	1.59E-05	0.000183	946	1885	1221	2506	3130	1611
Solyc01g109130.2.1	-1.66137	4.819107	0.00015	0.001272	794	1154	702	2293	1484	948
Solyc02g082510.1.1	-1.66121	7.349649	0.000138	0.001189	3506	7793	4691	8479	14148	6154
Solyc07g065960.1.1	-1.65994	3.53396	0.000372	0.002752	377	499	208	617	810	534
Solyc10g078490.1.1	-1.65753	6.530029	0.00016	0.001343	2702	3446	2403	5873	5729	3930
Solyc08g007190.2.1	-1.65706	4.840308	1.45E-05	0.000168	610	1383	825	1485	2310	1180
Solyc06g082780.2.1	-1.65555	3.782188	0.000464	0.003318	407	692	237	1021	829	476
Solyc01g098910.2.1	-1.65537	6.880261	0.000137	0.001179	2868	5036	3380	7748	8385	4167
Solyc05g008920.2.1	-1.65533	5.590487	4.43E-05	0.000449	901	2521	1424	2834	3883	1713
Solyc01g106420.2.1	-1.65472	5.192076	2.59E-05	0.00028	846	1799	952	2404	2406	1404
Solyc03g044260.2.1	-1.65471	6.833923	0.000151	0.001284	2195	5541	3575	4876	8673	5853
Solyc11g005670.1.1	-1.65448	8.839648	0.001047	0.006586	15672	21290	6483	20190	38330	20993
Solyc08g082610.2.1	-1.64913	9.198749	0.00025	0.001966	11160	33406	15660	31868	47156	23158
Solyc05g007460.2.1	-1.64596	5.88113	0.000554	0.003841	2240	1957	1076	3644	4179	2269
Solyc07g064290.2.1	-1.64546	5.770063	7.54E-05	0.00071	1490	2289	1417	3396	3901	2064
Solyc10g076360.1.1	-1.64458	4.692581	6.14E-05	0.000596	804	1005	601	1540	1579	1188
Solyc12g088370.1.1	-1.64383	5.995372	0.000129	0.001125	1905	2810	1374	3719	4902	2418
Solyc01g103770.2.1	-1.64194	3.051921	0.000589	0.004051	141	440	261	459	615	344
Solyc11g071330.1.1	-1.64044	4.990822	1.68E-05	0.000192	744	1493	884	1928	2168	1300
Solyc03g043640.2.1	-1.63982	3.081299	0.00081	0.005316	152	448	258	353	839	322
Solyc06g053230.2.1	-1.63809	6.179282	0.000338	0.002544	2526	2317	1689	4428	5225	2771
Solyc03g019680.2.1	-1.63757	6.814361	0.000331	0.002504	3588	4392	2536	7491	7797	3988
Solyc01g103470.2.1	-1.63743	3.334011	0.000979	0.006227	332	474	161	562	611	494
Solyc06g065210.2.1	-1.63675	5.431205	0.000583	0.004016	1585	1449	863	3379	2548	1368
Solyc09g075730.2.1	-1.63595	2.643323	0.001605	0.009453	202	267	121	310	476	279
Solyc06g082070.2.1	-1.63452	3.83397	0.000252	0.001983	400	410	483	656	1007	729
Solyc01g110150.2.1	-1.63337	5.273366	0.000279	0.002161	1083	1267	1218	3169	2073	1231
Solyc10g006540.2.1	-1.63235	6.813912	0.000169	0.001412	3138	4793	2862	6943	8243	4159
Solyc01g006150.2.1	-1.63082	6.937513	0.000156	0.001315	3448	5031	3212	6131	9883	5172
Solyc05g008200.2.1	-1.63079	7.921923	0.000196	0.001603	5749	10626	7259	14904	16017	10023
Solyc09g005920.2.1	-1.62997	6.016033	9.39E-05	0.000856	1753	2711	1746	4003	4528	2503
Solyc01g102290.2.1	-1.62955	7.400285	0.001121	0.006997	6420	6371	2728	8687	14396	6483
Solyc06g051720.2.1	-1.62941	5.846827	8.79E-05	0.000808	1156	2510	1990	3459	4069	2283
Solyc06g005260.2.1	-1.62906	6.478014	0.000227	0.001812	2787	3469	2117	5460	5549	3889
Solyc07g054060.2.1	-1.62813	3.633661	0.001565	0.009262	456	337	303	460	1379	431
Solyc04g074940.2.1	-1.62635	4.37955	0.00012	0.001055	557	803	616	1500	1104	832
Solyc11g044470.1.1	-1.62544	4.838708	4.59E-05	0.000463	587	1408	876	1144	2650	1237
Solyc02g089630.2.1	-1.62507	7.741896	0.000778	0.005142	2618	12431	7565	11670	15606	9181
Solyc04g064810.2.1	-1.62469	6.211157	9.39E-05	0.000856	1576	3480	2311	4065	5931	2847
Solyc01g091030.2.1	-1.62424	6.097241	0.000226	0.001809	2279	2454	1603	3393	5025	3189
Solyc02g021440.2.1	-1.62392	8.357464	0.00033	0.002496	9779	14261	7507	20121	24344	11985
Solyc06g024290.1.1	-1.62167	4.299809	0.000481	0.003417	575	529	675	1118	923	1101
Solyc04g007990.1.1	-1.62049	4.706374	0.000838	0.005465	310	918	1318	1622	1674	1085
Solyc11g071600.1.1	-1.62045	3.522514	0.000594	0.004077	228	786	221	531	901	530
Solyc04g080090.2.1	-1.6196	4.617962	2.95E-05	0.000315	588	1159	685	1467	1767	956
Solyc12g044910.1.1	-1.61687	4.740949	3.35E-05	0.000354	638	1125	840	1668	1847	1027
Solyc04g007530.2.1	-1.61664	4.373362	7.93E-05	0.000741	591	901	513	930	1940	796
Solyc10g081570.1.1	-1.61403	5.034257	7.43E-05	0.000703	934	1670	660	1764	2841	1151
Solyc11g039960.1.1	-1.61239	5.146995	0.000491	0.003472	1354	1107	736	2487	2343	1193
Solyc05g054550.2.1	-1.61202	5.05961	3.79E-05	0.000393	957	1273	939	2060	2161	1377
Solyc06g011490.2.1	-1.60545	3.747182	0.001186	0.007333	272	467	550	365	1086	804
Solyc03g123730.2.1	-1.60349	6.756482	0.001536	0.009132	3475	4185	2592	9473	5193	3225
Solyc02g089400.2.1	-1.60322	7.218528	0.001174	0.007269	5620	4996	3030	10114	10073	5144
Solyc12g011330.2.1	-1.60249	6.400309	0.000197	0.00161	2317	3658	2274	5596	5745	3010
Solyc04g008260.2.1	-1.60058	3.391453	0.000861	0.005593	253	438	335	332	1085	451
Solyc01g094550.2.1	-1.59939	6.80367	0.000177	0.001468	2610	4994	3488	6867	7805	4290
Solyc11g011780.1.1	-1.59559	6.11503	0.000879	0.005691	2416	2755	1407	2476	4674	4205

Solyc01g107290.2.1	-1.59554	4.780367	4.21E-05	0.00043	698	1347	716	1708	1732	1144
Solyc03g123870.2.1	-1.59401	7.247537	0.000228	0.00182	3435	7985	4167	8801	11148	5951
Solyc01g109760.2.1	-1.59276	3.965122	0.000201	0.001637	507	635	355	912	1121	616
Solyc09g009220.2.1	-1.59172	5.036208	0.000421	0.003056	478	1783	1190	1033	3194	1532
Solyc08g061160.1.1	-1.5891	5.255652	0.000759	0.005034	1341	980	1118	2032	1806	2213
Solyc03g058340.2.1	-1.5884	5.136327	0.000179	0.001481	1215	1286	810	2278	2158	1418
Solyc06g053810.2.1	-1.58801	5.391322	0.000854	0.00556	597	1517	2035	1469	3409	2228
Solyc01g088690.2.1	-1.588	4.218248	0.000111	0.000987	320	1010	617	972	1407	786
Solyc06g007540.2.1	-1.58723	7.197616	0.000271	0.00211	3418	6824	4514	6235	10950	7467
Solyc10g055410.1.1	-1.58615	5.150337	5.76E-05	0.000565	1010	1562	922	2187	2149	1540
Solyc08g006030.2.1	-1.58569	4.34309	0.000151	0.00128	628	781	535	1290	1536	667
Solyc07g055710.2.1	-1.58534	6.72436	0.000378	0.002785	2872	5905	2122	5684	8547	4021
Solyc08g016210.2.1	-1.58511	6.675641	0.001249	0.007655	2276	6508	2164	2928	8665	5745
Solyc05g007490.2.1	-1.58473	4.813129	0.000103	0.000931	665	1037	1025	1924	1781	1014
Solyc05g009730.1.1	-1.58422	3.361907	0.001573	0.009306	218	385	401	293	895	562
Solyc01g091620.2.1	-1.58409	7.047186	0.000323	0.002454	2614	7125	4041	5559	11716	5696
Solyc04g024340.2.1	-1.58356	4.81494	3.30E-05	0.000349	664	1203	925	1450	2180	1173
Solyc03g098220.2.1	-1.5824	7.888573	0.000956	0.006103	8359	8928	5150	15866	15339	8660
Solyc06g076350.2.1	-1.57441	4.122245	0.000333	0.00252	523	530	577	1173	1148	612
Solyc01g104970.2.1	-1.57344	7.262013	0.00035	0.002614	4544	7006	3699	8700	11428	5998
Solyc02g087950.2.1	-1.57312	4.028921	0.000132	0.001143	466	756	406	990	1158	615
Solyc02g088200.2.1	-1.56827	5.313617	6.59E-05	0.000633	738	2090	1335	1871	2991	1842
Solyc10g078500.1.1	-1.56756	3.876384	0.000402	0.002932	395	453	544	916	882	623
RRNA_SL2.40sc06977_2	-1.56705	5.867015	0.000377	0.00278	1918	2055	1575	2636	3779	3145
Solyc04g072640.2.1	-1.56661	5.826537	0.000297	0.002279	1258	2992	1651	1960	5095	2727
Solyc11g044620.1.1	-1.56657	4.696044	0.000728	0.004862	878	688	810	1481	1210	1413
Solyc01g017490.1.1	-1.56565	3.850182	0.001483	0.008861	479	352	482	795	689	799
Solyc02g072300.2.1	-1.56542	7.612891	0.000309	0.002355	4973	9314	5569	11536	14204	7471
Solyc12g055860.1.1	-1.56375	4.698098	5.06E-05	0.000505	707	1087	774	1469	1842	1060
Solyc11g072530.1.1	-1.56086	5.97344	0.000142	0.001214	1507	3212	1768	3319	5150	2360
Solyc05g054170.2.1	-1.55705	5.293854	9.08E-05	0.000832	1240	1655	963	2132	3061	1509
Solyc05g012900.2.1	-1.55574	3.509924	0.000827	0.005409	402	465	237	539	900	494
Solyc01g008820.2.1	-1.55322	7.053941	0.000382	0.002813	2754	6336	4617	8464	8829	4988
Solyc08g082820.2.1	-1.55097	8.999027	0.001151	0.00715	9432	21261	21291	33644	26790	22500
Solyc09g075920.1.1	-1.55042	5.029491	0.001271	0.007769	959	2170	397	1315	2742	1514
Solyc09g013110.2.1	-1.54985	5.260846	6.87E-05	0.000656	1138	1642	1030	2006	2686	1709
Solyc11g007720.1.1	-1.54862	6.909004	0.000235	0.001868	2926	5688	3689	6243	8753	5204
Solyc04g007520.2.1	-1.54789	5.314274	0.000139	0.001192	646	2381	1303	2071	3052	1628
Solyc01g086640.2.1	-1.5477	6.019601	0.000495	0.003486	2165	3075	1265	3142	5351	2630
Solyc05g052790.2.1	-1.54689	5.325988	5.11E-05	0.000509	1052	1796	1205	2276	2938	1567
Solyc01g006430.2.1	-1.54595	8.722638	0.000461	0.003299	12544	17479	11830	22482	30416	18044
Solyc06g068820.2.1	-1.54565	4.911426	0.000193	0.001581	992	1318	673	1966	1949	1111
Solyc12g006200.1.1	-1.54506	4.281465	8.95E-05	0.000821	426	912	662	1011	1408	842
Solyc09g083320.1.1	-1.54413	4.48896	0.000115	0.00102	426	1218	742	1015	1853	965
Solyc12g056630.1.1	-1.54263	4.212431	0.000177	0.001468	353	1005	612	1174	1222	700
Solyc01g099790.2.1	-1.54104	7.456796	0.000513	0.003598	5380	7395	4676	10479	12184	6831
Solyc03g118810.1.1	-1.53776	6.816294	0.001038	0.006535	3468	6214	2059	7525	7354	3986
Solyc02g069350.2.1	-1.53746	4.119058	9.92E-05	0.000898	441	796	535	838	1367	739
Solyc08g065860.2.1	-1.5373	4.693	6.77E-05	0.000647	707	1223	711	1216	2051	1118
Solyc09g011230.2.1	-1.537	5.205834	0.000107	0.00096	1127	1514	1017	2107	2269	1674
Solyc06g009050.2.1	-1.5361	8.474789	0.000945	0.006049	12237	13304	8955	21570	21438	15384
Solyc02g082920.2.1	-1.5358	11.8165	0.000885	0.005725	79773	189770	110816	132891	337041	156614
Solyc02g086360.2.1	-1.53557	4.45385	0.000374	0.00276	794	904	447	1186	1756	808
Solyc06g065740.2.1	-1.53463	5.172037	0.000195	0.001597	1242	1416	863	2146	2556	1365
Solyc12g014350.1.1	-1.53389	7.805683	0.000485	0.003439	5584	11395	6344	13915	14380	8859
Solyc07g052790.1.1	-1.53332	5.110092	0.000533	0.003727	639	2226	956	1053	3340	1608
Solyc03g121860.1.1	-1.52635	6.635846	0.000292	0.002248	2657	4992	2683	5404	7262	4051
Solyc04g008010.2.1	-1.52549	5.840525	0.000191	0.001567	1317	3029	1715	3380	4029	2205
Solyc01g007980.2.1	-1.52501	5.946396	0.000673	0.004546	2200	2625	1282	3602	3741	2745
Solyc11g011190.1.1	-1.52266	5.730415	0.000206	0.00167	1099	3084	1569	2420	3892	2527
Solyc03g120390.2.1	-1.52253	6.255381	0.000239	0.001895	1709	4156	2281	4058	5616	3159
Solyc09g005910.2.1	-1.52193	7.363708	0.000543	0.003781	4936	7288	4440	9748	11844	6151
Solyc05g013450.2.1	-1.51922	6.06979	0.000227	0.00181	1468	3268	2305	3250	5056	2963
Solyc02g021600.1.1	-1.51905	4.776906	0.000105	0.000945	534	1551	881	1560	1810	1164
Solyc11g022540.1.1	-1.51572	6.751634	0.000347	0.002598	2478	5412	3457	6093	8238	3957
Solyc05g008070.2.1	-1.51549	3.725645	0.000703	0.004716	441	655	259	701	1004	525
Solyc02g088690.2.1	-1.51524	7.270575	0.000652	0.004421	3403	8679	4553	9472	12047	4926
Solyc01g100290.2.1	-1.51435	4.608924	9.52E-05	0.000866	584	1169	790	1452	1732	911
Solyc07g047800.2.1	-1.51401	9.1007	0.000925	0.005954	17404	24564	13662	35254	36428	19931
Solyc02g089730.1.1	-1.51396	3.546908	0.000413	0.003009	253	610	378	655	827	469
Solyc01g067000.2.1	-1.51382	6.806744	0.000426	0.003092	3030	6044	2769	6143	8472	4309
Solyc02g071520.2.1	-1.51378	4.347207	0.000605	0.004143	323	1094	786	721	1665	1031
Solyc01g079410.2.1	-1.5137	3.201242	0.001558	0.009235	196	436	329	649	548	319
Solyc01g094050.1.1	-1.51328	5.254022	0.000158	0.001327	923	1991	1123	2625	2436	1295
Solyc11g011220.1.1	-1.51119	6.360573	0.000293	0.002251	2176	3699	2571	4708	5196	3586
Solyc11g012510.1.1	-1.50915	5.707156	0.000599	0.004106	868	3568	1502	2192	4626	2164
Solyc02g090740.2.1	-1.50826	4.397392	0.00011	0.000979	596	923	630	1142	1531	854
Solyc10g084140.1.1	-1.50714	7.003927	0.000401	0.002929	3446	6402	3586	7266	9547	4835
Solyc08g013900.2.1	-1.50652	6.253909	0.00085	0.005537	2811	3008	1698	4233	5784	2881
Solyc10g047140.1.1	-1.50646	6.783635	0.00054	0.003764	3479	4911	2808	6452	7652	4287
Solyc02g082830.1.1	-1.50534	6.197975	0.000961	0.006127	1172	4094	2771	2743	6503	3309
Solyc01g108200.2.1	-1.50484	5.124797	0.00043	0.003114	1058	1459	1006	2632	1695	1296
Solyc12g009230.1.1	-1.50411	3.846504	0.000258	0.002018	380	674	437	752	921	675
Solyc12g096050.1.1	-1.50321	5.762222	0.000262	0.002051	1564	2357	1616	3272	3664	2091

Solyc10g086420.1.1	-1.50145	7.107652	0.000545	0.003791	3578	7295	3781	6096	12082	5517
Solyc11g066060.1.1	-1.50026	8.985454	0.001148	0.007138	8657	29973	17488	30875	35458	18547
Solyc04g082480.2.1	-1.4993	5.656653	0.000401	0.002929	861	3413	1466	2554	3727	2150
Solyc12g005590.1.1	-1.49915	4.641211	0.000172	0.001428	719	1096	739	1460	2006	807
RRNA_SL2.40sc06977_1	-1.49642	6.58229	0.001215	0.00748	2977	3000	3358	4557	5389	5297
Solyc02g069720.2.1	-1.4957	6.567484	0.000501	0.003529	3027	3920	2622	5057	6799	3949
Solyc01g005680.2.1	-1.49266	4.241023	0.000223	0.001787	548	726	628	940	1298	872
Solyc06g076260.2.1	-1.49217	5.645085	0.000157	0.001323	1427	2173	1535	2476	3735	2149
Solyc09g075510.2.1	-1.49214	4.608745	0.000139	0.001197	660	1331	618	1352	1647	1029
Solyc03g006570.2.1	-1.49089	6.518142	0.00038	0.002801	2432	4534	2691	5025	7125	3373
Solyc06g072030.1.1	-1.48915	5.925337	0.00032	0.002433	1693	2477	2026	3538	3870	2559
Solyc10g084800.1.1	-1.4886	7.496698	0.000645	0.004387	4966	8585	5332	10849	12776	6600
Solyc08g069000.2.1	-1.48859	5.942951	0.001391	0.0084	2242	2120	1645	4285	4278	1808
Solyc01g008550.2.1	-1.48834	8.87965	0.000843	0.005495	14311	19948	13785	28170	30248	19093
Solyc01g098410.2.1	-1.48724	4.135138	0.000449	0.003228	348	1016	569	645	1359	906
Solyc01g105100.2.1	-1.48673	7.111228	0.000508	0.003571	3630	7031	4014	8061	9676	5305
Solyc12g088440.1.1	-1.48643	4.815065	0.000159	0.001338	801	1389	766	1773	1992	957
Solyc05g026050.2.1	-1.48261	5.646333	0.000157	0.001324	1309	2418	1552	2750	3753	1910
Solyc03g117110.2.1	-1.48103	5.291459	0.000343	0.002578	1213	1609	1161	2778	2321	1335
Solyc11g068710.1.1	-1.48082	6.484962	0.000945	0.006049	2464	5677	1771	5040	6305	3551
Solyc10g007010.1.1	-1.48041	3.653631	0.00077	0.005094	271	729	383	466	1009	622
Solyc07g063950.2.1	-1.47935	3.96869	0.000311	0.002371	313	802	575	780	1143	676
Solyc06g082650.2.1	-1.4776	8.400846	0.000673	0.004544	7872	17556	10973	15631	25172	15343
Solyc04g077290.1.1	-1.47473	3.492393	0.00077	0.005099	252	526	414	501	953	456
Solyc04g009800.2.1	-1.47439	6.416977	0.000463	0.00331	2591	3659	2537	4760	5834	3518
Solyc02g069240.1.1	-1.47333	4.084999	0.000461	0.0033	334	971	570	642	1347	834
Solyc03g007470.2.1	-1.47272	6.728486	0.001308	0.007962	3714	4245	2728	7013	6023	4166
Solyc09g075020.2.1	-1.47207	10.5031	0.00126	0.007709	36724	85279	37589	59474	128326	60225
Solyc03g121070.2.1	-1.47191	6.252559	0.000998	0.006327	1220	4041	3131	4433	4840	3195
Solyc05g052210.2.1	-1.47083	5.023749	0.00019	0.001564	763	1999	860	1352	2744	1407
Solyc11g005350.1.1	-1.46944	4.236533	0.000853	0.005553	663	659	544	697	1466	955
Solyc11g065000.1.1	-1.46908	6.603698	0.000557	0.003857	2289	5539	2855	5673	6011	4149
Solyc07g008170.2.1	-1.46873	5.044054	0.000123	0.001081	872	1753	929	1969	2101	1333
Solyc11g040110.1.1	-1.46604	6.357822	0.000546	0.003794	1630	4400	2964	3888	5851	3770
Solyc10g079260.1.1	-1.46572	5.724675	0.000238	0.001891	1296	2704	1692	2467	4114	2264
Solyc07g049280.2.1	-1.46513	4.270934	0.000258	0.002018	414	894	749	994	1419	796
Solyc12g009990.1.1	-1.465	8.316892	0.001549	0.009194	10265	14301	8469	22315	19797	10506
Solyc08g066870.2.1	-1.46123	7.481357	0.001123	0.007003	5848	8425	4397	10010	13754	6363
Solyc11g010390.1.1	-1.45943	3.781902	0.000392	0.002881	329	669	474	744	889	606
Solyc09g075910.1.1	-1.45935	5.053642	0.001252	0.007671	1029	2204	483	1862	2420	1256
Solyc03g119420.2.1	-1.45837	5.712314	0.000247	0.001952	1247	2758	1694	2353	3938	2393
Solyc11g017190.1.1	-1.45673	3.624775	0.000492	0.003476	310	614	399	620	890	527
Solyc06g074840.2.1	-1.45604	6.063497	0.000412	0.003003	2007	3097	1922	3434	5017	2701
Solyc12g011130.1.1	-1.45418	3.862636	0.001232	0.007566	552	512	378	858	976	560
Solyc09g008340.2.1	-1.45355	6.849519	0.000561	0.003879	3247	5487	3501	6126	7926	4906
Solyc04g071510.2.1	-1.45314	3.680559	0.000636	0.004337	275	717	425	545	1051	555
Solyc06g009380.2.1	-1.45276	7.747353	0.000845	0.005509	4847	10541	7823	11965	15026	8543
Solyc08g077350.2.1	-1.4526	6.17543	0.000464	0.003318	1570	3625	2654	3640	5051	3186
Solyc01g087570.2.1	-1.45211	3.955759	0.000438	0.003163	350	767	559	684	1351	606
Solyc03g117950.2.1	-1.45205	9.525016	0.000943	0.006043	19789	38413	21480	35546	59538	29112
Solyc07g053910.2.1	-1.45159	7.127932	0.001123	0.007003	4615	6613	3448	8521	9176	5309
Solyc01g098160.2.1	-1.4494	7.315151	0.000823	0.005389	4356	7747	4918	9625	9973	6374
Solyc12g014600.1.1	-1.44796	4.542815	0.00049	0.00347	723	895	766	1570	1397	834
Solyc02g093360.2.1	-1.44546	5.100236	0.000953	0.006089	546	2284	1157	1181	2940	1636
Solyc07g064440.2.1	-1.44515	6.265635	0.000669	0.004518	1698	4743	2257	3745	6425	2878
Solyc04g072910.2.1	-1.44506	7.37987	0.001565	0.009265	6072	7130	3919	8925	11693	6849
Solyc06g074650.2.1	-1.44299	7.432752	0.000874	0.005667	3962	10102	5256	8740	13333	6823
Solyc12g006180.1.1	-1.44285	5.209912	0.000298	0.002289	1182	1518	1123	2272	2524	1325
Solyc06g050770.2.1	-1.44113	7.113875	0.00071	0.004764	3684	6650	4519	7833	9735	5342
Solyc10g050230.1.1	-1.44044	5.226756	0.000159	0.001336	988	1970	1121	1987	2814	1442
Solyc04g057880.2.1	-1.43915	6.001996	0.000421	0.003056	1555	3582	1959	3225	4928	2547
Solyc09g098130.1.1	-1.43899	3.986381	0.000615	0.004207	298	958	543	680	1264	697
Solyc03g113390.2.1	-1.43857	6.438368	0.000792	0.005223	1851	5441	2590	3928	7206	3491
Solyc02g088680.1.1	-1.43767	3.87998	0.000561	0.003883	368	825	434	820	1193	482
Solyc11g011610.1.1	-1.43751	5.891663	0.000516	0.003617	1776	3109	1537	3364	3831	2475
Solyc07g047720.2.1	-1.43674	5.232432	0.000254	0.001994	774	1962	1412	1684	2994	1600
Solyc08g080080.2.1	-1.43666	4.965713	0.000274	0.002129	907	1344	1030	2017	2076	1065
Solyc03g005300.2.1	-1.43556	5.250311	0.000299	0.002293	1102	1768	1177	2507	2265	1409
Solyc07g066070.2.1	-1.43503	6.246506	0.001258	0.0077	2784	3113	1890	4562	4776	3021
Solyc11g069690.1.1	-1.43463	3.963945	0.000614	0.004199	331	803	581	637	1122	782
Solyc01g100850.2.1	-1.43452	4.454508	0.00093	0.005978	508	1051	814	1689	1179	683
Solyc10g079420.1.1	-1.43439	8.800532	0.001245	0.007633	14483	21024	11691	23319	31204	18755
Solyc12g094550.1.1	-1.43362	5.212934	0.000217	0.001741	1059	1685	1193	1763	3138	1386
Solyc02g069710.2.1	-1.43239	4.717141	0.000223	0.001784	803	1235	757	1392	2026	981
Solyc05g014120.1.1	-1.43199	3.271012	0.001541	0.009159	302	435	279	472	701	414
Solyc01g111090.2.1	-1.43171	6.104226	0.000441	0.003181	1783	3583	2154	3430	5055	2884
Solyc01g100000.2.1	-1.4287	4.712919	0.000334	0.002523	814	979	904	1476	1730	1072
Solyc03g081220.1.1	-1.4277	5.025417	0.00016	0.001342	873	1779	936	1498	2533	1382
Solyc10g079710.1.1	-1.42751	3.327247	0.001464	0.008768	225	587	316	549	769	359
Solyc05g007190.2.1	-1.4271	6.015114	0.000558	0.003863	1899	3187	1879	3774	4613	2337
Solyc04g077920.2.1	-1.42659	7.235863	0.000945	0.006049	4178	7657	4548	6891	12683	5884
Solyc03g096810.2.1	-1.4238	6.752795	0.000711	0.004769	2963	5769	3115	5487	8089	4318
Solyc08g067810.2.1	-1.42164	5.042854	0.000155	0.001308	872	1767	993	1598	2430	1405
RRNA_11_13	-1.42086	4.079962	0.000761	0.005041	517	704	552	606	1424	790

Solyc01g112050.2.1	-1.42048	6.61956	0.000888	0.005738	2674	5408	2796	5800	7303	3322
Solyc01g094060.2.1	-1.42038	6.593562	0.000707	0.004742	2468	5105	3074	5621	6472	3729
Solyc04g082030.1.1	-1.41908	6.849394	0.001578	0.009331	3979	4719	3276	6986	8727	3648
Solyc11g013490.1.1	-1.41742	5.561113	0.000304	0.002324	1387	2329	1391	2181	3763	1933
Solyc12g098820.1.1	-1.4173	6.770655	0.000794	0.005234	3201	5311	3277	5593	8365	4223
Solyc09g005870.1.1	-1.41593	4.650764	0.000571	0.003944	610	1211	917	855	2221	1148
Solyc08g008490.2.1	-1.41586	5.878488	0.000752	0.004999	1399	3629	1683	2426	5016	2456
Solyc07g041010.2.1	-1.41511	6.516009	0.000817	0.005353	2688	4271	2865	5437	5809	3620
Solyc04g009380.1.1	-1.41354	5.518639	0.001532	0.00911	1903	1730	1009	2521	3346	1722
Solyc05g012070.2.1	-1.41249	6.31609	0.000655	0.00444	1886	4197	2762	3680	5948	3489
Solyc07g005200.2.1	-1.41122	7.013125	0.000933	0.00599	3946	6009	3893	7067	9184	5023
Solyc05g055480.2.1	-1.41107	5.941441	0.000858	0.005584	1833	3049	1785	3982	3417	2434
Solyc03g095710.2.1	-1.40641	5.043966	0.000199	0.00162	844	1676	1113	1707	2619	1198
Solyc06g059980.2.1	-1.40467	4.391487	0.000352	0.002626	476	1043	799	1041	1650	806
Solyc01g007900.2.1	-1.40442	4.356804	0.000432	0.003123	484	1280	589	1174	1444	755
Solyc04g007860.2.1	-1.40212	7.040057	0.000954	0.006097	3470	6974	4165	7096	10065	4765
Solyc03g113870.1.1	-1.39975	5.165072	0.000782	0.005164	1346	1632	814	2076	2350	1407
Solyc05g054770.2.1	-1.39916	5.322541	0.000612	0.004189	680	2418	1545	1827	3111	1684
Solyc09g061310.2.1	-1.39893	6.600833	0.000934	0.005994	2330	5199	3335	5782	6570	3553
Solyc06g083440.2.1	-1.39641	4.311526	0.000428	0.003104	511	991	686	1205	1254	757
Solyc07g065740.2.1	-1.39529	4.957887	0.000248	0.001959	916	1588	910	1597	2421	1156
Solyc09g074860.2.1	-1.395	5.895616	0.000575	0.003969	1580	2892	2058	2748	4231	2706
Solyc11g007770.1.1	-1.39457	6.602878	0.001431	0.008605	2073	6056	3142	3892	6949	4880
Solyc03g117030.1.1	-1.39434	5.495782	0.000303	0.002317	1148	2490	1432	2156	3314	1928
Solyc01g094860.2.1	-1.39198	5.356353	0.000399	0.002921	1293	2195	1036	2211	3026	1531
Solyc09g005080.1.1	-1.38967	4.681238	0.00078	0.005154	789	1540	571	1255	2228	869
Solyc04g081410.2.1	-1.38883	5.695012	0.000418	0.003036	1426	2468	1774	2764	3554	2117
Solyc08g062500.2.1	-1.38849	6.23135	0.001638	0.009608	2618	3394	2012	4897	4763	2574
Solyc04g064820.2.1	-1.38844	4.67908	0.000667	0.00451	675	1339	838	1810	1519	822
Solyc10g079000.1.1	-1.38786	5.596096	0.000348	0.002607	1353	2480	1520	2522	3269	2052
Solyc06g051410.2.1	-1.38662	4.850051	0.001429	0.008597	949	1113	964	874	2222	1576
Solyc10g075040.1.1	-1.38388	5.27842	0.000306	0.00234	1022	2146	1206	1939	3193	1381
Solyc09g008310.2.1	-1.38362	5.354562	0.000814	0.005337	1534	1603	1127	2295	3017	1453
Solyc06g034110.2.1	-1.38344	5.052953	0.000262	0.002049	942	1578	1112	1938	2262	1224
Solyc07g005600.2.1	-1.38108	7.98584	0.001319	0.008016	6363	13395	8712	12496	19799	9772
Solyc03g121590.2.1	-1.38092	8.520251	0.001396	0.008422	9620	19863	11827	19438	27441	13777
Solyc06g060100.2.1	-1.37929	4.156272	0.000605	0.004147	473	831	651	1096	1129	659
Solyc01g107740.2.1	-1.37848	4.615962	0.001212	0.007465	901	1181	563	1541	1695	797
Solyc05g009020.2.1	-1.37543	4.305059	0.000419	0.003047	522	1065	637	983	1518	764
Solyc04g007710.2.1	-1.37417	7.626808	0.001466	0.008775	5757	10132	6072	10456	15062	7222
Solyc07g040690.2.1	-1.37232	6.115475	0.000805	0.005289	1864	3749	2203	3717	5159	2560
Solyc05g008830.2.1	-1.37019	7.011305	0.001375	0.008316	4183	6088	3795	6874	8892	5203
Solyc05g041870.1.1	-1.3701	4.502059	0.000658	0.004457	574	1225	764	1492	1274	846
Solyc03g097970.2.1	-1.36889	6.352242	0.001083	0.006777	1784	4740	2916	4468	5595	3219
Solyc11g065070.1.1	-1.36836	6.654453	0.00117	0.007253	2541	5564	3355	5063	8107	3633
Solyc02g065050.1.1	-1.36464	4.592453	0.000885	0.005721	611	1701	567	1233	1594	1050
Solyc01g007770.2.1	-1.36449	4.604026	0.000594	0.004079	556	1519	771	1031	2059	969
Solyc06g065720.1.1	-1.36413	5.252636	0.000415	0.003022	929	2059	1346	1991	3078	1302
Solyc04g014270.2.1	-1.36307	6.490013	0.000987	0.006269	2346	4819	3012	4783	6023	3714
Solyc08g006940.2.1	-1.36193	5.243346	0.000455	0.003267	825	2362	1261	1833	2796	1562
Solyc05g055760.2.1	-1.36149	5.416464	0.001549	0.009194	756	2194	1938	2363	2193	2075
Solyc01g099810.2.1	-1.36108	5.814573	0.001193	0.007371	1148	3248	2141	2414	4647	2288
Solyc11g069780.1.1	-1.35991	5.019143	0.000313	0.002387	775	1820	1126	1479	2384	1414
Solyc05g009160.2.1	-1.35683	5.275844	0.000421	0.003055	912	2222	1339	1842	3161	1445
Solyc01g088710.2.1	-1.35589	4.694184	0.000399	0.002917	638	1432	893	1403	1904	944
Solyc05g056100.2.1	-1.35348	5.991137	0.000902	0.005814	1598	3812	1982	3353	4462	2523
Solyc09g010850.2.1	-1.35343	6.284524	0.001562	0.009251	2546	4254	1968	4468	5147	2986
Solyc01g068390.2.1	-1.34928	6.566851	0.001228	0.007544	2535	5251	3060	5127	6890	3508
Solyc09g064470.2.1	-1.34914	5.518006	0.000967	0.006159	1079	2610	1613	3089	2681	1572
Solyc10g055650.1.1	-1.34895	5.872635	0.001169	0.007251	1819	2667	1947	3590	3624	2190
Solyc10g079080.1.1	-1.34654	6.482655	0.001712	0.009986	2905	4203	2740	5521	5843	3125
Solyc06g082490.2.1	-1.34646	5.731262	0.000799	0.005259	1330	2903	1858	3043	3740	1892
Solyc10g007390.2.1	-1.34629	8.075251	0.001668	0.009762	6850	14619	9404	13379	20328	10530
Solyc05g055070.2.1	-1.3441	6.246713	0.001321	0.008027	2491	3422	2399	3949	4943	3261
Solyc01g112040.2.1	-1.34164	4.973701	0.000449	0.003228	890	1638	1028	1644	2509	1069
Solyc07g007050.1.1	-1.34154	3.748723	0.001275	0.007788	350	793	417	566	1029	593
Solyc04g051820.2.1	-1.33984	5.13666	0.000439	0.003164	988	2042	1032	1771	2703	1314
Solyc09g008290.2.1	-1.33719	4.045438	0.000912	0.005879	484	839	536	953	947	701
Solyc07g052640.2.1	-1.33624	4.793746	0.001029	0.006493	945	1407	742	1804	1583	1018
Solyc02g078320.1.1	-1.33564	4.873874	0.00066	0.00447	680	1635	1086	1833	1809	1058
Solyc05g046300.2.1	-1.33483	5.315315	0.00057	0.003934	1258	2080	1153	1874	3270	1467
Solyc06g068960.1.1	-1.33403	4.556999	0.00162	0.009519	644	1536	609	831	1616	1275
Solyc01g057460.2.1	-1.33129	5.167216	0.000532	0.003717	1041	1879	1162	2127	2406	1281
Solyc01g068000.2.1	-1.32994	5.300207	0.000549	0.003815	1279	2029	1127	2065	2748	1558
Solyc01g096460.2.1	-1.32963	4.246218	0.001413	0.008505	645	908	550	1223	1134	672
Solyc08g067480.2.1	-1.32923	5.08745	0.000543	0.003781	838	1856	1235	1364	2625	1537
Solyc08g066680.2.1	-1.32793	5.767898	0.001376	0.008323	1163	3335	1978	2999	3318	2316
Solyc03g097020.2.1	-1.32458	5.760224	0.001602	0.00944	1147	3526	1856	2985	4107	1831
Solyc12g042570.1.1	-1.32378	4.056244	0.001179	0.007298	467	1064	437	860	1052	726
Solyc05g053230.2.1	-1.32367	6.519731	0.001612	0.00949	2687	4948	2876	3932	7167	3889
Solyc01g100660.2.1	-1.32166	6.185751	0.001407	0.008482	1915	3831	2617	4062	4278	3128
Solyc07g051940.2.1	-1.32116	4.884142	0.001038	0.006535	977	1781	668	1540	1916	1250
Solyc04g024710.2.1	-1.31841	4.818824	0.000667	0.00451	844	1380	959	1725	1816	995
Solyc04g009310.2.1	-1.31771	4.892189	0.00059	0.004056	937	1323	1032	1569	2116	1128

Solyc03g006000.2.1	-1.31689	5.047342	0.000684	0.004606	1117	1656	943	1899	2158	1247
Solyc12g005570.1.1	-1.31503	5.439217	0.000827	0.005407	984	2277	1772	2163	2675	1994
Solyc04g077600.2.1	-1.31362	5.095482	0.000513	0.003597	967	1871	1112	1950	2231	1298
Solyc02g031830.1.1	-1.31121	5.539416	0.001582	0.009339	1708	2108	1312	2830	3134	1562
Solyc01g098210.2.1	-1.30842	4.425761	0.000999	0.006334	736	990	664	1215	1462	786
Solyc03g095510.2.1	-1.30659	6.022518	0.001708	0.009969	1479	3479	2636	3407	4978	2279
Solyc11g062390.1.1	-1.30278	4.659121	0.001087	0.006803	867	1200	763	1415	1881	838
Solyc05g006590.2.1	-1.30261	5.78949	0.001181	0.007303	1558	2799	1985	2847	3956	2137
Solyc06g050730.2.1	-1.30187	4.196343	0.001053	0.006614	586	935	566	880	1448	678
Solyc05g009010.1.1	-1.29993	4.290863	0.001655	0.009693	501	1417	493	1026	1293	800
Solyc07g042590.2.1	-1.29279	5.570799	0.001471	0.008798	1699	2104	1475	2445	2820	2161
Solyc12g005760.1.1	-1.28698	4.379037	0.001484	0.008863	686	1134	586	1267	1188	808
Solyc06g082120.2.1	-1.28046	5.519176	0.001456	0.00873	1548	2184	1467	2805	2947	1577
Solyc01g010940.2.1	-1.28025	5.738303	0.001356	0.008217	1383	2838	2039	2739	3760	2074
Solyc05g008100.1.1	-1.27967	5.027318	0.000929	0.005973	835	1874	1168	2049	1964	1152
Solyc01g100410.2.1	-1.27633	4.809826	0.000729	0.004867	835	1548	905	1458	1965	1076
Solyc01g010150.2.1	-1.27505	4.167407	0.001646	0.009649	415	1095	662	1071	1040	703
Solyc05g018610.1.1	-1.27334	5.390937	0.00081	0.005316	1174	2231	1509	1865	3133	1754
Solyc07g008080.2.1	-1.27096	5.62785	0.00121	0.007451	1472	2672	1648	2802	3327	1784
Solyc12g055750.1.1	-1.26824	4.877011	0.000997	0.006324	774	1611	1092	1744	1726	1138
Solyc07g048030.2.1	-1.26809	4.499523	0.001003	0.006356	647	1243	775	1147	1721	807
Solyc10g078570.1.1	-1.26602	4.33565	0.001129	0.007034	526	1063	787	990	1494	771
Solyc09g008410.2.1	-1.26559	5.3935	0.000993	0.006304	1355	2042	1430	2037	3231	1558
Solyc09g097870.2.1	-1.26242	4.795681	0.001542	0.009164	668	1410	1191	1712	1843	897
Solyc07g065140.2.1	-1.2624	4.779663	0.00089	0.005749	795	1461	969	1545	1791	1026
Solyc02g091520.2.1	-1.25792	4.578637	0.00145	0.008703	544	1560	846	1135	1510	1088
Solyc02g065650.1.1	-1.25699	4.33434	0.001339	0.008126	657	1026	656	1098	1351	759
Solyc04g072010.2.1	-1.256	5.436118	0.001188	0.007343	1417	2349	1311	2360	2919	1618
Solyc04g072120.2.1	-1.25252	5.438624	0.001581	0.009337	1536	2028	1384	2504	2810	1569
Solyc03g031750.1.1	-1.25165	4.662754	0.001075	0.006731	782	1391	819	1307	1807	945
Solyc08g082920.2.1	-1.25115	4.610221	0.001195	0.007382	803	1278	770	1288	1658	937
Solyc04g072110.2.1	-1.24994	5.411472	0.001154	0.007167	1116	2190	1721	2287	2761	1674
Solyc09g059520.2.1	-1.24181	5.099812	0.000943	0.006044	993	1975	1150	1810	2302	1323
Solyc04g015580.2.1	-1.23842	4.639097	0.001353	0.008199	646	1461	916	1418	1634	897
Solyc12g095790.1.1	-1.23789	5.254634	0.001201	0.007412	1171	1986	1343	2145	2524	1386
Solyc05g011930.2.1	-1.23377	4.864596	0.001563	0.009256	953	1799	776	1436	2155	1085
Solyc11g017170.1.1	-1.23285	4.61359	0.001615	0.0095	810	1329	758	1389	1664	847
Solyc12g015740.1.1	-1.22904	4.914587	0.001151	0.00715	833	1577	1183	1568	1947	1218
Solyc10g018160.1.1	-1.22091	4.606459	0.001477	0.008832	707	1430	823	1316	1474	995
Solyc03g116600.2.1	-1.21265	4.417436	0.001559	0.009238	608	1201	780	1115	1488	787
Solyc02g091430.2.1	-1.20617	5.336623	0.001617	0.009507	1372	2129	1307	1990	2614	1697
Solyc02g081380.2.1	-1.20457	5.460853	0.00167	0.009774	1261	2375	1684	2333	2883	1695
Solyc01g010860.2.1	-1.20004	4.891828	0.001409	0.008494	816	1783	1067	1539	1984	1142
Solyc07g005430.2.1	-1.19684	5.352557	0.001679	0.009818	1314	2015	1520	2090	2698	1611
Solyc02g094170.2.1	-1.18263	4.766269	0.001708	0.009969	855	1503	954	1355	1808	1086
Solyc07g066290.2.1	1.203968	4.670867	0.001675	0.009796	1655	3245	2218	481	750	494
Solyc01g096600.2.1	1.20448	4.963488	0.001633	0.009583	1761	4776	2540	657	989	509
Solyc03g121260.2.1	1.218071	4.796395	0.001315	0.007997	1890	3709	2229	509	882	505
Solyc05g005640.2.1	1.226305	5.033718	0.001494	0.008915	1751	4765	2984	594	976	631
Solyc12g100200.1.1	1.236993	5.118574	0.001258	0.0077	2381	4218	3068	878	890	542
Solyc10g085460.1.1	1.238917	5.351327	0.00126	0.007709	2862	5531	3166	707	1308	748
Solyc02g070670.2.1	1.244963	5.141868	0.001509	0.008996	1729	5209	3401	765	973	608
Solyc03g121440.2.1	1.254271	4.844257	0.001173	0.007268	2130	3206	2540	505	873	536
Solyc07g005030.2.1	1.26449	4.690706	0.001119	0.006988	1471	3675	2341	611	711	390
Solyc06g076770.2.1	1.266082	5.043502	0.001266	0.007742	2435	3690	2945	504	1059	635
Solyc08g014470.2.1	1.267657	4.290994	0.001555	0.00922	1132	2483	1947	449	587	277
Solyc05g053710.2.1	1.274266	4.234884	0.001139	0.00709	1291	2510	1550	379	514	336
Solyc03g007920.2.1	1.274401	5.075197	0.001229	0.007549	1669	4512	3570	630	1063	560
Solyc12g056740.1.1	1.275417	5.456007	0.000938	0.006017	2614	6399	3774	877	1299	732
Solyc06g008960.2.1	1.276088	4.328207	0.001595	0.009405	1072	2983	1840	333	617	376
Solyc03g116350.2.1	1.280911	4.8288	0.000776	0.005133	1943	3867	2312	667	709	465
Solyc04g040190.1.1	1.282973	4.90987	0.000752	0.004999	1851	4193	2638	671	887	439
Solyc06g009220.2.1	1.287889	4.54145	0.001463	0.008764	1672	2908	1968	637	565	312
Solyc01g089870.2.1	1.300096	5.13515	0.001197	0.007392	1770	5370	3306	550	1156	621
Solyc04g079060.2.1	1.301145	4.369305	0.001408	0.008489	1172	2819	1994	538	526	278
Solyc10g081720.1.1	1.301999	4.528618	0.001307	0.007955	2009	2351	1870	532	655	327
Solyc07g053610.2.1	1.302658	5.86203	0.001444	0.008667	4147	8391	4294	1037	1626	1089
Solyc04g014500.2.1	1.309704	5.148371	0.001611	0.009484	1933	5277	3263	463	1276	630
Solyc02g089340.2.1	1.311443	4.007041	0.00117	0.007251	926	2518	1332	313	464	266
Solyc03g113350.2.1	1.314109	4.665403	0.001403	0.008466	2377	2845	1698	598	637	390
Solyc05g054940.2.1	1.317893	4.148766	0.001651	0.009675	921	2366	1863	391	386	327
Solyc11g065870.1.1	1.31915	4.398324	0.000901	0.005813	1812	2560	1492	433	566	352
Solyc07g045420.2.1	1.3221	6.181179	0.00141	0.008496	4318	10486	6482	1420	2303	1063
Solyc07g017800.2.1	1.322671	5.027489	0.001462	0.008763	3217	3614	2027	693	1037	430
Solyc03g005870.2.1	1.322703	3.689467	0.001591	0.009386	775	1666	1260	235	358	231
Solyc03g083360.2.1	1.324338	4.632963	0.000806	0.005294	1360	3123	2650	509	695	395
Solyc12g088750.1.1	1.324527	4.421596	0.000752	0.004999	1505	2742	1850	341	690	369
Solyc01g067550.2.1	1.325426	4.75703	0.000952	0.006084	1580	3487	2716	396	846	508
Solyc12g014460.1.1	1.326391	4.952443	0.000503	0.003539	1806	4539	2769	672	807	497
Solyc01g009230.2.1	1.326679	5.297349	0.000493	0.003479	2352	5568	3574	759	1088	672
Solyc09g098330.2.1	1.331848	4.457627	0.001335	0.008102	1074	3266	2196	331	647	425
Solyc11g010490.1.1	1.33201	4.014366	0.00122	0.007505	871	2285	1586	278	453	297
Solyc06g073740.2.1	1.332779	5.511392	0.000922	0.005934	2555	5785	4806	839	1515	660
Solyc02g081850.2.1	1.334281	4.93404	0.000995	0.006315	2866	3141	2258	692	739	495

Solyc01g008480.2.1	1.33625	3.758215	0.00129	0.00787	835	2036	1120	304	365	197
Solyc04g082680.2.1	1.337433	4.657233	0.000986	0.006266	1664	3395	2224	340	782	495
Solyc09g007310.2.1	1.338927	4.706276	0.000717	0.004802	1436	3686	2546	589	597	441
Solyc02g021140.2.1	1.339624	4.601656	0.001298	0.007911	1175	3443	2558	360	775	435
Solyc01g068270.2.1	1.341821	4.32969	0.001125	0.007016	1427	2038	2074	432	440	367
Solyc12g007090.1.1	1.342781	4.325806	0.001023	0.006464	1571	2290	1722	448	414	366
Solyc11g005020.1.1	1.343081	5.156035	0.000397	0.002909	2227	5174	3072	686	1029	572
Solyc02g069080.2.1	1.344586	4.096938	0.000741	0.004933	1054	2316	1594	298	489	301
Solyc02g070260.2.1	1.345006	5.175382	0.001326	0.008057	1610	5172	3964	819	778	631
Solyc12g015680.1.1	1.347512	4.534626	0.000855	0.005563	1829	2592	2017	578	608	296
Solyc09g018790.2.1	1.349853	5.501104	0.000514	0.003602	3397	5508	3894	974	1159	722
Solyc03g006420.2.1	1.351821	4.495257	0.000508	0.003567	1509	3235	1848	387	638	402
Solyc01g068610.2.1	1.351958	5.799603	0.001545	0.009175	2934	7800	5686	1393	1311	793
Solyc01g080810.2.1	1.354829	5.223907	0.00042	0.00305	2416	5000	3411	835	1026	525
Solyc01g102970.2.1	1.35795	4.775041	0.000477	0.003393	1693	3984	2391	429	867	464
Solyc07g040940.2.1	1.361534	5.084532	0.000284	0.002195	2383	4497	2899	701	874	551
Solyc11g069290.1.1	1.36986	5.081146	0.000401	0.002929	2474	3998	3098	521	1031	596
Solyc06g083770.2.1	1.37009	3.683101	0.001055	0.006623	977	1515	1122	234	338	226
Solyc01g102540.2.1	1.370274	4.69615	0.001638	0.009611	1005	4536	2533	413	814	430
Solyc07g045310.2.1	1.370721	4.438678	0.001482	0.008861	2171	2017	1602	515	541	302
Solyc01g091860.2.1	1.373468	3.675494	0.00163	0.00957	881	1746	1078	179	341	265
Solyc02g090380.2.1	1.375008	3.900087	0.001217	0.007491	863	2334	1282	373	353	202
Solyc04g014870.2.1	1.377392	4.277525	0.00058	0.003993	1231	2647	1780	416	426	337
Solyc11g011250.1.1	1.377775	4.909344	0.000637	0.00434	1526	4442	3022	462	851	561
Solyc12g062480.1.1	1.378472	3.456771	0.001542	0.009164	701	1492	1003	222	331	148
Solyc07g006140.2.1	1.37975	4.468038	0.000535	0.003735	1838	2677	1719	510	592	293
Solyc03g043600.1.1	1.380026	3.917542	0.000756	0.005016	916	2328	1271	267	443	243
Solyc02g068240.2.1	1.380663	5.322686	0.000613	0.004194	3534	4518	3064	975	919	576
Solyc02g069830.2.1	1.382347	3.656143	0.001227	0.007537	1082	1525	932	252	316	209
Solyc01g096900.2.1	1.385109	5.008784	0.000832	0.005432	1366	5236	3280	538	971	526
Solyc04g076850.2.1	1.389286	7.224018	0.001133	0.007058	10420	20717	12545	2631	4143	2540
Solyc02g091240.1.1	1.389418	4.921968	0.000318	0.002417	2097	3786	2818	479	836	560
Solyc12g010330.1.1	1.390095	4.003399	0.000893	0.005767	890	2176	1647	244	452	297
Solyc11g022460.1.1	1.390284	3.962136	0.00114	0.007094	866	2225	1529	214	493	276
Solyc12g049400.1.1	1.397121	4.886548	0.001368	0.008278	2543	3250	2429	790	834	267
Solyc08g082090.1.1	1.399776	5.182617	0.001693	0.009887	2389	7412	1718	584	1081	608
Solyc07g066670.2.1	1.400681	4.881292	0.00118	0.007303	1268	5094	2811	404	988	485
Solyc05g006900.1.1	1.401235	4.433742	0.001088	0.006805	1248	3732	1666	264	685	404
Solyc09g092330.1.1	1.402961	5.968493	0.000793	0.005228	3636	9644	5577	1000	2005	969
Solyc08g076470.2.1	1.405105	4.683783	0.000968	0.006161	2080	3078	2118	641	769	238
Solyc07g064950.2.1	1.405909	4.858557	0.000325	0.002464	1745	3952	2835	449	833	513
Solyc12g010740.1.1	1.406273	3.518072	0.000967	0.006161	739	1657	989	222	311	175
Solyc04g014210.2.1	1.407662	5.219575	0.000288	0.00222	2353	5661	3136	628	953	684
Solyc10g076860.1.1	1.41008	3.496406	0.001186	0.007333	639	1631	1087	223	289	178
Solyc08g063040.2.1	1.411035	5.009917	0.000949	0.00607	3373	3441	1984	737	766	464
Solyc03g019940.2.1	1.411192	5.147812	0.000481	0.003417	1672	5361	3708	649	896	612
Solyc11g065180.1.1	1.411704	4.397708	0.001055	0.006623	1018	3389	2042	271	635	397
Solyc04g080770.2.1	1.415317	4.013741	0.000612	0.004188	870	2216	1696	295	406	278
Solyc02g083180.2.1	1.417389	4.871877	0.000582	0.004011	1444	4639	2856	449	726	580
Solyc01g111600.2.1	1.417809	5.415148	0.000815	0.005341	3973	4637	3206	1089	865	603
Solyc03g033420.2.1	1.418037	4.49097	0.000616	0.004209	1123	3073	2486	345	651	390
Solyc03g120670.2.1	1.419485	5.012553	0.000213	0.001716	2192	4294	2932	555	766	609
Solyc04g063230.2.1	1.420651	5.023761	0.000777	0.005138	1747	4431	3465	822	907	320
Solyc11g068720.1.1	1.421429	3.68457	0.001159	0.007198	749	1792	1264	174	415	215
Solyc03g116670.2.1	1.424771	7.408245	0.001107	0.006922	11051	26506	13640	3632	3787	2753
Solyc09g011170.2.1	1.42542	5.814551	0.00065	0.004418	3349	6727	6209	1057	1669	789
Solyc07g018000.2.1	1.42597	3.571784	0.001393	0.008409	597	1980	1083	186	346	199
Solyc09g083410.2.1	1.426957	4.290635	0.000441	0.003181	1258	2449	1966	409	404	341
Solyc09g074330.2.1	1.429964	3.927191	0.001366	0.008267	755	2643	1333	197	474	269
Solyc05g013300.1.1	1.430059	4.32249	0.000621	0.00424	1935	2276	1355	367	502	337
Solyc06g011530.2.1	1.430361	3.427431	0.001119	0.006986	794	1367	938	191	337	146
Solyc03g114510.2.1	1.431165	3.866203	0.000878	0.005688	942	1962	1372	374	307	192
Solyc01g005020.2.1	1.433599	6.156485	0.001201	0.007412	3124	10648	7939	1255	2031	1105
Solyc02g093970.2.1	1.437186	4.524168	0.000267	0.002081	1871	3097	1702	483	619	310
Solyc08g076720.2.1	1.438048	3.879084	0.000953	0.006089	1227	2062	995	347	399	164
Solyc02g091840.2.1	1.43955	6.361617	0.000796	0.005244	4969	11957	7685	1157	2505	1404
Solyc07g063570.2.1	1.444919	5.378232	0.000493	0.003479	3211	5289	3510	1118	859	506
Solyc07g021550.2.1	1.44606	3.934118	0.000946	0.00605	786	2303	1542	207	420	290
Solyc05g048810.2.1	1.446302	3.464022	0.00166	0.009717	516	2018	947	195	309	168
Solyc01g010270.2.1	1.446762	4.015355	0.00158	0.009337	598	2660	1780	262	500	239
Solyc04g009510.2.1	1.449877	4.486732	0.001581	0.009339	2480	2129	1424	555	506	283
Solyc01g094290.2.1	1.452363	6.215882	0.000718	0.004809	4545	10834	6909	1015	2257	1276
Solyc09g074410.2.1	1.453386	3.396127	0.001691	0.009876	712	1205	1093	204	197	201
Solyc05g018210.2.1	1.454206	4.552209	0.001649	0.009663	1695	3092	2060	738	405	238
Solyc08g077510.2.1	1.455212	4.873423	0.0002	0.00163	2494	3178	2549	568	814	407
Solyc05g009760.1.1	1.457546	3.738003	0.000493	0.003479	1027	1635	1169	284	308	199
Solyc11g008910.1.1	1.459375	3.33562	0.001099	0.006873	777	1222	892	166	258	175
Solyc02g086550.2.1	1.463407	4.059391	0.000258	0.00202	1255	2213	1395	276	425	288
Solyc10g083520.1.1	1.466213	4.862833	0.000201	0.001636	1788	4458	2567	416	807	518
Solyc03g119400.2.1	1.46837	4.275839	0.000438	0.003161	975	3067	1911	330	614	255
Solyc03g026150.2.1	1.468428	4.047469	0.000213	0.001716	1130	2307	1458	315	406	259
Solyc03g034020.2.1	1.468891	4.737477	0.000121	0.001067	1827	3854	2272	477	750	389
Solyc05g005180.2.1	1.468901	3.971719	0.000645	0.004393	1359	1691	1345	351	301	252
Solyc10g081040.1.1	1.470295	5.300299	0.000669	0.004518	2622	7731	2162	617	1275	549

Solyc06g075390.2.1	1.47098	3.976016	0.000535	0.003734	1053	2442	1260	383	387	177
Solyc07g045190.1.1	1.471684	3.825771	0.000515	0.003606	1105	1583	1334	261	304	254
Solyc06g082750.2.1	1.471738	4.43986	0.000883	0.005709	1273	2692	2395	535	328	359
Solyc01g111400.2.1	1.472035	5.671429	0.00065	0.004414	4518	6342	3737	669	1804	724
Solyc01g011510.2.1	1.472581	5.315259	0.000663	0.004488	3365	5413	2830	1025	1061	357
Solyc01g008180.2.1	1.472627	3.444138	0.00089	0.00575	803	1384	971	176	353	144
Solyc03g032150.2.1	1.472986	3.796605	0.00049	0.00347	808	2051	1331	229	437	189
Solyc03g121050.2.1	1.473069	3.623172	0.000628	0.004285	911	1549	1109	187	308	227
Solyc03g007600.2.1	1.476871	5.835337	0.000552	0.003833	5092	6585	4494	1321	1366	719
Solyc12g017420.1.1	1.477046	4.451296	0.000146	0.001245	1521	2981	1955	441	486	349
Solyc01g090200.2.1	1.477054	5.01162	0.000147	0.00125	2114	4345	3082	733	825	385
Solyc07g055290.2.1	1.477909	4.33642	0.000178	0.001473	1304	2890	1843	316	571	325
Solyc12g009150.1.1	1.478001	5.093106	0.000126	0.001098	1986	5011	3310	575	867	574
Solyc11g032200.1.1	1.478331	3.163782	0.001215	0.007478	622	1164	832	181	213	133
Solyc01g099110.2.1	1.478566	5.223833	0.000334	0.002523	3527	4532	2558	855	822	518
Solyc03g113370.2.1	1.47918	3.777688	0.000932	0.005988	804	2231	1177	161	407	252
Solyc05g051680.2.1	1.479637	4.195551	0.000337	0.002542	1715	2225	1265	337	476	277
Solyc02g085780.2.1	1.482284	4.898943	0.000293	0.002251	1795	4114	2994	371	835	561
Solyc01g109850.2.1	1.482429	6.421282	0.000341	0.002568	6155	11288	7725	1687	2129	1265
Solyc01g094830.2.1	1.483016	4.085874	0.000717	0.004803	1006	2361	1706	192	468	338
Solyc10g051200.1.1	1.484824	4.677545	0.000354	0.002635	1663	3704	2305	674	527	299
Solyc09g064270.2.1	1.485812	4.265156	0.000553	0.003837	1039	2892	1921	234	556	353
Solyc01g105670.2.1	1.485935	4.17183	0.000222	0.001775	1197	2305	1782	385	388	273
Solyc07g065260.2.1	1.486481	4.706125	0.000483	0.003427	1361	4149	2533	310	849	431
Solyc01g079650.2.1	1.487354	3.716695	0.000826	0.005403	667	2383	1106	267	371	158
Solyc03g097000.2.1	1.487696	3.211478	0.001437	0.008638	564	1348	870	127	276	152
Solyc02g063450.2.1	1.487763	7.961989	0.000917	0.005911	15624	30226	27058	5661	6206	3034
Solyc09g010190.2.1	1.487775	3.818842	0.000551	0.003825	773	1986	1482	201	413	231
Solyc12g096550.1.1	1.490373	5.852143	0.000489	0.003467	5109	7834	3891	850	1727	906
Solyc03g025600.2.1	1.492029	5.750519	0.000346	0.002594	4415	6438	4608	868	1236	997
Solyc08g075530.2.1	1.492146	5.526669	0.000353	0.002633	2300	7536	4491	655	1314	777
Solyc09g009430.2.1	1.492637	6.28488	0.000368	0.00273	5629	9201	7709	1418	1836	1286
Solyc04g049380.2.1	1.496252	4.255035	0.001322	0.008034	1848	2037	1438	541	368	201
Solyc03g059070.2.1	1.496337	3.413999	0.001283	0.007834	687	1406	1053	125	332	182
Solyc11g008990.1.1	1.496389	5.323045	9.15E-05	0.000837	3190	4933	3454	762	972	611
Solyc10g008630.2.1	1.49747	4.360875	0.000218	0.001751	1342	3092	1777	486	445	266
Solyc05g050940.2.1	1.498346	3.016168	0.001483	0.008861	522	1057	803	147	234	105
Solyc03g113770.2.1	1.499244	3.877823	0.000599	0.00411	678	2469	1455	241	420	217
Solyc12g099280.1.1	1.500323	4.415341	0.000252	0.001982	1411	3326	1755	292	724	296
Solyc02g093940.2.1	1.501286	3.547952	0.001588	0.009375	568	1831	1205	142	292	237
Solyc09g008730.2.1	1.501371	3.910048	0.000246	0.001942	1099	2103	1256	235	415	239
Solyc02g091640.2.1	1.502032	4.425604	0.00021	0.0017	1104	3365	2152	379	621	291
Solyc08g077890.2.1	1.502249	6.791764	0.000611	0.00418	6819	14862	11335	2585	2430	1443
Solyc02g068030.1.1	1.503256	5.06429	0.000278	0.002156	3384	3908	2144	546	983	480
Solyc07g019440.2.1	1.503731	5.101977	0.00014	0.001206	1925	5098	3428	503	934	583
Solyc08g082400.1.1	1.505881	4.628266	0.001191	0.007358	2873	2481	1384	553	623	291
Solyc06g065440.1.1	1.506523	5.474699	0.000519	0.00363	2028	6176	5294	919	848	744
Solyc12g006480.1.1	1.506634	6.014275	0.001036	0.006529	2716	11851	6160	893	1971	1015
Solyc05g015490.2.1	1.507248	6.391776	0.0004	0.002925	5449	13832	6624	1613	2419	1031
Solyc12g068070.1.1	1.510169	4.594591	0.00039	0.002866	1184	3798	2491	313	649	444
Solyc01g081050.2.1	1.516134	4.500872	0.000133	0.001152	1303	3772	1965	386	617	332
Solyc11g030600.2.1	1.516449	5.177068	0.000183	0.001506	2629	5297	2936	439	1123	597
Solyc06g064940.2.1	1.516966	5.284765	0.000256	0.00201	2072	6085	3868	492	1242	609
Solyc10g078750.1.1	1.519172	4.39789	0.000307	0.002344	2131	2420	1391	363	589	296
Solyc02g088000.2.1	1.519936	6.815316	0.000334	0.002523	7490	16883	9793	2275	2704	1573
Solyc05g049900.2.1	1.519965	3.620472	0.000449	0.003228	782	1862	1091	171	352	200
Solyc02g081910.1.1	1.520568	4.146424	0.001037	0.006532	697	3025	1890	249	614	224
Solyc10g005180.2.1	1.520672	5.032052	0.000264	0.002061	2431	3995	3101	861	704	339
Solyc01g087290.2.1	1.522294	4.151175	0.000375	0.002767	1234	2383	1650	461	351	206
Solyc08g065150.1.1	1.523248	3.642632	0.000292	0.002248	812	1732	1189	224	313	187
Solyc05g026140.2.1	1.525585	3.285589	0.001227	0.00754	921	1137	709	211	245	115
Solyc06g062480.2.1	1.527847	3.878145	0.000205	0.001666	924	2224	1327	237	382	233
Solyc10g024410.1.1	1.528462	5.37365	7.14E-05	0.000679	2900	5822	3688	664	1192	597
Solyc08g067030.2.1	1.53162	2.960706	0.001283	0.007834	575	935	747	121	235	106
Solyc12g042770.1.1	1.533472	6.979052	0.000566	0.003916	11769	14158	9891	2674	3164	1543
Solyc05g005770.2.1	1.533953	3.526638	0.000395	0.002895	797	1874	867	209	290	167
Solyc04g010290.2.1	1.534651	3.285637	0.001365	0.008263	556	1361	1022	113	295	164
Solyc11g069500.1.1	1.536973	3.404883	0.000569	0.003931	825	1205	1013	216	263	135
Solyc02g036470.2.1	1.538325	4.520023	0.000172	0.001431	1998	2932	1743	524	473	305
Solyc09g011710.2.1	1.542604	7.173376	0.001459	0.008748	5519	27635	13952	2150	4408	2021
Solyc08g076450.2.1	1.542684	5.666087	0.00165	0.009667	5172	5409	3641	1498	910	476
Solyc12g005180.1.1	1.543546	5.327623	0.000438	0.003161	3227	4775	3632	1120	687	447
Solyc01g087250.2.1	1.545499	5.435012	0.000179	0.001479	2771	6540	3903	1073	924	482
Solyc09g075860.2.1	1.546087	5.279207	0.000387	0.002847	4030	3996	2785	916	749	517
Solyc06g076850.2.1	1.546211	8.404142	0.001151	0.00715	34155	36272	24732	7979	7583	3928
Solyc04g082400.2.1	1.547849	6.053406	0.00039	0.002864	3760	10924	6055	1562	1310	870
Solyc06g073780.2.1	1.547961	5.876764	0.000214	0.001724	3571	9242	5323	1272	1385	728
Solyc05g056260.2.1	1.548786	5.096872	7.45E-05	0.000704	2181	4669	3425	478	932	564
Solyc03g096800.2.1	1.548816	5.508518	0.000368	0.002727	4514	5793	2828	1020	1146	492
Solyc01g104910.2.1	1.549323	5.352558	0.000195	0.001597	2283	7897	3011	584	1117	660
Solyc08g006510.2.1	1.549386	3.079957	0.001179	0.007298	450	1301	853	169	213	106
Solyc04g015450.2.1	1.549841	6.32053	0.000648	0.004408	7356	9546	6080	2003	1570	947
Solyc07g005140.2.1	1.549896	3.503308	0.00168	0.009818	430	1559	1482	225	303	134
Solyc09g042740.2.1	1.550654	4.632139	8.25E-05	0.000767	1640	3203	2521	513	531	355

Solyc01g105540.2.1	1.550904	4.885441	0.000113	0.001006	2731	3065	2526	594	718	388
Solyc02g021050.2.1	1.551122	3.559025	0.000457	0.003273	743	1617	1176	224	223	201
Solyc09g008930.2.1	1.551412	4.682224	0.000845	0.005509	2801	2680	1650	657	442	327
Solyc03g006140.2.1	1.55163	6.336803	0.00026	0.002033	5461	9251	8865	1458	1935	1216
Solyc09g072650.2.1	1.556682	3.851692	0.000945	0.00605	539	2524	1561	211	358	247
Solyc03g097130.2.1	1.557198	3.737385	0.000903	0.005824	1139	1624	1099	367	247	136
Solyc12g017410.1.1	1.558118	3.509332	0.000371	0.002745	658	1726	1110	183	307	166
Solyc04g039730.2.1	1.558142	3.127353	0.000934	0.005997	525	1248	873	174	231	102
Solyc09g065120.2.1	1.558607	5.562868	0.000111	0.000987	4256	6024	3487	804	1117	751
Solyc11g062100.1.1	1.559533	4.203376	0.000873	0.005662	692	3003	2133	272	622	216
Solyc01g096470.2.1	1.559747	3.302579	0.000761	0.005041	709	1353	879	143	208	190
Solyc05g008940.2.1	1.56481	3.285038	0.001016	0.006424	527	1677	869	222	230	106
Solyc01g007740.2.1	1.566574	4.88115	6.47E-05	0.000624	1611	4339	3100	543	700	426
Solyc03g081240.2.1	1.566741	5.13644	0.000223	0.001786	3662	3225	2793	557	992	492
Solyc08g007950.2.1	1.568877	4.483032	9.11E-05	0.000835	1292	3084	2398	391	488	364
Solyc03g098280.2.1	1.570228	6.74642	0.000822	0.005381	5380	17889	10631	1197	3430	1676
Solyc09g014450.2.1	1.57033	4.269348	0.000271	0.00211	925	3484	1777	269	566	284
Solyc01g097800.2.1	1.571779	3.415203	0.001037	0.006531	608	1585	1076	119	257	213
Solyc01g103750.2.1	1.572746	5.329124	6.39E-05	0.000617	2865	5034	3981	730	788	682
Solyc01g096870.2.1	1.57489	5.747887	0.000308	0.002348	2611	8725	5572	762	1305	938
Solyc06g051080.2.1	1.574974	4.666996	5.76E-05	0.000564	1762	3869	2137	406	749	328
Solyc01g094890.2.1	1.575663	5.090193	0.000494	0.003484	1556	4467	4329	367	1013	580
Solyc12g096500.1.1	1.576058	6.853278	0.00024	0.001904	8756	15613	10133	2459	2676	1426
Solyc02g068910.2.1	1.57624	4.326154	0.000799	0.005258	998	3119	2131	185	524	408
Solyc04g016240.2.1	1.576624	4.906912	0.000489	0.003469	1268	5076	3212	330	953	469
Solyc05g013960.2.1	1.577102	4.139886	8.72E-05	0.000804	1451	2138	1546	343	410	240
Solyc11g006340.1.1	1.577952	4.474717	0.001258	0.0077	952	4336	1998	211	833	300
Solyc04g049390.2.1	1.578233	3.940816	0.000276	0.002145	1446	1603	1286	328	346	191
Solyc05g013310.1.1	1.578359	3.034092	0.001489	0.008889	805	1025	530	173	170	111
Solyc06g065480.2.1	1.57844	5.595592	0.000727	0.004857	5209	5303	3099	1224	826	603
Solyc07g017990.2.1	1.579042	3.094845	0.000945	0.00605	472	1545	705	155	209	118
Solyc07g066430.2.1	1.579624	4.263263	0.000717	0.004802	724	3236	2170	241	486	346
Solyc06g082610.2.1	1.581467	2.806523	0.001417	0.008525	498	899	672	140	195	71
Solyc03g113800.2.1	1.584889	6.706901	0.000256	0.002005	6000	14956	11025	2093	2322	1428
Solyc06g008760.1.1	1.586076	4.685987	0.00055	0.003822	986	3933	3161	337	836	342
Solyc07g062610.2.1	1.588311	5.035735	5.88E-05	0.000575	2098	4594	3257	676	597	506
Solyc06g048540.2.1	1.588403	5.423922	0.000192	0.001572	2157	6495	4690	523	1132	754
Solyc07g063530.2.1	1.589485	5.129105	0.000237	0.001882	3385	3836	2736	872	702	378
Solyc04g005800.2.1	1.590224	5.452971	0.000274	0.002128	1921	6340	5320	642	1338	573
Solyc01g095280.2.1	1.590647	3.867412	0.000287	0.002212	759	2262	1521	189	397	238
Solyc05g013320.1.1	1.592048	4.32751	0.00019	0.001564	2049	2443	1280	385	461	275
Solyc01g103430.2.1	1.595671	6.354619	0.000266	0.002077	7374	10243	6297	1255	1703	1479
Solyc11g020300.1.1	1.596521	5.726617	0.000863	0.005607	2241	8874	5779	521	1624	890
Solyc06g062840.2.1	1.596731	3.291515	0.001062	0.006662	661	1282	980	187	139	183
Solyc01g106430.2.1	1.597241	6.287343	0.000695	0.004671	6950	8059	7218	1980	1217	1008
Solyc05g014790.2.1	1.597595	4.870883	4.01E-05	0.000414	1685	4381	2968	482	742	422
Solyc11g072420.1.1	1.598977	3.808135	0.000128	0.001112	817	2199	1337	254	332	195
Solyc07g054090.2.1	1.603049	2.919886	0.001392	0.008405	419	1203	736	86	223	120
Solyc04g076180.2.1	1.603388	4.727911	8.50E-05	0.000788	1542	4332	2442	331	754	417
Solyc06g053980.2.1	1.604578	3.835919	0.00066	0.004467	1160	1666	1329	391	258	136
Solyc04g049850.2.1	1.604697	4.116248	8.23E-05	0.000766	999	2648	1726	262	431	271
Solyc01g079680.2.1	1.606041	4.375811	0.000687	0.004624	1062	3404	2087	173	595	393
Solyc02g091530.2.1	1.606176	3.43476	0.000592	0.004068	740	1471	1042	269	218	111
Solyc05g012790.2.1	1.606757	3.772084	0.000223	0.001782	1065	1337	1492	220	374	181
Solyc05g006650.2.1	1.609775	4.546108	0.000149	0.001266	2239	2711	1774	529	442	300
Solyc01g068600.2.1	1.61034	2.94257	0.00153	0.009102	390	1231	789	176	184	73
Solyc11g010300.1.1	1.61043	3.960137	0.000126	0.001099	1361	1830	1325	324	341	195
Solyc05g053280.2.1	1.610636	4.161707	1.00E-04	0.000904	1002	2678	1857	262	411	304
Solyc06g075790.2.1	1.61163	4.220358	5.62E-05	0.000552	1476	2389	1655	352	393	272
Solyc03g118860.2.1	1.612842	3.11309	0.001027	0.006481	677	1076	793	213	159	98
Solyc12g096760.1.1	1.617078	5.787477	8.93E-05	0.000821	3318	8170	5557	1044	1203	780
Solyc02g079770.2.1	1.617211	6.537425	0.00017	0.001419	7896	10779	8370	1357	2480	1373
Solyc09g074520.2.1	1.617351	5.173805	4.77E-05	0.000478	3097	4911	2749	682	690	565
Solyc06g063300.2.1	1.621543	3.371012	0.000488	0.003461	720	1497	931	125	236	195
Solyc07g032110.2.1	1.622065	3.492141	0.000194	0.001585	701	1701	1070	201	287	141
Solyc07g055200.2.1	1.622699	5.599663	0.000165	0.001383	2355	7789	5189	608	1358	755
Solyc07g016180.2.1	1.625533	4.484071	0.000145	0.001239	1095	4169	2014	483	480	266
Solyc05g005860.1.1	1.62652	2.59356	0.001241	0.007612	435	865	526	122	119	83
Solyc11g071460.1.1	1.626545	5.480954	9.48E-05	0.000863	4068	4532	4114	800	859	723
Solyc01g094940.2.1	1.626883	4.398097	4.69E-05	0.000472	1760	2701	1778	299	595	294
Solyc04g077660.2.1	1.628682	4.364739	0.000357	0.002655	1123	3210	2114	185	621	351
Solyc03g025590.2.1	1.628864	3.69966	0.000111	0.000988	1062	1690	1103	210	291	203
Solyc09g083330.2.1	1.629247	3.571434	0.000932	0.005988	1361	1082	823	246	289	128
Solyc02g093460.2.1	1.629335	3.308327	0.000481	0.003417	496	1501	1091	165	229	146
Solyc03g121480.2.1	1.629997	3.723352	9.73E-05	0.000882	1023	1634	1246	226	304	191
Solyc04g064680.2.1	1.630223	3.437203	0.000275	0.002138	706	1537	1058	199	200	174
Solyc04g081420.2.1	1.631078	3.240311	0.000994	0.006307	858	1156	741	230	207	86
Solyc05g010610.2.1	1.631181	2.485003	0.001298	0.007911	375	847	495	88	139	80
Solyc03g119110.2.1	1.631878	2.670408	0.001051	0.006602	513	745	597	121	136	87
Solyc09g010300.2.1	1.633158	3.942634	0.00022	0.001765	1310	1805	1369	360	257	199
Solyc02g083110.1.1	1.635321	2.862014	0.001686	0.009851	609	939	600	189	139	67
Solyc10g007840.2.1	1.639533	3.680863	0.000321	0.002436	771	1927	1273	136	326	233
Solyc05g009630.2.1	1.640545	3.351762	0.000411	0.002997	697	1614	858	201	283	95
Solyc03g080060.1.1	1.641763	5.426889	0.000924	0.005945	3861	5620	3265	1287	772	293

Solyc03g124030.2.1	1.641871	5.101831	0.000444	0.003201	2772	4640	2890	1005	526	304
Solyc06g036000.2.1	1.644352	3.40453	0.000469	0.003342	628	1544	1092	116	286	178
Solyc01g088460.2.1	1.645279	2.904676	0.000889	0.005746	651	821	685	128	135	127
Solyc06g062900.2.1	1.648413	2.44453	0.001479	0.008846	427	754	452	86	107	92
Solyc12g015970.1.1	1.648829	4.851378	1.75E-05	0.000199	2222	3791	2641	515	731	352
Solyc01g087200.2.1	1.648891	3.861059	0.00049	0.003471	625	2586	1502	159	416	230
Solyc12g088400.1.1	1.649234	3.81962	0.000919	0.005917	697	2934	1080	129	461	211
Solyc01g087240.2.1	1.649788	4.335307	6.56E-05	0.00063	1113	3444	1884	272	520	312
Solyc06g073180.2.1	1.650313	3.846184	0.000837	0.005465	1102	2889	679	312	365	128
Solyc12g043020.1.1	1.65153	4.501119	8.14E-05	0.000759	1443	3110	2356	254	587	389
Solyc03g097460.2.1	1.652425	5.413281	2.18E-05	0.00024	2911	6279	3933	694	896	678
Solyc12g005300.1.1	1.653012	4.297642	0.000637	0.004337	1250	1630	2732	477	332	231
Solyc04g008290.2.1	1.653779	5.650121	0.000688	0.00463	2571	8442	5042	378	1489	905
Solyc01g103530.2.1	1.653853	4.297588	0.000364	0.002705	900	2617	2537	218	480	356
Solyc09g013100.2.1	1.654541	4.32089	3.42E-05	0.00036	1199	2908	2069	347	469	270
Solyc10g005330.2.1	1.65482	4.979058	1.75E-05	0.000199	1928	5118	2892	507	799	426
Solyc03g118380.1.1	1.655413	3.430632	0.000194	0.001585	808	1597	893	148	266	169
Solyc10g084280.1.1	1.655519	4.616279	9.69E-05	0.000879	1270	3788	2653	290	643	403
Solyc01g105690.2.1	1.656521	3.107209	0.000389	0.002861	713	1130	722	150	220	104
Solyc11g011230.1.1	1.656998	2.974332	0.000515	0.003606	613	1087	667	109	181	129
Solyc05g016380.2.1	1.657361	3.444567	0.000165	0.001383	684	1774	963	192	247	148
Solyc12g094630.1.1	1.657405	5.876011	0.001505	0.008977	6288	5706	4495	1715	885	513
Solyc04g080380.2.1	1.658318	3.269688	0.001643	0.009633	839	1317	726	272	173	74
Solyc01g079860.2.1	1.658813	3.247907	0.00042	0.00305	499	1488	999	163	250	112
Solyc12g007310.1.1	1.658852	6.002646	5.28E-05	0.000523	4339	8836	6384	1124	1541	835
Solyc06g053840.2.1	1.659698	4.346469	8.40E-05	0.000779	1643	3480	1256	276	506	319
Solyc12g049390.1.1	1.659804	4.199778	0.000176	0.001458	926	2784	2054	204	493	304
Solyc07g055470.2.1	1.660079	5.466155	0.000179	0.001479	4683	4998	3000	1000	863	506
Solyc04g051580.2.1	1.660205	3.745281	0.000236	0.001877	599	2034	1589	210	319	195
Solyc04g015180.2.1	1.662344	2.912113	0.001621	0.009526	367	1222	798	79	259	93
Solyc09g011480.2.1	1.664723	4.043248	3.37E-05	0.000355	1155	2372	1524	305	396	198
Solyc02g021700.2.1	1.666802	3.130719	0.000539	0.003755	520	1367	852	106	249	127
Solyc06g051960.2.1	1.667385	5.24477	4.68E-05	0.000471	3637	4333	3025	750	731	521
Solyc12g070270.1.1	1.668832	3.122541	0.001203	0.007422	798	1032	709	220	176	74
Solyc06g007430.1.1	1.671549	5.218192	1.92E-05	0.000215	2827	5747	2948	541	1023	490
Solyc06g064460.2.1	1.671956	3.771159	0.000385	0.00283	1284	1761	988	345	249	132
Solyc12g096770.1.1	1.672537	4.190862	0.000347	0.002601	765	2697	2289	412	406	175
Solyc03g025310.2.1	1.673336	4.685824	2.13E-05	0.000235	1636	3582	2680	362	630	392
Solyc02g087140.2.1	1.673651	3.415357	0.000223	0.001785	778	1525	955	148	329	123
Solyc02g091950.2.1	1.675732	3.249388	0.000927	0.005964	432	1422	1134	107	294	129
Solyc10g039290.1.1	1.676273	5.004203	6.15E-05	0.000596	3326	4056	2018	565	831	368
Solyc07g045210.1.1	1.677153	4.907092	6.10E-05	0.000593	1433	4978	3208	395	736	476
Solyc05g052350.2.1	1.67727	2.903858	0.000517	0.003619	580	1030	649	130	135	120
Solyc01g106010.2.1	1.677565	4.18525	0.000101	0.000913	1804	2172	1321	387	364	215
Solyc02g067890.2.1	1.679064	4.595801	0.000142	0.001219	1491	3439	2533	228	600	452
Solyc06g069390.2.1	1.679115	2.702978	0.000795	0.005236	511	757	645	88	164	97
Solyc03g025510.2.1	1.682488	4.057933	6.64E-05	0.000637	1243	2238	1560	343	419	154
Solyc07g018010.2.1	1.682972	3.531401	0.000308	0.002351	852	1932	852	203	352	99
Solyc09g010120.2.1	1.683348	3.244009	0.000432	0.003123	835	1035	875	116	232	155
Solyc07g006520.2.1	1.685693	4.842216	9.15E-05	0.000837	2710	3246	2381	684	524	304
Solyc02g036350.2.1	1.685899	9.26411	0.00018	0.001487	53958	82791	48008	8777	16851	8098
Solyc02g069020.2.1	1.687589	3.232082	0.000534	0.003728	714	1266	859	105	317	110
Solyc11g068730.1.1	1.689312	4.756899	1.18E-05	0.00014	1788	3941	2636	409	650	386
Solyc01g056690.2.1	1.690318	3.292198	0.000186	0.001531	723	1347	905	170	192	146
Solyc04g017690.2.1	1.690561	7.615621	0.000372	0.002751	19484	23247	14540	4443	3459	2332
Solyc09g091860.2.1	1.690638	3.295493	0.000208	0.001681	595	1434	1015	150	257	125
Solyc06g062600.2.1	1.691648	4.035674	0.00017	0.001414	1358	2270	1334	400	345	137
Solyc04g072690.2.1	1.691746	2.340606	0.001205	0.007429	336	811	436	82	130	61
Solyc11g066660.1.1	1.691784	3.531464	0.000975	0.006204	1303	1166	786	278	247	97
Solyc03g097590.2.1	1.69725	5.615719	0.000105	0.000943	4791	5907	3681	643	1580	541
Solyc02g089120.2.1	1.698079	3.750164	6.62E-05	0.000636	1010	2040	1111	270	252	176
Solyc07g042150.2.1	1.698654	3.119821	0.000437	0.003155	528	1207	939	116	261	103
Solyc04g079940.2.1	1.699834	6.16386	8.97E-05	0.000823	6854	9282	5172	1351	1673	823
Solyc08g048390.1.1	1.701756	5.189867	9.91E-05	0.000897	4025	3822	2542	697	903	379
Solyc11g068820.1.1	1.701893	5.292949	0.001551	0.009202	4292	3521	3141	1198	475	328
Solyc10g085680.1.1	1.70215	2.35643	0.001378	0.008328	326	680	549	87	93	80
Solyc04g051190.2.1	1.702161	5.840791	7.36E-05	0.000697	3903	8649	5308	1271	1175	593
Solyc08g023490.2.1	1.702471	2.41417	0.001431	0.008605	404	726	481	121	109	51
Solyc11g062430.1.1	1.704539	3.804315	4.38E-05	0.000445	911	2092	1346	216	370	170
Solyc06g082390.2.1	1.704554	5.19878	1.09E-05	0.000131	2455	5923	3215	545	867	531
Solyc03g044160.1.1	1.706015	3.914357	9.24E-05	0.000845	808	2660	1415	192	406	213
Solyc11g010280.1.1	1.70658	5.199468	0.000398	0.002914	2102	6176	3499	262	903	740
Solyc11g017200.1.1	1.708003	4.763752	1.09E-05	0.000131	2039	3576	2615	514	548	354
Solyc11g013750.1.1	1.709508	3.369171	0.000699	0.004696	989	1280	774	264	164	103
Solyc05g051770.2.1	1.709595	2.937898	0.00038	0.002803	602	1006	697	152	140	101
Solyc12g095780.1.1	1.710318	3.307127	0.000333	0.002518	476	1403	1212	163	222	134
Solyc10g086240.1.1	1.710428	4.794052	0.000429	0.003111	1448	4513	2911	195	715	539
Solyc06g084290.2.1	1.711499	3.106714	0.000394	0.002887	546	1146	939	113	254	104
Solyc08g079880.1.1	1.711614	3.330117	0.001205	0.00743	483	895	1572	148	279	119
Solyc01g079820.2.1	1.714078	4.83245	7.07E-05	0.000673	1585	4196	3153	283	765	466
Solyc01g017600.2.1	1.716902	4.997281	0.000243	0.001925	1746	2688	4862	528	587	506
Solyc04g081070.2.1	1.717292	5.279238	6.00E-05	0.000585	2246	6131	3940	384	1123	588
Solyc03g078000.2.1	1.718302	5.925446	0.000267	0.002084	5104	7227	5715	1586	994	560
Solyc12g021360.1.1	1.718992	3.687985	6.12E-05	0.000595	1045	1638	1181	254	249	162

Solyc10g008790.2.1	1.719737	2.363744	0.001235	0.00758	326	682	559	101	94	67
Solyc04g045470.2.1	1.720246	2.887581	0.000773	0.005114	397	1050	855	149	186	64
Solyc07g006810.2.1	1.72057	3.899437	4.68E-05	0.000471	844	2493	1444	217	378	198
Solyc03g120710.2.1	1.721006	5.226684	2.43E-05	0.000264	1909	6171	3957	687	774	485
Solyc10g038190.1.1	1.721367	2.433715	0.000939	0.006019	371	646	597	95	111	72
Solyc11g066110.1.1	1.721805	6.314461	0.000578	0.003984	7765	9278	6278	2156	1218	712
Solyc01g109830.2.1	1.724133	4.204638	6.75E-05	0.000645	893	3146	1934	231	481	266
Solyc03g113240.2.1	1.724472	5.136587	0.000212	0.00171	3422	4105	2782	934	566	311
Solyc01g097160.2.1	1.726637	3.120942	0.000768	0.005085	626	1132	877	215	118	101
Solyc08g048550.2.1	1.728928	4.061773	0.000213	0.001718	1538	1952	1422	416	230	190
Solyc11g072710.1.1	1.729508	7.202943	0.000213	0.001718	15987	17407	9537	2518	3094	2010
Solyc11g068950.1.1	1.733394	7.581241	0.000183	0.001511	10310	30451	20431	2260	5386	2633
Solyc06g009630.1.1	1.737084	6.74895	0.000157	0.001322	9040	11587	10969	2376	1807	1273
Solyc08g076310.2.1	1.738159	2.679584	0.001014	0.006413	280	1108	695	99	170	72
Solyc03g116560.1.1	1.740636	3.021634	0.000267	0.002084	570	1220	737	103	217	109
Solyc04g077010.2.1	1.7411	6.093898	3.69E-05	0.000385	5687	10288	5175	937	1717	938
Solyc10g078920.1.1	1.741329	5.223123	0.000895	0.005779	4784	2686	2622	935	605	361
Solyc01g108300.2.1	1.742553	5.830643	3.03E-05	0.000323	4152	8008	5400	797	1617	658
Solyc03g005000.2.1	1.742571	4.757834	1.75E-05	0.000199	2284	3402	2433	569	484	322
Solyc05g008040.2.1	1.742932	2.999986	0.000816	0.005343	315	1427	888	141	196	85
Solyc09g083050.2.1	1.743385	4.024195	0.000728	0.004864	576	4033	1180	185	406	254
Solyc01g107330.2.1	1.743438	3.846125	0.000585	0.004027	665	2441	1565	108	449	217
Solyc01g106820.2.1	1.743983	3.359858	0.001007	0.006377	906	1667	627	272	132	105
Solyc02g067660.2.1	1.746261	3.659677	5.57E-05	0.000548	1026	1840	1023	232	308	128
Solyc06g068140.2.1	1.747866	6.944945	0.000139	0.001197	11530	16901	8860	2655	2455	1262
Solyc09g072900.2.1	1.748496	3.509407	0.000173	0.001437	822	1671	1040	260	233	99
Solyc09g063010.2.1	1.749304	3.735807	0.000871	0.005653	1735	1443	588	256	299	140
Solyc08g082120.2.1	1.751236	6.78111	0.000264	0.002061	4176	20642	11968	1799	2241	1555
Solyc11g072540.1.1	1.751449	5.249008	6.15E-05	0.000597	3996	4286	2782	804	630	472
Solyc02g062140.2.1	1.751858	4.989677	0.000135	0.001164	3520	3630	2067	742	558	318
Solyc04g008690.2.1	1.753234	3.49774	6.11E-05	0.000594	859	1543	1052	175	221	171
Solyc12g014390.1.1	1.753272	5.013442	0.000268	0.002086	2767	3855	3004	912	436	270
Solyc12g070280.1.1	1.754812	2.764678	0.000722	0.00483	599	867	566	154	131	63
Solyc10g083490.1.1	1.755122	4.598372	8.53E-06	0.000106	1519	3535	2531	422	552	284
Solyc10g005530.2.1	1.755715	5.53353	5.31E-05	0.000526	2413	8187	4553	504	1289	656
Solyc07g045170.2.1	1.755872	2.363874	0.001394	0.008412	231	851	577	71	129	67
Solyc06g066030.2.1	1.756084	5.418999	7.98E-06	0.0001	3551	5115	4125	758	944	510
Solyc06g066340.2.1	1.756775	2.050746	0.001709	0.009971	203	694	435	66	99	49
Solyc02g086090.2.1	1.757112	3.363549	0.000132	0.001142	967	1302	798	140	257	138
Solyc11g068790.1.1	1.757265	3.966458	4.55E-05	0.000459	934	2365	1634	179	430	215
Solyc05g007120.2.1	1.757703	4.61509	1.04E-05	0.000126	1790	3519	2285	324	509	399
Solyc05g015050.2.1	1.759193	5.668887	0.001545	0.009179	6884	3999	2942	1318	731	491
Solyc09g011210.2.1	1.763218	2.666188	0.000552	0.00383	423	868	661	113	102	95
Solyc10g075100.1.1	1.765447	9.047357	0.000282	0.002183	34900	89125	46079	9608	15528	3809
Solyc03g113030.2.1	1.766123	3.14563	0.000515	0.003611	451	1105	1167	138	150	144
Solyc03g119600.1.1	1.766742	3.846904	0.000109	0.000975	975	2437	1199	149	293	268
Solyc01g110020.2.1	1.767477	3.446719	0.000183	0.001506	661	1701	1095	112	248	188
Solyc04g050540.2.1	1.76904	3.918303	3.77E-05	0.000391	856	2285	1648	259	378	156
Solyc06g006000.2.1	1.77017	5.733177	5.47E-05	0.00054	4117	4912	6455	742	1183	777
Solyc04g016360.2.1	1.771068	5.2931	6.37E-05	0.000616	1677	6476	4620	450	966	596
Solyc04g079790.2.1	1.772008	5.194207	7.95E-06	0.0001	2585	4639	3948	688	682	465
Solyc01g094570.2.1	1.772246	3.23996	0.000114	0.00101	807	1271	789	137	230	120
Solyc12g098350.1.1	1.774367	4.033258	2.49E-05	0.000271	1555	1924	1362	301	335	194
Solyc06g069450.2.1	1.774824	2.589023	0.000541	0.003768	509	855	475	75	132	94
Solyc07g049230.1.1	1.775113	3.73906	5.82E-05	0.00057	810	2014	1392	274	226	162
Solyc11g069470.1.1	1.775569	4.808057	5.92E-06	7.78E-05	1971	4428	2472	368	741	355
Solyc06g007760.2.1	1.77565	4.27515	0.001033	0.006512	1867	1994	1758	582	205	156
Solyc01g080080.2.1	1.776671	3.731124	3.29E-05	0.000348	900	2025	1254	179	273	209
Solyc01g079360.2.1	1.778173	5.12124	2.04E-05	0.000228	2686	4685	3304	801	596	348
Solyc10g079840.1.1	1.778299	3.040117	0.00035	0.002616	810	990	629	98	220	109
Solyc08g083010.2.1	1.781221	4.484812	0.001412	0.008503	2878	1778	1486	620	279	197
Solyc02g093630.2.1	1.781348	6.99212	4.97E-05	0.000496	8180	17795	13757	1663	3402	1594
Solyc04g008730.2.1	1.781958	4.241006	4.04E-05	0.000415	1407	2084	2151	203	501	273
Solyc04g078810.2.1	1.782145	2.314473	0.001635	0.009595	251	664	630	60	150	54
Solyc04g080390.2.1	1.783612	4.341205	9.87E-06	0.000121	1749	2658	1739	379	390	246
Solyc08g081830.2.1	1.784853	3.252031	0.000154	0.0013	626	1376	973	106	242	139
Solyc01g105460.2.1	1.785311	4.502053	8.51E-06	0.000106	1452	3279	2376	343	596	247
Solyc03g026110.2.1	1.786751	4.801519	4.88E-06	6.57E-05	2032	4151	2550	379	574	432
Solyc03g117810.2.1	1.789227	5.513731	6.56E-06	8.50E-05	2949	6964	4560	667	1150	547
Solyc01g103110.2.1	1.789518	3.906454	1.28E-05	0.000151	1210	2061	1331	232	347	185
Solyc12g014150.1.1	1.79046	2.013182	0.001382	0.00835	311	450	434	77	82	43
Solyc01g080150.2.1	1.791633	3.901309	0.000494	0.003484	427	2427	2054	206	372	189
Solyc08g005860.2.1	1.792041	4.185874	2.53E-05	0.000275	874	2998	2040	289	399	231
Solyc08g068590.2.1	1.792174	3.42785	0.000372	0.002752	866	1387	1014	277	152	98
Solyc05g009920.2.1	1.794029	3.418982	0.000131	0.001139	751	1474	1083	223	156	137
Solyc12g005280.1.1	1.794132	2.584414	0.00099	0.006286	254	903	760	93	154	63
Solyc01g107430.2.1	1.795123	3.87943	0.000167	0.001395	1776	1567	871	246	301	188
Solyc08g043180.2.1	1.795502	4.41636	0.000147	0.001254	1483	2788	2297	563	334	165
Solyc08g081310.2.1	1.796716	5.426399	3.53E-06	4.93E-05	3046	6153	4226	725	953	504
Solyc07g006340.2.1	1.796892	3.573449	0.000135	0.001166	1211	1342	935	250	217	125
Solyc02g069030.2.1	1.798752	2.94655	0.000649	0.004408	822	924	520	168	154	67
Solyc01g006970.2.1	1.798883	4.69779	5.04E-06	6.76E-05	1699	4308	2341	343	641	341
Solyc09g092160.2.1	1.799748	3.904749	0.000677	0.004566	1727	1601	985	394	189	141
Solyc09g065130.2.1	1.800594	4.219682	0.000193	0.001581	701	3498	2062	190	444	299

Solyc10g085100.1.1	1.802773	3.957893	0.000118	0.001041	847	2470	1680	342	345	113
Solyc02g086150.2.1	1.803619	2.586587	0.000558	0.003866	342	886	666	85	108	96
Solyc01g087260.2.1	1.804825	5.945264	0.000535	0.003735	7349	6295	3988	1546	859	593
Solyc11g045100.1.1	1.804861	2.356287	0.000574	0.003962	326	766	517	72	117	67
Solyc01g096190.2.1	1.807773	4.219127	7.44E-06	9.47E-05	1380	2709	1728	239	466	243
Solyc05g052710.2.1	1.808494	4.571597	1.85E-05	0.000209	2201	2606	2209	465	389	300
Solyc07g042260.2.1	1.809167	4.262818	2.00E-05	0.000223	1952	2564	1272	305	429	232
Solyc12g005040.1.1	1.809844	2.997293	0.000574	0.003964	907	804	571	137	129	116
Solyc03g113620.2.1	1.810971	5.214006	1.70E-05	0.000194	3925	4596	2517	658	715	460
Solyc04g025040.1.1	1.811344	5.789496	1.08E-05	0.00013	3816	7803	5670	790	1161	786
Solyc07g063430.2.1	1.811876	7.09991	0.000494	0.003484	13789	18379	9345	3597	1835	1221
Solyc09g011830.2.1	1.815114	3.481559	6.06E-05	0.00059	588	1861	1180	174	232	146
Solyc08g082670.2.1	1.815847	4.405808	0.000209	0.001695	2716	1579	1515	385	419	246
Solyc11g012820.1.1	1.815905	2.972254	0.000147	0.001252	532	1187	755	98	188	107
Solyc09g056160.2.1	1.818116	4.817592	6.42E-05	0.000619	1065	5124	3254	355	755	340
Solyc01g079390.2.1	1.819033	5.493389	8.81E-06	0.000109	4292	5758	3543	807	853	553
Solyc12g014110.1.1	1.821481	4.295621	0.00049	0.003471	547	2933	2910	226	435	305
Solyc01g105410.2.1	1.823932	7.182732	3.43E-05	0.000361	11616	23816	10907	2120	3591	1680
Solyc07g063610.2.1	1.824708	2.640464	0.000686	0.004621	338	814	787	120	144	52
Solyc12g013890.1.1	1.825546	3.696935	0.000816	0.005346	1300	1508	1030	367	189	79
Solyc04g051780.2.1	1.825957	3.104211	0.000118	0.001045	619	1293	793	107	228	103
Solyc01g005330.2.1	1.827794	5.228768	6.40E-06	8.32E-05	2370	5740	3848	440	809	585
Solyc04g081210.2.1	1.828361	4.927735	2.32E-06	3.42E-05	2233	4449	2873	408	776	363
Solyc02g078860.2.1	1.830723	2.943316	0.000329	0.002493	517	1067	811	167	105	91
Solyc11g069510.1.1	1.830938	3.88101	8.38E-06	0.000104	1175	2018	1355	231	325	176
Solyc10g083380.1.1	1.831173	5.797159	0.000175	0.001451	5404	7955	3735	1214	1292	353
Solyc09g091010.2.1	1.831591	3.584914	2.87E-05	0.000307	1017	1471	1137	217	236	136
Solyc08g079180.2.1	1.831951	4.867705	7.07E-05	0.000673	1130	5990	2916	325	726	411
Solyc01g097820.2.1	1.832773	1.862438	0.001466	0.008776	247	575	329	54	104	30
Solyc02g005210.2.1	1.832975	4.765685	2.00E-06	3.03E-05	1983	3867	2656	399	554	375
Solyc03g117600.2.1	1.833708	5.647424	6.32E-06	8.23E-05	3229	7009	5509	839	1143	536
Solyc10g086220.1.1	1.834111	5.619292	1.63E-05	0.000187	2546	8200	5327	595	1109	718
Solyc02g085030.2.1	1.836207	2.868002	0.000557	0.003857	324	1049	954	141	118	89
Solyc11g065930.1.1	1.836765	7.463344	0.00105	0.0066	4170	27864	26348	2520	3816	2356
Solyc09g098580.2.1	1.840147	4.493016	8.72E-06	0.000108	1849	3261	1908	464	396	234
Solyc12g015640.1.1	1.840885	3.36572	4.19E-05	0.000429	861	1501	843	175	201	125
Solyc12g099160.1.1	1.842116	4.252508	0.000112	0.001	1195	2731	2091	142	414	356
Solyc09g082810.2.1	1.846838	5.481738	0.000319	0.002424	1205	9006	5252	522	957	686
Solyc06g075360.2.1	1.848361	3.491303	0.000158	0.00133	520	1835	1318	113	292	156
Solyc10g084250.1.1	1.851929	2.638663	0.000401	0.002929	346	855	755	79	122	95
Solyc02g092610.2.1	1.851963	2.549759	0.001236	0.007585	429	688	655	40	139	103
Solyc02g092920.2.1	1.854731	5.279274	7.73E-05	0.000725	1953	6346	4395	331	754	735
Solyc06g069630.2.1	1.855326	2.881887	0.000946	0.00605	759	791	606	182	86	74
Solyc01g081490.2.1	1.856738	4.289968	1.09E-05	0.000131	1502	2637	1923	404	311	215
Solyc03g083090.2.1	1.856858	6.253034	1.04E-05	0.000126	5722	10286	7702	1075	1495	1087
Solyc08g066140.2.1	1.857271	3.821064	8.07E-06	0.000101	953	2023	1475	215	306	170
Solyc09g091240.2.1	1.858452	4.336041	4.54E-05	0.000459	1349	2535	2357	473	305	185
Solyc08g005800.2.1	1.858541	6.007742	5.75E-05	0.000564	3162	7979	9010	1088	1425	672
Solyc06g009790.2.1	1.859484	2.939325	0.000408	0.002974	484	1123	820	59	204	116
Solyc10g083350.1.1	1.859511	2.929864	0.000118	0.00104	541	989	821	135	156	80
Solyc05g051460.2.1	1.859535	4.854937	0.000881	0.005703	689	4972	4073	231	810	413
Solyc05g056370.2.1	1.859608	5.644895	2.17E-05	0.000239	5536	5609	3571	851	1027	564
Solyc04g071700.2.1	1.8609	2.857486	0.001346	0.008165	709	849	598	195	79	62
Solyc04g049630.2.1	1.860901	3.740816	2.24E-05	0.000246	713	2045	1546	185	301	168
Solyc03g112940.2.1	1.861435	3.461699	4.04E-05	0.000416	690	1927	991	200	231	109
Solyc04g015850.2.1	1.862147	2.774291	0.000343	0.002578	613	833	614	145	126	60
Solyc10g081310.1.1	1.862418	5.526243	5.00E-05	0.000499	1987	7951	5376	491	959	748
Solyc09g074100.2.1	1.862966	4.328758	1.41E-05	0.000165	1941	2794	1434	398	361	208
Solyc06g051900.2.1	1.864235	3.242796	6.66E-05	0.000638	767	1286	870	158	153	131
Solyc07g061900.2.1	1.864863	5.391001	6.32E-06	8.23E-05	3216	5457	4290	870	699	435
Solyc11g008540.1.1	1.865876	4.734542	6.93E-05	0.00066	3026	2626	2029	578	391	284
Solyc05g052030.1.1	1.866916	6.157322	9.13E-06	0.000113	6180	9747	6137	949	1551	961
Solyc03g078080.2.1	1.868108	4.440935	0.000722	0.00483	2219	2443	1773	638	203	162
Solyc04g007000.1.1	1.868305	6.188177	0.000129	0.001126	7252	9938	5124	1692	1336	535
Solyc11g045250.1.1	1.868597	2.84365	0.000745	0.004953	261	1329	829	73	168	106
Solyc10g007320.2.1	1.868935	3.814456	0.000191	0.001564	480	2161	1961	195	261	207
Solyc06g048950.2.1	1.869386	2.936828	0.000116	0.001027	680	881	731	123	142	98
Solyc06g065260.2.1	1.871254	5.170264	2.26E-06	3.34E-05	3372	4952	2759	551	746	443
Solyc09g065300.2.1	1.871406	3.305831	6.26E-05	0.000606	708	1332	1036	192	191	95
Solyc04g080470.2.1	1.871794	3.013267	0.000153	0.001292	635	1053	795	80	202	113
Solyc02g079360.1.1	1.873529	2.505521	0.000793	0.005227	514	653	528	132	87	49
Solyc06g050760.1.1	1.875249	3.816459	0.000103	0.00093	1446	1540	1170	322	208	133
Solyc01g099260.2.1	1.879366	4.835707	1.96E-06	2.98E-05	1971	4200	2892	336	641	400
Solyc01g109100.2.1	1.880379	2.360254	0.001146	0.007128	449	576	507	103	124	30
Solyc02g070390.2.1	1.881106	3.835198	8.30E-05	0.000772	722	2364	1597	122	382	200
Solyc09g009940.2.1	1.881292	6.705284	0.001559	0.00924	12600	9988	7336	3017	1111	718
Solyc04g077130.1.1	1.884374	3.156942	0.000208	0.001684	403	1255	1195	112	195	120
Solyc03g117430.2.1	1.884782	7.607935	0.000367	0.002726	26305	16291	11862	3322	3837	2200
Solyc02g092530.2.1	1.884897	4.488988	3.92E-06	5.41E-05	1599	3574	2042	412	348	282
Solyc03g078610.2.1	1.887328	3.463912	0.000273	0.002126	1379	1107	677	212	180	124
Solyc03g083200.2.1	1.887642	1.776866	0.000967	0.006161	199	553	351	52	72	39
Solyc04g078620.2.1	1.889855	2.540231	0.000732	0.00488	261	989	669	54	140	85
Solyc02g086860.2.1	1.891552	3.371585	0.000334	0.002524	361	1856	1269	109	225	156
Solyc05g056520.2.1	1.891762	1.711011	0.001314	0.007989	252	516	267	32	88	40

Solyc06g053910.2.1	1.892494	4.889542	3.77E-05	0.000391	1572	5353	2974	237	866	382
Solyc01g104580.2.1	1.893655	3.197535	0.00015	0.001277	449	1404	1118	101	203	132
Solyc12g036140.1.1	1.89412	2.817787	0.000731	0.004874	751	750	564	168	103	58
Solyc11g008620.1.1	1.896569	5.351001	9.26E-05	0.000846	4996	3707	2931	871	728	349
Solyc12g008870.1.1	1.897092	3.171216	0.000498	0.003503	391	1574	1036	68	224	140
Solyc08g080200.2.1	1.897731	5.345857	1.20E-06	1.94E-05	3661	5364	3491	592	844	503
Solyc03g044150.2.1	1.897769	5.721295	3.16E-06	4.47E-05	4223	6473	5494	744	1120	658
Solyc01g098990.2.1	1.897787	3.973081	5.70E-06	7.54E-05	1360	1839	1554	225	302	210
Solyc04g009530.2.1	1.898653	3.198285	0.000249	0.001963	494	1428	1050	205	123	93
Solyc11g008550.1.1	1.89911	3.186059	4.02E-05	0.000414	629	1515	806	137	184	110
Solyc03g119920.1.1	1.900845	3.530804	1.13E-05	0.000135	944	1621	1042	165	256	134
Solyc11g008150.1.1	1.901376	5.861629	4.28E-06	5.85E-05	4408	8728	5356	998	1164	617
Solyc08g079750.2.1	1.901415	2.42723	0.001093	0.006834	189	1125	550	90	91	67
Solyc10g011690.2.1	1.901944	3.521574	1.16E-05	0.000139	790	1780	1111	160	258	134
Solyc08g082250.2.1	1.903409	3.891624	2.06E-05	0.00023	1468	1659	1295	200	248	229
Solyc09g008940.2.1	1.904507	3.283183	0.000103	0.00093	917	1123	880	195	139	111
Solyc06g075010.2.1	1.905021	6.230215	0.000242	0.001914	2143	13031	10206	986	1619	971
Solyc03g113280.2.1	1.90665	4.064423	0.000166	0.001393	1466	1898	1680	412	159	175
Solyc01g068260.2.1	1.907785	2.438689	0.000252	0.001982	321	855	573	76	112	67
Solyc04g009730.2.1	1.908298	2.340461	0.000296	0.002272	372	826	428	64	115	62
Solyc04g017700.2.1	1.910144	3.752391	0.001415	0.008516	2083	777	715	262	210	149
Solyc02g064950.2.1	1.911133	4.88247	2.66E-06	3.85E-05	1913	4193	3272	317	743	372
Solyc02g067540.2.1	1.911514	3.999267	6.74E-06	8.68E-05	877	2600	1757	238	291	212
Solyc01g090740.2.1	1.912834	2.755931	0.000371	0.002744	290	1281	717	76	144	96
Solyc10g007600.2.1	1.913371	5.503985	0.000108	0.000962	2220	4454	7212	743	620	667
Solyc12g016220.1.1	1.915543	2.657884	0.000172	0.001428	359	1059	649	81	143	76
Solyc07g005510.2.1	1.916388	4.808618	9.13E-05	0.000836	3692	2616	1662	529	461	307
Solyc08g065800.1.1	1.919638	2.642216	0.000668	0.004513	334	953	730	49	196	68
Solyc01g067890.2.1	1.919862	6.17739	0.000804	0.005288	9038	7830	4254	1927	995	447
Solyc03g097190.2.1	1.920461	5.305819	1.25E-06	2.00E-05	2426	5857	4431	513	884	486
Solyc12g095760.1.1	1.920894	5.665811	0.00049	0.003471	1486	8806	6888	360	1290	777
Solyc01g094500.2.1	1.922466	2.545288	0.000147	0.00125	453	773	583	89	115	68
Solyc03g116790.2.1	1.922725	3.770276	1.43E-05	0.000166	822	1751	1711	212	310	134
Solyc07g005190.2.1	1.923198	2.561814	0.000368	0.00273	334	854	697	102	79	81
Solyc08g005310.2.1	1.923315	3.954362	3.97E-06	5.47E-05	1027	2397	1570	190	325	210
Solyc10g011660.2.1	1.923344	5.653402	1.05E-05	0.000127	5347	7126	3055	758	1108	540
Solyc08g063090.2.1	1.924121	2.23057	0.000755	0.005011	348	700	437	80	123	29
Solyc09g061860.2.1	1.924526	4.823578	0.00022	0.001766	2030	5590	1919	764	365	177
Solyc05g013160.2.1	1.924792	4.303783	1.54E-06	2.40E-05	1384	2909	2004	314	357	242
Solyc04g082040.2.1	1.924985	3.024886	0.000167	0.001397	800	710	846	127	182	80
Solyc08g006530.2.1	1.925801	4.45391	2.67E-06	3.87E-05	1553	3442	2070	377	502	183
Solyc09g009420.1.1	1.925815	4.166967	7.45E-05	0.000704	2094	1384	1593	283	449	149
Solyc03g098630.2.1	1.926157	4.898188	3.37E-06	4.73E-05	2734	4100	2423	589	521	282
Solyc09g013090.2.1	1.92685	3.338329	3.08E-05	0.000327	940	1433	770	135	191	139
Solyc01g097910.2.1	1.927132	3.51861	0.000724	0.004843	1569	1029	630	257	154	109
Solyc01g006930.2.1	1.9295	3.986971	0.001251	0.007666	491	3369	1719	74	439	249
Solyc07g061970.2.1	1.930826	4.396136	2.08E-06	3.12E-05	1247	3208	2358	347	397	236
Solyc01g091480.2.1	1.931094	3.174695	6.17E-05	0.000598	478	1504	992	124	210	97
Solyc07g062740.2.1	1.932635	2.435332	0.000556	0.003854	622	597	368	84	126	49
Solyc08g067210.2.1	1.934191	4.006006	9.58E-06	0.000118	849	2879	1651	190	405	180
Solyc06g083690.2.1	1.934511	4.909674	5.98E-05	0.000584	2444	3275	3385	735	343	270
Solyc05g009820.2.1	1.935041	2.942962	0.000481	0.003419	763	617	827	166	135	58
Solyc03g032040.2.1	1.935433	1.711009	0.001037	0.006531	174	592	320	38	70	43
Solyc01g107800.2.1	1.935442	2.987231	0.000132	0.001146	443	1473	745	84	211	89
Solyc03g005340.2.1	1.937117	4.807491	1.49E-06	2.35E-05	1590	4141	3313	410	595	314
Solyc08g082000.2.1	1.937199	2.357937	0.000377	0.002783	299	757	586	56	130	59
Solyc01g087390.2.1	1.93818	2.21082	0.000354	0.002635	315	764	421	75	102	42
Solyc11g065820.1.1	1.938319	2.640211	0.001291	0.007873	217	1212	711	45	165	87
Solyc04g082740.2.1	1.938335	2.482777	0.000296	0.002276	397	896	508	90	141	41
Solyc10g009410.1.1	1.940246	5.281487	4.01E-06	5.52E-05	4126	5149	2620	512	932	420
Solyc11g020780.1.1	1.943862	5.069417	7.06E-06	9.03E-05	2225	5624	3171	279	937	416
Solyc03g118510.2.1	1.944309	2.871011	0.001021	0.006454	313	1001	1035	47	162	125
Solyc03g025320.2.1	1.945251	5.036513	1.76E-05	0.0002	3217	4549	2413	731	549	245
Solyc12g013920.1.1	1.945773	3.510337	0.000227	0.001813	445	2304	1199	92	283	162
Solyc09g082630.2.1	1.946731	3.790688	9.22E-06	0.000114	816	2411	1366	237	293	124
Solyc12g019100.1.1	1.948032	1.533752	0.001631	0.009578	161	417	342	54	39	34
Solyc04g011670.2.1	1.948844	3.272098	0.000143	0.001225	434	1611	1163	94	198	148
Solyc01g107020.2.1	1.948863	1.902232	0.000742	0.004938	294	513	356	75	62	33
Solyc09g091590.2.1	1.949817	2.093366	0.000808	0.005306	195	713	501	43	101	55
Solyc05g018700.2.1	1.949874	5.782585	0.000235	0.00187	1674	9487	7496	491	1234	825
Solyc02g083680.1.1	1.949874	2.679623	0.000198	0.001613	304	1054	756	92	128	75
Solyc07g047950.1.1	1.950564	3.978272	6.86E-06	8.81E-05	1417	1915	1484	226	237	233
Solyc07g061990.2.1	1.951183	3.823303	6.48E-06	8.40E-05	1405	1831	1098	196	277	177
Solyc07g062520.2.1	1.951477	3.11353	0.000206	0.001672	417	1425	1019	76	238	103
Solyc07g061780.2.1	1.951695	4.6616	9.24E-06	0.000114	1331	4134	2890	234	615	343
Solyc11g069330.1.1	1.953575	5.658772	0.000167	0.001395	1992	10077	5450	461	1691	421
Solyc12g089000.1.1	1.953936	3.200418	0.000174	0.001445	476	1565	1010	73	245	120
Solyc08g068100.2.1	1.955479	3.550875	9.41E-05	0.000857	1038	1703	944	262	128	123
Solyc02g084720.2.1	1.956757	4.453716	5.65E-05	0.000555	743	3636	2976	244	544	254
Solyc05g054240.2.1	1.958407	3.287562	8.52E-05	0.000788	472	1649	1126	102	258	108
Solyc07g045180.2.1	1.959241	2.448147	0.000971	0.006183	269	892	636	46	182	46
Solyc03g112150.1.1	1.961253	5.11571	3.63E-07	6.75E-06	2369	5434	3448	512	734	371
Solyc11g011440.1.1	1.962321	5.41931	0.000304	0.002323	1152	11205	3592	430	1120	498
Solyc07g041340.1.1	1.96276	2.649256	0.000295	0.002269	731	686	428	98	109	75

Solyc04g072400.2.1	1.962906	5.129332	5.82E-05	0.00057	1333	6409	4206	286	770	545
Solyc01g080090.2.1	1.964115	2.13755	0.000426	0.003091	352	564	443	50	119	42
Solyc02g083970.1.1	1.964518	3.24124	0.000301	0.002305	675	1294	1015	53	226	155
Solyc01g095470.2.1	1.965363	2.629247	0.001146	0.007128	204	1668	429	85	128	71
Solyc03g118290.2.1	1.969748	7.147988	1.74E-05	0.000199	8202	24456	14623	1590	3667	1527
Solyc08g067340.2.1	1.971382	5.302395	0.000694	0.004661	1621	9752	3003	175	1179	548
Solyc08g078010.2.1	1.973712	5.23041	2.68E-06	3.87E-05	3042	4648	3948	706	513	433
Solyc06g065690.2.1	1.974056	3.617396	2.13E-05	0.000236	591	1982	1487	182	274	118
Solyc02g094060.2.1	1.97485	2.841301	7.72E-05	0.000725	513	1001	754	132	114	74
Solyc01g104710.2.1	1.979062	4.500938	1.03E-06	1.69E-05	1464	3554	2361	332	366	302
Solyc01g108530.2.1	1.979375	3.409172	0.0003	0.002297	1417	958	649	206	159	107
Solyc07g064330.2.1	1.981014	5.211955	6.12E-07	1.07E-05	2444	6358	3476	598	749	362
Solyc10g049210.1.1	1.9812	3.076732	5.75E-05	0.000564	678	1052	881	151	119	99
Solyc06g084470.2.1	1.98326	2.077523	0.000651	0.004418	419	407	412	79	64	43
Solyc01g105010.2.1	1.983702	2.675379	0.000739	0.004918	196	1089	871	91	155	55
Solyc06g065390.2.1	1.983743	5.426257	2.66E-06	3.86E-05	4296	4904	3803	754	652	495
Solyc01g101240.2.1	1.984538	8.054805	8.79E-06	0.000109	20763	37326	26338	4688	4795	2603
Solyc06g048470.2.1	1.985112	3.16053	5.58E-05	0.000549	507	1221	1130	131	140	120
Solyc05g016330.2.1	1.987982	4.656526	1.20E-06	1.94E-05	1962	3496	2526	469	442	231
Solyc10g085340.1.1	1.99085	2.6399	0.000106	0.000953	603	791	515	100	120	62
Solyc09g065270.2.1	1.992444	5.621888	5.99E-05	0.000585	5040	5281	4439	1150	542	444
Solyc03g095490.2.1	1.992751	3.584989	1.05E-05	0.000127	932	1283	1441	152	231	154
Solyc11g069170.1.1	1.995631	2.807729	9.17E-05	0.000839	363	1194	775	100	122	87
Solyc02g082260.2.1	1.996326	4.100222	0.000137	0.001185	959	4295	961	361	207	174
Solyc10g081100.1.1	1.996666	3.960534	1.49E-05	0.000173	1474	1642	1562	307	213	160
Solyc09g008240.2.1	1.997311	5.029922	2.45E-06	3.58E-05	1652	4587	4354	503	690	311
Solyc07g007670.2.1	1.998169	5.163047	2.11E-06	3.16E-05	3055	4725	3431	711	615	293
Solyc11g009050.1.1	1.998435	2.468984	0.000144	0.001229	323	819	646	83	103	61
Solyc07g032710.2.1	1.998954	2.345298	0.001619	0.009517	234	884	585	114	99	22
Solyc11g022400.1.1	2.002169	3.235699	1.92E-05	0.000215	617	1510	953	157	176	91
Solyc03g120530.2.1	2.002181	1.684933	0.001622	0.00953	110	542	423	42	69	34
Solyc02g092480.2.1	2.002394	3.441278	3.05E-05	0.000325	613	1685	1259	99	248	146
Solyc01g111320.2.1	2.002782	3.308438	0.000141	0.001206	340	1815	1241	119	220	113
Solyc03g113630.2.1	2.003631	4.19913	1.93E-06	2.95E-05	1247	2873	1865	310	392	147
Solyc05g006920.2.1	2.006865	4.343747	1.25E-05	0.000148	2015	2287	1843	159	498	274
Solyc09g007280.2.1	2.008087	3.922516	0.000126	0.001103	483	2956	1844	142	404	166
Solyc07g007270.2.1	2.008102	3.032065	0.000937	0.006009	556	1339	785	30	238	115
Solyc03g114030.2.1	2.008661	4.08827	1.47E-05	0.000171	665	3003	2117	219	353	196
Solyc10g086730.1.1	2.009501	5.128325	0.000301	0.002304	4933	3363	1743	804	490	253
Solyc02g092560.2.1	2.009806	6.181742	1.99E-05	0.000223	7906	9342	5018	1362	1030	769
Solyc08g082190.2.1	2.010087	9.557308	5.22E-05	0.000518	38230	139320	79586	15056	12302	6311
Solyc10g080190.1.1	2.010235	3.373846	7.00E-06	8.95E-05	786	1481	1036	136	197	127
Solyc03g097950.2.1	2.010299	4.210082	1.35E-05	0.000158	1506	2355	1918	408	237	160
Solyc01g105180.2.1	2.010434	3.256562	0.001364	0.008258	337	1939	1068	41	219	164
Solyc07g061740.2.1	2.014223	4.65528	0.001172	0.007263	3652	1952	1435	660	213	195
Solyc02g088490.2.1	2.014248	4.79014	3.86E-07	7.13E-06	1745	4672	2772	365	485	362
Solyc11g005080.1.1	2.015146	3.574741	5.30E-06	7.05E-05	993	1857	986	201	217	114
Solyc03g051810.2.1	2.01524	4.109717	4.32E-06	5.89E-05	1706	1867	1629	311	297	160
Solyc03g019880.2.1	2.015287	4.824257	8.92E-07	1.49E-05	2357	3862	2721	534	435	275
Solyc02g070770.2.1	2.015669	3.929025	2.20E-06	3.27E-05	1019	1973	1830	220	273	179
Solyc11g062350.1.1	2.015672	2.040401	0.000245	0.001934	283	679	381	52	89	44
Solyc08g007000.2.1	2.015921	2.308324	0.001332	0.008087	199	980	533	30	121	72
Solyc01g005210.2.1	2.017618	6.511198	2.01E-06	3.03E-05	6984	13960	8580	1410	1703	962
Solyc08g041710.2.1	2.017902	3.177851	0.000111	0.000993	357	1734	1032	116	217	85
Solyc10g044710.1.1	2.019745	2.349195	0.000295	0.002267	446	680	461	103	83	39
Solyc09g014760.2.1	2.020448	5.524843	3.94E-06	5.44E-05	3021	7043	4956	926	781	353
Solyc08g081210.2.1	2.020501	3.928512	2.16E-06	3.22E-05	990	2547	1502	168	348	176
Solyc09g007410.2.1	2.021891	2.317064	0.000286	0.002209	330	632	603	47	100	71
Solyc04g054190.2.1	2.022449	4.373042	1.95E-05	0.000219	2511	2821	1020	319	401	208
Solyc01g103230.2.1	2.022483	4.130147	1.54E-05	0.000178	673	3073	2215	220	327	223
Solyc07g017510.2.1	2.023174	8.012573	5.82E-05	0.00057	23839	35798	21704	5681	4186	1692
Solyc01g102350.2.1	2.023585	4.543568	7.46E-05	0.000704	1154	3903	2760	187	797	173
Solyc12g007230.1.1	2.024566	3.333204	0.000143	0.001225	528	1660	1166	91	325	76
Solyc09g064720.2.1	2.028635	4.607966	5.61E-07	9.94E-06	1623	3624	2665	415	417	242
Solyc11g005640.1.1	2.028939	1.958571	0.000607	0.004159	438	397	307	60	80	34
Solyc10g006030.2.1	2.030072	5.300843	0.000121	0.001065	4236	3851	3599	979	369	317
Solyc06g076270.2.1	2.030671	2.407805	0.000319	0.002424	219	1055	565	67	118	54
Solyc01g106580.2.1	2.031108	2.935904	0.00024	0.001905	314	1099	1093	69	159	108
Solyc03g116320.2.1	2.0346	3.314523	0.000503	0.003537	1206	1310	515	237	114	74
Solyc05g009080.2.1	2.036314	1.556403	0.00114	0.007095	265	509	178	56	44	26
Solyc01g087040.2.1	2.040622	4.062697	0.000333	0.002516	2241	1427	1104	382	155	158
Solyc08g008630.2.1	2.040892	4.323987	0.000237	0.001883	2606	1868	1325	483	239	138
Solyc10g081770.1.1	2.041117	2.458887	0.000655	0.00444	335	568	790	37	147	67
Solyc07g039380.2.1	2.041334	2.829213	3.32E-05	0.00035	645	900	657	93	146	76
Solyc02g071260.2.1	2.041397	4.162546	1.61E-06	2.51E-05	1606	2436	1593	327	304	156
Solyc08g065220.2.1	2.042396	7.14984	0.001115	0.006963	23690	8780	5862	2355	2426	1433
Solyc01g008230.2.1	2.042881	4.413574	4.17E-05	0.000427	936	3361	2813	147	467	322
Solyc11g072600.1.1	2.043474	4.277776	0.000596	0.004088	2872	1386	1123	452	229	147
Solyc01g067630.2.1	2.045988	2.060491	0.000341	0.002562	237	629	491	74	68	38
Solyc03g097670.2.1	2.046488	4.939023	8.84E-07	1.48E-05	1588	5426	3388	376	708	307
Solyc01g086810.2.1	2.048163	4.465983	4.74E-06	6.41E-05	1380	3691	2284	167	491	316
Solyc05g050630.2.1	2.048792	3.728389	1.49E-06	2.34E-05	926	2035	1356	165	246	164
Solyc09g090840.2.1	2.049765	3.80146	2.84E-06	4.08E-05	790	2099	1683	199	280	139
Solyc09g064430.2.1	2.051036	4.810285	1.69E-06	2.61E-05	2991	3365	2213	495	443	274

Solyc03g081260.2.1	2.051351	3.503857	5.87E-05	0.000574	1360	1075	883	107	283	125
Solyc03g112950.2.1	2.05139	4.304631	6.86E-07	1.18E-05	1155	3559	1976	270	351	234
Solyc07g007800.2.1	2.051769	2.239522	0.000421	0.003056	377	959	267	77	96	37
Solyc01g007890.1.1	2.052174	3.435015	5.39E-05	0.000533	675	1871	1071	71	250	156
Solyc01g091780.2.1	2.052805	3.648836	7.51E-06	9.54E-05	1139	1622	1152	181	161	176
Solyc10g079830.1.1	2.054781	2.822249	2.96E-05	0.000316	518	1061	705	79	147	84
Solyc02g083500.2.1	2.056607	3.882377	5.96E-06	7.83E-05	1046	1993	1668	278	187	154
Solyc01g080300.2.1	2.056695	2.435238	0.000122	0.001072	398	721	591	57	93	78
Solyc11g067050.1.1	2.058168	4.20188	4.19E-06	5.75E-05	1162	2793	2070	148	450	228
Solyc07g005870.2.1	2.058538	2.465528	0.000135	0.001166	338	867	606	100	95	45
Solyc01g097860.2.1	2.059673	4.840848	2.39E-06	3.51E-05	2814	3535	2507	557	477	219
Solyc08g079070.2.1	2.060126	5.688223	3.59E-07	6.70E-06	3533	7797	5503	725	914	589
Solyc04g077390.2.1	2.06173	5.549405	1.36E-05	0.000159	2839	9418	3971	324	1187	599
Solyc01g111930.2.1	2.062145	3.252225	0.000101	0.000915	950	1108	850	208	110	81
Solyc06g066430.2.1	2.062755	1.879641	0.000649	0.004408	249	446	454	28	75	54
Solyc02g071590.1.1	2.063327	2.122351	0.00094	0.00603	498	487	315	92	73	25
Solyc04g008370.1.1	2.066946	2.850905	0.000101	0.00091	305	1136	965	101	142	72
Solyc06g008300.2.1	2.068233	3.931729	0.000116	0.00103	977	2918	1320	73	458	177
Solyc02g080990.1.1	2.068306	2.723435	5.83E-05	0.000571	374	1030	771	100	102	76
Solyc09g011300.2.1	2.068508	3.601708	6.85E-06	8.80E-05	663	1927	1439	130	232	159
Solyc08g005490.2.1	2.068623	5.481696	2.02E-06	3.04E-05	4180	5326	4308	387	1076	535
Solyc03g005280.2.1	2.069309	3.465604	7.84E-05	0.000733	724	949	1672	199	145	117
Solyc07g009150.2.1	2.069597	2.641773	6.82E-05	0.000651	332	1105	671	77	130	66
Solyc04g009560.2.1	2.069873	3.997635	3.58E-06	4.99E-05	910	2477	1890	147	336	211
Solyc11g067060.1.1	2.070984	4.25816	9.46E-06	0.000116	1489	2540	2046	417	253	143
Solyc04g078610.2.1	2.07189	4.75351	3.02E-07	5.70E-06	1725	3635	3320	328	549	302
Solyc07g056580.2.1	2.073164	2.845298	4.34E-05	0.000441	420	1320	699	74	163	80
Solyc06g050370.1.1	2.073245	2.169846	0.000574	0.00396	197	748	564	79	91	29
Solyc10g079070.1.1	2.073407	4.857821	2.75E-06	3.97E-05	1693	4585	3340	227	665	383
Solyc06g030530.2.1	2.073556	3.56012	2.14E-06	3.19E-05	790	1891	1208	160	235	120
Solyc03g113270.2.1	2.073705	7.539347	3.64E-06	5.08E-05	11281	31256	19817	2420	3465	2145
Solyc08g079630.2.1	2.073845	2.670331	0.000205	0.00166	334	1099	709	46	132	94
Solyc08g079310.2.1	2.075927	3.132998	0.000828	0.005414	958	1122	618	233	76	53
Solyc04g078900.2.1	2.076139	2.996448	0.000293	0.002251	658	1318	637	53	272	59
Solyc08g065360.2.1	2.076648	3.751013	1.19E-06	1.93E-05	915	2219	1328	181	290	125
Solyc06g059800.2.1	2.076964	4.796032	1.31E-06	2.10E-05	2449	3912	2484	228	547	409
Solyc01g068290.2.1	2.077052	3.749056	3.27E-06	4.62E-05	1042	2014	1295	240	176	143
Solyc07g064970.2.1	2.077488	4.063072	1.51E-06	2.37E-05	994	2273	2135	185	343	198
Solyc06g068210.1.1	2.0783	2.564661	0.00013	0.001133	520	846	512	45	136	77
Solyc09g083350.2.1	2.078934	5.053823	5.44E-08	1.25E-06	2626	4979	3158	474	583	365
Solyc03g093800.1.1	2.079323	3.64375	4.23E-06	5.79E-05	958	2395	885	171	269	113
Solyc01g096450.2.1	2.080188	3.128509	0.000154	0.001299	678	737	1204	175	127	69
Solyc03g119080.2.1	2.080868	7.182588	4.96E-05	0.000496	7229	18807	21038	1174	3683	1673
Solyc08g077430.2.1	2.08096	4.839038	4.16E-07	7.61E-06	1833	5017	2791	276	594	368
Solyc02g093080.2.1	2.081397	3.539835	4.52E-05	0.000457	514	1944	1471	99	211	177
Solyc02g084020.2.1	2.081937	3.835137	5.45E-05	0.000538	789	2488	1549	81	393	172
Solyc07g049320.2.1	2.083589	1.493243	0.000896	0.00578	213	318	333	45	33	35
Solyc05g006970.2.1	2.083764	2.453893	0.000293	0.002251	340	841	613	36	150	61
Solyc04g077670.2.1	2.084881	1.940622	0.000882	0.005709	151	633	505	64	80	25
Solyc07g043160.1.1	2.086042	3.47747	1.68E-05	0.000193	1036	1422	1017	199	124	129
Solyc07g056640.1.1	2.086376	1.990889	0.000923	0.005944	404	366	402	78	38	43
Solyc08g061630.2.1	2.086961	2.831263	0.000134	0.001159	636	758	774	147	107	49
Solyc04g080140.2.1	2.087247	2.978646	3.28E-05	0.000348	403	1399	875	100	171	75
Solyc08g042050.2.1	2.089731	6.476399	1.28E-06	2.04E-05	5632	14053	9822	1110	1643	1048
Solyc03g113610.2.1	2.089966	3.178115	0.000105	0.00094	344	1549	1191	84	185	118
Solyc06g069280.2.1	2.090207	2.774252	3.71E-05	0.000386	465	1045	724	79	165	61
Solyc01g104410.2.1	2.091175	3.150755	3.09E-05	0.000329	591	1415	922	70	193	119
Solyc01g097340.2.1	2.09228	3.332552	0.000466	0.003329	1132	1001	862	256	107	56
Solyc04g071140.2.1	2.094763	8.255426	0.000569	0.003931	41913	28121	18607	7411	3263	2014
Solyc08g006760.2.1	2.095611	3.332245	0.000424	0.003073	1507	754	556	184	157	85
Solyc01g107200.2.1	2.09643	1.792736	0.000646	0.004393	259	453	379	58	33	45
Solyc06g034290.2.1	2.098042	5.192932	9.64E-07	1.59E-05	3908	4081	3140	365	787	433
Solyc11g068360.1.1	2.098377	2.094601	0.000213	0.001716	284	562	513	39	90	53
Solyc01g099180.2.1	2.099246	3.257799	2.98E-05	0.000318	615	978	1372	107	184	115
Solyc10g074970.1.1	2.100724	3.369639	6.67E-05	0.000639	362	1895	1324	113	184	135
Solyc03g019670.2.1	2.100973	3.61736	0.000139	0.001194	652	1945	1495	57	305	173
Solyc09g010400.2.1	2.10158	6.105275	2.93E-06	4.19E-05	6860	7873	6421	994	945	873
Solyc04g009540.1.1	2.101828	5.243357	1.42E-07	2.92E-06	2544	5360	4398	564	693	365
Solyc11g007990.1.1	2.102073	5.817792	3.15E-05	0.000334	3968	8468	6024	1304	742	334
Solyc11g010170.1.1	2.104924	3.659714	1.35E-06	2.15E-05	803	1951	1412	182	231	125
Solyc04g074050.2.1	2.105842	3.726552	0.000306	0.002336	594	3217	1038	60	332	185
Solyc03g119700.2.1	2.106409	4.343332	0.000219	0.001754	2991	1749	1052	400	207	209
Solyc10g006330.2.1	2.107078	2.780893	0.000273	0.002121	759	915	456	150	90	46
Solyc07g008670.2.1	2.107681	2.472418	0.000138	0.001191	578	622	483	90	70	64
Solyc02g092670.1.1	2.108	5.86088	0.000481	0.00342	3330	9183	6869	183	1934	606
Solyc03g078100.2.1	2.108499	2.010042	0.000287	0.002215	195	618	518	54	68	45
Solyc06g007680.1.1	2.108679	2.089168	0.000339	0.002551	262	530	558	35	105	46
Solyc03g005190.2.1	2.110074	3.563972	4.25E-05	0.000434	1218	1202	1143	241	192	72
Solyc06g083680.2.1	2.110092	5.894145	0.000248	0.001958	8121	4794	4122	1310	552	530
Solyc02g080320.2.1	2.110494	2.550845	0.000287	0.002212	232	1205	630	50	147	61
Solyc12g094380.1.1	2.112907	3.850591	2.83E-05	0.000304	1812	1253	1130	131	299	183
Solyc06g083890.2.1	2.114805	3.058324	2.44E-05	0.000265	432	1427	959	89	167	97
Solyc06g084460.2.1	2.115989	3.388897	0.00035	0.002618	360	1718	1486	238	128	67
Solyc01g068130.2.1	2.116141	2.360436	0.000251	0.001973	324	555	722	43	116	61

Solyc02g070490.2.1	2.116929	3.438949	5.29E-06	7.05E-05	1083	1390	908	173	186	98
Solyc08g083140.2.1	2.117543	1.56812	0.000762	0.005047	122	533	355	32	56	35
Solyc08g075280.2.1	2.128608	5.067411	2.27E-06	3.36E-05	3242	5216	2398	663	505	236
Solyc09g091580.2.1	2.12886	5.284106	0.000214	0.001721	3798	6093	2731	1046	371	175
Solyc01g095700.2.1	2.128872	5.321049	1.10E-07	2.34E-06	2971	5374	4508	412	816	466
Solyc10g076740.1.1	2.129365	2.943442	7.44E-05	0.000704	427	1467	759	54	189	90
Solyc10g083360.1.1	2.129866	5.482315	1.50E-07	3.04E-06	3238	7355	4293	641	920	369
Solyc01g079520.2.1	2.130148	2.314507	0.000166	0.001393	284	741	608	43	95	67
Solyc01g105430.2.1	2.132959	1.887784	0.000906	0.005843	447	497	193	64	72	22
Solyc12g010380.1.1	2.133022	3.736638	4.36E-05	0.000443	1646	1257	1025	264	174	107
Solyc05g009310.2.1	2.133588	3.902502	0.000677	0.004564	2192	724	1161	314	143	136
Solyc02g068930.2.1	2.133696	1.955064	0.000278	0.002157	185	666	460	49	82	34
Solyc03g098440.2.1	2.13509	3.281706	1.84E-05	0.000208	911	1201	922	171	110	105
Solyc01g105030.2.1	2.135331	4.739789	5.24E-05	0.000519	3672	2196	1814	516	314	246
Solyc12g035190.1.1	2.139362	1.76547	0.000878	0.005686	314	468	289	72	54	16
Solyc02g093140.2.1	2.140384	4.547907	3.61E-07	6.72E-06	2174	2829	2302	324	312	296
Solyc10g080490.1.1	2.141108	2.766955	0.000436	0.003154	861	619	508	146	73	53
Solyc04g057870.2.1	2.142168	4.960758	1.47E-07	3.00E-06	1890	4875	3611	495	495	295
Solyc08g023440.2.1	2.142333	4.079447	1.43E-05	0.000166	1168	2640	1782	376	220	100
Solyc06g050130.2.1	2.142492	2.601641	0.000147	0.00125	354	1038	654	114	111	33
Solyc02g085870.2.1	2.142618	5.564256	2.47E-06	3.60E-05	4367	7674	3473	358	1181	521
Solyc06g062580.2.1	2.144622	1.957437	0.001056	0.006626	476	237	378	68	55	34
Solyc02g080260.2.1	2.145428	2.055993	0.000341	0.002567	212	786	434	44	111	29
Solyc01g095180.2.1	2.146235	2.435207	8.59E-05	0.000794	277	992	586	55	106	63
Solyc09g065030.2.1	2.147957	3.860324	2.48E-05	0.00027	651	2252	1979	104	364	161
Solyc02g086050.2.1	2.148058	2.609868	4.97E-05	0.000496	337	1009	705	80	132	49
Solyc01g005830.2.1	2.148326	2.574991	0.001707	0.009968	221	1061	772	20	123	99
Solyc06g054010.2.1	2.148677	3.059363	0.000139	0.001195	285	1989	794	107	120	105
Solyc02g077670.2.1	2.149101	3.11793	9.59E-06	0.000118	561	1398	931	85	176	101
Solyc08g074390.2.1	2.149323	2.133306	0.000238	0.001894	219	770	502	37	84	58
Solyc08g005010.2.1	2.1495	6.129775	4.06E-06	5.59E-05	7166	9907	5215	1285	982	592
Solyc02g093930.2.1	2.150324	1.821253	0.00049	0.00347	235	613	334	72	50	22
Solyc09g090410.2.1	2.150831	4.445185	3.60E-05	0.000376	1999	2389	2340	505	188	165
Solyc09g005020.1.1	2.15127	1.083323	0.000988	0.006275	214	237	188	31	31	22
Solyc01g005540.2.1	2.152779	3.495835	1.51E-05	0.000175	655	1749	1350	83	211	164
Solyc10g044780.1.1	2.15291	2.408026	8.69E-05	0.000802	358	747	615	77	116	35
Solyc07g008900.2.1	2.153985	3.609933	2.46E-06	3.60E-05	685	1985	1435	170	248	100
Solyc08g005430.2.1	2.154049	2.867633	4.78E-05	0.00048	623	1018	695	49	169	90
Solyc08g079790.1.1	2.155382	2.025796	0.000725	0.004846	194	668	507	23	79	62
Solyc02g086830.2.1	2.155578	6.322911	2.21E-05	0.000243	8431	9395	6916	1697	927	596
Solyc09g082830.2.1	2.157735	5.008334	1.93E-06	2.94E-05	4059	3399	2186	445	507	353
Solyc03g119740.2.1	2.159343	3.597282	1.85E-06	2.83E-05	1245	1448	1055	140	243	123
Solyc01g080700.2.1	2.161043	1.451901	0.001028	0.00649	155	358	364	54	33	21
Solyc07g053600.2.1	2.161778	3.557319	1.55E-05	0.000179	852	2081	1043	77	314	122
Solyc02g089950.1.1	2.16295	1.632487	0.000294	0.00226	191	480	348	45	43	34
Solyc07g006230.1.1	2.163703	3.011665	1.34E-05	0.000158	583	1056	947	78	181	82
Solyc10g005920.2.1	2.164201	5.198063	6.61E-08	1.49E-06	2908	5646	3510	379	631	473
Solyc01g028830.2.1	2.164744	4.050197	7.56E-06	9.59E-05	1257	2172	1884	104	299	259
Solyc07g053710.2.1	2.16483	4.283378	1.30E-07	2.70E-06	1225	3071	2201	256	362	189
Solyc03g006600.1.1	2.165644	2.794265	6.34E-05	0.000613	296	1308	811	83	105	87
Solyc05g042120.2.1	2.166418	3.432472	5.53E-06	7.33E-05	628	1706	1275	98	202	139
Solyc06g048960.2.1	2.167308	3.280282	4.04E-06	5.56E-05	565	1624	1090	115	183	102
Solyc02g068050.2.1	2.167606	4.689476	9.35E-08	2.01E-06	2561	3410	2182	374	434	248
Solyc09g010360.2.1	2.169532	2.328061	6.53E-05	0.000628	320	795	550	79	77	46
Solyc06g083660.2.1	2.170127	3.566274	3.23E-06	4.56E-05	1172	1488	1048	153	153	155
Solyc09g011720.2.1	2.172734	2.131897	0.00087	0.00565	336	431	576	69	28	63
Solyc02g079020.2.1	2.173519	3.825059	4.66E-07	8.41E-06	915	2566	1362	155	256	165
Solyc05g005560.2.1	2.173538	3.379402	8.36E-06	0.000104	672	1332	1348	89	205	131
Solyc04g015480.2.1	2.174649	5.37078	7.73E-06	9.79E-05	4415	5023	3420	868	563	252
Solyc08g083350.2.1	2.175346	4.587335	4.06E-05	0.000417	2450	2544	2362	551	197	183
Solyc08g066120.2.1	2.175415	4.028111	2.72E-07	5.23E-06	1072	2634	1753	172	354	160
Solyc01g080460.2.1	2.176019	6.670032	3.92E-06	5.41E-05	6680	18808	9527	1016	2658	796
Solyc07g005390.2.1	2.177556	6.939228	0.000151	0.001278	10854	21509	8751	3022	1328	578
Solyc05g005760.2.1	2.177631	2.260589	0.000185	0.001527	392	448	617	80	64	45
Solyc09g011140.2.1	2.17802	3.86197	3.11E-07	5.86E-06	1042	2463	1381	208	271	123
Solyc01g094910.2.1	2.178095	6.947279	0.000809	0.005312	3728	22786	16975	408	2956	1785
Solyc03g117040.2.1	2.178355	6.525173	5.32E-07	9.49E-06	6109	15434	9543	1004	1777	1010
Solyc05g054340.2.1	2.178396	3.148777	0.000435	0.003144	385	1428	1194	34	212	123
Solyc03g096830.2.1	2.178583	3.05423	6.53E-05	0.000628	980	867	651	152	128	58
Solyc02g092310.2.1	2.182016	4.561417	0.00043	0.003116	3526	2151	1160	558	211	153
Solyc09g065690.2.1	2.184561	3.132878	3.81E-06	5.28E-05	645	1466	821	115	140	95
Solyc01g110820.2.1	2.184753	2.574477	0.000137	0.001185	364	1046	613	38	164	56
Solyc05g014310.2.1	2.187579	4.822815	5.78E-06	7.62E-05	3211	2990	2395	567	363	200
Solyc01g010190.2.1	2.188132	2.772379	0.000622	0.004246	292	1092	930	28	195	73
Solyc01g079800.2.1	2.190241	1.76204	0.00019	0.001559	221	510	381	35	76	30
Solyc10g086660.1.1	2.190697	3.443619	5.37E-06	7.13E-05	1069	1128	1133	117	250	93
Solyc09g014770.2.1	2.190772	6.544024	6.69E-07	1.16E-05	5590	16564	9864	1062	1827	951
Solyc12g055940.1.1	2.191704	3.438531	7.34E-06	9.36E-05	1030	1301	1059	199	142	88
Solyc09g083260.2.1	2.191881	1.599053	0.001623	0.00953	153	441	403	13	84	32
Solyc10g075020.1.1	2.193284	2.517958	0.001505	0.008977	484	771	574	156	39	25
Solyc03g044090.2.1	2.194175	1.967526	0.000359	0.002668	245	607	442	23	96	44
Solyc01g107690.2.1	2.194235	2.526078	0.000193	0.001581	245	823	847	62	144	41
Solyc05g056270.2.1	2.1949	3.498606	2.96E-06	4.23E-05	633	1915	1295	172	152	118
Solyc02g082480.2.1	2.196324	3.281417	9.28E-05	0.000847	306	1575	1455	111	223	77

Solyc07g008310.2.1	2.196428	3.411474	1.63E-06	2.52E-05	775	1396	1257	151	173	102
Solyc03g117250.2.1	2.197965	3.879565	3.84E-05	0.000398	685	1404	2560	144	223	204
Solyc04g064930.2.1	2.199724	3.282684	3.49E-06	4.89E-05	898	1325	885	150	168	77
Solyc06g016770.2.1	2.199806	3.126988	7.77E-05	0.000728	374	1784	948	59	173	118
Solyc03g058190.2.1	2.200598	5.838924	2.19E-07	4.31E-06	4041	9233	5897	866	979	480
Solyc12g099960.1.1	2.200869	2.12417	0.000648	0.004408	511	539	289	94	49	27
Solyc05g014110.2.1	2.201478	3.278176	3.82E-06	5.28E-05	736	1544	937	84	185	120
Solyc11g066050.1.1	2.204809	2.477175	7.32E-05	0.000694	251	915	726	68	107	51
Solyc02g078570.2.1	2.204817	0.740565	0.001048	0.006588	123	216	186	19	26	19
Solyc06g007770.2.1	2.204921	3.402069	7.78E-06	9.84E-05	526	1447	1516	144	190	94
Solyc01g007880.2.1	2.206773	2.964614	5.39E-06	7.17E-05	663	1117	745	115	121	73
Solyc01g010520.2.1	2.207224	4.867427	5.10E-08	1.18E-06	2059	4521	3129	272	616	311
Solyc01g008960.2.1	2.209189	7.690106	9.74E-07	1.61E-05	13870	34078	21772	2934	3769	1724
Solyc07g063690.1.1	2.209656	2.07529	0.000733	0.004883	247	398	664	86	43	32
Solyc06g053600.2.1	2.210355	3.680842	2.50E-06	3.65E-05	918	1383	1736	195	228	98
Solyc06g074460.2.1	2.210944	2.813523	2.94E-05	0.000315	394	1093	867	63	167	64
Solyc07g063960.2.1	2.211409	4.911087	5.53E-06	7.32E-05	2661	3617	3226	645	303	214
Solyc02g070440.2.1	2.211491	4.483508	3.38E-07	6.33E-06	1111	3847	2732	237	395	258
Solyc01g006830.2.1	2.213044	4.779376	5.00E-07	8.97E-06	2870	3504	2269	221	496	367
Solyc07g049500.2.1	2.215445	2.383583	0.00015	0.001277	223	1012	598	45	119	51
Solyc08g082180.2.1	2.215692	4.102712	2.00E-06	3.02E-05	886	2769	2175	133	342	214
Solyc02g094590.2.1	2.215931	2.740165	7.82E-05	0.000732	266	1312	784	62	151	63
Solyc09g008790.2.1	2.216292	3.110982	3.29E-06	4.63E-05	609	1546	786	111	149	84
Solyc06g007120.2.1	2.219762	3.240113	4.63E-06	6.28E-05	568	1660	1004	115	203	73
Solyc03g121180.2.1	2.219824	5.55819	7.42E-08	1.64E-06	4457	6086	4448	644	887	388
Solyc02g069130.2.1	2.22151	2.0313	0.000363	0.002693	350	426	484	75	72	19
Solyc01g111040.2.1	2.222389	5.261783	6.19E-08	1.40E-06	3147	5634	3788	630	598	300
Solyc01g107660.2.1	2.224413	5.088819	0.000197	0.001608	4210	2968	2895	846	249	194
Solyc11g072110.1.1	2.224696	1.825113	0.000281	0.002174	174	585	440	43	81	23
Solyc06g065990.1.1	2.224785	3.720524	0.000657	0.004451	1747	1125	985	338	96	66
Solyc09g097820.2.1	2.225335	4.015212	7.30E-05	0.000693	580	3284	1935	87	443	159
Solyc02g082690.2.1	2.225757	2.960363	2.84E-05	0.000305	364	1504	867	124	135	54
Solyc08g044260.2.1	2.225924	5.772087	2.56E-07	4.96E-06	4323	8835	5090	893	811	452
Solyc11g072480.1.1	2.226128	4.152742	2.18E-07	4.30E-06	1771	2171	1646	225	369	138
Solyc02g078160.2.1	2.226336	3.551639	1.04E-05	0.000126	1096	2247	648	202	169	90
Solyc10g085670.1.1	2.226666	1.730182	0.000455	0.003267	354	275	350	36	79	23
Solyc08g042140.2.1	2.228413	1.982315	0.0004	0.002924	299	622	382	85	47	21
Solyc02g080980.2.1	2.229674	2.889751	7.90E-06	9.95E-05	425	1221	863	91	117	80
Solyc02g089080.2.1	2.231832	3.149033	4.54E-06	6.17E-05	644	1302	971	142	124	78
Solyc06g071370.1.1	2.232031	1.652968	0.000356	0.002646	289	373	316	25	45	47
Solyc11g027830.1.1	2.233057	2.239404	0.001148	0.007139	463	573	438	112	25	35
Solyc09g065150.1.1	2.233158	0.805239	0.00161	0.009481	197	217	119	25	35	10
Solyc02g079190.2.1	2.233209	5.227026	2.43E-05	0.000265	4798	4251	2368	779	386	267
Solyc03g121120.2.1	2.233434	3.580609	5.77E-06	7.61E-05	721	2161	1241	82	290	123
Solyc02g086130.2.1	2.23521	4.677561	2.04E-07	4.04E-06	1458	4229	3027	225	458	319
Solyc04g045580.2.1	2.235588	2.832182	8.04E-06	0.000101	470	1142	774	106	107	64
Solyc05g018620.2.1	2.237607	4.106846	1.40E-05	0.000164	1590	2810	1305	376	202	92
Solyc02g068040.2.1	2.238316	1.563571	0.000934	0.005995	361	365	178	55	45	16
Solyc01g105020.2.1	2.239796	4.504421	0.000651	0.004418	3711	1505	1122	516	225	128
Solyc10g047050.1.1	2.239864	3.555453	2.79E-06	4.01E-05	541	2294	1320	136	207	120
Solyc09g005750.2.1	2.240446	3.225352	1.99E-06	3.02E-05	705	1401	977	95	156	111
Solyc02g069290.2.1	2.241135	5.991957	1.55E-06	2.41E-05	3471	11825	6926	505	1400	682
Solyc04g076660.2.1	2.24135	2.28075	0.000169	0.001408	234	857	582	33	105	56
Solyc12g005240.1.1	2.241739	2.487701	0.000196	0.001603	523	620	597	119	56	37
Solyc02g077310.2.1	2.241773	1.610009	0.000682	0.004595	178	521	332	18	44	50
Solyc12g021280.1.1	2.241952	3.779476	1.71E-07	3.45E-06	1181	2006	1294	163	202	160
Solyc11g071500.1.1	2.242574	0.629765	0.001306	0.00795	104	203	184	16	35	11
Solyc10g011770.2.1	2.242745	4.150018	2.37E-05	0.000259	2097	1623	1591	368	217	104
Solyc06g069030.2.1	2.243798	4.488451	1.19E-07	2.48E-06	1491	3500	2531	370	318	184
Solyc02g068270.2.1	2.245765	3.883984	4.55E-07	8.24E-06	1480	1696	1429	210	264	115
Solyc02g077090.2.1	2.246068	3.66892	3.95E-07	7.27E-06	887	2036	1343	117	255	135
Solyc03g122270.2.1	2.246812	5.197136	2.68E-05	0.000289	1144	6902	5000	236	834	418
Solyc07g005570.2.1	2.247269	4.490269	1.92E-08	4.95E-07	1643	3477	2368	295	335	235
Solyc10g054100.1.1	2.248973	3.06394	0.000194	0.001589	264	2296	663	123	159	55
Solyc03g117010.2.1	2.24956	3.813071	1.38E-06	2.19E-05	736	2590	1572	129	222	184
Solyc12g014420.1.1	2.249735	1.775317	0.000211	0.001704	224	618	324	24	67	42
Solyc02g079430.2.1	2.250208	4.167995	7.34E-07	1.26E-05	964	3730	1722	150	350	209
Solyc02g084450.2.1	2.25224	1.937928	0.000456	0.003272	120	813	449	38	78	36
Solyc10g085980.1.1	2.252989	2.72819	0.000456	0.003271	791	651	548	143	42	55
Solyc03g120910.2.1	2.253002	4.251643	5.25E-07	9.40E-06	2149	2143	1584	289	267	175
Solyc09g074640.2.1	2.254384	4.307001	2.04E-07	4.04E-06	1719	2566	2068	331	249	173
Solyc01g097290.2.1	2.256078	4.965975	1.68E-06	2.59E-05	1795	4763	3964	235	848	243
Solyc03g083560.1.1	2.256736	2.992908	0.000329	0.002492	900	780	726	29	149	125
Solyc05g005080.2.1	2.256974	5.179293	1.65E-08	4.33E-07	2625	5234	4106	508	513	363
Solyc02g085760.2.1	2.258491	6.211338	2.63E-07	5.08E-06	7245	10995	5905	1064	1170	651
Solyc02g093210.2.1	2.258501	2.792052	0.000383	0.002819	247	1136	1002	32	158	86
Solyc01g008790.2.1	2.260378	1.868657	9.43E-05	0.000859	206	595	430	41	64	36
Solyc03g114150.2.1	2.262379	7.758661	3.46E-05	0.000364	27850	22550	15144	4042	2266	1800
Solyc01g005520.2.1	2.263417	4.893031	4.59E-06	6.23E-05	4017	2952	1915	486	447	222
Solyc09g083030.1.1	2.263889	2.16715	6.18E-05	0.000599	248	683	571	49	88	40
Solyc08g078210.2.1	2.264526	3.657194	1.28E-05	0.000152	1587	1432	839	221	183	85
Solyc05g012580.1.1	2.264601	7.250737	1.53E-05	0.000177	16371	21303	11176	2985	2080	862
Solyc02g030210.2.1	2.265451	3.18261	1.93E-06	2.94E-05	585	1630	907	103	148	97
Solyc07g056020.2.1	2.265983	5.806342	7.34E-08	1.62E-06	4884	8629	4992	791	843	518

Solyc01g010200.2.1	2.266555	5.869133	6.61E-06	8.54E-05	2408	10506	7604	434	1305	614
Solyc05g051850.2.1	2.267778	6.24326	1.11E-05	0.000133	9471	9290	4747	554	1822	726
Solyc05g015150.2.1	2.27003	3.420513	1.67E-06	2.58E-05	901	1468	1104	102	252	84
Solyc11g067140.1.1	2.27039	1.639056	0.000339	0.002554	227	395	375	41	71	15
Solyc01g005550.2.1	2.27119	4.197242	2.68E-06	3.87E-05	648	3620	2306	217	322	175
Solyc08g066390.2.1	2.271803	1.167332	0.000388	0.002852	157	408	195	20	40	25
Solyc03g119860.2.1	2.272627	2.52405	0.000196	0.001597	746	474	463	103	72	42
Solyc02g068440.2.1	2.2733	2.356278	2.11E-05	0.000234	365	782	551	60	98	43
Solyc06g005560.2.1	2.274746	1.848658	0.000467	0.003335	159	443	572	27	61	47
Solyc01g106090.2.1	2.276476	3.779787	0.000115	0.001017	1658	1448	1077	318	108	80
Solyc09g082210.2.1	2.279393	4.366356	3.25E-05	0.000344	876	3587	2726	90	446	271
Solyc12g100030.1.1	2.279646	4.432029	1.51E-07	3.07E-06	1992	3211	1869	319	413	131
Solyc05g014260.2.1	2.280698	6.095456	4.90E-05	0.00049	4609	15009	4980	1424	1010	255
Solyc08g008670.2.1	2.281138	1.226715	0.000352	0.002627	162	329	268	33	28	24
Solyc08g076480.2.1	2.28118	5.578533	9.60E-08	2.06E-06	2954	7810	5513	747	700	383
Solyc01g096480.2.1	2.28168	3.394352	4.79E-05	0.000481	507	1922	1248	219	98	71
Solyc03g058160.2.1	2.282153	3.958177	4.06E-07	7.45E-06	1692	1554	1506	191	246	155
Solyc01g014320.2.1	2.282676	1.434851	0.000809	0.005313	93	437	392	41	38	22
Solyc12g014440.1.1	2.285077	4.516745	3.40E-06	4.77E-05	849	4211	3037	224	314	303
Solyc02g077880.2.1	2.28525	8.147456	3.14E-06	4.46E-05	31228	38897	20585	4560	3368	2612
Solyc02g079200.1.1	2.287072	3.801447	4.30E-06	5.88E-05	1250	1500	1616	265	161	96
Solyc12g099200.1.1	2.288509	4.978244	0.000125	0.001096	1420	5633	3988	88	798	383
Solyc03g111120.2.1	2.28863	1.274587	0.000643	0.004376	156	258	345	20	36	32
Solyc04g005060.2.1	2.288815	1.334339	0.000597	0.004097	240	405	178	45	38	13
Solyc04g081890.1.1	2.290437	1.58209	0.001357	0.008224	205	781	121	26	79	18
Solyc12g013840.1.1	2.291951	2.763237	7.37E-05	0.000698	704	1126	411	105	136	31
Solyc10g078440.1.1	2.292014	5.378349	1.99E-08	5.12E-07	3695	7030	3272	598	620	362
Solyc06g005750.2.1	2.292409	2.621739	0.000225	0.001801	232	1310	689	102	117	26
Solyc03g082560.2.1	2.29378	3.849001	1.12E-07	2.37E-06	1101	2071	1579	205	238	112
Solyc01g102810.2.1	2.293903	3.231847	2.02E-05	0.000225	517	1623	1098	56	164	131
Solyc07g064360.1.1	2.294424	0.874615	0.0008	0.005263	142	227	209	13	33	22
Solyc02g077280.2.1	2.294614	3.977546	3.36E-06	4.73E-05	878	2777	1803	99	255	228
Solyc03g120230.2.1	2.295181	2.587387	1.63E-05	0.000187	333	923	768	66	97	63
Solyc09g060110.2.1	2.297425	1.739874	0.000192	0.001576	233	338	473	47	45	31
Solyc11g040040.1.1	2.299343	2.896085	9.26E-06	0.000114	562	901	925	69	104	98
Solyc11g066300.1.1	2.300283	2.026792	7.41E-05	0.000701	246	597	510	63	57	32
Solyc05g009040.2.1	2.300472	1.871497	0.000824	0.005391	139	1055	225	42	42	46
Solyc06g071330.2.1	2.300738	1.984291	0.000371	0.002749	360	408	454	44	29	61
Solyc08g080250.2.1	2.301396	2.774811	0.000242	0.001915	303	1179	891	27	149	88
Solyc04g074860.2.1	2.303564	1.428659	0.000584	0.00402	129	412	362	17	49	34
Solyc08g068200.2.1	2.304917	3.639321	2.00E-06	3.03E-05	865	1919	1394	78	253	148
Solyc06g084140.2.1	2.30492	2.480493	0.000121	0.001064	661	674	397	68	132	27
Solyc02g090580.2.1	2.305132	1.929963	0.000413	0.003006	128	659	538	29	67	46
Solyc01g067800.2.1	2.305338	4.547182	3.71E-07	6.89E-06	1164	3645	3170	199	478	234
Solyc05g054350.2.1	2.305604	5.036889	1.31E-05	0.000155	4601	3732	1664	586	434	217
Solyc03g080180.2.1	2.307203	4.981322	9.43E-07	1.57E-05	1719	5210	3927	588	399	199
Solyc05g050500.1.1	2.308019	4.02947	2.47E-06	3.60E-05	1709	2259	1284	304	223	92
Solyc11g020670.1.1	2.308465	4.360603	7.07E-08	1.57E-06	1347	4158	1774	272	261	218
Solyc07g040950.2.1	2.311214	3.965821	7.01E-08	1.56E-06	1580	1962	1425	194	250	145
Solyc09g005260.2.1	2.311468	8.803702	6.50E-07	1.13E-05	46321	56341	39603	5713	7847	3653
Solyc12g100160.1.1	2.312299	5.522825	9.86E-08	2.11E-06	4154	5468	5030	728	565	400
Solyc03g083610.2.1	2.314708	3.854313	1.84E-06	2.83E-05	770	2070	2016	108	313	144
Solyc01g005030.2.1	2.31473	4.679284	4.22E-06	5.78E-05	1171	4516	3281	142	563	292
Solyc01g100190.2.1	2.315929	2.376468	0.000265	0.002072	209	996	636	26	119	57
Solyc04g081370.2.1	2.317289	4.333608	7.86E-07	1.34E-05	1689	3358	1755	120	487	196
Solyc12g007010.1.1	2.317322	5.391232	0.00022	0.001766	6362	3714	2226	946	395	202
Solyc05g006000.2.1	2.317358	2.160857	4.07E-05	0.000418	313	523	595	49	72	45
Solyc08g077880.2.1	2.318534	6.075939	1.05E-07	2.23E-06	4242	12454	6877	902	936	654
Solyc07g006220.1.1	2.31915	5.381345	2.20E-06	3.28E-05	1506	8562	5075	380	922	348
Solyc12g096030.1.1	2.320379	2.957473	4.29E-05	0.000437	294	1381	1052	70	102	105
Solyc02g068090.2.1	2.322883	6.485062	7.55E-05	0.00071	10646	8225	8229	1922	620	633
Solyc03g111600.2.1	2.323064	2.505286	2.05E-05	0.000229	347	845	707	85	70	51
Solyc08g082680.2.1	2.323563	1.964508	0.00043	0.003114	429	414	352	27	39	66
Solyc02g077050.2.1	2.323935	3.531379	7.67E-06	9.71E-05	1152	1870	812	106	306	64
Solyc06g068480.2.1	2.324121	4.422152	5.59E-05	0.00055	2385	2301	1950	500	192	92
Solyc03g083570.2.1	2.324622	3.31735	3.79E-07	7.03E-06	815	1585	935	119	174	85
Solyc05g013720.2.1	2.324887	4.894589	1.94E-08	4.99E-07	2006	5598	2795	263	609	280
Solyc09g007940.2.1	2.326163	1.376137	0.001169	0.007249	79	376	420	39	29	24
Solyc01g088100.2.1	2.327006	6.407059	0.000209	0.001691	3120	16716	10761	281	2382	809
Solyc07g056040.2.1	2.328239	3.545561	1.76E-07	3.53E-06	778	1931	1270	147	175	110
Solyc03g005230.2.1	2.328572	7.406981	0.000368	0.002728	23917	16112	10656	3982	1315	826
Solyc12g005030.1.1	2.328604	3.213374	6.58E-07	1.14E-05	779	1331	936	118	144	83
Solyc01g111660.2.1	2.328915	2.694404	0.000266	0.002074	759	429	701	118	50	58
Solyc09g065920.2.1	2.329493	2.397266	0.00067	0.004527	169	1183	591	22	133	53
Solyc09g065480.1.1	2.330084	2.601229	1.77E-05	0.000201	433	1031	599	84	59	69
Solyc08g081080.2.1	2.330882	3.425723	2.67E-05	0.000289	453	1918	1405	60	250	110
Solyc09g018510.2.1	2.331394	4.437445	8.20E-07	1.39E-05	2031	2561	2296	402	223	158
Solyc09g005230.2.1	2.331729	2.408335	9.19E-05	0.00084	472	645	572	104	53	34
Solyc03g097650.2.1	2.331886	0.810193	0.001709	0.009969	124	212	220	15	49	7
Solyc04g077320.1.1	2.333927	0.990755	0.000955	0.006102	135	338	194	12	25	31
Solyc10g005790.2.1	2.334431	3.17723	5.11E-06	6.84E-05	866	1020	959	100	99	117
Solyc11g066820.1.1	2.335278	3.821399	2.53E-06	3.69E-05	829	2342	1683	90	353	121
Solyc05g051740.2.1	2.337595	1.129237	0.000811	0.00532	270	178	191	32	30	17
Solyc09g092270.2.1	2.338447	4.685735	0.000539	0.003756	2977	3858	1517	682	153	83

Solyc09g064520.2.1	2.338525	1.117677	0.000274	0.002126	179	299	220	21	31	25
Solyc01g067480.2.1	2.340378	2.797433	6.45E-06	8.38E-05	431	1173	777	91	80	75
Solyc01g065750.2.1	2.340567	0.763855	0.001029	0.006493	97	264	203	29	22	10
Solyc06g009400.2.1	2.341651	5.975322	2.60E-07	5.04E-06	3922	9485	7861	954	735	574
Solyc06g076570.1.1	2.342659	3.124685	2.56E-06	3.73E-05	814	1053	909	127	108	79
Solyc01g110340.2.1	2.342782	2.48298	0.000346	0.002591	804	470	360	89	101	25
Solyc07g025510.2.1	2.344577	1.420286	0.000191	0.00157	155	397	339	33	48	19
Solyc05g013460.2.1	2.345307	3.923223	7.34E-08	1.62E-06	904	2451	1833	192	238	132
Solyc02g081480.2.1	2.346421	1.85286	7.54E-05	0.00071	282	593	337	37	82	22
Solyc01g097520.2.1	2.347835	4.675906	6.65E-06	8.58E-05	700	4740	3733	303	458	206
Solyc02g088100.2.1	2.347851	1.877769	0.000228	0.001818	397	471	292	23	96	27
Solyc06g062400.1.1	2.348767	1.234062	0.000853	0.005554	92	577	204	19	35	29
Solyc04g054480.2.1	2.349189	6.776309	2.18E-07	4.30E-06	6767	16699	13709	1039	1980	1090
Solyc10g052490.1.1	2.349975	6.489512	4.59E-06	6.23E-05	3790	15612	12191	665	2003	824
Solyc07g056240.2.1	2.35085	2.160252	0.000495	0.003489	651	541	170	66	51	41
Solyc05g054760.2.1	2.351057	7.705867	5.29E-06	7.04E-05	8143	31637	32130	2559	3232	1911
Solyc05g012600.2.1	2.351531	3.5274	1.94E-06	2.96E-05	620	1777	1529	91	179	145
Solyc04g076740.2.1	2.351709	2.888827	1.57E-06	2.45E-05	544	1159	785	82	107	78
Solyc07g005010.2.1	2.354758	2.690316	1.32E-05	0.000156	361	1028	812	51	134	60
Solyc09g092670.2.1	2.35479	2.303341	2.59E-05	0.00028	290	731	635	44	88	50
Solyc02g067090.2.1	2.355351	4.809105	1.94E-08	5.00E-07	1708	5164	2959	237	493	306
Solyc01g088670.1.1	2.356273	1.671314	0.000167	0.001397	180	500	395	21	58	37
Solyc03g005010.2.1	2.35662	3.806596	3.20E-08	7.79E-07	964	2420	1432	163	229	125
Solyc05g011820.1.1	2.357674	0.531674	0.001403	0.008463	62	235	190	20	20	11
Solyc09g075940.2.1	2.358311	1.622544	0.000512	0.003596	256	294	401	21	33	49
Solyc09g007190.2.1	2.358618	3.741006	0.000445	0.003204	1940	1101	879	313	122	47
Solyc02g089250.2.1	2.359499	4.223586	4.03E-05	0.000415	2561	1347	1541	361	166	131
Solyc09g098040.2.1	2.35981	6.99317	8.56E-07	1.44E-05	13087	16541	11332	2105	1612	913
Solyc07g055720.2.1	2.360129	4.26528	0.000489	0.003467	3448	750	987	315	248	136
Solyc11g011210.1.1	2.36073	0.626979	0.000871	0.005653	150	161	159	17	19	17
Solyc05g012620.2.1	2.360867	3.431926	8.24E-05	0.000767	1399	1124	764	225	88	66
Solyc01g109930.2.1	2.362722	2.272241	2.08E-05	0.000231	276	800	578	50	82	45
Solyc05g014100.1.1	2.364064	3.727366	4.71E-08	1.10E-06	927	2313	1326	161	222	108
Solyc01g101100.2.1	2.365251	4.143915	8.58E-08	1.87E-06	1907	2149	1543	232	217	177
Solyc07g007020.2.1	2.365402	1.159526	0.000385	0.00283	161	365	221	13	40	27
Solyc11g069710.1.1	2.366535	2.334526	0.000301	0.002305	220	878	662	20	113	57
Solyc09g083130.2.1	2.367359	4.492234	0.000123	0.001079	2844	2309	1727	531	184	86
Solyc07g006200.2.1	2.368415	3.531992	2.23E-07	4.38E-06	797	1550	1473	113	204	110
Solyc01g009670.2.1	2.368891	1.855162	0.001116	0.006971	578	257	190	61	44	24
Solyc12g009440.1.1	2.368904	5.924292	1.03E-06	1.69E-05	7230	5716	5565	917	851	448
Solyc02g090680.2.1	2.370568	2.83877	2.11E-06	3.16E-05	666	1122	587	89	105	64
Solyc01g006020.2.1	2.370775	6.159387	9.67E-05	0.000878	11309	5048	4156	1289	730	512
Solyc07g045440.1.1	2.371242	3.476244	6.61E-07	1.15E-05	626	1656	1487	147	179	84
Solyc09g074420.2.1	2.371265	3.771039	1.00E-06	1.65E-05	1109	1941	1461	192	109	159
Solyc04g074870.2.1	2.371385	3.26062	2.30E-05	0.000252	1277	885	710	157	129	62
Solyc06g030540.2.1	2.372225	0.527874	0.001068	0.006693	96	235	148	22	23	7
Solyc02g083310.2.1	2.373756	5.273952	9.95E-05	0.000899	6388	2911	1804	697	375	287
Solyc08g048240.2.1	2.375312	2.870128	5.28E-06	7.03E-05	367	1173	973	79	127	63
Solyc02g080500.2.1	2.377897	3.87169	8.38E-07	1.42E-05	920	2313	1758	88	271	175
Solyc11g072500.1.1	2.378618	2.54863	4.97E-05	0.000496	246	800	923	64	75	65
Solyc10g006800.2.1	2.379174	7.320762	2.73E-07	5.24E-06	17210	19967	13857	1782	2844	1321
Solyc01g008520.2.1	2.380314	0.748073	0.000701	0.004706	173	250	115	25	20	13
Solyc03g119310.2.1	2.383705	2.500117	0.000958	0.006112	97	1277	746	31	109	66
Solyc04g054270.2.1	2.384206	1.529452	6.82E-05	0.000651	241	418	286	33	50	22
Solyc09g014400.2.1	2.38459	3.812462	0.000137	0.00118	323	2844	1986	67	309	152
Solyc03g119200.2.1	2.384743	2.927392	3.53E-05	0.00037	268	1667	871	119	97	55
Solyc01g103250.2.1	2.38512	1.714115	0.000152	0.001289	177	565	389	20	63	36
Solyc06g062370.2.1	2.38553	3.944724	0.00085	0.005538	503	1504	3023	46	476	109
Solyc03g117550.1.1	2.387431	1.661629	0.000409	0.00298	213	474	367	17	35	52
Solyc06g072350.2.1	2.388037	6.425012	3.69E-06	5.13E-05	4373	9751	14135	681	1319	1044
Solyc08g076300.2.1	2.388754	1.693972	0.000879	0.005693	142	590	403	10	71	38
Solyc02g083190.1.1	2.389644	0.569463	0.000685	0.004611	100	209	173	20	19	12
Solyc01g068470.2.1	2.392715	2.28841	0.000214	0.001722	609	594	319	94	50	27
Solyc04g076100.2.1	2.39393	1.527262	0.000186	0.001528	199	270	432	30	45	27
Solyc09g090870.2.1	2.395293	3.520529	3.09E-07	5.82E-06	677	2127	1237	106	228	94
Solyc06g051250.1.1	2.395885	0.866666	0.000294	0.002263	162	270	160	20	31	14
Solyc04g014680.2.1	2.396115	3.403785	1.47E-05	0.000171	573	1729	1351	48	199	134
Solyc09g015770.2.1	2.396246	6.400087	9.60E-06	0.000118	5104	19152	6826	400	2003	825
Solyc03g119690.1.1	2.396567	5.587158	1.11E-06	1.81E-05	5827	5402	3760	811	647	279
Solyc12g015710.1.1	2.396907	2.647234	5.22E-06	6.97E-05	397	1031	717	52	108	65
Solyc04g050960.2.1	2.397543	3.918163	1.98E-07	3.93E-06	815	2728	1777	121	260	163
Solyc09g074650.2.1	2.398008	4.0392	4.38E-08	1.03E-06	1233	2479	1815	241	180	148
Solyc12g056800.1.1	2.398551	4.912917	1.05E-08	2.89E-07	1674	4892	3844	314	539	254
Solyc10g086470.1.1	2.399861	1.322309	0.000115	0.001022	188	383	259	33	36	19
Solyc04g079050.1.1	2.400842	2.087048	9.93E-05	0.000899	384	692	345	74	62	18
Solyc07g052670.2.1	2.402723	1.802621	0.000177	0.001463	124	668	453	42	59	25
Solyc09g010450.1.1	2.403073	1.697007	7.63E-05	0.000717	176	478	436	36	46	31
Solyc02g084590.2.1	2.404863	2.528821	2.22E-05	0.000244	348	922	701	36	126	53
Solyc02g080570.2.1	2.40595	5.041107	9.77E-09	2.71E-07	1742	5889	3973	318	542	322
Solyc08g015960.2.1	2.407419	3.650677	3.61E-07	6.72E-06	604	2188	1618	150	183	112
Solyc12g094430.1.1	2.407529	3.790191	2.94E-07	5.59E-06	1306	1819	1367	222	142	112
Solyc04g040160.2.1	2.408514	3.368691	0.001522	0.009067	1701	773	492	248	68	38
Solyc01g096390.2.1	2.408697	4.64345	3.02E-08	7.39E-07	1251	4862	2853	239	413	244
Solyc09g082980.2.1	2.408828	3.741029	5.81E-08	1.32E-06	1002	1767	1638	173	173	121

Solyc07g045460.2.1	2.408889	2.573413	0.000168	0.001399	195	1404	638	45	150	36
Solyc05g044480.2.1	2.40891	3.52036	6.00E-05	0.000585	366	2089	1651	56	275	105
Solyc04g011900.2.1	2.40909	3.914039	0.000143	0.001223	2162	1249	1044	324	106	83
Solyc05g051240.1.1	2.409419	3.689791	4.39E-05	0.000445	703	2846	1182	49	365	97
Solyc01g005730.2.1	2.413236	3.478591	2.52E-05	0.000274	983	1460	1191	37	256	122
Solyc09g010540.2.1	2.413286	1.266592	0.000593	0.004073	177	356	262	10	36	35
Solyc10g081470.1.1	2.414035	4.221863	2.72E-08	6.74E-07	1739	2302	1978	269	235	149
Solyc06g066620.2.1	2.414448	4.793717	4.31E-07	7.86E-06	3382	2689	2427	440	317	208
Solyc11g066900.1.1	2.414565	3.634966	1.15E-06	1.87E-05	921	1755	1463	73	195	163
Solyc11g007530.1.1	2.415288	1.16978	0.000725	0.004843	215	269	224	36	13	23
Solyc11g044360.1.1	2.415989	2.313559	7.26E-06	9.27E-05	364	764	543	54	81	42
Solyc03g005820.2.1	2.416531	2.422074	2.50E-05	0.000271	259	974	656	71	92	32
Solyc05g047590.2.1	2.419507	1.416322	0.001006	0.00637	87	443	398	16	69	17
Solyc03g006870.2.1	2.422259	5.890545	6.08E-09	1.77E-07	4844	9515	5788	678	929	508
Solyc04g051660.2.1	2.42289	3.88788	8.96E-06	0.000111	418	3163	1910	126	286	129
Solyc01g020440.2.1	2.423903	3.4711	2.69E-06	3.89E-05	463	2108	1408	86	214	103
Solyc03g082960.2.1	2.424453	4.537426	1.31E-07	2.73E-06	1969	3729	2165	401	292	128
Solyc08g081140.2.1	2.424825	4.729843	1.59E-08	4.20E-07	1926	4650	2629	407	288	215
Solyc11g009010.1.1	2.424836	0.895209	0.000494	0.003483	97	333	212	12	34	19
Solyc05g054600.2.1	2.424948	2.766337	7.59E-06	9.63E-05	327	1231	844	57	111	71
Solyc10g080360.1.1	2.426242	1.542161	0.000199	0.001621	305	234	336	38	33	27
Solyc05g024020.1.1	2.427103	1.504422	0.001714	0.009994	433	257	141	56	28	13
Solyc08g007530.2.1	2.428116	0.529421	0.000691	0.004642	83	242	162	14	19	15
Solyc09g007260.2.1	2.429681	2.682002	6.87E-06	8.82E-05	504	806	782	80	121	35
Solyc12g014220.1.1	2.430257	2.352203	1.17E-05	0.000139	389	615	650	68	66	42
Solyc04g082110.2.1	2.435552	1.197134	0.001005	0.006366	123	354	298	7	50	25
Solyc10g080600.1.1	2.435918	1.823362	0.00028	0.002168	301	464	383	15	44	56
Solyc08g065260.2.1	2.436162	3.71912	2.42E-07	4.72E-06	1548	1452	1114	162	176	117
Solyc11g020040.1.1	2.436989	3.551408	0.000293	0.002251	227	2949	1360	51	237	133
Solyc04g071610.2.1	2.437726	6.030625	1.13E-06	1.83E-05	3701	14988	5575	1021	982	345
Solyc05g025500.2.1	2.437899	3.290204	6.28E-07	1.10E-05	951	1337	906	145	129	67
Solyc07g038160.2.1	2.43799	1.886143	0.000124	0.001082	473	286	333	35	60	34
Solyc12g056220.1.1	2.440106	6.426783	3.03E-06	4.32E-05	10484	9739	6747	1485	839	599
Solyc08g007430.1.1	2.440156	0.617963	0.001056	0.006627	99	266	155	7	24	20
Solyc11g065680.1.1	2.440985	1.890218	0.000277	0.002147	531	313	247	33	43	46
Solyc04g079080.2.1	2.442889	0.129336	0.001025	0.006477	85	158	109	13	14	9
Solyc02g087060.2.1	2.445071	2.71267	0.000497	0.003502	177	1096	1045	24	108	91
Solyc07g005930.2.1	2.44537	4.757668	2.13E-08	5.45E-07	2091	4174	2904	202	611	199
Solyc03g113460.1.1	2.445428	2.724774	7.38E-05	0.000698	874	507	575	85	53	75
Solyc06g053830.2.1	2.445434	2.951743	1.36E-06	2.16E-05	564	1625	586	90	124	59
Solyc12g088650.1.1	2.445681	2.360028	0.000136	0.001179	242	836	701	24	74	73
Solyc10g078160.1.1	2.445916	2.726091	1.33E-06	2.11E-05	538	917	732	68	112	54
Solyc08g079100.2.1	2.447047	3.276448	6.09E-05	0.000592	1201	1287	604	157	172	28
Solyc06g072710.2.1	2.448109	2.013027	0.000153	0.001291	331	596	414	77	48	15
Solyc04g011580.2.1	2.449602	4.308143	4.40E-09	1.31E-07	1734	2564	2187	182	350	173
Solyc09g065160.2.1	2.45	4.699157	4.88E-07	8.78E-06	2849	3514	2063	458	310	135
Solyc07g043330.1.1	2.454108	3.176035	2.00E-06	3.02E-05	476	1433	1212	81	116	105
Solyc01g028870.2.1	2.455332	3.79842	7.18E-08	1.59E-06	730	2449	1725	138	233	119
Solyc02g070210.2.1	2.457185	1.944189	0.000503	0.003538	225	255	707	62	36	29
Solyc08g007770.2.1	2.457481	2.778053	2.23E-06	3.32E-05	546	1191	622	93	73	62
Solyc07g043490.1.1	2.460414	3.355023	0.000138	0.001189	192	2915	982	137	129	82
Solyc04g053120.2.1	2.460664	4.126689	1.71E-08	4.47E-07	1272	2609	2029	255	220	124
Solyc05g006170.2.1	2.462696	4.788203	2.43E-06	3.56E-05	980	5341	3726	159	550	278
Solyc04g071800.2.1	2.464313	5.681609	4.55E-07	8.23E-06	5633	6786	4209	883	571	299
Solyc05g005880.2.1	2.464744	5.425109	8.23E-06	0.000103	4860	4491	4100	878	367	201
Solyc04g071930.2.1	2.465332	3.542061	0.001531	0.009107	2051	620	574	265	83	41
Solyc06g082980.2.1	2.467571	5.807057	6.66E-06	8.60E-05	4688	9557	5032	1152	467	260
Solyc04g074080.2.1	2.469721	3.205264	0.000274	0.002128	1125	903	808	210	68	30
Solyc11g071550.1.1	2.471687	2.475215	7.79E-05	0.00073	600	392	677	53	125	28
Solyc08g008380.2.1	2.47229	2.996302	0.000133	0.00115	1095	766	557	158	79	33
Solyc04g009260.1.1	2.472405	1.612866	9.68E-05	0.000879	195	351	446	24	60	24
Solyc11g069430.1.1	2.47246	6.602524	2.63E-07	5.08E-06	8613	14723	9357	588	1548	1168
Solyc01g097510.2.1	2.473271	2.972193	7.36E-05	0.000697	252	1467	1114	46	193	54
Solyc07g062300.2.1	2.475687	2.541371	3.78E-06	5.25E-05	401	890	684	45	105	53
Solyc07g042500.2.1	2.475721	5.153933	6.16E-08	1.39E-06	1700	7710	3752	288	696	291
Solyc02g077160.2.1	2.480338	2.505163	3.66E-06	5.09E-05	451	841	610	44	86	61
Solyc01g010480.2.1	2.482446	7.244433	1.06E-06	1.74E-05	7608	24757	20753	1012	3013	1377
Solyc08g065820.1.1	2.484826	0.366496	0.001513	0.009016	53	180	195	9	25	10
Solyc11g007830.1.1	2.487779	4.965943	2.02E-10	7.98E-09	2394	5021	3298	327	453	281
Solyc04g056580.2.1	2.489659	4.070941	5.23E-08	1.20E-06	1606	2046	1790	247	236	96
Solyc03g093880.2.1	2.490128	3.071964	7.36E-07	1.26E-05	597	1622	761	75	105	95
Solyc07g063770.2.1	2.490953	2.937492	1.70E-05	0.000194	378	1931	609	44	150	74
Solyc08g006270.1.1	2.493642	0.721476	0.000256	0.00201	127	261	161	20	19	14
Solyc05g009780.2.1	2.494331	4.140865	3.29E-08	7.98E-07	1096	2758	2224	238	275	101
Solyc09g089690.2.1	2.495937	4.119184	2.62E-06	3.81E-05	2035	1824	1576	318	151	101
Solyc03g119640.2.1	2.496064	3.044359	3.94E-07	7.26E-06	793	1274	695	82	139	64
Solyc02g086730.1.1	2.496065	5.822815	3.25E-06	4.59E-05	6507	5882	5355	1039	506	321
Solyc01g095170.2.1	2.497992	1.894005	0.000717	0.004802	66	911	449	27	71	31
Solyc02g082120.2.1	2.498024	5.390696	2.19E-09	6.91E-08	3673	7022	3699	406	819	275
Solyc03g113010.1.1	2.498161	3.58168	2.46E-08	6.19E-07	1196	1640	1093	124	175	105
Solyc08g075120.2.1	2.498527	2.363663	9.24E-05	0.000845	311	685	724	42	36	77
Solyc09g098290.2.1	2.499034	3.609398	6.29E-07	1.10E-05	950	1604	1491	202	112	84
Solyc12g013810.1.1	2.49917	5.884719	3.69E-07	6.86E-06	7552	5891	4813	805	689	467
Solyc01g098340.2.1	2.499585	3.603978	1.68E-08	4.39E-07	899	2127	1207	116	172	118

Solyc12g008400.1.1	2.499732	2.358088	8.58E-06	0.000107	415	807	512	41	60	63
Solyc08g077050.2.1	2.500558	3.897082	8.85E-08	1.92E-06	1241	2098	1634	229	140	115
Solyc12g056510.1.1	2.504309	3.545761	2.01E-07	4.00E-06	1066	1714	1119	174	111	93
Solyc11g066670.1.1	2.505692	3.542668	1.44E-06	2.28E-05	491	2300	1450	149	112	112
Solyc07g043600.2.1	2.506969	4.057195	7.97E-08	1.74E-06	1042	2686	2042	108	349	135
Solyc10g055760.1.1	2.507188	3.386897	0.000314	0.00239	609	2758	651	20	244	109
Solyc12g006320.1.1	2.507255	6.843677	1.03E-07	2.21E-06	7395	22128	11633	1665	1428	762
Solyc05g018510.2.1	2.512191	4.73805	5.48E-05	0.00054	3681	2687	1826	554	257	81
Solyc04g009740.2.1	2.513929	4.858205	2.30E-08	5.82E-07	3458	3454	2320	396	380	197
Solyc02g090890.2.1	2.514355	5.744902	1.66E-05	0.00019	7238	6121	3426	241	1390	367
Solyc05g053570.2.1	2.515295	1.317792	7.98E-05	0.000746	139	464	274	23	37	22
Solyc12g088430.1.1	2.515587	0.269992	0.00076	0.005041	109	127	136	12	15	11
Solyc06g008050.2.1	2.518308	0.56061	0.001256	0.007691	146	237	101	8	14	22
Solyc08g067240.2.1	2.519187	2.037812	8.99E-06	0.000111	316	669	416	32	69	37
Solyc03g117860.2.1	2.51934	6.153512	0.000948	0.006062	13638	4064	2152	1306	855	253
Solyc07g006630.2.1	2.519871	3.351031	1.40E-06	2.22E-05	1219	1045	915	116	187	55
Solyc10g018530.1.1	2.520361	1.246117	0.000171	0.001428	213	394	192	26	47	10
Solyc04g071280.1.1	2.522938	2.304902	3.90E-06	5.39E-05	332	875	519	54	66	42
Solyc04g028470.1.1	2.522988	0.677859	0.000325	0.002467	160	233	125	13	22	16
Solyc10g009570.2.1	2.524353	3.871572	0.001484	0.008863	2928	569	451	247	94	115
Solyc03g117540.2.1	2.52818	3.793467	2.70E-08	6.71E-07	1195	1980	1466	189	203	78
Solyc12g007140.1.1	2.528329	3.639185	6.22E-07	1.09E-05	714	2156	1520	68	197	145
Solyc08g082570.2.1	2.529046	2.972616	4.30E-06	5.87E-05	690	1187	764	136	77	42
Solyc07g053020.1.1	2.530549	4.151669	9.14E-06	0.000113	1028	3227	2069	50	348	206
Solyc09g011770.2.1	2.532796	1.608574	0.000161	0.001351	109	491	465	24	50	27
Solyc06g071790.2.1	2.535591	6.526883	3.73E-05	0.000388	9665	11024	9089	1980	577	378
Solyc12g014250.1.1	2.536571	6.553936	3.30E-06	4.65E-05	3590	18244	12558	518	1818	893
Solyc05g012100.2.1	2.540263	4.589149	2.79E-07	5.34E-06	1045	5017	2834	157	502	185
Solyc02g070000.2.1	2.540417	2.75977	0.000303	0.002317	284	1220	920	14	149	71
Solyc02g079870.2.1	2.542599	3.082745	5.84E-07	1.03E-05	632	1082	1097	72	161	59
Solyc03g113680.2.1	2.543442	2.520696	0.000132	0.001146	846	400	433	91	48	41
Solyc02g064640.2.1	2.544314	3.357199	9.56E-07	1.58E-05	605	1546	1377	103	209	50
Solyc02g077260.2.1	2.545769	1.204152	7.28E-05	0.000691	166	452	193	25	31	18
Solyc03g093550.1.1	2.548116	3.523241	3.47E-05	0.000365	308	3345	979	70	206	110
Solyc03g006970.1.1	2.55059	3.141376	0.000152	0.00129	632	1714	802	197	48	31
Solyc08g079110.2.1	2.551333	2.724754	8.41E-05	0.00078	626	743	753	115	27	55
Solyc09g007550.2.1	2.552353	2.273122	1.29E-05	0.000152	436	595	541	28	77	52
Solyc10g044840.1.1	2.553487	1.844977	0.000229	0.001828	543	303	217	52	38	24
Solyc06g059870.1.1	2.555003	6.399983	5.80E-08	1.32E-06	8996	11591	7241	1142	832	693
Solyc01g098560.2.1	2.555123	4.916205	2.45E-08	6.18E-07	2957	4716	2537	482	316	176
Solyc10g085330.1.1	2.555155	1.622773	5.06E-05	0.000504	304	449	263	19	63	23
Solyc03g112210.1.1	2.556011	3.288034	1.39E-07	2.87E-06	937	1300	979	126	135	61
Solyc03g111700.2.1	2.558638	4.109578	3.46E-08	8.30E-07	1887	2505	1324	249	189	116
Solyc07g018360.2.1	2.559189	6.997127	8.75E-09	2.45E-07	8667	20194	15411	1295	1853	1049
Solyc02g082680.2.1	2.56027	4.27128	1.23E-09	4.08E-08	1876	2487	1976	169	302	166
Solyc05g050220.2.1	2.561104	0.645042	0.001085	0.006788	57	428	117	19	22	9
Solyc11g010960.1.1	2.56139	2.842398	9.29E-05	0.000847	897	825	540	137	76	21
Solyc12g019110.1.1	2.562051	2.983259	1.43E-06	2.27E-05	482	1342	948	47	124	85
Solyc01g096660.2.1	2.562364	5.284282	1.39E-08	3.75E-07	4790	4479	3105	501	489	275
Solyc01g087500.2.1	2.563666	1.90416	0.000523	0.003659	161	291	743	36	27	47
Solyc12g011010.1.1	2.566115	1.403268	4.13E-05	0.000423	265	330	255	22	40	23
Solyc02g093720.2.1	2.566504	2.236054	0.000433	0.003131	702	308	360	82	29	32
Solyc08g080000.2.1	2.566611	2.258739	9.94E-05	0.000899	263	884	559	29	127	19
Solyc08g075950.1.1	2.567217	0.925196	0.00035	0.002618	89	276	278	19	18	20
Solyc01g091450.2.1	2.568465	2.149529	2.08E-05	0.000231	317	713	494	21	83	43
Solyc08g007380.2.1	2.569192	1.409631	7.61E-05	0.000715	156	417	338	29	47	13
Solyc11g012690.1.1	2.56995	1.553669	0.000263	0.002052	239	384	341	10	37	42
Solyc06g060880.2.1	2.570073	1.087817	0.000119	0.001051	207	320	174	26	21	18
Solyc07g056310.2.1	2.570655	0.550784	0.000808	0.005309	119	189	165	5	27	15
Solyc07g005830.2.1	2.571132	4.654517	1.80E-09	5.78E-08	2117	3462	2859	345	271	183
Solyc07g062870.2.1	2.571519	5.075323	2.13E-09	6.74E-08	2480	4797	4168	469	336	255
Solyc01g010130.2.1	2.574026	2.405038	1.42E-06	2.25E-05	400	807	595	51	77	43
Solyc09g010140.1.1	2.57567	0.430592	0.001048	0.006588	157	162	100	22	12	7
Solyc09g089540.2.1	2.576058	10.02423	0.000318	0.002416	16420	94003	238461	9709	15363	8845
Solyc07g066610.2.1	2.577903	7.544322	5.96E-06	7.83E-05	27020	16218	13298	2797	1867	1232
Solyc10g083300.1.1	2.578538	2.804889	0.000162	0.00136	845	868	524	146	51	22
Solyc04g081320.2.1	2.578783	4.061623	0.00158	0.009337	3337	620	567	330	115	74
Solyc06g076470.2.1	2.578834	4.136422	2.21E-07	4.36E-06	2299	1549	1533	229	215	120
Solyc12g010870.1.1	2.580874	4.373295	4.14E-09	1.24E-07	1993	2438	2308	262	295	123
Solyc11g065530.1.1	2.582828	2.972989	0.000148	0.001262	201	1577	1135	51	200	33
Solyc09g064870.2.1	2.583374	0.69328	0.000779	0.005149	201	154	134	23	23	6
Solyc09g007570.1.1	2.584415	0.031625	0.001535	0.009127	114	103	92	14	14	4
Solyc03g118240.2.1	2.584523	4.519528	3.12E-10	1.20E-08	1693	3267	2830	250	299	184
Solyc01g091800.2.1	2.584877	2.552741	9.86E-06	0.000121	257	937	866	44	109	43
Solyc12g010910.1.1	2.585197	2.718593	9.28E-05	0.000847	549	692	880	127	66	18
Solyc11g056650.1.1	2.586089	2.792973	6.33E-06	8.24E-05	475	1663	458	41	107	73
Solyc05g052160.2.1	2.589817	3.243664	1.19E-06	1.93E-05	657	1657	1017	44	171	95
Solyc04g072780.2.1	2.593462	5.064893	8.65E-07	1.45E-05	1233	6652	4480	170	510	381
Solyc10g086640.1.1	2.596675	0.683241	0.000311	0.002372	105	183	230	16	17	15
Solyc06g069170.2.1	2.596711	5.705314	1.22E-09	4.04E-08	4201	7416	6055	655	676	343
Solyc08g065950.1.1	2.599956	3.641822	1.13E-07	2.38E-06	1176	1883	1155	145	94	130
Solyc05g051450.1.1	2.600481	1.14369	0.000334	0.00252	254	346	128	12	50	13
Solyc01g102300.2.1	2.60057	4.275801	0.000153	0.001297	332	5119	2254	71	419	168
Solyc01g006940.2.1	2.6006	4.371691	6.12E-10	2.18E-08	1707	2812	2429	248	283	135

Solyc01g079570.2.1	2.600958	0.543187	0.000623	0.004253	137	142	172	7	23	15
Solyc06g054250.2.1	2.6013	4.370102	3.62E-10	1.35E-08	1805	2937	2220	187	253	202
Solyc01g057200.2.1	2.602046	2.112735	4.64E-06	6.29E-05	305	778	437	32	63	41
Solyc12g010050.1.1	2.60335	1.480269	0.000172	0.001431	186	366	377	10	55	26
Solyc03g114250.2.1	2.603854	2.731265	7.78E-07	1.32E-05	602	980	652	88	84	39
Solyc10g081730.1.1	2.604374	0.219256	0.000791	0.005218	77	186	127	11	9	13
Solyc03g123490.1.1	2.605322	3.519574	9.19E-07	1.53E-05	593	2182	1378	57	171	131
Solyc09g074880.2.1	2.607129	1.938969	6.30E-05	0.00061	322	431	487	37	24	48
Solyc03g096850.2.1	2.609561	4.288467	0.000344	0.002583	2723	1635	1569	447	106	45
Solyc01g005720.2.1	2.610063	3.088007	1.88E-06	2.87E-05	620	1278	1013	38	160	80
Solyc03g093340.2.1	2.610685	2.762159	1.30E-05	0.000153	917	660	511	50	71	81
Solyc03g078640.1.1	2.611621	3.384812	1.41E-05	0.000165	410	1913	1465	39	208	98
Solyc08g065430.2.1	2.611965	2.1472	0.00017	0.001414	255	818	508	17	122	20
Solyc08g065940.2.1	2.61445	5.69124	4.55E-09	1.35E-07	5355	7426	4490	693	565	351
Solyc01g056780.2.1	2.615671	3.773344	3.58E-08	8.57E-07	1391	1623	1428	190	155	83
Solyc06g074820.2.1	2.61716	4.748536	2.07E-07	4.10E-06	1185	3612	4476	212	524	168
Solyc11g013390.1.1	2.617732	0.473018	0.000311	0.002368	103	192	161	18	16	8
Solyc04g054990.2.1	2.6182	5.199842	2.56E-08	6.39E-07	1832	4768	5972	296	591	298
Solyc05g006770.2.1	2.619211	0.371466	0.001032	0.006505	69	208	163	6	14	17
Solyc03g096460.2.1	2.619353	3.500997	0.001427	0.008589	2070	396	648	235	59	45
Solyc07g006000.2.1	2.621336	4.442572	1.40E-06	2.22E-05	2751	2091	1927	353	143	150
Solyc01g111260.2.1	2.62325	4.720608	2.55E-08	6.37E-07	1091	5747	3078	287	392	169
Solyc09g065760.2.1	2.627472	3.114384	1.10E-06	1.80E-05	381	2017	889	85	101	78
Solyc01g108070.2.1	2.62781	2.899381	5.44E-07	9.68E-06	775	897	748	97	91	45
Solyc01g100760.2.1	2.634584	3.482541	1.55E-06	2.41E-05	495	1921	1587	167	130	53
Solyc05g051640.2.1	2.63558	1.185004	0.000112	0.000999	116	294	353	18	36	17
Solyc12g014610.1.1	2.637753	4.390088	3.52E-10	1.32E-08	2178	3012	1823	204	321	144
Solyc10g006370.2.1	2.639872	4.110534	2.24E-09	7.02E-08	1404	2361	2059	216	167	136
Solyc04g071150.2.1	2.639969	2.121043	0.000137	0.00118	460	382	511	78	25	24
Solyc10g084970.1.1	2.640139	2.698688	3.14E-07	5.92E-06	590	1011	608	72	76	50
Solyc05g026240.1.1	2.645098	2.260072	1.65E-05	0.000189	304	696	640	71	60	19
Solyc09g089730.2.1	2.648148	4.183493	4.78E-09	1.41E-07	1118	3661	1866	117	285	167
Solyc06g008220.2.1	2.648441	4.150302	2.06E-08	5.29E-07	946	3018	2362	110	300	154
Solyc01g106590.2.1	2.649827	1.788409	6.22E-06	8.12E-05	249	559	384	36	46	24
Solyc09g008640.1.1	2.652173	1.713589	6.76E-05	0.000646	216	539	384	54	34	12
Solyc01g058160.2.1	2.652929	0.020968	0.000738	0.004914	88	164	84	11	16	4
Solyc09g010160.1.1	2.654288	2.914338	0.000453	0.003253	130	1524	1176	22	165	62
Solyc12g008410.1.1	2.654985	3.507302	5.19E-07	9.30E-06	603	2077	1420	54	193	110
Solyc10g084320.1.1	2.655921	7.690971	2.32E-05	0.000254	11288	16442	39252	3420	1485	1206
Solyc09g074260.1.1	2.663476	2.768185	0.000292	0.002248	988	672	436	116	90	11
Solyc03g063560.2.1	2.663875	8.027335	5.65E-05	0.000555	38998	23115	17446	4915	1916	984
Solyc02g089690.2.1	2.665265	1.212996	0.000138	0.001191	116	358	328	24	41	9
Solyc11g067130.1.1	2.666574	0.528324	0.000234	0.001866	129	203	141	9	27	9
Solyc11g006680.1.1	2.667208	3.818001	2.04E-09	6.48E-08	952	2278	1708	155	197	87
Solyc12g055720.1.1	2.669074	4.412516	2.49E-07	4.85E-06	1749	3467	2184	371	150	106
Solyc03g111260.2.1	2.672512	4.521313	1.29E-10	5.29E-09	1594	3837	2658	176	327	200
Solyc05g050550.2.1	2.673064	1.508719	0.000803	0.005284	381	283	212	57	18	8
Solyc07g045140.2.1	2.675196	1.983843	0.000202	0.00164	379	694	295	59	62	6
Solyc09g056170.2.1	2.677286	1.15306	0.000195	0.001597	198	247	270	23	43	6
Solyc12g013980.1.1	2.678385	0.034521	0.000481	0.003417	75	180	93	10	14	6
Solyc04g063240.2.1	2.68129	3.334211	0.000225	0.001798	1606	870	559	198	72	29
Solyc07g006910.2.1	2.681603	1.278018	0.000493	0.003479	75	421	372	34	34	7
Solyc03g013160.2.1	2.683617	2.118851	0.000729	0.004867	121	513	850	10	79	45
Solyc03g034170.2.1	2.684061	3.87884	0.00036	0.002675	2497	970	822	295	61	63
Solyc02g070040.1.1	2.686847	2.420128	0.000392	0.002876	150	1617	422	19	131	30
Solyc01g057830.2.1	2.68686	6.038172	1.35E-06	2.15E-05	6321	8431	7107	1175	464	288
Solyc08g016720.1.1	2.688952	3.483874	0.000786	0.005187	84	2346	1846	52	246	71
Solyc06g050170.2.1	2.689993	4.140109	7.04E-09	2.02E-07	2198	1954	1472	146	268	130
Solyc06g073940.2.1	2.690306	2.292162	1.88E-05	0.000212	175	1209	511	36	79	36
Solyc01g111990.2.1	2.690692	4.782176	2.03E-11	9.98E-10	2331	4175	2951	260	415	179
Solyc07g056330.2.1	2.691406	1.397531	0.00033	0.002498	65	566	357	17	50	16
Solyc10g039270.1.1	2.692977	2.12658	3.90E-06	5.39E-05	286	654	564	54	45	29
Solyc03g119590.1.1	2.693184	2.410875	0.000143	0.001227	236	1292	512	16	54	77
Solyc06g083220.2.1	2.69375	2.709007	4.41E-07	8.00E-06	359	1215	788	56	85	54
Solyc05g009570.2.1	2.694388	2.536643	1.71E-06	2.64E-05	570	678	642	77	54	38
Solyc04g071420.2.1	2.695717	3.447078	3.41E-08	8.20E-07	701	1843	1317	67	161	101
Solyc08g076970.2.1	2.696597	11.22356	4.01E-07	7.38E-06	107693	400204	352019	31085	26036	14413
Solyc03g083770.1.1	2.701589	4.947916	2.55E-06	3.71E-05	3756	3456	2690	360	594	66
Solyc03g114640.2.1	2.701611	2.822761	8.19E-06	0.000103	792	1039	533	109	85	22
Solyc11g013270.1.1	2.702407	0.299688	0.000872	0.005659	100	165	136	7	8	17
Solyc04g071470.2.1	2.702461	2.567855	1.90E-05	0.000214	598	867	527	100	31	35
Solyc08g078300.2.1	2.703886	2.080868	0.000106	0.000953	114	878	593	21	79	32
Solyc07g062260.2.1	2.70499	2.032456	0.000396	0.002903	123	664	675	10	62	48
Solyc05g007780.2.1	2.706342	2.724882	0.00083	0.005424	1234	390	257	120	41	28
Solyc01g107670.2.1	2.707234	4.69368	0.000463	0.003312	403	5553	3967	42	502	263
Solyc04g007410.2.1	2.710627	3.034551	6.44E-08	1.45E-06	811	1009	877	83	109	54
Solyc09g065840.2.1	2.710653	3.819274	5.38E-08	1.23E-06	1829	1434	1181	164	174	85
Solyc02g090020.2.1	2.71228	2.897872	7.13E-05	0.000679	1079	707	508	123	33	49
Solyc01g111110.2.1	2.715101	0.667523	0.000725	0.004843	62	271	226	6	19	19
Solyc01g088600.2.1	2.717326	2.126546	2.75E-06	3.96E-05	251	735	559	40	65	27
Solyc10g085390.1.1	2.717884	2.359457	1.07E-05	0.000129	330	863	616	21	64	61
Solyc11g066410.1.1	2.717983	4.771427	7.11E-08	1.58E-06	2345	3383	3395	443	214	132
Solyc08g081160.2.1	2.719837	3.063642	7.86E-06	9.91E-05	222	1598	1284	63	128	62
Solyc07g052390.2.1	2.720243	4.121365	8.71E-10	3.00E-08	1193	2581	2261	117	238	159

Solyc02g081060.2.1	2.720736	2.306208	2.94E-05	0.000314	693	301	470	49	68	31
Solyc04g082310.2.1	2.733503	4.36678	2.64E-08	6.58E-07	930	3756	2847	122	273	204
Solyc11g044530.1.1	2.733837	2.43492	9.91E-05	0.000898	594	533	589	101	39	15
Solyc07g063190.2.1	2.735341	6.706458	1.41E-08	3.80E-07	10444	13785	10707	1372	934	679
Solyc06g051860.1.1	2.73788	2.679654	0.00143	0.008599	44	1277	1106	30	145	33
Solyc11g010600.1.1	2.739341	6.721193	8.69E-09	2.44E-07	6620	18433	12834	1254	1366	543
Solyc02g087400.1.1	2.739346	4.258805	2.64E-10	1.03E-08	2038	2493	1821	204	240	121
Solyc01g099980.2.1	2.73979	3.236118	6.53E-06	8.47E-05	280	2186	1172	124	112	43
Solyc06g007160.2.1	2.740297	0.346529	0.000448	0.003224	114	162	135	18	8	8
Solyc06g082910.2.1	2.740417	3.836633	1.20E-05	0.000142	497	2463	2240	42	285	118
Solyc11g072510.1.1	2.741216	2.451407	1.32E-06	2.11E-05	386	780	708	61	44	46
Solyc09g076030.2.1	2.742408	2.249243	1.54E-05	0.000178	336	709	595	22	107	24
Solyc08g016080.2.1	2.743148	5.101693	6.85E-05	0.000654	6212	2935	1092	553	298	141
Solyc01g088610.2.1	2.743535	4.474704	8.95E-08	1.94E-06	925	3887	3274	117	304	223
Solyc05g013510.2.1	2.744241	3.989263	3.42E-09	1.04E-07	1951	2091	1173	164	193	107
Solyc02g071720.2.1	2.744253	0.049544	0.001714	0.009993	41	164	150	3	16	10
Solyc12g010230.1.1	2.746448	0.284345	0.000615	0.004203	78	184	149	17	15	3
Solyc01g057260.2.1	2.746716	2.68874	4.17E-07	7.62E-06	658	873	619	46	114	38
Solyc11g044940.1.1	2.746924	1.84433	6.29E-06	8.20E-05	185	607	484	32	44	27
Solyc01g104950.2.1	2.748743	7.615717	3.86E-05	0.000399	33791	14378	9951	3028	1580	974
Solyc10g085600.1.1	2.751176	2.455584	1.31E-06	2.10E-05	311	909	726	35	89	40
Solyc02g090900.2.1	2.751314	2.187943	4.29E-05	0.000438	254	699	642	15	100	29
Solyc09g007110.2.1	2.7532	2.98407	5.98E-06	7.85E-05	321	1446	1126	32	114	82
Solyc02g081980.2.1	2.755961	2.136886	0.001021	0.006453	589	244	469	88	13	17
Solyc10g080390.1.1	2.759353	3.056171	6.31E-07	1.10E-05	825	1112	843	123	66	44
Solyc06g005710.2.1	2.763764	4.519969	0.000117	0.001035	4183	1253	1149	391	144	102
Solyc10g054110.1.1	2.764599	4.41084	1.62E-06	2.51E-05	671	6485	1647	264	246	109
Solyc06g068950.2.1	2.765698	1.288491	1.78E-05	0.000203	183	364	286	27	25	16
Solyc08g007240.2.1	2.765781	6.432898	4.24E-06	5.80E-05	11957	8606	6598	1393	488	481
Solyc08g029090.2.1	2.765994	1.367527	5.85E-05	0.000572	116	635	238	16	40	19
Solyc03g117850.2.1	2.766108	4.371509	0.000172	0.001431	3834	1511	720	360	158	69
Solyc02g092810.2.1	2.770092	2.358866	1.13E-05	0.000135	388	615	709	27	43	65
Solyc08g067840.2.1	2.774192	3.065438	3.71E-07	6.89E-06	734	901	1110	105	65	59
Solyc06g075090.2.1	2.774595	1.671592	0.000125	0.001091	376	623	115	22	62	14
Solyc02g037530.2.1	2.774636	3.417971	2.78E-08	6.86E-07	589	2112	1241	71	173	74
Solyc03g097380.2.1	2.77514	2.339612	1.99E-06	3.02E-05	332	1113	440	32	58	50
Solyc02g080530.2.1	2.775309	6.880355	9.05E-09	2.54E-07	9534	22215	10756	1541	1236	603
Solyc03g118740.2.1	2.775914	1.595465	2.43E-05	0.000264	185	483	389	13	50	25
Solyc01g094690.2.1	2.777671	2.882148	1.68E-06	2.59E-05	418	854	1220	54	66	78
Solyc06g071290.2.1	2.782645	3.625126	3.39E-05	0.000358	1869	1060	843	213	57	61
Solyc07g007260.2.1	2.784254	0.177786	0.001595	0.009402	151	50	120	10	11	9
Solyc08g066450.1.1	2.785221	0.414296	0.000395	0.002898	132	122	160	6	25	8
Solyc03g053110.2.1	2.787959	5.582862	6.84E-10	2.41E-08	4753	5815	5351	595	466	284
Solyc08g082890.2.1	2.789327	3.200734	2.88E-05	0.000309	197	1370	1736	61	96	93
Solyc10g008270.2.1	2.790493	3.737612	1.15E-06	1.87E-05	1978	1581	692	156	185	53
Solyc01g080580.2.1	2.790731	1.986053	6.79E-06	8.75E-05	493	540	267	39	56	20
Solyc12g014040.1.1	2.792546	0.44703	0.001284	0.007835	70	176	210	14	4	15
Solyc04g009180.1.1	2.792834	3.172425	2.17E-05	0.000239	408	1552	1305	21	185	69
Solyc03g033410.2.1	2.793918	3.027193	4.55E-05	0.000459	186	1508	1338	32	146	64
Solyc09g009040.2.1	2.794273	4.848963	3.22E-08	7.82E-07	1010	5267	4379	243	444	166
Solyc07g039190.2.1	2.795966	2.940262	1.56E-07	3.14E-06	517	1246	947	96	88	35
Solyc09g061930.2.1	2.796952	1.560649	1.01E-05	0.000123	168	462	402	26	30	24
Solyc08g062960.2.1	2.798364	2.817996	1.27E-05	0.00015	993	782	460	97	87	21
Solyc10g080940.1.1	2.798506	4.789169	1.15E-11	5.90E-10	1943	4241	3514	263	368	167
Solyc04g072030.1.1	2.800695	3.251021	4.24E-06	5.80E-05	313	1587	1559	45	128	93
Solyc12g008420.1.1	2.801847	2.285641	5.38E-07	9.58E-06	328	850	559	36	61	39
Solyc06g084320.1.1	2.801999	1.911623	0.000114	0.00101	355	752	240	66	19	15
Solyc02g085750.2.1	2.802606	2.325	0.000927	0.005964	629	509	448	4	134	25
Solyc11g010810.1.1	2.805537	4.97278	2.16E-11	1.06E-09	1936	5029	4199	260	426	214
Solyc01g103360.2.1	2.807861	0.312856	0.001569	0.009285	169	90	108	5	29	4
Solyc05g009680.1.1	2.808591	3.675914	1.10E-06	1.79E-05	565	2245	1849	46	163	141
Solyc11g012360.1.1	2.813311	5.301521	3.61E-05	0.000377	5752	5868	1456	683	338	89
Solyc04g079340.2.1	2.813653	2.214139	2.73E-06	3.94E-05	492	485	517	29	51	45
Solyc02g086740.1.1	2.815061	4.186279	4.33E-05	0.000441	2665	1358	1513	339	85	62
Solyc03g114850.2.1	2.815803	2.81242	1.86E-05	0.00021	229	1555	906	24	107	67
Solyc08g076880.2.1	2.816245	5.008763	1.51E-06	2.37E-05	1335	9581	2302	125	613	228
Solyc05g012680.2.1	2.816868	1.131617	0.001223	0.007518	42	402	360	5	35	21
Solyc04g015490.2.1	2.819421	4.730077	4.10E-05	0.00042	3046	3360	2376	536	126	54
Solyc08g006260.1.1	2.819482	0.492392	0.000251	0.001977	62	265	178	11	23	6
Solyc12g044410.1.1	2.819711	2.886473	1.18E-07	2.47E-06	400	1384	921	63	111	40
Solyc04g007790.2.1	2.819868	7.862704	2.68E-09	8.28E-08	16958	34697	29730	2433	2959	1256
Solyc12g044840.1.1	2.821854	-0.28575	0.001223	0.007517	38	162	90	5	15	3
Solyc11g072610.1.1	2.825995	0.665528	8.26E-05	0.000768	139	234	159	11	15	15
Solyc03g080010.2.1	2.826475	3.639481	2.46E-10	9.61E-09	930	1995	1463	107	143	93
Solyc02g092590.2.1	2.832453	3.282365	1.39E-07	2.86E-06	1168	1109	880	116	118	41
Solyc09g060100.2.1	2.835165	1.913986	3.59E-05	0.000375	258	654	428	62	27	12
Solyc08g082170.2.1	2.835675	4.691817	6.82E-12	3.65E-10	1886	3928	3245	254	274	178
Solyc03g119060.2.1	2.835835	5.867745	1.21E-10	4.97E-09	3698	11308	6503	463	789	395
Solyc07g064600.2.1	2.838315	10.82231	3.17E-05	0.000337	113461	171044	318095	32261	11580	5110
Solyc06g074070.2.1	2.839505	1.371243	5.07E-05	0.000505	243	288	311	37	24	9
Solyc10g075070.1.1	2.843007	7.748946	0.000636	0.004336	1910	56377	29192	510	4544	1466
Solyc07g064830.2.1	2.844287	2.515273	2.09E-05	0.000232	184	1184	794	23	106	38
Solyc11g011980.1.1	2.844548	2.981986	1.51E-06	2.37E-05	973	1054	587	58	154	27
Solyc03g113210.2.1	2.84693	3.123474	3.00E-07	5.68E-06	624	895	1375	47	129	68

Solyc09g090510.2.1	2.848648	5.396807	8.38E-05	0.000778	6769	3452	2616	799	219	102
Solyc01g104860.2.1	2.849061	1.33429	0.001042	0.006554	381	196	180	43	23	3
Solyc06g054260.1.1	2.854727	8.15516	0.000362	0.002684	52268	17447	13574	5316	1384	805
Solyc03g044470.2.1	2.855776	4.019003	1.46E-07	2.98E-06	899	2807	2172	53	281	130
Solyc08g066940.2.1	2.856735	4.250299	6.72E-07	1.16E-05	557	3716	2901	92	228	188
Solyc10g081490.1.1	2.860542	2.173251	0.000675	0.004554	679	381	318	83	34	5
Solyc07g049310.2.1	2.861939	2.562673	2.17E-06	3.23E-05	501	810	703	86	52	21
Solyc02g067310.2.1	2.864765	3.067178	1.26E-06	2.02E-05	359	1461	1240	40	90	88
Solyc08g006540.2.1	2.867607	2.613882	9.02E-07	1.51E-05	552	811	706	69	34	50
Solyc05g041430.2.1	2.868806	1.372768	0.000271	0.002113	400	173	194	35	27	8
Solyc08g081940.2.1	2.869236	2.91698	4.32E-08	1.01E-06	753	983	798	80	62	54
Solyc11g066510.1.1	2.87019	2.198455	1.26E-05	0.000149	258	872	551	14	84	33
Solyc05g015310.2.1	2.870249	2.563891	2.88E-07	5.48E-06	299	1069	790	45	71	43
Solyc01g008920.2.1	2.871785	2.408645	1.80E-05	0.000205	839	433	339	63	45	31
Solyc03g020080.2.1	2.872835	5.754202	8.47E-05	0.000785	6147	5481	5845	1082	201	113
Solyc11g071620.1.1	2.875385	4.453166	6.41E-10	2.27E-08	2037	2747	2598	276	174	124
Solyc04g009720.1.1	2.875518	0.558095	9.58E-05	0.000871	95	298	138	10	14	13
Solyc09g075960.1.1	2.876203	0.343797	0.000183	0.001508	93	126	188	10	15	8
Solyc03g118410.2.1	2.879522	3.535695	8.34E-08	1.82E-06	1375	1218	1153	153	102	53
Solyc02g080540.1.1	2.880225	6.008175	0.001552	0.009209	10290	4992	4300	1425	159	66
Solyc02g089550.2.1	2.882517	2.374253	4.34E-07	7.91E-06	469	595	656	38	77	29
Solyc02g083130.2.1	2.882518	1.730722	1.33E-05	0.000157	213	516	435	46	28	13
Solyc09g007150.2.1	2.885229	4.443701	1.12E-09	3.74E-08	978	4260	2907	226	229	127
Solyc06g006110.2.1	2.88683	4.978688	4.38E-11	2.00E-09	2701	3276	4475	284	331	220
Solyc04g050920.2.1	2.888666	3.518634	1.07E-07	2.28E-06	422	2265	1626	89	172	60
Solyc06g082810.2.1	2.888936	2.154364	1.90E-05	0.000214	159	988	557	18	57	42
Solyc01g095670.2.1	2.889901	4.111153	1.14E-08	3.12E-07	2119	1915	1580	221	164	76
Solyc06g054270.2.1	2.890826	4.335365	7.87E-08	1.72E-06	1902	4332	1224	286	183	71
Solyc07g008540.2.1	2.891779	4.046092	8.38E-10	2.90E-08	1323	2569	1910	74	238	137
Solyc07g047850.2.1	2.895564	6.416049	0.000457	0.003275	16054	6526	2828	1596	345	234
Solyc12g087940.1.1	2.899319	1.601691	0.001056	0.006626	485	159	240	52	24	4
Solyc09g007360.2.1	2.901303	2.351839	2.75E-05	0.000296	392	719	648	10	61	59
Solyc02g065280.2.1	2.904174	4.388061	2.50E-05	0.000271	276	4638	3287	89	296	178
Solyc05g007470.2.1	2.904267	5.399479	1.35E-05	0.000159	1168	10248	5316	91	802	313
Solyc03g121300.2.1	2.905349	4.011196	6.79E-09	1.96E-07	1394	2181	1951	213	160	58
Solyc04g081760.2.1	2.906144	1.308124	1.41E-05	0.000164	256	356	220	16	21	24
Solyc04g080580.2.1	2.90966	6.460282	0.000639	0.004353	19321	2863	1980	1185	700	402
Solyc04g076260.1.1	2.910937	1.034122	0.001469	0.008788	79	373	293	1	33	20
Solyc06g050350.2.1	2.912127	1.614219	2.27E-05	0.00025	242	407	390	9	40	30
Solyc06g005970.2.1	2.913469	3.393594	1.89E-07	3.77E-06	365	2059	1534	65	133	80
Solyc11g040340.1.1	2.91377	1.075188	0.001211	0.007456	34	227	461	8	27	19
Solyc09g091510.2.1	2.916369	2.147314	2.58E-06	3.75E-05	292	999	382	56	38	20
Solyc05g056300.2.1	2.91712	5.316634	0.000249	0.001965	7559	2743	1445	697	268	75
Solyc11g070000.1.1	2.918447	0.703208	4.05E-05	0.000417	119	286	167	11	15	14
Solyc01g091050.2.1	2.919924	3.64011	8.61E-05	0.000796	2177	924	634	206	89	29
Solyc08g079550.1.1	2.921764	1.902387	0.000161	0.001351	193	519	593	59	10	20
Solyc04g007210.2.1	2.927168	3.509214	1.92E-09	6.12E-08	1297	1477	1033	90	162	58
Solyc01g087060.2.1	2.936492	0.292686	0.000815	0.005341	200	84	72	8	18	6
Solyc04g006970.2.1	2.93808	3.93325	3.29E-08	7.98E-07	696	4018	1390	164	180	63
Solyc03g111050.2.1	2.940547	3.227466	1.22E-06	1.96E-05	315	1961	1304	39	128	78
Solyc04g072550.1.1	2.94208	-0.76789	0.001614	0.009495	22	91	84	4	7	3
Solyc10g080840.1.1	2.943725	3.038035	1.42E-07	2.92E-06	800	1272	779	109	54	41
Solyc10g055240.1.1	2.944654	2.837403	1.06E-06	1.74E-05	652	1364	566	98	35	40
Solyc05g006050.2.1	2.945106	4.5561	7.85E-08	1.72E-06	921	4675	3304	88	309	212
Solyc03g115980.1.1	2.948304	5.063232	0.001292	0.007877	6426	2187	1204	683	92	47
Solyc10g008510.1.1	2.952902	1.57165	5.92E-05	0.000578	482	227	184	23	27	23
Solyc02g066930.2.1	2.954042	2.857944	1.13E-08	3.08E-07	477	1267	879	50	80	54
Solyc05g005540.2.1	2.956117	2.585954	3.07E-05	0.000327	235	570	1229	68	30	44
Solyc08g079840.1.1	2.957013	3.106994	6.93E-08	1.55E-06	861	1208	874	104	52	53
Solyc04g008500.2.1	2.959294	3.486292	7.26E-10	2.55E-08	729	2155	1256	72	160	66
Solyc06g006040.1.1	2.959487	3.571338	3.62E-10	1.35E-08	1280	1917	940	81	135	87
Solyc01g090680.2.1	2.961894	5.531499	8.05E-09	2.29E-07	6510	5356	3153	519	465	209
Solyc02g065220.2.1	2.971391	4.588887	2.80E-11	1.33E-09	1474	4564	2877	133	323	170
Solyc12g009540.1.1	2.97684	1.331561	0.000196	0.001603	81	278	504	28	30	7
Solyc06g061230.2.1	2.97951	4.804745	5.51E-06	7.31E-05	465	3661	5939	175	343	197
Solyc07g018070.2.1	2.980253	2.935233	0.000279	0.002162	143	1311	1395	16	55	102
Solyc02g077270.2.1	2.98087	3.258492	1.90E-08	4.92E-07	514	1950	1139	48	133	67
Solyc08g079680.2.1	2.98144	2.089651	5.99E-07	1.05E-05	412	510	499	42	49	18
Solyc03g096890.1.1	2.983739	0.071402	0.000513	0.003598	98	61	158	9	10	6
Solyc06g054320.1.1	2.985299	0.449925	6.61E-05	0.000635	76	248	164	11	14	8
Solyc05g054680.1.1	2.986923	1.796302	1.98E-06	3.01E-05	311	529	364	16	57	19
Solyc09g082580.2.1	2.987214	3.007004	6.11E-09	1.77E-07	622	1282	946	48	82	67
Solyc07g020790.2.1	2.987272	1.840066	4.19E-06	5.75E-05	184	630	490	35	46	12
Solyc03g013290.1.1	2.991447	-0.20102	0.000543	0.003779	105	107	63	5	14	3
Solyc08g008270.2.1	2.991736	1.766035	2.69E-05	0.00029	113	593	545	24	55	12
Solyc04g007110.2.1	2.992035	2.304393	6.49E-07	1.13E-05	478	652	544	60	39	22
Solyc07g063860.2.1	2.996582	2.855827	5.33E-07	9.49E-06	340	1317	1022	30	121	42
Solyc06g060830.2.1	2.996783	2.051342	5.86E-06	7.72E-05	135	930	538	26	43	32
Solyc12g010020.1.1	3.002132	13.41832	5.18E-06	6.92E-05	463445	1381480	2023930	160790	77194	32680
Solyc12g008430.1.1	3.005254	-0.0666	0.000722	0.00483	28	213	115	9	8	5
Solyc05g048830.2.1	3.00711	1.417575	3.80E-05	0.000394	125	636	271	8	41	19
Solyc01g095430.2.1	3.008733	6.506504	0.000375	0.002767	18599	3678	3485	1469	400	294
Solyc05g005920.2.1	3.008836	3.785933	6.62E-08	1.49E-06	414	2961	1967	93	136	106
Solyc01g005100.2.1	3.009307	3.231234	1.24E-09	4.08E-08	687	1654	1062	75	78	71

Solyc12g019450.1.1	3.012247	-0.52262	0.001546	0.009182	87	91	40	3	5	7
Solyc06g036310.2.1	3.012722	2.652048	3.26E-07	6.13E-06	375	1623	482	31	85	43
Solyc12g010030.1.1	3.018401	9.913585	3.50E-06	4.90E-05	39056	108127	189514	13140	7274	3256
Solyc01g088250.2.1	3.019729	2.603756	3.01E-06	4.29E-05	202	980	1042	29	87	39
Solyc11g012640.1.1	3.020436	1.923425	1.57E-05	0.000181	111	636	646	24	36	30
Solyc02g086180.2.1	3.023941	5.342485	0.000712	0.004772	4877	9392	869	791	87	66
Solyc02g089700.2.1	3.026133	1.455384	1.24E-05	0.000147	243	433	280	9	49	14
Solyc09g065780.2.1	3.027758	3.164366	0.000338	0.002544	1596	766	373	158	40	15
Solyc04g014650.2.1	3.028949	1.182743	0.000153	0.001292	131	460	250	3	39	16
Solyc04g071620.2.1	3.030715	6.945004	8.18E-06	0.000102	15521	23333	5117	1782	1059	238
Solyc01g094370.2.1	3.032922	4.470106	2.69E-12	1.53E-10	1752	4054	2289	182	293	94
Solyc01g111630.2.1	3.033942	7.234296	0.000676	0.004562	27749	10203	7039	2854	442	228
Solyc06g075500.2.1	3.034445	2.994479	2.49E-07	4.85E-06	511	1387	1005	25	134	49
Solyc12g006630.1.1	3.036292	1.861728	2.90E-05	0.00031	94	688	585	16	49	25
Solyc10g055780.1.1	3.038149	1.239954	6.38E-05	0.000617	350	218	165	27	22	8
Solyc05g006550.2.1	3.038497	3.882844	0.000724	0.004843	3320	450	284	198	104	52
Solyc04g040220.2.1	3.041628	1.387876	0.000144	0.001228	64	484	429	15	46	8
Solyc10g009190.1.1	3.043274	3.039615	1.30E-06	2.08E-05	298	2050	928	34	140	42
Solyc09g007850.2.1	3.043432	7.437721	3.46E-08	8.29E-07	24611	15555	14822	1807	1436	912
Solyc07g055740.1.1	3.04351	2.90542	6.40E-08	1.45E-06	963	1015	532	37	96	52
Solyc10g006970.2.1	3.044042	4.96305	2.49E-05	0.00027	6082	1609	1362	407	183	140
Solyc07g055050.2.1	3.045077	4.94136	1.04E-10	4.36E-09	3645	2605	3601	237	366	163
Solyc08g015830.1.1	3.04552	0.053987	0.000349	0.002611	139	125	64	4	15	6
Solyc12g044260.1.1	3.04562	1.85786	2.62E-06	3.81E-05	228	639	449	13	57	22
Solyc02g077850.2.1	3.048336	3.867702	7.82E-11	3.37E-09	896	2598	1871	82	156	112
Solyc08g081220.1.1	3.053704	1.166883	0.000288	0.002218	292	263	171	37	9	5
Solyc03g071590.2.1	3.056247	3.042535	8.77E-05	0.000807	594	1344	1019	149	33	10
Solyc01g014270.2.1	3.056771	2.132741	3.04E-05	0.000324	409	643	466	70	25	8
Solyc03g031940.2.1	3.059965	2.842062	5.35E-05	0.00053	944	772	611	108	61	7
Solyc04g078740.2.1	3.061129	2.195366	7.31E-07	1.25E-05	274	645	695	34	65	17
Solyc03g121970.1.1	3.061501	3.756465	1.63E-11	8.19E-10	1303	1748	1569	98	165	71
Solyc12g070120.1.1	3.061867	0.168598	0.00027	0.002103	49	223	138	4	11	10
Solyc06g051940.2.1	3.062327	3.290734	1.44E-06	2.28E-05	242	1526	1834	83	113	45
Solyc02g090260.1.1	3.063903	3.211226	3.60E-09	1.09E-07	1007	1346	833	68	67	75
Solyc11g021360.1.1	3.064225	0.814608	0.001513	0.009016	224	134	184	1	41	6
Solyc09g091100.2.1	3.065249	3.473723	6.85E-10	2.41E-08	1359	1458	917	98	101	64
Solyc05g050090.2.1	3.065941	2.800252	4.46E-07	8.09E-06	421	880	1124	25	78	61
Solyc04g051470.1.1	3.070105	0.281814	0.000578	0.003984	24	252	180	8	16	5
Solyc01g109790.2.1	3.070282	4.215485	5.83E-11	2.58E-09	920	4168	2120	152	195	101
Solyc06g061090.2.1	3.070526	1.020024	2.92E-05	0.000312	131	310	271	6	29	14
Solyc07g065500.1.1	3.072849	4.169132	0.000354	0.002635	3535	919	764	295	39	64
Solyc09g072690.1.1	3.073354	3.149136	1.88E-06	2.88E-05	583	874	1537	24	92	88
Solyc02g078950.2.1	3.075063	2.162057	3.38E-06	4.75E-05	156	718	761	40	36	27
Solyc05g053550.2.1	3.076086	2.79181	1.81E-05	0.000205	327	1778	657	75	95	8
Solyc01g090790.2.1	3.077745	5.099919	8.80E-12	4.61E-10	3831	5104	2944	254	480	136
Solyc01g009570.2.1	3.077915	1.887155	6.55E-07	1.14E-05	251	452	566	30	34	22
Solyc03g025430.1.1	3.078878	0.0116	0.000412	0.002999	105	195	51	12	6	4
Solyc06g083580.2.1	3.079816	1.796829	0.000748	0.004972	356	444	370	65	12	2
Solyc01g094700.2.1	3.080682	3.008114	5.23E-05	0.000519	977	1185	589	139	32	13
Solyc11g069190.1.1	3.080815	3.923996	1.39E-09	4.55E-08	744	2348	2415	92	134	122
Solyc06g074110.2.1	3.082118	3.089048	1.49E-07	3.03E-06	284	1771	1222	60	105	43
Solyc01g094720.2.1	3.085717	2.808449	9.09E-09	2.55E-07	521	1257	775	64	48	46
Solyc03g116160.1.1	3.08894	2.174997	4.51E-07	8.16E-06	535	669	338	23	43	37
Solyc10g077040.1.1	3.089954	5.892483	4.21E-08	9.90E-07	8252	6166	4825	737	379	250
Solyc10g009080.2.1	3.090113	2.652586	1.11E-06	1.81E-05	638	648	790	59	24	51
Solyc10g055390.1.1	3.092948	1.372196	0.001381	0.008348	130	451	362	0	37	24
Solyc05g007130.2.1	3.094808	1.900996	6.71E-06	8.66E-05	110	771	549	32	36	19
Solyc04g014730.2.1	3.096961	4.019049	2.77E-05	0.000298	303	2566	3161	47	321	65
Solyc05g007790.2.1	3.098528	-0.25841	0.001261	0.007709	131	80	40	5	13	2
Solyc03g120850.2.1	3.099545	5.684385	4.04E-10	1.50E-08	2549	8763	7699	263	500	417
Solyc12g095810.1.1	3.103041	-0.22413	0.00168	0.009818	24	186	108	1	14	5
Solyc01g099750.2.1	3.104034	4.135565	2.55E-11	1.22E-09	1529	2694	1999	73	220	126
Solyc12g014490.1.1	3.105176	2.276383	7.55E-05	0.00071	67	1175	700	28	36	42
Solyc06g071500.2.1	3.107377	4.160305	3.43E-07	6.41E-06	382	4643	2277	101	247	92
Solyc01g006400.2.1	3.108557	10.20386	3.54E-06	4.94E-05	29093	146785	248145	12871	8682	5503
Solyc08g081770.2.1	3.109401	3.134789	9.27E-09	2.59E-07	1052	928	903	57	118	40
Solyc01g109150.2.1	3.109643	1.488802	0.000175	0.001454	100	353	533	37	25	4
Solyc07g041920.2.1	3.110167	6.860241	0.001557	0.009231	19039	3844	11142	2150	103	256
Solyc01g105050.2.1	3.110227	4.167598	1.41E-08	3.80E-07	2383	1715	1694	213	147	60
Solyc12g096780.1.1	3.112399	3.386552	4.85E-06	6.55E-05	494	1407	1826	51	211	20
Solyc06g074190.2.1	3.113561	2.990602	5.68E-07	1.00E-05	245	1899	1015	40	114	41
Solyc02g085250.2.1	3.113744	2.091834	1.48E-07	3.02E-06	252	812	518	27	41	29
Solyc06g073910.2.1	3.11432	0.182469	0.000119	0.001049	51	215	146	7	14	5
Solyc07g007250.2.1	3.114379	10.62975	1.90E-05	0.000214	65697	186770	307213	23473	4852	6196
Solyc02g068600.2.1	3.115282	2.060425	2.25E-06	3.34E-05	211	775	564	14	64	24
Solyc02g080310.2.1	3.11563	0.852345	0.000209	0.001691	55	233	344	9	12	17
Solyc04g072850.2.1	3.116064	3.024584	7.63E-08	1.68E-06	310	1456	1278	68	84	39
Solyc03g005030.2.1	3.119333	3.099299	4.14E-08	9.76E-07	617	1372	1090	28	90	76
Solyc01g073760.2.1	3.120253	1.793218	4.98E-07	8.96E-06	199	681	413	24	42	16
Solyc12g008980.1.1	3.12034	3.188511	4.54E-06	6.17E-05	928	998	1119	128	23	43
Solyc01g073750.2.1	3.123067	5.800468	7.42E-12	3.95E-10	3566	11354	6198	319	569	394
Solyc10g009170.1.1	3.12438	0.255063	0.000114	0.001012	134	188	81	9	16	3
Solyc11g065220.1.1	3.12654	-0.44108	0.001174	0.007269	58	126	68	2	15	1
Solyc06g008740.2.1	3.12705	2.652085	8.27E-06	0.000103	314	1127	892	13	123	27

Solyc09g010600.1.1	3.129405	-0.63077	0.000619	0.00423	42	71	94	5	7	2
Solyc08g078760.1.1	3.129633	2.262865	0.000336	0.002537	36	1576	471	39	59	17
Solyc02g092970.2.1	3.132235	1.301187	4.02E-05	0.000414	164	292	384	31	9	12
Solyc08g076650.2.1	3.137619	5.57912	1.51E-06	2.37E-05	883	11060	7186	173	669	307
Solyc03g112580.2.1	3.139716	2.426623	1.54E-07	3.12E-06	462	720	677	20	55	45
Solyc04g078470.2.1	3.140212	1.534802	4.95E-06	6.66E-05	158	404	459	27	17	18
Solyc05g015510.2.1	3.140407	5.059746	2.16E-14	1.74E-12	3134	5076	3571	187	375	212
Solyc05g008270.2.1	3.143763	2.613627	1.38E-07	2.85E-06	264	1112	912	48	71	25
Solyc02g090100.1.1	3.144281	4.08687	6.29E-10	2.23E-08	1634	2486	1831	190	162	46
Solyc07g045540.2.1	3.145452	2.952698	0.00031	0.002361	1268	487	589	130	36	7
Solyc11g071800.1.1	3.148329	3.360592	6.85E-08	1.54E-06	348	2105	1509	51	139	55
Solyc07g054210.2.1	3.148791	5.116909	7.63E-11	3.29E-09	4792	3378	3037	301	319	172
Solyc04g078520.2.1	3.149535	0.903116	3.16E-05	0.000336	271	166	144	12	20	10
Solyc03g118370.2.1	3.152109	2.007519	8.73E-05	0.000805	629	352	273	53	35	5
Solyc02g068140.2.1	3.152851	2.970536	7.72E-08	1.70E-06	752	959	959	91	68	21
Solyc07g041910.2.1	3.156344	6.08621	0.000754	0.00501	11156	2187	6592	1201	93	142
Solyc05g008350.2.1	3.157373	6.050193	1.22E-11	6.27E-10	4413	10554	9086	603	584	315
Solyc05g013380.2.1	3.161848	6.675891	0.000105	0.000947	20233	6321	3742	1505	405	301
Solyc05g013150.2.1	3.163115	1.640933	4.03E-07	7.41E-06	197	602	357	20	30	19
Solyc11g069990.1.1	3.168575	2.203669	5.17E-06	6.90E-05	233	946	573	12	82	20
Solyc01g007130.2.1	3.175113	2.68451	2.01E-05	0.000225	206	1215	1021	10	89	49
Solyc01g067460.1.1	3.176174	1.284808	3.10E-05	0.000329	313	283	200	31	16	6
Solyc07g062040.2.1	3.182476	0.096557	0.000736	0.004905	99	175	94	1	22	3
Solyc09g010220.2.1	3.184208	0.920476	9.37E-05	0.000855	276	167	146	21	17	4
Solyc10g045240.1.1	3.185213	2.416877	5.94E-06	7.81E-05	388	860	674	63	55	6
Solyc02g081600.2.1	3.186149	1.707044	0.001294	0.007889	102	677	476	2	81	5
Solyc03g119150.2.1	3.186495	3.77711	9.15E-06	0.000113	2426	1071	710	186	78	35
Solyc05g005950.2.1	3.187918	4.815294	7.40E-06	9.43E-05	2460	4990	2822	467	95	41
Solyc12g088840.1.1	3.192087	4.880487	5.32E-15	4.58E-13	2440	4871	3344	227	253	166
Solyc01g090330.1.1	3.192573	2.313475	0.00014	0.001204	49	996	887	19	46	41
Solyc01g097000.2.1	3.193502	0.75758	7.34E-05	0.000696	102	235	251	20	6	8
Solyc09g015080.2.1	3.195983	1.677711	1.95E-06	2.96E-05	154	460	529	19	27	22
Solyc08g082660.2.1	3.197371	1.317313	0.000491	0.003472	494	114	104	29	13	10
Solyc06g008030.2.1	3.199547	6.007559	3.05E-10	1.18E-08	8521	8343	4842	617	507	286
Solyc01g005870.1.1	3.200158	5.541988	6.04E-10	2.16E-08	4520	5757	5732	162	750	216
Solyc09g014520.2.1	3.206268	7.748634	0.001078	0.006748	48880	5961	4931	3280	777	483
Solyc04g009420.2.1	3.211271	0.767962	1.84E-05	0.000209	117	296	199	11	24	4
Solyc06g054240.2.1	3.211702	0.116009	0.000276	0.002141	60	164	154	10	13	1
Solyc12g042100.1.1	3.21354	-0.97653	0.001348	0.008177	61	69	29	5	4	1
Solyc07g007150.1.1	3.213803	2.892045	5.35E-10	1.93E-08	789	1028	745	50	66	44
Solyc11g069270.1.1	3.214677	5.076497	5.82E-14	4.30E-12	2720	5079	4261	270	227	211
Solyc05g053190.2.1	3.217453	2.104471	1.39E-06	2.21E-05	161	850	629	20	60	19
Solyc10g008740.2.1	3.217894	5.63952	2.80E-10	1.09E-08	5221	6123	5697	558	277	214
Solyc11g065320.1.1	3.218637	4.185434	5.80E-10	2.08E-08	744	3200	2894	93	227	94
Solyc11g073020.1.1	3.218943	3.884526	6.19E-09	1.79E-07	2179	1708	962	139	140	50
Solyc06g083040.2.1	3.219331	6.152717	1.81E-09	5.79E-08	4824	7803	11994	714	605	250
Solyc03g115200.2.1	3.220288	2.358999	0.00069	0.004637	68	755	1072	4	52	51
Solyc01g091530.2.1	3.220332	2.618021	2.41E-07	4.72E-06	402	742	992	46	28	48
Solyc02g079980.2.1	3.224798	2.259078	0.000138	0.001191	827	280	327	69	16	13
Solyc08g045780.2.1	3.227198	2.46407	2.47E-08	6.21E-07	358	929	728	25	52	40
Solyc03g093330.2.1	3.228109	0.630225	4.43E-05	0.000449	71	200	267	8	14	10
Solyc01g010970.2.1	3.228227	0.268863	0.001442	0.008662	49	241	157	0	20	7
Solyc03g078150.2.1	3.22903	2.500176	0.000309	0.002354	47	1951	505	20	37	55
Solyc09g083290.2.1	3.231808	4.179045	1.01E-10	4.28E-09	1619	3479	1613	111	268	51
Solyc06g036390.1.1	3.238216	1.601947	0.001413	0.008505	644	119	93	44	14	5
Solyc09g063130.2.1	3.239466	7.405717	0.000212	0.001713	31026	10582	9576	2768	464	260
Solyc01g094020.2.1	3.247262	3.394021	8.76E-05	0.000807	2072	493	502	141	62	20
Solyc02g070540.2.1	3.251683	3.158429	3.62E-06	5.04E-05	206	1432	1702	27	106	61
Solyc07g006870.2.1	3.255465	2.053392	1.11E-06	1.81E-05	139	937	555	32	40	17
Solyc05g005480.2.1	3.256713	4.60891	1.10E-13	7.77E-12	2193	4645	2200	215	182	115
Solyc09g091880.2.1	3.257853	1.118398	0.000228	0.001818	31	508	321	7	20	17
Solyc03g114450.2.1	3.258064	4.913965	4.61E-11	2.09E-09	1179	6615	4062	231	278	139
Solyc10g076790.1.1	3.25841	1.198738	0.00112	0.00699	268	293	207	0	15	28
Solyc02g090430.2.1	3.258489	6.142085	3.81E-05	0.000395	11779	4677	5254	1023	400	80
Solyc09g007380.2.1	3.259395	-0.72221	0.000666	0.004507	76	55	52	4	6	2
Solyc05g010710.1.1	3.262012	1.016166	2.07E-05	0.00023	192	244	245	4	28	12
Solyc11g042640.1.1	3.264628	5.030541	3.86E-07	7.13E-06	5449	2633	2132	403	166	103
Solyc11g010690.1.1	3.269843	3.510424	3.46E-11	1.61E-09	640	2170	1497	76	118	52
Solyc04g008060.2.1	3.276849	1.795351	7.51E-06	9.54E-05	237	542	468	5	44	25
Solyc03g118630.2.1	3.278501	-0.07498	0.00034	0.002556	84	142	96	1	8	9
Solyc02g080600.1.1	3.287526	2.170363	5.61E-08	1.28E-06	532	657	364	25	34	31
Solyc07g018140.2.1	3.288072	1.955634	4.10E-05	0.00042	562	422	276	54	12	10
Solyc06g053140.2.1	3.288824	4.230278	1.67E-10	6.71E-09	2117	2243	2016	193	149	54
Solyc06g071320.2.1	3.292116	-0.20246	0.000158	0.001327	68	180	65	7	4	5
Solyc06g073090.2.1	3.292147	7.438991	0.000565	0.003905	36915	7943	5427	2710	420	305
Solyc06g051200.2.1	3.296708	4.259642	1.25E-12	7.53E-11	1755	2949	2143	174	168	63
Solyc04g077780.2.1	3.29739	6.511209	5.53E-07	9.82E-06	15129	8249	5563	1080	533	255
Solyc05g009170.1.1	3.298216	1.244913	0.001305	0.007946	60	569	313	0	35	16
Solyc07g042190.2.1	3.302077	5.75809	9.50E-07	1.58E-05	1296	10398	9315	131	771	300
Solyc02g092220.1.1	3.303841	0.118631	0.001136	0.007076	54	176	156	0	17	6
Solyc03g006360.2.1	3.308309	2.913534	2.54E-05	0.000276	260	2738	360	66	90	11
Solyc05g009470.2.1	3.3087	5.492813	2.77E-13	1.86E-11	4336	5676	5612	288	526	167
Solyc09g074630.2.1	3.312534	-0.24917	0.000201	0.001632	38	116	135	7	6	3
Solyc11g009020.1.1	3.31303	2.44237	0.000131	0.001139	968	358	322	78	22	8

Solyc11g009060.1.1	3.313046	3.870581	5.44E-07	9.68E-06	2737	1150	609	128	109	61
Solyc11g007850.1.1	3.315115	4.613192	3.80E-07	7.05E-06	845	6075	3013	40	310	170
Solyc08g005560.2.1	3.317237	4.34774	3.14E-13	2.08E-11	1983	3463	1932	185	148	81
Solyc08g067970.2.1	3.317431	1.05589	8.02E-05	0.000749	57	323	380	10	10	18
Solyc08g078840.2.1	3.317859	0.03548	6.63E-05	0.000636	70	170	121	7	12	2
Solyc08g080780.2.1	3.318849	3.400184	9.95E-08	2.13E-06	1234	1148	1152	130	57	25
Solyc09g091060.2.1	3.31912	0.128688	0.000349	0.002611	27	201	175	4	7	9
Solyc02g067120.2.1	3.325626	3.043171	2.31E-10	9.08E-09	634	1671	810	68	57	39
Solyc03g083100.2.1	3.329167	4.463819	4.65E-15	4.04E-13	2096	3104	2580	159	178	109
Solyc04g064720.2.1	3.332063	3.739147	6.96E-11	3.03E-09	741	2203	2001	117	86	61
Solyc07g064160.2.1	3.333022	5.450157	1.50E-09	4.87E-08	2807	12422	2805	444	299	141
Solyc02g076980.2.1	3.335818	2.69734	2.06E-05	0.00023	591	1529	386	12	132	12
Solyc09g097780.2.1	3.339629	5.951618	2.81E-10	1.09E-08	5945	12254	4939	645	445	170
Solyc09g082760.2.1	3.344714	5.451984	4.22E-11	1.94E-09	2595	5467	7525	173	418	289
Solyc08g083130.2.1	3.344742	1.504028	2.69E-05	0.00029	133	902	164	25	29	5
Solyc12g040790.1.1	3.346787	1.883087	0.000117	0.001035	52	890	550	8	58	15
Solyc04g077510.2.1	3.349615	2.664569	0.001112	0.006946	89	1023	1283	1	85	46
Solyc03g013240.2.1	3.350707	2.007935	1.31E-06	2.10E-05	461	624	324	38	40	6
Solyc01g067290.2.1	3.353989	2.528445	9.43E-09	2.63E-07	463	745	812	50	34	27
Solyc07g063230.2.1	3.354234	3.539045	5.35E-09	1.57E-07	769	2011	1533	26	157	64
Solyc03g120640.2.1	3.355311	6.772407	0.000161	0.001351	20909	6436	5553	1654	262	169
Solyc06g072700.2.1	3.356742	1.108221	0.00016	0.001345	193	408	185	32	5	3
Solyc07g061800.2.1	3.358378	-0.4171	0.000541	0.003768	70	170	31	6	3	4
Solyc06g076220.2.1	3.358899	1.672261	1.95E-06	2.96E-05	121	481	563	17	24	20
Solyc03g033610.1.1	3.360554	-0.03348	0.000921	0.005932	88	135	107	0	18	3
Solyc09g031750.2.1	3.361233	4.995668	2.69E-15	2.44E-13	2697	5923	3259	220	327	115
Solyc02g080510.1.1	3.362182	4.691063	2.82E-05	0.000302	278	6561	3872	47	404	119
Solyc08g076820.2.1	3.36501	-0.70416	0.00158	0.009337	83	71	36	2	2	6
Solyc01g100530.2.1	3.366108	2.079751	4.30E-08	1.01E-06	254	754	564	35	32	16
Solyc09g074850.2.1	3.366689	-0.05835	0.000859	0.005586	116	73	107	13	2	2
Solyc11g068680.1.1	3.367378	1.510614	1.07E-05	0.000129	244	377	368	4	23	26
Solyc02g071210.2.1	3.367867	6.008803	4.62E-11	2.09E-09	2709	14631	8287	381	654	285
Solyc10g009270.2.1	3.370574	2.392516	3.77E-06	5.23E-05	126	1169	792	22	74	17
Solyc01g109040.2.1	3.370651	3.557104	0.001333	0.008095	2644	345	324	182	24	15
Solyc07g062550.2.1	3.371966	-0.06675	8.78E-05	0.000808	81	155	95	2	10	6
Solyc02g077910.2.1	3.372543	1.073398	1.41E-05	0.000164	110	406	273	4	28	11
Solyc10g083440.1.1	3.377003	1.320642	0.000759	0.005034	30	799	248	2	39	12
Solyc01g086920.2.1	3.379381	2.514718	1.04E-06	1.71E-05	741	574	563	67	30	13
Solyc02g084640.2.1	3.380213	2.879556	8.04E-06	0.000101	130	1877	1024	20	105	32
Solyc02g091140.2.1	3.384714	3.710026	1.04E-05	0.000126	128	2745	2342	44	130	84
Solyc11g007540.1.1	3.386357	0.883934	0.000208	0.001686	341	124	86	16	18	3
Solyc01g111310.2.1	3.390152	1.66361	0.000122	0.001071	168	463	513	1	47	18
Solyc02g091990.2.1	3.396737	1.580031	2.38E-07	4.66E-06	227	469	379	18	35	8
Solyc05g009330.2.1	3.397832	1.786892	6.54E-06	8.48E-05	85	606	616	15	28	22
Solyc10g009320.2.1	3.400766	-0.57394	0.001594	0.009402	87	97	34	0	10	3
Solyc07g062530.2.1	3.402506	-0.20713	0.000126	0.001101	36	196	95	5	8	3
Solyc11g007160.1.1	3.403213	0.94151	0.000343	0.002578	118	330	253	0	28	11
Solyc02g089880.2.1	3.404206	-0.20251	0.000136	0.001173	81	137	79	9	3	3
Solyc12g099650.1.1	3.406	5.642509	0.001616	0.009502	11482	1291	1241	775	94	52
Solyc07g061790.2.1	3.406128	4.55525	8.16E-10	2.84E-08	964	4891	3349	232	124	86
Solyc01g049880.2.1	3.409926	2.965359	9.35E-10	3.19E-08	902	950	812	61	71	23
Solyc01g096370.2.1	3.414775	3.772257	1.13E-09	3.78E-08	446	2966	2004	67	140	64
Solyc10g054840.1.1	3.417673	3.388434	0.000328	0.002488	2121	617	385	154	30	10
Solyc11g020960.1.1	3.418081	1.883033	0.000168	0.001402	45	471	832	13	57	9
Solyc04g057980.2.1	3.42163	1.456972	7.25E-05	0.000688	482	205	154	31	9	9
Solyc01g090560.2.1	3.42183	2.664241	2.01E-06	3.03E-05	138	1124	1167	27	80	23
Solyc03g120520.1.1	3.422081	0.099699	0.000384	0.002827	22	245	148	2	12	6
Solyc03g119930.1.1	3.425582	4.615237	1.09E-07	2.31E-06	2908	2905	2542	275	184	26
Solyc02g070220.2.1	3.42576	1.270139	1.95E-05	0.000219	112	409	370	6	12	23
Solyc09g008320.2.1	3.426746	1.335037	5.40E-06	7.17E-05	240	342	293	25	21	3
Solyc03g026210.2.1	3.429537	2.731658	4.83E-10	1.76E-08	534	984	865	45	39	37
Solyc09g008860.2.1	3.430234	-0.04254	0.000411	0.002997	48	103	175	9	2	5
Solyc11g007900.1.1	3.432734	5.16383	1.51E-12	8.97E-11	2327	5937	5043	119	426	176
Solyc05g024160.2.1	3.433951	1.228507	0.000648	0.004407	71	531	321	0	26	18
Solyc04g015750.2.1	3.435978	5.508019	1.54E-05	0.000178	8039	4476	2055	600	208	50
Solyc01g096220.2.1	3.436339	0.114422	0.001254	0.00768	43	172	173	0	7	11
Solyc06g084250.2.1	3.438451	1.964529	3.12E-08	7.61E-07	217	832	459	22	29	21
Solyc07g056250.2.1	3.440622	0.508193	8.18E-05	0.000762	57	343	158	4	22	3
Solyc07g006600.1.1	3.446673	-0.03415	6.47E-05	0.000623	67	177	106	2	8	7
Solyc04g082050.2.1	3.450023	2.567908	6.41E-06	8.33E-05	399	1248	634	4	84	32
Solyc09g065910.1.1	3.455369	6.055583	3.01E-06	4.29E-05	11529	5182	4175	821	265	130
Solyc03g098060.1.1	3.457635	0.747173	9.16E-06	0.000113	116	343	170	4	23	6
Solyc12g008900.1.1	3.460776	4.048253	1.19E-05	0.000142	1560	1916	2249	231	64	10
Solyc12g049510.1.1	3.464037	2.188583	2.93E-07	5.57E-06	317	698	637	31	13	32
Solyc01g066640.2.1	3.470488	-0.44674	0.000111	0.000991	81	99	61	4	7	2
Solyc09g091050.2.1	3.479797	4.394301	6.77E-15	5.76E-13	1415	3558	2860	123	202	78
Solyc02g078040.2.1	3.480157	1.585397	0.000147	0.001254	42	410	652	7	19	25
Solyc04g082000.2.1	3.480295	4.580704	0.001031	0.006505	5778	438	392	310	66	46
Solyc03g112390.2.1	3.480993	-0.54416	0.001069	0.006697	51	84	92	0	11	2
Solyc02g071700.2.1	3.485379	4.340884	6.64E-10	2.34E-08	704	3620	3453	104	234	63
Solyc04g005250.2.1	3.485856	2.284973	9.13E-07	1.52E-05	154	1358	524	18	61	17
Solyc06g050900.2.1	3.486637	0.964084	0.00115	0.007146	123	391	221	0	41	2
Solyc12g014030.1.1	3.486902	0.154596	3.96E-05	0.000409	108	102	150	9	6	4
Solyc12g007070.1.1	3.491798	3.577189	1.72E-12	1.01E-10	1064	1828	1416	98	70	46

Solyc08g083320.2.1	3.49229	7.551895	3.43E-10	1.30E-08	13098	44943	16716	1770	1098	548
Solyc07g056140.2.1	3.493166	5.976972	1.25E-06	2.01E-05	7693	9608	5001	831	206	84
Solyc10g079750.1.1	3.497637	0.65605	0.000121	0.001064	58	226	284	12	17	1
Solyc08g083230.1.1	3.500458	2.592509	5.04E-09	1.49E-07	272	1198	862	34	57	22
Solyc09g083150.2.1	3.502323	4.57146	5.30E-06	7.05E-05	3769	2073	1799	312	48	48
Solyc08g006690.1.1	3.505787	2.802049	4.71E-07	8.50E-06	1028	668	576	54	73	9
Solyc02g093420.2.1	3.51063	1.03731	6.03E-05	0.000588	314	209	136	2	19	15
Solyc10g084150.1.1	3.510913	1.89921	1.16E-05	0.000138	155	715	560	4	55	15
Solyc10g009290.1.1	3.510981	4.526061	4.24E-09	1.27E-07	653	3943	4231	79	242	113
Solyc05g013560.2.1	3.511088	3.601062	1.84E-08	4.77E-07	1841	1102	987	118	72	29
Solyc06g083070.2.1	3.513377	3.869806	1.23E-06	1.98E-05	2546	1075	948	147	107	19
Solyc06g065670.2.1	3.514973	2.584978	2.28E-08	5.79E-07	838	713	462	50	30	24
Solyc04g054370.1.1	3.518848	1.745479	3.62E-08	8.66E-07	281	497	419	23	17	17
Solyc02g064770.2.1	3.521236	5.148543	1.62E-16	1.73E-14	2983	7168	3381	210	321	129
Solyc07g018240.1.1	3.522543	1.518638	0.000345	0.002587	382	261	286	42	4	2
Solyc05g010240.2.1	3.522638	2.497269	1.95E-06	2.96E-05	919	396	450	47	50	9
Solyc07g006300.2.1	3.526299	2.865007	1.41E-07	2.90E-06	499	1591	746	10	97	34
Solyc05g025600.1.1	3.526481	4.854068	2.37E-06	3.49E-05	5091	2225	1766	350	85	61
Solyc09g009190.2.1	3.527261	5.409191	2.88E-14	2.25E-12	2635	7701	5786	159	423	206
Solyc06g006080.2.1	3.5308	6.05519	2.80E-14	2.21E-12	5577	9248	9050	303	509	366
Solyc08g075490.2.1	3.530959	4.515222	1.06E-11	5.49E-10	2758	3601	1792	210	130	65
Solyc10g008520.2.1	3.537919	6.605004	7.29E-11	3.16E-09	9189	13639	11936	884	667	216
Solyc03g115900.2.1	3.53852	4.431198	0.000226	0.001805	4883	609	643	263	90	26
Solyc05g008950.2.1	3.53948	2.85851	1.05E-05	0.000127	314	1736	873	4	100	36
Solyc05g005980.2.1	3.541151	1.950804	1.77E-06	2.73E-05	255	861	389	43	18	6
Solyc06g062460.2.1	3.543936	3.565464	1.31E-10	5.37E-09	456	2426	1768	64	107	45
Solyc11g072120.1.1	3.544542	1.487915	5.31E-07	9.48E-06	112	659	345	13	20	15
Solyc12g055840.1.1	3.551023	4.468355	8.80E-14	6.39E-12	2995	2561	1929	122	183	92
Solyc09g074840.2.1	3.551677	0.283259	0.00036	0.002677	43	276	153	10	12	0
Solyc11g012130.1.1	3.552873	2.745131	3.75E-06	5.21E-05	98	1911	849	53	45	20
Solyc07g044860.2.1	3.553004	8.421067	0.000326	0.002474	69841	16943	15480	4841	620	312
Solyc01g080910.2.1	3.56357	4.728182	4.14E-14	3.14E-12	1529	4117	4199	147	244	92
Solyc12g038160.1.1	3.563819	2.325907	3.98E-07	7.31E-06	148	1123	734	15	48	26
Solyc07g006570.2.1	3.564864	5.955842	1.60E-10	6.49E-09	2212	10080	11274	392	410	272
Solyc06g074090.2.1	3.564994	3.524862	7.56E-05	0.000711	54	3780	1298	27	117	63
Solyc01g096320.2.1	3.569408	3.255776	0.000394	0.002892	88	4249	312	37	146	11
Solyc03g114160.1.1	3.57185	1.782945	7.82E-05	0.000732	82	1213	240	6	54	8
Solyc12g088320.1.1	3.577766	0.850567	6.38E-06	8.30E-05	264	163	148	12	14	5
Solyc09g048980.2.1	3.583148	-0.40691	0.000121	0.001066	39	99	120	2	6	4
Solyc02g055440.2.1	3.583885	3.413294	8.76E-06	0.000109	1766	958	699	132	35	12
Solyc09g075360.2.1	3.585657	2.206092	1.35E-05	0.000158	543	642	423	59	12	5
Solyc03g031840.2.1	3.590919	3.163463	3.95E-10	1.47E-08	478	1832	1178	26	74	53
Solyc01g109370.2.1	3.594628	1.318203	6.09E-06	7.97E-05	103	486	367	24	14	4
Solyc09g008380.2.1	3.594785	0.572441	0.001435	0.008625	45	99	354	1	4	16
Solyc05g007510.2.1	3.594795	6.083453	4.29E-14	3.25E-12	4582	13901	7832	272	660	284
Solyc06g054570.1.1	3.599559	1.744772	0.000127	0.001111	566	395	137	42	5	6
Solyc03g123410.1.1	3.59973	0.912825	1.17E-05	0.00014	270	170	163	16	13	3
Solyc10g005690.2.1	3.599799	0.454972	0.00018	0.001485	152	166	142	0	17	7
Solyc04g072310.2.1	3.600935	1.292384	2.65E-07	5.11E-06	176	485	264	9	28	7
Solyc10g078610.1.1	3.601985	-0.97797	0.000973	0.006191	80	39	29	1	5	2
Solyc06g073540.2.1	3.604393	5.183945	6.25E-11	2.74E-09	4566	5647	2730	347	161	97
Solyc02g069460.2.1	3.607008	6.800041	9.87E-06	0.000121	19637	7427	7711	1357	270	187
Solyc05g024260.2.1	3.609226	5.034914	2.11E-06	3.16E-05	1419	11088	1989	390	92	54
Solyc12g006520.1.1	3.618637	2.323643	1.25E-06	2.01E-05	150	1311	614	10	59	21
Solyc01g106910.2.1	3.620282	3.983803	6.16E-14	4.54E-12	1285	3331	1498	81	161	43
Solyc07g049440.2.1	3.622841	3.133952	6.62E-10	2.34E-08	783	2226	495	58	70	24
Solyc02g083810.2.1	3.631081	7.31999	1.18E-07	2.47E-06	27716	12210	10684	1506	729	397
Solyc03g062660.2.1	3.637323	0.102922	7.30E-05	0.000693	42	171	176	2	9	6
Solyc04g071650.2.1	3.641633	5.589257	1.70E-11	8.46E-10	1624	10220	7389	206	419	201
Solyc02g069110.2.1	3.644242	4.186942	3.70E-08	8.83E-07	461	2293	3977	54	180	82
Solyc04g005380.2.1	3.650058	1.224048	6.40E-06	8.32E-05	101	767	139	17	15	6
Solyc11g008530.1.1	3.652042	4.848195	1.91E-09	6.12E-08	5174	2748	1354	150	151	154
Solyc01g105680.2.1	3.652659	4.412239	4.67E-16	4.61E-14	1779	3910	2337	95	212	67
Solyc08g065970.2.1	3.655887	3.448728	8.81E-14	6.39E-12	1068	1645	1227	56	96	35
Solyc10g054900.1.1	3.664818	-0.79449	0.001207	0.007439	12	103	92	1	3	4
Solyc08g069230.2.1	3.667972	-0.06464	0.001454	0.008719	154	48	80	8	0	5
Solyc10g055770.1.1	3.67351	1.51152	1.94E-07	3.87E-06	140	571	388	10	17	17
Solyc02g005180.2.1	3.679412	2.257816	4.32E-08	1.01E-06	505	782	445	28	50	6
Solyc01g009080.2.1	3.681257	2.516205	4.04E-05	0.000416	1185	309	216	58	24	9
Solyc07g019650.2.1	3.681615	3.076983	1.27E-09	4.17E-08	426	1736	1145	19	78	43
Solyc01g094790.2.1	3.687658	5.413444	3.34E-07	6.26E-06	1573	9216	6297	35	497	209
Solyc08g067330.1.1	3.689778	1.686749	0.000444	0.003201	752	54	87	18	13	17
Solyc01g058320.2.1	3.691055	2.54578	9.77E-09	2.71E-07	420	1059	725	18	75	12
Solyc12g008830.1.1	3.69433	1.402304	0.000101	0.000913	141	580	314	0	35	11
Solyc07g065860.2.1	3.697834	-0.1396	0.000609	0.004168	26	167	144	0	9	5
Solyc03g044480.2.1	3.697972	2.497554	7.42E-11	3.21E-09	491	1099	547	34	39	18
Solyc06g068440.2.1	3.698796	3.984888	3.48E-10	1.32E-08	461	3713	2298	53	128	76
Solyc02g068760.2.1	3.701114	3.578812	1.75E-05	0.000199	2307	663	668	130	51	10
Solyc12g098370.1.1	3.701514	2.37747	7.42E-07	1.27E-05	190	1106	766	8	65	18
Solyc07g019430.1.1	3.710909	0.743082	1.20E-06	1.94E-05	198	246	137	8	15	4
Solyc06g050840.2.1	3.71392	1.138555	0.000158	0.001327	149	386	283	0	35	5
Solyc03g083050.2.1	3.720598	-0.98354	0.000691	0.004646	44	93	37	5	2	0
Solyc08g061980.2.1	3.725947	3.598146	0.000128	0.001114	209	2354	2246	2	125	71
Solyc01g006690.2.1	3.727107	2.713965	8.77E-08	1.90E-06	705	1019	639	66	15	16

Solyc09g097890.2.1	3.732064	-0.89663	0.000384	0.002829	44	70	63	3	5	0
Solyc03g005900.2.1	3.733525	1.454006	1.65E-07	3.33E-06	392	294	221	15	17	10
Solyc10g078770.1.1	3.736547	1.846932	2.79E-05	0.0003	191	1301	110	27	29	3
Solyc04g016290.2.1	3.738842	1.616266	6.89E-08	1.54E-06	369	422	276	7	26	15
Solyc11g012040.1.1	3.741064	0.622066	0.000255	0.002004	24	306	268	1	18	6
Solyc09g075900.2.1	3.742363	1.129561	4.50E-07	8.15E-06	169	374	261	5	13	14
Solyc02g071610.2.1	3.746251	3.380765	4.29E-11	1.97E-09	1497	1362	725	62	78	27
Solyc07g052980.2.1	3.752437	4.809809	5.23E-17	5.90E-15	2321	3504	4204	129	184	120
Solyc02g079950.2.1	3.760131	7.717123	0.000173	0.001435	43237	9346	10455	2600	281	199
Solyc05g014560.2.1	3.761378	-1.0978	0.000604	0.004139	51	49	45	4	1	1
Solyc01g098640.2.1	3.763105	4.287354	1.15E-11	5.91E-10	2239	2607	2046	170	82	43
Solyc07g041720.1.1	3.7771	2.115552	1.73E-06	2.67E-05	805	250	271	30	23	12
Solyc01g005990.2.1	3.784606	2.139683	3.83E-07	7.08E-06	543	578	410	42	19	5
Solyc09g059550.1.1	3.795095	-0.90849	0.00052	0.003634	63	33	63	4	2	1
Solyc02g030200.1.1	3.799002	-0.42498	0.000253	0.001985	52	156	66	0	5	5
Solyc10g006230.2.1	3.802613	7.563578	0.000555	0.003849	40225	6981	8751	2397	168	119
Solyc07g007280.2.1	3.802664	-0.04691	5.26E-05	0.000521	146	79	76	2	8	4
Solyc02g068060.2.1	3.811522	2.239373	2.70E-10	1.05E-08	504	665	509	17	30	22
Solyc02g070950.1.1	3.820842	2.248872	0.000507	0.003561	1137	136	76	47	12	8
Solyc03g031760.2.1	3.826362	2.798551	2.51E-11	1.21E-09	542	1329	787	34	61	14
Solyc10g007120.2.1	3.832585	2.217774	2.96E-10	1.15E-08	346	703	659	22	35	13
Solyc02g083120.1.1	3.833179	2.328569	2.14E-09	6.76E-08	236	1087	673	15	37	22
Solyc11g008040.1.1	3.834441	-0.77244	0.000754	0.00501	44	58	87	5	0	2
Solyc07g043660.2.1	3.8349	0.623606	0.000107	0.000954	40	334	232	1	7	12
Solyc08g062370.2.1	3.843334	-0.89048	0.000115	0.001017	38	89	59	3	3	1
Solyc12g006460.1.1	3.849667	4.455414	1.35E-09	4.43E-08	506	6374	2625	60	163	100
Solyc07g066150.1.1	3.850289	7.007177	0.000404	0.002948	28124	4509	5235	1550	173	67
Solyc01g096360.2.1	3.858269	3.720019	1.71E-12	1.00E-10	750	2524	1850	30	116	53
Solyc04g081810.2.1	3.85928	0.030002	0.00053	0.003706	182	92	42	0	11	4
Solyc06g082950.2.1	3.870273	6.255172	0.000181	0.001492	16870	2977	2723	873	122	56
Solyc01g095530.2.1	3.870876	1.101042	1.25E-06	2.01E-05	306	301	130	14	14	3
Solyc04g008330.1.1	3.872194	2.984676	8.02E-07	1.36E-05	260	1456	1379	75	29	8
Solyc07g055930.2.1	3.874478	2.629534	1.59E-05	0.000183	155	1680	788	2	64	28
Solyc12g098600.1.1	3.881159	2.566473	1.47E-07	3.01E-06	639	737	703	57	13	10
Solyc05g026100.1.1	3.887505	-0.83916	0.00059	0.004058	22	118	66	0	4	3
Solyc10g018300.1.1	3.902273	6.543035	5.18E-08	1.20E-06	14273	11451	6232	860	332	109
Solyc06g074970.1.1	3.914171	2.308084	2.94E-08	7.22E-07	216	1091	677	8	34	26
Solyc01g091230.2.1	3.918976	0.774463	2.63E-07	5.07E-06	114	285	232	5	11	7
Solyc11g008480.1.1	3.927107	-0.35833	0.000272	0.002117	109	90	53	7	3	0
Solyc04g009980.2.1	3.93005	-0.47502	0.000396	0.002901	24	104	127	0	5	4
Solyc03g093540.1.1	3.931579	1.950198	3.84E-07	7.10E-06	161	1114	366	5	26	21
Solyc03g005770.1.1	3.934152	5.451283	2.17E-06	3.24E-05	8820	1776	2594	394	116	72
Solyc06g053210.2.1	3.934682	-1.11084	0.000567	0.003922	58	59	30	0	5	1
Solyc07g014590.2.1	3.93676	2.938281	3.36E-08	8.09E-07	160	1727	1253	20	56	31
Solyc02g071380.2.1	3.94832	2.016581	0.000172	0.001428	716	560	98	19	44	0
Solyc03g025390.2.1	3.951468	4.545091	1.12E-12	6.80E-11	988	5056	3372	155	92	63
Solyc12g062250.1.1	3.957561	2.561601	1.59E-09	5.15E-08	611	917	623	45	27	8
Solyc06g068620.2.1	3.966737	2.344341	2.15E-08	5.49E-07	316	747	820	6	44	21
Solyc08g079240.2.1	3.967435	3.605328	6.93E-11	3.02E-09	1539	1593	1147	85	26	41
Solyc01g100770.2.1	3.977281	4.67403	1.93E-11	9.54E-10	1984	3650	3777	186	141	27
Solyc08g066840.2.1	3.978708	5.427164	2.42E-06	3.55E-05	662	10351	7028	28	422	172
Solyc07g008570.2.1	3.981666	4.13036	8.74E-09	2.45E-07	270	4754	2504	63	137	47
Solyc05g043330.2.1	3.993673	-0.27072	0.000458	0.00328	136	83	41	5	0	4
Solyc01g008930.2.1	3.995355	1.226089	1.35E-07	2.80E-06	103	554	286	6	14	10
Solyc10g009580.2.1	3.998795	1.458729	8.15E-06	0.000102	411	408	138	25	8	2
Solyc08g080050.2.1	4.003847	5.182766	8.82E-07	1.48E-05	6227	3094	2512	350	91	27
Solyc08g075100.2.1	4.012093	3.317347	9.20E-14	6.66E-12	754	1781	1265	34	40	49
Solyc03g005960.2.1	4.036369	1.865218	6.89E-06	8.84E-05	40	672	730	8	30	11
Solyc05g006440.2.1	4.036609	4.169053	8.13E-13	5.03E-11	1865	2415	2192	129	78	26
Solyc02g070800.2.1	4.042215	6.390985	8.67E-11	3.70E-09	12793	7605	7555	574	358	148
Solyc07g043480.1.1	4.043158	1.536537	2.06E-06	3.09E-05	74	919	279	20	11	5
Solyc06g051460.2.1	4.046695	1.951846	9.84E-06	0.00012	258	645	554	0	28	21
Solyc01g099560.2.1	4.050677	-0.09135	5.59E-06	7.39E-05	55	184	112	2	5	4
Solyc07g043390.2.1	4.056443	6.044005	3.86E-12	2.14E-10	3663	20161	4845	400	261	164
Solyc04g079560.2.1	4.056645	1.159312	0.000173	0.001435	65	361	424	0	30	3
Solyc03g117800.2.1	4.063265	3.97003	3.18E-09	9.70E-08	349	5528	1310	43	105	55
Solyc07g043400.1.1	4.068589	3.087766	1.07E-09	3.58E-08	482	2441	696	46	59	9
Solyc03g115540.1.1	4.070578	3.045503	1.20E-13	8.46E-12	997	1349	672	33	48	24
Solyc11g006230.1.1	4.071025	-0.38967	0.000517	0.003619	64	214	23	0	9	1
Solyc05g011890.1.1	4.072839	2.607122	7.14E-08	1.58E-06	178	2011	533	11	51	19
Solyc03g005760.1.1	4.072887	7.055359	1.02E-10	4.32E-09	22014	9696	11383	727	733	262
Solyc01g095930.2.1	4.074268	-0.18025	0.000578	0.003984	22	215	113	0	2	7
Solyc06g005220.2.1	4.076027	-0.97422	0.000319	0.002424	22	113	54	0	5	1
Solyc01g009260.1.1	4.077162	-0.84089	0.000866	0.005625	10	96	96	0	3	3
Solyc04g054740.2.1	4.090399	5.162076	6.29E-06	8.20E-05	6385	3366	2010	356	43	25
Solyc11g071240.1.1	4.097948	-0.56522	0.000239	0.001897	111	53	41	3	1	3
Solyc01g058170.2.1	4.106283	-0.09936	2.99E-06	4.27E-05	66	133	131	3	6	2
Solyc01g098500.2.1	4.107301	4.110766	5.61E-14	4.15E-12	679	3406	2806	63	114	44
Solyc04g082010.1.1	4.107777	6.80284	0.001078	0.006751	27814	1982	1951	1156	101	49
Solyc10g078260.1.1	4.108454	2.599757	9.54E-12	4.98E-10	335	1468	677	26	28	19
Solyc03g119960.2.1	4.109292	1.690627	4.09E-06	5.61E-05	121	630	519	1	19	18
Solyc08g074650.2.1	4.111847	3.091458	3.96E-08	9.37E-07	406	954	1750	65	28	10
Solyc10g078250.1.1	4.114974	0.798922	7.89E-07	1.34E-05	76	360	245	10	9	2
Solyc07g053200.2.1	4.11637	2.94033	1.66E-11	8.32E-10	327	1685	1096	30	52	16

Solyc01g079470.2.1	4.117295	3.233969	9.93E-10	3.37E-08	1181	780	1203	17	94	18
Solyc01g090730.2.1	4.124022	-0.18563	0.000122	0.001074	29	145	150	0	6	4
Solyc03g020070.2.1	4.138379	3.739299	0.000455	0.003267	3167	377	335	135	13	5
Solyc09g008670.2.1	4.143219	12.14973	6.11E-07	1.07E-05	166195	442270	1049170	37666	8923	5891
Solyc01g079620.2.1	4.147631	-0.07074	6.14E-05	0.000596	75	210	76	0	4	6
Solyc02g065780.1.1	4.149943	0.234878	7.53E-05	0.000709	23	207	219	2	12	1
Solyc06g082940.2.1	4.165186	7.314979	3.26E-05	0.000346	29546	9208	11388	1555	139	94
Solyc08g081890.2.1	4.167646	1.781889	4.59E-07	8.29E-06	319	509	427	1	33	10
Solyc01g087770.2.1	4.170899	1.371105	7.70E-05	0.000723	603	75	66	5	15	10
Solyc03g093180.1.1	4.172824	-2.1603	0.00158	0.009337	18	35	15	0	2	0
Solyc04g077570.1.1	4.194787	-0.39125	2.65E-05	0.000286	104	54	79	3	3	2
Solyc10g009380.2.1	4.195264	1.368904	1.41E-05	0.000165	41	660	384	9	23	1
Solyc10g006900.2.1	4.196941	4.401009	2.85E-08	7.01E-07	4304	1264	1003	135	67	38
Solyc03g112760.2.1	4.205957	3.919618	1.11E-14	9.24E-13	1076	2297	2316	29	118	40
Solyc06g068040.2.1	4.220516	2.499443	7.32E-11	3.17E-09	531	555	889	12	39	16
Solyc10g076710.1.1	4.225869	3.809206	5.41E-11	2.42E-09	398	2964	2355	27	100	41
Solyc05g056050.2.1	4.228751	3.994865	0.000109	0.000976	3996	172	331	112	46	20
Solyc12g008580.1.1	4.247702	0.501621	6.21E-06	8.11E-05	59	307	193	1	14	2
Solyc03g019790.2.1	4.250823	1.272365	9.98E-09	2.77E-07	249	388	246	14	8	4
Solyc04g016220.2.1	4.251095	1.700357	7.26E-09	2.08E-07	326	521	350	16	20	2
Solyc08g076980.2.1	4.259777	3.651878	6.01E-09	1.75E-07	258	1366	3060	41	62	37
Solyc07g053630.2.1	4.267946	4.543895	8.63E-18	1.06E-15	1143	5097	3242	72	116	71
Solyc07g052950.2.1	4.273882	-0.61935	0.000985	0.006265	24	155	73	6	0	0
Solyc10g012050.2.1	4.275822	3.617643	1.14E-10	4.73E-09	305	2112	2429	43	73	25
Solyc01g096280.1.1	4.276743	0.574194	1.05E-05	0.000127	70	331	189	0	13	4
Solyc02g077720.2.1	4.281936	1.388013	3.23E-09	9.82E-08	142	655	276	9	12	7
Solyc08g005770.2.1	4.301096	-0.48458	0.000626	0.004271	14	53	174	0	4	3
Solyc10g055520.1.1	4.306525	-0.91831	8.03E-05	0.00075	45	75	59	0	3	2
Solyc01g006330.2.1	4.308536	1.431532	0.000454	0.00326	616	96	78	24	2	1
Solyc03g113060.2.1	4.325773	3.753059	8.52E-17	9.38E-15	844	2248	2091	43	48	47
Solyc03g044590.2.1	4.327254	-0.33313	0.000158	0.001334	28	184	98	0	2	5
Solyc10g080340.1.1	4.366076	-0.59803	5.16E-05	0.000513	44	104	86	0	3	3
Solyc02g071020.2.1	4.376267	6.186242	9.16E-06	0.000113	14252	5334	3745	609	71	36
Solyc06g019170.2.1	4.381516	2.93632	2.37E-06	3.49E-05	42	2754	782	30	36	14
Solyc07g051820.2.1	4.385191	4.043399	1.63E-07	3.29E-06	213	4540	2448	15	136	37
Solyc09g014740.2.1	4.387049	2.828853	2.97E-14	2.32E-12	550	1595	707	19	43	14
Solyc09g074450.1.1	4.420977	0.185008	0.00021	0.001696	73	265	109	10	0	0
Solyc08g005960.1.1	4.425087	3.427516	4.45E-08	1.04E-06	976	1295	1619	6	20	66
Solyc02g077430.2.1	4.431682	5.324008	3.88E-15	3.43E-13	4144	6224	4564	174	221	33
Solyc02g081920.2.1	4.433707	3.392092	2.80E-09	8.65E-08	302	2375	1680	13	89	16
Solyc06g008500.2.1	4.43816	0.195172	0.000203	0.001651	100	129	167	10	0	0
Solyc08g077060.2.1	4.44547	1.521877	2.34E-07	4.58E-06	280	472	316	10	21	0
Solyc02g077420.2.1	4.485108	5.168356	2.07E-18	2.70E-16	3674	4317	4982	110	213	49
Solyc10g086250.1.1	4.491632	2.445215	0.000783	0.005167	7	1586	823	0	43	14
Solyc04g015620.2.1	4.492437	2.814376	6.00E-05	0.000585	1779	133	102	39	14	11
Solyc02g076690.2.1	4.500927	6.112398	1.60E-08	4.22E-07	14684	3950	2942	351	165	120
Solyc07g005680.2.1	4.511724	0.165004	4.51E-05	0.000456	25	181	218	0	6	4
Solyc01g107730.2.1	4.514322	-0.10843	1.02E-05	0.000125	37	110	183	1	4	3
Solyc10g086580.1.1	4.518061	8.715515	3.14E-05	0.000334	100761	14782	9588	2982	555	264
Solyc05g012510.2.1	4.521104	5.230971	2.48E-14	1.97E-12	1040	7969	6477	111	150	91
Solyc12g013710.1.1	4.547998	2.800039	1.43E-06	2.26E-05	1394	450	356	47	9	5
Solyc06g048620.2.1	4.549141	0.769264	6.05E-06	7.93E-05	72	333	263	11	4	0
Solyc02g030170.2.1	4.553415	6.653019	2.45E-20	4.20E-18	9270	17425	11889	209	470	262
Solyc12g011450.1.1	4.564176	6.484641	0.000115	0.00102	22764	2133	1108	606	155	39
Solyc06g049030.2.1	4.565543	2.073684	2.94E-06	4.21E-05	182	1074	514	0	38	6
Solyc04g005070.2.1	4.573834	2.865543	2.01E-07	4.00E-06	84	2274	926	9	50	13
Solyc10g084950.1.1	4.580315	2.033282	1.89E-12	1.11E-10	382	625	513	13	11	11
Solyc10g018780.1.1	4.585288	-0.22553	2.47E-05	0.000269	31	170	127	0	4	3
Solyc09g065620.2.1	4.599663	6.470767	1.95E-07	3.89E-06	537	15162	20101	179	487	174
Solyc10g047110.1.1	4.602582	0.414076	0.001	0.006336	328	17	26	6	6	0
Solyc12g062500.1.1	4.602996	0.244054	0.000254	0.001997	257	38	49	7	3	0
Solyc09g011080.2.1	4.623361	0.388778	7.92E-06	9.97E-05	250	136	37	4	5	2
Solyc02g070990.1.1	4.627307	1.776102	0.000316	0.002403	893	61	16	14	7	7
Solyc02g064690.2.1	4.631031	2.489809	4.06E-09	1.22E-07	464	1177	587	6	48	3
Solyc02g068170.1.1	4.635161	-0.04544	0.000144	0.001233	202	57	31	0	5	3
Solyc02g064720.2.1	4.638804	0.404525	3.26E-05	0.000345	36	461	95	0	10	2
Solyc02g037550.2.1	4.642648	0.879478	0.000191	0.001567	8	531	262	0	8	7
Solyc06g068160.2.1	4.650846	0.033875	0.00024	0.001905	199	126	9	3	6	0
Solyc05g056070.2.1	4.662335	6.367437	7.08E-07	1.22E-05	16405	4377	5242	519	141	33
Solyc03g117690.2.1	4.665365	2.396914	4.13E-12	2.27E-10	297	1211	652	16	26	5
Solyc02g090030.2.1	4.671316	6.763163	6.05E-11	2.66E-09	17641	11554	8106	577	209	112
Solyc12g006470.1.1	4.679528	6.170547	4.85E-10	1.76E-08	5728	18174	6082	431	118	44
Solyc05g015800.2.1	4.682449	3.563679	6.38E-06	8.30E-05	51	1813	2830	13	92	11
Solyc02g070380.1.1	4.70073	-1.01797	0.001439	0.008642	93	30	20	0	0	3
Solyc11g069980.1.1	4.704918	-0.82736	6.14E-05	0.000596	55	53	75	3	1	0
Solyc05g050370.1.1	4.705002	-1.58972	0.000514	0.003606	16	66	34	2	0	0
Solyc03g093700.1.1	4.706091	-1.51479	0.000867	0.005632	17	55	46	0	0	2
Solyc06g005070.1.1	4.71997	0.872107	1.70E-06	2.63E-05	231	340	108	9	0	4
Solyc10g006530.2.1	4.720528	0.673474	0.000338	0.002548	341	46	82	11	1	0
Solyc02g077140.2.1	4.73613	-0.46516	2.10E-05	0.000232	67	110	79	0	6	0
Solyc03g046580.1.1	4.739099	-0.36201	0.000245	0.001936	157	17	48	3	3	0
Solyc04g050930.2.1	4.746286	2.010424	3.01E-08	7.36E-07	199	792	617	22	9	1
Solyc07g063910.2.1	4.749588	2.537837	0.000197	0.001606	611	1705	137	42	0	1
Solyc01g021730.2.1	4.753984	-2.3308	0.001467	0.008781	9	45	11	0	1	0

Solyc02g080200.2.1	4.76339	0.687872	3.10E-06	4.40E-05	260	135	130	0	6	6
Solyc11g008630.1.1	4.76434	3.081568	2.41E-11	1.17E-09	1138	1010	848	18	54	4
Solyc12g094720.1.1	4.775088	1.560752	3.38E-10	1.28E-08	355	385	313	3	9	10
Solyc03g114310.2.1	4.776484	0.431342	1.52E-06	2.39E-05	64	255	204	0	9	2
Solyc07g045080.2.1	4.785121	0.993577	0.000344	0.00258	449	47	85	13	0	1
Solyc02g067360.2.1	4.794994	1.722171	5.37E-09	1.57E-07	305	581	369	6	2	14
Solyc09g083440.2.1	4.803825	10.55987	3.74E-08	8.89E-07	21867	156851	393792	5743	2276	2951
Solyc12g010550.1.1	4.810781	2.24507	1.56E-06	2.43E-05	54	1540	565	2	14	18
Solyc01g097770.2.1	4.811937	5.107389	6.80E-15	5.76E-13	1609	9373	3913	137	110	27
Solyc03g043740.2.1	4.812077	0.792992	7.31E-07	1.25E-05	318	136	101	7	5	1
Solyc10g055730.1.1	4.817547	1.189657	1.19E-10	4.94E-09	201	344	300	4	8	5
Solyc12g042180.1.1	4.823783	-0.4254	7.30E-05	0.000693	149	56	21	1	2	2
Solyc06g054130.1.1	4.845318	-1.49385	0.000462	0.003307	48	33	26	2	0	0
Solyc03g119540.2.1	4.848743	4.663975	1.44E-24	4.01E-22	2045	5192	2894	42	87	61
Solyc04g014530.1.1	4.864668	2.987002	3.07E-13	2.04E-11	331	1511	1345	21	14	20
Solyc01g087780.2.1	4.876759	4.850983	3.16E-09	9.66E-08	5662	2102	1599	144	35	25
Solyc07g042630.2.1	4.882776	3.176566	7.00E-12	3.74E-10	345	3050	734	25	37	8
Solyc12g008800.1.1	4.892231	2.667166	5.42E-13	3.46E-11	736	828	738	26	15	5
Solyc03g083510.2.1	4.898792	-1.12576	4.84E-05	0.000484	25	73	63	0	3	0
Solyc10g008720.2.1	4.902065	1.887767	8.29E-09	2.35E-07	209	627	601	19	3	3
Solyc02g077980.1.1	4.909808	0.580979	8.39E-07	1.42E-05	69	412	148	0	5	5
Solyc05g009270.2.1	4.921149	-1.5485	8.86E-05	0.000814	26	47	39	0	2	0
Solyc03g122360.2.1	4.940298	2.06114	1.28E-05	0.000151	42	1030	714	0	11	16
Solyc02g064940.1.1	4.940814	4.970985	9.17E-18	1.11E-15	2735	5918	3677	22	148	65
Solyc02g071010.2.1	4.94487	5.482231	0.000841	0.005484	12089	181	354	266	19	13
Solyc02g066920.2.1	4.950008	0.590728	0.000354	0.002635	367	46	22	6	0	3
Solyc02g020940.2.1	4.957067	5.172257	6.87E-21	1.30E-18	4635	4663	3752	96	137	34
Solyc01g110180.2.1	4.972991	-0.06982	4.21E-05	0.00043	23	193	159	0	7	0
Solyc02g065530.2.1	4.989519	-0.249	2.12E-05	0.000235	127	71	73	0	6	0
Solyc03g005780.1.1	4.996771	5.399715	0.000439	0.003164	11498	272	185	224	38	15
Solyc05g008300.1.1	4.999934	-0.38993	4.39E-05	0.000445	85	86	86	0	0	4
Solyc02g081340.2.1	5.002807	1.726745	3.82E-05	0.000396	4	1298	292	8	12	3
Solyc10g007690.2.1	5.012367	7.137348	2.41E-08	6.09E-07	31820	6667	5087	558	224	141
Solyc11g045530.1.1	5.013548	-1.25217	0.000537	0.003745	16	49	76	0	0	2
Solyc06g083900.2.1	5.017023	0.973334	9.87E-07	1.63E-05	31	251	462	5	3	4
Solyc11g010340.1.1	5.018216	-2.02611	0.00096	0.006123	17	24	32	0	0	1
Solyc08g083360.2.1	5.023526	3.969666	4.87E-08	1.13E-06	3310	665	889	69	30	6
Solyc08g066630.2.1	5.049473	-2.12312	0.000337	0.002538	22	27	19	0	1	0
Solyc08g006830.2.1	5.064251	1.334052	2.38E-11	1.15E-09	281	392	258	3	8	5
Solyc02g063150.2.1	5.105696	7.824537	0.00028	0.002169	58368	4666	3369	1262	67	30
Solyc05g009500.2.1	5.116688	0.864655	8.24E-06	0.000103	146	337	216	0	0	9
Solyc02g085020.2.1	5.131268	1.578014	0.000466	0.003329	0	1210	243	9	9	1
Solyc01g102720.2.1	5.140013	1.045825	1.41E-07	2.90E-06	237	258	241	10	2	0
Solyc05g026490.2.1	5.151976	0.812448	0.000109	0.000974	431	44	34	2	10	0
Solyc05g010060.2.1	5.153127	-1.91214	0.000495	0.003489	16	39	30	0	0	1
Solyc08g075880.2.1	5.158398	1.585603	2.84E-12	1.61E-10	232	551	386	5	12	2
Solyc11g072310.1.1	5.1794	0.165517	0.001242	0.007619	0	236	219	0	6	1
Solyc07g043460.2.1	5.179936	4.323027	3.21E-05	0.000341	391	6526	2298	107	1	4
Solyc01g090760.2.1	5.182954	-0.8322	3.48E-05	0.000365	93	58	27	1	2	0
Solyc12g005020.1.1	5.197839	-0.33514	0.000856	0.005567	184	28	15	4	0	0
Solyc10g083860.1.1	5.198277	-0.69249	9.63E-06	0.000118	72	100	44	0	1	2
Solyc09g065800.2.1	5.212244	-1.3568	0.00137	0.00829	77	35	3	0	2	0
Solyc10g055790.1.1	5.212674	-0.86048	1.88E-05	0.000212	62	43	71	0	3	0
Solyc07g006550.2.1	5.21421	-1.16029	0.000121	0.001064	44	101	18	2	0	0
Solyc09g075240.1.1	5.21827	-1.86612	0.00038	0.002802	22	33	30	0	0	1
Solyc04g009880.1.1	5.232361	-1.98006	0.000212	0.001712	26	25	24	0	1	0
Solyc12g096490.1.1	5.261434	-0.06695	4.39E-06	5.97E-05	168	89	51	0	3	2
Solyc03g116730.2.1	5.323196	3.377047	0.000825	0.005396	2879	18	27	42	5	6
Solyc08g061560.2.1	5.335251	2.421645	4.57E-12	2.50E-10	297	1020	831	15	14	1
Solyc06g009190.2.1	5.372842	3.79766	7.70E-08	1.69E-06	186	2803	2817	23	61	1
Solyc08g075150.2.1	5.388533	0.398148	1.45E-06	2.29E-05	34	438	117	0	6	1
Solyc01g014380.2.1	5.395105	-0.0179	1.20E-05	0.000142	36	247	121	0	0	4
Solyc12g098900.1.1	5.403511	1.883053	4.14E-05	0.000424	588	816	20	16	2	0
Solyc09g007480.2.1	5.414448	1.639315	5.64E-12	3.05E-10	221	651	380	2	13	2
Solyc12g009650.1.1	5.43947	5.971179	1.49E-05	0.000172	16900	543	600	192	65	48
Solyc02g092050.2.1	5.445245	-0.35685	0.001612	0.009488	0	150	157	0	4	0
Solyc07g006620.2.1	5.453056	1.687646	1.45E-08	3.87E-07	492	319	296	0	16	2
Solyc12g009930.1.1	5.463362	6.731125	3.18E-08	7.74E-07	396	26970	19440	91	349	127
Solyc02g088190.2.1	5.470391	1.114679	3.51E-10	1.32E-08	236	291	260	7	2	1
Solyc02g070230.1.1	5.556175	0.110236	1.15E-06	1.86E-05	78	200	133	0	0	4
Solyc10g085610.1.1	5.559962	1.485265	2.81E-10	1.09E-08	213	563	338	0	12	2
Solyc08g013670.2.1	5.566246	6.744107	1.78E-06	2.74E-05	26971	2866	2236	364	47	56
Solyc09g061730.1.1	5.569092	0.369324	1.71E-05	0.000195	8	419	153	0	5	1
Solyc08g067320.1.1	5.569914	1.020687	4.74E-05	0.000476	490	24	72	5	0	3
Solyc05g007090.2.1	5.586518	0.378993	2.56E-08	6.39E-07	114	207	162	2	0	3
Solyc06g074760.1.1	5.623219	-0.93107	2.31E-06	3.41E-05	51	72	57	0	2	0
Solyc11g012880.1.1	5.635031	-1.65646	0.000104	0.000938	45	23	25	0	1	0
Solyc08g082650.2.1	5.654237	-1.50722	0.000344	0.00258	59	27	17	0	0	1
Solyc11g066270.1.1	5.659126	1.243348	5.26E-07	9.40E-06	25	610	388	0	6	4
Solyc10g084340.1.1	5.672331	-0.0766	9.24E-08	1.99E-06	89	152	107	0	4	0
Solyc09g018890.2.1	5.693193	3.35983	7.20E-07	1.24E-05	2585	327	171	28	14	2
Solyc08g074480.1.1	5.704967	6.141108	1.76E-13	1.22E-11	9112	8215	8200	14	154	127
Solyc04g024830.1.1	5.705691	-1.56799	2.07E-05	0.00023	25	57	33	0	1	0
Solyc04g076190.1.1	5.711203	-1.63355	0.001665	0.009748	70	14	3	0	1	0

Solyc02g069390.1.1	5.74342	-0.6245	9.46E-06	0.000116	31	107	101	0	0	2
Solyc02g071030.1.1	5.772695	4.135817	1.03E-06	1.70E-05	3936	921	688	0	73	11
Solyc12g056920.1.1	5.784049	-1.39139	0.000185	0.001525	60	45	14	0	0	1
Solyc05g005960.2.1	5.794958	3.035224	5.36E-19	7.67E-17	408	1717	1258	11	16	6
Solyc11g066010.1.1	5.813323	-1.47896	1.43E-05	0.000166	25	49	46	0	1	0
Solyc02g071000.1.1	5.909601	2.799439	0.001305	0.007946	1952	12	5	20	5	0
Solyc01g073990.2.1	5.913468	-0.11584	3.34E-08	8.05E-07	64	138	138	0	2	1
Solyc12g014140.1.1	5.921123	3.71747	1.42E-22	3.14E-20	1251	1523	2083	18	11	16
Solyc10g008710.2.1	5.938306	0.378441	2.63E-08	6.55E-07	143	138	173	0	1	3
Solyc03g031870.1.1	6.105439	1.423358	0.000457	0.003272	727	42	2	7	1	0
Solyc07g032490.2.1	6.204579	-1.14148	3.07E-06	4.36E-05	39	44	66	0	1	0
Solyc01g073640.2.1	6.208499	5.842606	4.13E-10	1.52E-08	643	14706	10053	35	160	9
Solyc12g017270.1.1	6.257837	-2.85696	0.001495	0.008918	7	21	10	0	0	0
Solyc06g064550.2.1	6.272815	1.001332	5.81E-10	2.08E-08	245	217	247	0	6	0
Solyc06g054370.1.1	6.301578	-2.83153	0.001441	0.008658	7	18	13	0	0	0
Solyc07g043420.2.1	6.312472	8.132366	4.88E-13	3.13E-11	28828	82316	10863	539	129	84
Solyc08g007330.2.1	6.345558	1.245141	7.50E-10	2.62E-08	65	539	391	0	3	3
Solyc02g084850.2.1	6.355112	5.950264	4.42E-05	0.000448	112	32788	792	42	159	1
Solyc09g082660.2.1	6.384328	1.677827	0.000352	0.002627	0	1339	253	0	10	0
Solyc04g080250.2.1	6.395447	-2.78595	0.001671	0.009777	16	14	7	0	0	0
Solyc06g009710.2.1	6.454875	-2.74248	0.001205	0.007429	12	10	16	0	0	0
Solyc07g045570.1.1	6.483293	-2.69582	0.001203	0.00742	5	21	18	0	0	0
Solyc06g068170.1.1	6.506238	-2.66297	0.001336	0.008109	7	35	7	0	0	0
Solyc04g072700.2.1	6.522194	1.128402	2.68E-08	6.66E-07	223	694	33	3	2	0
Solyc03g114940.2.1	6.544842	1.667596	7.22E-13	4.48E-11	179	528	544	0	3	4
Solyc04g078200.2.1	6.589086	4.2354	1.14E-07	2.40E-06	78	4933	3402	0	34	13
Solyc08g008050.2.1	6.595442	-2.63988	0.000763	0.005055	16	13	13	0	0	0
Solyc09g042670.2.1	6.633641	-2.61452	0.001108	0.006924	13	8	21	0	0	0
Solyc01g014560.2.1	6.639872	-2.58536	0.001006	0.006368	15	29	5	0	0	0
Solyc04g014970.1.1	6.651395	-2.58758	0.000489	0.003467	13	18	15	0	0	0
Solyc01g013900.2.1	6.684231	-2.55491	0.000418	0.003041	10	22	17	0	0	0
Solyc03g062710.2.1	6.706306	0.184703	7.32E-08	1.62E-06	188	71	107	2	0	0
Solyc03g078810.2.1	6.74068	-2.5254	0.000476	0.003389	18	19	11	0	0	0
Solyc11g066580.1.1	6.749255	0.533966	0.000108	0.000967	0	514	162	0	3	0
Solyc04g072320.1.1	6.776359	-2.51849	0.001462	0.008762	27	13	5	0	0	0
Solyc08g008480.2.1	6.790311	-2.48842	0.000353	0.002632	16	16	17	0	0	0
Solyc06g065450.2.1	6.790964	-2.47477	0.000512	0.003597	7	19	26	0	0	0
Solyc03g045070.1.1	6.840061	-0.49757	2.42E-05	0.000263	156	36	19	1	0	0
Solyc05g007010.1.1	6.871244	0.79289	1.11E-11	5.74E-10	213	214	195	0	2	1
Solyc01g108060.1.1	6.879985	-2.41875	0.000351	0.002623	21	22	10	0	0	0
Solyc06g006090.1.1	6.88556	-0.51768	1.44E-07	2.95E-06	70	75	95	0	1	0
Solyc01g060350.1.1	6.909108	-2.3947	0.000231	0.001842	18	20	16	0	0	0
Solyc04g005210.1.1	6.929268	-2.3528	0.000307	0.002347	7	37	19	0	0	0
Solyc12g056200.1.1	6.982901	-2.3548	0.001221	0.007505	18	4	29	0	0	0
Solyc05g008240.2.1	7.014897	-2.30455	0.000731	0.004873	5	15	39	0	0	0
Solyc02g083520.2.1	7.041671	-2.27504	0.00013	0.001131	14	35	16	0	0	0
Solyc08g068190.2.1	7.044675	-2.28678	0.000145	0.001242	20	24	16	0	0	0
Solyc07g007420.2.1	7.063068	-2.25382	0.000453	0.003256	4	29	33	0	0	0
Solyc01g094360.2.1	7.066974	-2.2545	0.000297	0.002279	6	27	32	0	0	0
Solyc05g014710.2.1	7.07927	-0.3358	1.38E-07	2.85E-06	20	152	139	0	1	0
Solyc01g073780.2.1	7.081179	-2.29233	0.00141	0.008494	38	9	5	0	0	0
Solyc04g077380.2.1	7.084543	-2.25471	0.000456	0.003271	26	32	5	0	0	0
Solyc03g098490.1.1	7.094253	-2.2272	0.000191	0.001564	7	36	26	0	0	0
Solyc02g022890.1.1	7.122644	-2.21484	8.68E-05	0.000801	15	32	20	0	0	0
Solyc10g074680.1.1	7.130052	-2.20754	8.71E-05	0.000803	15	34	19	0	0	0
Solyc08g077070.2.1	7.188014	-2.15303	0.000101	0.000909	10	41	23	0	0	0
Solyc06g068130.2.1	7.282249	4.104108	8.39E-05	0.000779	0	5540	2556	0	28	2
Solyc07g045580.1.1	7.330384	-2.04611	8.77E-05	0.000807	10	31	36	0	0	0
Solyc12g036370.1.1	7.33351	-2.04334	4.95E-05	0.000495	14	36	28	0	0	0
Solyc01g008390.1.1	7.333593	-2.09009	0.001388	0.008386	48	10	3	0	0	0
Solyc05g005170.2.1	7.339495	-2.09006	0.001683	0.009834	47	3	9	0	0	0
Solyc03g113260.1.1	7.344111	-2.04924	0.000353	0.002634	34	36	4	0	0	0
Solyc07g043500.1.1	7.359935	4.965299	3.23E-08	7.84E-07	31	8999	5304	9	4	23
Solyc12g044320.1.1	7.36061	-2.0141	6.47E-05	0.000623	10	44	29	0	0	0
Solyc12g040870.1.1	7.371649	-1.96678	0.001009	0.006383	3	94	6	0	0	0
Solyc03g078800.2.1	7.381763	-1.99941	4.58E-05	0.000463	18	49	17	0	0	0
Solyc02g085590.2.1	7.428552	-1.92143	0.001304	0.007943	6	99	2	0	0	0
Solyc11g071290.1.1	7.438188	2.928158	5.85E-24	1.50E-21	789	1037	1045	4	1	4
Solyc02g072550.1.1	7.445111	-1.9251	0.000404	0.002949	2	69	27	0	0	0
Solyc06g008390.2.1	7.446479	-1.9571	3.63E-05	0.000378	26	39	17	0	0	0
Solyc07g049430.2.1	7.453747	-1.97918	0.000343	0.002577	46	17	7	0	0	0
Solyc06g066710.1.1	7.464416	-1.9138	0.00012	0.001058	6	69	23	0	0	0
Solyc09g065350.1.1	7.47942	-1.95763	0.000268	0.00209	46	16	9	0	0	0
Solyc11g020860.1.1	7.509589	-1.90569	2.29E-05	0.000251	21	32	31	0	0	0
NCRNA_5_310	7.539814	-1.88593	3.58E-05	0.000374	33	35	16	0	0	0
Solyc01g090800.2.1	7.555526	-1.851	6.45E-05	0.000622	7	50	39	0	0	0
Solyc12g038350.1.1	7.574725	-1.84742	1.64E-05	0.000188	23	43	25	0	0	0
Solyc01g091180.2.1	7.646232	-1.77339	2.37E-05	0.000259	12	64	29	0	0	0
Solyc10g021280.1.1	7.687097	-1.74957	1.24E-05	0.000147	17	46	37	0	0	0
Solyc06g068550.2.1	7.687739	-1.74587	1.80E-05	0.000204	13	49	40	0	0	0
Solyc05g009950.2.1	7.700017	1.032002	1.00E-10	4.25E-09	47	492	334	0	2	0
Solyc10g054820.1.1	7.707515	0.26603	7.98E-11	3.43E-09	89	205	167	0	1	0
Solyc01g095940.2.1	7.710902	-1.72313	2.13E-05	0.000236	11	55	40	0	0	0
Solyc09g090090.1.1	7.736936	-1.70179	1.35E-05	0.000158	14	58	36	0	0	0

Solyc01g011230.2.1	7.810098	-1.65922	1.07E-05	0.000129	39	35	25	0	0	0
Solyc07g009450.1.1	7.87011	-1.58554	0.001067	0.006689	0	49	68	0	0	0
Solyc01g091870.2.1	7.883314	-1.60657	5.01E-05	0.0005	57	30	11	0	0	0
Solyc12g035400.1.1	7.953944	-1.51455	4.53E-06	6.16E-05	21	80	28	0	0	0
Solyc04g011870.1.1	7.961936	-1.53586	0.000973	0.006191	62	46	0	0	0	0
Solyc09g015030.1.1	8.015045	-1.47822	3.73E-06	5.18E-05	43	50	25	0	0	0
Solyc04g064610.2.1	8.072331	0.677786	1.85E-05	0.000209	430	12	14	1	0	0
Solyc04g050730.2.1	8.15669	0.752381	4.04E-08	9.54E-07	387	60	66	1	0	0
Solyc12g055970.1.1	8.165748	-1.35717	6.81E-06	8.76E-05	62	31	26	0	0	0
Solyc03g123580.2.1	8.202093	-1.29479	7.34E-06	9.36E-05	9	92	55	0	0	0
Solyc07g053900.2.1	8.266443	-1.26524	2.77E-06	3.98E-05	62	37	31	0	0	0
Solyc03g006880.2.1	8.271728	-1.2258	0.000394	0.002887	0	110	61	0	0	0
Solyc01g100910.2.1	8.282956	-1.27307	0.001406	0.008482	101	12	0	0	0	0
Solyc10g085280.1.1	8.317019	-1.19534	3.40E-05	0.000359	3	81	80	0	0	0
Solyc01g007800.2.1	8.34426	-1.19675	2.86E-06	4.11E-05	56	24	54	0	0	0
Solyc12g098590.1.1	8.349987	1.605981	5.03E-06	6.75E-05	0	1256	260	0	2	0
Solyc01g007810.1.1	8.381475	-1.17341	2.05E-05	0.000229	91	23	15	0	0	0
Solyc12g088700.1.1	8.419512	-1.11388	4.15E-07	7.61E-06	26	68	70	0	0	0
Solyc02g032410.2.1	8.442988	-1.08882	2.46E-07	4.80E-06	29	97	50	0	0	0
Solyc01g079100.2.1	8.445287	-1.09943	2.11E-06	3.16E-05	32	28	91	0	0	0
Solyc08g067500.1.1	8.542333	-0.97689	0.000281	0.002177	0	187	39	0	0	0
Solyc08g078420.1.1	8.628384	-0.92114	8.27E-06	0.000103	5	80	111	0	0	0
Solyc05g005870.2.1	8.648469	-0.94333	0.001522	0.009065	136	4	0	0	0	0
Solyc06g082410.1.1	8.724208	-0.83266	1.36E-07	2.80E-06	22	126	72	0	0	0
Solyc01g108330.2.1	8.813801	-0.75843	3.94E-08	9.35E-07	36	83	94	0	0	0
Solyc02g079600.2.1	8.861966	-0.71981	8.45E-07	1.42E-05	95	106	13	0	0	0
Solyc03g026050.2.1	8.868397	-0.70748	1.08E-07	2.29E-06	59	122	48	0	0	0
Solyc02g077970.2.1	8.978379	-0.57748	0.000329	0.002493	0	319	11	0	0	0
Solyc11g007250.1.1	9.007438	-0.58314	3.40E-08	8.19E-07	67	93	77	0	0	0
Solyc07g014620.1.1	9.185455	-0.40947	1.01E-07	2.16E-06	14	151	134	0	0	0
Solyc08g078410.1.1	9.252813	-0.35346	5.05E-06	6.77E-05	5	48	223	0	0	0
Solyc12g009920.1.1	9.255529	-0.3395	6.24E-07	1.09E-05	6	214	116	0	0	0
Solyc03g025190.2.1	9.344746	-0.25078	7.42E-07	1.27E-05	7	321	63	0	0	0
Solyc08g069130.1.1	9.452605	-0.18872	0.001444	0.00867	241	0	0	0	0	0
Solyc09g091960.2.1	9.746763	0.119954	9.28E-09	2.59E-07	15	298	157	0	0	0
Solyc06g072130.2.1	9.777692	0.162277	0.000149	0.001265	0	577	6	0	0	0
Solyc12g044200.1.1	9.923299	0.281977	5.37E-10	1.93E-08	47	181	240	0	0	0
Solyc01g090340.2.1	10.07776	0.438575	9.63E-08	2.07E-06	5	494	139	0	0	0
Solyc01g107460.2.1	10.74814	1.08268	1.68E-10	6.73E-09	32	800	183	0	0	0
Solyc12g088170.1.1	11.12209	1.448076	5.62E-07	9.94E-06	0	1069	269	0	0	0
Solyc02g070970.1.1	12.15088	2.451585	1.04E-06	1.71E-05	1553	5	11	0	0	0

DE genes identified with pairwise correlation of *O. neolycopersici* - and mock-infected tomato leaf tissue.

GeneID	logFC	logCPM	PValue	FDR	ONm1	ONm2	ONm3	ON1	ON2	ON3		
Solyc10g085800.1.1	-7.73092	-1.754	6.17E-06	0.007916	0	0	0	15	37	36	ON up	1
Solyc12g010170.1.1	1.429663	3.799572	8.25E-06	0.009689	976	1244	748	241	642	424	ON down	22
Solyc12g005850.1.1	1.612131	6.924314	8.14E-09	2.74E-05	10244	9856	6463	2471	4154	3325		
Solyc06g053550.2.1	1.615667	7.433248	3.22E-06	0.004565	13413	16860	8085	2612	8710	3626		
Solyc01g095740.2.1	1.65864	5.507719	1.28E-10	6.91E-07	3567	3903	2583	918	1447	1257		
Solyc05g054660.1.1	1.683046	6.472682	7.77E-07	0.001308	8375	7250	4037	1900	3282	1724		
Solyc11g018740.1.1	1.773582	6.359719	1.09E-09	4.88E-06	6139	8657	3981	1160	2778	2528		
Solyc04g079430.2.1	1.897093	2.392105	6.00E-06	0.007916	290	607	325	73	158	149		
Solyc04g015970.2.1	1.979665	4.357257	3.46E-08	7.78E-05	1179	2731	1006	301	716	396		
Solyc04g074680.1.1	2.22707	3.036699	2.64E-07	0.000508	477	1175	385	92	172	232		
Solyc12g019980.1.1	2.233276	3.512992	1.73E-08	4.67E-05	968	1288	522	116	216	364		
Solyc02g037540.1.1	2.378996	3.01061	5.49E-09	2.11E-05	605	880	497	73	135	244		
Solyc01g095570.2.1	2.828918	7.259117	3.47E-08	7.78E-05	16831	20665	2742	1575	2814	1890		
Solyc03g112340.1.1	3.073511	2.690231	1.92E-06	0.002875	524	1103	130	86	63	55		
Solyc02g079950.2.1	3.309369	3.008074	2.23E-11	2.00E-07	893	1004	260	65	98	78		
Solyc09g005930.2.1	3.388209	1.59012	1.64E-08	4.67E-05	233	288	267	5	52	40		
Solyc06g011290.1.1	3.946297	1.639174	8.27E-06	0.009689	147	688	74	2	53	17		
Solyc12g005300.1.1	3.989107	2.102704	6.33E-07	0.001136	159	885	202	4	19	71		
NCRNA_9_267	4.683345	3.399859	1.69E-07	0.000351	1237	1725	104	46	69	7		
Solyc09g066490.2.1	4.868461	4.220067	2.41E-12	3.24E-08	1913	3387	204	56	65	79		
Solyc03g123740.2.1	5.227832	1.841789	7.53E-16	2.03E-11	320	562	157	9	15	6		
Solyc07g065640.2.1	8.723894	1.756516	2.96E-11	2.00E-07	341	668	18	2	0	0		
Solyc02g070770.2.1	9.632533	-0.0238	1.02E-06	0.001615	97	194	1	0	0	0		

DE genes identified with pairwise correlation of *P. infestans* - and mock-infected tomato leaf tissue.

Gene ID	logFC	logCPM	PValue	FDR	Pim1	Pim2	Pim3	Pi1	Pi2	Pi3		
Solyc07g062370.1.1	-9.89317	0.441344	7.88E-09	1.53E-06	0	0	0	212	207	4	Pi Up	661
Solyc02g085800.1.1	-9.86693	0.409334	1.92E-10	6.72E-08	0	0	0	257	115	16	Pi Down	49
Solyc02g036240.1.1	-9.40284	-0.03971	8.02E-10	2.08E-07	0	0	0	176	27	57		
NCRNA_6_224	-9.09401	-0.33203	8.14E-05	0.003759	0	0	0	198	0	9		
Solyc07g052130.1.1	-8.93315	-0.44697	2.00E-08	3.54E-06	0	0	0	62	152	15		
Solyc07g052450.1.1	-8.89471	-0.48559	5.98E-09	1.19E-06	0	0	0	76	130	12		
Solyc09g066400.1.1	-8.80407	1.506435	5.69E-09	1.15E-06	1	0	0	644	176	12		
Solyc08g080660.1.1	-8.57545	3.598801	6.02E-09	1.19E-06	4	0	1	197	4455	60		
Solyc11g006240.1.1	-8.43962	-0.89771	6.51E-07	6.85E-05	0	0	0	65	87	5		
Solyc12g056180.1.1	-8.36988	-1.00463	8.32E-06	0.00059	0	0	0	79	4	40		
Solyc01g073860.2.1	-8.23484	-1.08203	3.40E-07	3.89E-05	0	0	0	54	73	8		
Solyc05g055270.1.1	-8.05869	-1.25418	7.74E-08	1.08E-05	0	0	0	34	47	31		
Solyc07g052370.2.1	-7.95619	2.710651	2.82E-09	6.09E-07	4	0	0	421	1933	34		
Solyc03g096180.2.1	-7.68816	-1.55906	1.53E-06	0.000142	0	0	0	11	56	25		
Solyc04g083140.1.1	-7.66002	0.427715	2.56E-07	3.09E-05	1	0	0	69	389	9		
Solyc01g094160.1.1	-7.64665	-1.5674	1.18E-05	0.000787	0	0	0	11	76	10		
Solyc03g031450.2.1	-7.54358	-1.66769	2.07E-05	0.00126	0	0	0	28	54	4		
Solyc02g090470.2.1	-7.53213	-1.67652	5.15E-06	0.00039	0	0	0	19	58	9		
Solyc01g097060.2.1	-7.44254	-1.7779	3.68E-06	0.00029	0	0	0	33	31	10		
Solyc05g014300.1.1	-7.36235	-1.83892	3.75E-05	0.002037	0	0	0	36	31	4		
Solyc02g086850.1.1	-7.33505	-1.86682	1.82E-06	0.000164	0	0	0	22	32	15		
Solyc03g098560.1.1	-7.26424	-1.90303	2.02E-05	0.001236	0	0	0	21	43	6		
Solyc09g014720.1.1	-7.23025	0.572335	1.52E-10	5.38E-08	0	0	2	207	238	20		
Solyc07g061710.1.1	-7.17636	-1.99115	6.30E-06	0.000461	0	0	0	13	34	16		
Solyc02g080830.1.1	-7.13765	-2.00164	2.67E-05	0.001574	0	0	0	12	44	9		
Solyc07g049670.2.1	-7.12866	-2.03895	1.13E-05	0.000758	0	0	0	25	24	10		
Solyc03g005500.1.1	-7.01867	1.746596	4.95E-23	7.84E-19	0	5	1	401	451	185		
Solyc04g011480.2.1	-6.98186	2.069511	1.02E-17	1.64E-14	0	6	2	485	791	105		
Solyc11g008950.1.1	-6.92061	-2.16581	0.000129	0.005581	0	0	0	7	42	8		
Solyc08g080310.1.1	-6.85872	0.710279	8.29E-13	4.96E-10	0	0	3	156	327	46		
Solyc05g055370.1.1	-6.83121	-2.2536	7.47E-05	0.003492	0	0	0	15	30	6		
NCRNA_6_5	-6.82425	-2.25815	0.000135	0.005812	0	0	0	5	33	13		
Solyc01g008610.2.1	-6.81863	-0.56347	7.75E-08	1.08E-05	0	1	0	100	82	12		
Solyc12g015700.1.1	-6.7492	-2.32719	5.43E-05	0.002693	0	0	0	10	26	11		
Solyc08g013920.2.1	-6.72307	-2.33942	0.000104	0.004677	0	0	0	14	27	6		
Solyc06g075100.2.1	-6.62565	1.35053	1.83E-16	2.38E-13	0	6	0	344	318	115		
Solyc09g014460.1.1	-6.58449	-2.45951	0.000115	0.0051	0	0	0	15	19	7		
Solyc03g044180.1.1	-6.55944	3.096492	1.70E-20	5.78E-17	8	5	5	1028	1469	280		
Solyc03g118000.1.1	-6.4786	3.087065	2.78E-19	7.58E-16	8	8	3	1133	1323	263		
Solyc01g087960.1.1	-6.43716	-0.18287	3.84E-08	6.08E-06	0	2	0	132	113	14		
Solyc10g054350.1.1	-6.30946	-0.32562	2.35E-06	0.0002	0	1	1	173	18	19		
Solyc06g061210.2.1	-6.29896	1.364497	3.62E-09	7.70E-07	0	8	0	260	577	28		
Solyc07g056100.1.1	-5.99646	-0.57595	6.23E-09	1.22E-06	0	2	0	73	93	27		
Solyc02g067600.1.1	-5.95822	-0.32801	7.30E-07	7.53E-05	2	0	0	61	162	22		
Solyc04g012100.1.1	-5.78685	-1.46717	3.07E-06	0.00025	0	0	1	38	46	12		
Solyc01g087280.1.1	-5.60097	0.223055	7.36E-08	1.05E-05	1	4	0	219	56	43		
Solyc11g065910.1.1	-5.5454	-1.66109	1.54E-05	0.000973	0	1	0	20	54	11		
Solyc11g069560.1.1	-5.47644	2.13741	3.51E-07	3.97E-05	0	27	0	581	748	82		
Solyc02g081370.2.1	-5.45028	0.574576	3.72E-10	1.11E-07	2	0	5	150	82	169		
Solyc02g077570.1.1	-5.42169	-0.07764	9.09E-07	8.97E-05	2	2	0	167	66	27		
Solyc08g023510.1.1	-5.3722	4.48723	2.48E-10	7.94E-08	23	96	4	2003	5050	570		
Solyc11g072920.1.1	-5.30264	-0.16893	1.30E-05	0.000852	0	5	0	118	142	7		
Solyc02g089920.1.1	-5.28205	-0.77709	4.81E-07	5.28E-05	0	1	2	86	34	32		
Solyc02g036220.1.1	-5.27245	0.203607	2.21E-06	0.00019	0	7	0	226	65	26		
Solyc02g068460.1.1	-5.26455	-0.21988	9.56E-06	0.000659	0	5	0	169	34	25		
Solyc04g011750.2.1	-5.26338	-0.3406	2.79E-05	0.001619	3	0	0	79	141	16		
Solyc08g029000.2.1	-5.25931	7.739473	2.19E-08	3.78E-06	262	910	50	15455	57017	3172		
Solyc09g066350.1.1	-5.25142	-1.23327	5.28E-05	0.002643	0	0	2	45	69	5		
Solyc04g079260.2.1	-5.2332	4.46999	2.39E-11	1.12E-08	26	102	4	1925	4482	922		
Solyc07g056610.1.1	-5.10268	-1.36305	4.14E-05	0.002179	0	2	0	9	73	26		
Solyc07g040710.2.1	-5.05871	3.755863	2.75E-12	1.56E-09	7	91	8	1630	2067	555		
Solyc10g050880.1.1	-5.02984	3.816082	6.44E-22	3.51E-18	20	26	49	1775	2122	526		
Solyc08g007090.1.1	-4.9973	-0.70711	2.63E-05	0.001556	0	4	0	89	73	9		
Solyc10g079320.1.1	-4.96652	1.192701	1.96E-06	0.000174	6	4	3	334	22	235		
Solyc04g025530.2.1	-4.93945	5.445416	2.21E-21	1.00E-17	29	257	83	4584	7523	1873		
Solyc03g005520.1.1	-4.93008	-0.17556	1.01E-06	9.88E-05	3	0	2	99	126	31		
Solyc01g107400.2.1	-4.92972	-0.49495	6.14E-06	0.000454	0	3	2	110	79	10		
Solyc10g018340.1.1	-4.88057	2.590114	1.80E-09	4.21E-07	2	49	3	696	987	217		
Solyc02g062300.2.1	-4.83545	3.278303	1.28E-06	0.000121	19	28	21	462	3035	31		
Solyc10g076240.1.1	-4.82207	2.431197	2.77E-06	0.00023	18	0	10	677	966	85		
Solyc09g013150.2.1	-4.79992	4.112897	1.01E-22	9.17E-19	21	55	70	2267	2559	584		
Solyc10g076370.1.1	-4.79831	-0.24453	5.05E-05	0.002556	0	7	0	167	39	18		
Solyc01g095080.2.1	-4.76606	4.123513	4.01E-07	4.48E-05	1	173	12	2081	2657	720		
Solyc07g052140.2.1	-4.75209	4.237827	0.000192	0.007755	42	9	82	683	6310	12		
Solyc05g050350.1.1	-4.73323	5.521222	5.76E-23	7.84E-19	79	210	88	4868	8131	1752		
Solyc02g087070.2.1	-4.72693	4.827691	5.28E-20	1.60E-16	35	122	98	3233	5269	721		
Solyc09g098010.2.1	-4.70936	-1.13333	2.92E-05	0.001675	1	0	2	18	81	28		
Solyc11g072930.1.1	-4.70897	1.321983	5.37E-09	1.09E-06	6	10	3	410	257	63		
Solyc06g068900.2.1	-4.67419	0.499115	9.10E-05	0.004159	0	14	0	295	66	24		

Solyc01g106510.2.1	-4.66775	-0.03166	3.77E-05	0.00204	0	0	9	182	63	21
Solyc09g009980.1.1	-4.65471	0.243849	2.02E-07	2.50E-05	0	2	9	162	150	33
Solyc02g094270.1.1	-4.64589	-1.31045	5.29E-05	0.002643	0	3	0	22	77	14
Solyc02g068470.1.1	-4.64104	1.433482	5.40E-08	8.11E-06	8	8	4	451	294	50
Solyc07g052120.2.1	-4.64079	2.301432	3.27E-08	5.24E-06	4	40	5	506	1042	74
Solyc04g064880.2.1	-4.61835	7.034537	5.57E-05	0.002756	179	1094	2	15169	22702	4124
Solyc03g005040.1.1	-4.57603	1.229278	6.05E-10	1.65E-07	1	19	4	322	282	89
Solyc05g012660.1.1	-4.57007	2.433987	2.78E-14	2.61E-11	9	32	10	697	748	203
Solyc02g080090.1.1	-4.54591	0.176251	6.63E-08	9.66E-06	2	7	1	123	176	36
Solyc08g078650.2.1	-4.53467	5.247909	1.18E-11	5.97E-09	95	196	30	5867	4019	1452
Solyc01g073800.2.1	-4.48614	-0.66593	5.21E-05	0.00262	0	6	0	35	141	15
Solyc02g068670.1.1	-4.4328	2.330804	7.93E-18	1.54E-14	6	31	18	425	872	275
Solyc09g011540.2.1	-4.4225	1.687635	2.81E-09	6.09E-07	2	9	26	503	378	76
Solyc07g039570.2.1	-4.41394	2.274891	2.03E-08	3.57E-06	10	37	2	405	1016	151
Solyc11g005840.1.1	-4.40249	2.794592	1.01E-17	1.64E-14	16	29	25	372	1305	510
Solyc04g040130.1.1	-4.3915	4.985111	1.41E-20	5.49E-17	50	220	85	2288	6558	1493
Solyc07g056210.2.1	-4.36304	5.028728	1.02E-17	1.64E-14	62	217	79	4134	4955	1070
Solyc07g051950.2.1	-4.32732	0.932409	3.51E-07	3.97E-05	2	18	2	158	409	43
Solyc03g096190.1.1	-4.32092	1.791438	1.49E-09	3.68E-07	6	29	4	461	467	112
Solyc03g121190.2.1	-4.31873	1.353355	1.51E-09	3.71E-07	6	19	2	295	359	109
Solyc04g054950.2.1	-4.29643	2.857955	1.97E-08	3.50E-06	26	9	30	566	1680	164
Solyc03g020030.2.1	-4.27562	-0.00752	0.000157	0.00651	0	3	9	53	263	7
Solyc11g007470.1.1	-4.25876	1.777585	2.05E-07	2.51E-05	13	19	0	446	375	173
Solyc02g071820.2.1	-4.24837	6.543774	4.60E-13	3.13E-10	326	342	200	7517	19691	3386
Solyc03g019690.1.1	-4.19098	-1.38124	0.000116	0.00514	0	4	0	22	63	18
Solyc01g080570.2.1	-4.18508	4.225948	5.89E-10	1.62E-07	68	105	12	1547	3185	1100
Solyc03g115060.2.1	-4.14322	5.575075	8.26E-17	1.12E-13	75	430	149	4650	8925	1670
Solyc04g072070.2.1	-4.12554	5.017745	5.47E-11	2.33E-08	50	362	41	3526	5229	1323
Solyc11g072140.1.1	-4.12482	0.036797	6.84E-07	7.16E-05	1	12	0	76	141	74
Solyc08g078170.1.1	-4.12467	-1.1767	9.41E-06	0.00065	0	2	3	42	53	21
Solyc11g007390.1.1	-4.11468	2.846812	4.41E-09	9.16E-07	33	28	7	904	724	443
Solyc08g068660.1.1	-4.10883	-1.09292	5.74E-05	0.002827	1	1	3	59	52	12
Solyc08g068630.2.1	-4.08421	-0.26148	5.87E-07	6.32E-05	0	6	5	86	121	31
Solyc02g077520.2.1	-4.07426	-0.72474	0.000229	0.008968	1	6	0	24	142	16
Solyc06g068890.1.1	-4.04977	-0.58617	0.000199	0.007986	0	9	0	89	77	16
Solyc10g078220.1.1	-4.04842	4.804745	0.000174	0.007133	8	463	7	1362	7192	935
Solyc11g007500.1.1	-4.03672	3.370752	4.89E-13	3.25E-10	16	112	24	1287	1244	527
Solyc07g056080.1.1	-3.98664	3.249347	1.88E-17	2.70E-14	17	93	31	880	1349	622
Solyc11g067000.1.1	-3.97896	-0.30451	0.000147	0.006168	0	12	0	122	63	30
Solyc12g045020.1.1	-3.97467	3.240793	6.01E-09	1.19E-06	16	117	11	1039	1505	363
Solyc12g009110.1.1	-3.96165	1.511825	5.41E-07	5.88E-05	4	5	34	255	98	380
Solyc01g098370.1.1	-3.94925	0.53543	1.39E-08	2.59E-06	2	15	4	176	146	81
Solyc05g015850.2.1	-3.93252	4.980049	1.34E-09	3.34E-07	77	357	30	3045	5334	1429
Solyc02g092890.1.1	-3.92893	3.334328	2.25E-18	5.10E-15	31	53	55	1139	1375	500
Solyc09g098080.2.1	-3.90607	5.270326	9.21E-06	0.00064	11	675	41	5103	4252	1938
Solyc07g062480.1.1	-3.90578	0.559433	2.18E-05	0.001315	8	3	5	138	287	26
Solyc02g079890.1.1	-3.9054	5.172334	3.56E-06	0.000283	195	33	125	2404	1238	5684
Solyc06g034370.1.1	-3.87708	-0.37035	9.20E-07	9.05E-05	1	7	3	56	128	37
Solyc07g005110.2.1	-3.87178	3.36809	2.44E-07	2.95E-05	23	132	7	1272	1480	367
Solyc01g073830.1.1	-3.86564	-0.73484	4.57E-05	0.002365	3	2	2	35	107	27
Solyc01g109140.2.1	-3.85883	4.180731	1.72E-05	0.001075	59	183	15	1372	4611	156
Solyc08g068710.1.1	-3.85157	4.718161	1.56E-13	1.21E-10	45	202	195	2722	5068	609
Solyc07g063730.1.1	-3.84424	3.889629	5.25E-11	2.27E-08	28	184	34	1501	2550	555
Solyc03g120260.2.1	-3.82775	5.671714	6.23E-22	3.51E-18	183	281	253	4375	8927	2552
Solyc08g078180.1.1	-3.82599	2.208523	5.66E-08	8.43E-06	8	63	6	421	823	184
Solyc08g006740.2.1	-3.82232	4.72071	3.96E-14	3.60E-11	55	272	111	3314	3961	779
Solyc04g079240.2.1	-3.82122	0.999378	1.21E-05	0.0008	5	13	12	27	552	82
Solyc06g066590.2.1	-3.81429	3.539183	6.12E-07	6.49E-05	15	182	12	1256	1977	371
Solyc03g098670.1.1	-3.79332	0.844225	6.99E-05	0.003307	1	31	0	174	302	62
Solyc05g009120.2.1	-3.78513	3.625855	3.26E-18	6.82E-15	34	94	69	1163	2077	558
Solyc02g077290.1.1	-3.7772	3.449558	5.24E-09	1.07E-06	26	139	17	1436	1393	407
Solyc07g008120.2.1	-3.76446	-0.03193	4.78E-06	0.000365	1	14	1	106	103	57
Solyc02g067700.1.1	-3.75765	0.625203	5.46E-06	0.000406	7	6	8	241	156	33
Solyc05g010020.2.1	-3.73452	4.431066	1.60E-09	3.84E-07	64	262	27	2115	3503	984
Solyc06g069650.2.1	-3.72862	0.511391	2.42E-06	0.000206	1	6	18	106	295	38
Solyc07g018200.1.1	-3.71116	3.22912	8.88E-11	3.56E-08	31	98	22	1116	1305	374
Solyc08g068680.2.1	-3.70642	6.0651	1.02E-12	5.93E-10	119	950	193	7317	9347	3366
Solyc01g009320.1.1	-3.70517	-0.41491	5.22E-05	0.002621	1	0	11	53	126	34
Solyc03g115610.2.1	-3.68474	5.052435	7.69E-19	1.90E-15	89	332	160	3822	4760	1391
Solyc12g088000.1.1	-3.68297	-1.44037	0.000252	0.009666	1	0	4	20	52	22
Solyc09g075820.2.1	-3.68217	7.090041	1.39E-15	1.58E-12	413	1425	478	15309	20376	5583
Solyc02g070090.1.1	-3.65745	2.259051	1.93E-07	2.41E-05	5	64	26	677	658	89
Solyc11g007450.1.1	-3.6543	0.637143	1.71E-08	3.08E-06	2	11	15	199	145	82
Solyc12g049030.1.1	-3.62328	5.853654	7.97E-07	8.01E-05	113	980	52	5258	10493	2269
Solyc03g093890.2.1	-3.61677	4.326778	2.97E-13	2.25E-10	81	189	57	2248	2805	918
Solyc09g011330.1.1	-3.60239	4.379799	4.82E-10	1.35E-07	58	285	43	1687	3919	895
Solyc02g092120.2.1	-3.59468	2.484837	4.57E-10	1.31E-07	14	78	13	541	779	323
Solyc10g083290.1.1	-3.57851	6.189465	1.44E-13	1.15E-10	256	806	230	7908	11248	2941
Solyc12g096960.1.1	-3.55744	3.401767	5.60E-10	1.56E-07	39	123	25	928	1882	441
Solyc01g009480.2.1	-3.55419	0.01198	4.11E-06	0.00032	0	11	9	125	106	43
Solyc12g006840.1.1	-3.54059	4.727635	5.55E-16	6.57E-13	74	315	126	2396	4619	1133
Solyc05g046110.2.1	-3.53707	-0.44879	0.000238	0.009242	4	7	0	97	54	36
Solyc03g043900.1.1	-3.47692	1.679531	7.76E-07	7.89E-05	7	52	7	359	483	104

Solyc07g066550.2.1	-3.47562	3.056167	9.01E-12	4.63E-09	26	91	47	669	1604	312
Solyc05g055030.1.1	-3.47209	1.165584	2.69E-07	3.22E-05	4	9	33	203	392	77
Solyc05g008220.2.1	-3.46702	3.442557	8.09E-11	3.29E-08	50	87	55	1437	1398	346
Solyc01g103650.2.1	-3.44908	6.238386	2.75E-16	3.40E-13	289	657	499	5458	15254	3081
Solyc07g048070.2.1	-3.43712	5.456882	1.19E-10	4.37E-08	58	804	180	4298	5932	2635
Solyc01g005160.2.1	-3.42852	0.084369	2.99E-05	0.001701	1	3	18	44	197	67
Solyc03g034360.2.1	-3.42617	3.97173	5.58E-13	3.62E-10	34	248	66	1218	2376	1052
Solyc02g064830.2.1	-3.42141	4.247167	2.96E-15	3.22E-12	43	278	99	2156	2053	1182
Solyc12g017700.1.1	-3.41198	0.923271	4.58E-05	0.002365	5	24	10	367	93	38
Solyc02g091070.2.1	-3.40814	2.974638	2.23E-08	3.82E-06	22	87	58	1032	1187	135
Solyc09g011870.1.1	-3.40028	7.163588	1.14E-08	2.16E-06	943	376	836	14308	23214	5855
Solyc01g006950.2.1	-3.39727	5.751446	1.55E-11	7.55E-09	189	775	147	5693	7667	2571
Solyc06g062920.2.1	-3.39626	3.694802	8.18E-08	1.14E-05	18	259	34	1069	2082	725
Solyc08g074680.2.1	-3.39406	4.794609	3.28E-06	0.000265	209	62	106	4066	3178	776
Solyc11g044840.1.1	-3.39285	5.256059	5.22E-08	7.90E-06	173	493	57	4413	5476	1452
Solyc01g008690.2.1	-3.38752	1.884926	1.61E-07	2.06E-05	19	37	11	497	413	133
Solyc04g072620.1.1	-3.35446	1.218661	2.38E-05	0.001424	1	25	31	411	157	57
Solyc02g079530.2.1	-3.34301	2.948855	1.97E-09	4.44E-07	14	127	43	939	1009	295
Solyc10g086710.1.1	-3.33253	2.592404	1.13E-10	4.22E-08	24	59	42	808	681	224
Solyc05g007110.2.1	-3.32834	0.683755	1.09E-05	0.000735	12	5	9	140	251	69
Solyc10g076510.1.1	-3.32656	6.600315	5.48E-12	2.87E-09	484	902	477	11543	14225	3113
Solyc05g054380.1.1	-3.32015	4.97216	2.62E-09	5.79E-07	205	146	172	3301	5193	1004
Solyc10g055820.1.1	-3.31965	1.93381	6.47E-08	9.48E-06	8	64	17	312	696	148
Solyc07g008620.1.1	-3.31375	4.956735	1.37E-17	2.08E-14	151	290	173	2889	4597	1675
Solyc02g065090.2.1	-3.3126	0.50025	0.000202	0.008066	4	21	5	43	374	33
Solyc12g008670.1.1	-3.30601	-0.76058	0.000167	0.006891	0	8	5	80	35	30
Solyc03g112030.1.1	-3.30558	0.84551	1.75E-07	2.22E-05	7	16	14	159	304	63
Solyc05g014590.2.1	-3.29734	2.181347	6.75E-05	0.003232	40	14	8	554	609	147
Solyc03g116700.2.1	-3.29335	7.278339	1.42E-08	2.63E-06	318	3085	490	16440	23252	6447
Solyc03g007200.1.1	-3.29246	0.732638	1.40E-06	0.000132	9	10	13	163	88	168
Solyc05g052680.1.1	-3.28498	3.005845	0.000159	0.006584	46	1	101	684	1210	461
Solyc03g043700.2.1	-3.27778	3.22367	4.58E-14	3.90E-11	27	76	114	776	1554	464
Solyc08g021880.1.1	-3.27771	0.49976	3.47E-05	0.001913	4	22	5	167	207	29
Solyc02g092860.2.1	-3.26295	4.524064	1.17E-14	1.18E-11	106	245	134	2622	3130	959
Solyc05g052670.1.1	-3.26219	5.964935	4.30E-06	0.000334	264	57	1020	4530	11854	2853
Solyc05g008210.2.1	-3.25438	2.707591	8.32E-09	1.60E-06	30	64	44	751	1028	163
Solyc03g113120.2.1	-3.25283	-1.5184	0.000259	0.009953	0	5	2	24	37	23
Solyc12g100270.1.1	-3.24948	3.40418	1.51E-08	2.76E-06	38	155	49	715	2293	332
Solyc05g053150.1.1	-3.23429	5.115489	4.06E-11	1.81E-08	176	336	207	5411	2170	1737
Solyc10g083690.2.1	-3.23411	5.485248	5.43E-07	5.88E-05	245	472	180	5389	7709	585
Solyc02g092870.1.1	-3.22881	1.285715	0.000154	0.006448	18	16	8	410	219	37
Solyc04g072000.2.1	-3.22745	4.954733	7.09E-10	1.88E-07	42	557	245	2528	5683	1270
Solyc01g007960.2.1	-3.21299	3.834394	2.07E-09	4.63E-07	53	230	51	1399	2314	546
Solyc06g076910.1.1	-3.20741	0.047233	2.91E-05	0.001675	0	14	12	90	162	39
Solyc12g100260.1.1	-3.20502	3.459261	3.95E-06	0.000309	22	239	29	511	2460	500
Solyc04g058170.1.1	-3.18817	2.519798	3.40E-07	3.89E-05	17	107	18	689	759	202
Solyc01g108360.2.1	-3.18601	0.804791	2.62E-07	3.14E-05	6	9	25	132	280	89
Solyc11g006030.1.1	-3.18562	3.508588	7.05E-13	4.36E-10	37	153	89	914	2019	501
Solyc11g007490.1.1	-3.1815	3.032311	2.36E-10	7.75E-08	35	97	56	1024	1043	281
Solyc12g088390.1.1	-3.17847	3.350542	7.63E-07	7.79E-05	60	94	54	1017	1963	166
Solyc01g096720.2.1	-3.16708	2.94808	5.23E-06	0.000393	46	68	44	1447	431	179
Solyc01g105630.2.1	-3.16281	2.359647	6.22E-11	2.61E-08	10	78	47	350	796	346
Solyc03g119660.1.1	-3.15047	1.913835	3.05E-07	3.56E-05	20	18	44	462	501	113
Solyc07g042430.1.1	-3.14532	1.514305	3.47E-05	0.001913	0	44	39	227	508	113
Solyc05g008640.1.1	-3.14481	2.586451	2.66E-06	0.000222	40	31	48	1031	392	183
Solyc08g067610.2.1	-3.13668	5.073129	8.38E-13	4.96E-10	92	572	231	2523	6167	1532
Solyc01g009810.2.1	-3.13439	3.682975	3.94E-05	0.002108	22	322	20	561	2576	794
Solyc02g031990.1.1	-3.12845	3.382327	2.30E-10	7.63E-08	21	133	132	1121	1588	360
Solyc05g010000.1.1	-3.12782	0.962421	3.38E-05	0.001875	5	4	40	256	180	87
Solyc10g076480.1.1	-3.12683	5.722384	3.13E-10	9.58E-08	331	560	203	6466	6388	2212
Solyc06g036420.1.1	-3.12667	0.680551	1.67E-06	0.000153	2	19	21	170	224	57
Solyc04g024780.1.1	-3.12454	0.691754	3.25E-05	0.001814	2	33	8	146	278	46
Solyc03g098740.1.1	-3.11922	6.117079	3.26E-05	0.001818	111	1822	94	7878	8945	3128
Solyc07g062490.1.1	-3.10664	5.856534	3.70E-06	0.000291	449	317	300	4517	12913	997
Solyc12g007050.1.1	-3.09278	5.029553	3.16E-13	2.33E-10	168	428	168	3397	4564	1500
Solyc09g014820.2.1	-3.08116	0.280651	5.77E-05	0.002836	1	26	6	105	193	45
Solyc08g006750.2.1	-3.06884	4.313362	3.94E-12	2.15E-09	71	291	163	2277	2868	654
Solyc11g073080.1.1	-3.06472	1.296969	3.10E-06	0.000252	9	23	30	305	347	49
Solyc12g005940.1.1	-3.05023	3.725685	4.08E-07	4.54E-05	90	129	64	2081	1162	385
Solyc08g062490.2.1	-3.04884	5.034167	3.83E-10	1.13E-07	91	657	180	3768	4317	1326
Solyc12g005720.1.1	-3.03881	5.060315	6.86E-13	4.35E-10	100	575	262	2975	5608	1355
Solyc03g114890.2.1	-3.03121	3.589484	1.89E-09	4.29E-07	42	222	64	1382	1557	493
Solyc11g068940.1.1	-3.02095	2.324652	1.54E-09	3.75E-07	18	60	56	375	924	199
Solyc12g044950.1.1	-3.01816	2.879624	2.19E-10	7.35E-08	20	85	103	570	1316	311
Solyc03g020060.2.1	-3.01065	1.411019	8.82E-06	0.000618	23	19	11	144	258	295
Solyc04g078660.1.1	-3.01042	4.878217	7.93E-14	6.54E-11	93	400	339	3332	4124	1056
Solyc10g085010.1.1	-3.0044	7.52461	3.99E-10	1.16E-07	740	3531	740	19176	23863	9441
Solyc08g068610.2.1	-2.99418	3.993274	2.30E-08	3.86E-06	114	154	82	1825	2267	514
Solyc08g007460.2.1	-2.99192	7.608505	7.46E-15	7.82E-12	968	2762	1295	18739	28004	9675
Solyc08g068720.1.1	-2.98179	0.319037	1.83E-05	0.001127	4	18	11	175	98	49
Solyc03g114230.1.1	-2.97023	3.175073	7.05E-06	0.000508	64	22	116	699	1754	286
Solyc06g074140.1.1	-2.96873	3.854686	0.000155	0.006481	87	11	250	1457	2426	410
Solyc07g063710.1.1	-2.94892	1.384623	7.57E-05	0.003529	18	12	30	186	558	50

Solyc02g077370.1.1	-2.93429	4.696575	3.94E-12	2.15E-09	86	513	172	2163	4002	1363
Solyc10g008080.1.1	-2.92593	-0.09118	1.73E-05	0.001075	2	10	14	108	87	46
Solyc01g010060.2.1	-2.91574	3.513248	1.70E-07	2.16E-05	48	237	41	1289	1431	492
Solyc07g006420.1.1	-2.91227	3.761383	1.11E-07	1.47E-05	33	305	90	1612	1792	456
Solyc09g082230.1.1	-2.9097	3.998628	9.72E-11	3.78E-08	104	153	141	1710	2296	589
Solyc10g008730.2.1	-2.90138	2.790255	2.41E-08	3.96E-06	41	98	41	824	837	268
Solyc11g069220.1.1	-2.90065	1.612142	3.78E-09	7.91E-07	16	37	29	263	412	174
Solyc02g082950.2.1	-2.89797	2.669872	4.91E-08	7.48E-06	42	54	63	707	908	202
Solyc07g049550.2.1	-2.89077	5.915388	3.56E-06	0.000283	451	316	644	5515	12071	955
Solyc07g062630.1.1	-2.88912	0.714613	4.68E-07	5.16E-05	9	14	20	137	216	93
Solyc08g068600.2.1	-2.88387	5.893974	4.80E-11	2.11E-08	191	1160	501	6799	8009	2169
Solyc08g075550.2.1	-2.87963	4.80916	3.82E-13	2.74E-10	138	471	183	3111	3198	1462
Solyc09g091670.2.1	-2.87415	5.57515	5.97E-05	0.002896	96	1424	84	4126	7766	2071
Solyc01g107390.2.1	-2.87122	2.974612	2.93E-07	3.44E-05	65	55	60	1001	842	312
Solyc11g010210.1.1	-2.85613	1.117774	4.31E-06	0.000334	5	30	32	196	367	62
Solyc08g066020.1.1	-2.84512	3.942338	6.45E-06	0.000471	139	92	105	1750	2399	331
Solyc12g056600.2.1	-2.84189	4.828691	4.35E-06	0.000336	275	104	210	3737	1619	1963
Solyc01g005800.2.1	-2.8403	0.446336	0.000107	0.004751	7	12	18	205	109	37
Solyc06g073760.2.1	-2.83741	3.575476	3.85E-06	0.000302	86	170	45	1482	1605	301
Solyc12g009520.1.1	-2.83263	3.963049	1.85E-09	4.23E-07	104	209	98	1503	2410	594
Solyc02g069800.1.1	-2.83112	7.17188	6.17E-10	1.65E-07	1061	1704	848	15717	19295	5907
Solyc05g005700.2.1	-2.82357	4.124513	7.64E-08	1.08E-05	46	417	118	1553	2926	638
Solyc05g056080.2.1	-2.82353	2.294991	1.49E-08	2.74E-06	18	71	62	487	748	170
Solyc02g092820.2.1	-2.80541	3.962645	6.79E-08	9.79E-06	54	367	83	1732	2003	641
Solyc02g082910.2.1	-2.80128	5.522192	3.72E-05	0.002025	497	306	100	5208	5657	1976
Solyc10g079810.1.1	-2.80032	2.96565	4.79E-10	1.35E-07	51	99	64	731	1083	382
Solyc04g016230.2.1	-2.78962	3.360734	1.19E-06	0.000114	77	74	114	1429	1153	256
Solyc03g116900.2.1	-2.78872	8.270929	4.35E-14	3.83E-11	1374	5009	3250	35311	37247	13536
Solyc01g096420.2.1	-2.78308	5.276477	1.34E-07	1.75E-05	199	828	142	2935	6816	1672
Solyc03g094120.2.1	-2.77127	3.876162	3.94E-05	0.002108	24	460	50	1730	1492	755
Solyc07g055400.1.1	-2.77125	2.420199	3.11E-07	3.61E-05	28	65	66	243	1149	228
Solyc09g057960.1.1	-2.77036	1.117927	0.000114	0.005077	18	23	13	156	430	52
Solyc04g076730.1.1	-2.76875	1.654952	1.97E-06	0.000174	26	17	37	312	377	164
Solyc12g006380.1.1	-2.75388	3.31116	2.22E-06	0.00019	87	73	84	1429	900	332
Solyc06g066630.1.1	-2.75226	1.920505	1.16E-06	0.000112	28	39	35	257	705	145
Solyc03g080190.2.1	-2.7425	5.423284	2.10E-06	0.000182	376	498	186	3218	8483	1249
Solyc01g079140.2.1	-2.74183	3.040245	1.82E-07	2.28E-05	54	121	60	1112	824	287
Solyc02g093250.2.1	-2.73761	3.84234	8.23E-10	2.12E-07	60	241	166	1813	1653	491
Solyc05g012180.2.1	-2.73488	3.646578	6.15E-10	1.65E-07	61	184	149	1454	1636	419
Solyc02g072070.2.1	-2.73175	1.449915	5.31E-05	0.002648	10	66	13	391	207	109
Solyc01g086680.2.1	-2.7312	2.185286	1.45E-05	0.000931	15	114	24	546	580	135
Solyc03g005480.2.1	-2.72828	1.206297	4.03E-05	0.002138	20	26	13	257	302	72
Solyc08g080130.2.1	-2.72695	7.585964	1.24E-11	6.14E-09	1316	2305	1834	19930	27216	7444
Solyc03g119370.1.1	-2.72115	3.519858	2.31E-08	3.86E-06	43	191	151	1373	1541	316
Solyc07g017880.2.1	-2.71852	2.629724	3.87E-05	0.002086	52	17	91	844	550	247
Solyc06g035810.1.1	-2.70806	0.595418	2.26E-05	0.001355	9	19	16	86	277	65
Solyc10g007280.2.1	-2.70617	7.251182	2.00E-07	2.49E-05	1418	1585	740	16113	20161	6453
Solyc09g075920.1.1	-2.70314	4.400869	2.71E-14	2.61E-11	94	377	227	1679	3185	1136
Solyc11g007890.1.1	-2.67325	2.301251	5.40E-06	0.000403	35	21	87	396	810	201
Solyc09g090970.2.1	-2.66301	2.127901	1.13E-05	0.000757	19	111	17	250	718	269
Solyc08g006470.2.1	-2.66294	4.213532	4.96E-05	0.002521	204	96	122	2573	2005	486
Solyc08g068770.1.1	-2.6622	6.584746	2.28E-08	3.86E-06	350	2439	645	8955	14524	3904
Solyc09g005560.2.1	-2.66016	1.97956	1.17E-05	0.00078	8	99	39	538	325	166
Solyc05g053600.2.1	-2.65688	6.33829	6.79E-08	9.79E-06	633	735	927	9376	11636	2006
Solyc06g069130.2.1	-2.65602	3.725551	2.06E-07	2.52E-05	33	339	123	1475	1640	536
Solyc08g062360.2.1	-2.6458	5.908911	2.13E-10	7.27E-08	229	1473	451	5328	8130	3280
Solyc02g085360.2.1	-2.64268	5.038412	1.10E-10	4.17E-08	251	276	430	3481	4257	1386
Solyc04g058160.1.1	-2.64186	3.121852	1.82E-09	4.21E-07	52	136	90	1021	934	389
Solyc01g073820.2.1	-2.63486	1.898942	3.58E-07	4.03E-05	14	84	32	254	578	211
Solyc04g016000.2.1	-2.63442	3.367943	1.60E-08	2.91E-06	42	154	166	912	1755	323
Solyc01g073840.1.1	-2.62193	0.2839	0.000101	0.004547	4	20	17	141	144	35
Solyc09g011630.2.1	-2.60343	3.91553	2.38E-11	1.12E-08	85	195	225	1279	2370	641
Solyc05g044490.2.1	-2.60285	1.471086	9.31E-05	0.004228	23	39	18	246	481	64
Solyc02g062550.2.1	-2.60159	3.453567	2.81E-09	6.09E-07	54	235	91	1202	1154	575
Solyc03g123680.1.1	-2.60075	4.567501	6.63E-05	0.00319	279	176	90	2769	3068	744
Solyc09g007730.2.1	-2.60038	2.345277	1.07E-07	1.42E-05	24	60	91	412	845	192
Solyc09g091540.1.1	-2.5984	2.180844	6.18E-06	0.000455	27	37	84	224	284	601
Solyc02g036480.1.1	-2.5917	6.056116	4.39E-08	6.80E-06	244	1844	410	6571	8365	3355
Solyc02g090110.2.1	-2.58841	5.911885	2.93E-11	1.35E-08	395	914	643	6194	8553	2176
Solyc07g045530.1.1	-2.58339	3.605047	3.69E-08	5.88E-06	64	279	79	892	1907	638
Solyc06g009290.2.1	-2.53834	6.194849	3.02E-11	1.37E-08	429	1052	1077	8568	8956	2560
Solyc01g079940.2.1	-2.52414	2.71802	1.52E-07	1.95E-05	41	99	86	558	1096	218
Solyc09g011550.2.1	-2.52275	3.938398	1.62E-06	0.00015	44	448	127	1072	2677	662
Solyc09g011260.1.1	-2.51156	2.142012	2.23E-05	0.001341	41	67	24	497	525	162
Solyc04g054690.2.1	-2.50634	3.622421	6.30E-06	0.000461	77	295	60	1258	1776	401
Solyc02g031790.1.1	-2.49357	4.178495	3.34E-10	1.01E-07	120	297	213	1978	2262	697
Solyc08g028780.1.1	-2.4903	3.404115	1.73E-08	3.09E-06	54	202	138	713	1911	428
Solyc04g064870.2.1	-2.48544	6.625528	3.99E-10	1.16E-07	388	2409	1139	7781	15544	4548
Solyc12g036390.1.1	-2.48138	5.458755	3.56E-09	7.64E-07	301	808	422	5402	4990	1485
Solyc01g095960.2.1	-2.47885	3.834353	1.30E-05	0.000852	111	86	273	762	3032	466
Solyc06g008620.1.1	-2.47679	1.383214	2.26E-05	0.001355	12	59	26	198	448	87
Solyc12g056360.1.1	-2.4694	3.667091	7.37E-08	1.05E-05	99	236	91	1289	1580	570
Solyc08g068730.1.1	-2.46922	6.121016	2.41E-08	3.96E-06	246	1743	863	8349	8044	2433

Solyc02g080080.2.1	-2.46142	6.191701	4.67E-07	5.16E-05	553	1505	438	8039	9724	2388
Solyc12g045030.1.1	-2.45414	7.157621	1.26E-08	2.37E-06	1047	2729	1183	10610	24877	5532
Solyc12g006280.1.1	-2.43915	0.250593	0.000141	0.005973	4	18	23	125	141	41
Solyc11g005350.1.1	-2.43398	3.252309	4.79E-05	0.002452	112	82	70	1198	995	327
Solyc04g071070.2.1	-2.43023	1.424426	0.000193	0.007785	21	56	14	193	478	85
Solyc08g077330.2.1	-2.42638	5.726923	3.96E-05	0.002114	476	1059	193	7478	4458	1830
Solyc04g077300.2.1	-2.42625	2.879429	5.44E-07	5.88E-05	46	120	106	848	930	216
Solyc04g005050.1.1	-2.42623	5.823508	9.39E-11	3.71E-08	314	1226	647	5479	7937	2224
Solyc04g079250.2.1	-2.42587	4.373534	2.27E-08	3.86E-06	55	495	348	1202	3444	1192
Solyc09g007520.2.1	-2.4249	4.51396	7.21E-06	0.000517	192	425	152	2847	2840	539
Solyc10g076780.1.1	-2.42478	3.030769	1.84E-06	0.000165	79	82	97	863	1060	293
Solyc04g074780.2.1	-2.40154	1.471262	2.78E-05	0.001619	20	49	30	368	222	109
Solyc10g084960.1.1	-2.39748	3.220532	8.95E-05	0.004106	22	330	63	759	1482	365
Solyc02g091700.2.1	-2.39743	3.944219	2.08E-06	0.000181	82	370	158	1591	2345	371
Solyc03g119240.2.1	-2.39352	3.498767	1.82E-07	2.28E-05	42	274	161	1148	1539	404
Solyc02g062790.2.1	-2.38681	1.68725	0.000172	0.007044	20	58	43	482	279	65
Solyc05g056380.2.1	-2.38496	1.057139	3.64E-06	0.000288	9	39	34	144	293	117
Solyc09g098160.2.1	-2.3838	2.880017	8.44E-08	1.17E-05	24	174	117	709	879	371
Solyc02g085300.2.1	-2.37701	3.227817	2.44E-06	0.000207	52	247	73	1097	1000	371
Solyc07g008600.1.1	-2.36849	1.699268	1.66E-06	0.000153	20	61	43	310	418	136
Solyc09g008740.1.1	-2.36351	0.601279	0.000162	0.006713	13	25	14	120	224	59
Solyc11g008200.1.1	-2.35767	1.790585	1.43E-05	0.000925	32	49	37	367	416	129
Solyc02g061770.2.1	-2.35576	4.931059	0.00016	0.00663	248	775	81	3492	3782	963
Solyc09g007890.1.1	-2.34402	2.096636	0.000185	0.007532	12	144	34	333	723	140
Solyc09g082240.2.1	-2.33788	1.86615	3.16E-05	0.001777	16	72	64	468	385	98
Solyc08g065210.1.1	-2.33575	1.415578	7.83E-05	0.003641	15	65	26	356	180	121
Solyc11g066800.1.1	-2.33516	4.320024	6.81E-05	0.00325	220	256	156	2955	1728	484
Solyc06g069740.1.1	-2.33476	1.562358	0.000195	0.00784	20	81	12	298	322	143
Solyc09g011590.2.1	-2.3316	7.350452	1.26E-10	4.53E-08	726	4075	2273	13547	22958	8046
Solyc11g017280.1.1	-2.32716	4.996328	3.00E-10	9.30E-08	231	675	324	3053	4062	1462
Solyc01g096430.2.1	-2.32303	4.432991	8.49E-08	1.17E-05	144	388	321	1813	3717	596
Solyc12g011090.1.1	-2.32141	0.720779	5.32E-05	0.002648	9	41	15	137	197	89
Solyc08g021870.1.1	-2.31973	0.211205	7.32E-05	0.003428	4	20	23	85	145	65
Solyc01g009930.1.1	-2.31732	2.873284	0.000193	0.007769	30	261	30	505	1148	363
Solyc12g010710.1.1	-2.3148	3.55012	1.37E-07	1.77E-05	104	171	147	910	1873	468
Solyc10g079930.1.1	-2.31447	6.68767	4.91E-12	2.62E-09	695	2317	1092	7369	11542	7985
Solyc04g078290.2.1	-2.31367	7.370713	8.58E-10	2.18E-07	988	4291	1548	13925	21354	9140
Solyc03g020010.1.1	-2.31338	2.228783	5.12E-05	0.002583	41	90	38	296	878	157
Solyc01g073890.2.1	-2.31247	3.964608	1.58E-05	0.000997	85	486	85	1766	1679	652
Solyc09g075910.1.1	-2.30184	4.048274	2.34E-08	3.89E-06	99	386	198	1274	2686	651
Solyc01g007050.1.1	-2.3014	1.981541	1.36E-05	0.000889	11	122	46	348	450	228
Solyc01g086660.2.1	-2.3006	1.94751	4.95E-05	0.002521	17	68	80	518	372	103
Solyc10g055200.1.1	-2.29705	6.542601	1.82E-09	4.21E-07	708	1914	996	8041	13774	3813
Solyc11g018800.1.1	-2.29208	2.153229	1.93E-05	0.001187	25	90	69	388	735	113
Solyc10g017570.2.1	-2.28276	3.275173	8.97E-08	1.22E-05	59	194	148	683	1627	391
Solyc02g092110.2.1	-2.27861	4.854965	2.45E-10	7.94E-08	257	483	335	2171	4191	1504
Solyc03g026230.1.1	-2.27852	3.629619	4.56E-06	0.000351	60	255	226	1787	982	376
Solyc12g097000.1.1	-2.27059	2.093893	4.04E-06	0.000315	42	65	46	378	531	210
Solyc09g091550.2.1	-2.26918	6.246193	7.01E-05	0.003312	1052	463	645	5178	5739	7766
Solyc05g009230.1.1	-2.2649	3.411048	5.35E-06	0.0004	122	144	87	872	1502	495
Solyc09g090470.2.1	-2.26045	3.443191	1.39E-06	0.000131	82	221	130	873	1827	339
Solyc09g011990.1.1	-2.24952	2.229277	4.71E-06	0.00036	39	69	73	376	735	169
Solyc06g062330.1.1	-2.24398	2.878622	2.44E-05	0.00145	58	91	141	423	1477	218
Solyc05g010030.2.1	-2.24047	1.759372	6.08E-06	0.000451	17	80	50	233	527	156
Solyc04g072800.2.1	-2.23851	3.131108	1.22E-07	1.60E-05	62	137	161	959	971	353
Solyc06g071070.1.1	-2.2378	5.07561	9.34E-08	1.26E-05	295	447	555	3174	5092	1007
Solyc02g068680.1.1	-2.23324	4.216433	3.11E-07	3.61E-05	71	609	196	1277	2449	1206
Solyc07g056200.2.1	-2.2316	6.524314	2.92E-07	3.44E-05	768	2269	604	8934	10356	4773
Solyc07g040890.1.1	-2.22493	3.031104	5.22E-06	0.000393	60	221	56	649	1093	429
Solyc01g107780.2.1	-2.21883	6.14702	3.66E-09	7.72E-07	710	1082	823	6646	8751	3360
Solyc05g051530.2.1	-2.2125	4.791967	2.05E-06	0.000179	201	781	182	2537	3771	1134
Solyc04g079820.2.1	-2.20823	1.828605	1.90E-06	0.000169	18	54	85	288	446	191
Solyc03g114500.2.1	-2.20427	5.382302	1.07E-10	4.12E-08	291	848	640	3886	5637	1672
Solyc08g014570.2.1	-2.20094	6.671688	2.02E-06	0.000178	1208	920	1349	10043	13022	4085
Solyc04g071890.2.1	-2.199	6.486671	5.42E-08	8.11E-06	822	1579	1097	5340	15994	3868
Solyc11g011240.1.1	-2.19118	6.03074	1.09E-05	0.000737	808	680	706	5861	9965	2096
Solyc02g077040.2.1	-2.1911	8.686555	1.07E-07	1.42E-05	3175	11001	3116	36308	47803	23403
Solyc08g067340.2.1	-2.18343	6.793446	0.000152	0.006363	1605	1189	543	11460	11487	5618
Solyc04g051690.2.1	-2.18254	4.935277	8.80E-10	2.22E-07	249	607	409	3342	3085	1426
Solyc05g041540.2.1	-2.17949	4.711167	5.92E-07	6.33E-05	237	520	287	2686	3561	790
Solyc03g007380.1.1	-2.17786	2.382521	2.94E-05	0.001681	36	54	133	196	858	357
Solyc08g021820.2.1	-2.17619	0.90395	0.000204	0.00815	17	34	22	171	244	68
Solyc02g083850.2.1	-2.1734	2.767195	0.000104	0.004662	82	101	55	609	1001	224
Solyc09g014990.2.1	-2.1727	6.580421	2.92E-10	9.13E-08	533	2315	1571	8998	12179	4186
Solyc03g097840.2.1	-2.16972	6.578902	1.61E-09	3.84E-07	832	1679	1383	9413	12008	3885
Solyc07g065250.2.1	-2.16797	4.000105	7.25E-08	1.04E-05	132	269	263	1555	2166	555
Solyc08g074970.1.1	-2.16691	3.693598	2.85E-07	3.38E-05	105	235	200	1365	1604	442
Solyc05g053330.2.1	-2.15579	4.773637	1.72E-05	0.001074	300	164	549	3110	2736	1157
Solyc03g115930.1.1	-2.15451	5.74896	1.65E-06	0.000152	470	1387	363	5765	5745	2374
Solyc04g074680.1.1	-2.14726	4.750478	5.88E-05	0.002877	81	1085	221	2591	3173	1256
Solyc01g067020.2.1	-2.14539	5.900382	1.47E-06	0.000137	642	1071	554	5862	8043	2056
Solyc03g122350.2.1	-2.14491	7.636597	1.20E-10	4.37E-08	1615	3961	2898	18424	24348	9447
Solyc10g085860.1.1	-2.14116	2.476448	0.00013	0.005618	13	206	68	356	799	320

Solyc03g119970.2.1	-2.13548	5.358401	7.61E-10	1.99E-07	307	793	702	4708	3997	1751
Solyc03g113080.2.1	-2.1333	3.121455	2.71E-06	0.000225	52	228	122	757	1283	302
Solyc01g060020.2.1	-2.13308	8.877529	2.17E-06	0.000187	4038	11425	4394	37420	77106	15333
Solyc01g080800.2.1	-2.12605	4.302382	6.92E-05	0.003296	66	779	171	1070	3212	1093
Solyc06g035960.2.1	-2.11791	8.104329	1.81E-09	4.21E-07	1938	5768	4772	29611	30735	10998
Solyc08g066270.1.1	-2.11788	1.672928	3.16E-05	0.001777	26	74	36	279	406	139
Solyc01g099910.2.1	-2.11603	3.86057	1.42E-05	0.000924	91	343	241	1412	2238	311
Solyc09g007900.2.1	-2.11076	7.763018	4.37E-13	3.05E-10	1497	4529	3929	18600	24193	12999
Solyc07g043320.2.1	-2.10622	7.564384	8.13E-09	1.57E-06	1490	3707	3177	18627	24772	6767
Solyc03g116890.2.1	-2.1012	5.711483	4.59E-09	9.47E-07	446	810	1008	4714	7385	1919
Solyc04g025650.2.1	-2.09913	4.185065	0.000102	0.004595	214	202	267	1250	3456	436
Solyc03g116690.2.1	-2.09618	2.481363	5.02E-06	0.000381	30	165	73	358	813	306
Solyc03g119250.2.1	-2.08854	6.006091	1.76E-06	0.000161	353	2178	629	6904	6726	2805
Solyc01g088400.2.1	-2.08239	3.825859	8.64E-08	1.18E-05	137	215	242	1486	1106	877
Solyc01g106620.2.1	-2.07909	7.627824	0.000186	0.007552	2285	4524	854	16775	29081	7331
Solyc09g014280.1.1	-2.0771	6.329902	2.05E-07	2.51E-05	910	741	1488	5555	10635	4869
Solyc01g067120.2.1	-2.06868	2.683668	4.60E-06	0.000353	24	192	115	442	867	367
Solyc08g068870.2.1	-2.067	8.892377	0.000216	0.008525	7037	6179	2582	46740	55121	21011
Solyc08g062330.2.1	-2.06371	3.321667	4.45E-08	6.80E-06	78	206	171	902	1182	486
Solyc06g082570.1.1	-2.06355	5.938766	7.15E-05	0.00336	852	601	692	6782	7155	1988
Solyc03g044900.2.1	-2.06141	6.217918	4.21E-05	0.002195	547	2338	599	6621	11635	1996
Solyc01g106500.2.1	-2.04516	5.639387	7.36E-07	7.56E-05	578	827	576	4950	6155	1877
Solyc02g080890.2.1	-2.04348	5.171058	8.44E-07	8.39E-05	245	1059	434	3889	4192	1236
Solyc12g056960.1.1	-2.02993	4.112274	7.80E-07	7.90E-05	154	335	287	1227	2892	597
Solyc09g011270.2.1	-2.02456	2.924449	1.53E-05	0.000971	74	173	84	740	922	291
Solyc08g016210.2.1	-2.0217	7.477079	9.40E-05	0.004253	1689	5110	1109	18401	22790	5502
Solyc02g079590.2.1	-2.00321	6.397926	7.33E-11	3.02E-08	772	1536	1521	5818	11293	4845
Solyc01g009690.1.1	-1.99909	4.559132	6.62E-06	0.000482	191	715	213	2489	2611	908
Solyc01g014840.2.1	-1.98672	4.43196	2.70E-08	4.41E-06	179	494	351	2171	2428	896
Solyc03g113070.2.1	-1.98039	2.787978	7.10E-07	7.38E-05	52	156	127	652	741	338
Solyc05g050800.2.1	-1.9758	7.0618	2.68E-10	8.47E-08	1101	3094	2219	10572	17324	6719
Solyc12g057140.1.1	-1.97214	4.613221	2.03E-06	0.000178	119	853	348	2425	2771	1033
Solyc01g096510.2.1	-1.97022	5.687493	1.05E-05	0.000712	255	2027	508	4585	6264	2384
Solyc10g007880.2.1	-1.96953	4.032245	2.68E-05	0.001575	189	299	206	2081	961	832
Solyc05g008820.2.1	-1.95764	3.104052	2.92E-05	0.001675	57	256	125	876	1060	273
Solyc09g005090.1.1	-1.95333	3.60316	1.22E-06	0.000116	102	348	138	986	1420	667
Solyc12g036330.1.1	-1.95001	2.957361	7.41E-07	7.59E-05	60	189	134	696	883	377
Solyc03g111720.2.1	-1.94623	6.667869	1.19E-05	0.000791	1223	918	2145	11278	9042	4726
Solyc02g093950.2.1	-1.93829	1.964133	8.67E-05	0.003983	31	119	41	291	499	199
Solyc05g056170.2.1	-1.93796	6.811916	2.06E-10	7.12E-08	868	2545	2226	10207	12644	5557
Solyc04g009440.2.1	-1.92275	6.497037	1.65E-06	0.000152	944	1310	1885	7554	13413	2949
Solyc01g086730.2.1	-1.92106	4.494004	3.47E-07	3.96E-05	258	420	321	1917	2830	1019
Solyc03g118970.2.1	-1.91313	6.706916	9.79E-07	9.60E-05	1210	1077	2322	9403	11033	5625
Solyc09g047910.2.1	-1.91233	3.139269	1.03E-05	0.000705	72	163	204	857	1109	290
Solyc12g044940.1.1	-1.90939	5.847019	2.09E-08	3.65E-06	454	1561	918	4204	8321	2549
Solyc07g052790.1.1	-1.90891	5.431432	3.08E-05	0.001742	518	938	432	4460	5313	1308
Solyc01g086670.2.1	-1.9089	3.593795	1.04E-05	0.000709	143	164	224	803	1809	551
Solyc03g095770.2.1	-1.90766	6.635032	2.56E-06	0.000215	969	2862	924	8517	12152	4674
Solyc05g014390.2.1	-1.90683	5.922307	2.79E-05	0.001619	536	1716	745	5921	8513	1492
Solyc06g005480.2.1	-1.90182	3.893877	4.01E-07	4.48E-05	128	312	299	1174	2107	590
Solyc09g011560.2.1	-1.90047	5.45123	2.33E-07	2.83E-05	410	1186	535	3903	5423	1853
Solyc12g010410.1.1	-1.88906	2.842934	4.14E-05	0.002179	38	244	115	634	905	287
Solyc05g041910.2.1	-1.88614	7.490516	3.02E-08	4.87E-06	1281	4907	3372	16647	21632	7464
Solyc04g074000.2.1	-1.88555	9.067914	8.80E-08	1.20E-05	5501	13641	6455	40604	63650	31078
Solyc02g079570.2.1	-1.88393	5.857193	4.44E-08	6.80E-06	592	1066	1107	5799	6276	2440
Solyc01g091840.2.1	-1.88184	4.956407	2.10E-06	0.000182	310	820	369	3019	3673	1179
Solyc06g062420.2.1	-1.86332	3.495405	2.08E-05	0.001265	64	418	150	975	1307	543
Solyc08g076390.2.1	-1.86041	5.206335	5.43E-07	5.88E-05	313	927	665	4017	3940	1268
Solyc10g080010.1.1	-1.85584	4.697455	1.11E-06	0.000107	215	800	338	2416	3046	1076
Solyc03g033840.2.1	-1.84971	5.694322	6.24E-07	6.59E-05	580	1305	566	3948	6580	2621
Solyc03g006700.2.1	-1.84561	3.578677	2.73E-05	0.001598	75	407	183	898	1765	449
Solyc04g008100.1.1	-1.83001	4.712113	1.53E-05	0.000973	198	988	241	1579	3168	1790
Solyc12g042480.1.1	-1.82667	6.456892	1.61E-05	0.001012	1066	1570	1438	7512	12871	2576
Solyc06g061170.2.1	-1.82182	3.503222	6.78E-05	0.003242	131	255	154	1066	1452	363
Solyc12g088040.1.1	-1.82015	2.926494	0.000124	0.005402	60	252	96	524	1164	291
Solyc11g005630.1.1	-1.81628	6.513553	3.56E-06	0.000283	828	3015	943	5900	12177	5153
Solyc05g056500.1.1	-1.80939	2.994984	1.99E-05	0.001217	69	178	173	665	1097	277
Solyc03g112970.1.1	-1.80872	2.44893	5.59E-05	0.002758	62	114	88	508	594	237
Solyc10g007900.2.1	-1.80609	4.801888	4.95E-06	0.000377	228	907	376	2600	3433	1013
Solyc01g104690.2.1	-1.80314	5.305295	4.52E-05	0.002342	410	770	809	1962	7682	1208
Solyc02g093050.2.1	-1.79925	6.754901	6.24E-08	9.19E-06	1090	2343	2077	10103	12159	4623
Solyc09g082890.1.1	-1.79485	1.533228	0.000107	0.004767	22	79	55	251	289	153
Solyc09g011740.2.1	-1.7908	6.315601	1.02E-06	9.89E-05	824	2013	1221	6937	10378	2954
Solyc01g097270.2.1	-1.79031	8.132059	7.08E-05	0.003331	3986	4681	3689	20804	42662	9847
Solyc03g119590.1.1	-1.78972	4.423028	4.22E-05	0.002199	254	600	185	1846	2483	1002
Solyc05g050380.2.1	-1.7801	7.603615	6.85E-06	0.000498	2392	4634	2292	16384	24210	8393
Solyc09g010780.2.1	-1.77937	3.287837	1.39E-05	0.000906	89	277	154	973	974	426
Solyc02g081360.2.1	-1.77635	4.325984	8.06E-06	0.000575	272	380	269	1512	1560	1604
Solyc02g093310.2.1	-1.77597	3.210784	9.96E-06	0.000683	61	260	210	716	1257	378
Solyc03g119810.1.1	-1.76582	3.392078	3.67E-05	0.002001	98	217	244	1122	1123	337
Solyc09g072810.2.1	-1.76225	4.431567	2.89E-05	0.001669	237	211	643	1398	2972	1104
Solyc07g055710.2.1	-1.75956	5.103815	1.78E-05	0.001101	428	618	616	2441	5416	1139
Solyc02g032950.2.1	-1.75574	5.776611	2.94E-05	0.001681	643	1398	691	6129	5394	1853

Solyc05g016310.1.1	-1.75475	4.822566	5.28E-06	0.000397	214	943	459	2982	2861	1067
Solyc10g011810.2.1	-1.74739	6.174108	4.01E-08	6.31E-06	559	2107	1459	5448	9455	3314
Solyc01g100010.2.1	-1.74581	4.545067	2.08E-05	0.001263	237	732	268	2052	2886	905
Solyc08g080640.1.1	-1.74534	6.949763	0.000123	0.005384	1736	2930	1039	12212	11325	6146
Solyc02g080070.2.1	-1.74478	7.726405	2.79E-07	3.32E-05	2161	5517	3531	17084	26847	9283
Solyc05g014380.2.1	-1.74146	6.802273	3.99E-05	0.002127	1262	2981	1463	10554	14368	3330
Solyc02g014030.1.1	-1.73817	4.585571	9.94E-08	1.33E-05	207	674	459	1742	3160	1138
Solyc01g098140.2.1	-1.738	3.457717	4.20E-05	0.002195	51	421	212	816	1317	581
Solyc06g076560.1.1	-1.73328	2.816242	5.16E-05	0.002596	78	143	142	375	1057	362
Solyc04g048900.2.1	-1.73108	7.276449	3.56E-06	0.000283	2013	3129	2374	12734	19258	6768
Solyc01g111350.2.1	-1.73091	4.605593	0.00023	0.008983	106	870	570	3006	2047	764
Solyc08g078050.1.1	-1.72258	5.331661	4.39E-08	6.80E-06	415	805	933	3668	4336	1833
Solyc04g011770.2.1	-1.7216	2.928843	1.31E-05	0.00086	68	167	187	689	833	332
Solyc11g005700.1.1	-1.72051	3.587721	7.22E-06	0.000517	102	326	251	1209	1188	503
Solyc02g081350.2.1	-1.71996	2.102044	0.000136	0.005812	52	82	82	302	346	338
Solyc01g109710.2.1	-1.71803	6.459878	3.36E-05	0.001867	1259	1401	1442	7819	10941	3262
Solyc02g082450.2.1	-1.71718	4.738978	1.08E-05	0.000731	248	612	619	3025	2633	825
Solyc01g089890.2.1	-1.71211	4.444158	6.98E-06	0.000505	203	727	282	1644	2778	1009
Solyc02g090490.2.1	-1.70784	5.822385	2.95E-06	0.000242	575	1343	1156	4725	7876	1812
Solyc02g079170.2.1	-1.70549	7.078416	4.14E-05	0.002179	1916	2320	2157	14499	12610	5365
Solyc04g074710.2.1	-1.69789	2.371459	0.000247	0.009513	51	93	138	412	707	178
Solyc02g086590.2.1	-1.69626	5.108961	1.09E-06	0.000105	404	723	670	3011	4148	1384
Solyc07g008140.2.1	-1.69308	5.25997	3.23E-07	3.73E-05	208	1234	975	3237	3126	2579
Solyc08g081490.2.1	-1.69181	4.564312	8.65E-06	0.000609	253	672	354	2306	2545	913
Solyc12g008500.1.1	-1.68778	6.285641	2.99E-05	0.001701	1058	978	1785	7129	8924	3124
Solyc03g112980.1.1	-1.68321	2.24838	0.000182	0.007411	60	111	71	309	571	272
Solyc09g009130.2.1	-1.67796	5.046746	3.45E-06	0.000277	359	787	647	3655	2755	1383
Solyc11g007420.1.1	-1.67069	2.511389	3.77E-05	0.00204	55	155	110	501	548	298
Solyc09g064780.2.1	-1.66305	5.130757	2.51E-06	0.000212	431	840	564	2946	4150	1507
Solyc01g112220.2.1	-1.66254	4.405464	1.42E-06	0.000133	210	533	453	1781	2631	831
Solyc02g079750.2.1	-1.65998	5.716427	6.70E-05	0.003221	401	1675	1114	3024	9349	1551
Solyc04g056340.2.1	-1.65828	4.081188	4.18E-05	0.002193	205	391	298	1316	2384	574
Solyc12g008960.1.1	-1.65775	4.892014	2.40E-05	0.00143	223	1168	420	2566	3472	1240
Solyc04g077310.2.1	-1.65604	4.051723	0.000143	0.006046	188	418	293	1749	1856	451
Solyc07g043250.1.1	-1.65286	5.507337	2.16E-08	3.75E-06	406	1312	949	3626	5315	2176
Solyc04g074480.2.1	-1.65224	9.158566	8.33E-07	8.31E-05	6762	10729	13069	49287	63610	26110
Solyc02g093230.2.1	-1.65109	3.929276	0.000107	0.004751	140	534	212	1289	1965	534
Solyc03g117980.2.1	-1.64949	7.291422	4.82E-05	0.002465	1801	4287	2422	15892	17665	4832
Solyc02g085830.2.1	-1.63466	3.684399	0.000136	0.005812	161	220	299	1344	1261	461
Solyc03g078490.2.1	-1.63289	9.415741	2.59E-06	0.000216	7645	19113	11164	64250	67217	31529
Solyc08g014150.2.1	-1.62819	5.330675	8.99E-06	0.000628	509	892	735	3126	5460	1481
Solyc02g088090.1.1	-1.62798	7.043239	6.09E-06	0.000451	1067	4786	2228	12091	14138	5610
Solyc05g016060.2.1	-1.62696	5.30508	4.48E-05	0.002326	346	1568	463	3253	4651	1745
Solyc05g052750.2.1	-1.61987	4.614855	6.22E-08	9.19E-06	225	698	532	1916	2669	1297
Solyc02g067530.2.1	-1.61069	5.063088	4.27E-08	6.68E-06	292	900	831	2765	3645	1626
Solyc06g074530.1.1	-1.61009	5.845606	6.93E-07	7.24E-05	576	1764	1023	4404	7083	2605
Solyc02g093860.2.1	-1.60153	7.301753	8.05E-07	8.07E-05	1305	4559	3896	14788	16321	6658
Solyc07g049660.2.1	-1.59977	7.174604	1.43E-05	0.000925	1786	4010	2117	12163	16998	6026
Solyc10g012370.2.1	-1.59869	7.494071	3.40E-05	0.001883	2236	3541	4064	14863	24425	5762
Solyc05g015840.2.1	-1.58901	4.121274	3.59E-05	0.001971	209	502	260	1250	2289	748
Solyc09g075120.2.1	-1.58825	7.007959	0.000117	0.005146	1990	2156	2239	11827	14293	4960
Solyc08g077740.1.1	-1.58623	1.97615	0.000199	0.007986	42	98	86	323	362	222
Solyc02g093270.2.1	-1.58537	7.899808	7.83E-07	7.90E-05	2455	6192	5432	23324	20571	11727
Solyc04g009900.2.1	-1.57889	5.341595	9.28E-05	0.004226	349	1138	1029	4822	3596	1127
Solyc04g006940.2.1	-1.57309	5.317788	9.09E-06	0.000633	435	1235	659	4003	3622	1715
Solyc11g015900.1.1	-1.57027	3.661002	0.000104	0.004674	114	480	185	1102	1268	611
Solyc03g083440.2.1	-1.56724	6.244264	1.27E-05	0.000836	929	1864	1467	7065	8467	2742
Solyc09g075020.2.1	-1.56666	9.929299	1.20E-06	0.000114	11668	23140	20483	88755	88934	50018
Solyc02g091650.2.1	-1.56451	4.44461	1.51E-05	0.000965	230	657	403	2076	2250	850
Solyc07g008590.1.1	-1.56407	3.864714	3.63E-05	0.001987	186	377	245	1150	1692	660
Solyc01g005920.2.1	-1.56186	3.488472	4.92E-05	0.002511	91	278	339	886	1422	432
Solyc09g083060.2.1	-1.5524	5.308148	1.23E-07	1.61E-05	395	1121	892	3397	4034	1924
Solyc02g087740.2.1	-1.5498	2.574052	0.000123	0.005384	67	156	124	476	444	413
Solyc09g091660.2.1	-1.53826	9.373885	2.43E-06	0.000206	7573	17984	13509	53083	78243	28763
Solyc05g050360.2.1	-1.53718	5.992009	1.97E-05	0.001211	743	1983	999	4807	8167	2582
Solyc12g087920.1.1	-1.52481	4.744781	5.90E-07	6.33E-05	313	574	691	2017	2914	1409
Solyc09g090730.1.1	-1.5236	6.565731	9.90E-06	0.000681	1121	2751	1695	7712	11262	3869
Solyc12g040570.1.1	-1.51485	3.431398	1.89E-05	0.001164	104	339	232	646	1220	677
Solyc10g006710.2.1	-1.51063	4.521889	0.000145	0.006099	194	972	332	1666	2915	988
Solyc11g056620.1.1	-1.50991	4.788202	8.73E-06	0.000613	359	677	546	2518	2637	1273
Solyc02g062800.2.1	-1.50762	5.437121	3.18E-05	0.001781	554	1348	602	3913	3982	2131
Solyc01g006150.2.1	-1.50751	6.544912	8.64E-07	8.56E-05	774	3135	2221	7583	9907	4541
Solyc07g054830.2.1	-1.50454	5.866552	7.39E-06	0.000528	720	1543	1157	5095	6399	2368
Solyc06g082590.1.1	-1.50164	6.385495	0.000208	0.008253	1221	2118	1239	7370	9888	2872
Solyc06g050810.2.1	-1.50153	4.680721	5.97E-07	6.36E-05	240	785	612	1968	2830	1268
Solyc03g113040.2.1	-1.4983	3.764723	3.93E-05	0.002108	160	359	293	1155	1474	578
Solyc04g049090.2.1	-1.4955	8.178793	1.39E-05	0.000906	3591	7973	5508	22977	34933	11837
Solyc12g094650.1.1	-1.49279	6.536843	4.02E-05	0.002138	1204	2863	1326	6965	10892	4325
Solyc02g083460.2.1	-1.47936	6.709729	1.45E-05	0.000931	1378	2655	2058	9950	8816	5128
Solyc08g069060.2.1	-1.47356	6.508902	0.000164	0.006758	1321	1851	1927	9316	8639	3230
Solyc04g074030.2.1	-1.46825	6.462984	6.97E-05	0.003307	995	3337	1151	5383	10312	5176
Solyc03g098730.1.1	-1.4658	6.308583	5.95E-05	0.002891	670	3428	1233	4753	9570	4527
Solyc01g110960.2.1	-1.46193	2.49071	0.000175	0.007163	62	162	125	370	595	332

Solyc04g005160.1.1	-1.45941	8.113535	1.49E-05	0.00095	3201	8825	5130	22380	31295	11918
Solyc02g087580.2.1	-1.45816	2.925939	0.000164	0.006777	74	178	237	479	969	367
Solyc05g052210.2.1	-1.45601	4.721486	2.99E-06	0.000245	299	715	630	2084	2943	1187
Solyc02g089060.2.1	-1.4527	4.554902	2.74E-05	0.0016	147	903	623	1606	2808	1167
Solyc05g007150.2.1	-1.44494	5.708623	1.51E-07	1.94E-05	504	1424	1509	3028	6130	3159
Solyc06g063210.2.1	-1.4449	4.977386	2.68E-05	0.001575	410	943	536	2711	3095	1472
Solyc03g005470.2.1	-1.44485	6.95094	2.07E-06	0.000181	1276	3872	2856	9445	14113	5670
Solyc06g063180.1.1	-1.44479	5.349003	2.56E-06	0.000215	354	1329	1065	2940	5034	1772
Solyc10g038080.1.1	-1.44291	4.205227	3.63E-05	0.001987	113	602	605	1362	2074	898
Solyc03g111800.2.1	-1.44153	6.316887	3.14E-06	0.000254	974	2041	1881	5159	10120	3825
Solyc08g061250.2.1	-1.44091	3.462353	3.55E-05	0.001953	114	338	258	813	891	752
Solyc05g051570.2.1	-1.42831	4.88234	1.78E-05	0.001101	367	846	604	2391	3314	1229
Solyc09g011580.2.1	-1.42632	7.281525	3.15E-05	0.001777	1972	2561	4969	8496	16887	10638
Solyc08g078040.2.1	-1.42579	6.964782	2.89E-06	0.000238	1538	2563	3620	9582	12228	6881
Solyc02g086270.2.1	-1.40911	3.87264	0.000224	0.008798	110	603	323	1225	1549	625
Solyc08g078310.2.1	-1.40484	4.551565	6.35E-05	0.003068	310	601	524	1638	3061	922
Solyc03g118720.2.1	-1.4035	4.9374	8.43E-06	0.000596	320	801	901	2210	3801	1273
Solyc04g064530.1.1	-1.39854	6.277798	8.17E-05	0.003766	1079	2337	1267	5702	9216	3391
Solyc08g082820.2.1	-1.39632	8.878587	8.00E-05	0.003715	3582	14173	15884	18257	56479	35035
Solyc03g006080.2.1	-1.39458	4.419786	5.06E-05	0.002556	250	640	486	1522	2747	839
Solyc07g047800.2.1	-1.39258	8.634498	2.56E-05	0.001516	5565	9488	8917	29701	44957	18324
Solyc06g053710.2.1	-1.39248	7.135715	0.000212	0.008392	2207	2768	3081	13664	10593	6993
Solyc04g007580.1.1	-1.3887	5.631885	1.82E-06	0.000164	564	1666	1004	3592	5103	2693
Solyc08g065480.2.1	-1.38739	6.612251	1.60E-05	0.001005	1202	2687	2333	8570	9887	4093
Solyc07g043560.2.1	-1.37818	7.01266	6.94E-06	0.000503	1128	3877	4062	6359	13331	9673
Solyc10g055680.1.1	-1.37717	5.978098	0.000234	0.009106	463	2777	1354	5672	6464	2418
Solyc10g074570.1.1	-1.37238	5.746751	2.48E-05	0.00147	721	1532	1091	4594	5436	2304
Solyc10g012170.2.1	-1.37068	4.077359	5.95E-05	0.002891	183	500	442	1279	2013	672
Solyc05g052280.2.1	-1.35962	3.519045	0.000144	0.006099	124	380	265	848	1274	526
Solyc06g082080.2.1	-1.34988	3.544356	0.000137	0.005858	116	383	305	766	1411	547
Solyc06g050930.2.1	-1.34842	5.315798	2.86E-06	0.000236	461	1308	882	2982	3913	2134
Solyc10g045420.1.1	-1.34806	4.600789	5.36E-05	0.002663	276	805	541	2040	2571	974
Solyc01g087870.2.1	-1.34402	4.863069	1.80E-06	0.000163	281	886	867	1733	2992	1873
Solyc06g065560.1.1	-1.34376	5.95162	4.70E-05	0.002415	685	2050	1452	4941	7248	2293
Solyc09g090040.2.1	-1.33774	5.524651	0.000113	0.005002	604	1019	1330	3779	5421	1578
Solyc12g009550.1.1	-1.3283	3.622793	0.000164	0.006758	121	354	387	745	1552	587
Solyc06g007930.2.1	-1.32743	6.066642	1.11E-05	0.000744	682	2208	1795	5258	7368	2836
Solyc04g081510.2.1	-1.32306	5.455167	7.67E-05	0.003572	539	1194	1176	3942	4617	1521
Solyc09g009840.1.1	-1.32077	5.628444	6.98E-05	0.003307	600	1671	1034	4070	5520	1853
Solyc12g009220.1.1	-1.3157	7.310873	6.26E-05	0.003028	1895	5011	3790	11794	19254	6121
Solyc06g071280.2.1	-1.31469	6.423564	2.79E-05	0.001619	1110	2771	1757	6272	9307	4093
Solyc01g007990.2.1	-1.30139	5.277687	1.88E-06	0.000169	453	1139	1059	2584	4110	2112
Solyc01g079610.2.1	-1.29213	7.063138	1.01E-05	0.000694	1169	4388	4334	7376	14600	8383
Solyc04g081570.2.1	-1.29103	9.854057	3.65E-05	0.001992	8722	32168	26592	43373	99375	66035
Solyc05g013750.2.1	-1.2803	7.227686	8.99E-05	0.004118	1876	4229	4061	12248	16547	5608
Solyc09g089640.2.1	-1.27601	5.531298	6.94E-05	0.0033	627	1454	983	3929	4466	1984
Solyc02g077470.2.1	-1.27361	4.715043	4.11E-05	0.002176	311	730	776	2065	2792	1108
Solyc06g009140.2.1	-1.27138	8.943606	4.67E-05	0.002405	6796	12917	12754	40197	46230	23484
Solyc09g090070.1.1	-1.26817	7.2606	0.00013	0.005626	1964	5111	3344	12155	16753	6121
Solyc12g094660.1.1	-1.26662	6.625375	5.95E-05	0.002891	1341	2679	2550	7993	10042	4251
Solyc03g114600.2.1	-1.2597	4.852495	9.40E-05	0.004253	356	816	821	2501	2859	1132
Solyc02g067660.2.1	-1.25619	4.570479	0.000215	0.008488	291	826	531	1550	2999	973
Solyc02g080010.2.1	-1.2471	6.751567	0.00013	0.005626	822	4315	3268	7038	12679	5003
Solyc01g067070.2.1	-1.2451	5.088298	0.000144	0.006099	343	1058	1100	2635	3992	1202
Solyc08g081230.1.1	-1.23942	5.897153	6.74E-05	0.003231	572	2164	1721	3581	7579	2667
Solyc03g082920.2.1	-1.23363	9.259365	4.12E-05	0.002179	5847	20096	20141	30689	68049	39221
Solyc04g005550.1.1	-1.22178	4.579466	0.000116	0.00514	203	898	746	1798	2479	1079
Solyc03g034250.2.1	-1.21931	4.487929	0.000206	0.008192	268	620	713	1904	2215	882
Solyc04g011520.2.1	-1.21813	5.720622	5.44E-05	0.002697	708	1527	1403	4166	5463	2200
Solyc05g014980.2.1	-1.2154	6.098961	2.11E-05	0.001277	817	2145	1957	5266	6774	3201
Solyc08g066210.2.1	-1.20525	5.646234	7.05E-05	0.003324	619	1687	1274	3606	5746	2029
Solyc05g006510.1.1	-1.20459	4.690075	0.000231	0.009018	244	1026	704	1526	3246	1162
Solyc05g008310.2.1	-1.19526	4.276382	0.000141	0.005973	206	686	556	1437	2013	868
Solyc10g084400.1.1	-1.19508	7.161639	8.04E-05	0.003719	1502	4519	4745	9631	16858	6111
Solyc03g118040.2.1	-1.19346	9.88685	4.78E-05	0.002451	9197	30681	32724	51434	101827	57627
Solyc10g080770.1.1	-1.18355	7.099958	0.000127	0.005512	1806	4169	3769	10950	13842	5701
Solyc09g075450.2.1	-1.18351	6.742211	0.000123	0.005384	1405	3072	3131	7303	12461	4517
Solyc09g007910.2.1	-1.17979	6.960264	5.88E-05	0.002877	1145	3758	4822	8935	11696	6621
Solyc02g064680.2.1	-1.17411	7.400055	0.000118	0.005181	1867	5805	5032	14786	13007	8346
Solyc02g087330.2.1	-1.15924	5.564918	6.60E-05	0.003181	505	1674	1441	3257	5392	1999
Solyc01g104050.2.1	-1.15744	5.384127	0.000228	0.008921	605	1160	1152	3330	4212	1689
Solyc05g053930.2.1	-1.15673	5.774164	5.93E-05	0.002891	676	1570	1781	3674	6157	2437
Solyc11g017270.1.1	-1.14924	6.765545	0.000182	0.007411	1467	3369	3001	8699	10582	4616
Solyc08g079420.2.1	-1.14797	6.325459	0.000232	0.009018	1080	2014	2680	5694	8926	3330
Solyc01g100200.2.1	-1.14491	6.314436	9.29E-05	0.004226	1038	2743	2033	5709	8116	3714
Solyc06g009110.2.1	-1.13672	5.129771	0.000206	0.008194	497	1091	910	2661	3487	1533
Solyc06g073990.1.1	-1.12462	5.021323	5.04E-05	0.002556	395	955	1096	2214	2716	1968
Solyc04g079760.2.1	-1.12031	7.857918	8.27E-05	0.003807	2539	7004	8501	16416	21096	12465
Solyc10g051020.1.1	-1.1147	7.730382	0.000146	0.006153	2248	8397	6097	16031	17518	11594
Solyc03g115070.1.1	-1.08009	4.700482	0.00017	0.006991	322	845	835	1817	2556	1246
Solyc08g080680.2.1	-1.07571	5.035458	0.000219	0.008629	396	1152	1004	2131	3606	1472
Solyc12g099100.1.1	-1.05953	6.380858	0.000133	0.00573	972	2968	2675	6055	7628	4101
Solyc01g090430.2.1	-1.05497	5.220277	0.000214	0.008481	529	1167	1104	2606	3585	1803

Solyc03g007170.2.1	-1.03205	5.799557	0.000127	0.005513	711	1785	1885	3739	5304	2825
Solyc06g054010.2.1	1.00571	5.541305	0.000246	0.009508	1122	3382	3100	1373	1748	1614
Solyc11g069190.1.1	1.007456	5.896831	0.000201	0.008028	1331	4248	4338	1907	2696	1633
Solyc03g007370.2.1	1.048528	5.596316	0.000171	0.006999	1372	2797	3456	1330	1948	1598
Solyc04g077340.2.1	1.11929	4.540935	0.000131	0.005666	485	1795	1773	740	961	599
Solyc09g082980.2.1	1.12179	5.656813	2.21E-05	0.00133	1184	3809	3575	1382	2102	1484
Solyc06g073180.2.1	1.126168	7.367213	0.000106	0.004733	3907	11075	13024	4776	5787	5297
Solyc07g056240.2.1	1.144069	4.447126	0.000157	0.00651	540	1443	1687	581	791	712
Solyc02g082760.2.1	1.167022	8.419747	0.000146	0.006126	9373	18847	28211	9022	12316	11057
Solyc09g011010.2.1	1.17246	5.283084	0.000224	0.008798	763	2696	3499	782	1774	1231
Solyc06g075660.2.1	1.183053	4.882182	0.000251	0.00966	487	2613	2349	839	1042	889
Solyc03g082960.2.1	1.198063	6.040137	0.000187	0.007595	1615	4238	5415	1352	2315	2425
Solyc08g005560.2.1	1.233751	5.415953	8.03E-05	0.003719	1174	2450	3528	803	2048	1235
Solyc02g087060.2.1	1.237451	3.99613	0.000226	0.008876	388	1025	1348	494	461	477
Solyc07g032380.2.1	1.288015	5.470253	7.32E-05	0.003428	819	3820	3612	872	1622	1481
Solyc10g007110.2.1	1.329553	3.697684	0.000127	0.005512	399	972	786	269	512	376
Solyc05g013380.2.1	1.33709	8.172046	4.19E-05	0.002193	8050	15480	25884	6060	10042	9101
Solyc08g075120.2.1	1.33742	3.76131	0.000137	0.005861	261	973	1290	317	563	339
Solyc03g098320.2.1	1.369254	5.833457	1.71E-05	0.001073	1089	4750	4913	1058	2423	1581
Solyc12g040790.1.1	1.418508	6.588936	3.92E-05	0.002108	1499	9234	8243	1956	3551	2691
Solyc11g012850.1.1	1.442782	6.16101	1.06E-06	0.000103	1667	5898	5626	1526	2300	2090
Solyc05g011890.1.1	1.443192	7.823038	1.44E-05	0.000928	4382	17199	21724	7451	6272	4878
Solyc08g075490.2.1	1.451398	5.537502	3.16E-05	0.001777	1659	2786	3108	680	1763	1451
Solyc05g055840.2.1	1.469618	3.511831	0.00025	0.009624	243	716	1184	225	310	374
Solyc03g120420.2.1	1.504012	4.296646	0.000148	0.006211	764	1022	1342	523	380	598
Solyc05g005960.2.1	1.504922	6.06359	3.24E-05	0.001814	1327	5183	6388	1041	1982	2265
Solyc01g107460.2.1	1.552098	7.050857	0.000105	0.004721	1926	8948	16168	3349	3039	3680
Solyc05g009080.2.1	1.571707	4.384785	8.13E-06	0.000579	478	1402	2084	340	660	620
Solyc11g072110.1.1	1.59079	3.906978	0.00014	0.005973	329	861	1687	266	346	497
Solyc12g099930.1.1	1.594774	8.580382	5.78E-05	0.002836	8956	25559	38273	5717	8713	13695
Solyc06g072710.2.1	1.608997	5.789111	0.000242	0.009349	1061	3704	6180	594	1481	2020
Solyc08g065220.2.1	1.61024	8.887752	3.07E-05	0.001742	13114	27844	45771	7145	11199	16376
Solyc02g087840.2.1	1.630282	2.100895	0.00014	0.005965	127	314	330	121	96	97
Solyc03g112760.2.1	1.663908	5.843272	0.00012	0.005289	1016	4066	6559	546	2060	1726
Solyc04g054740.2.1	1.688605	9.516011	2.82E-05	0.001633	27568	41235	54653	17124	11110	21877
Solyc08g082590.2.1	1.707097	5.496629	1.96E-06	0.000174	737	3370	5230	888	1173	1164
Solyc02g037550.2.1	1.734469	5.653429	0.000188	0.00762	1468	3360	4502	2186	851	461
Solyc09g011080.2.1	1.757379	4.882802	0.000226	0.008877	776	932	3911	839	474	665
Solyc02g072540.2.1	1.796279	5.201799	8.54E-06	0.000602	911	2707	3574	441	755	1230
Solyc10g083940.1.1	1.921586	1.84771	9.19E-05	0.004192	98	319	283	93	122	29
Solyc03g123580.2.1	1.979813	1.266283	0.0002	0.008015	76	220	158	29	47	68
Solyc02g063030.2.1	2.013739	3.624256	3.38E-06	0.000272	183	924	1601	191	329	244
Solyc03g025710.2.1	2.13995	4.212043	0.000239	0.009242	218	1543	2494	222	169	569
Solyc01g102610.2.1	2.141276	6.227184	2.80E-08	4.54E-06	1357	5313	9731	1103	1467	1618
Solyc04g080040.2.1	2.306184	4.695605	9.06E-09	1.72E-06	783	1876	2595	366	292	601
Solyc10g084430.1.1	3.06431	0.961693	7.14E-07	7.40E-05	34	144	285	16	22	24
Solyc02g078150.2.1	3.63357	2.683671	9.23E-06	0.00064	559	183	131	26	101	36
Solyc05g007080.2.1	4.529816	-1.54383	1.13E-05	0.000758	14	23	35	0	3	1
RRNA_11_20	7.863301	6.037138	1.59E-05	0.001	7248	56	43	58	7	15
Solyc11g027710.1.1	13.70892	4.158088	4.43E-05	0.0023	2002	0	0	0	0	0

Supplementary Table S4

[illegible]

Supplementary Table S5: GO enrichement analysis. *S. lycorpersicum* leaf tissue during *P. infestans* invasion at 24 hpi. Gene to GO BP Conditional test for over-representation

GOBPID	Pvalue	OddsRatio	ExpCount	Count	Size	Term
GO:0019605	0.000	804.489	0	2	7	butyrate metabolic process
GO:0046916	0.002	35.384	0	2	115	cellular transition metal ion homeostasis
GO:0051707	0.002	9.532	1	4	1028	response to other organism
GO:0071395	0.003	31.711	0	2	128	cellular response to jasmonic acid stimulus
GO:0009607	0.003	8.609	1	4	1131	response to biotic stimulus
GO:0007568	0.004	25.799	0	2	175	aging
GO:0030003	0.005	23.035	0	2	175	cellular cation homeostasis
GO:0080187	0.005	258.557	0	1	8	floral organ senescence
GO:0055065	0.006	19.795	0	2	203	metal ion homeostasis
GO:0006955	0.007	9.881	0	3	665	immune response
GO:1990169	0.007	180.960	0	1	11	stress response to copper ion
GO:0050832	0.007	19.742	0	2	228	defense response to fungus
GO:0009751	0.007	18.847	0	2	213	response to salicylic acid
GO:1900056	0.008	150.783	0	1	13	negative regulation of leaf senescence
GO:0009605	0.009	6.569	1	4	1453	response to external stimulus
GO:0055082	0.009	16.613	0	2	241	cellular chemical homeostasis
GO:0010623	0.009	129.229	0	1	15	developmental programmed cell death
GO:0071407	0.009	16.134	0	2	248	cellular response to organic cyclic compound
GO:0030001	0.010	15.808	0	2	253	metal ion transport
GO:0009750	0.012	95.195	0	1	20	response to fructose
GO:0009626	0.012	14.096	0	2	283	plant-type hypersensitive response
GO:0050801	0.014	13.057	0	2	305	ion homeostasis
GO:0009862	0.014	78.622	0	1	24	systemic acquired resistance, salicylic acid mediated signaling pathway
GO:0009864	0.016	69.538	0	1	27	induced systemic resistance, jasmonic acid mediated signaling pathway
GO:0006952	0.017	5.248	1	4	1782	defense response
GO:0009749	0.020	54.767	0	1	34	response to glucose
GO:0009817	0.020	54.767	0	1	34	defense response to fungus, incompatible interaction
GO:0044700	0.022	4.854	1	4	1911	single organism signaling
GO:0006631	0.023	10.016	0	2	395	fatty acid metabolic process
GO:0009411	0.025	45.424	0	1	45	response to UV
GO:0002237	0.028	39.261	0	1	47	response to molecule of bacterial origin
GO:0009863	0.029	38.528	0	1	53	salicylic acid mediated signaling pathway
GO:0051716	0.029	3.955	2	5	3156	cellular response to stimulus
GO:0034284	0.029	37.621	0	1	49	response to monosaccharide
GO:2000024	0.034	32.232	0	1	57	regulation of leaf development

GO:0008219	0.035	7.939	0	2	495	cell death
GO:0007154	0.036	4.123	1	4	2208	cell communication
GO:0048827	0.038	7.515	0	2	522	phyllome development
GO:0009744	0.039	28.191	0	1	65	response to sucrose
GO:0010224	0.045	24.368	0	1	75	response to UV-B
GO:0009835	0.048	22.532	0	1	81	fruit ripening

Gene to GO CC Conditional test for over-representation

GOCCID	Pvalue	OddsRatio	ExpCount	Count	Size	Term
GO:0010282	0.006	198.587	0	1	9	senescence-associated vacuole
GO:0005777	0.011	14.063	0	2	244	peroxisome
GO:0005741	0.044	24.374	0	1	66	mitochondrial outer membrane

Gene to GO MF Conditional test for over-representation

GOMFID	Pvalue	OddsRatio	ExpCount	Count	Size	Term
GO:0047760	0.000	747.000	0	2	6	butyrate-CoA ligase activity
GO:0016877	0.001	63.434	0	2	49	ligase activity, forming carbon-sulfur bonds
GO:0005509	0.020	10.482	0	2	283	calcium ion binding
GO:0004197	0.036	30.093	0	1	47	cysteine-type endopeptidase activity

Supplementary Table S6: GO enrichement analysis. *S. lycorpersicum* leaf tissue during *B. cinerea* invasion at 24 hpi. Gene to GO BP Conditional test for over-representation

GOBPID	Pvalue	OddsRatio	ExpCount	Count	Size	Term
GO:0015783	0.002	754.250	0	1	3	GDP-fucose transport
GO:0015786	0.004	377.083	0	1	5	UDP-glucose transport
GO:0072334	0.004	377.083	0	1	5	UDP-galactose transmembrane transport
GO:0009833	0.004	301.650	0	1	6	plant-type primary cell wall biogenesis
GO:1901679	0.004	301.650	0	1	6	nucleotide transmembrane transport
GO:0015931	0.006	20.918	0	2	158	nucleobase-containing compound transport
GO:0072531	0.006	215.440	0	1	8	pyrimidine-containing compound transmembrane transport
GO:0010274	0.011	100.494	0	1	16	hydrotropism
GO:0015780	0.016	71.758	0	1	22	nucleotide-sugar transport
GO:0071669	0.016	12.009	0	2	272	plant-type cell wall organization or biogenesis
GO:1901000	0.018	62.778	0	1	25	regulation of response to salt stress
GO:2000070	0.019	60.263	0	1	26	regulation of response to water deprivation
GO:0033609	0.021	53.798	0	1	29	oxalate metabolic process
GO:0006383	0.021	53.798	0	1	29	transcription from RNA polymerase III promoter
GO:0006284	0.028	38.600	0	1	40	base-excision repair
GO:0009827	0.029	37.633	0	1	41	plant-type cell wall modification
GO:0015748	0.033	32.714	0	1	47	organophosphate ester transport
GO:0048653	0.034	32.016	0	1	48	anther development
GO:0031408	0.041	26.385	0	1	58	oxylipin biosynthetic process
GO:0045489	0.042	25.928	0	1	59	pectin biosynthetic process
GO:0030244	0.048	22.434	0	1	68	cellulose biosynthetic process

Gene to GO BP Conditional test for under-representation

GOBPID	Pvalue	OddsRatio	ExpCount	Count	Size	Term
GO:0050794	0.017	0.000	4	0	4892	regulation of cellular process
GO:0065007	0.037	0.165	4	1	6083	biological regulation

Gene to GO CC Conditional test for over-representation

GOCCID	Pvalue	OddsRatio	ExpCount	Count	Size	Term
GO:0032541	0.010	113.445	0	1	15	cortical endoplasmic reticulum
GO:0005793	0.012	93.412	0	1	18	endoplasmic reticulum-Golgi intermediate compartment
GO:0005666	0.016	69.023	0	1	24	DNA-directed RNA polymerase III complex
GO:0098827	0.017	63.495	0	1	26	endoplasmic reticulum subcompartment
GO:0044425	0.028	3.228	5	9	7406	membrane part
GO:0016021	0.048	2.800	5	8	6673	integral component of membrane

Gene to GO MF Conditional test for over-representation

GOMFID	Pvalue	OddsRatio	ExpCount	Count	Size	Term

GO:0015605	0.000	78.805	0	2	43	organophosphate ester transmembrane transporter activity
GO:0052821	0.002	747.115	0	1	3	DNA-7-methyladenine glycosylase activity
GO:0052822	0.002	747.115	0	1	3	DNA-3-methylguanine glycosylase activity
GO:0043916	0.002	747.115	0	1	3	DNA-7-methylguanine glycosylase activity
GO:0005457	0.002	747.115	0	1	3	GDP-fucose transmembrane transporter activity
GO:0005460	0.004	373.519	0	1	5	UDP-glucose transmembrane transporter activity
GO:0005459	0.004	373.519	0	1	5	UDP-galactose transmembrane transporter activity
GO:0016207	0.004	298.800	0	1	6	4-coumarate-CoA ligase activity
GO:0008725	0.008	149.362	0	1	11	DNA-3-methyladenine glycosylase activity
GO:0005347	0.010	114.876	0	1	14	ATP transmembrane transporter activity
GO:0015932	0.013	89.727	0	1	19	nucleobase-containing compound transmembrane transporter activity
GO:0015216	0.013	87.828	0	1	18	purine nucleotide transmembrane transporter activity
GO:0005338	0.013	87.828	0	1	18	nucleotide-sugar transmembrane transporter activity
GO:0008509	0.016	11.960	0	2	269	anion transmembrane transporter activity
GO:0019104	0.019	57.399	0	1	27	DNA N-glycosylase activity
GO:0046564	0.019	57.399	0	1	27	oxalate decarboxylase activity
GO:0005337	0.021	53.294	0	1	29	nucleoside transmembrane transporter activity
GO:0016877	0.035	31.056	0	1	49	ligase activity, forming carbon-sulfur bonds
GO:0030145	0.039	27.597	0	1	55	manganese ion binding
GO:0022892	0.041	4.570	1	3	1097	substrate-specific transporter activity
GO:0016702	0.042	25.252	0	1	60	oxidoreductase activity, acting on single donors with incorporation of molecular oxygen, incorporation of two atoms of oxygen

Supplementary Table S7: GO enrichement analysis. *S. lycorpersicum* leaf tissue during *B. cinerea* invasion at 24 hpi. Down regulated genes. Gene to GO BP Conditional test for over-representation

GOBPID	Pvalue	OddsRatio	ExpCount	Count	Size	Term
GO:0009733	0.000	20.764	0	5	534	response to auxin
GO:0009734	0.001	18.582	0	3	322	auxin-activated signaling pathway
GO:0006811	0.002	10.356	1	4	749	ion transport
GO:0006865	0.002	39.960	0	2	84	amino acid transport
GO:0010930	0.002	754.250	0	1	3	negative regulation of auxin mediated signaling pathway
GO:0080143	0.003	502.806	0	1	4	regulation of amino acid export
GO:0032890	0.005	251.361	0	1	7	regulation of organic acid transport
GO:0051952	0.005	251.361	0	1	7	regulation of amine transport
GO:0015711	0.006	20.137	0	2	164	organic anion transport
GO:0032870	0.006	6.951	1	4	1092	cellular response to hormone stimulus
GO:0009719	0.006	5.665	1	5	1804	response to endogenous stimulus
GO:0001736	0.006	188.500	0	1	9	establishment of planar polarity
GO:0007165	0.008	5.397	1	5	1884	signal transduction
GO:0023052	0.008	5.305	1	5	1913	signaling
GO:0065007	0.009	4.456	4	9	6083	biological regulation
GO:0048829	0.010	115.968	0	1	14	root cap development
GO:0010492	0.011	100.494	0	1	16	maintenance of shoot apical meristem identity
GO:0009635	0.013	88.662	0	1	18	response to herbicide
GO:0010033	0.014	4.597	2	5	2172	response to organic substance
GO:0002009	0.016	68.492	0	1	23	morphogenesis of an epithelium
GO:0010167	0.016	68.492	0	1	23	response to nitrate
GO:0009641	0.017	65.511	0	1	24	shade avoidance
GO:0070887	0.018	4.989	1	4	1485	cellular response to chemical stimulus
GO:0009958	0.022	50.206	0	1	31	positive gravitropism
GO:0044070	0.027	40.691	0	1	38	regulation of anion transport
GO:0010311	0.028	38.600	0	1	40	lateral root formation
GO:0006857	0.029	37.633	0	1	41	oligopeptide transport
GO:0015706	0.030	35.837	0	1	43	nitrate transport
GO:0042128	0.035	30.706	0	1	50	nitrate assimilation
GO:0022622	0.036	7.655	0	2	422	root system development
GO:0019827	0.039	27.855	0	1	55	stem cell population maintenance
GO:0010286	0.039	27.347	0	1	56	heat acclimation
GO:0042886	0.040	26.857	0	1	57	amide transport
GO:0010218	0.042	25.928	0	1	59	response to far red light
GO:2001057	0.043	25.061	0	1	61	reactive nitrogen species metabolic process
GO:0009629	0.043	25.061	0	1	61	response to gravity

GO:0048513	0.044	4.489	1	3	1137	organ development
GO:0048519	0.048	4.342	1	3	1173	negative regulation of biological process
GO:0010101	0.048	22.434	0	1	68	post-embryonic root morphogenesis
GO:0048765	0.049	21.781	0	1	70	root hair cell differentiation
GO:1902578	0.049	3.517	1	4	2035	single-organism localization
GO:0010054	0.050	21.469	0	1	71	trichoblast differentiation

Gene to GO CC Conditional test for over-representation

GOCCID	Pvalue	OddsRatio	ExpCount	Count	Size	Term
GO:0009986	0.022	50.559	0	1	35	cell surface

Gene to GO MF Conditional test for over-representation

GOMFID	Pvalue	OddsRatio	ExpCount	Count	Size	Term
GO:0008251	0.002	747.115	0	1	3	tRNA-specific adenosine deaminase activity
GO:0015293	0.005	21.135	0	2	154	symporter activity
GO:0010328	0.006	213.407	0	1	8	auxin influx transmembrane transporter activity
GO:0010011	0.011	106.665	0	1	15	auxin binding
GO:0019239	0.012	93.322	0	1	17	deaminase activity
GO:0016814	0.016	67.850	0	1	23	hydrolase activity, acting on carbon-nitrogen (but not peptide) bonds, in cyclic amidines
GO:0015112	0.031	34.676	0	1	44	nitrate transmembrane transporter activity
GO:0016799	0.042	25.688	0	1	59	hydrolase activity, hydrolyzing N-glycosyl compounds
GO:0016597	0.043	24.829	0	1	61	amino acid binding
GO:0018024	0.046	23.273	0	1	65	histone-lysine N-methyltransferase activity

Gene to GO MF Conditional test for under-representation

GOMFID	Pvalue	OddsRatio	ExpCount	Count	Size	Term
GO:0003824	0.022	0.281	8	4	11423	catalytic activity
GO:0000166	0.023	0.000	3	0	4596	nucleotide binding
GO:0001882	0.045	0.000	3	0	3862	nucleoside binding
GO:0032555	0.045	0.000	3	0	3861	purine ribonucleotide binding
GO:0032550	0.046	0.000	3	0	3839	purine ribonucleoside binding
GO:0035639	0.047	0.000	3	0	3818	purine ribonucleoside triphosphate binding
