# Supplementary material for: Pathogen recognition in compatible plant-microbe interactions
Source: Sci Rep. 2017 Jul 25;7:6383. doi: 10.1038/s41598-017-04792-5 (PMC5526865; doi:10.1038/s41598-017-04792-5)
Supplement: Supplementary file 2 — Supplementary Data 1 [file 41598_2017_4792_MOESM2_ESM.pdf]

Supplementary Data 1
Pathogen recognition in compatible plant-microbe interactions

Fabio Rezzonico[1], Oliver Rupp[2], Johannes Fahrentrapp[3]*

[1] Research group Environmental Genomics and Systems Biology, Zurich University of Applied Sciences, Wädenswil, Switzerland

[2] Bioinformatics and Systems Biology, University of Giessen, Giessen, Germany

[3] Research Group for Viticulture, Zurich University of Applied Sciences, Wädenswil, Switzerland

* Corresponding author: johannes.fahrentrapp@zhaw.ch

Abstract
Microbial infections in plant leaves remain a major challenge in agriculture. Hence an understanding of disease mechanisms at the molecular level is of paramount importance for identifying possible intervention points for their control. Whole-transcriptome changes during early disease stages in susceptible plant species are less well-documented than those of resistant ones. This study focuses on the differential transcriptional changes at 24 hours post inoculation (hpi) in tomato leaflets affected by three pathogens: (1) Phytophthora infestans, (2) Botrytis cinerea, and (3) Oidium neolycopersici. Grey mould (B. cinerea) was the disease that had progressed the most by 24 hpi, both in terms of visible symptoms as well as differential gene expression. By means of RNA-seq, we identified 50 differentially expressed tomato genes induced by B. cinerea infection and 18 by P. infestans infection at 24 hpi. Additionally, a set of 63 genes were differentially expressed during all three diseases when compared by a Bayesian approach to their respective mock infections. And Gene expression patterns were found to also depend on the inoculation technique. These findings suggest a specific and distinct transcriptional response in plant leaf tissue in reaction to B. cinerea and P. infestans invasion at 24 hpi, indicating that plants may recognize the attacking pathogen.

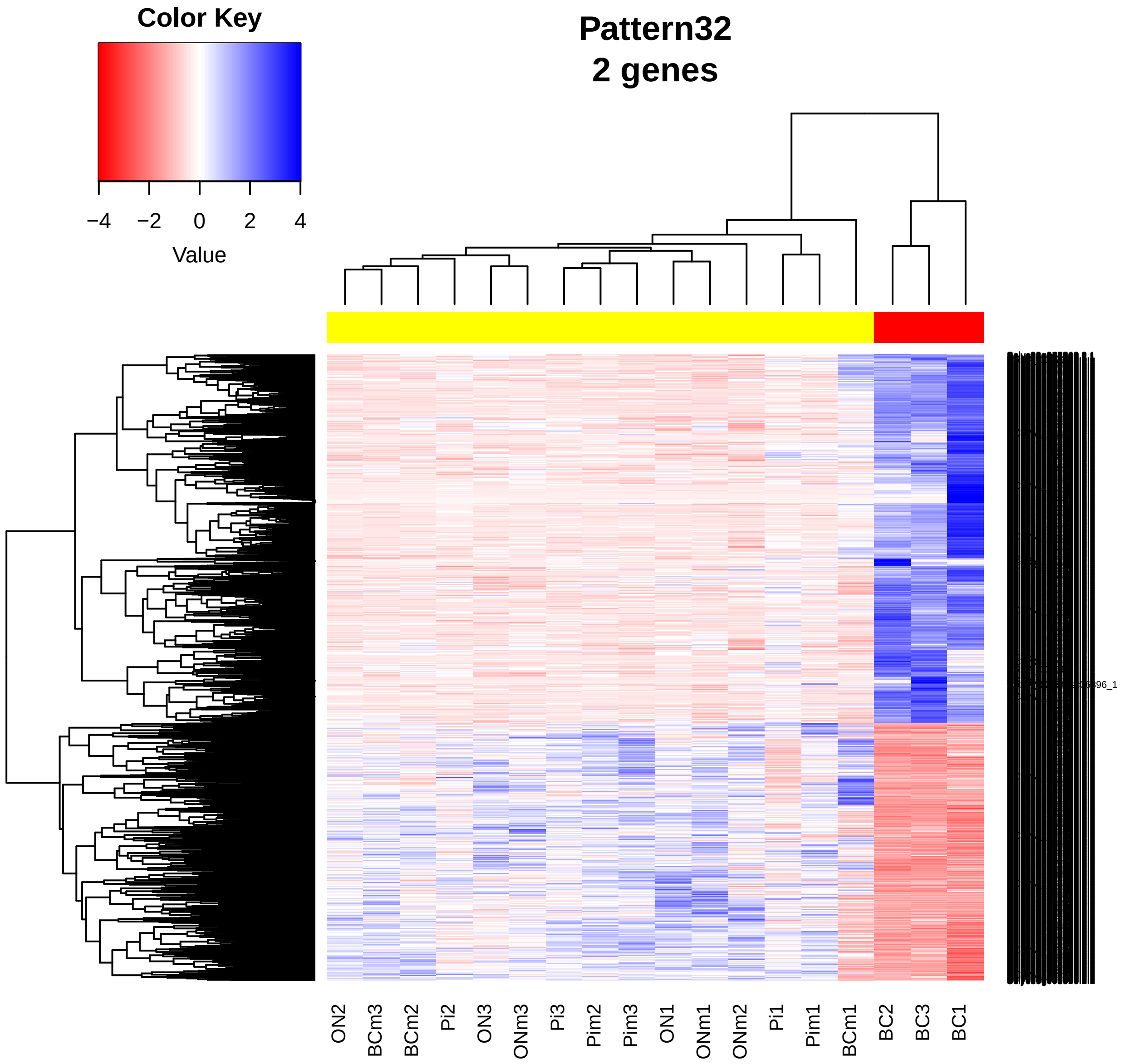
Color Key
−4
−2
0
2
4
Value
Pattern32
2 genes
ON2
BCm3
BCm2
Pi2
ON3
ONm3
Pi3
Pim2
Pim3
ON1
ONm1
ONm2
Pi1
Pim1
BCm1
BC2
BC3
BC1

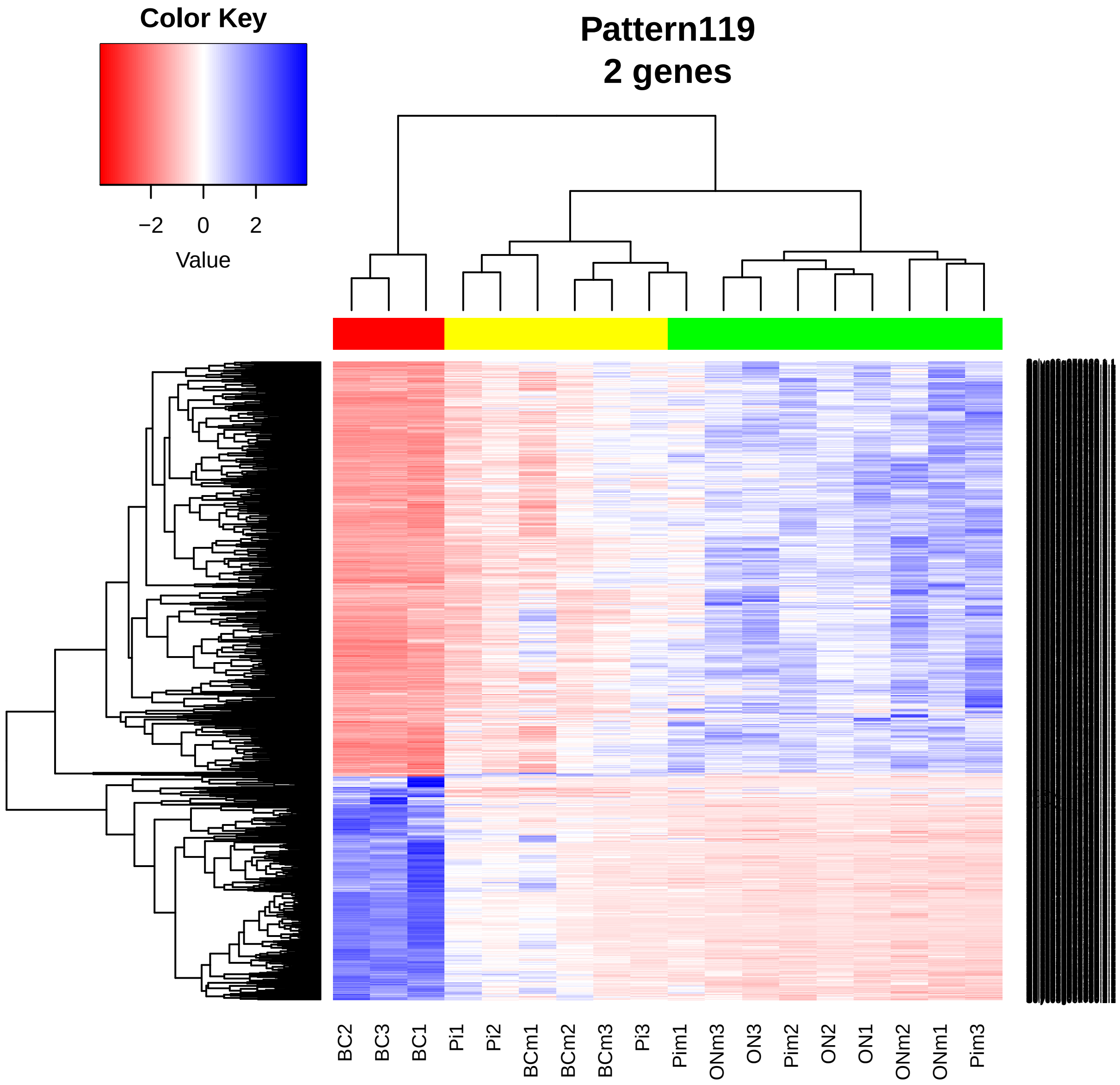
Color Key
−2
0
2
Value
Pattern119
2 genes
BC2
BC3
BC1
Pi1
Pi2
BCm1
BCm2
BCm3
Pi3
Pim1
ONm3
ON3
Pim2
ON2
ON1
ONm2
ONm1
Pim3

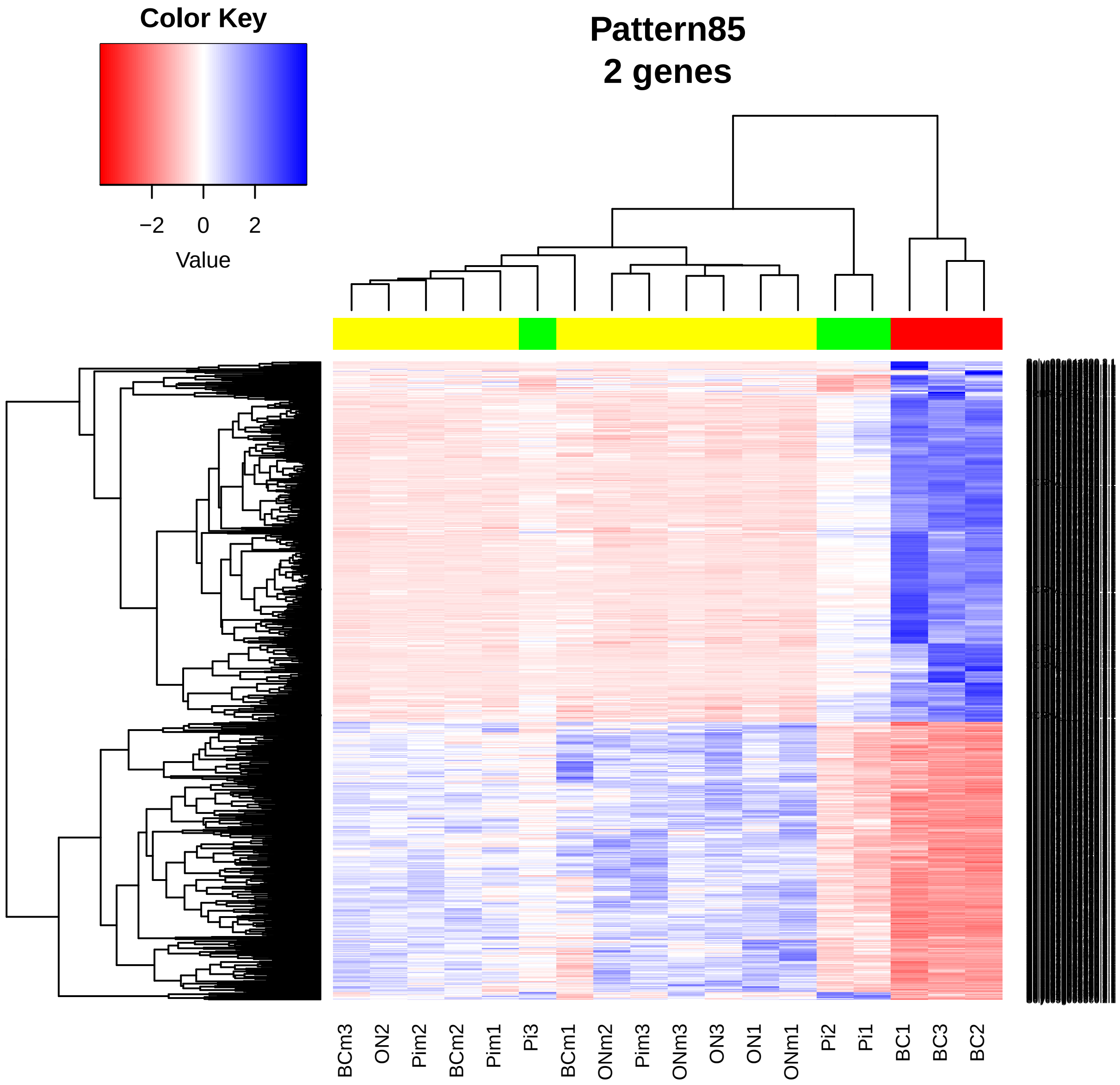

Color Key
−2
0
2
Value
Pattern85
2 genes
BCm3
ON2
Pim2
BCm2
Pim1
Pi3
BCm1
ONm2
Pim3
ONm3
ON3
ON1
ONm1
Pi2
Pi1
BC1
BC3
BC2

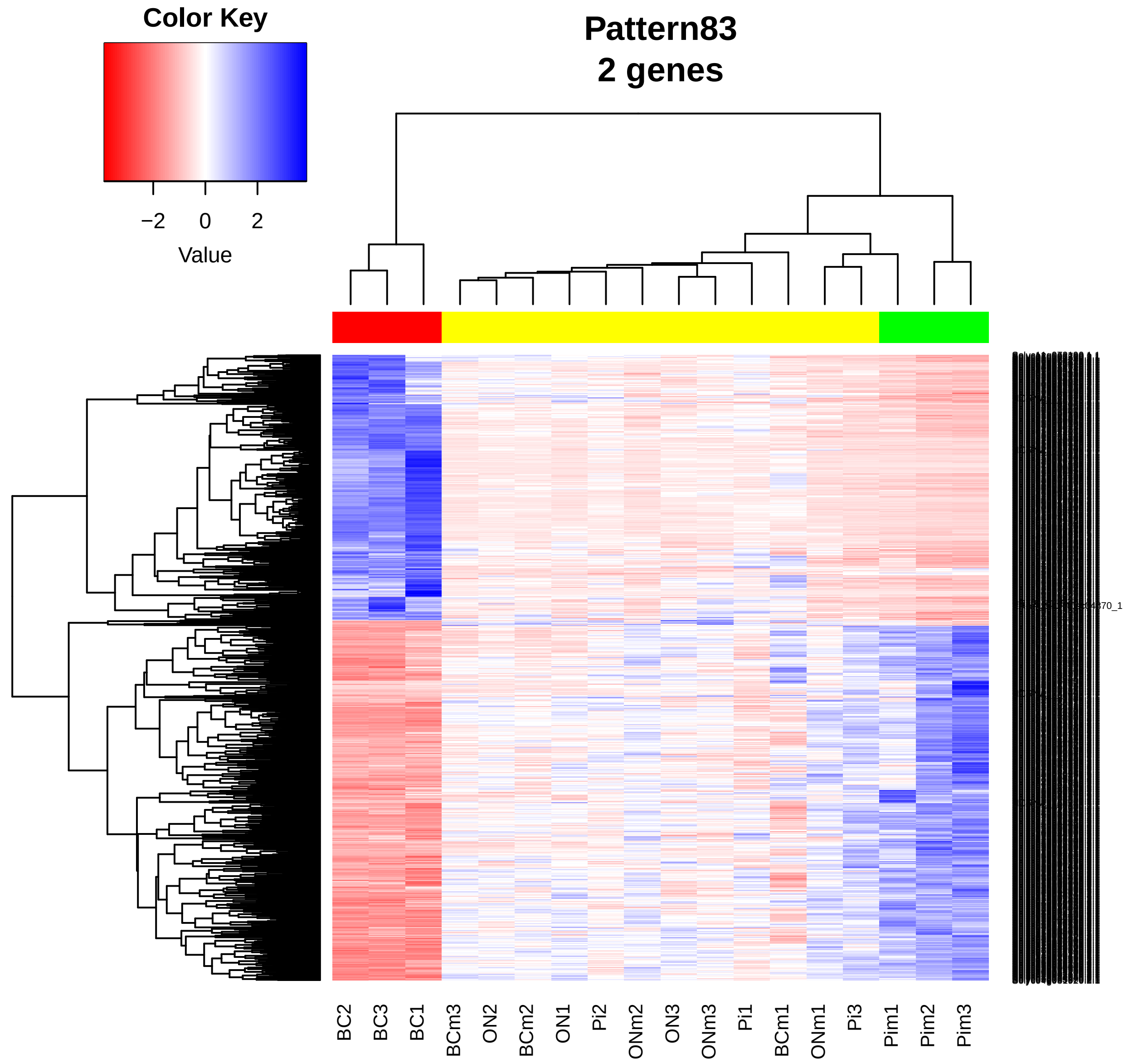

Color Key
−2
0
2
Value
Pattern83
2 genes
BC2
BC3
BC1
BCm3
ON2
BCm2
ON1
Pi2
ONm2
ON3
ONm3
Pi1
BCm1
ONm1
Pi3
Pim1
Pim2
Pim3

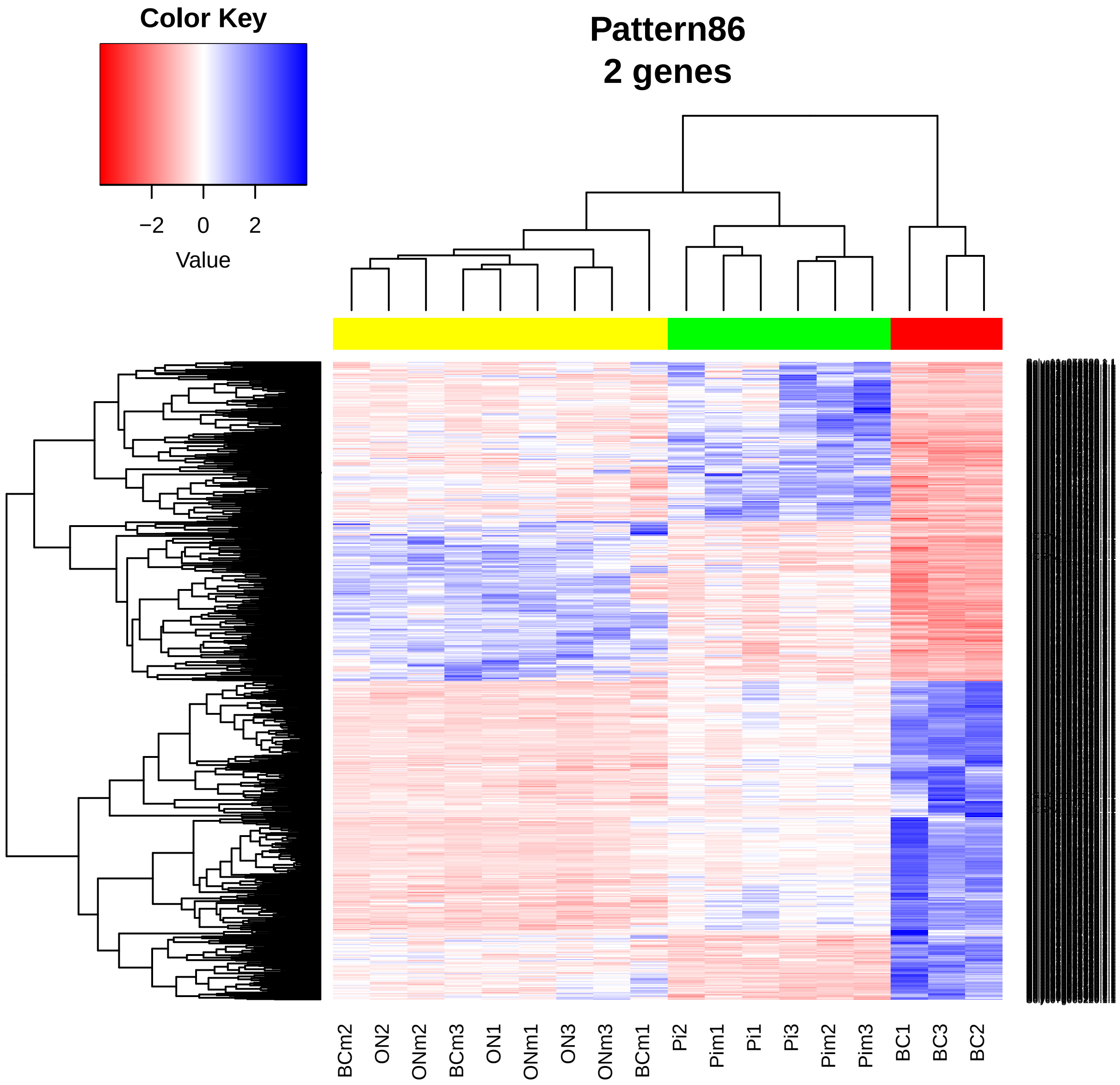

Color Key
−2
0
2
Value
Pattern86
2 genes
BCm2
ON2
ONm2
BCm3
ON1
ONm1
ON3
ONm3
BCm1
Pi2
Pim1
Pi1
Pi3
Pim2
Pim3
BC1
BC3
BC2

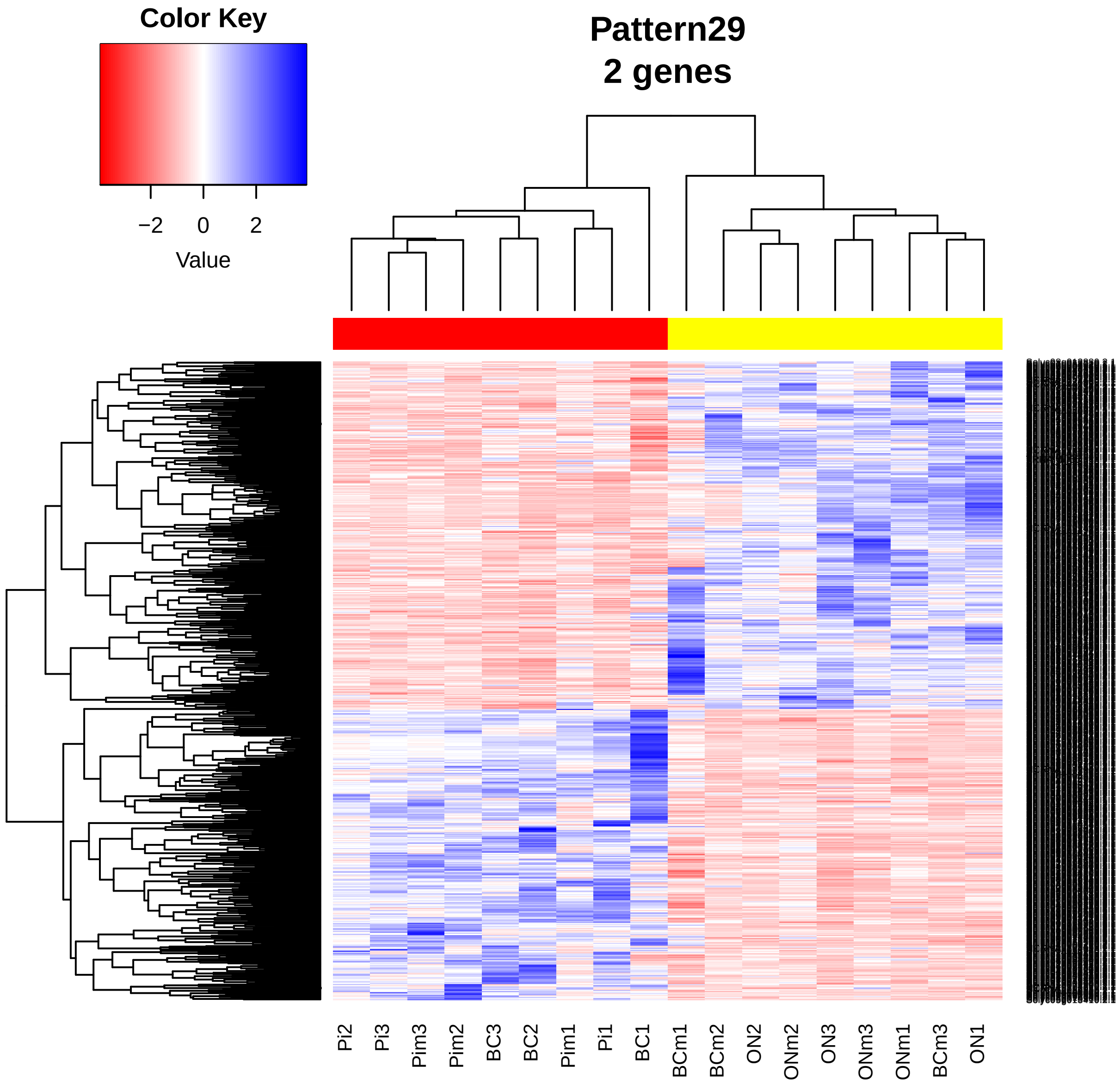

Color Key
−2
0
2
Value
Pattern29
2 genes
Pi2
Pi3
Pim3
Pim2
BC3
BC2
Pim1
Pi1
BC1
BCm1
BCm2
ON2
ONm2
ON3
ONm3
ONm1
BCm3
ON1

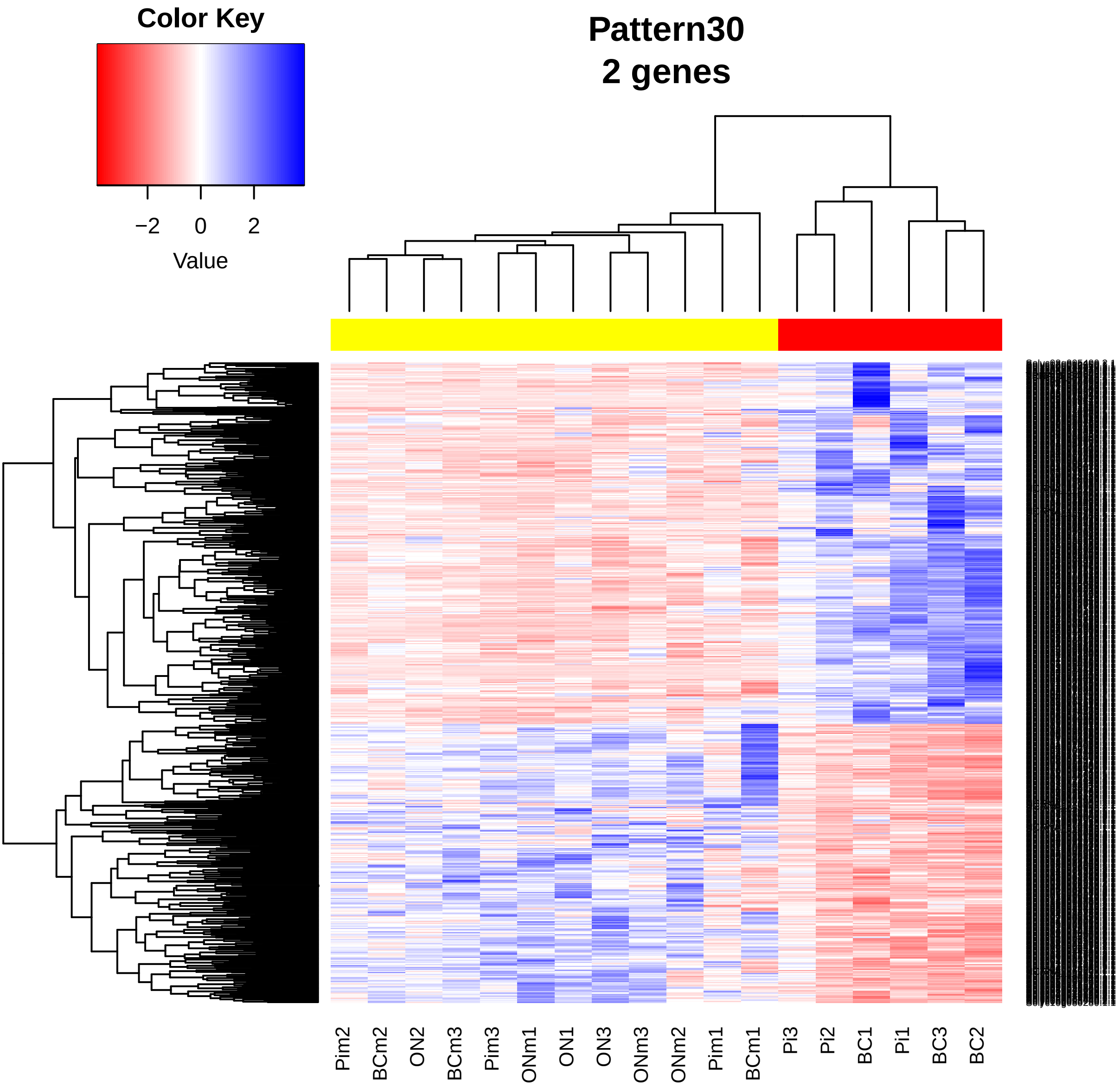
Color Key
−2
0
2
Value
Pattern30
2 genes
Pim2
BCm2
ON2
BCm3
Pim3
ONm1
ON1
ON3
ONm3
ONm2
Pim1
BCm1
Pi3
Pi2
BC1
Pi1
BC3
BC2

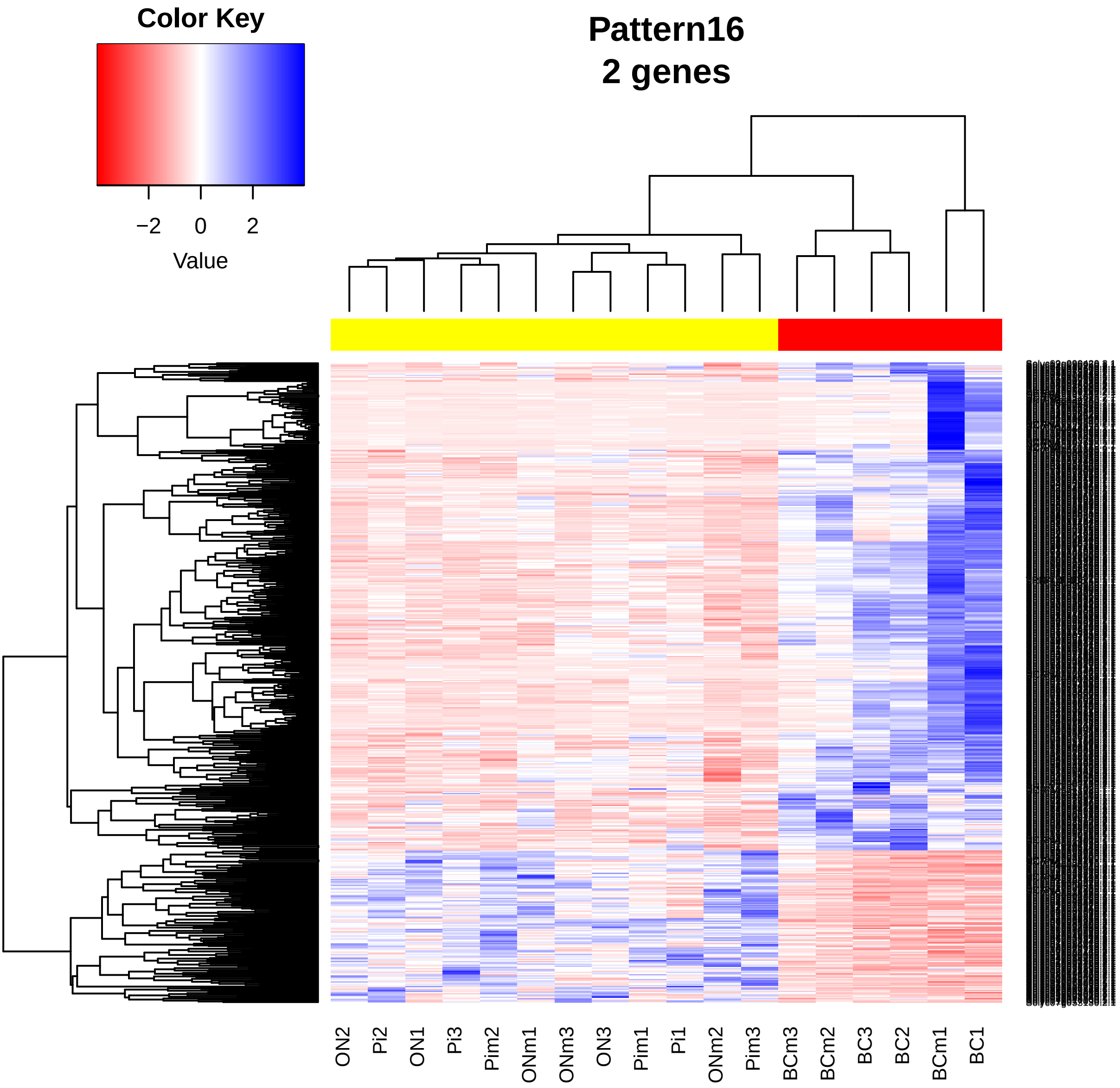
Color Key
−2
0
2
Value
Pattern16
2 genes
ON2
Pi2
ON1
Pi3
Pim2
ONm1
ONm3
ON3
Pim1
Pi1
ONm2
Pim3
BCm3
BCm2
BC3
BC2
BCm1
BC1

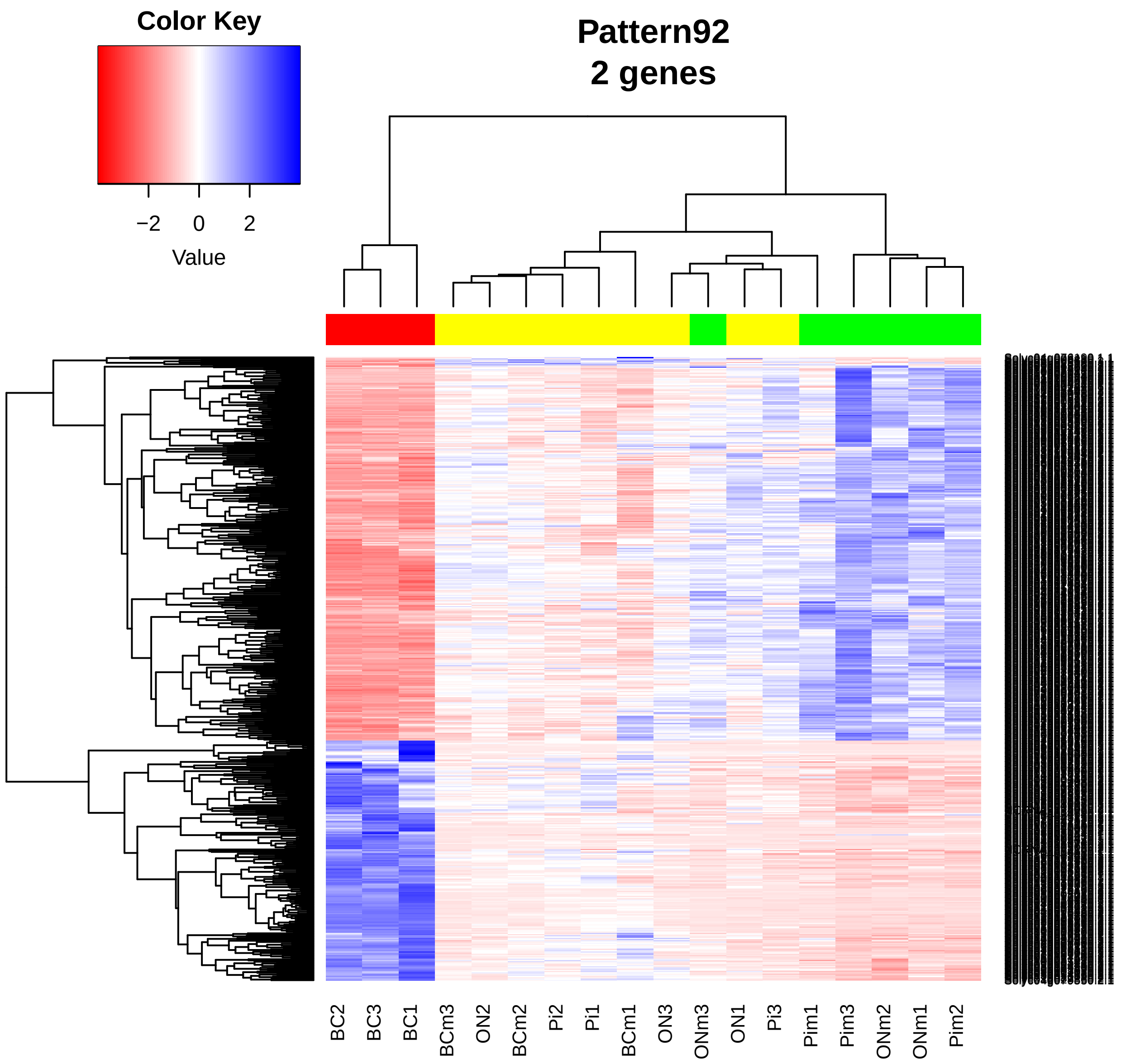
Color Key
−2
0
2
Value
Pattern92
2 genes
BC2
BC3
BC1
BCm3
ON2
BCm2
Pi2
Pi1
BCm1
ON3
ONm3
ON1
Pi3
Pim1
Pim3
ONm2
ONm1
Pim2

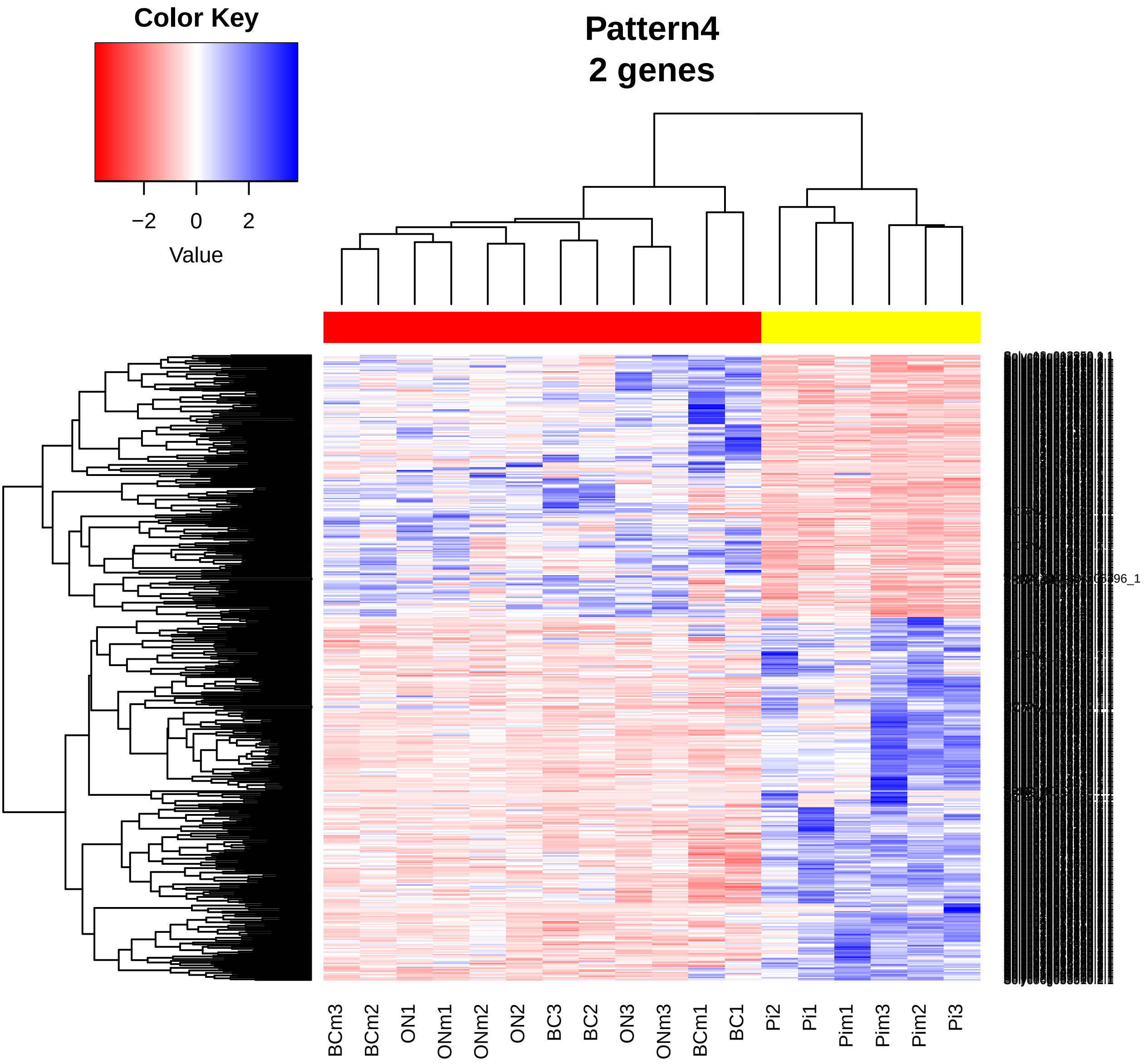
Color Key
−2
0
2
Value
Pattern4
2 genes
BCm3
BCm2
ON1
ONm1
ONm2
ON2
BC3
BC2
ON3
ONm3
BCm1
BC1
Pi2
Pi1
Pim1
Pim3
Pim2
Pi3

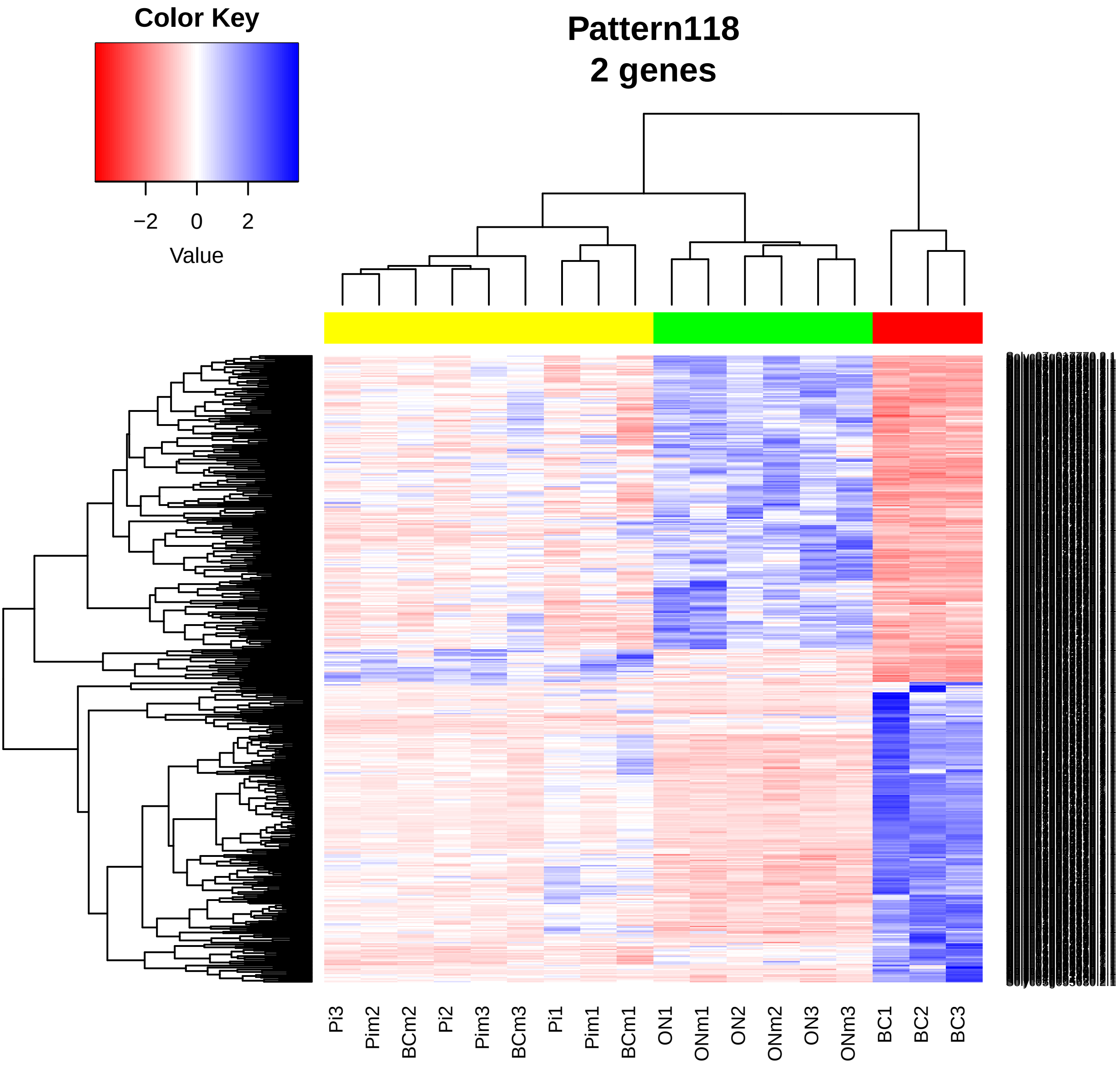
Color Key
−2
0
2
Value
Pattern118
2 genes
Pi3
Pim2
BCm2
Pi2
Pim3
BCm3
Pi1
Pim1
BCm1
ON1
ONm1
ON2
ONm2
ON3
ONm3
BC1
BC2
BC3

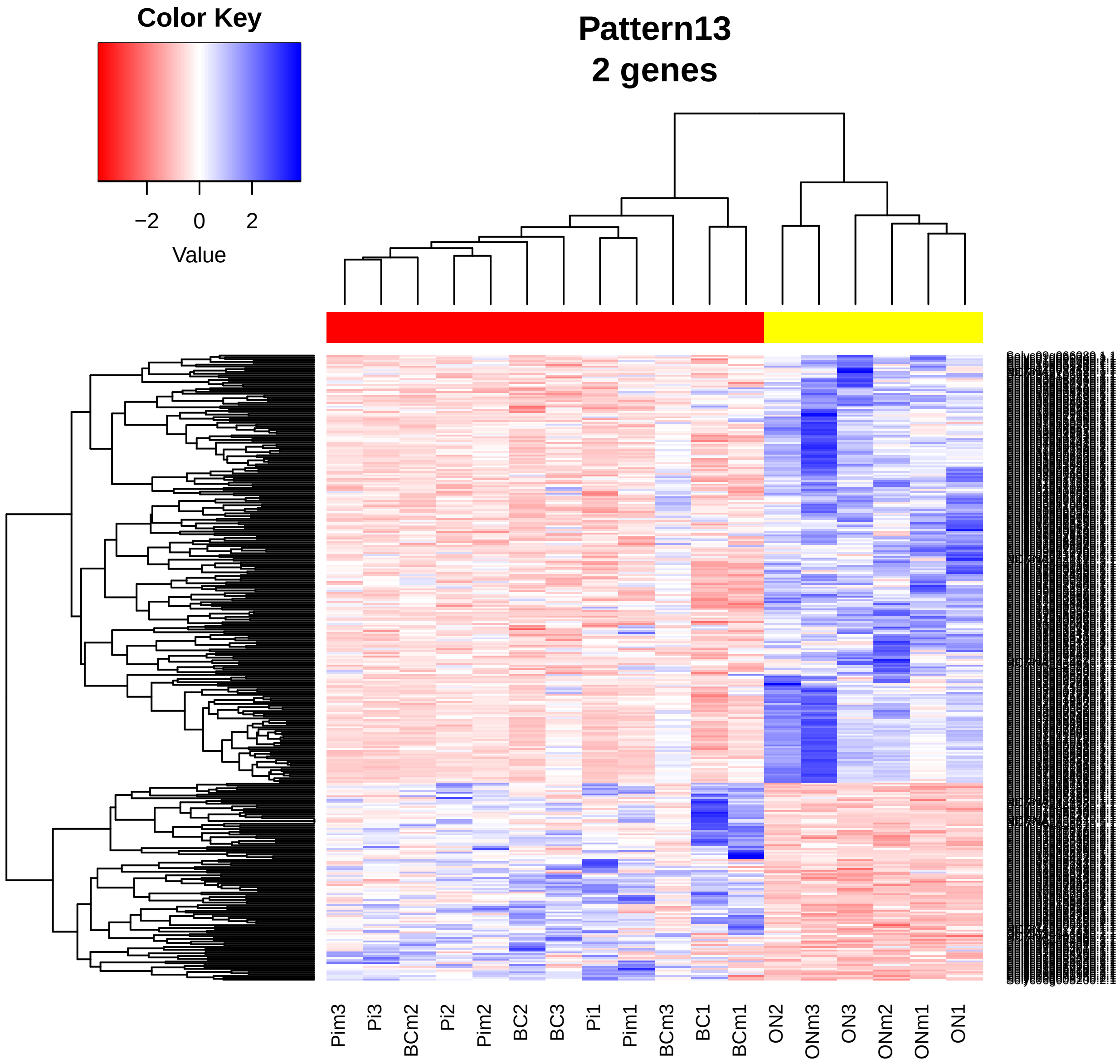
Color Key
−2
0
2
Value
Pattern13
2 genes
Pim3
Pi3
BCm2
Pi2
Pim2
BC2
BC3
Pi1
Pim1
BCm3
BC1
BCm1
ON2
ONm3
ON3
ONm2
ONm1
ON1

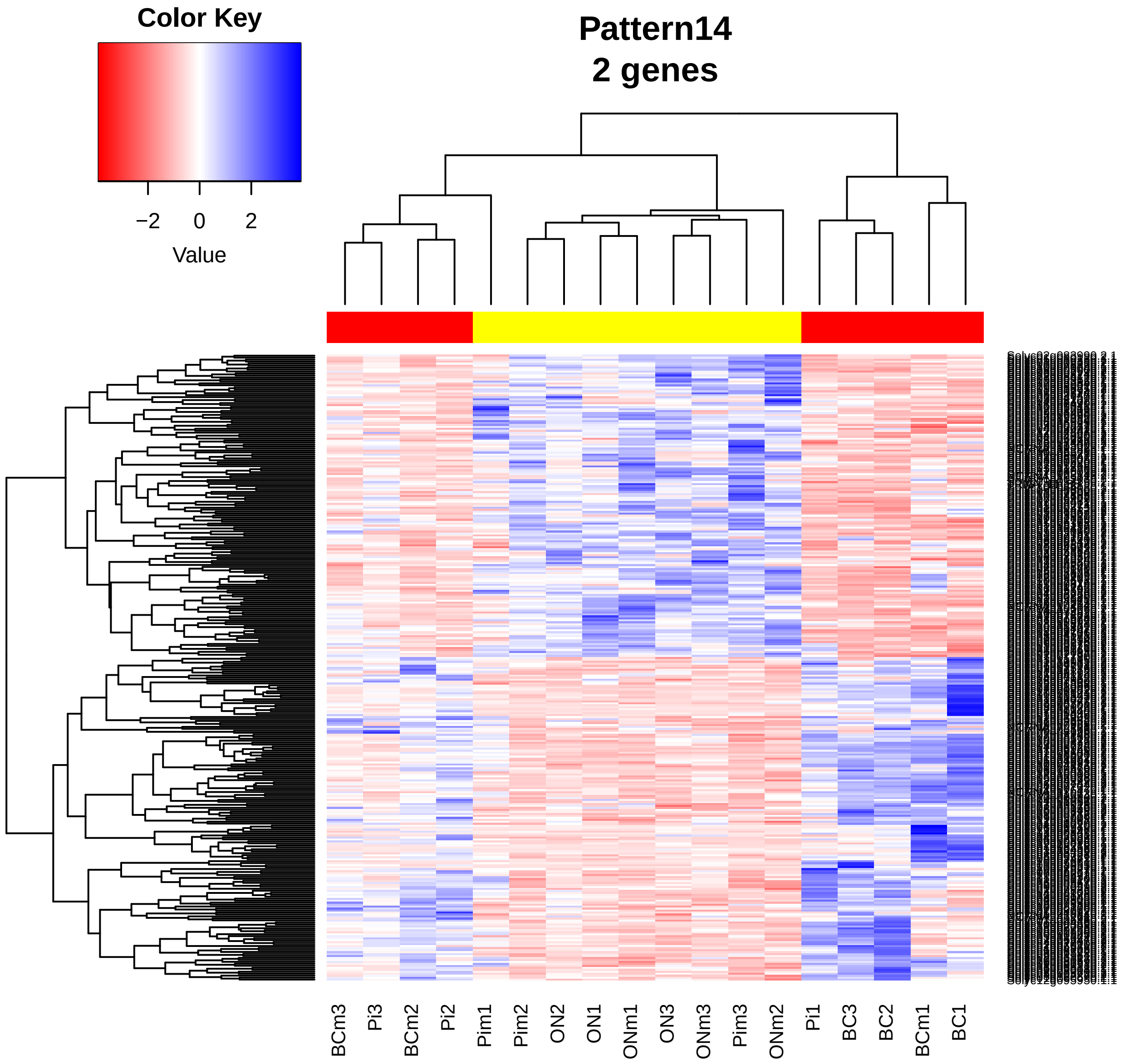
Color Key
−2
0
2
Value
Pattern14
2 genes
BCm3
Pi3
BCm2
Pi2
Pim1
Pim2
ON2
ON1
ONm1
ON3
ONm3
Pim3
ONm2
Pi1
BC3
BC2
BCm1
BC1

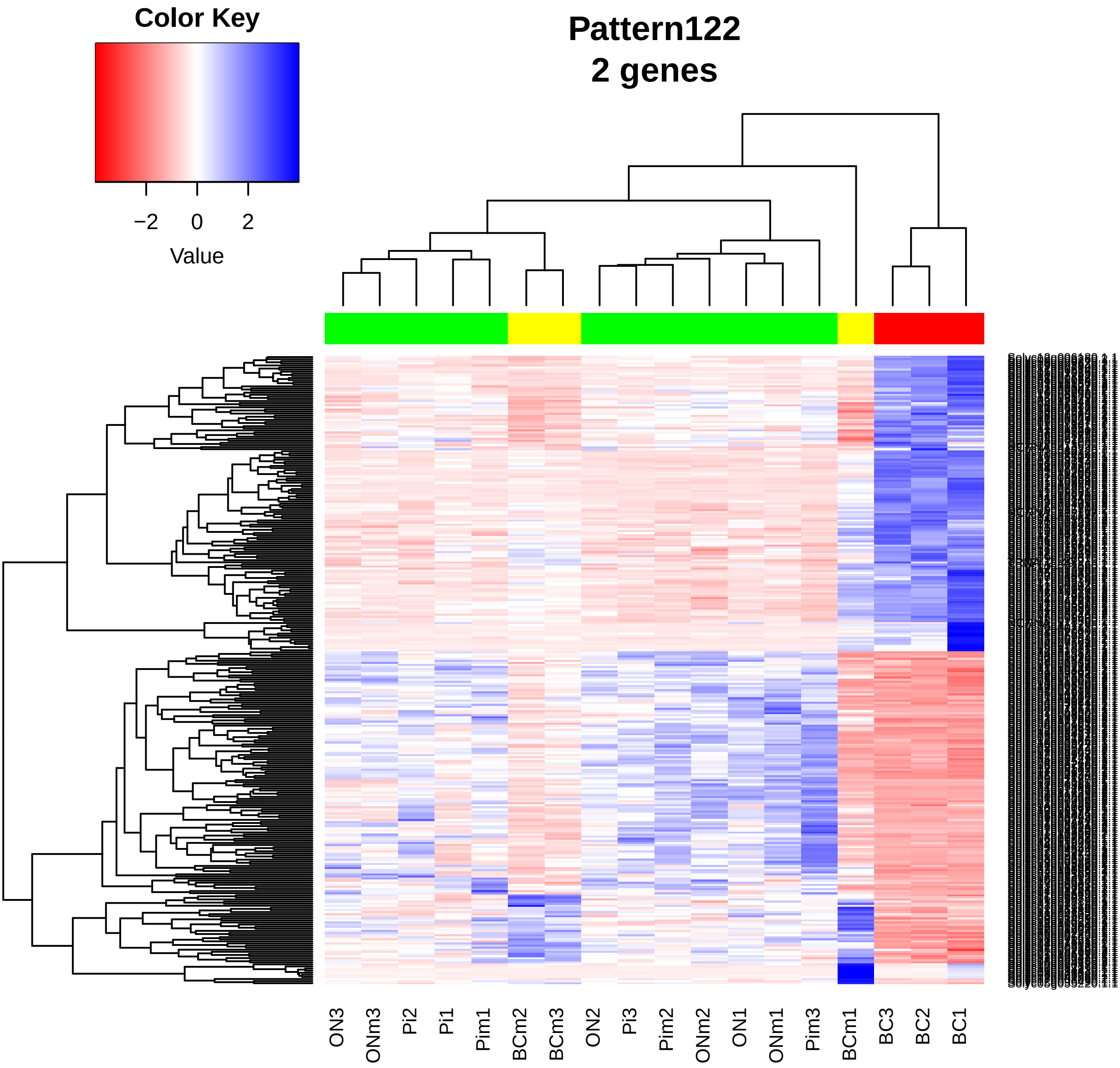
Color Key
−2
0
2
Value
Pattern122
2 genes
ON3
ONm3
Pi2
Pi1
Pim1
BCm2
BCm3
ON2
Pi3
Pim2
ONm2
ON1
ONm1
Pim3
BCm1
BC3
BC2
BC1

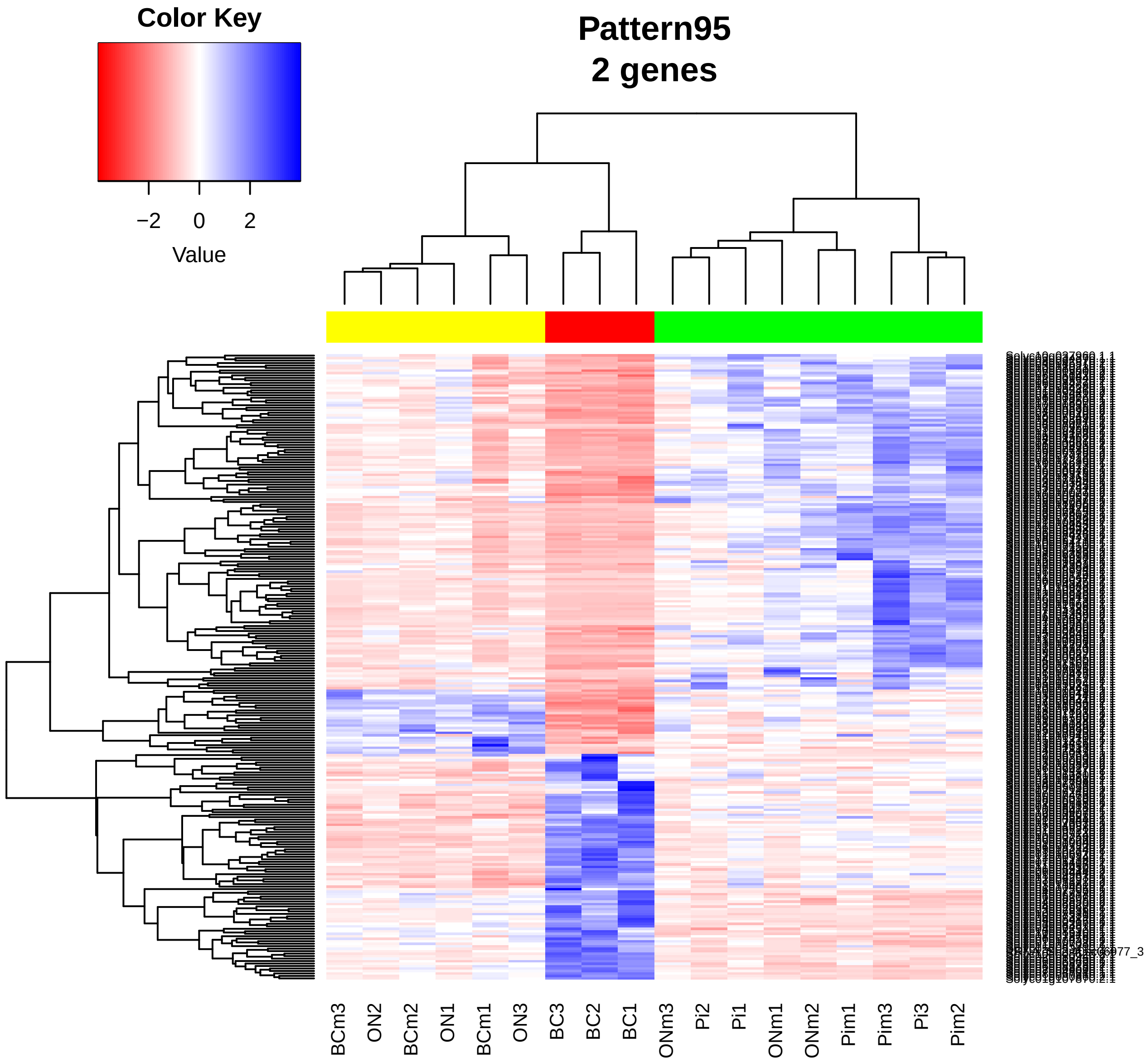
Color Key
−2
0
2
Value
Pattern95
2 genes
BCm3
ON2
BCm2
ON1
BCm1
ON3
BC3
BC2
BC1
ONm3
Pi2
Pi1
ONm1
ONm2
Pim1
Pim3
Pi3
Pim2

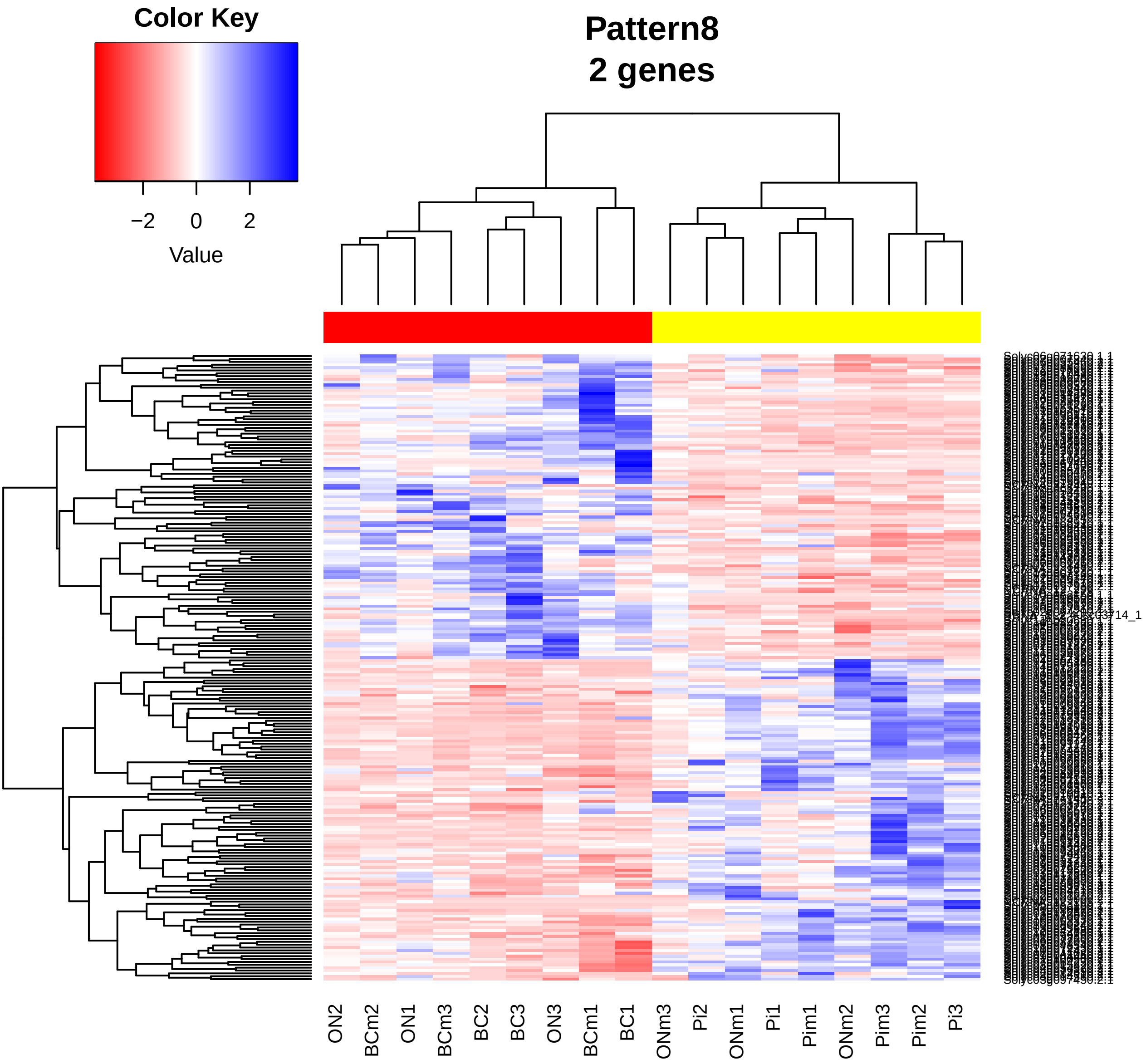
Color Key
−2
0
2
Value
Pattern8
2 genes
ON2
BCm2
ON1
BCm3
BC2
BC3
ON3
BCm1
BC1
ONm3
Pi2
ONm1
Pi1
Pim1
ONm2
Pim3
Pim2
Pi3

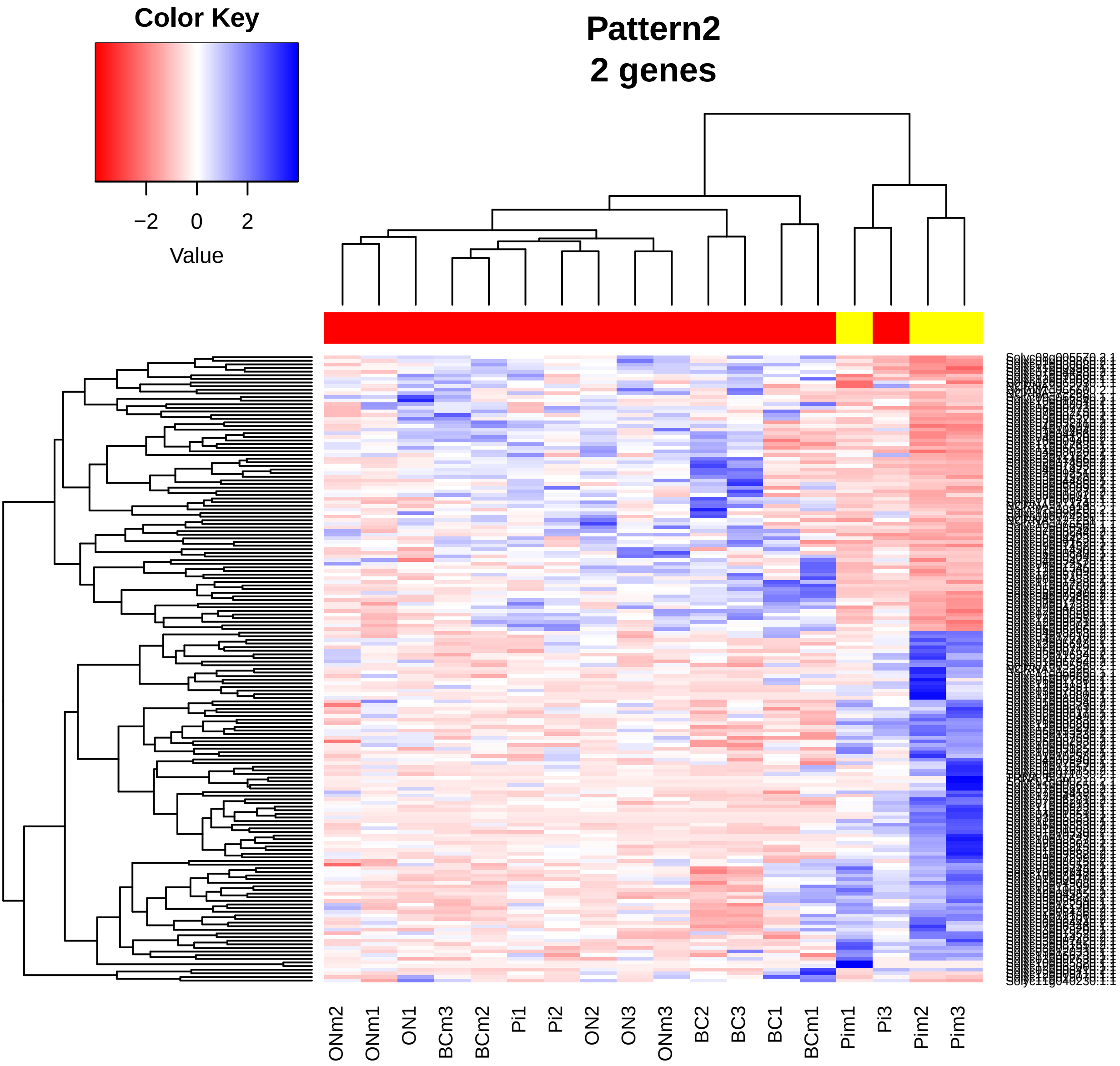
Color Key
−2
0
2
Value
Pattern2
2 genes
ONm2
ONm1
ON1
BCm3
BCm2
Pi1
Pi2
ON2
ON3
ONm3
BC2
BC3
BC1
BCm1
Pim1
Pi3
Pim2
Pim3

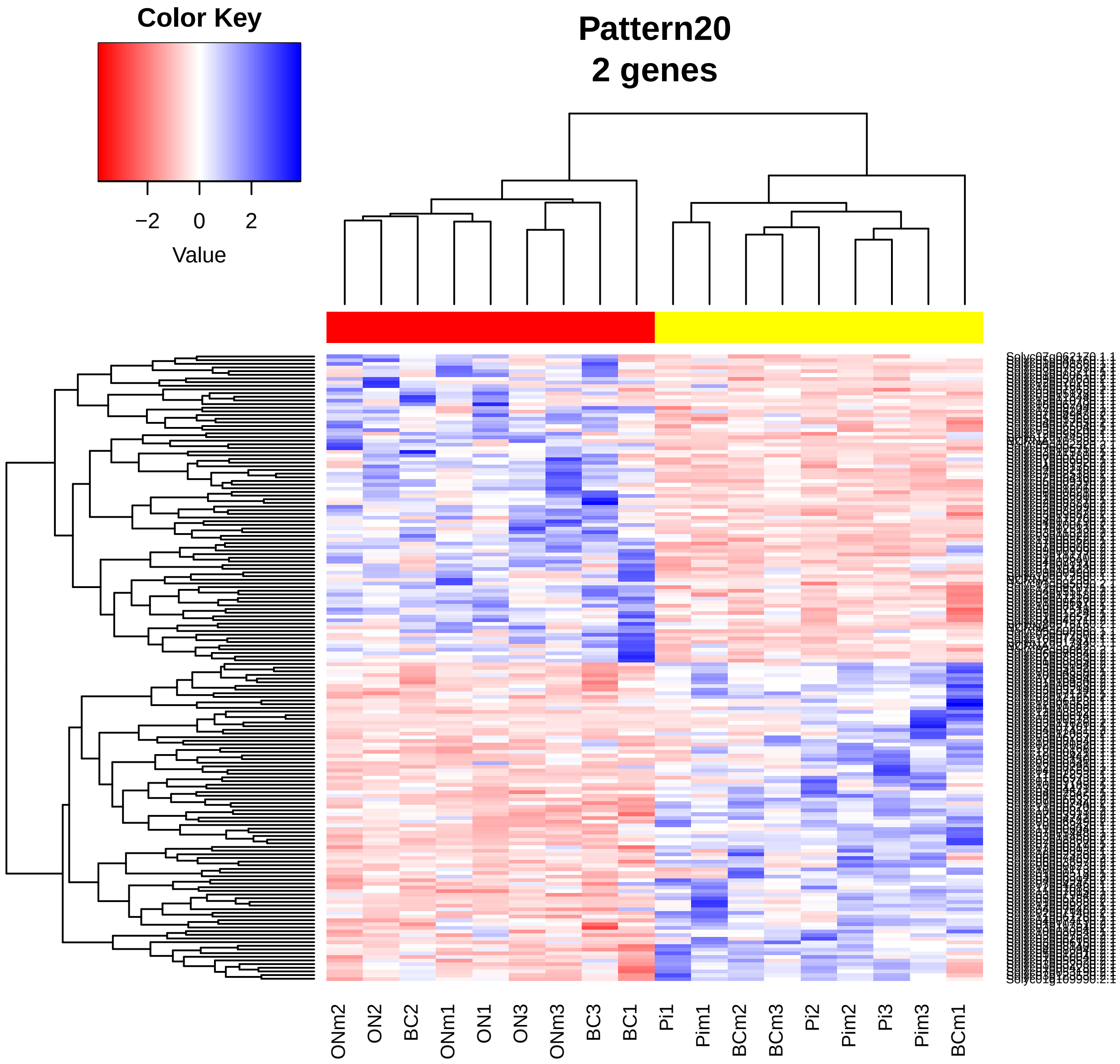

Color Key
−2
0
2
Value
Pattern20
2 genes
ONm2
ON2
BC2
ONm1
ON1
ON3
ONm3
BC3
BC1
Pi1
Pim1
BCm2
BCm3
Pi2
Pim2
Pi3
Pim3
BCm1

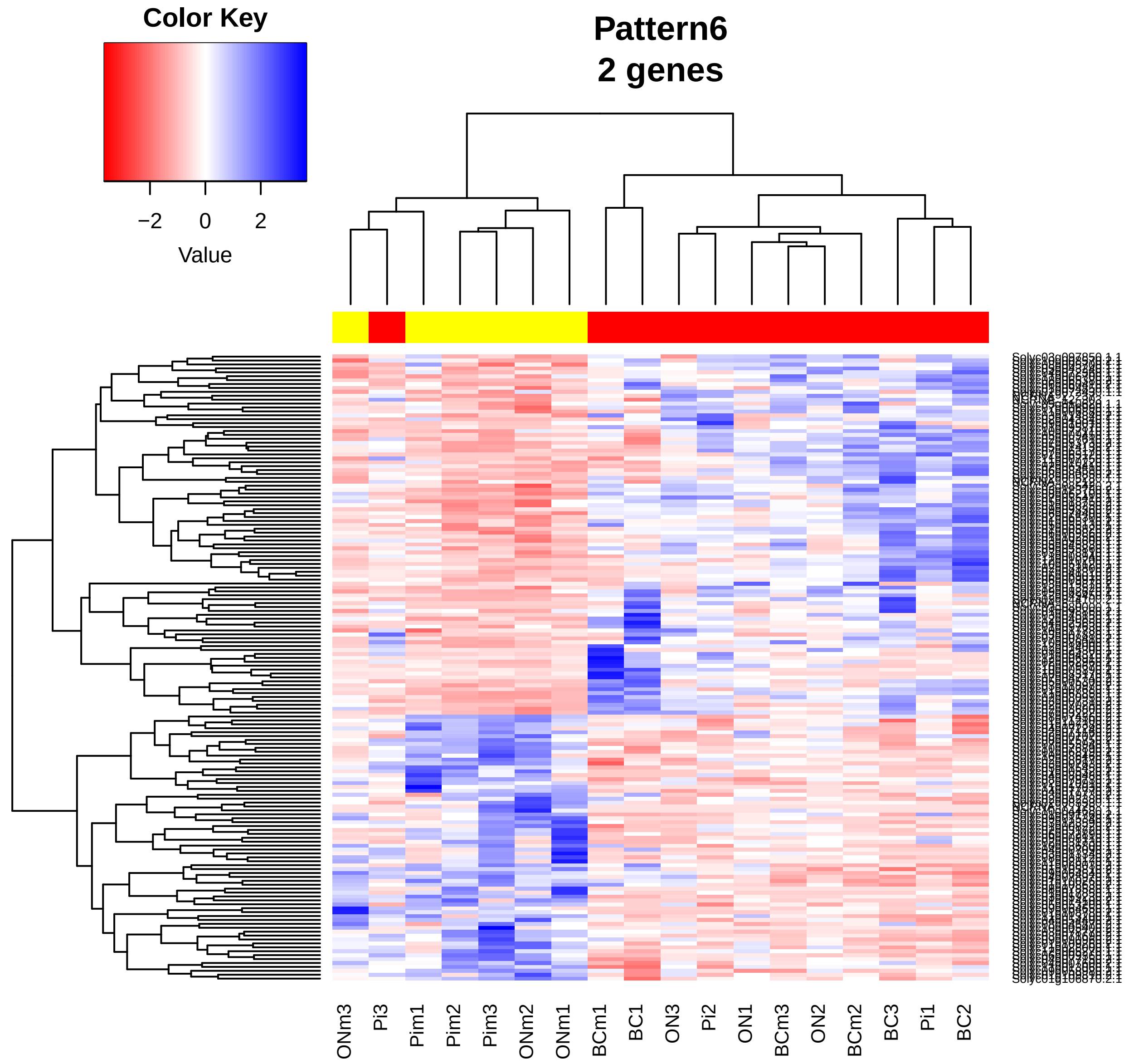
Pattern6
2 genes
Color Key
−2
0
2
Value
ONm3
Pi3
Pim1
Pim2
Pim3
ONm2
ONm1
BCm1
BC1
ON3
Pi2
ON1
BCm3
ON2
BCm2
BC3
Pi1
BC2

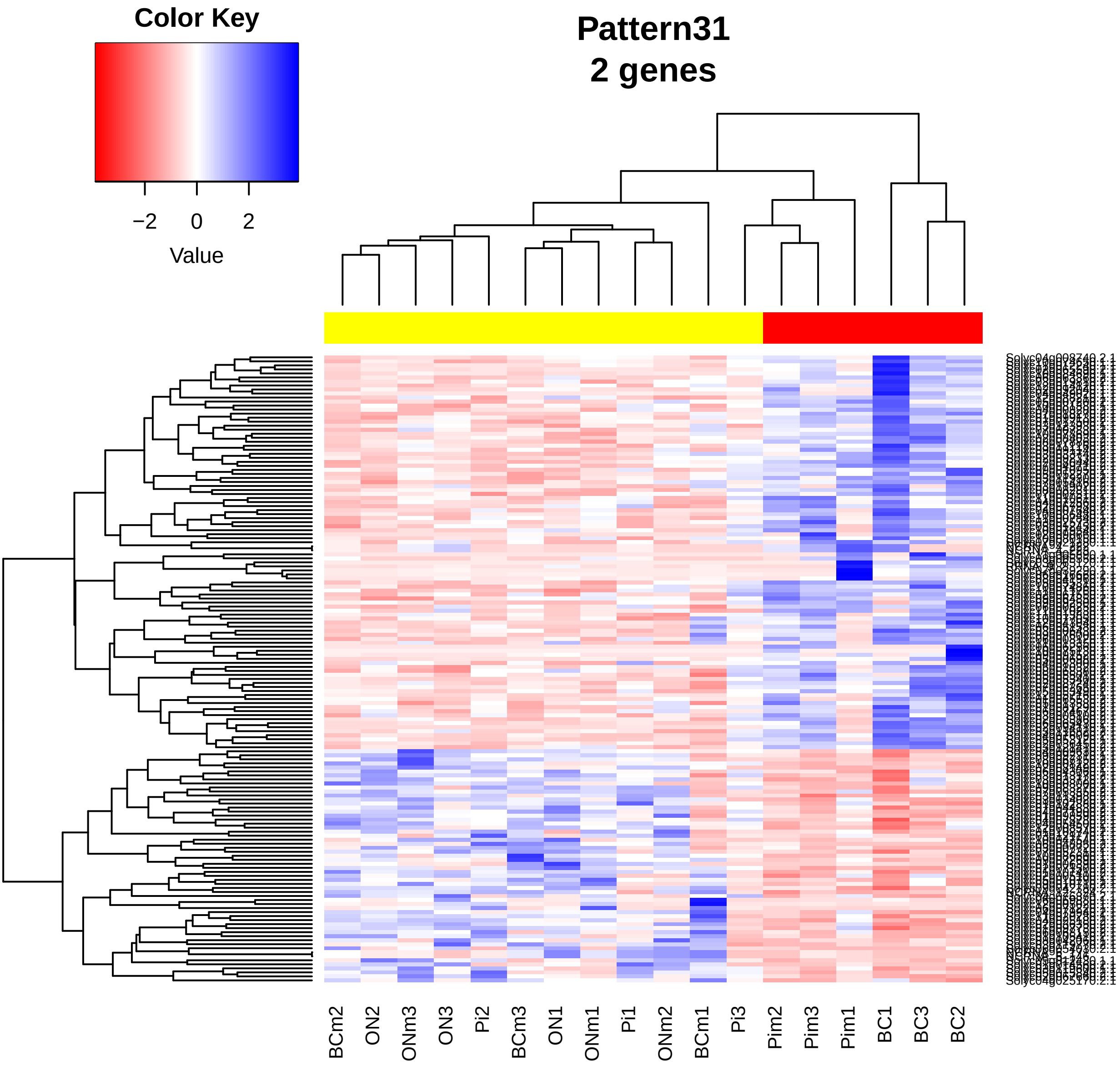
Color Key
−2
0
2
Value
Pattern31
2 genes
BCm2
ON2
ONm3
ON3
Pi2
BCm3
ON1
ONm1
Pi1
ONm2
BCm1
Pi3
Pim2
Pim3
Pim1
BC1
BC3
BC2
Solyc04g008740.2.1
Solyc04g025170.2.1

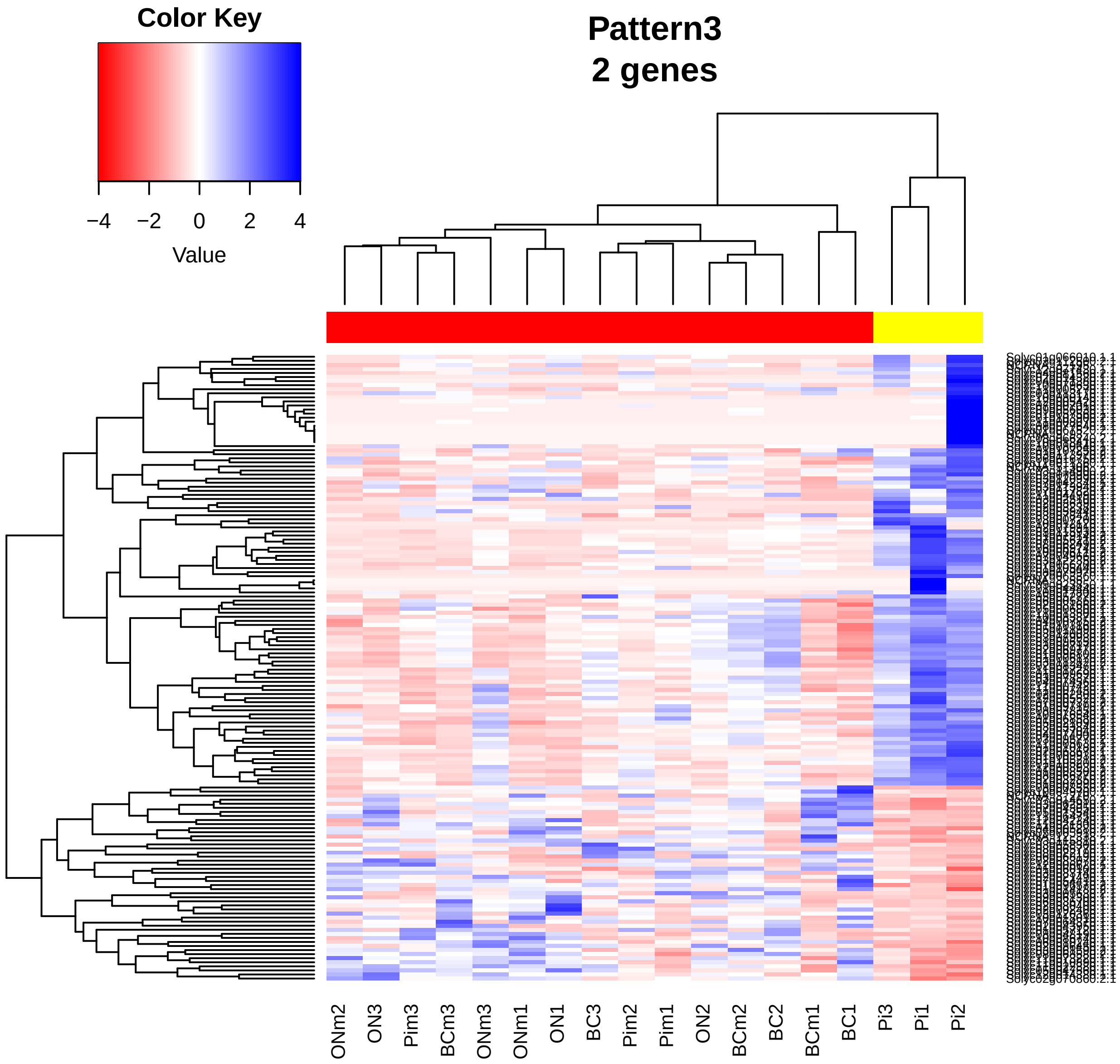
Color Key
−4
−2
0
2
4
Value
Pattern3
2 genes
ONm2
ON3
Pim3
BCm3
ONm3
ONm1
ON1
BC3
Pim2
Pim1
ON2
BCm2
BC2
BCm1
BC1
Pi3
Pi1
Pi2

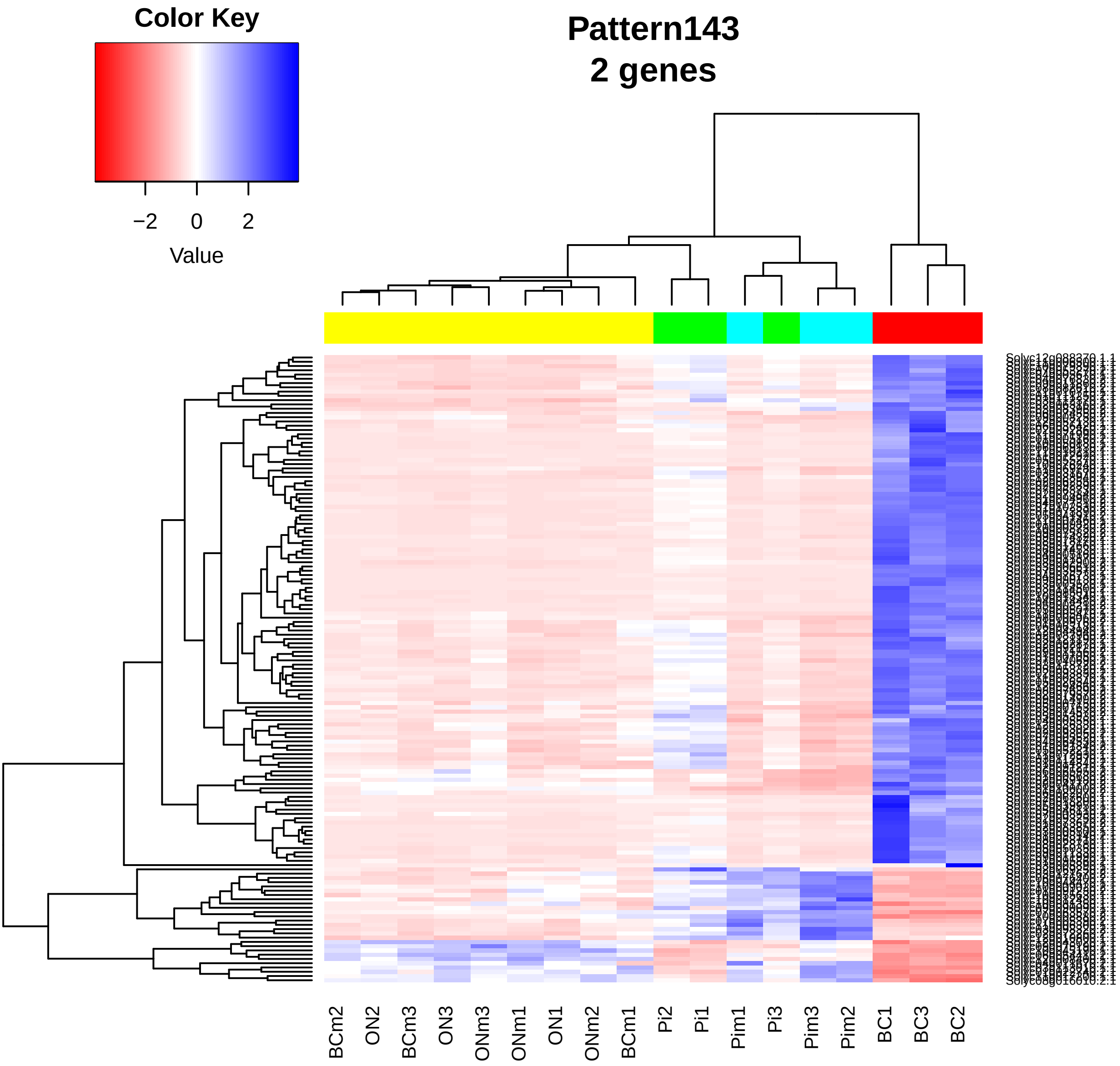
Color Key
−2
0
2
Value
Pattern143
2 genes
BCm2
ON2
BCm3
ON3
ONm3
ONm1
ON1
ONm2
BCm1
Pi2
Pi1
Pim1
Pi3
Pim3
Pim2
BC1
BC3
BC2

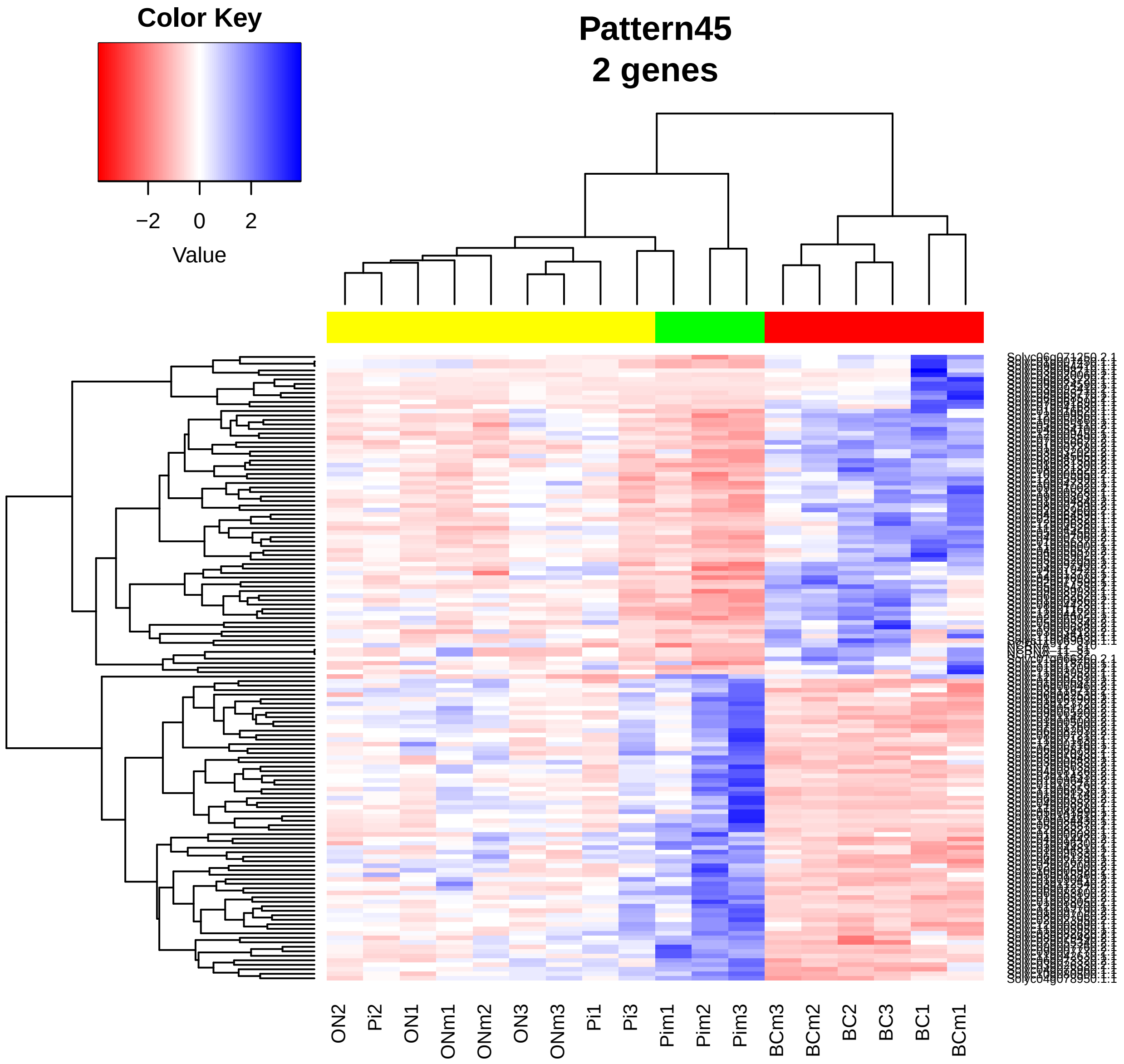

Color Key
−2
0
2
Value
Pattern45
2 genes
ON2
Pi2
ON1
ONm1
ONm2
ON3
ONm3
Pi1
Pi3
Pim1
Pim2
Pim3
BCm3
BCm2
BC2
BC3
BC1
BCm1

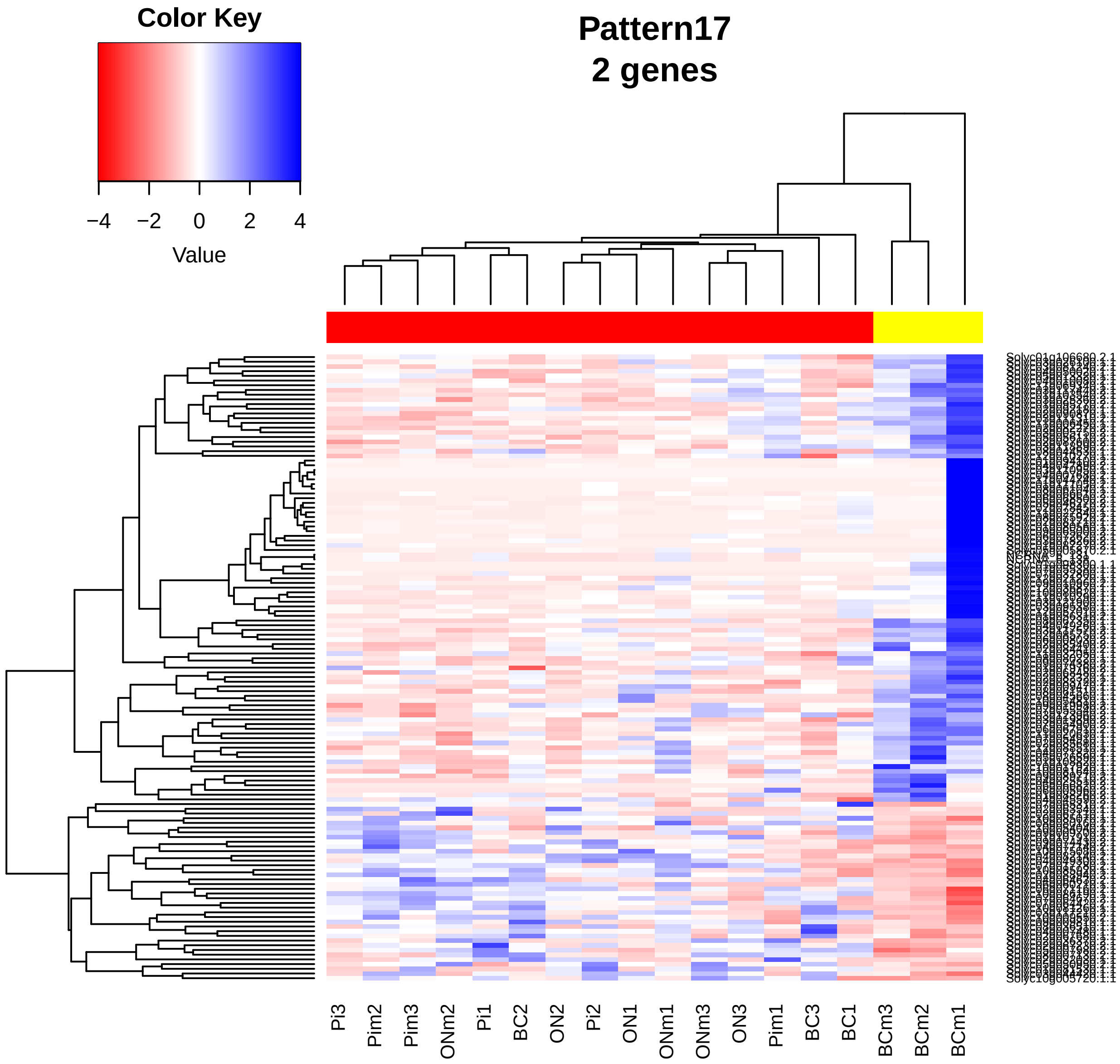
Color Key
−4
−2
0
2
4
Value
Pattern17
2 genes
Pi3
Pim2
Pim3
ONm2
Pi1
BC2
ON2
Pi2
ON1
ONm1
ONm3
ON3
Pim1
BC3
BC1
BCm3
BCm2
BCm1
Solyc01g106680.2.1
Solyc10g005720.1.1

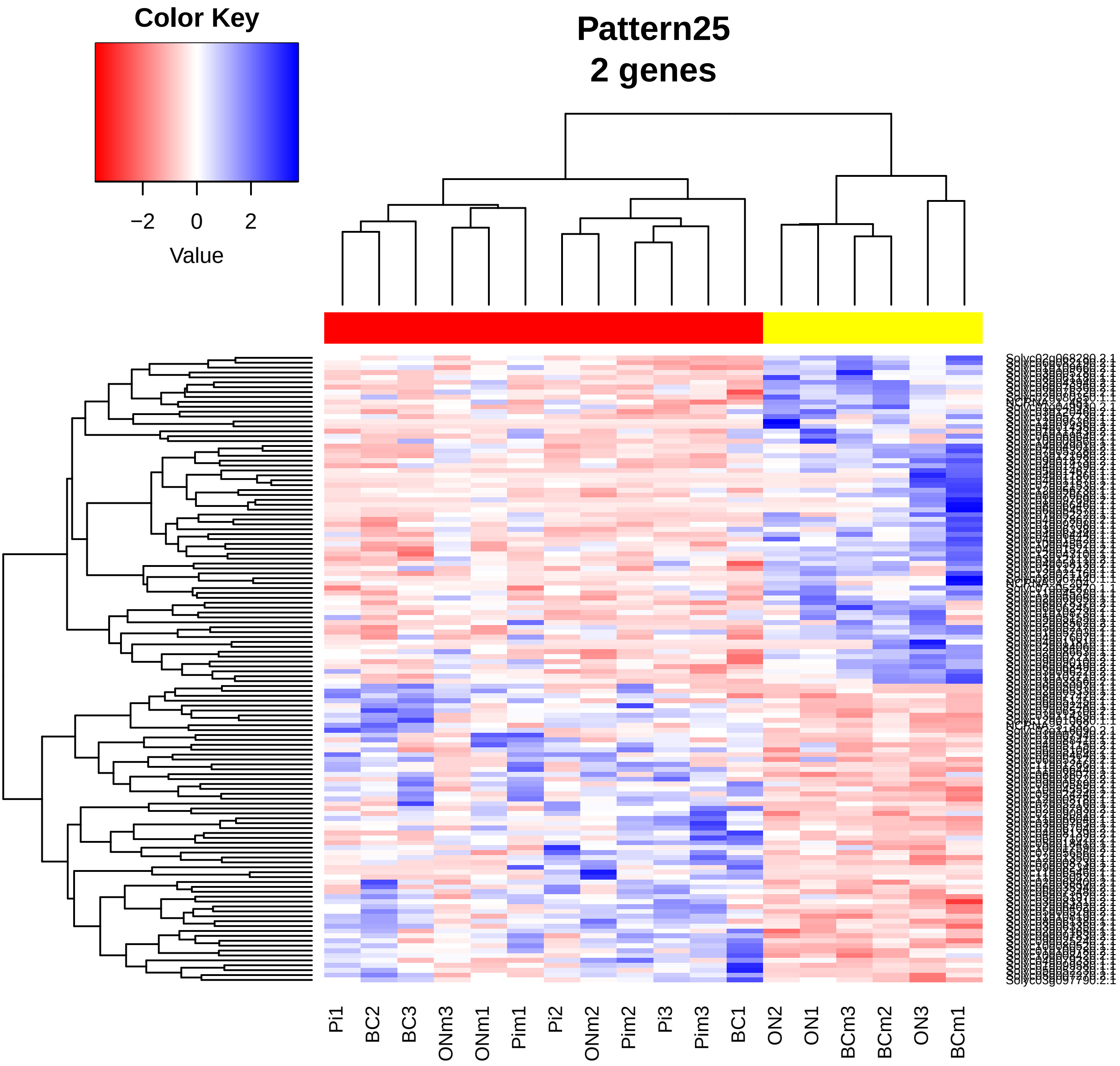
Color Key
−2
0
2
Value
Pattern25
2 genes
Solyc02g068280.2.1
Solyc03g097790.2.1
Pi1
BC2
BC3
ONm3
ONm1
Pim1
Pi2
ONm2
Pim2
Pi3
Pim3
BC1
ON2
ON1
BCm3
BCm2
ON3
BCm1

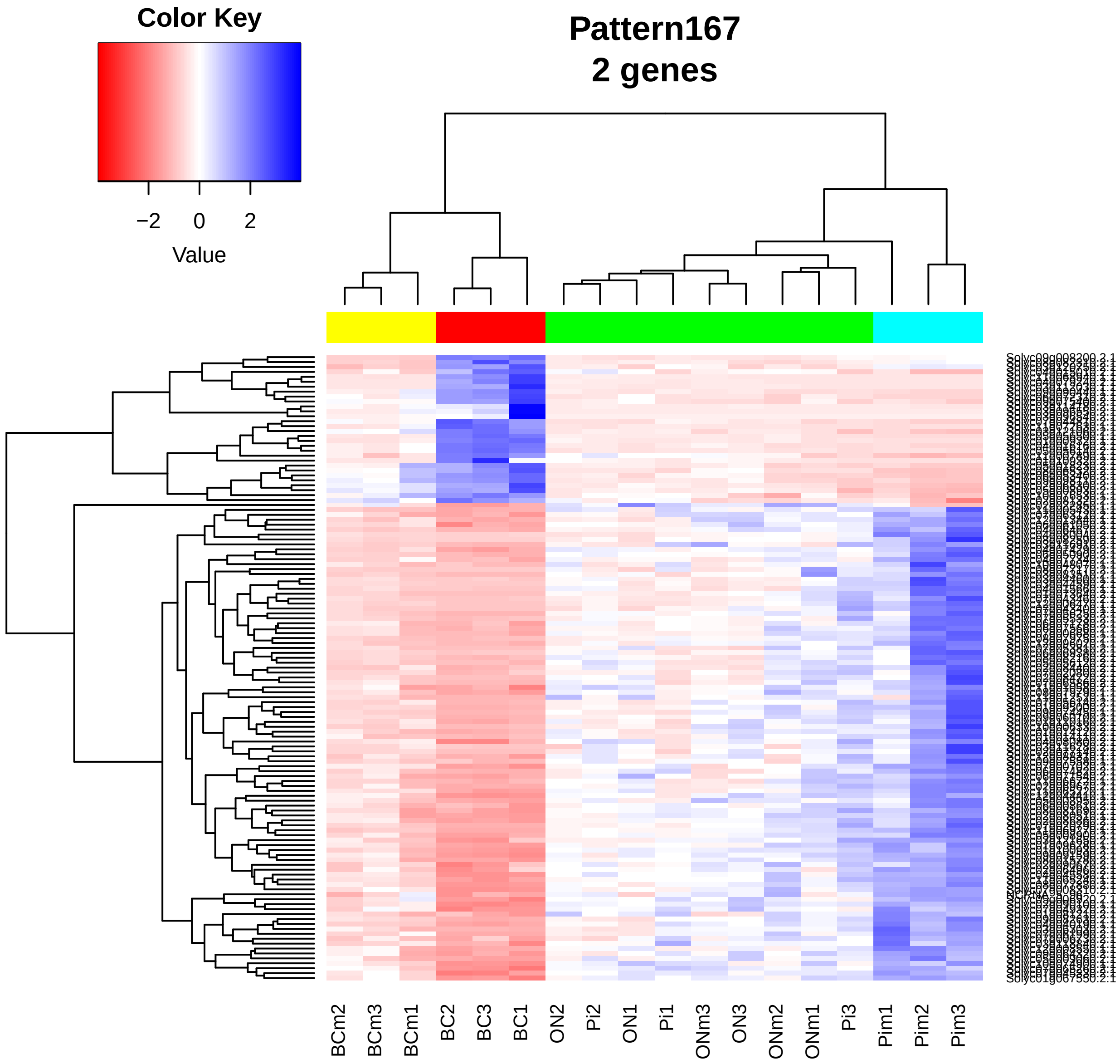
Color Key
−2
0
2
Value
Pattern167
2 genes
BCm2
BCm3
BCm1
BC2
BC3
BC1
ON2
Pi2
ON1
Pi1
ONm3
ON3
ONm2
ONm1
Pi3
Pim1
Pim2
Pim3

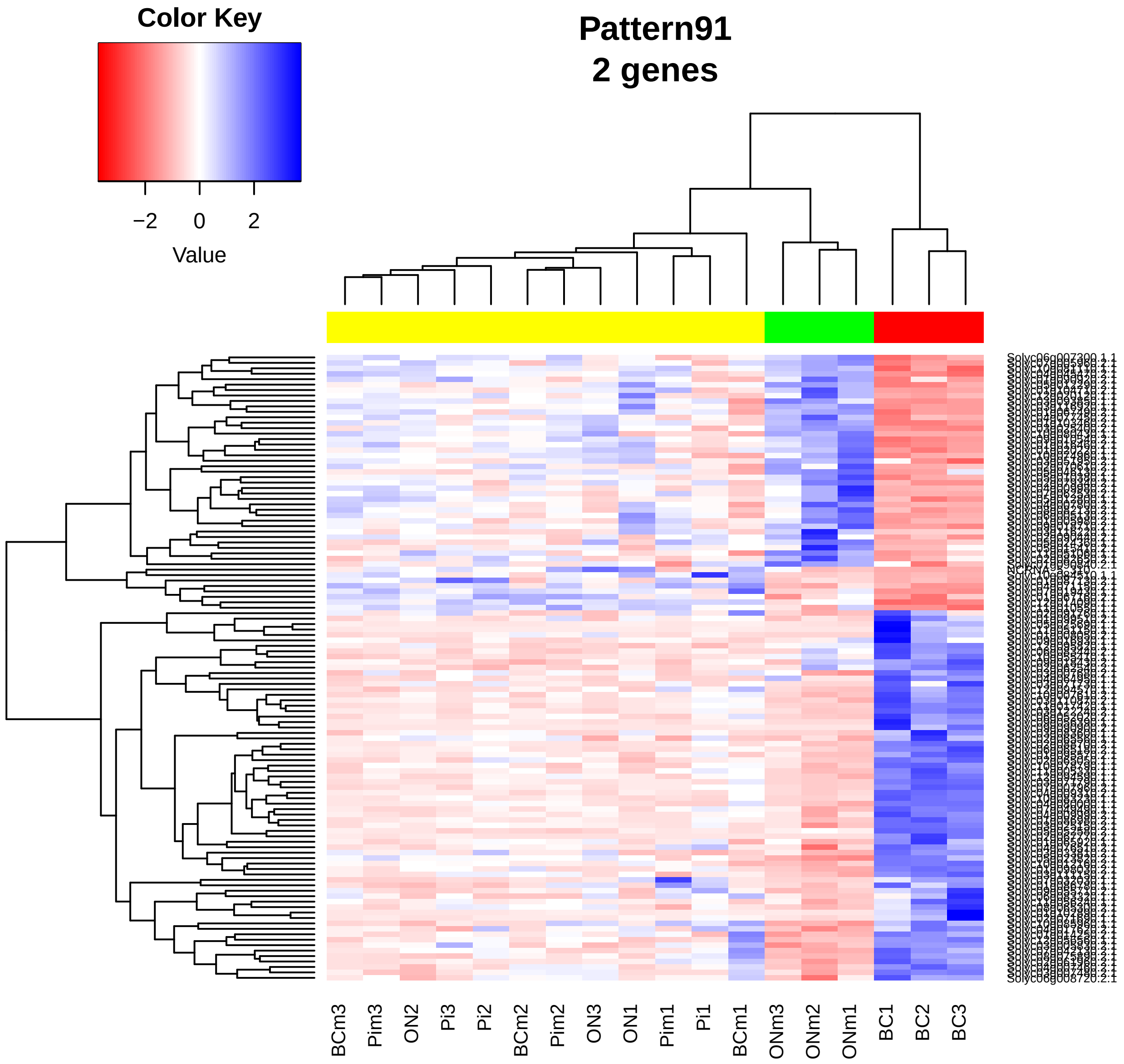
Color Key
−2
0
2
Value
Pattern91
2 genes
BCm3
Pim3
ON2
Pi3
Pi2
BCm2
Pim2
ON3
ON1
Pim1
Pi1
BCm1
ONm3
ONm2
ONm1
BC1
BC2
BC3

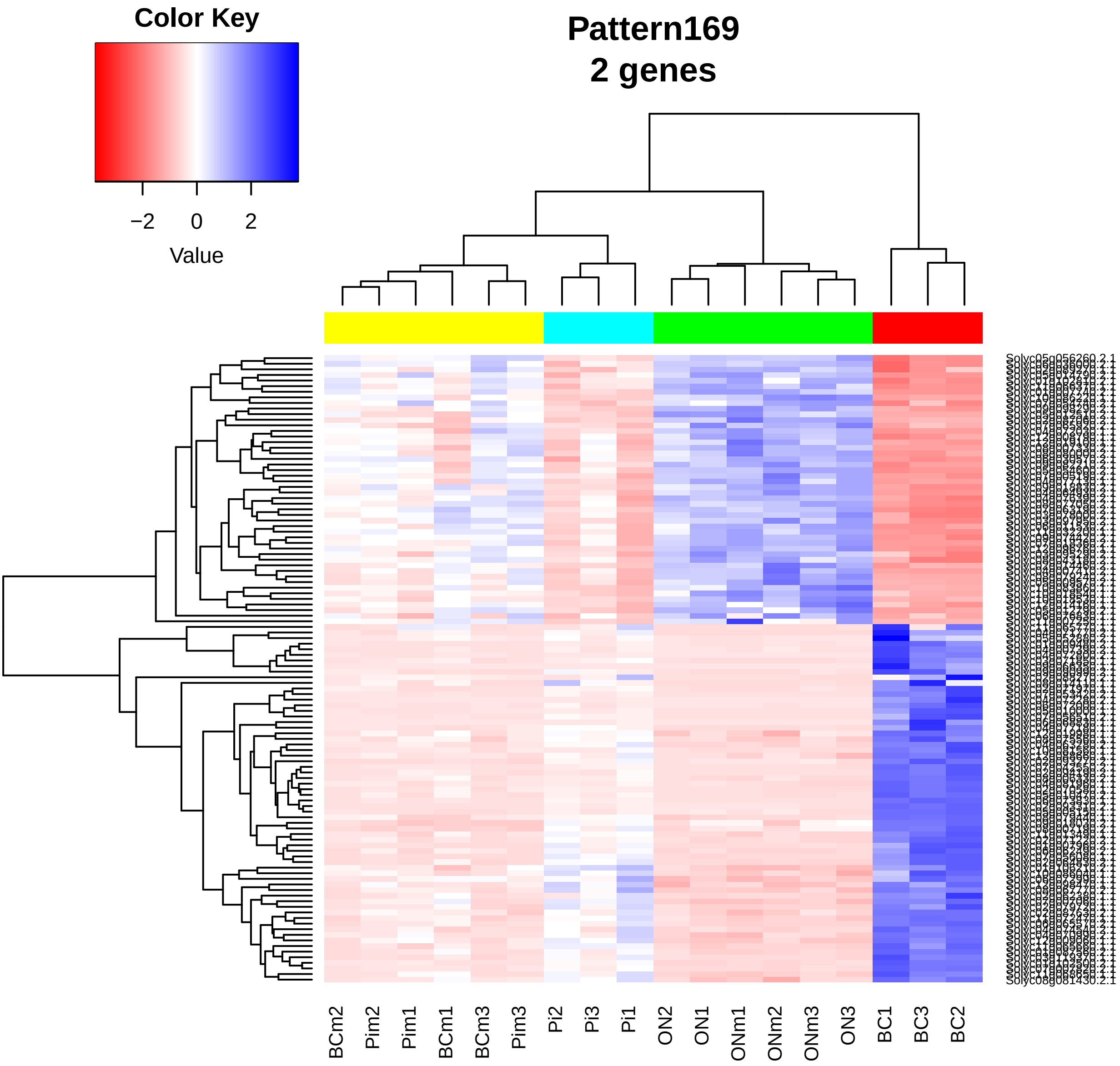
Color Key
−2
0
2
Value
Pattern169
2 genes
BCm2
Pim2
Pim1
BCm1
BCm3
Pim3
Pi2
Pi3
Pi1
ON2
ON1
ONm1
ONm2
ONm3
ON3
BC1
BC3
BC2
Solyc05g056260.2.1
Solyc08g081430.2.1

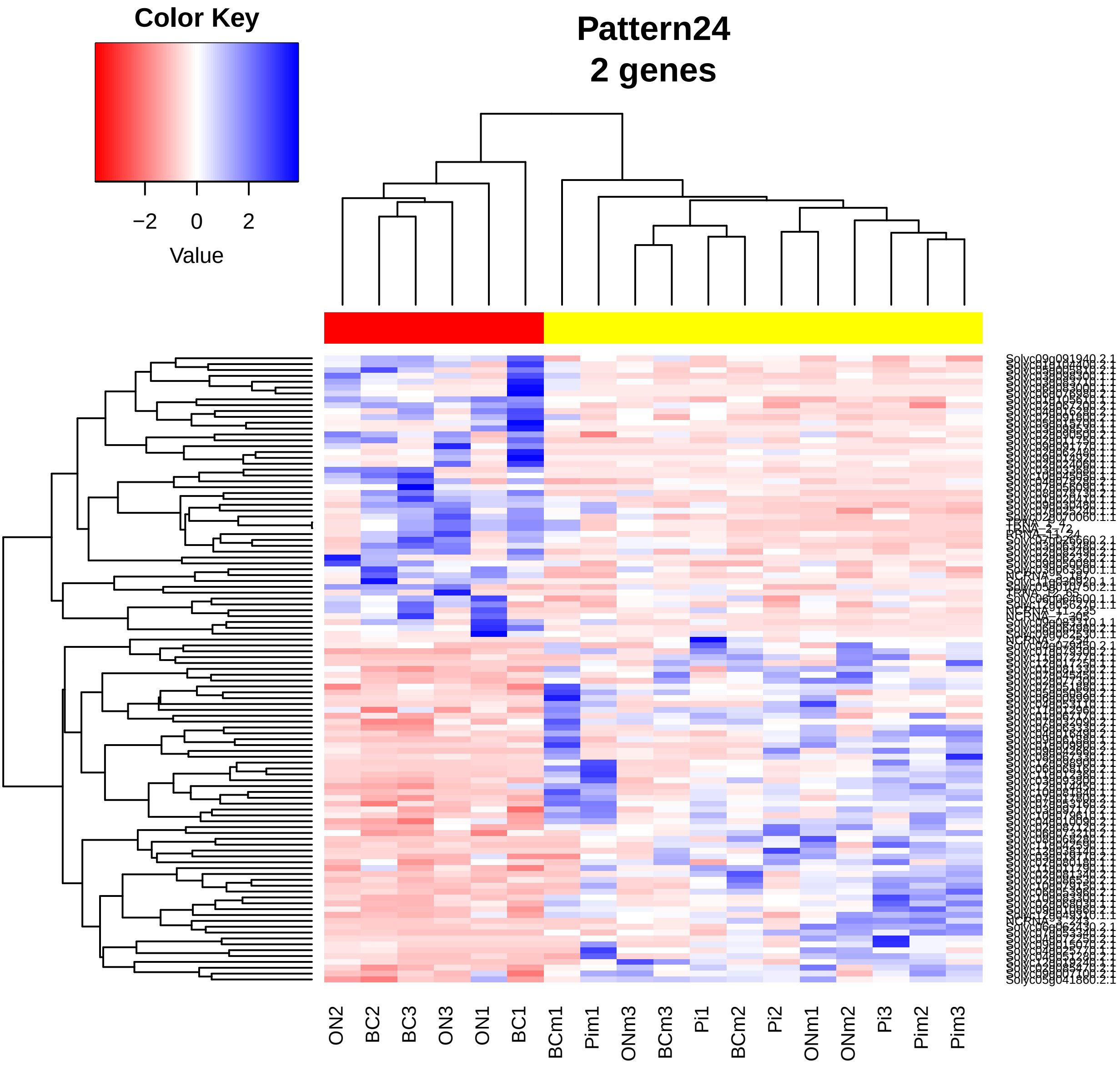
Color Key
−2
0
2
Value
Pattern24
2 genes
ON2
BC2
BC3
ON3
ON1
BC1
BCm1
Pim1
ONm3
BCm3
Pi1
BCm2
Pi2
ONm1
ONm2
Pi3
Pim2
Pim3

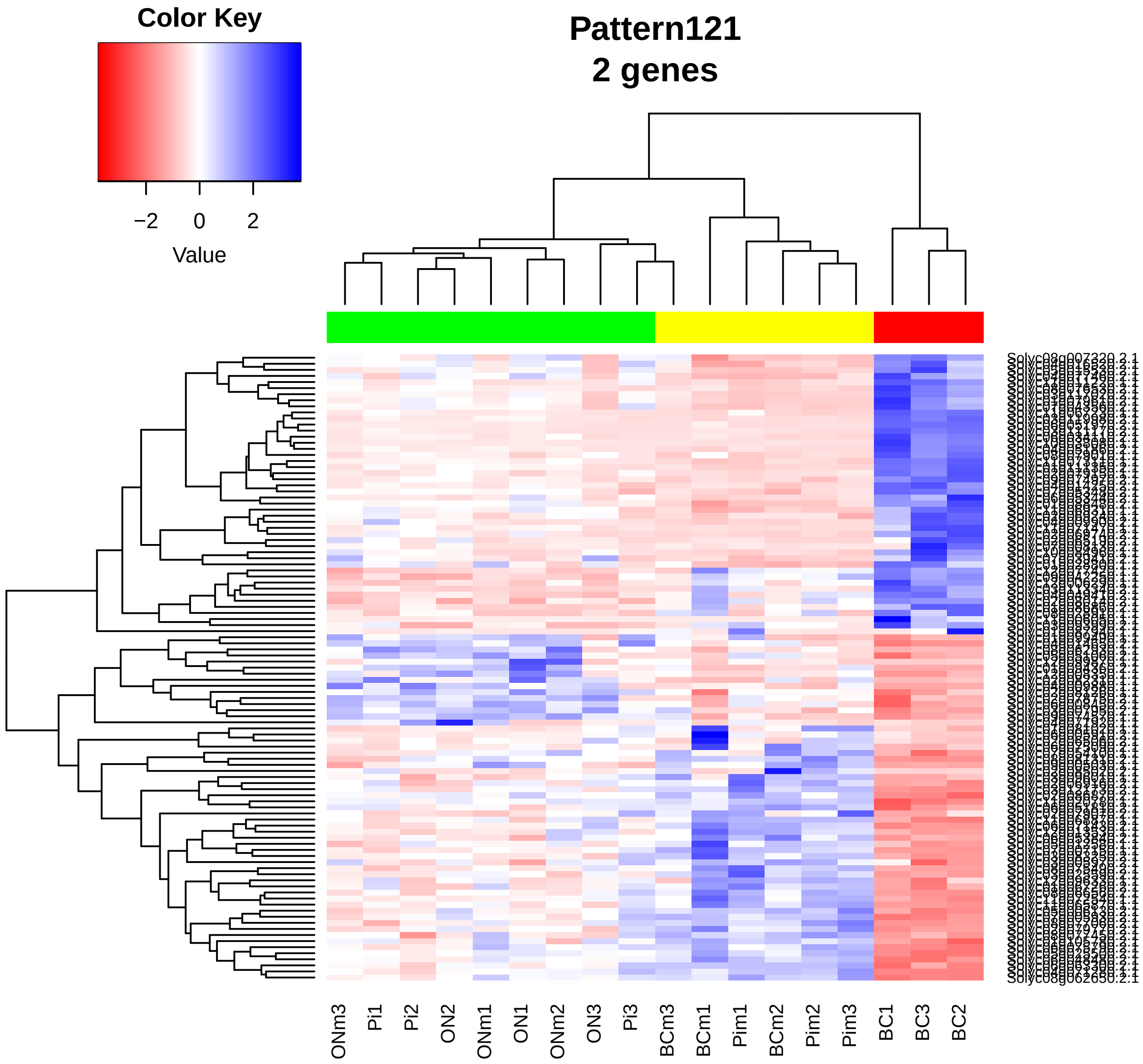
Color Key
−2
0
2
Value
Pattern121
2 genes
Solyc08g007320.2.1
Solyc08g062650.2.1
ONm3
Pi1
Pi2
ON2
ONm1
ON1
ONm2
ON3
Pi3
BCm3
BCm1
Pim1
BCm2
Pim2
Pim3
BC1
BC3
BC2

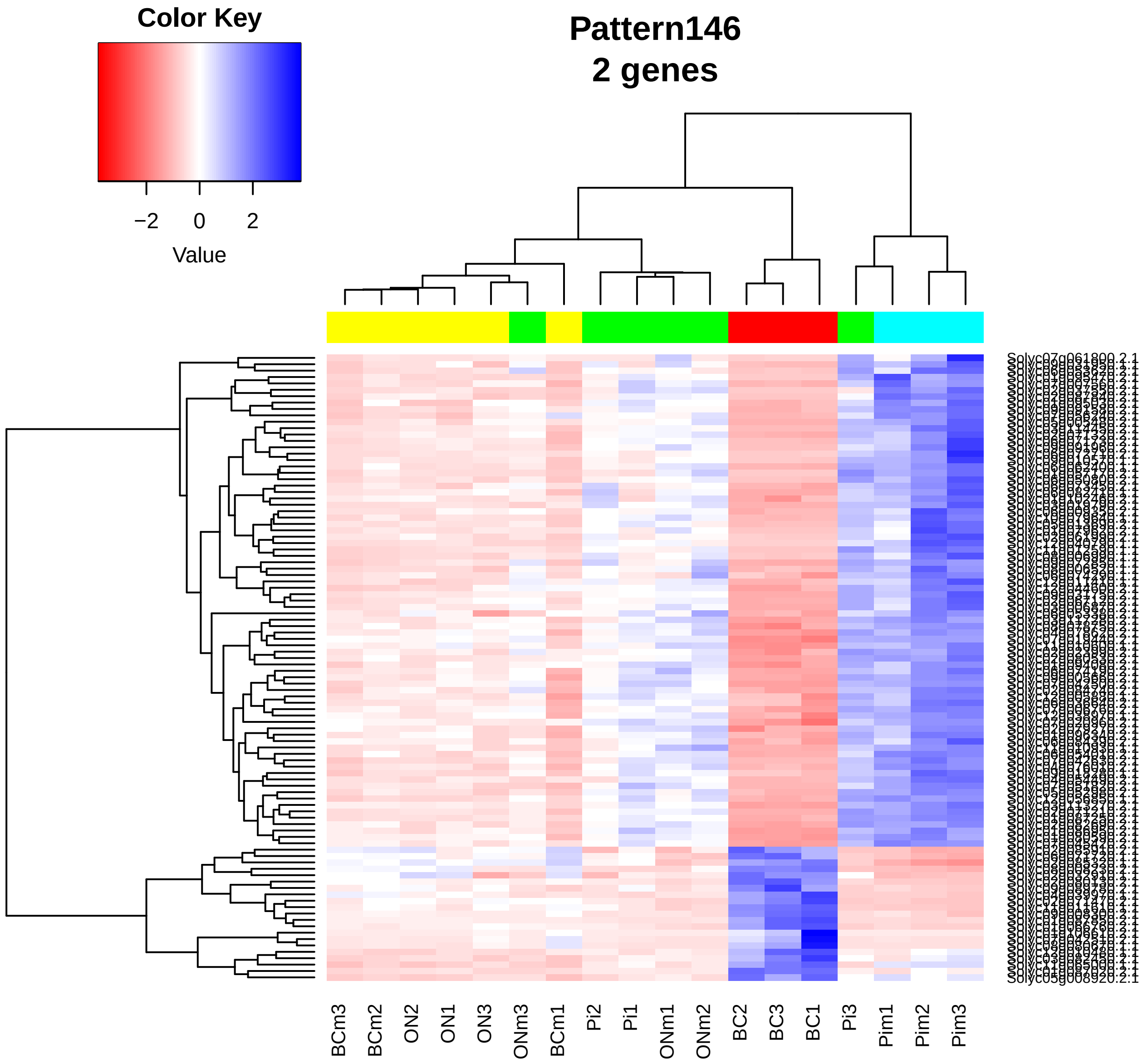
Color Key
−2
0
2
Value
Pattern146
2 genes
BCm3
BCm2
ON2
ON1
ON3
ONm3
BCm1
Pi2
Pi1
ONm1
ONm2
BC2
BC3
BC1
Pi3
Pim1
Pim2
Pim3
Solyc07g061800.2.1
Solyc05g008920.2.1

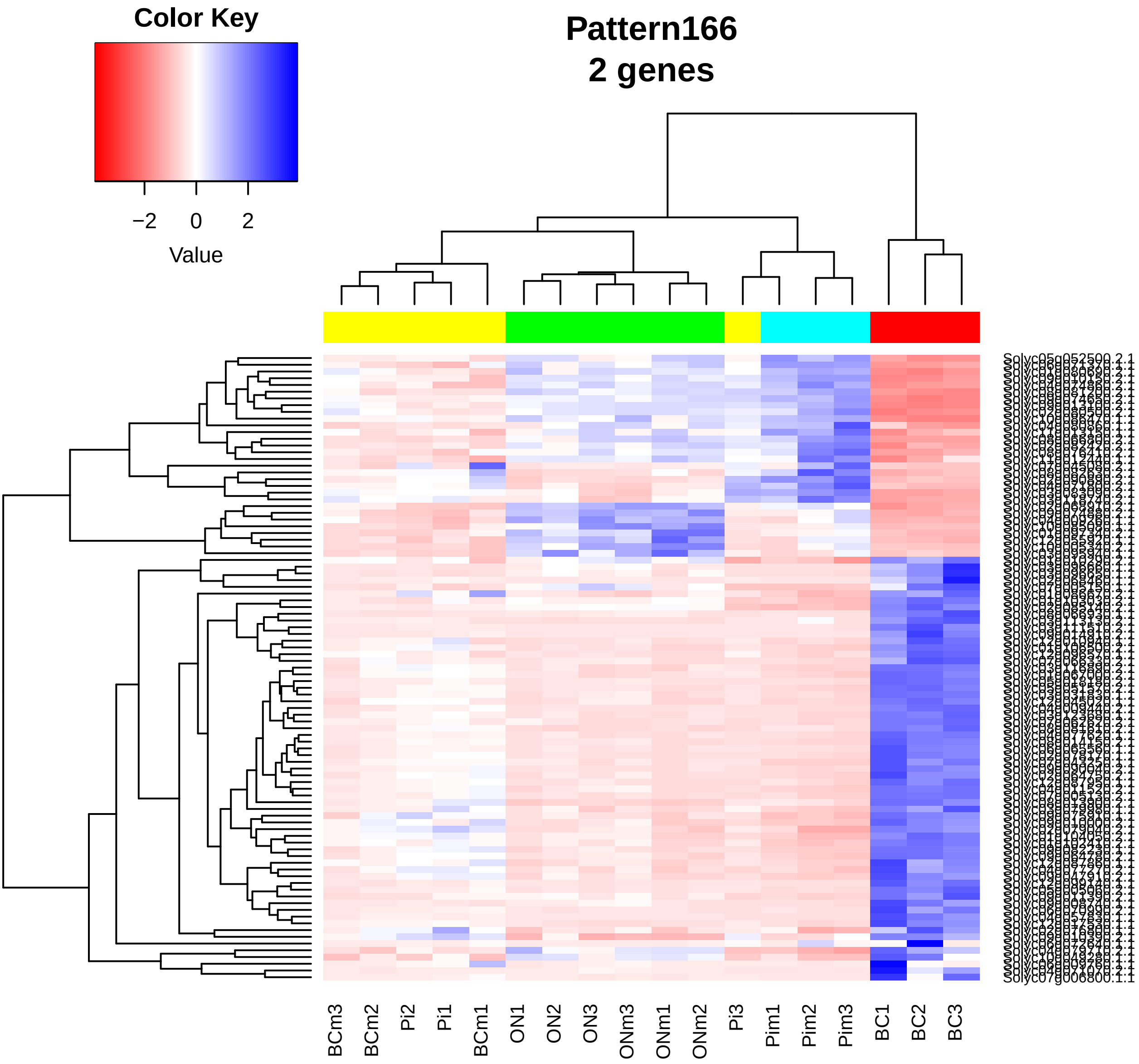
Color Key
−2
0
2
Value
Pattern166
2 genes
BCm3
BCm2
Pi2
Pi1
BCm1
ON1
ON2
ON3
ONm3
ONm1
ONm2
Pi3
Pim1
Pim2
Pim3
BC1
BC2
BC3
Solyc05g052500.2.1
Solyc07g006800.1.1

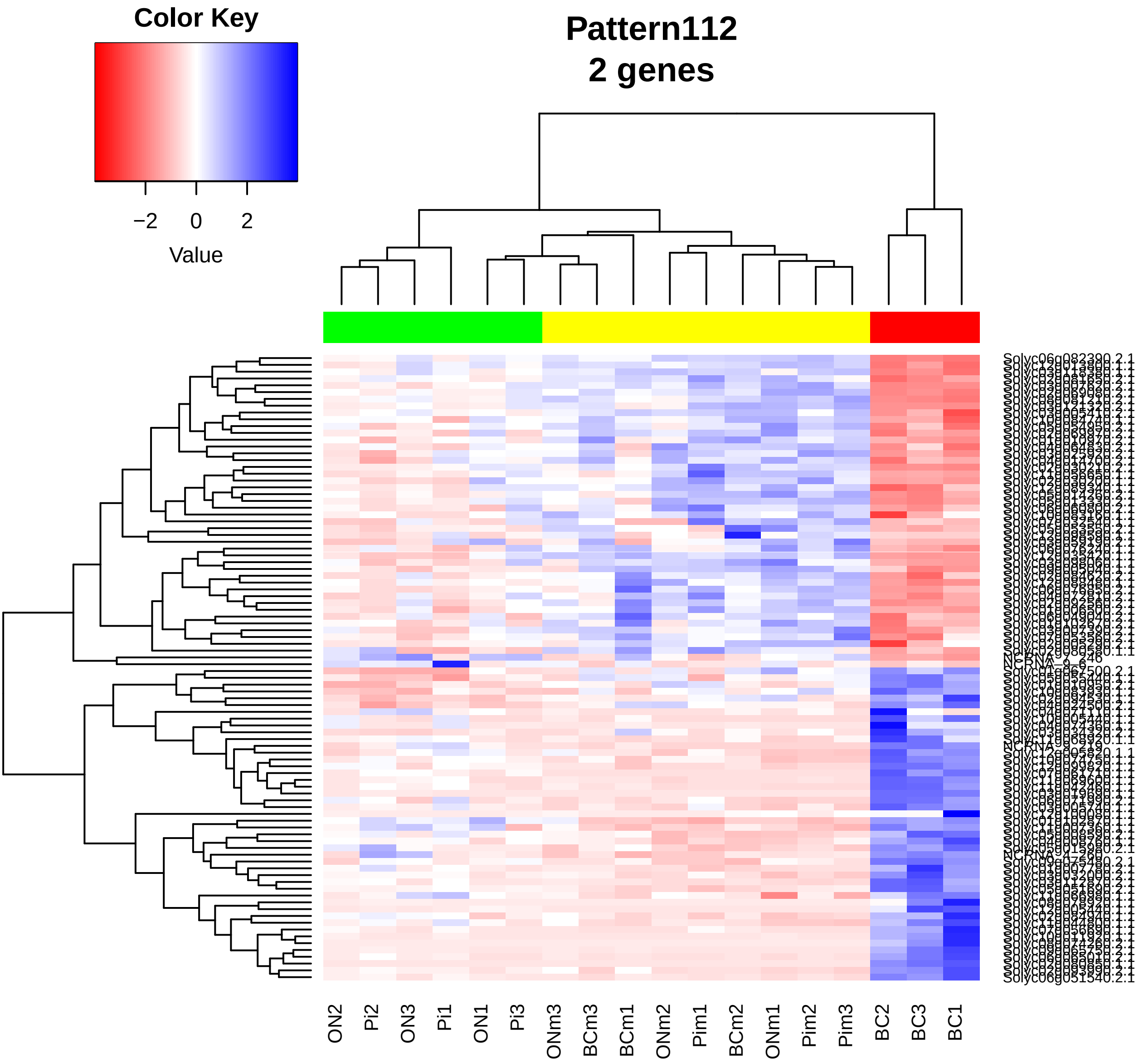
Color Key
−2
0
2
Value
Pattern112
2 genes
ON2
Pi2
ON3
Pi1
ON1
Pi3
ONm3
BCm3
BCm1
ONm2
Pim1
BCm2
ONm1
Pim2
Pim3
BC2
BC3
BC1
Solyc06g082390.2.1
Solyc06g051540.2.1

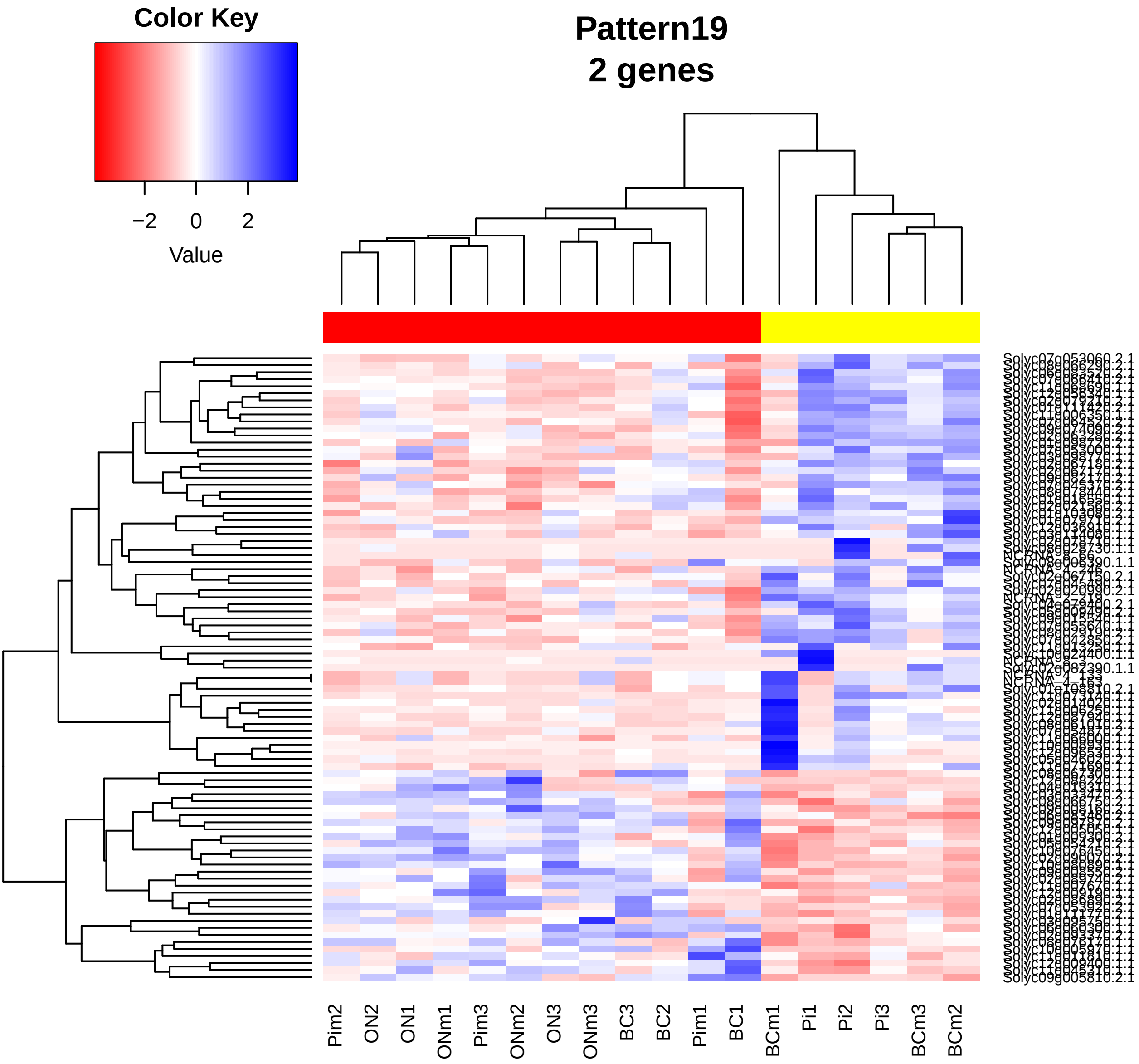
Color Key
−2
0
2
Value
Pattern19
2 genes
Pim2
ON2
ON1
ONm1
Pim3
ONm2
ON3
ONm3
BC3
BC2
Pim1
BC1
BCm1
Pi1
Pi2
Pi3
BCm3
BCm2
Solyc07g053060.2.1
Solyc09g005810.2.1

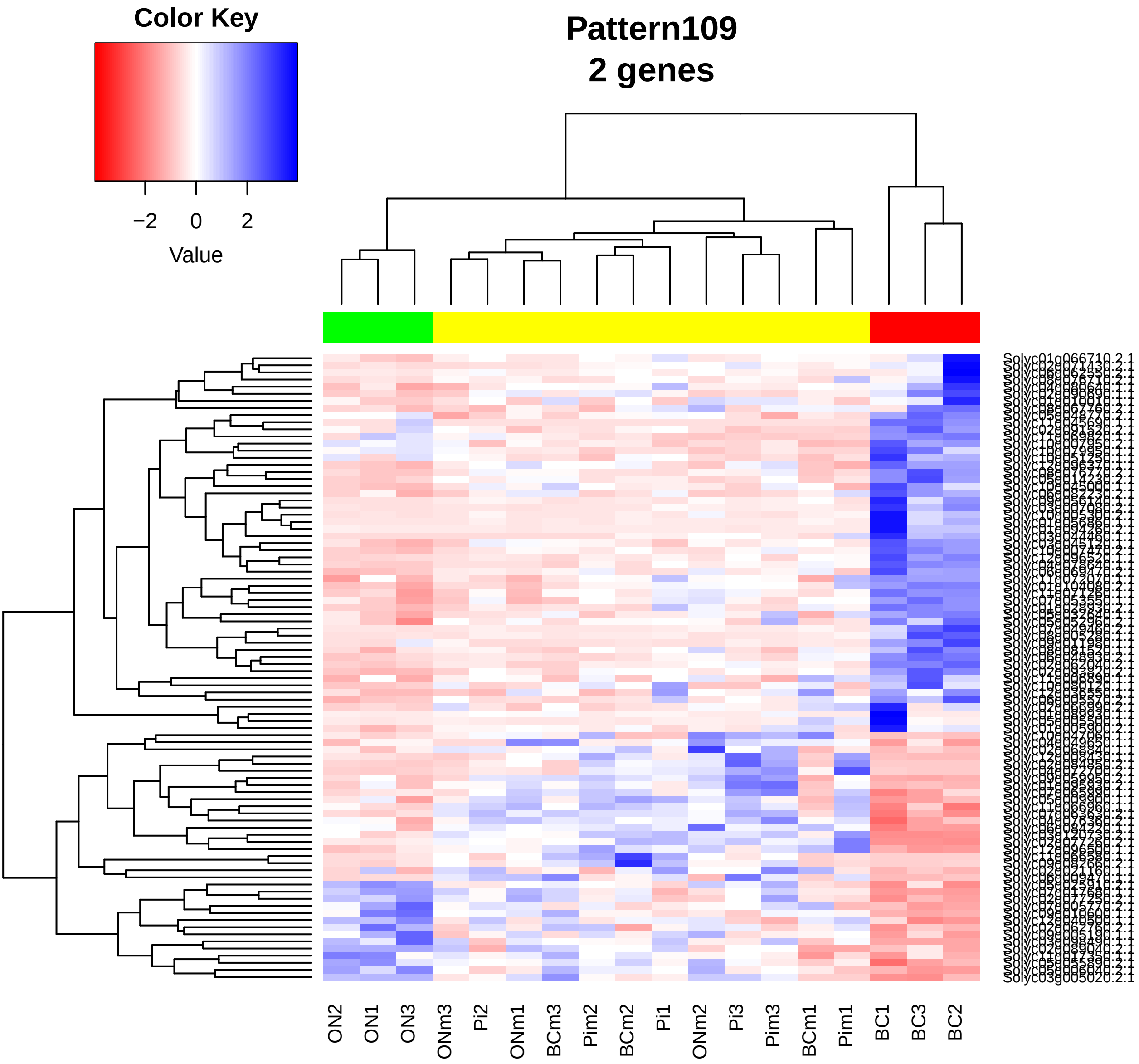
Color Key
−2
0
2
Value
Pattern109
2 genes
ON2
ON1
ON3
ONm3
Pi2
ONm1
BCm3
Pim2
BCm2
Pi1
ONm2
Pi3
Pim3
BCm1
Pim1
BC1
BC3
BC2
Solyc01g066710.2.1
Solyc03g005020.2.1

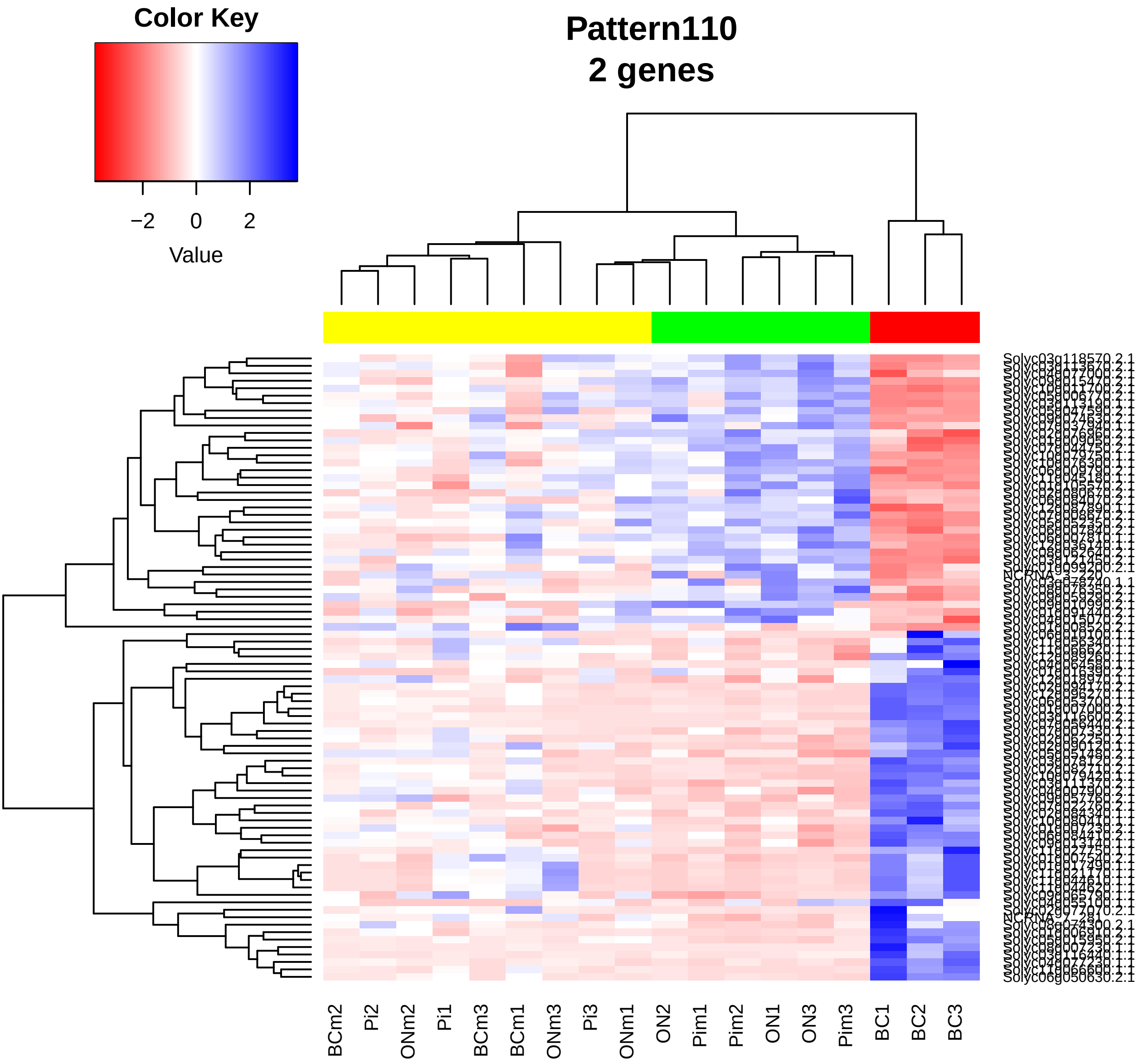
Color Key
−2
0
2
Value
Pattern110
2 genes
BCm2
Pi2
ONm2
Pi1
BCm3
BCm1
ONm3
Pi3
ONm1
ON2
Pim1
Pim2
ON1
ON3
Pim3
BC1
BC2
BC3
Solyc03g118570.2.1
Solyc06g050630.2.1

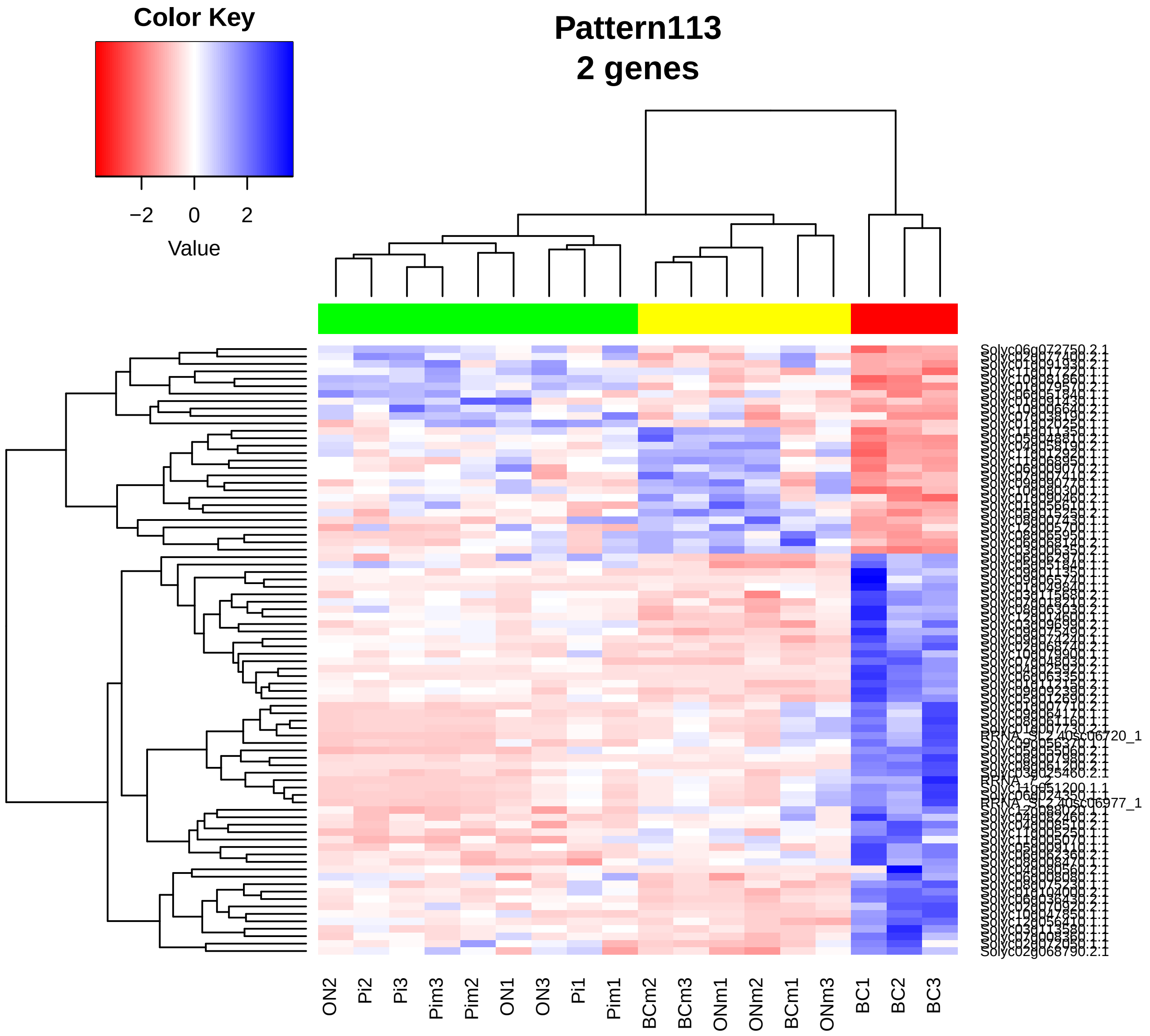

Color Key
−2
0
2
Value
Pattern113
2 genes
ON2
Pi2
Pi3
Pim3
Pim2
ON1
ON3
Pi1
Pim1
BCm2
BCm3
ONm1
ONm2
BCm1
ONm3
BC1
BC2
BC3
Solyc06g072750.2.1
RRNA_SL2.40sc06720_1
RRNA_SL2.40sc06977_1
Solyc02g068790.2.1

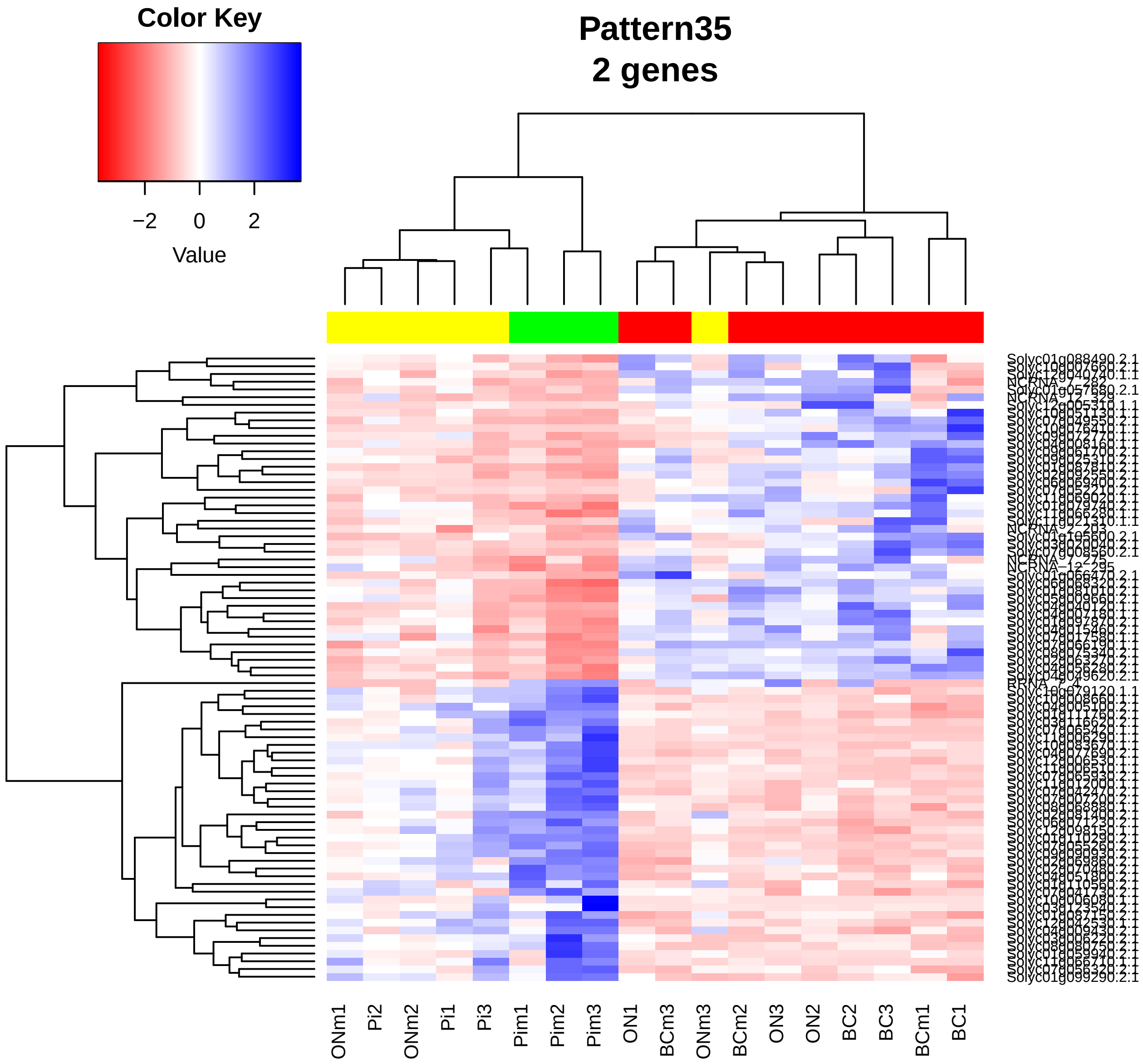
Pattern35
2 genes
Color Key
−2
0
2
Value
ONm1
Pi2
ONm2
Pi1
Pi3
Pim1
Pim2
Pim3
ON1
BCm3
ONm3
BCm2
ON3
ON2
BC2
BC3
BCm1
BC1
Solyc01g088490.2.1
Solyc01g099290.2.1

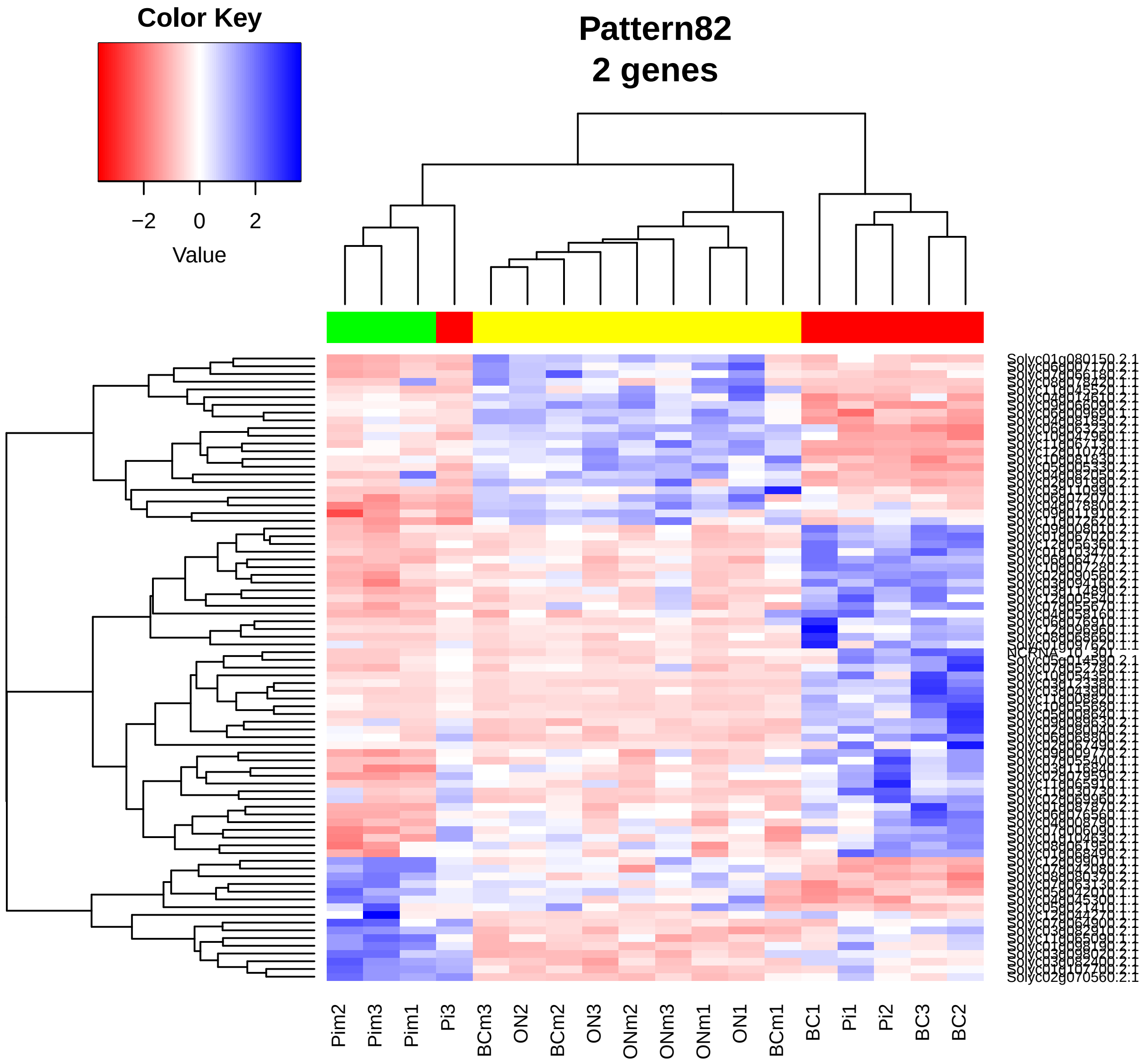
Color Key
−2
0
2
Value
Pattern82
2 genes
Pim2
Pim3
Pim1
Pi3
BCm3
ON2
BCm2
ON3
ONm2
ONm3
ONm1
ON1
BCm1
BC1
Pi1
Pi2
BC3
BC2
Solyc01g080150.2.1
Solyc02g070560.2.1

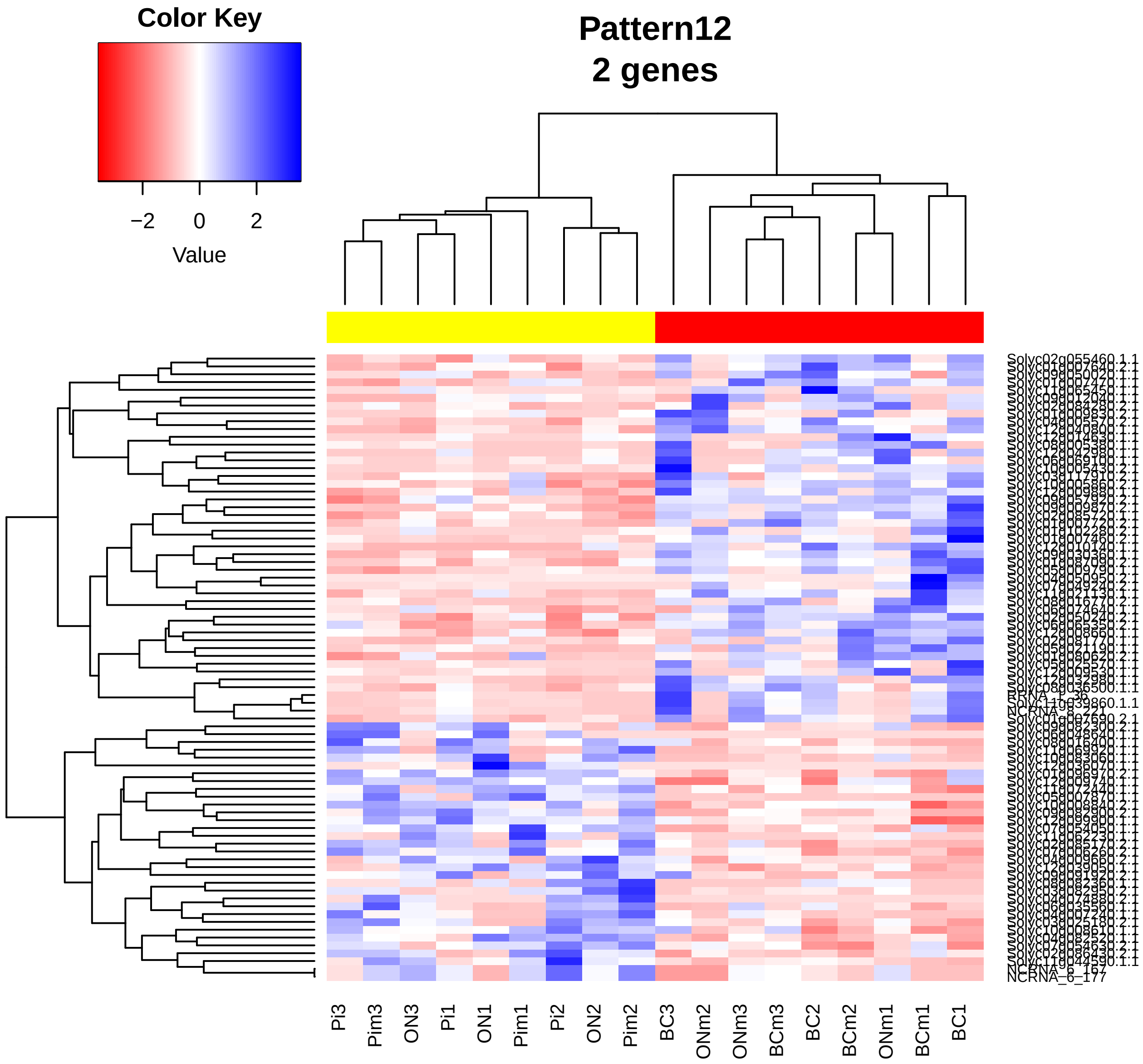

Color Key
−2
0
2
Value
Pattern12
2 genes
Solyc02g055460.1.1
NCRNA_6_177
Pi3
Pim3
ON3
Pi1
ON1
Pim1
Pi2
ON2
Pim2
BC3
ONm2
ONm3
BCm3
BC2
BCm2
ONm1
BCm1
BC1

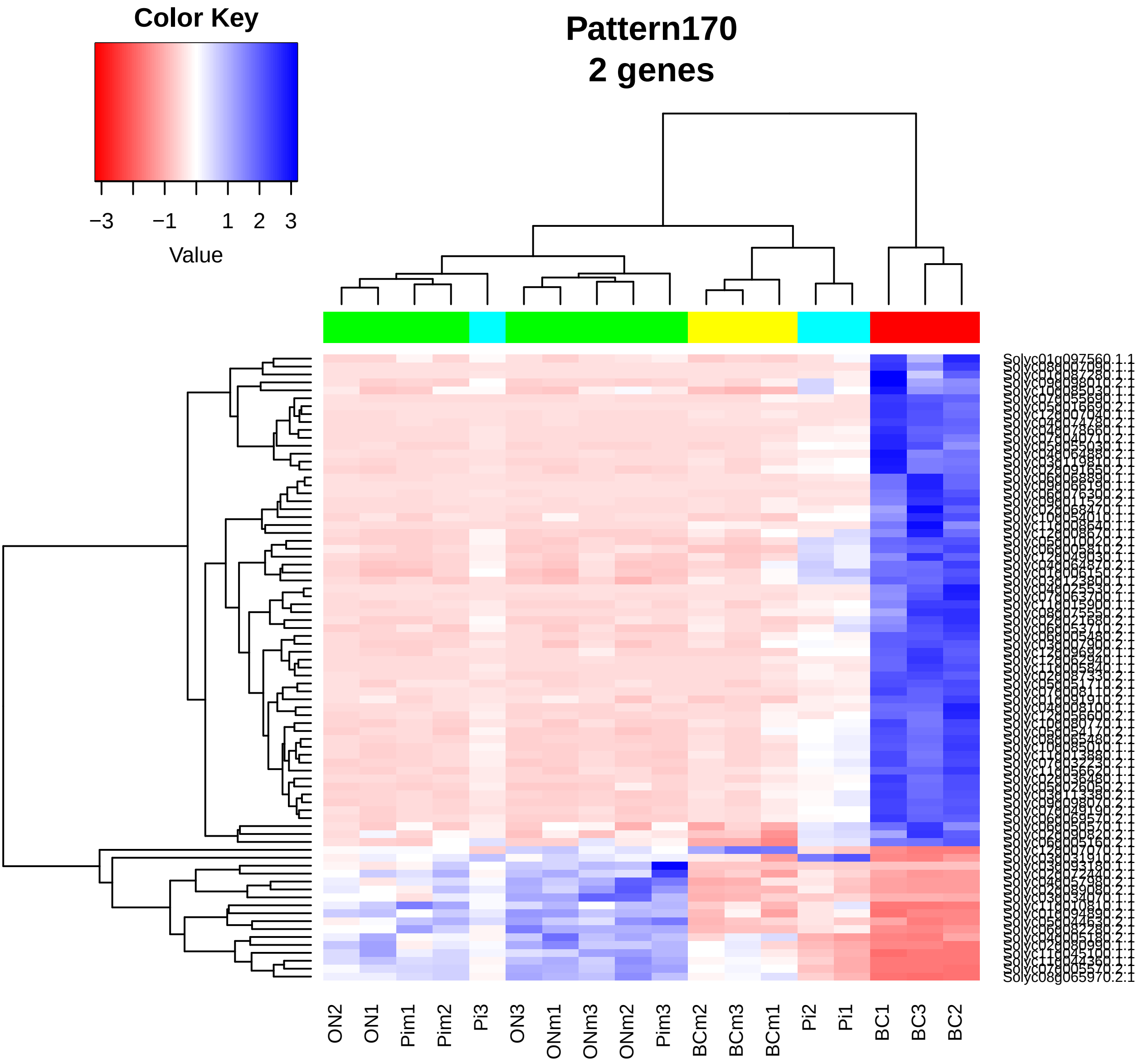
Color Key
−3
−1
1
2
3
Value
Pattern170
2 genes
ON2
ON1
Pim1
Pim2
Pi3
ON3
ONm1
ONm3
ONm2
Pim3
BCm2
BCm3
BCm1
Pi2
Pi1
BC1
BC3
BC2
Solyc01g097560.1.1
Solyc08g065970.2.1

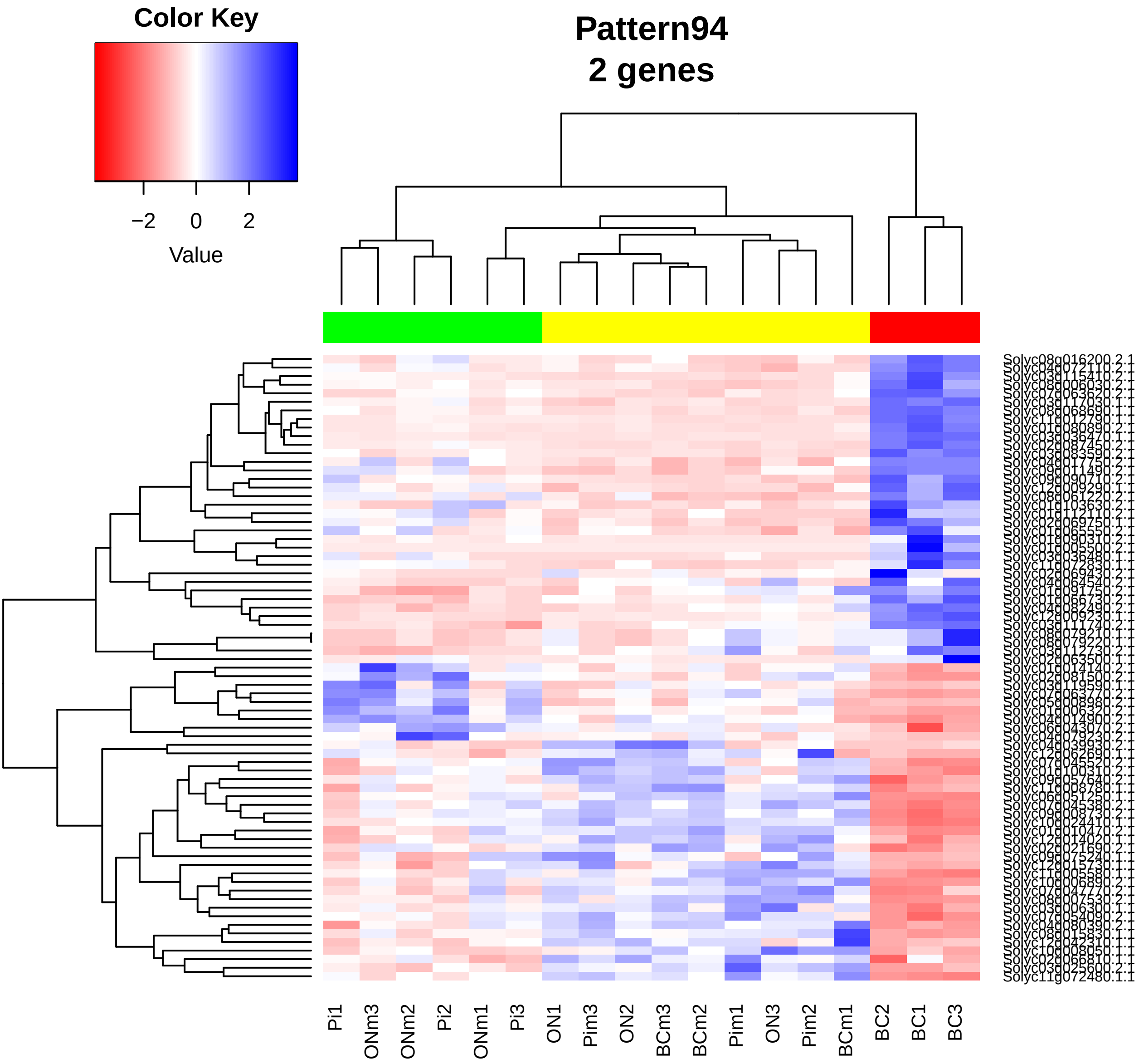
Color Key
−2
0
2
Value
Pattern94
2 genes
Pi1
ONm3
ONm2
Pi2
ONm1
Pi3
ON1
Pim3
ON2
BCm3
BCm2
Pim1
ON3
Pim2
BCm1
BC2
BC1
BC3
Solyc08g016200.2.1
Solyc11g072480.1.1

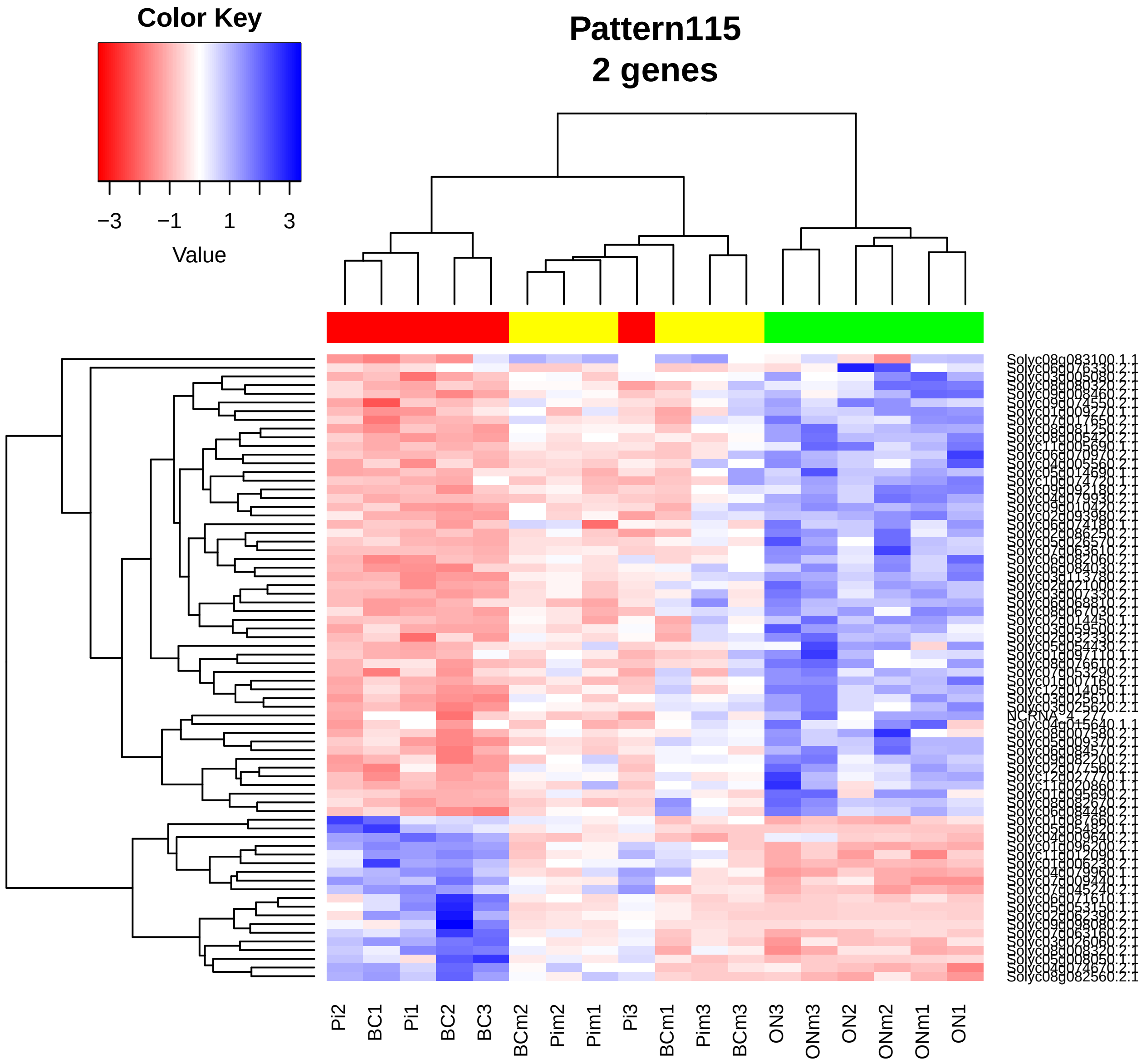
Pattern115
2 genes
Color Key
−3
−1
1
3
Value
Solyc08g083100.1.1
Solyc08g082560.2.1
Pi2
BC1
Pi1
BC2
BC3
BCm2
Pim2
Pim1
Pi3
BCm1
Pim3
BCm3
ON3
ONm3
ON2
ONm2
ONm1
ON1

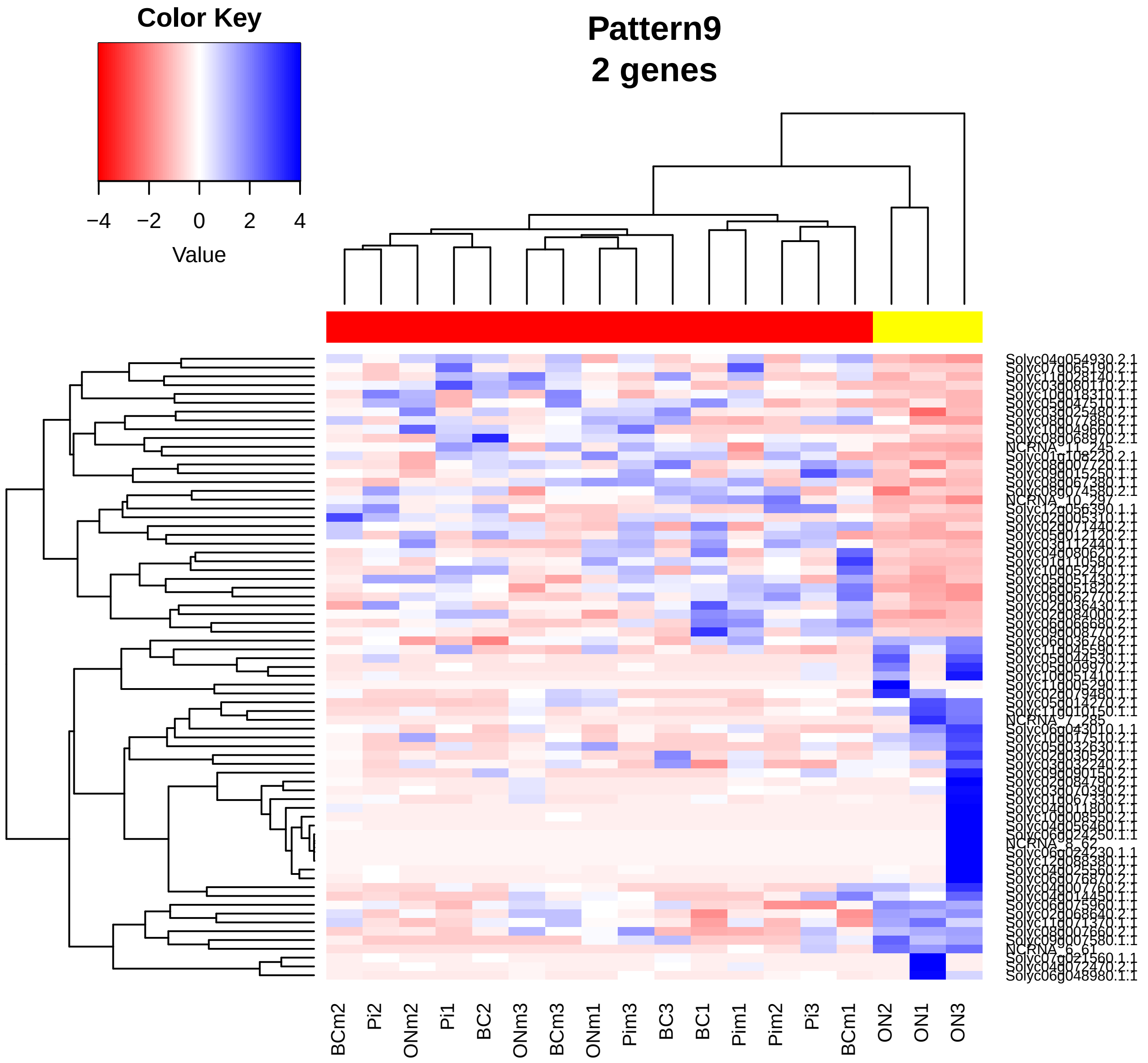
Color Key
−4 −2 0 2 4
Value
Pattern9
2 genes
BCm2
Pi2
ONm2
Pi1
BC2
ONm3
BCm3
ONm1
Pim3
BC3
BC1
Pim1
Pim2
Pi3
BCm1
ON2
ON1
ON3

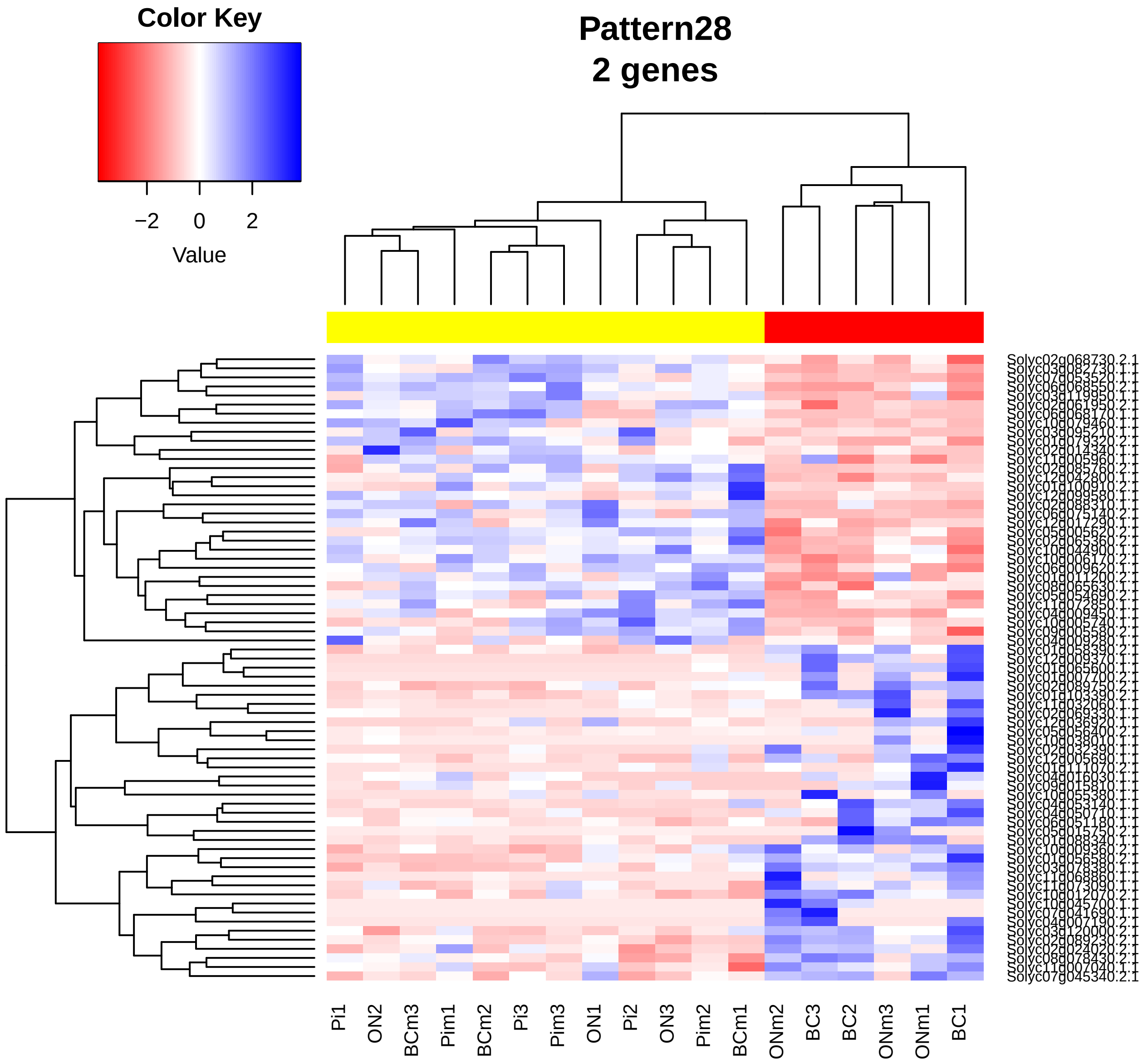
Color Key
−2
0
2
Value
Pattern28
2 genes
Solyc02g068730.2.1
Solyc07g045340.2.1
Pi1
ON2
BCm3
Pim1
BCm2
Pi3
Pim3
ON1
Pi2
ON3
Pim2
BCm1
ONm2
BC3
BC2
ONm3
ONm1
BC1

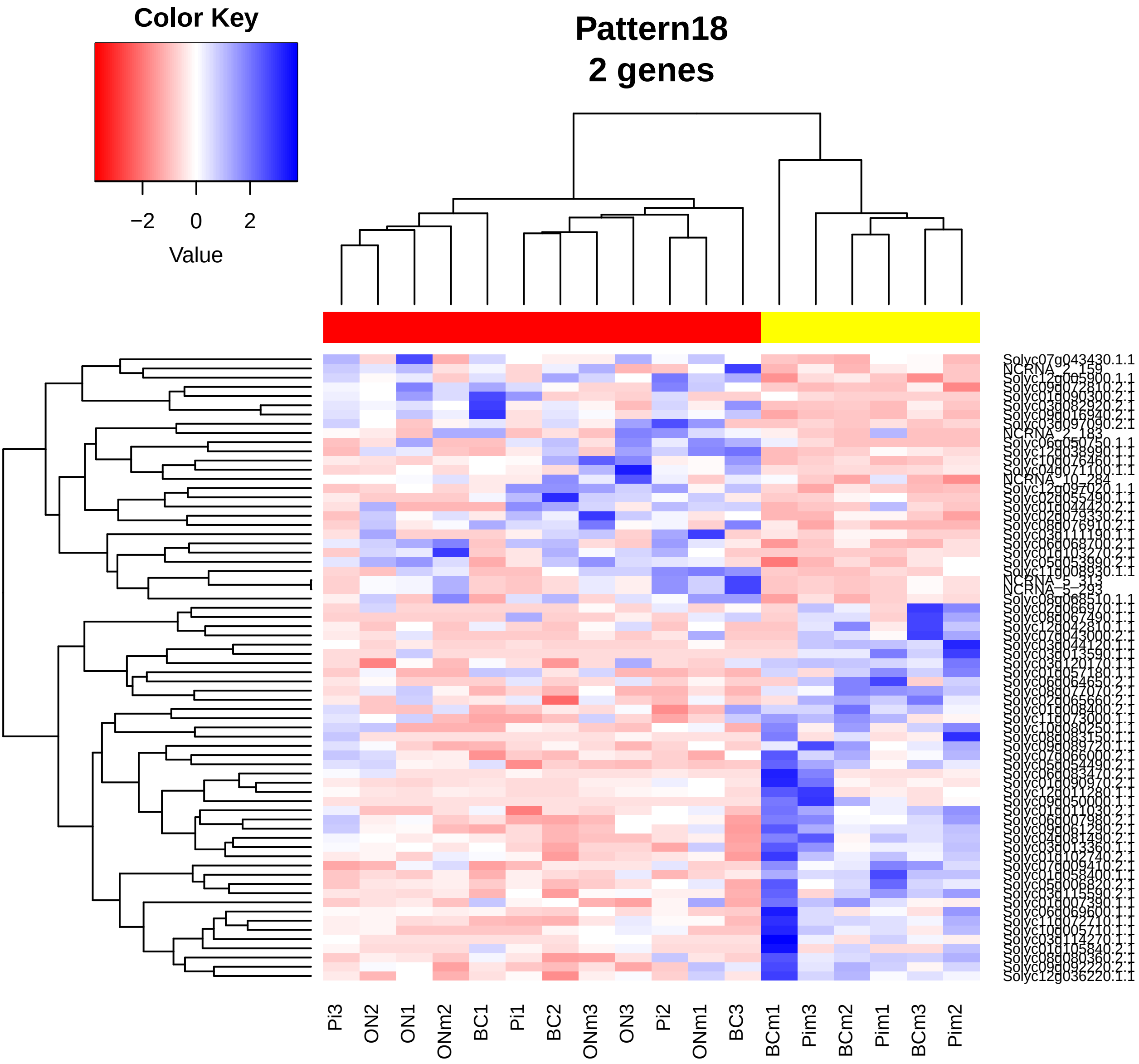
Color Key
−2
0
2
Value
Pattern18
2 genes
Pi3
ON2
ON1
ONm2
BC1
Pi1
BC2
ONm3
ON3
Pi2
ONm1
BC3
BCm1
Pim3
BCm2
Pim1
BCm3
Pim2
Solyc07g043430.1.1
NCRNA_2_159
Solyc12g005900.1.1
Solyc09g072810.2.1
Solyc01g090300.2.1
Solyc03g082920.2.1
Solyc09g016940.2.1
Solyc03g097090.2.1
NCRNA_2_183
Solyc06g050750.1.1
Solyc12g038990.1.1
Solyc10g076460.1.1
Solyc04g071100.1.1
NCRNA_10_284
Solyc12g097020.1.1
Solyc02g055490.1.1
Solyc01g044420.2.1
Solyc02g079330.2.1
Solyc08g076910.2.1
Solyc03g111190.1.1
Solyc06g068700.2.1
Solyc01g103270.2.1
Solyc05g053990.2.1
Solyc11g008930.1.1
NCRNA_5_313
NCRNA_5_293
Solyc08g068510.1.1
Solyc02g066970.1.1
Solyc08g067490.1.1
Solyc12g042810.1.1
Solyc07g043000.2.1
Solyc03g044120.1.1
Solyc03g013590.1.1
Solyc03g120170.1.1
Solyc01g057180.1.1
Solyc06g064650.2.1
Solyc08g077070.2.1
Solyc02g065660.2.1
Solyc01g008400.2.1
Solyc11g073000.1.1
Solyc10g080250.1.1
Solyc08g083150.1.1
Solyc09g089720.1.1
Solyc07g066000.2.1
Solyc05g054490.2.1
Solyc06g083470.2.1
Solyc01g090970.2.1
Solyc12g011280.1.1
Solyc09g050000.1.1
Solyc01g011030.2.1
Solyc06g007980.2.1
Solyc09g061290.2.1
Solyc04g081490.2.1
Solyc03g013360.1.1
Solyc01g102740.2.1
Solyc07g009410.2.1
Solyc01g058400.1.1
Solyc05g006820.2.1
Solyc03g115590.2.1
Solyc01g007390.1.1
Solyc06g069600.1.1
Solyc11g072710.1.1
Solyc10g005710.1.1
Solyc03g114270.1.1
Solyc01g105840.2.1
Solyc08g080360.2.1
Solyc09g092220.2.1
Solyc12g036220.1.1

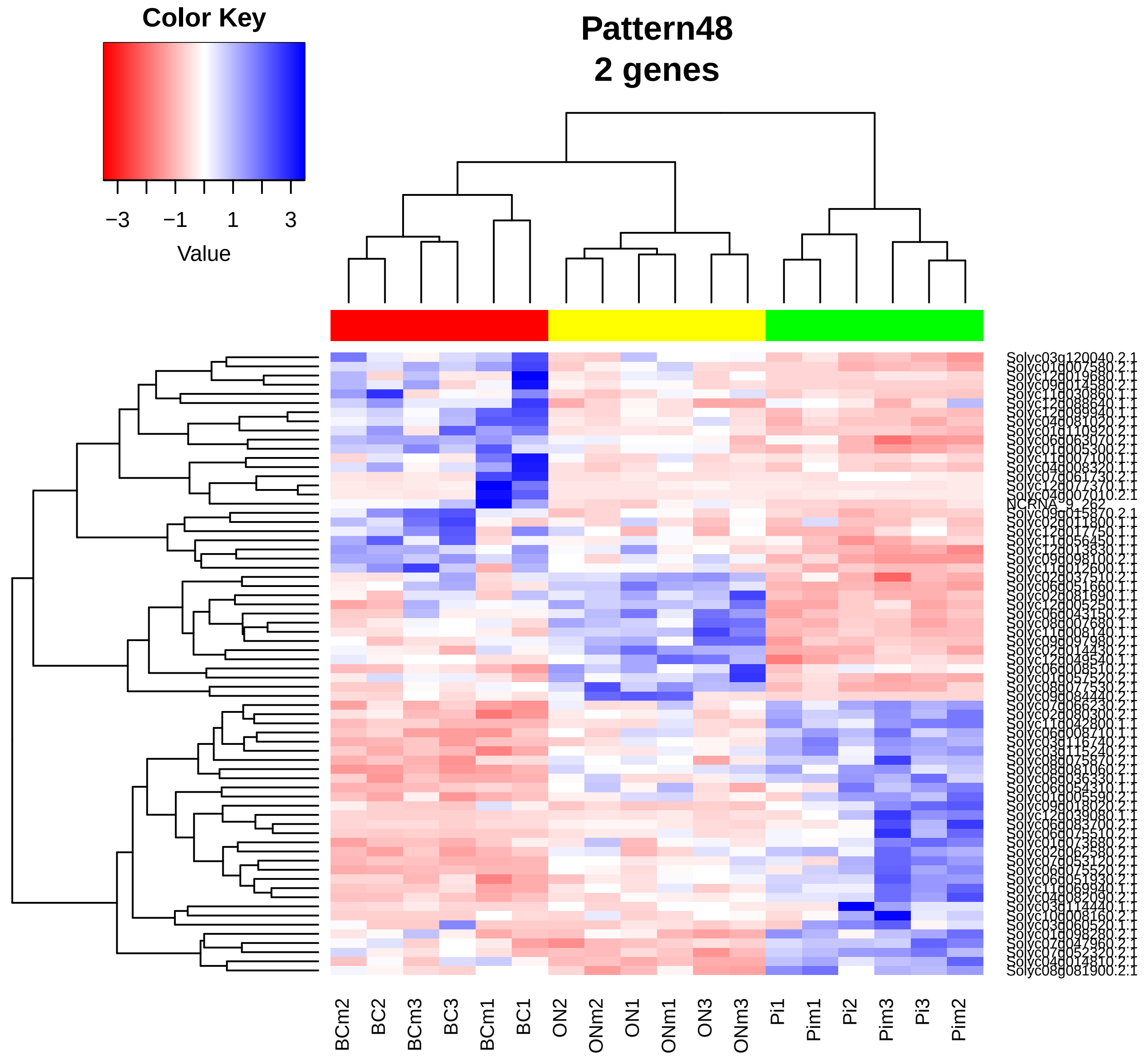
Color Key
−3
−1
1
3
Value
Pattern48
2 genes
BCm2
BC2
BCm3
BC3
BCm1
BC1
ON2
ONm2
ON1
ONm1
ON3
ONm3
Pi1
Pim1
Pi2
Pim3
Pi3
Pim2
Solyc03g120040.2.1
Solyc08g081900.2.1

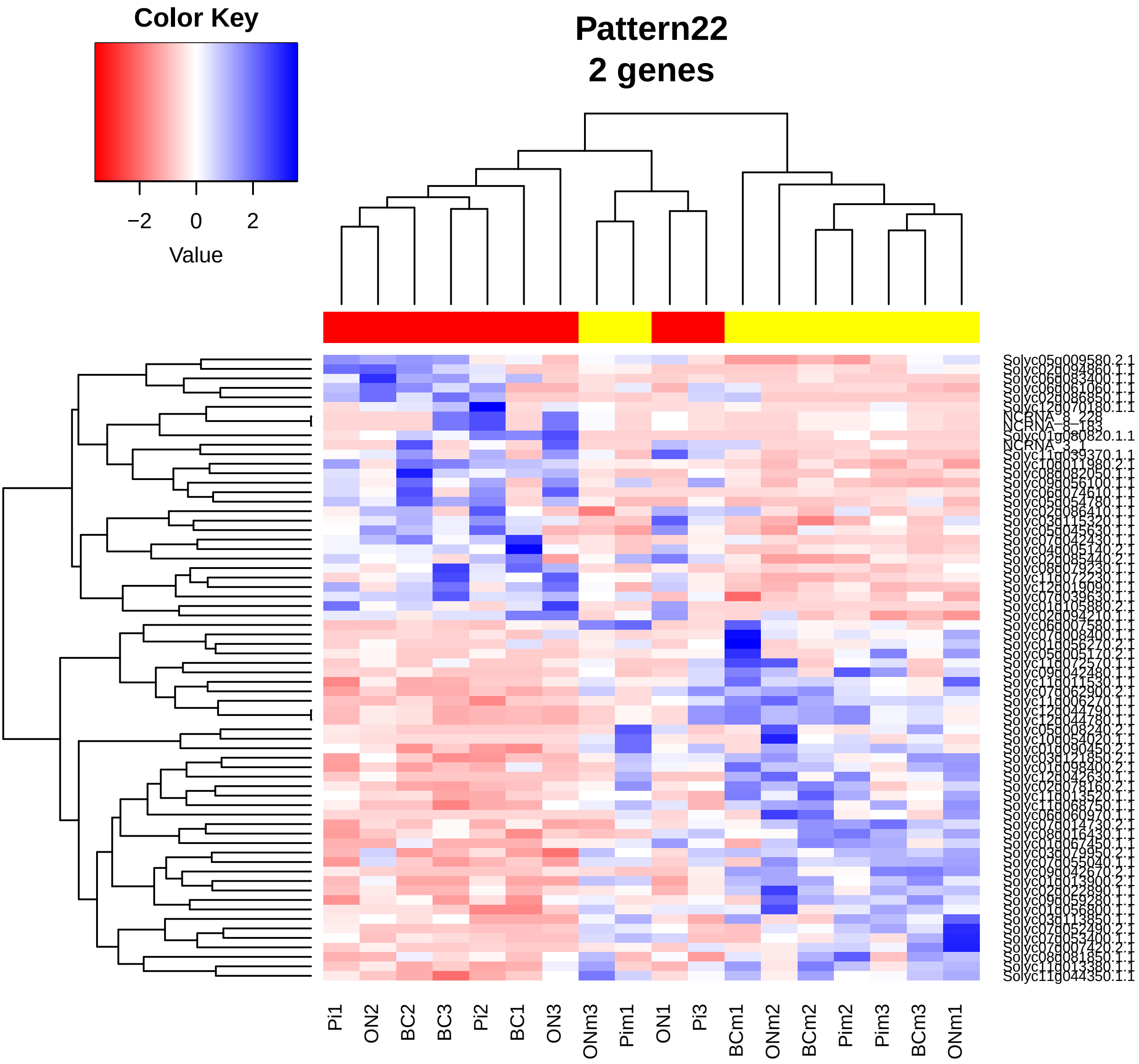

Color Key
−2
0
2
Value
Pattern22
2 genes
Solyc05g009580.2.1
Solyc11g044350.1.1
Pi1
ON2
BC2
BC3
Pi2
BC1
ON3
ONm3
Pim1
ON1
Pi3
BCm1
ONm2
BCm2
Pim2
Pim3
BCm3
ONm1

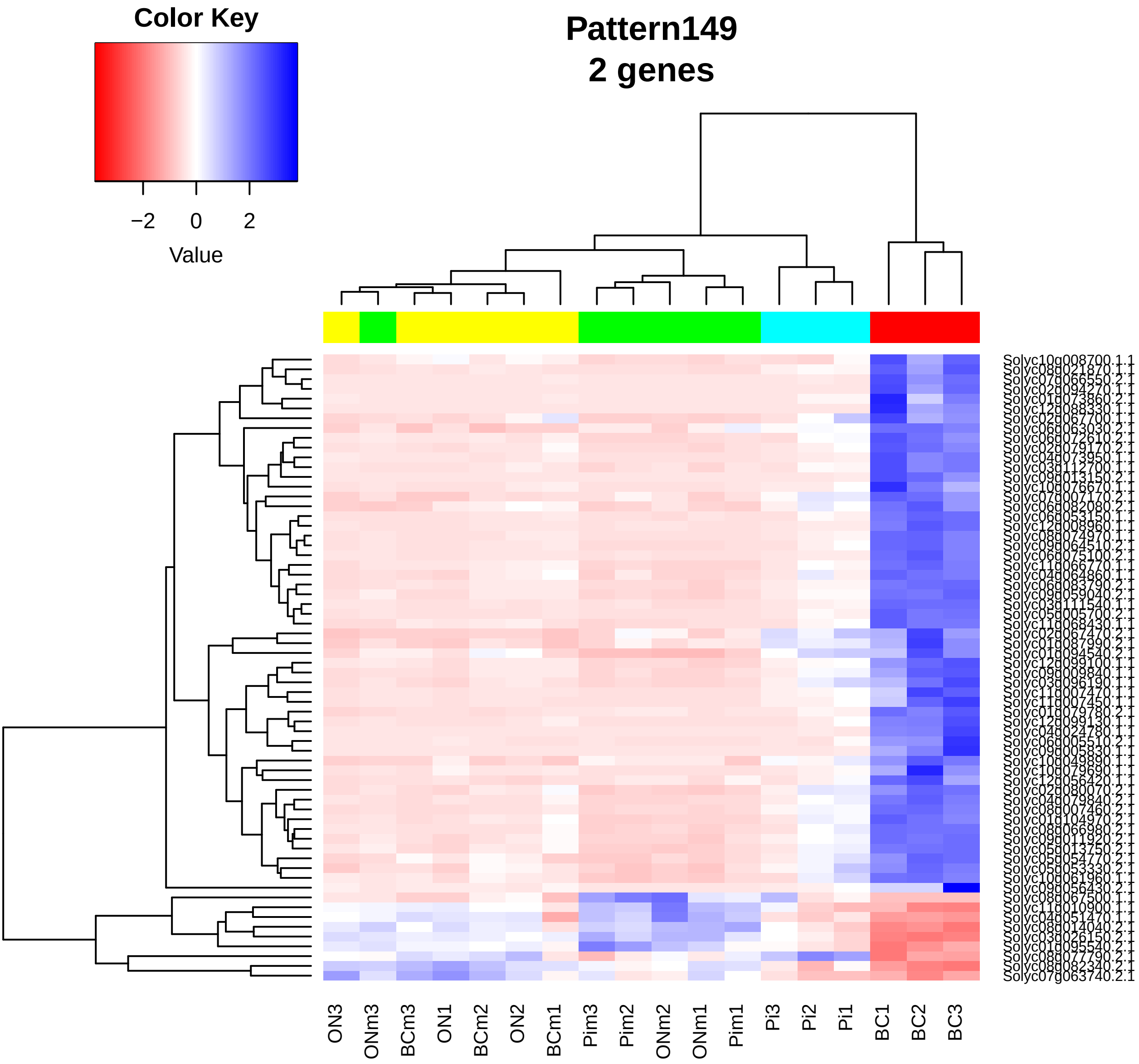
Color Key
−2
0
2
Value
Pattern149
2 genes
ON3
ONm3
BCm3
ON1
BCm2
ON2
BCm1
Pim3
Pim2
ONm2
ONm1
Pim1
Pi3
Pi2
Pi1
BC1
BC2
BC3

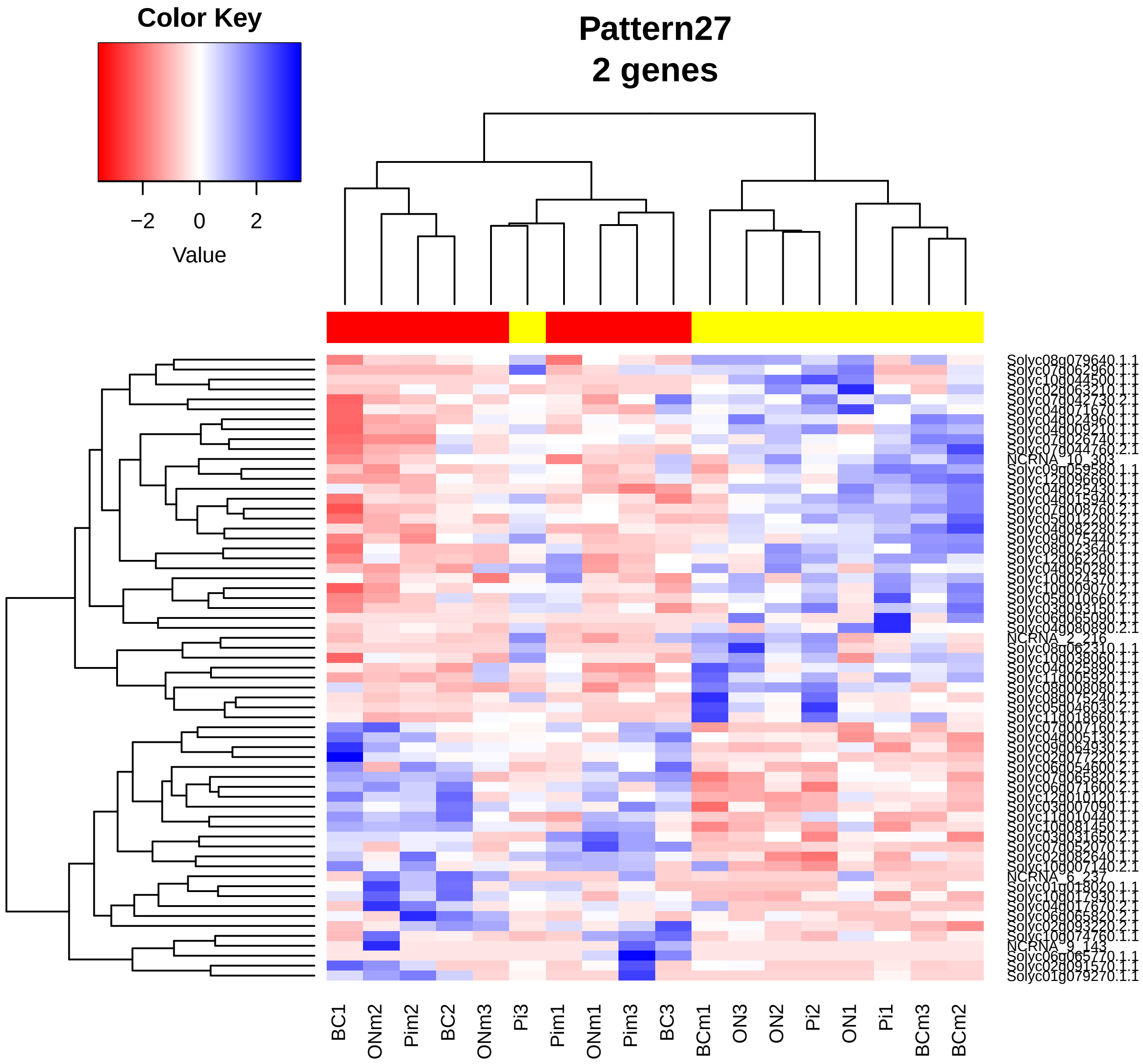

Color Key
−2
0
2
Value
Pattern27
2 genes
BC1
ONm2
Pim2
BC2
ONm3
Pi3
Pim1
ONm1
Pim3
BC3
BCm1
ON3
ON2
Pi2
ON1
Pi1
BCm3
BCm2
Solyc08g079640.1.1
Solyc07g062960.1.1
Solyc10g044500.1.1
Solyc02g063210.1.1
Solyc07g042730.2.1
Solyc04g071670.1.1
Solyc04g024960.1.1
Solyc04g009210.1.1
Solyc07g026740.1.1
Solyc07g044760.2.1
NCRNA_10_303
Solyc09g059580.1.1
Solyc12g096660.1.1
Solyc04g025430.1.1
Solyc04g015940.2.1
Solyc07g008760.2.1
Solyc05g012200.2.1
Solyc04g082280.2.1
Solyc09g075440.2.1
Solyc08g023640.1.1
Solyc12g062200.1.1
Solyc04g050280.1.1
Solyc10g024370.1.1
Solyc10g009070.2.1
Solyc05g010660.2.1
Solyc03g093150.1.1
Solyc06g065090.1.1
Solyc04g080890.2.1
NCRNA_2_216
Solyc08g062310.1.1
Solyc10g038060.1.1
Solyc04g025890.1.1
Solyc11g005920.1.1
Solyc08g008080.1.1
Solyc08g075240.2.1
Solyc05g046030.2.1
Solyc11g018660.1.1
Solyc07g007160.2.1
Solyc04g005130.2.1
Solyc09g064930.2.1
Solyc02g077220.2.1
Solyc06g054600.2.1
Solyc07g065820.2.1
Solyc06g071600.2.1
Solyc12g010120.1.1
Solyc03g007090.1.1
Solyc11g010440.1.1
Solyc10g081450.1.1
Solyc03g031650.2.1
Solyc07g052070.1.1
Solyc02g082640.1.1
Solyc10g007140.2.1
NCRNA_6_237
Solyc01g018020.1.1
Solyc10g017930.1.1
Solyc04g017670.2.1
Solyc06g065820.2.1
Solyc02g093220.2.1
Solyc10g074760.1.1
NCRNA_9_143
Solyc06g065770.1.1
Solyc02g091570.1.1
Solyc01g079270.1.1

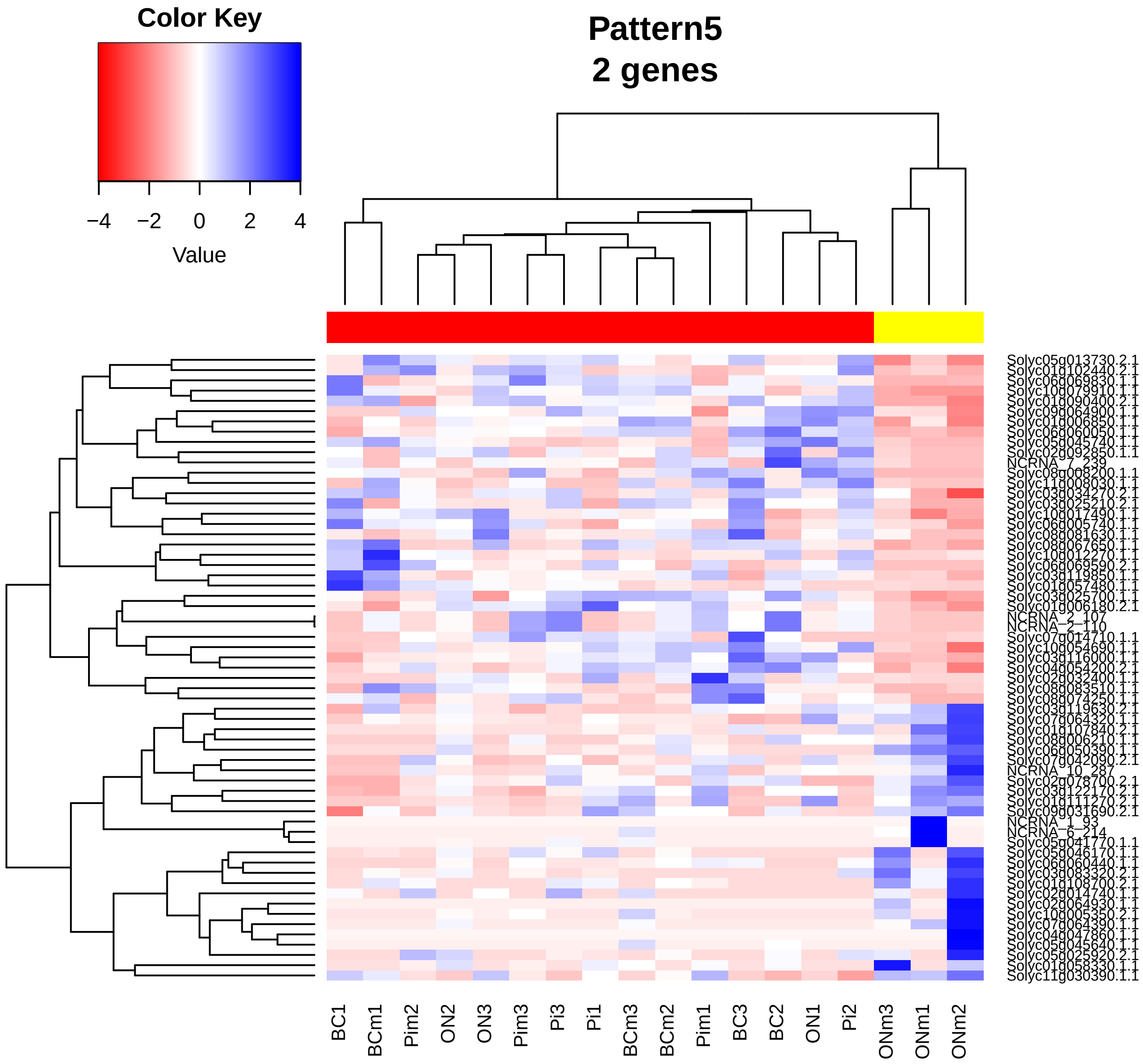

Color Key
−4
−2
0
2
4
Value
Pattern5
2 genes
Solyc05g013730.2.1
Solyc01g102440.2.1
Solyc06g069830.1.1
Solyc10g079910.1.1
Solyc01g090400.2.1
Solyc09g064900.1.1
Solyc01g006850.1.1
Solyc06g060050.1.1
Solyc05g045740.1.1
Solyc02g092850.1.1
NCRNA_7_239
Solyc08g008200.1.1
Solyc11g008030.1.1
Solyc03g034270.2.1
Solyc03g025210.2.1
Solyc10g017490.1.1
Solyc06g005740.1.1
Solyc08g081630.1.1
Solyc08g067650.1.1
Solyc10g012270.1.1
Solyc06g069590.2.1
Solyc03g119850.1.1
Solyc01g057480.1.1
Solyc03g025700.1.1
Solyc01g006180.2.1
NCRNA_2_107
NCRNA_2_110
Solyc07g014710.1.1
Solyc10g054690.1.1
Solyc03g116000.1.1
Solyc04g054200.2.1
Solyc02g032400.1.1
Solyc08g083510.1.1
Solyc08g074250.1.1
Solyc03g119630.2.1
Solyc07g064320.1.1
Solyc01g107840.2.1
Solyc08g006210.1.1
Solyc06g050390.1.1
Solyc07g042090.2.1
NCRNA_10_287
Solyc02g078700.2.1
Solyc03g122170.2.1
Solyc01g111270.2.1
Solyc09g031690.2.1
NCRNA_1_93
NCRNA_6_214
Solyc05g041770.1.1
Solyc05g046170.1.1
Solyc06g060440.1.1
Solyc03g083320.2.1
Solyc01g108700.2.1
Solyc02g014740.1.1
Solyc02g064930.1.1
Solyc10g005350.2.1
Solyc07g064390.1.1
Solyc04g047860.1.1
Solyc05g045640.1.1
Solyc05g025920.2.1
Solyc01g058330.1.1
Solyc11g030390.1.1
BC1
BCm1
Pim2
ON2
ON3
Pim3
Pi3
Pi1
BCm3
BCm2
Pim1
BC3
BC2
ON1
Pi2
ONm3
ONm1
ONm2

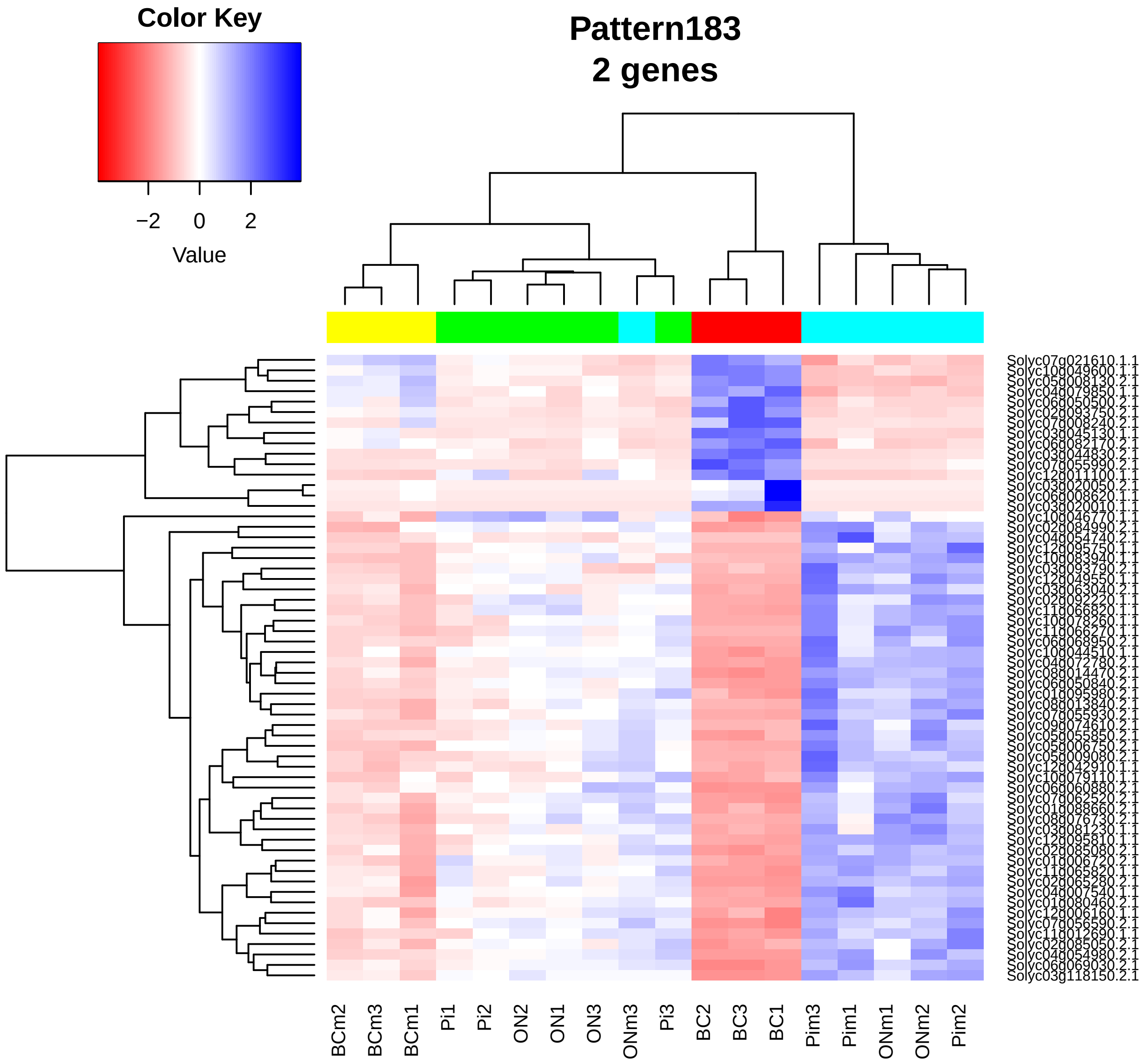

Color Key
−2
0
2
Value
Pattern183
2 genes
Solyc07g021610.1.1
Solyc10g049600.1.1
Solyc05g008130.2.1
Solyc04g079850.1.1
Solyc06g050500.2.1
Solyc02g093750.2.1
Solyc07g008240.2.1
Solyc03g045130.1.1
Solyc06g082170.2.1
Solyc03g044830.2.1
Solyc07g055990.2.1
Solyc12g011100.1.1
Solyc03g020050.2.1
Solyc06g008620.1.1
Solyc03g020010.1.1
Solyc10g046770.1.1
Solyc02g084990.2.1
Solyc04g054740.2.1
Solyc12g095750.1.1
Solyc10g083940.1.1
Solyc03g093790.2.1
Solyc12g049550.1.1
Solyc03g063040.2.1
Solyc02g092220.1.1
Solyc11g066820.1.1
Solyc10g078260.1.1
Solyc11g066270.1.1
Solyc06g068950.2.1
Solyc10g044510.1.1
Solyc04g072780.2.1
Solyc08g014470.2.1
Solyc06g050840.2.1
Solyc01g095980.2.1
Solyc08g013840.2.1
Solyc07g055930.2.1
Solyc09g074610.2.1
Solyc05g055850.2.1
Solyc05g006750.2.1
Solyc05g009080.2.1
Solyc12g042910.1.1
Solyc10g079110.1.1
Solyc06g060880.2.1
Solyc07g062520.2.1
Solyc01g088660.2.1
Solyc08g076730.2.1
Solyc03g081230.1.1
Solyc12g095810.1.1
Solyc02g085080.2.1
Solyc01g006720.2.1
Solyc11g065820.1.1
Solyc02g065280.2.1
Solyc04g007540.1.1
Solyc01g080460.2.1
Solyc12g006160.1.1
Solyc07g056590.2.1
Solyc11g012690.1.1
Solyc02g085050.2.1
Solyc04g054980.2.1
Solyc06g069030.2.1
Solyc03g118150.2.1
BCm2
BCm3
BCm1
Pi1
Pi2
ON2
ON1
ON3
ONm3
Pi3
BC2
BC3
BC1
Pim3
Pim1
ONm1
ONm2
Pim2

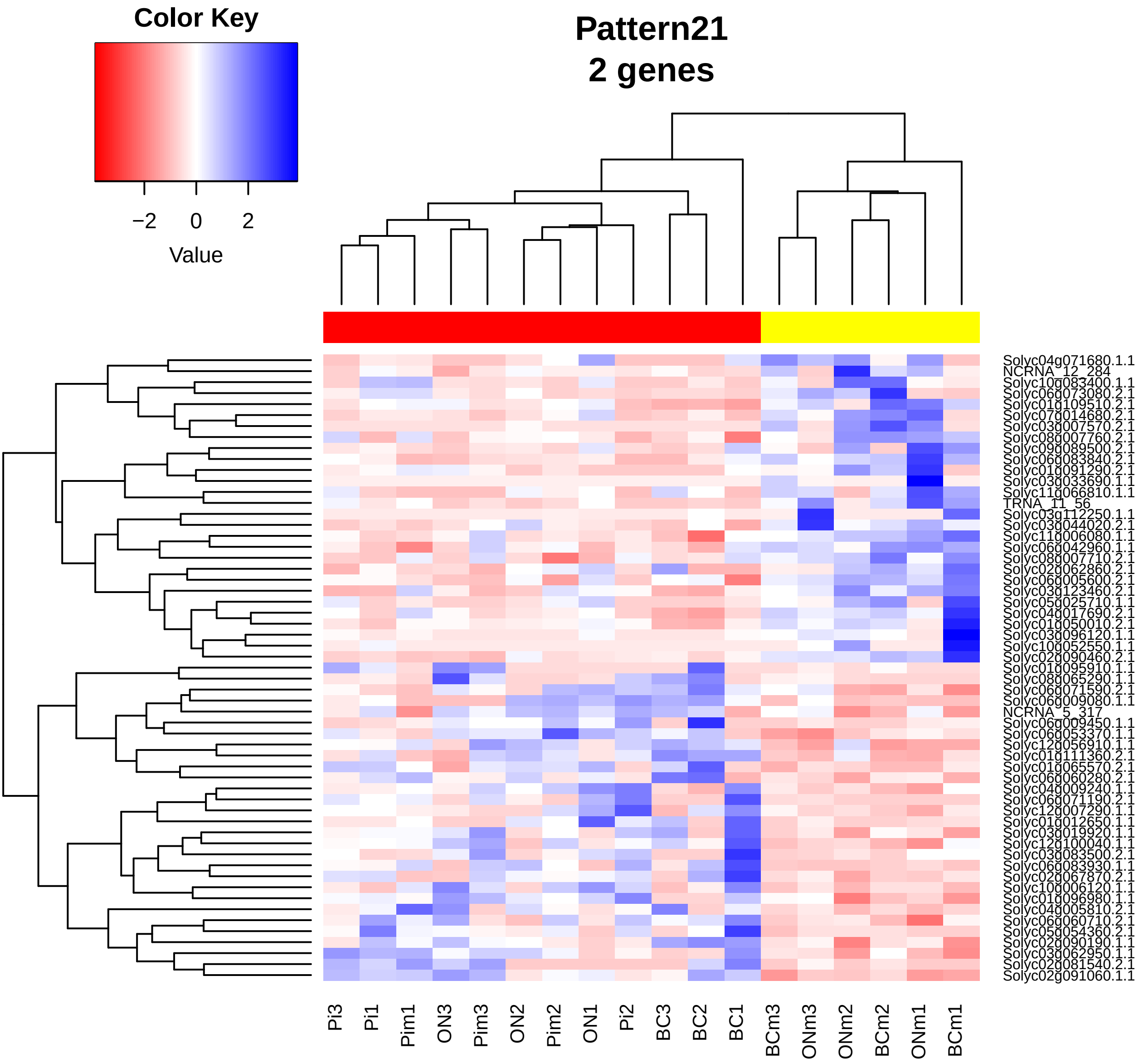
Color Key
−2
0
2
Value
Pattern21
2 genes
Solyc04g071680.1.1
NCRNA_12_284
Solyc10g083400.1.1
Solyc06g073080.2.1
Solyc01g109510.2.1
Solyc07g014680.2.1
Solyc03g007570.2.1
Solyc08g007760.2.1
Solyc09g089500.2.1
Solyc06g083840.2.1
Solyc01g091290.2.1
Solyc03g033690.1.1
Solyc11g066810.1.1
TRNA_11_56
Solyc03g112250.1.1
Solyc03g044020.2.1
Solyc11g006080.1.1
Solyc06g042960.1.1
Solyc08g007710.2.1
Solyc02g062860.2.1
Solyc06g005600.2.1
Solyc03g123460.2.1
Solyc05g025710.1.1
Solyc04g017690.2.1
Solyc01g050010.2.1
Solyc03g096120.1.1
Solyc10g052550.1.1
Solyc02g090460.2.1
Solyc01g095910.1.1
Solyc08g065290.1.1
Solyc06g071590.2.1
Solyc06g009080.1.1
NCRNA_5_317
Solyc06g009450.1.1
Solyc06g053370.1.1
Solyc12g056910.1.1
Solyc01g111360.2.1
Solyc01g065570.2.1
Solyc06g060280.2.1
Solyc04g009240.1.1
Solyc06g071190.2.1
Solyc12g007290.1.1
Solyc01g012650.1.1
Solyc03g019920.1.1
Solyc12g100040.1.1
Solyc03g083500.2.1
Solyc06g083930.1.1
Solyc02g067870.2.1
Solyc10g006120.1.1
Solyc01g096980.1.1
Solyc04g005810.2.1
Solyc06g060710.2.1
Solyc05g054360.2.1
Solyc02g090190.1.1
Solyc03g062950.1.1
Solyc02g081540.2.1
Solyc02g091060.1.1
Pi3
Pi1
Pim1
ON3
Pim3
ON2
Pim2
ON1
Pi2
BC3
BC2
BC1
BCm3
ONm3
ONm2
BCm2
ONm1
BCm1

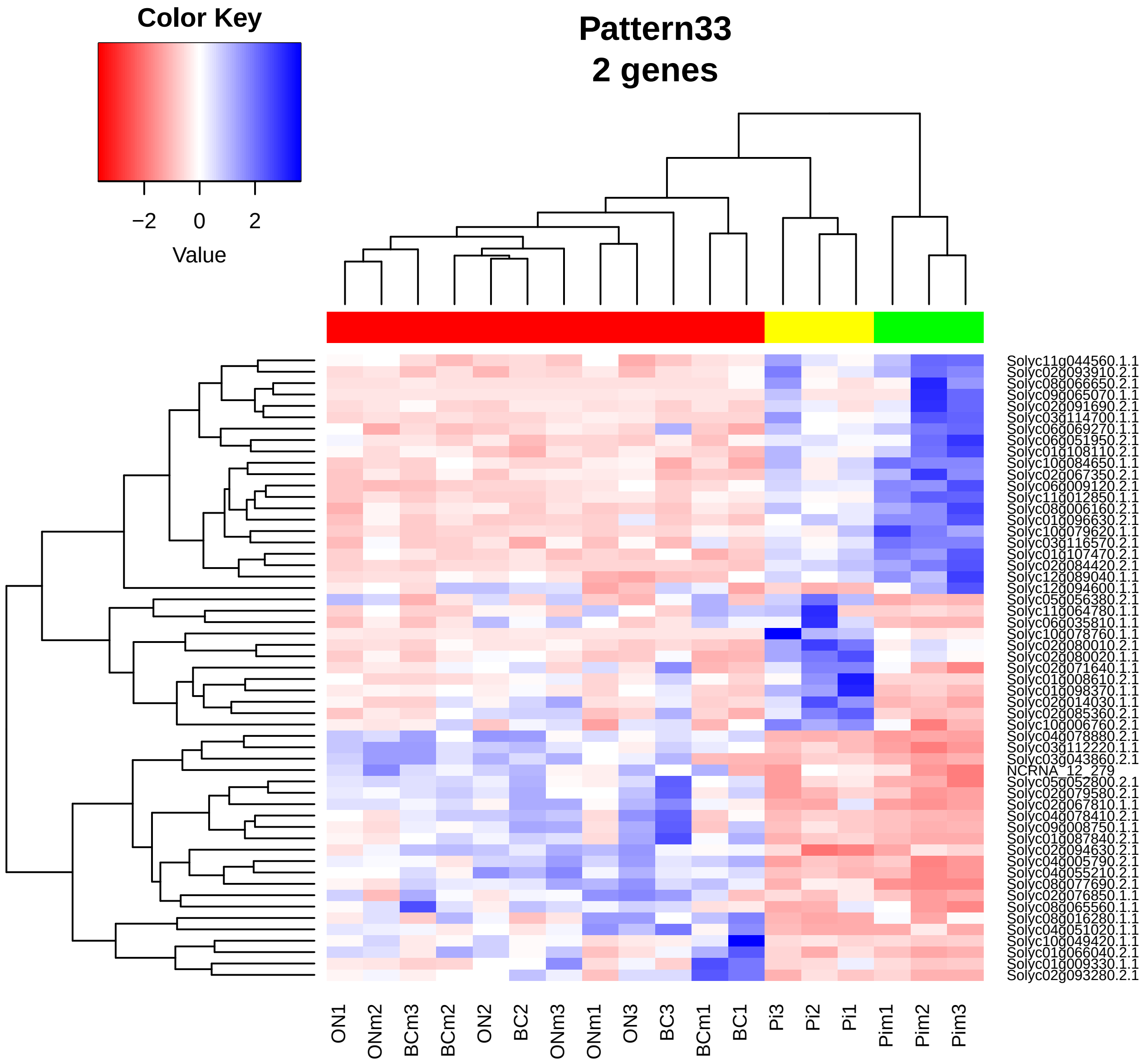

Color Key
−2
0
2
Value
Pattern33
2 genes
Solyc11g044560.1.1
Solyc02g093910.2.1
Solyc08g066650.2.1
Solyc09g065070.1.1
Solyc02g091690.2.1
Solyc03g114700.1.1
Solyc06g069270.1.1
Solyc06g051950.2.1
Solyc01g108110.2.1
Solyc10g084650.1.1
Solyc02g067350.2.1
Solyc06g009120.2.1
Solyc11g012850.1.1
Solyc08g006160.2.1
Solyc01g096630.2.1
Solyc10g079620.1.1
Solyc03g116570.2.1
Solyc01g107470.2.1
Solyc02g084420.2.1
Solyc12g089040.1.1
Solyc12g094600.1.1
Solyc05g056380.2.1
Solyc11g064780.1.1
Solyc06g035810.1.1
Solyc10g078760.1.1
Solyc02g080010.2.1
Solyc02g080020.1.1
Solyc02g071640.1.1
Solyc01g008610.2.1
Solyc01g098370.1.1
Solyc02g014030.1.1
Solyc02g085360.2.1
Solyc10g006760.2.1
Solyc04g078880.2.1
Solyc03g112220.1.1
Solyc03g043860.2.1
NCRNA_12_279
Solyc05g052800.2.1
Solyc02g079580.2.1
Solyc02g067810.1.1
Solyc04g078410.2.1
Solyc09g008750.1.1
Solyc01g087840.2.1
Solyc02g094630.2.1
Solyc04g005790.2.1
Solyc04g055210.2.1
Solyc08g077690.2.1
Solyc02g076850.1.1
Solyc08g065560.1.1
Solyc08g016280.1.1
Solyc04g051020.1.1
Solyc10g049420.1.1
Solyc01g066040.2.1
Solyc01g009330.1.1
Solyc02g093280.2.1
ON1
ONm2
BCm3
BCm2
ON2
BC2
ONm3
ONm1
ON3
BC3
BCm1
BC1
Pi3
Pi2
Pi1
Pim1
Pim2
Pim3

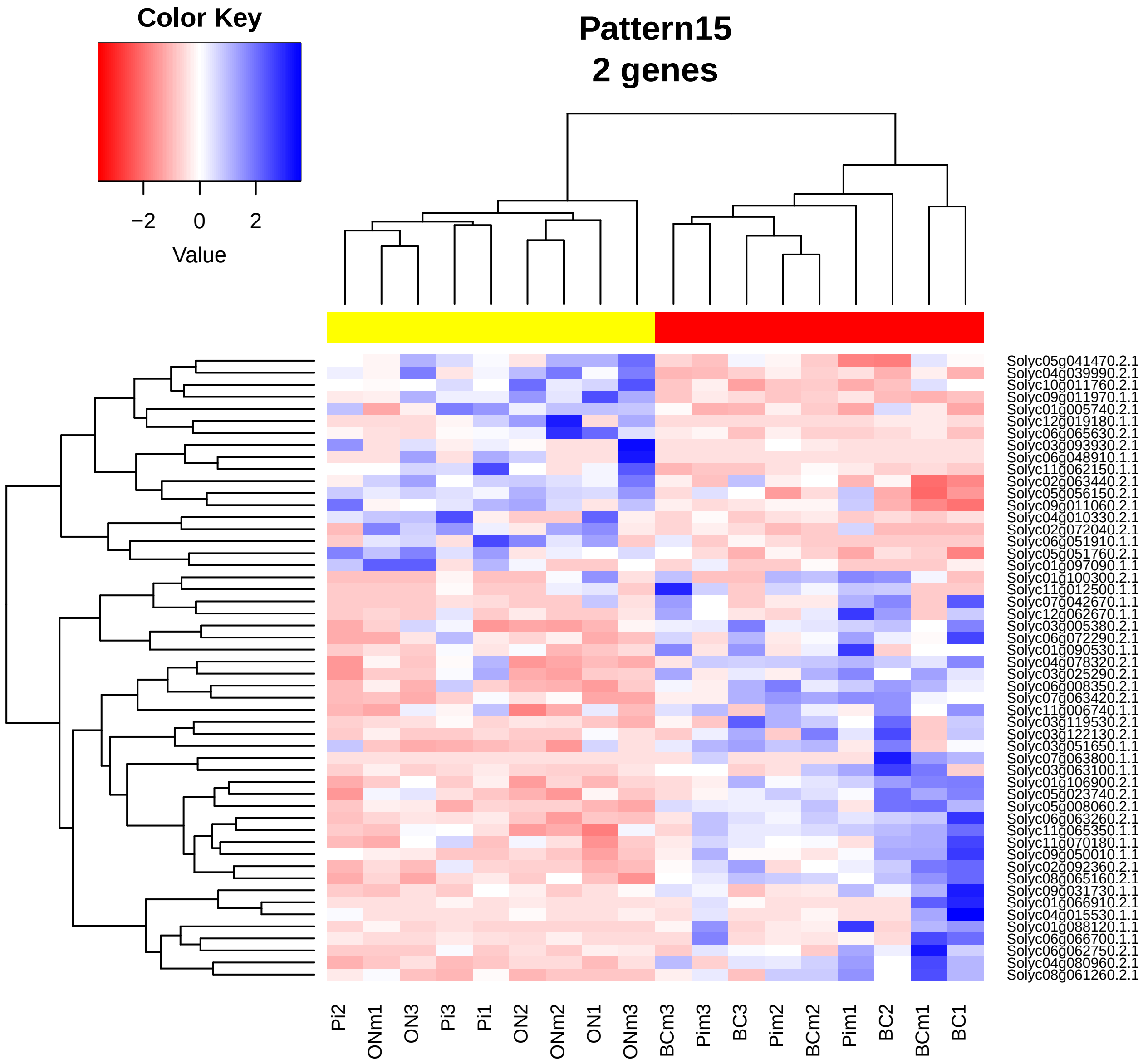

Color Key
−2
0
2
Value
Pattern15
2 genes
Solyc05g041470.2.1
Solyc04g039990.2.1
Solyc10g011760.2.1
Solyc09g011970.1.1
Solyc01g005740.2.1
Solyc12g019180.1.1
Solyc06g065630.2.1
Solyc03g093930.2.1
Solyc06g048910.1.1
Solyc11g062150.1.1
Solyc02g063440.2.1
Solyc05g056150.2.1
Solyc09g011060.2.1
Solyc04g010330.2.1
Solyc02g072040.2.1
Solyc06g051910.1.1
Solyc05g051760.2.1
Solyc01g097090.1.1
Solyc01g100300.2.1
Solyc11g012500.1.1
Solyc07g042670.1.1
Solyc12g062670.1.1
Solyc03g005380.2.1
Solyc06g072290.2.1
Solyc01g090530.1.1
Solyc04g078320.2.1
Solyc03g025290.2.1
Solyc06g008350.2.1
Solyc07g063420.2.1
Solyc11g006740.1.1
Solyc03g119530.2.1
Solyc03g122130.2.1
Solyc03g051650.1.1
Solyc07g063800.1.1
Solyc03g063100.1.1
Solyc01g106900.2.1
Solyc05g023740.2.1
Solyc05g008060.2.1
Solyc06g063260.2.1
Solyc11g065350.1.1
Solyc11g070180.1.1
Solyc09g050010.1.1
Solyc02g092360.2.1
Solyc08g065160.2.1
Solyc09g031730.1.1
Solyc01g066910.2.1
Solyc04g015530.1.1
Solyc01g088120.1.1
Solyc06g066700.1.1
Solyc06g062750.2.1
Solyc04g080960.2.1
Solyc08g061260.2.1
Pi2
ONm1
ON3
Pi3
Pi1
ON2
ONm2
ON1
ONm3
BCm3
Pim3
BC3
Pim2
BCm2
Pim1
BC2
BCm1
BC1

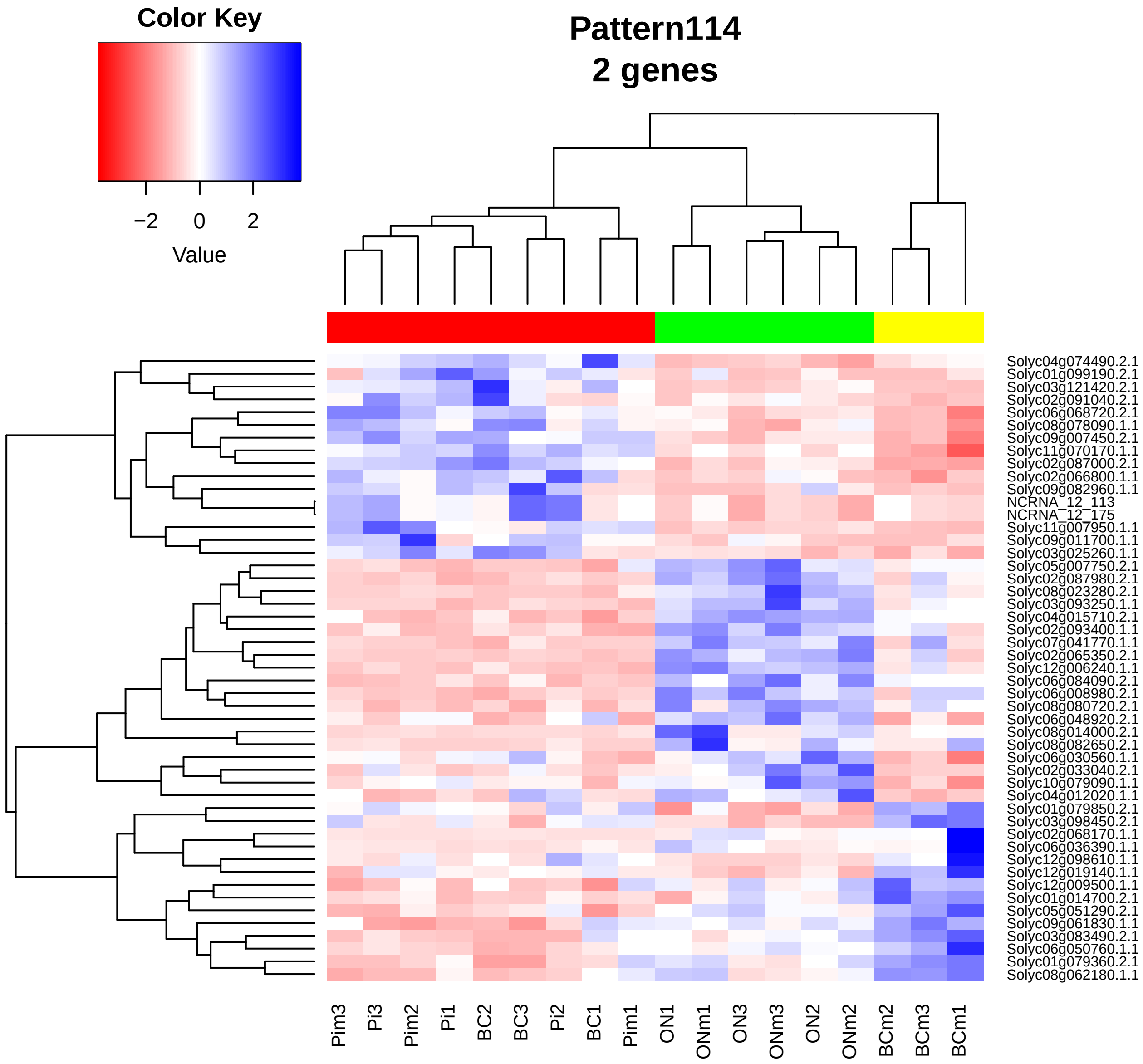
Color Key
−2
0
2
Value
Pattern114
2 genes
Solyc04g074490.2.1
Solyc01g099190.2.1
Solyc03g121420.2.1
Solyc02g091040.2.1
Solyc06g068720.2.1
Solyc08g078090.1.1
Solyc09g007450.2.1
Solyc11g070170.1.1
Solyc02g087000.2.1
Solyc02g066800.1.1
Solyc09g082960.1.1
NCRNA_12_113
NCRNA_12_175
Solyc11g007950.1.1
Solyc09g011700.1.1
Solyc03g025260.1.1
Solyc05g007750.2.1
Solyc02g087980.2.1
Solyc08g023280.2.1
Solyc03g093250.1.1
Solyc04g015710.2.1
Solyc02g093400.1.1
Solyc07g041770.1.1
Solyc02g065350.2.1
Solyc12g006240.1.1
Solyc06g084090.2.1
Solyc06g008980.2.1
Solyc08g080720.2.1
Solyc06g048920.2.1
Solyc08g014000.2.1
Solyc08g082650.2.1
Solyc06g030560.1.1
Solyc02g033040.2.1
Solyc10g079090.1.1
Solyc04g012020.1.1
Solyc01g079850.2.1
Solyc03g098450.2.1
Solyc02g068170.1.1
Solyc06g036390.1.1
Solyc12g098610.1.1
Solyc12g019140.1.1
Solyc12g009500.1.1
Solyc01g014700.2.1
Solyc05g051290.2.1
Solyc09g061830.1.1
Solyc03g083490.2.1
Solyc06g050760.1.1
Solyc01g079360.2.1
Solyc08g062180.1.1
Pim3
Pi3
Pim2
Pi1
BC2
BC3
Pi2
BC1
Pim1
ON1
ONm1
ON3
ONm3
ON2
ONm2
BCm2
BCm3
BCm1

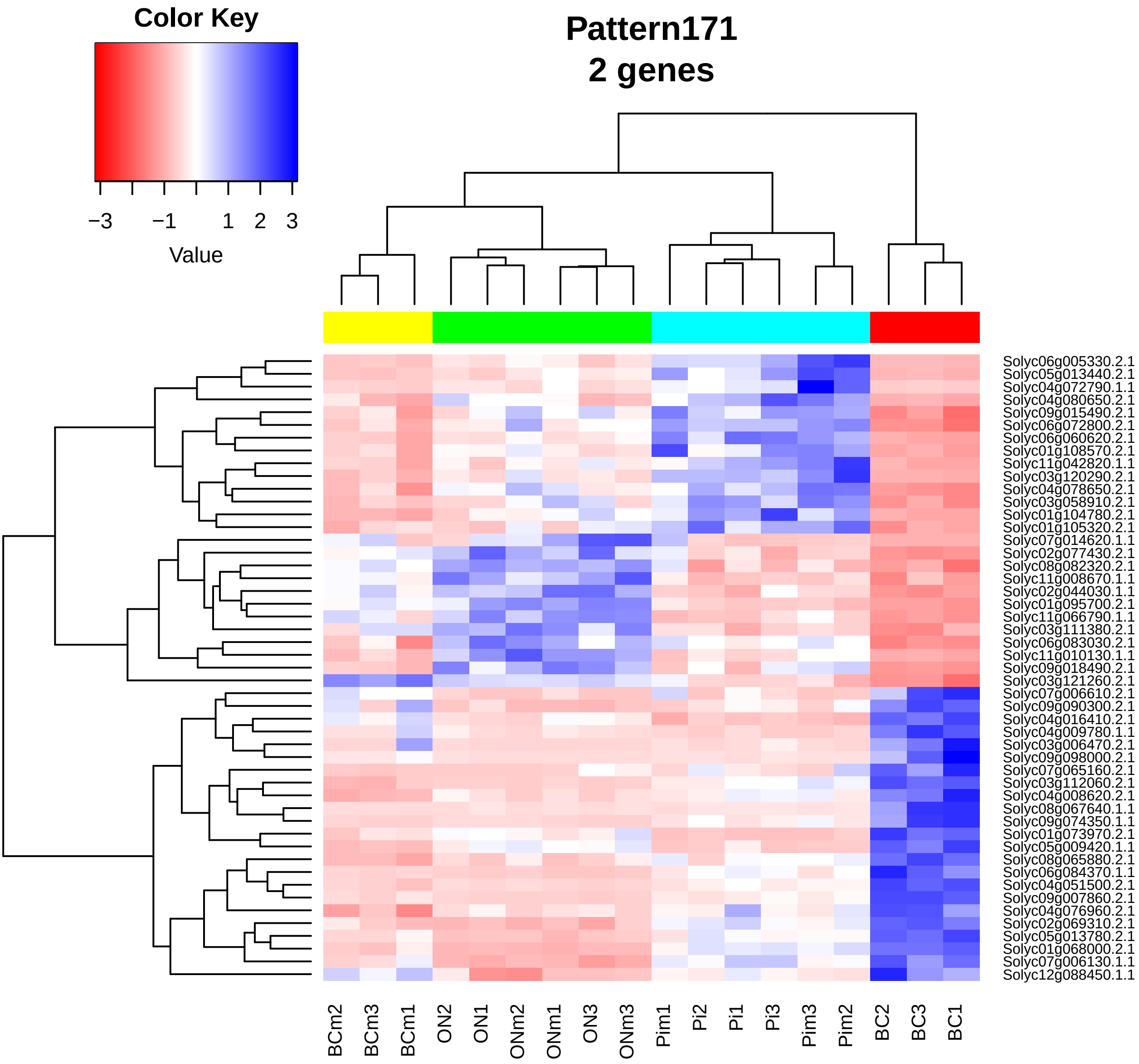

Color Key
−3
−1
1
2
3
Value
Pattern171
2 genes
Solyc06g005330.2.1
Solyc05g013440.2.1
Solyc04g072790.1.1
Solyc04g080650.2.1
Solyc09g015490.2.1
Solyc06g072800.2.1
Solyc06g060620.2.1
Solyc01g108570.2.1
Solyc11g042820.1.1
Solyc03g120290.2.1
Solyc04g078650.2.1
Solyc03g058910.2.1
Solyc01g104780.2.1
Solyc01g105320.2.1
Solyc07g014620.1.1
Solyc02g077430.2.1
Solyc08g082320.2.1
Solyc11g008670.1.1
Solyc02g044030.1.1
Solyc01g095700.2.1
Solyc11g066790.1.1
Solyc03g111380.2.1
Solyc06g083030.2.1
Solyc11g010130.1.1
Solyc09g018490.2.1
Solyc03g121260.2.1
Solyc07g006610.2.1
Solyc09g090300.2.1
Solyc04g016410.2.1
Solyc04g009780.1.1
Solyc03g006470.2.1
Solyc09g098000.2.1
Solyc07g065160.2.1
Solyc03g112060.2.1
Solyc04g008620.2.1
Solyc08g067640.1.1
Solyc09g074350.1.1
Solyc01g073970.2.1
Solyc05g009420.1.1
Solyc08g065880.2.1
Solyc06g084370.1.1
Solyc04g051500.2.1
Solyc09g007860.2.1
Solyc04g076960.2.1
Solyc02g069310.2.1
Solyc05g013780.2.1
Solyc01g068000.2.1
Solyc07g006130.1.1
Solyc12g088450.1.1
BCm2
BCm3
BCm1
ON2
ON1
ONm2
ONm1
ON3
ONm3
Pim1
Pi2
Pi1
Pi3
Pim3
Pim2
BC2
BC3
BC1

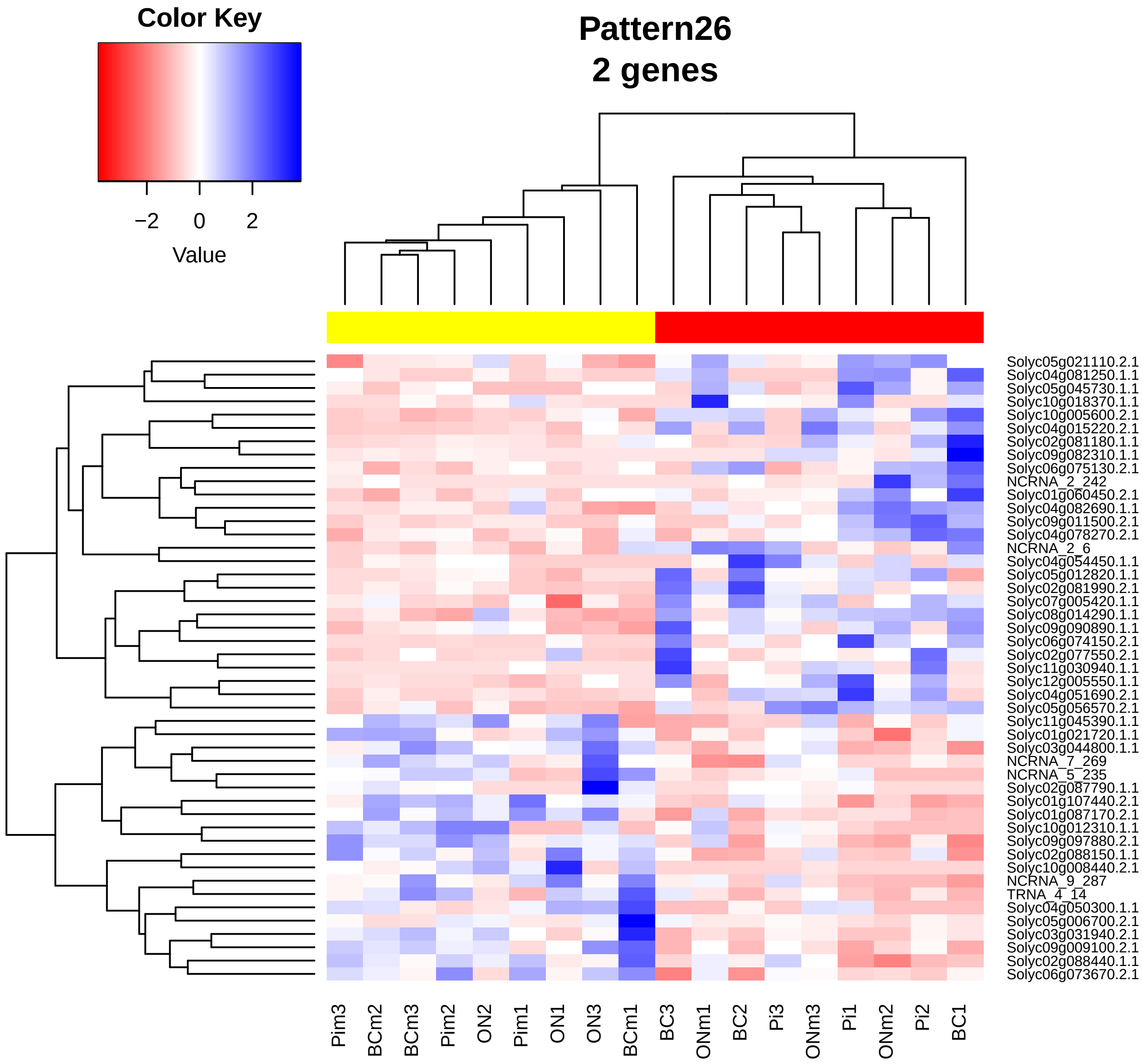
Color Key
−2
0
2
Value
Pattern26
2 genes
Solyc05g021110.2.1
Solyc04g081250.1.1
Solyc05g045730.1.1
Solyc10g018370.1.1
Solyc10g005600.2.1
Solyc04g015220.2.1
Solyc02g081180.1.1
Solyc09g082310.1.1
Solyc06g075130.2.1
NCRNA_2_242
Solyc01g060450.2.1
Solyc04g082690.1.1
Solyc09g011500.2.1
Solyc04g078270.2.1
NCRNA_2_6
Solyc04g054450.1.1
Solyc05g012820.1.1
Solyc02g081990.2.1
Solyc07g005420.1.1
Solyc08g014290.1.1
Solyc09g090890.1.1
Solyc06g074150.2.1
Solyc02g077550.2.1
Solyc11g030940.1.1
Solyc12g005550.1.1
Solyc04g051690.2.1
Solyc05g056570.2.1
Solyc11g045390.1.1
Solyc01g021720.1.1
Solyc03g044800.1.1
NCRNA_7_269
NCRNA_5_235
Solyc02g087790.1.1
Solyc01g107440.2.1
Solyc01g087170.2.1
Solyc10g012310.1.1
Solyc09g097880.2.1
Solyc02g088150.1.1
Solyc10g008440.2.1
NCRNA_9_287
TRNA_4_14
Solyc04g050300.1.1
Solyc05g006700.2.1
Solyc03g031940.2.1
Solyc09g009100.2.1
Solyc02g088440.1.1
Solyc06g073670.2.1
Pim3
BCm2
BCm3
Pim2
ON2
Pim1
ON1
ON3
BCm1
BC3
ONm1
BC2
Pi3
ONm3
Pi1
ONm2
Pi2
BC1

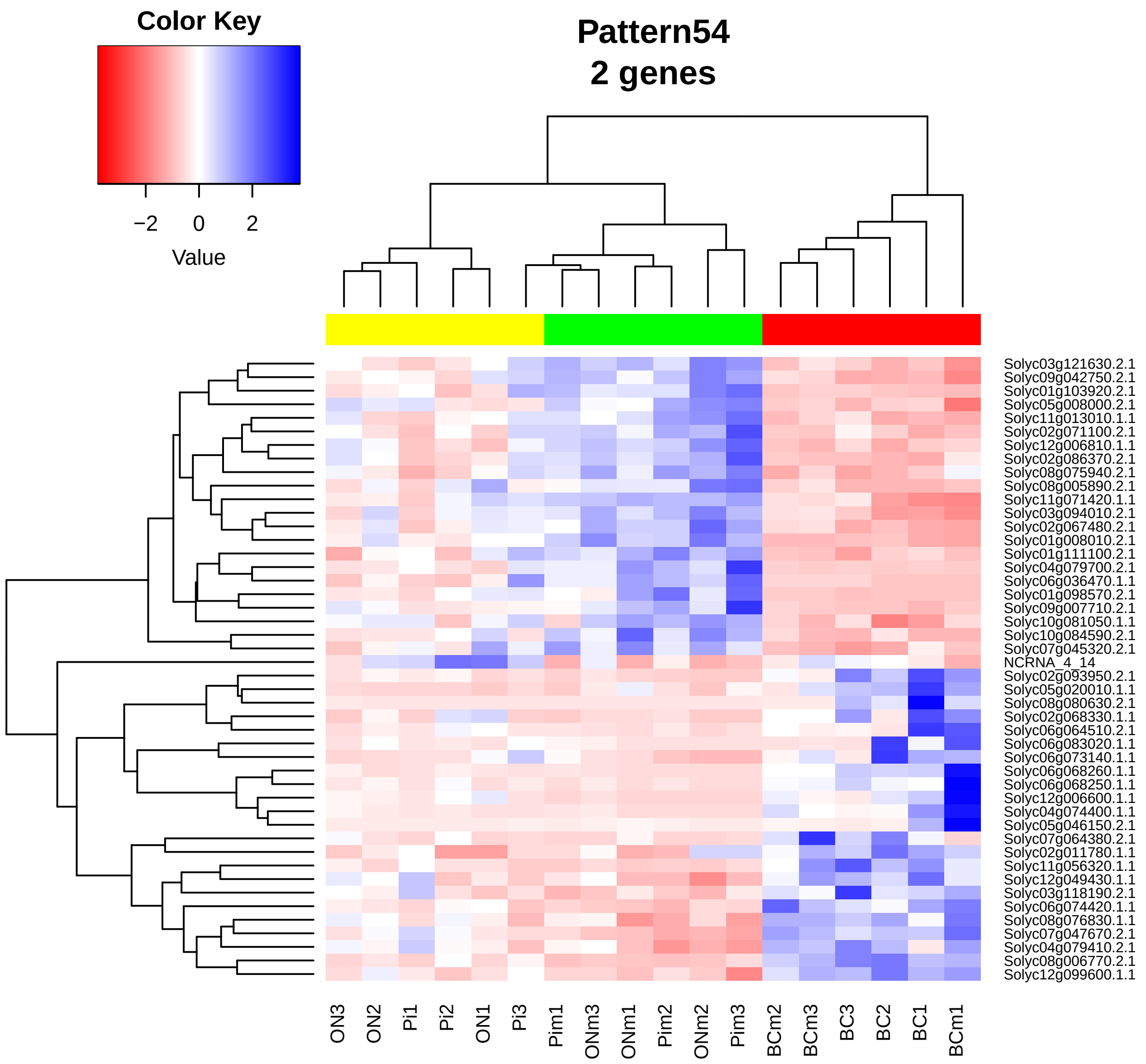
Color Key
−2
0
2
Value
Pattern54
2 genes
Solyc03g121630.2.1
Solyc09g042750.2.1
Solyc01g103920.2.1
Solyc05g008000.2.1
Solyc11g013010.1.1
Solyc02g071100.2.1
Solyc12g006810.1.1
Solyc02g086370.2.1
Solyc08g075940.2.1
Solyc08g005890.2.1
Solyc11g071420.1.1
Solyc03g094010.2.1
Solyc02g067480.2.1
Solyc01g008010.2.1
Solyc01g111100.2.1
Solyc04g079700.2.1
Solyc06g036470.1.1
Solyc01g098570.2.1
Solyc09g007710.2.1
Solyc10g081050.1.1
Solyc10g084590.2.1
Solyc07g045320.2.1
NCRNA_4_14
Solyc02g093950.2.1
Solyc05g020010.1.1
Solyc08g080630.2.1
Solyc02g068330.1.1
Solyc06g064510.2.1
Solyc06g083020.1.1
Solyc06g073140.1.1
Solyc06g068260.1.1
Solyc06g068250.1.1
Solyc12g006600.1.1
Solyc04g074400.1.1
Solyc05g046150.2.1
Solyc07g064380.2.1
Solyc02g011780.1.1
Solyc11g056320.1.1
Solyc12g049430.1.1
Solyc03g118190.2.1
Solyc06g074420.1.1
Solyc08g076830.1.1
Solyc07g047670.2.1
Solyc04g079410.2.1
Solyc08g006770.2.1
Solyc12g099600.1.1
ON3
ON2
Pi1
Pi2
ON1
Pi3
Pim1
ONm3
ONm1
Pim2
ONm2
Pim3
BCm2
BCm3
BC3
BC2
BC1
BCm1

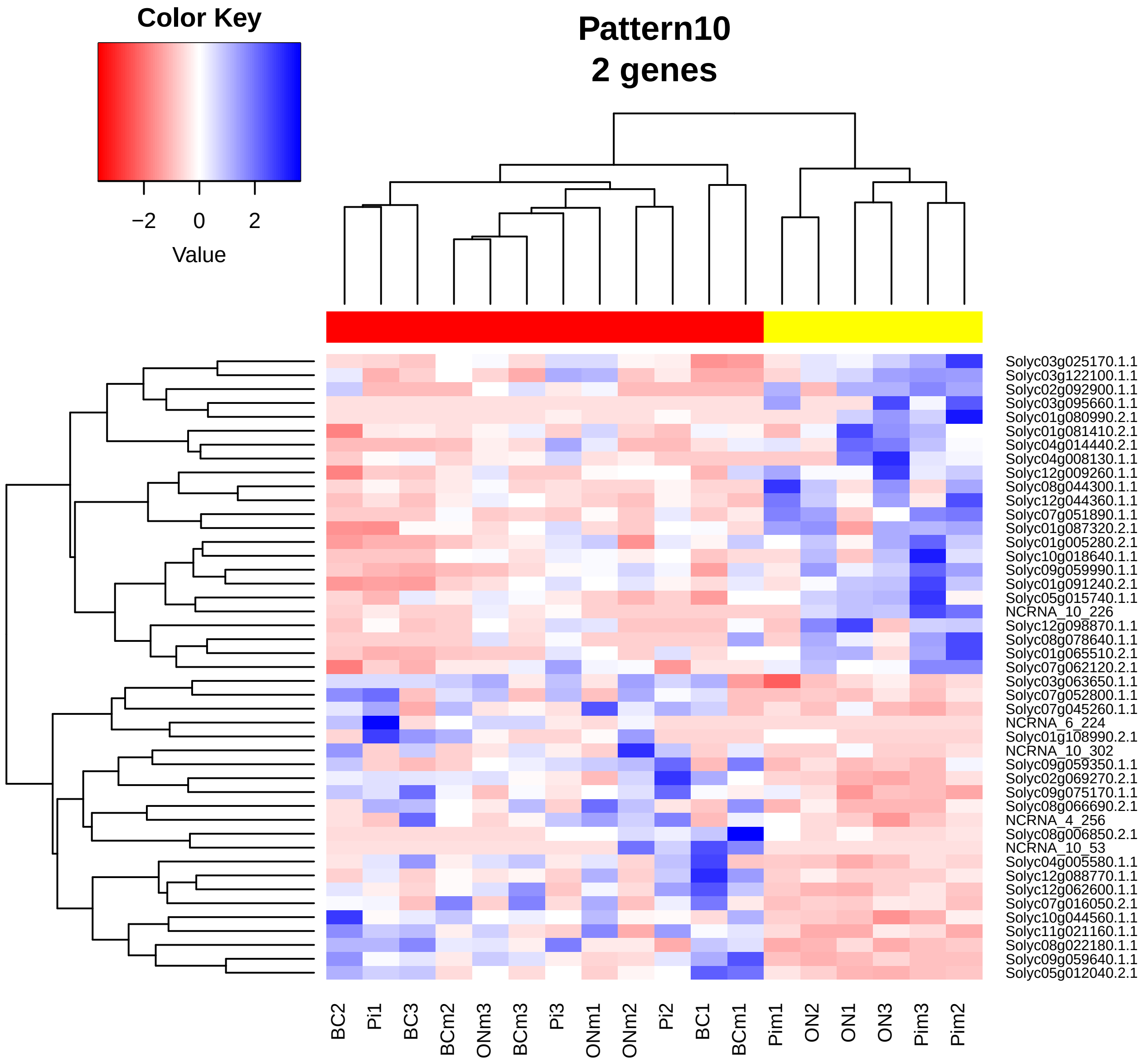
Color Key
−2
0
2
Value
Pattern10
2 genes
Solyc03g025170.1.1
Solyc03g122100.1.1
Solyc02g092900.1.1
Solyc03g095660.1.1
Solyc01g080990.2.1
Solyc01g081410.2.1
Solyc04g014440.2.1
Solyc04g008130.1.1
Solyc12g009260.1.1
Solyc08g044300.1.1
Solyc12g044360.1.1
Solyc07g051890.1.1
Solyc01g087320.2.1
Solyc01g005280.2.1
Solyc10g018640.1.1
Solyc09g059990.1.1
Solyc01g091240.2.1
Solyc05g015740.1.1
NCRNA_10_226
Solyc12g098870.1.1
Solyc08g078640.1.1
Solyc01g065510.2.1
Solyc07g062120.2.1
Solyc03g063650.1.1
Solyc07g052800.1.1
Solyc07g045260.1.1
NCRNA_6_224
Solyc01g108990.2.1
NCRNA_10_302
Solyc09g059350.1.1
Solyc02g069270.2.1
Solyc09g075170.1.1
Solyc08g066690.2.1
NCRNA_4_256
Solyc08g006850.2.1
NCRNA_10_53
Solyc04g005580.1.1
Solyc12g088770.1.1
Solyc12g062600.1.1
Solyc07g016050.2.1
Solyc10g044560.1.1
Solyc11g021160.1.1
Solyc08g022180.1.1
Solyc09g059640.1.1
Solyc05g012040.2.1
BC2
Pi1
BC3
BCm2
ONm3
BCm3
Pi3
ONm1
ONm2
Pi2
BC1
BCm1
Pim1
ON2
ON1
ON3
Pim3
Pim2

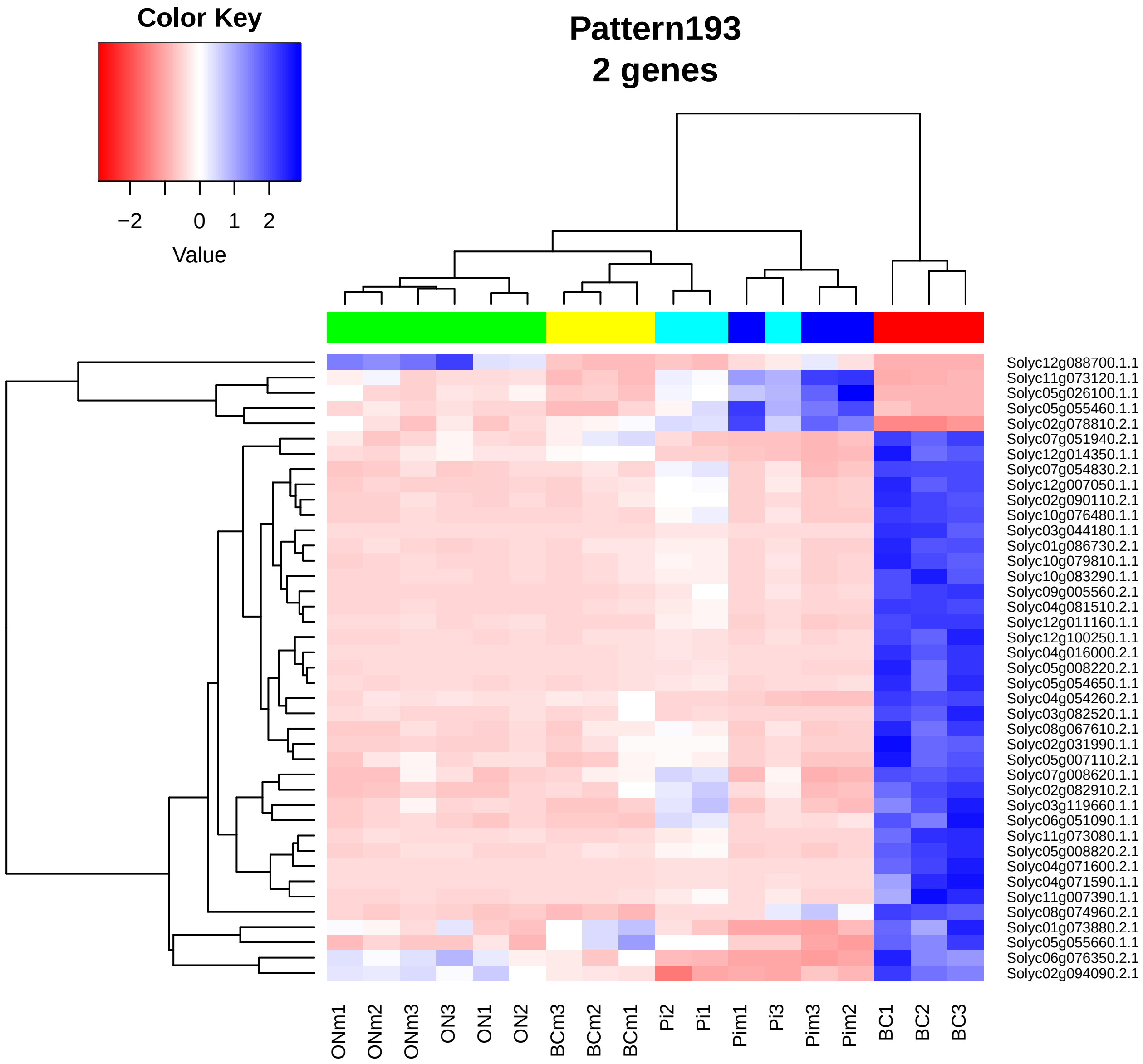

Color Key
−2
0
1
2
Value
Pattern193
2 genes
Solyc12g088700.1.1
Solyc11g073120.1.1
Solyc05g026100.1.1
Solyc05g055460.1.1
Solyc02g078810.2.1
Solyc07g051940.2.1
Solyc12g014350.1.1
Solyc07g054830.2.1
Solyc12g007050.1.1
Solyc02g090110.2.1
Solyc10g076480.1.1
Solyc03g044180.1.1
Solyc01g086730.2.1
Solyc10g079810.1.1
Solyc10g083290.1.1
Solyc09g005560.2.1
Solyc04g081510.2.1
Solyc12g011160.1.1
Solyc12g100250.1.1
Solyc04g016000.2.1
Solyc05g008220.2.1
Solyc05g054650.1.1
Solyc04g054260.2.1
Solyc03g082520.1.1
Solyc08g067610.2.1
Solyc02g031990.1.1
Solyc05g007110.2.1
Solyc07g008620.1.1
Solyc02g082910.2.1
Solyc03g119660.1.1
Solyc06g051090.1.1
Solyc11g073080.1.1
Solyc05g008820.2.1
Solyc04g071600.2.1
Solyc04g071590.1.1
Solyc11g007390.1.1
Solyc08g074960.2.1
Solyc01g073880.2.1
Solyc05g055660.1.1
Solyc06g076350.2.1
Solyc02g094090.2.1
ONm1
ONm2
ONm3
ON3
ON1
ON2
BCm3
BCm2
BCm1
Pi2
Pi1
Pim1
Pi3
Pim3
Pim2
BC1
BC2
BC3

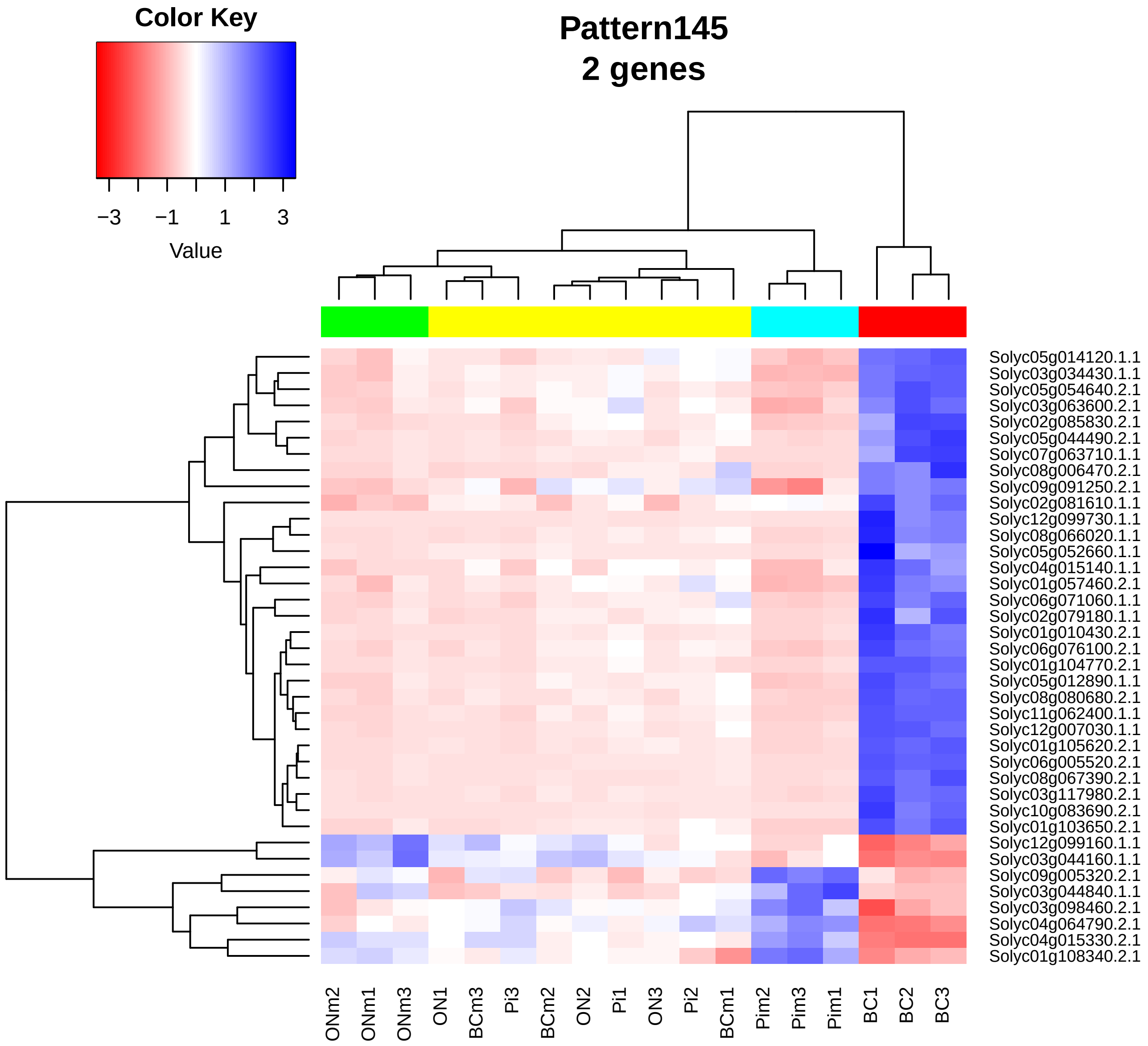
Color Key
−3
−1
1
3
Value
Pattern145
2 genes
Solyc05g014120.1.1
Solyc03g034430.1.1
Solyc05g054640.2.1
Solyc03g063600.2.1
Solyc02g085830.2.1
Solyc05g044490.2.1
Solyc07g063710.1.1
Solyc08g006470.2.1
Solyc09g091250.2.1
Solyc02g081610.1.1
Solyc12g099730.1.1
Solyc08g066020.1.1
Solyc05g052660.1.1
Solyc04g015140.1.1
Solyc01g057460.2.1
Solyc06g071060.1.1
Solyc02g079180.1.1
Solyc01g010430.2.1
Solyc06g076100.2.1
Solyc01g104770.2.1
Solyc05g012890.1.1
Solyc08g080680.2.1
Solyc11g062400.1.1
Solyc12g007030.1.1
Solyc01g105620.2.1
Solyc06g005520.2.1
Solyc08g067390.2.1
Solyc03g117980.2.1
Solyc10g083690.2.1
Solyc01g103650.2.1
Solyc12g099160.1.1
Solyc03g044160.1.1
Solyc09g005320.2.1
Solyc03g044840.1.1
Solyc03g098460.2.1
Solyc04g064790.2.1
Solyc04g015330.2.1
Solyc01g108340.2.1
ONm2
ONm1
ONm3
ON1
BCm3
Pi3
BCm2
ON2
Pi1
ON3
Pi2
BCm1
Pim2
Pim3
Pim1
BC1
BC2
BC3

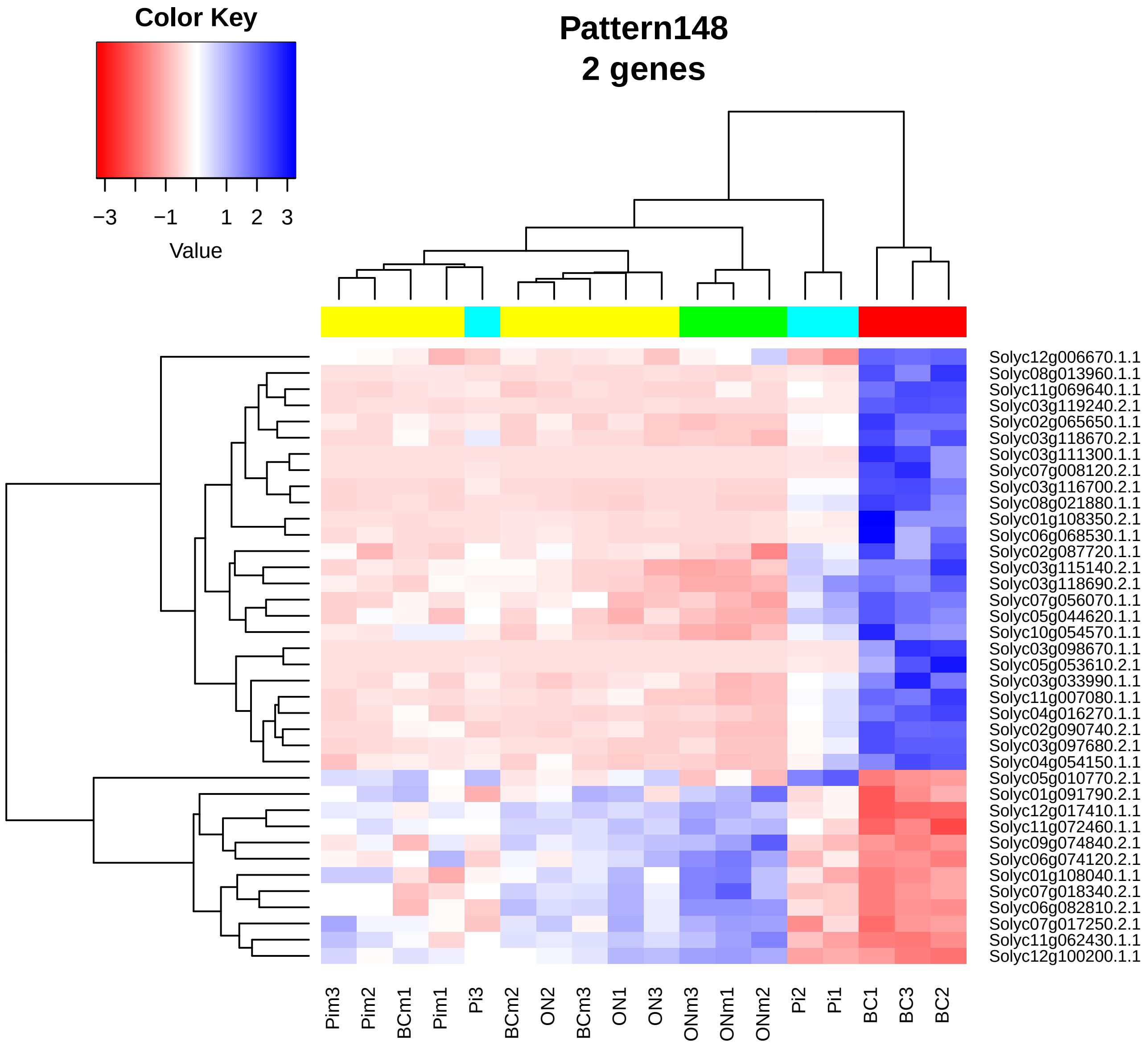

Color Key
−3
−1
1
2
3
Value
Pattern148
2 genes
Solyc12g006670.1.1
Solyc08g013960.1.1
Solyc11g069640.1.1
Solyc03g119240.2.1
Solyc02g065650.1.1
Solyc03g118670.2.1
Solyc03g111300.1.1
Solyc07g008120.2.1
Solyc03g116700.2.1
Solyc08g021880.1.1
Solyc01g108350.2.1
Solyc06g068530.1.1
Solyc02g087720.1.1
Solyc03g115140.2.1
Solyc03g118690.2.1
Solyc07g056070.1.1
Solyc05g044620.1.1
Solyc10g054570.1.1
Solyc03g098670.1.1
Solyc05g053610.2.1
Solyc03g033990.1.1
Solyc11g007080.1.1
Solyc04g016270.1.1
Solyc02g090740.2.1
Solyc03g097680.2.1
Solyc04g054150.1.1
Solyc05g010770.2.1
Solyc01g091790.2.1
Solyc12g017410.1.1
Solyc11g072460.1.1
Solyc09g074840.2.1
Solyc06g074120.2.1
Solyc01g108040.1.1
Solyc07g018340.2.1
Solyc06g082810.2.1
Solyc07g017250.2.1
Solyc11g062430.1.1
Solyc12g100200.1.1
Pim3
Pim2
BCm1
Pim1
Pi3
BCm2
ON2
BCm3
ON1
ON3
ONm3
ONm1
ONm2
Pi2
Pi1
BC1
BC3
BC2

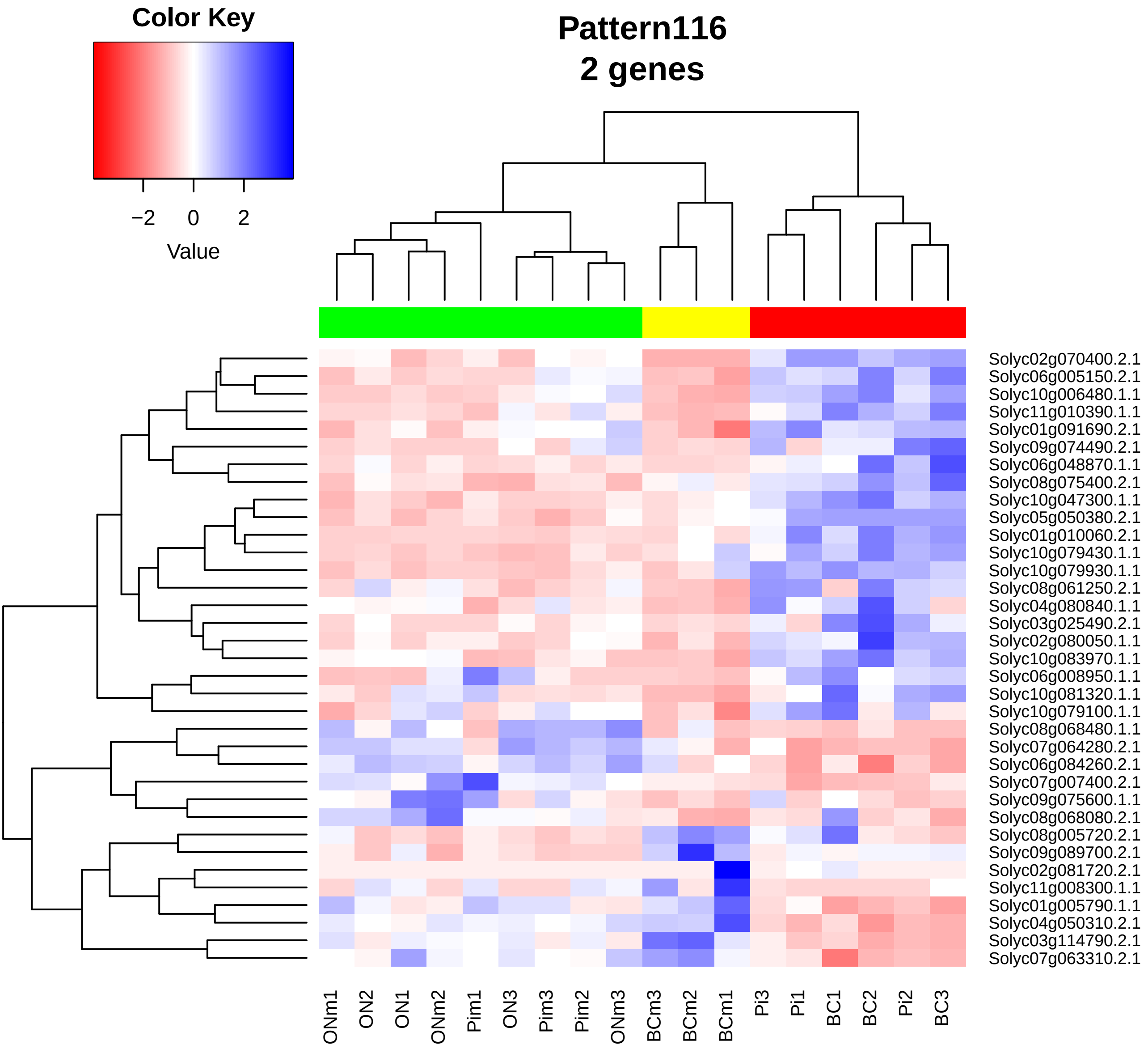

Color Key
−2
0
2
Value
Pattern116
2 genes
Solyc02g070400.2.1
Solyc06g005150.2.1
Solyc10g006480.1.1
Solyc11g010390.1.1
Solyc01g091690.2.1
Solyc09g074490.2.1
Solyc06g048870.1.1
Solyc08g075400.2.1
Solyc10g047300.1.1
Solyc05g050380.2.1
Solyc01g010060.2.1
Solyc10g079430.1.1
Solyc10g079930.1.1
Solyc08g061250.2.1
Solyc04g080840.1.1
Solyc03g025490.2.1
Solyc02g080050.1.1
Solyc10g083970.1.1
Solyc06g008950.1.1
Solyc10g081320.1.1
Solyc10g079100.1.1
Solyc08g068480.1.1
Solyc07g064280.2.1
Solyc06g084260.2.1
Solyc07g007400.2.1
Solyc09g075600.1.1
Solyc08g068080.2.1
Solyc08g005720.2.1
Solyc09g089700.2.1
Solyc02g081720.2.1
Solyc11g008300.1.1
Solyc01g005790.1.1
Solyc04g050310.2.1
Solyc03g114790.2.1
Solyc07g063310.2.1
ONm1
ON2
ON1
ONm2
Pim1
ON3
Pim3
Pim2
ONm3
BCm3
BCm2
BCm1
Pi3
Pi1
BC1
BC2
Pi2
BC3

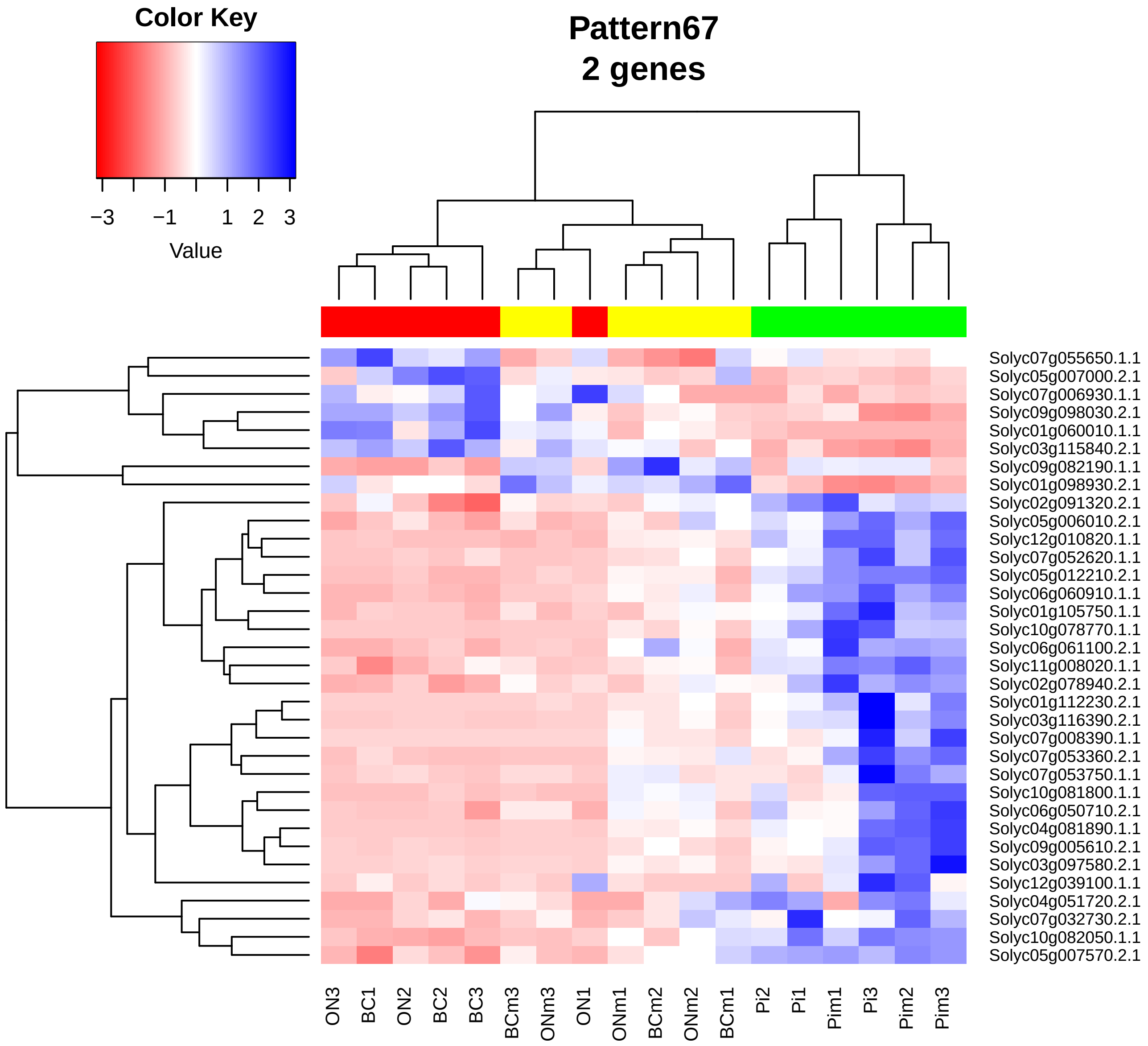
Color Key
−3
−1
1
2
3
Value
Pattern67
2 genes
Solyc07g055650.1.1
Solyc05g007000.2.1
Solyc07g006930.1.1
Solyc09g098030.2.1
Solyc01g060010.1.1
Solyc03g115840.2.1
Solyc09g082190.1.1
Solyc01g098930.2.1
Solyc02g091320.2.1
Solyc05g006010.2.1
Solyc12g010820.1.1
Solyc07g052620.1.1
Solyc05g012210.2.1
Solyc06g060910.1.1
Solyc01g105750.1.1
Solyc10g078770.1.1
Solyc06g061100.2.1
Solyc11g008020.1.1
Solyc02g078940.2.1
Solyc01g112230.2.1
Solyc03g116390.2.1
Solyc07g008390.1.1
Solyc07g053360.2.1
Solyc07g053750.1.1
Solyc10g081800.1.1
Solyc06g050710.2.1
Solyc04g081890.1.1
Solyc09g005610.2.1
Solyc03g097580.2.1
Solyc12g039100.1.1
Solyc04g051720.2.1
Solyc07g032730.2.1
Solyc10g082050.1.1
Solyc05g007570.2.1
ON3
BC1
ON2
BC2
BC3
BCm3
ONm3
ON1
ONm1
BCm2
ONm2
BCm1
Pi2
Pi1
Pim1
Pi3
Pim2
Pim3

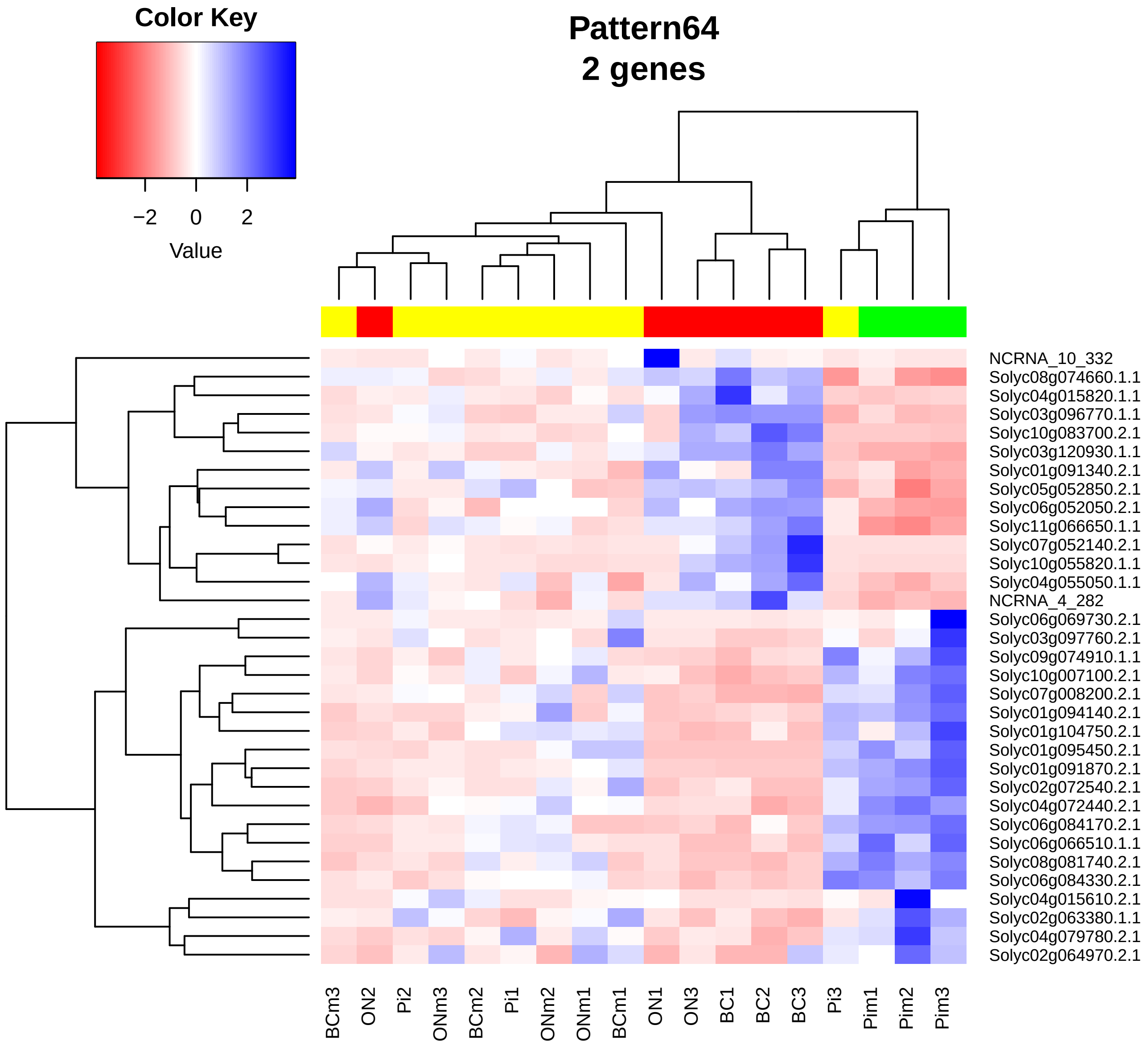
Color Key
−2
0
2
Value
Pattern64
2 genes
NCRNA_10_332
Solyc08g074660.1.1
Solyc04g015820.1.1
Solyc03g096770.1.1
Solyc10g083700.2.1
Solyc03g120930.1.1
Solyc01g091340.2.1
Solyc05g052850.2.1
Solyc06g052050.2.1
Solyc11g066650.1.1
Solyc07g052140.2.1
Solyc10g055820.1.1
Solyc04g055050.1.1
NCRNA_4_282
Solyc06g069730.2.1
Solyc03g097760.2.1
Solyc09g074910.1.1
Solyc10g007100.2.1
Solyc07g008200.2.1
Solyc01g094140.2.1
Solyc01g104750.2.1
Solyc01g095450.2.1
Solyc01g091870.2.1
Solyc02g072540.2.1
Solyc04g072440.2.1
Solyc06g084170.2.1
Solyc06g066510.1.1
Solyc08g081740.2.1
Solyc06g084330.2.1
Solyc04g015610.2.1
Solyc02g063380.1.1
Solyc04g079780.2.1
Solyc02g064970.2.1
BCm3
ON2
Pi2
ONm3
BCm2
Pi1
ONm2
ONm1
BCm1
ON1
ON3
BC1
BC2
BC3
Pi3
Pim1
Pim2
Pim3

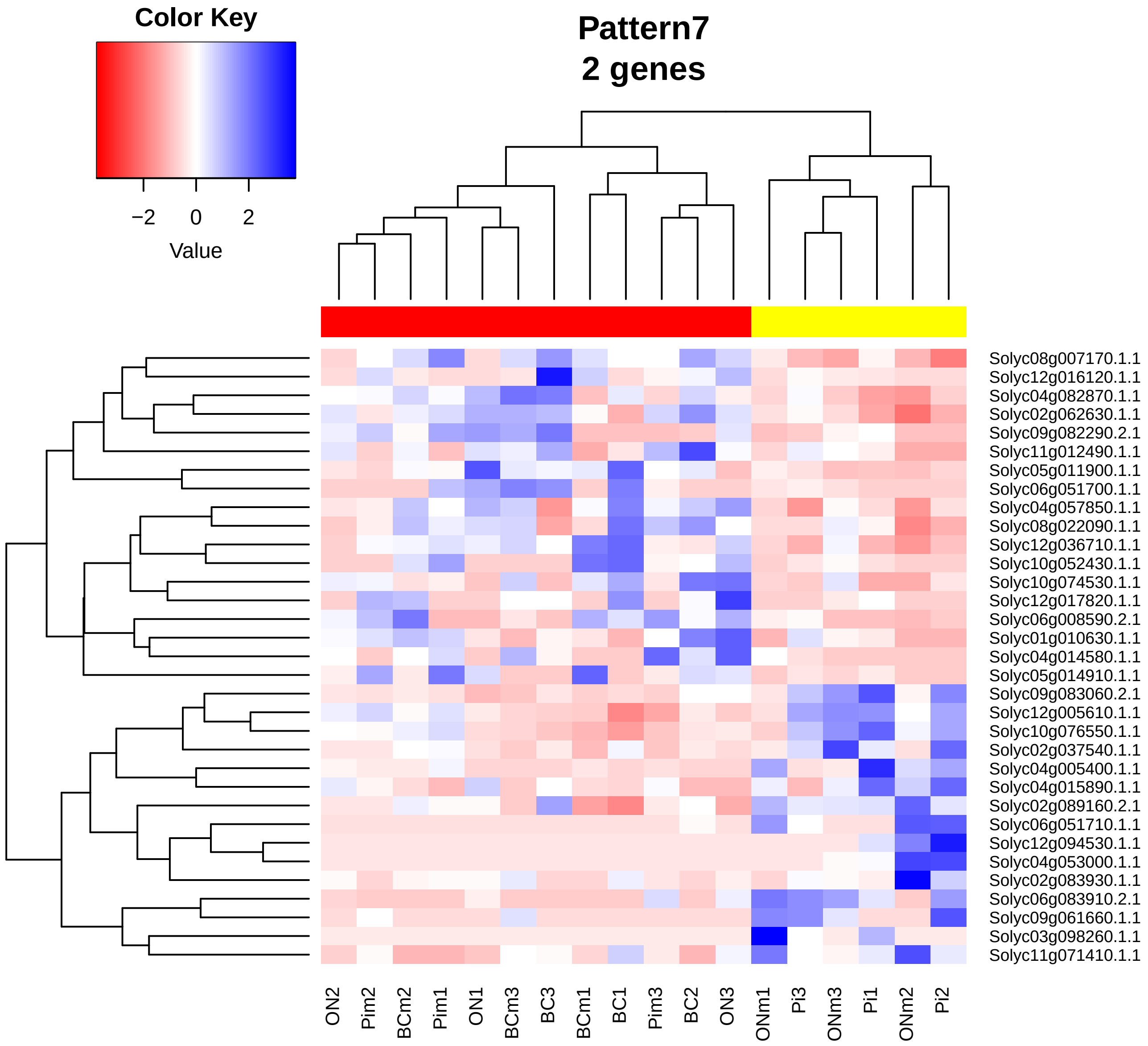

Color Key
−2
0
2
Value
Pattern7
2 genes
Solyc08g007170.1.1
Solyc12g016120.1.1
Solyc04g082870.1.1
Solyc02g062630.1.1
Solyc09g082290.2.1
Solyc11g012490.1.1
Solyc05g011900.1.1
Solyc06g051700.1.1
Solyc04g057850.1.1
Solyc08g022090.1.1
Solyc12g036710.1.1
Solyc10g052430.1.1
Solyc10g074530.1.1
Solyc12g017820.1.1
Solyc06g008590.2.1
Solyc01g010630.1.1
Solyc04g014580.1.1
Solyc05g014910.1.1
Solyc09g083060.2.1
Solyc12g005610.1.1
Solyc10g076550.1.1
Solyc02g037540.1.1
Solyc04g005400.1.1
Solyc04g015890.1.1
Solyc02g089160.2.1
Solyc06g051710.1.1
Solyc12g094530.1.1
Solyc04g053000.1.1
Solyc02g083930.1.1
Solyc06g083910.2.1
Solyc09g061660.1.1
Solyc03g098260.1.1
Solyc11g071410.1.1
ON2
Pim2
BCm2
Pim1
ON1
BCm3
BC3
BCm1
BC1
Pim3
BC2
ON3
ONm1
Pi3
ONm3
Pi1
ONm2
Pi2

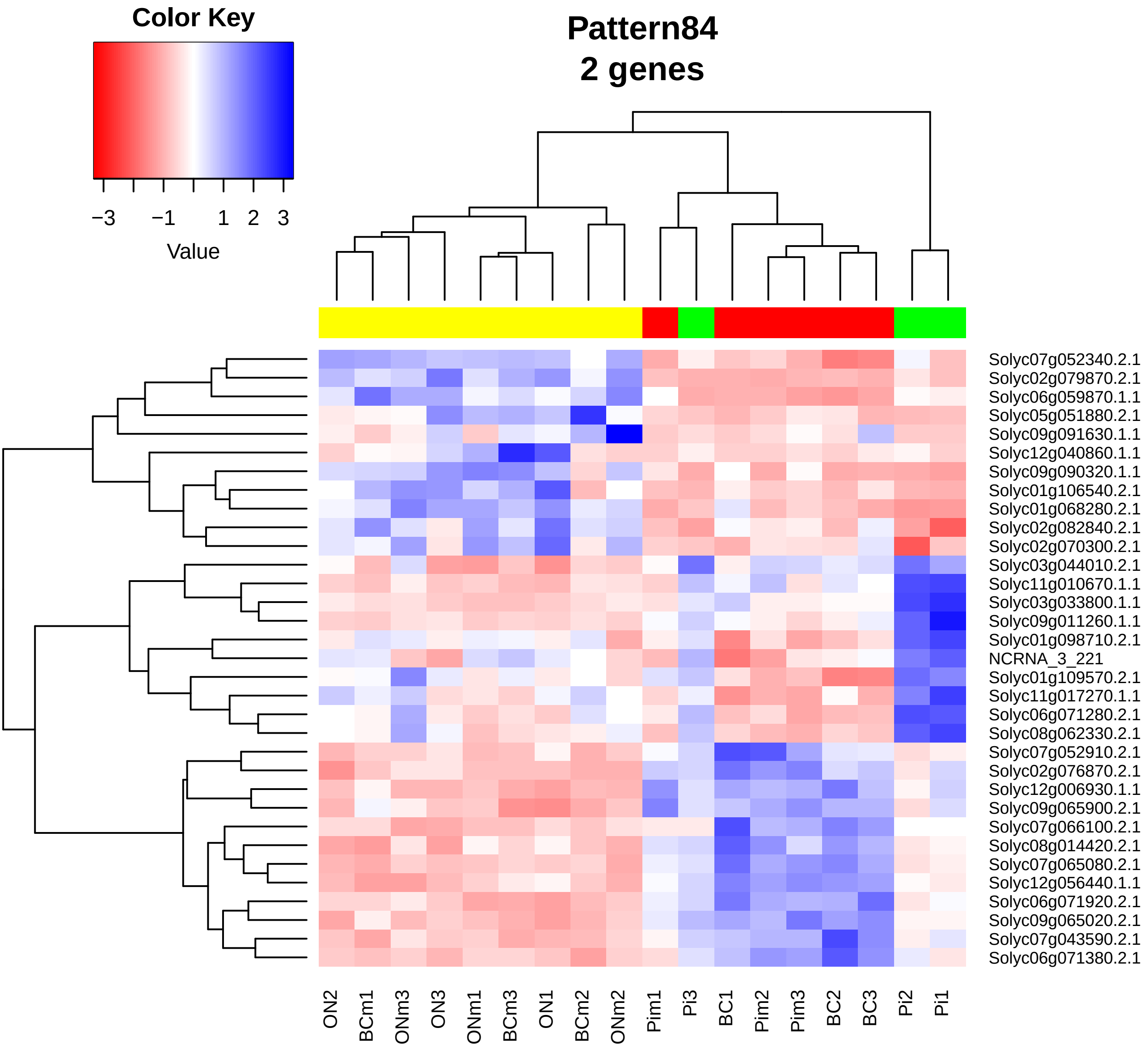
Color Key
−3
−1
1
2
3
Value
Pattern84
2 genes
Solyc07g052340.2.1
Solyc02g079870.2.1
Solyc06g059870.1.1
Solyc05g051880.2.1
Solyc09g091630.1.1
Solyc12g040860.1.1
Solyc09g090320.1.1
Solyc01g106540.2.1
Solyc01g068280.2.1
Solyc02g082840.2.1
Solyc02g070300.2.1
Solyc03g044010.2.1
Solyc11g010670.1.1
Solyc03g033800.1.1
Solyc09g011260.1.1
Solyc01g098710.2.1
NCRNA_3_221
Solyc01g109570.2.1
Solyc11g017270.1.1
Solyc06g071280.2.1
Solyc08g062330.2.1
Solyc07g052910.2.1
Solyc02g076870.2.1
Solyc12g006930.1.1
Solyc09g065900.2.1
Solyc07g066100.2.1
Solyc08g014420.2.1
Solyc07g065080.2.1
Solyc12g056440.1.1
Solyc06g071920.2.1
Solyc09g065020.2.1
Solyc07g043590.2.1
Solyc06g071380.2.1
ON2
BCm1
ONm3
ON3
ONm1
BCm3
ON1
BCm2
ONm2
Pim1
Pi3
BC1
Pim2
Pim3
BC2
BC3
Pi2
Pi1

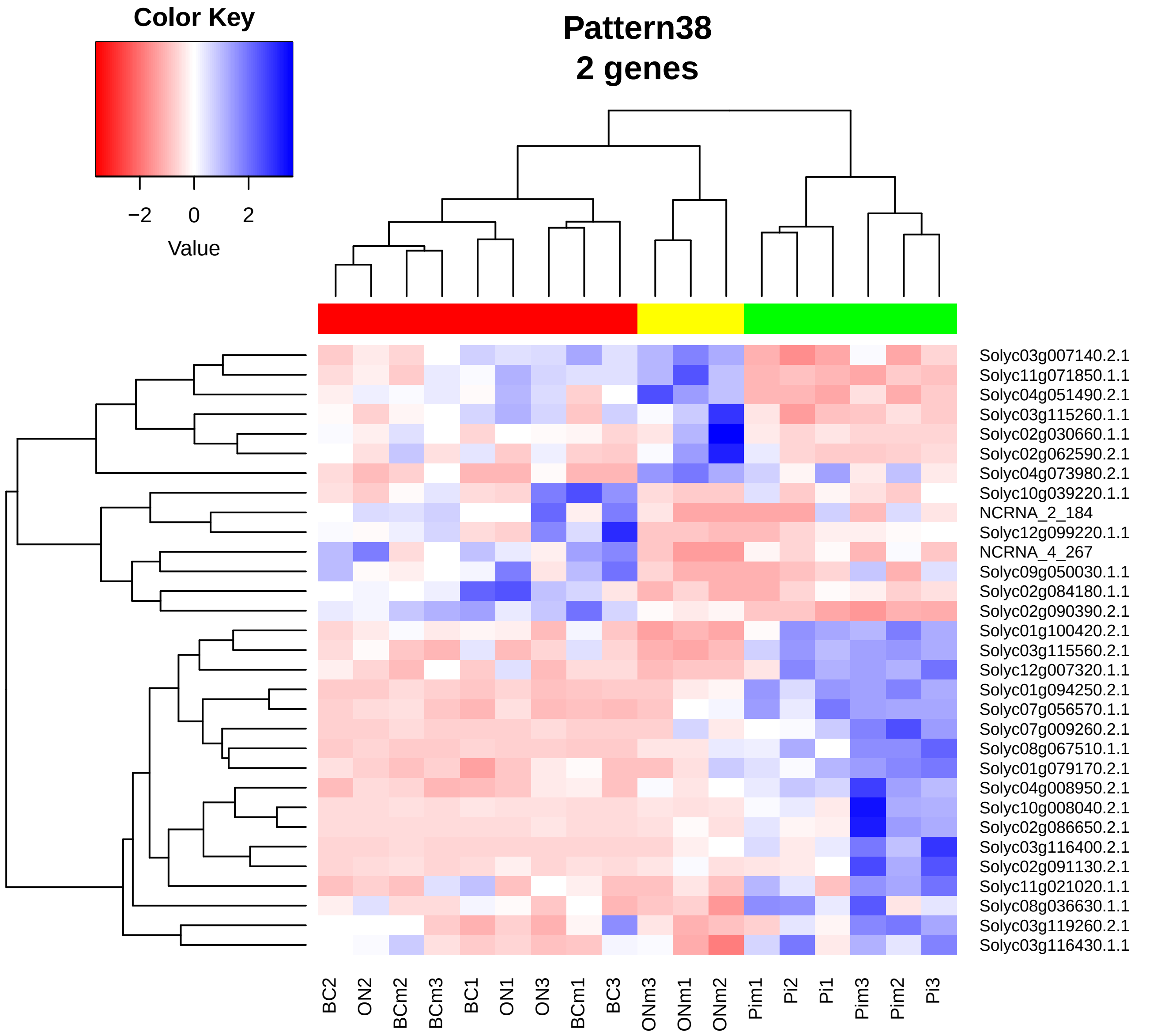
Color Key
−2
0
2
Value
Pattern38
2 genes
Solyc03g007140.2.1
Solyc11g071850.1.1
Solyc04g051490.2.1
Solyc03g115260.1.1
Solyc02g030660.1.1
Solyc02g062590.2.1
Solyc04g073980.2.1
Solyc10g039220.1.1
NCRNA_2_184
Solyc12g099220.1.1
NCRNA_4_267
Solyc09g050030.1.1
Solyc02g084180.1.1
Solyc02g090390.2.1
Solyc01g100420.2.1
Solyc03g115560.2.1
Solyc12g007320.1.1
Solyc01g094250.2.1
Solyc07g056570.1.1
Solyc07g009260.2.1
Solyc08g067510.1.1
Solyc01g079170.2.1
Solyc04g008950.2.1
Solyc10g008040.2.1
Solyc02g086650.2.1
Solyc03g116400.2.1
Solyc02g091130.2.1
Solyc11g021020.1.1
Solyc08g036630.1.1
Solyc03g119260.2.1
Solyc03g116430.1.1
BC2
ON2
BCm2
BCm3
BC1
ON1
ON3
BCm1
BC3
ONm3
ONm1
ONm2
Pim1
Pi2
Pi1
Pim3
Pim2
Pi3

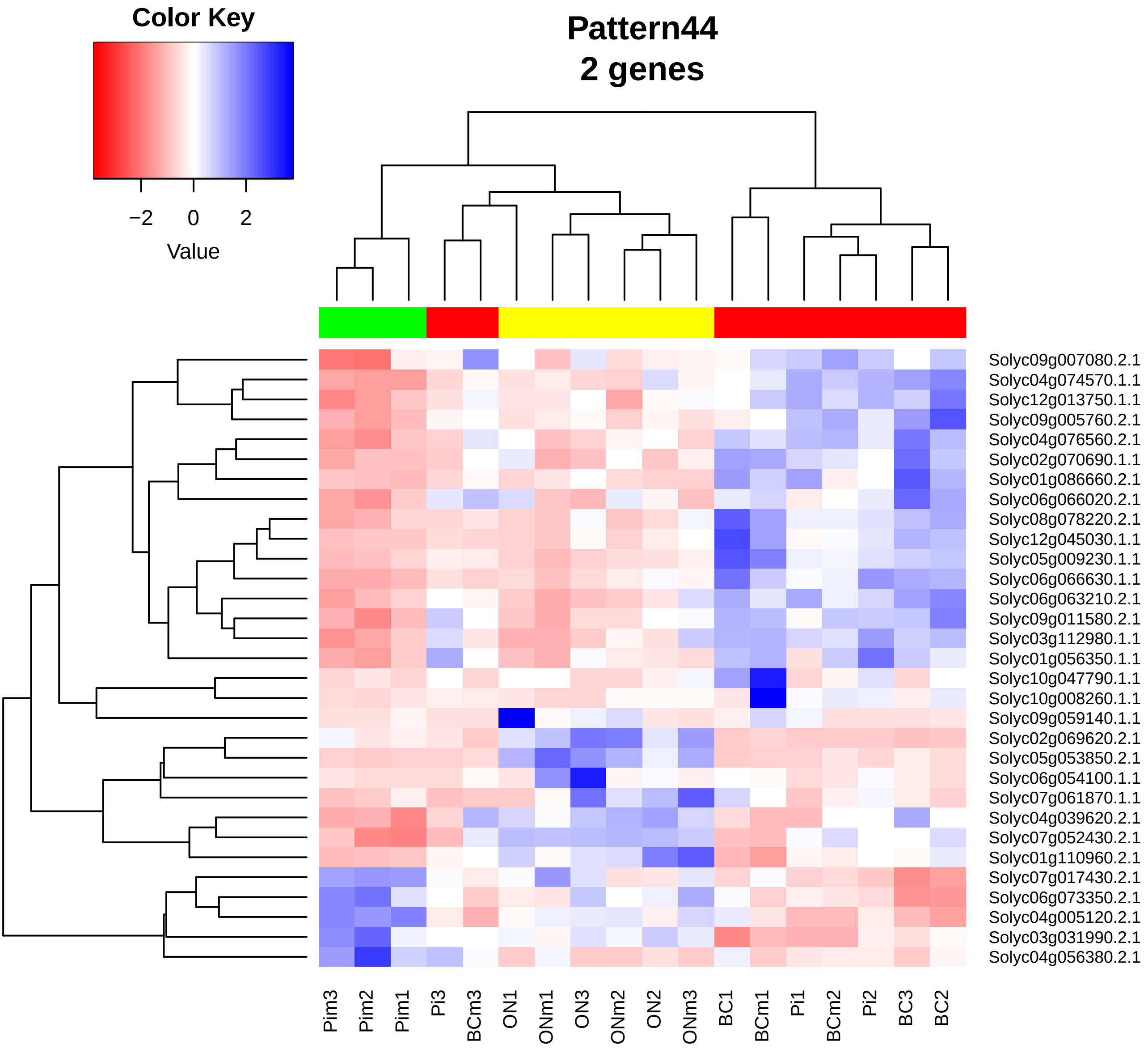
Color Key
−2
0
2
Value
Pattern44
2 genes
Solyc09g007080.2.1
Solyc04g074570.1.1
Solyc12g013750.1.1
Solyc09g005760.2.1
Solyc04g076560.2.1
Solyc02g070690.1.1
Solyc01g086660.2.1
Solyc06g066020.2.1
Solyc08g078220.2.1
Solyc12g045030.1.1
Solyc05g009230.1.1
Solyc06g066630.1.1
Solyc06g063210.2.1
Solyc09g011580.2.1
Solyc03g112980.1.1
Solyc01g056350.1.1
Solyc10g047790.1.1
Solyc10g008260.1.1
Solyc09g059140.1.1
Solyc02g069620.2.1
Solyc05g053850.2.1
Solyc06g054100.1.1
Solyc07g061870.1.1
Solyc04g039620.2.1
Solyc07g052430.2.1
Solyc01g110960.2.1
Solyc07g017430.2.1
Solyc06g073350.2.1
Solyc04g005120.2.1
Solyc03g031990.2.1
Solyc04g056380.2.1
Pim3
Pim2
Pim1
Pi3
BCm3
ON1
ONm1
ON3
ONm2
ON2
ONm3
BC1
BCm1
Pi1
BCm2
Pi2
BC3
BC2

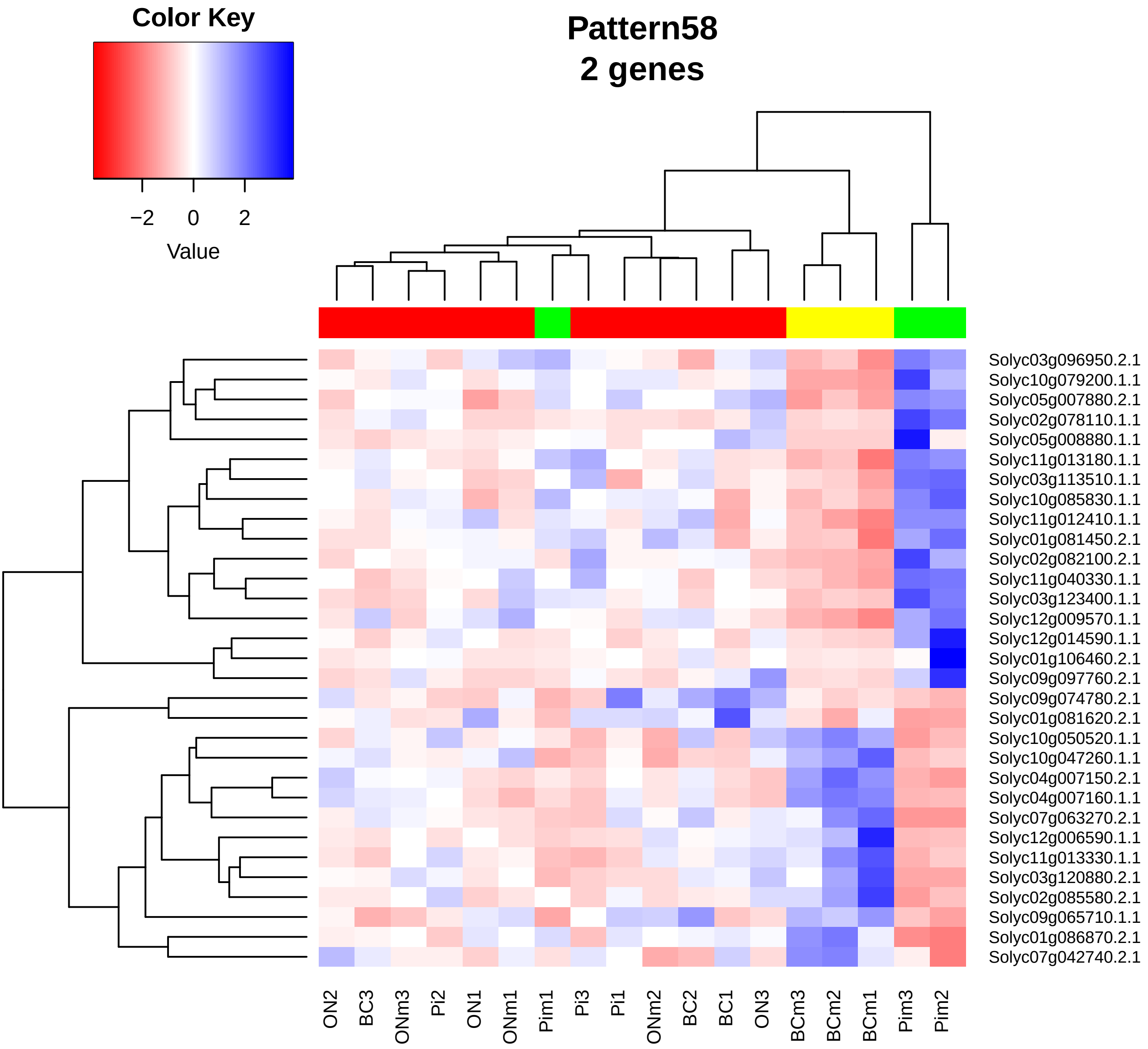
Color Key
−2
0
2
Value
Pattern58
2 genes
Solyc03g096950.2.1
Solyc10g079200.1.1
Solyc05g007880.2.1
Solyc02g078110.1.1
Solyc05g008880.1.1
Solyc11g013180.1.1
Solyc03g113510.1.1
Solyc10g085830.1.1
Solyc11g012410.1.1
Solyc01g081450.2.1
Solyc02g082100.2.1
Solyc11g040330.1.1
Solyc03g123400.1.1
Solyc12g009570.1.1
Solyc12g014590.1.1
Solyc01g106460.2.1
Solyc09g097760.2.1
Solyc09g074780.2.1
Solyc01g081620.2.1
Solyc10g050520.1.1
Solyc10g047260.1.1
Solyc04g007150.2.1
Solyc04g007160.1.1
Solyc07g063270.2.1
Solyc12g006590.1.1
Solyc11g013330.1.1
Solyc03g120880.2.1
Solyc02g085580.2.1
Solyc09g065710.1.1
Solyc01g086870.2.1
Solyc07g042740.2.1
ON2
BC3
ONm3
Pi2
ON1
ONm1
Pim1
Pi3
Pi1
ONm2
BC2
BC1
ON3
BCm3
BCm2
BCm1
Pim3
Pim2

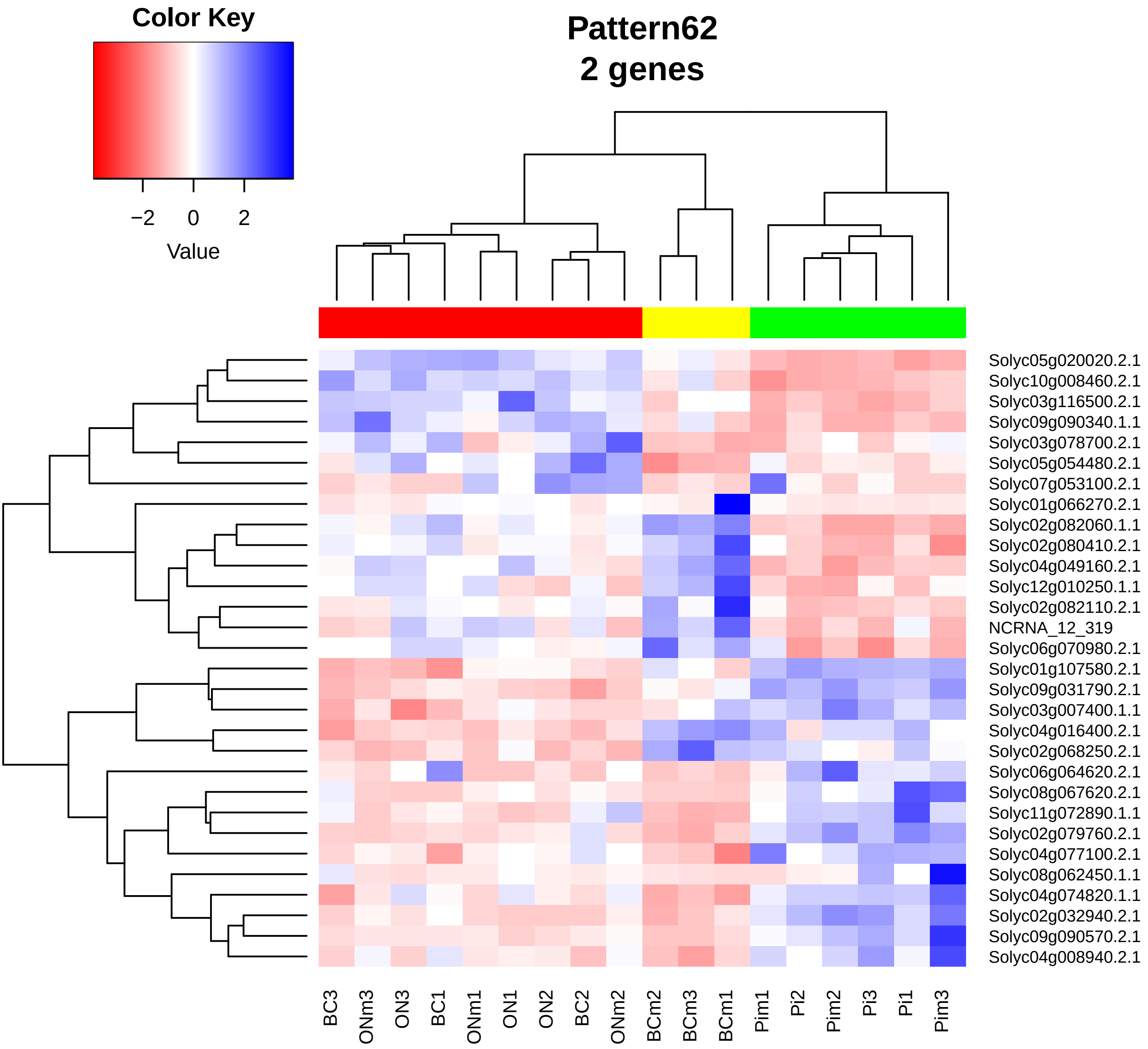

Color Key
−2
0
2
Value
Pattern62
2 genes
Solyc05g020020.2.1
Solyc10g008460.2.1
Solyc03g116500.2.1
Solyc09g090340.1.1
Solyc03g078700.2.1
Solyc05g054480.2.1
Solyc07g053100.2.1
Solyc01g066270.2.1
Solyc02g082060.1.1
Solyc02g080410.2.1
Solyc04g049160.2.1
Solyc12g010250.1.1
Solyc02g082110.2.1
NCRNA_12_319
Solyc06g070980.2.1
Solyc01g107580.2.1
Solyc09g031790.2.1
Solyc03g007400.1.1
Solyc04g016400.2.1
Solyc02g068250.2.1
Solyc06g064620.2.1
Solyc08g067620.2.1
Solyc11g072890.1.1
Solyc02g079760.2.1
Solyc04g077100.2.1
Solyc08g062450.1.1
Solyc04g074820.1.1
Solyc02g032940.2.1
Solyc09g090570.2.1
Solyc04g008940.2.1
BC3
ONm3
ON3
BC1
ONm1
ON1
ON2
BC2
ONm2
BCm2
BCm3
BCm1
Pim1
Pi2
Pim2
Pi3
Pi1
Pim3

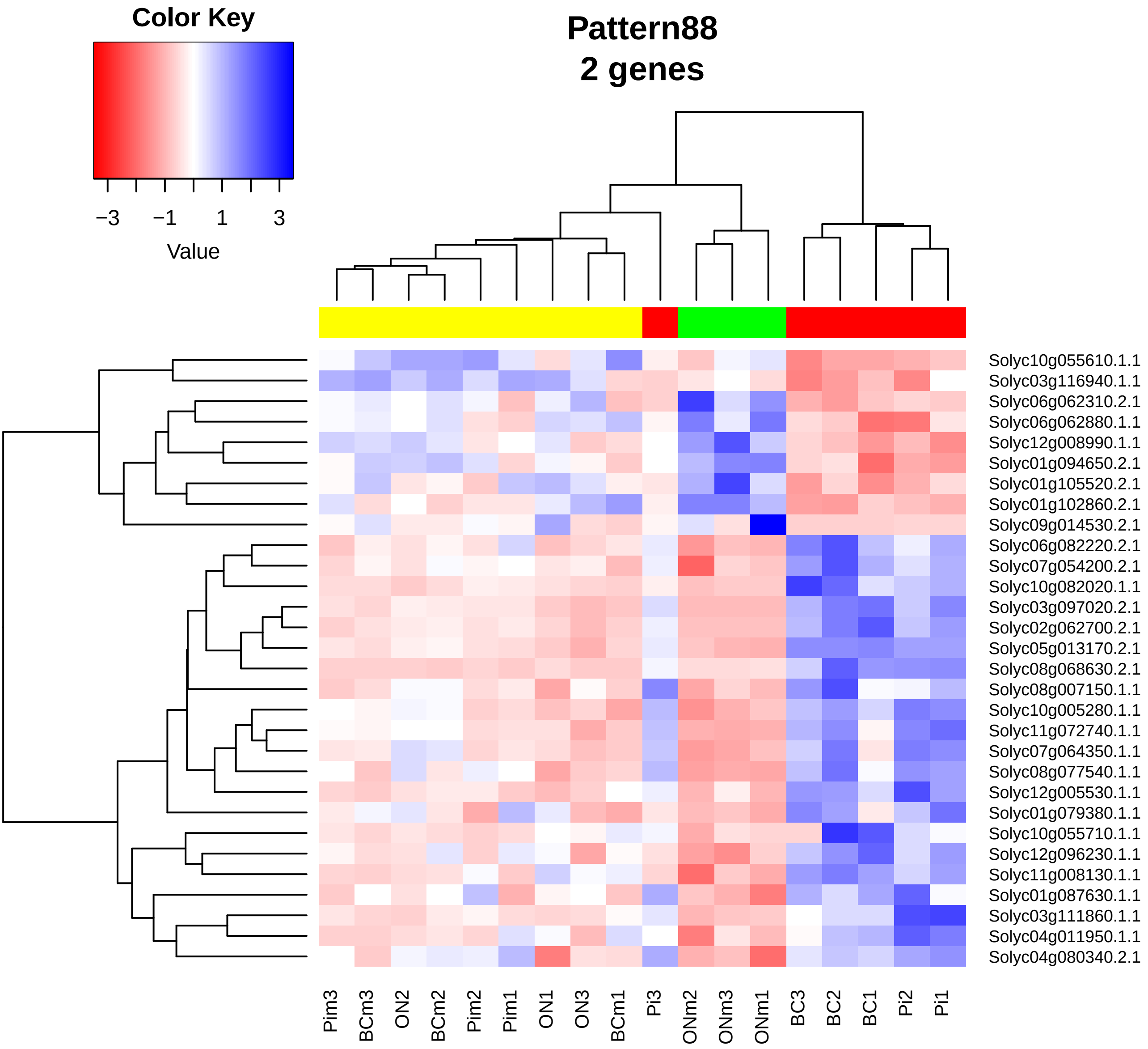

Color Key
−3
−1
1
3
Value
Pattern88
2 genes
Solyc10g055610.1.1
Solyc03g116940.1.1
Solyc06g062310.2.1
Solyc06g062880.1.1
Solyc12g008990.1.1
Solyc01g094650.2.1
Solyc01g105520.2.1
Solyc01g102860.2.1
Solyc09g014530.2.1
Solyc06g082220.2.1
Solyc07g054200.2.1
Solyc10g082020.1.1
Solyc03g097020.2.1
Solyc02g062700.2.1
Solyc05g013170.2.1
Solyc08g068630.2.1
Solyc08g007150.1.1
Solyc10g005280.1.1
Solyc11g072740.1.1
Solyc07g064350.1.1
Solyc08g077540.1.1
Solyc12g005530.1.1
Solyc01g079380.1.1
Solyc10g055710.1.1
Solyc12g096230.1.1
Solyc11g008130.1.1
Solyc01g087630.1.1
Solyc03g111860.1.1
Solyc04g011950.1.1
Solyc04g080340.2.1
Pim3
BCm3
ON2
BCm2
Pim2
Pim1
ON1
ON3
BCm1
Pi3
ONm2
ONm3
ONm1
BC3
BC2
BC1
Pi2
Pi1

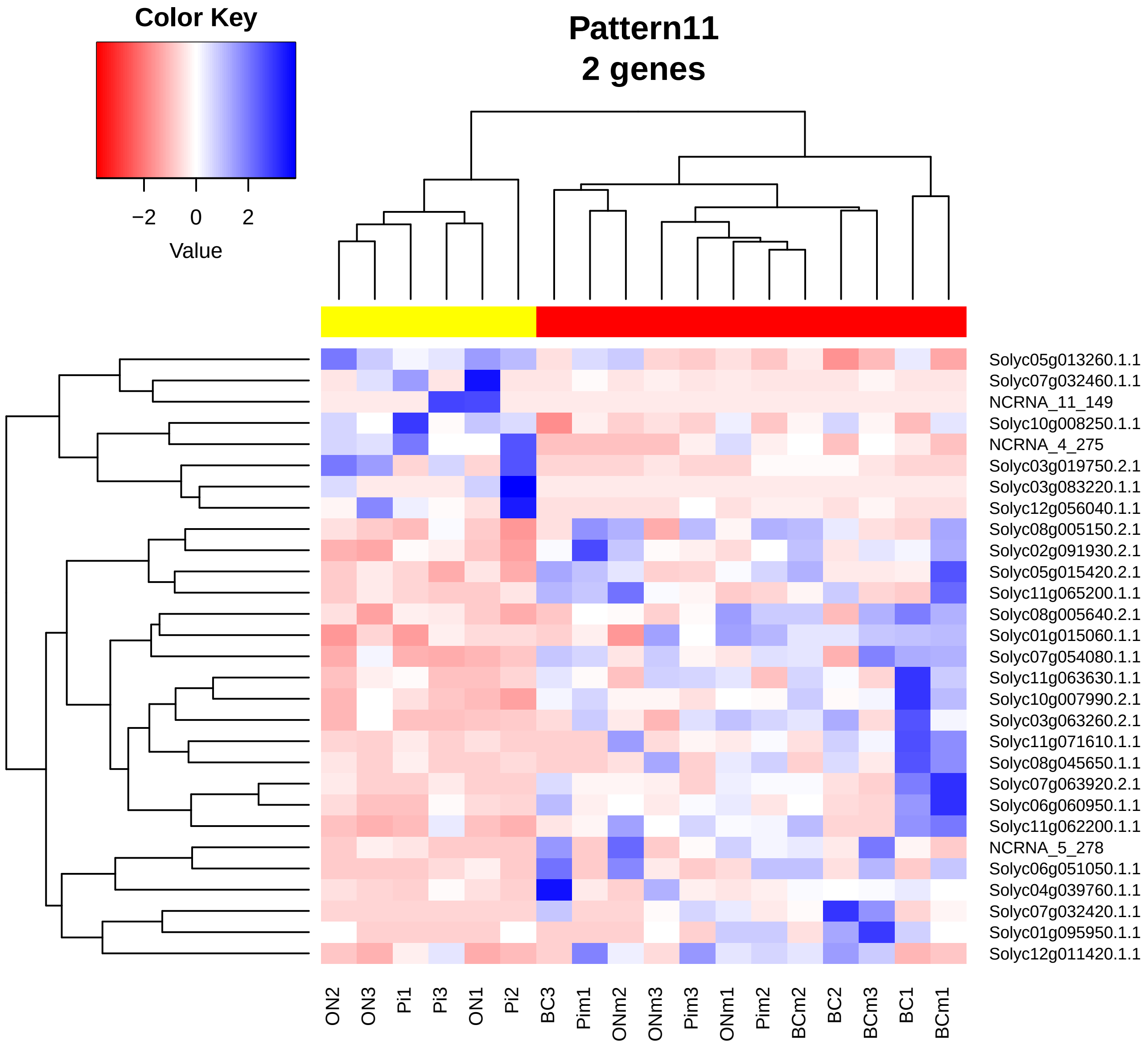
Color Key
−2
0
2
Value
Pattern11
2 genes
Solyc05g013260.1.1
Solyc07g032460.1.1
NCRNA_11_149
Solyc10g008250.1.1
NCRNA_4_275
Solyc03g019750.2.1
Solyc03g083220.1.1
Solyc12g056040.1.1
Solyc08g005150.2.1
Solyc02g091930.2.1
Solyc05g015420.2.1
Solyc11g065200.1.1
Solyc08g005640.2.1
Solyc01g015060.1.1
Solyc07g054080.1.1
Solyc11g063630.1.1
Solyc10g007990.2.1
Solyc03g063260.2.1
Solyc11g071610.1.1
Solyc08g045650.1.1
Solyc07g063920.2.1
Solyc06g060950.1.1
Solyc11g062200.1.1
NCRNA_5_278
Solyc06g051050.1.1
Solyc04g039760.1.1
Solyc07g032420.1.1
Solyc01g095950.1.1
Solyc12g011420.1.1
ON2
ON3
Pi1
Pi3
ON1
Pi2
BC3
Pim1
ONm2
ONm3
Pim3
ONm1
Pim2
BCm2
BC2
BCm3
BC1
BCm1

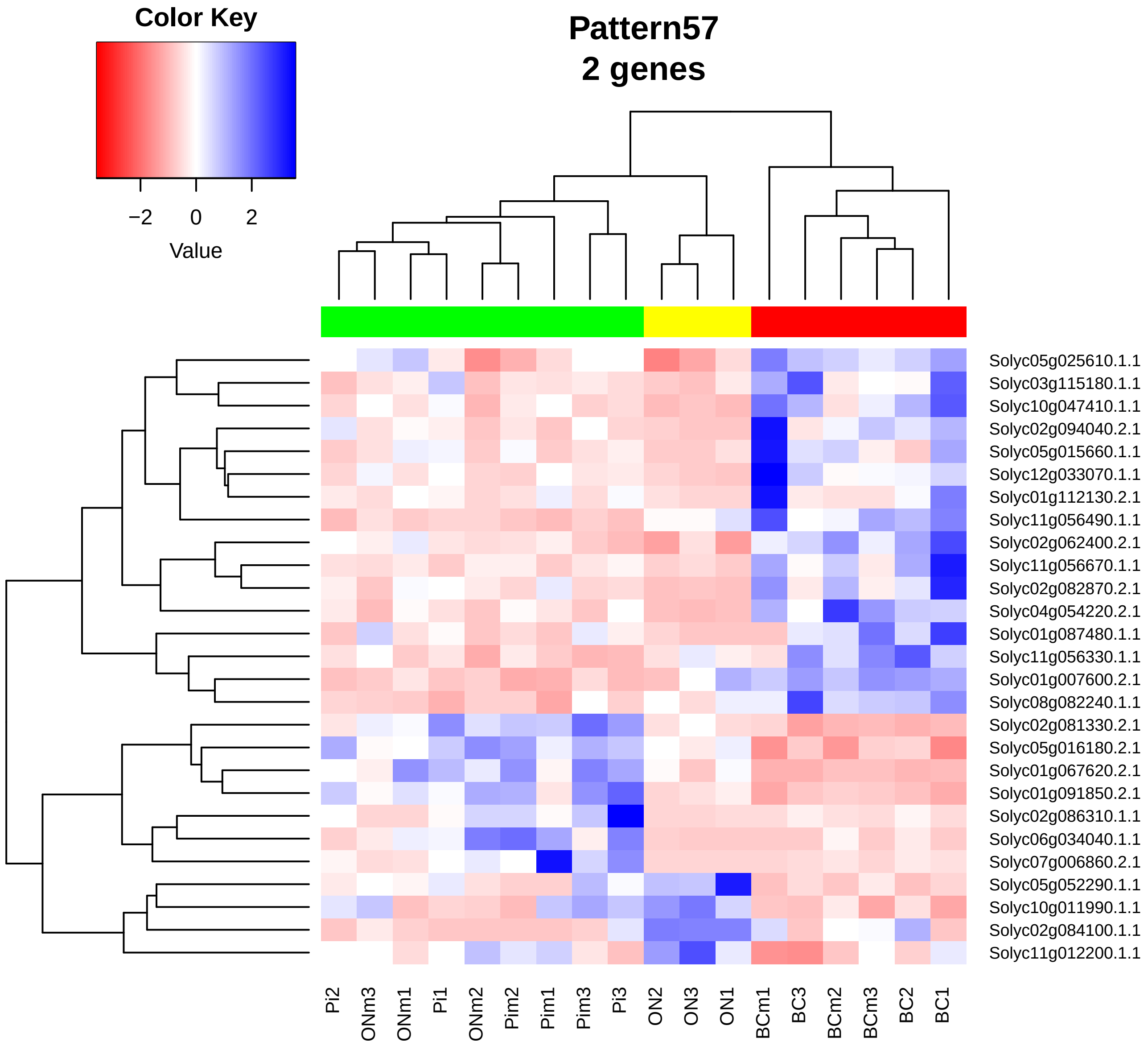
Color Key
−2
0
2
Value
Pattern57
2 genes
Solyc05g025610.1.1
Solyc03g115180.1.1
Solyc10g047410.1.1
Solyc02g094040.2.1
Solyc05g015660.1.1
Solyc12g033070.1.1
Solyc01g112130.2.1
Solyc11g056490.1.1
Solyc02g062400.2.1
Solyc11g056670.1.1
Solyc02g082870.2.1
Solyc04g054220.2.1
Solyc01g087480.1.1
Solyc11g056330.1.1
Solyc01g007600.2.1
Solyc08g082240.1.1
Solyc02g081330.2.1
Solyc05g016180.2.1
Solyc01g067620.2.1
Solyc01g091850.2.1
Solyc02g086310.1.1
Solyc06g034040.1.1
Solyc07g006860.2.1
Solyc05g052290.1.1
Solyc10g011990.1.1
Solyc02g084100.1.1
Solyc11g012200.1.1
Pi2
ONm3
ONm1
Pi1
ONm2
Pim2
Pim1
Pim3
Pi3
ON2
ON3
ON1
BCm1
BC3
BCm2
BCm3
BC2
BC1

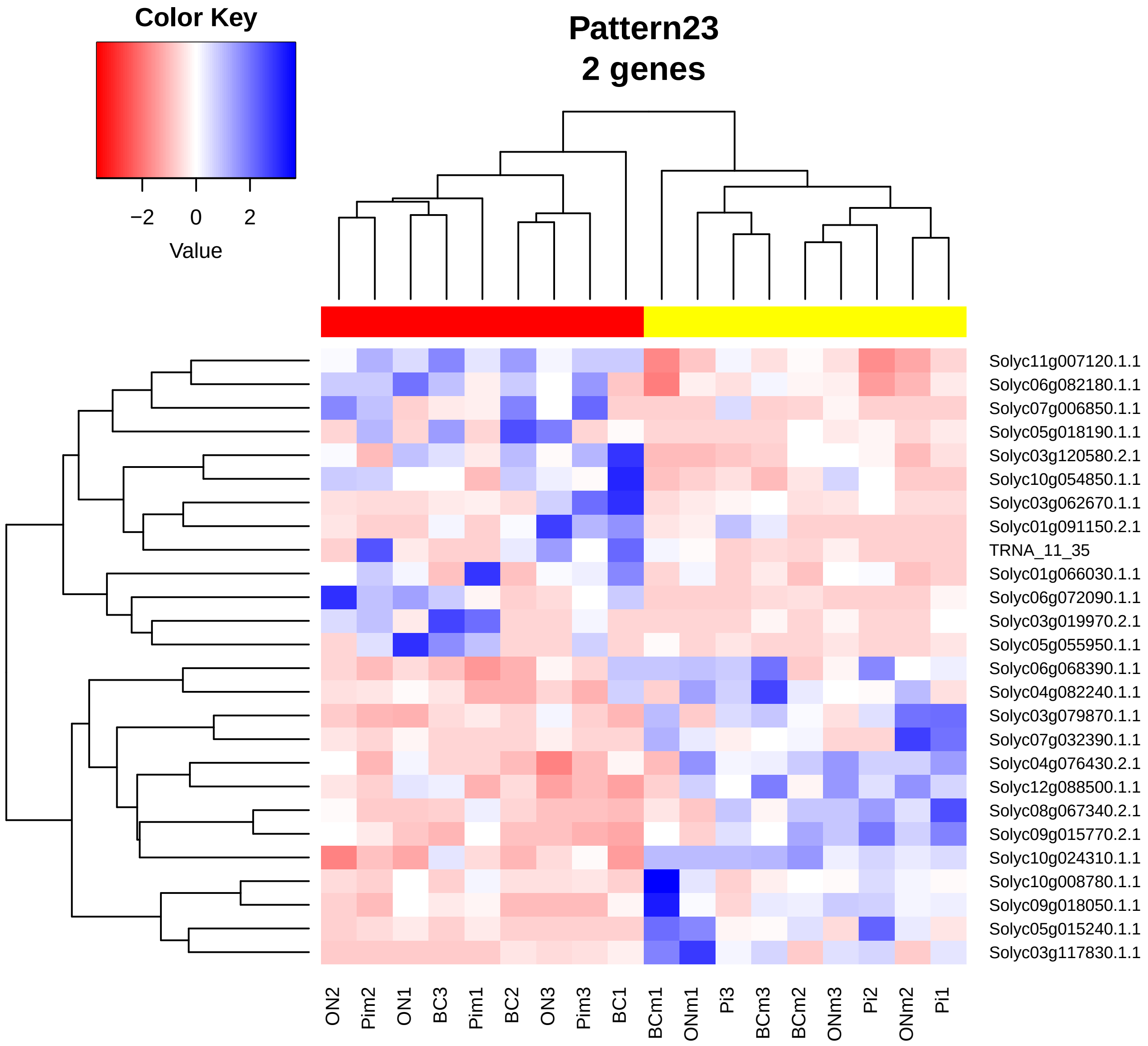

Color Key
−2
0
2
Value
Pattern23
2 genes
Solyc11g007120.1.1
Solyc06g082180.1.1
Solyc07g006850.1.1
Solyc05g018190.1.1
Solyc03g120580.2.1
Solyc10g054850.1.1
Solyc03g062670.1.1
Solyc01g091150.2.1
TRNA_11_35
Solyc01g066030.1.1
Solyc06g072090.1.1
Solyc03g019970.2.1
Solyc05g055950.1.1
Solyc06g068390.1.1
Solyc04g082240.1.1
Solyc03g079870.1.1
Solyc07g032390.1.1
Solyc04g076430.2.1
Solyc12g088500.1.1
Solyc08g067340.2.1
Solyc09g015770.2.1
Solyc10g024310.1.1
Solyc10g008780.1.1
Solyc09g018050.1.1
Solyc05g015240.1.1
Solyc03g117830.1.1
ON2
Pim2
ON1
BC3
Pim1
BC2
ON3
Pim3
BC1
BCm1
ONm1
Pi3
BCm3
BCm2
ONm3
Pi2
ONm2
Pi1

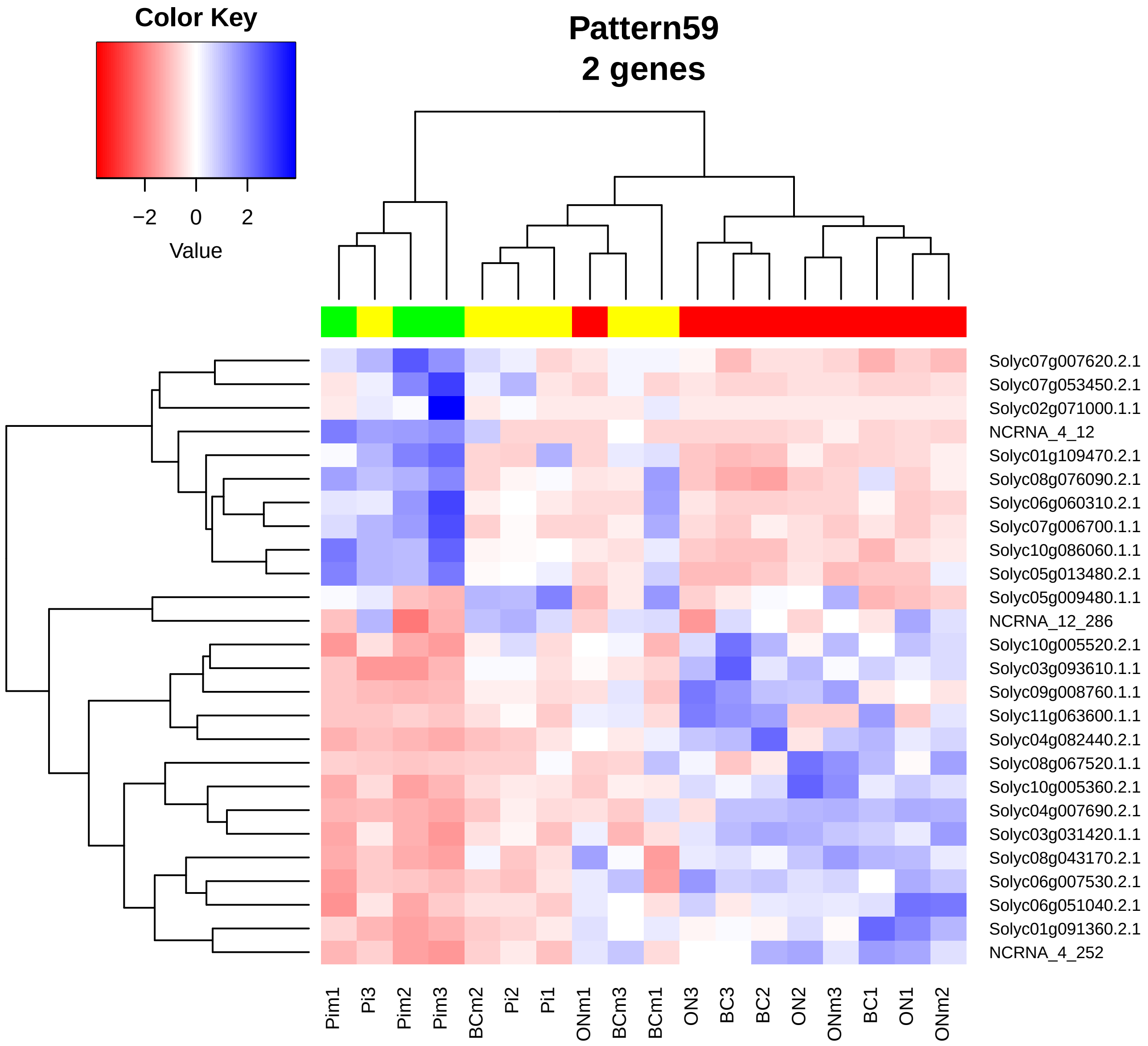

Color Key
−2
0
2
Value
Pattern59
2 genes
Solyc07g007620.2.1
Solyc07g053450.2.1
Solyc02g071000.1.1
NCRNA_4_12
Solyc01g109470.2.1
Solyc08g076090.2.1
Solyc06g060310.2.1
Solyc07g006700.1.1
Solyc10g086060.1.1
Solyc05g013480.2.1
Solyc05g009480.1.1
NCRNA_12_286
Solyc10g005520.2.1
Solyc03g093610.1.1
Solyc09g008760.1.1
Solyc11g063600.1.1
Solyc04g082440.2.1
Solyc08g067520.1.1
Solyc10g005360.2.1
Solyc04g007690.2.1
Solyc03g031420.1.1
Solyc08g043170.2.1
Solyc06g007530.2.1
Solyc06g051040.2.1
Solyc01g091360.2.1
NCRNA_4_252
Pim1
Pi3
Pim2
Pim3
BCm2
Pi2
Pi1
ONm1
BCm3
BCm1
ON3
BC3
BC2
ON2
ONm3
BC1
ON1
ONm2

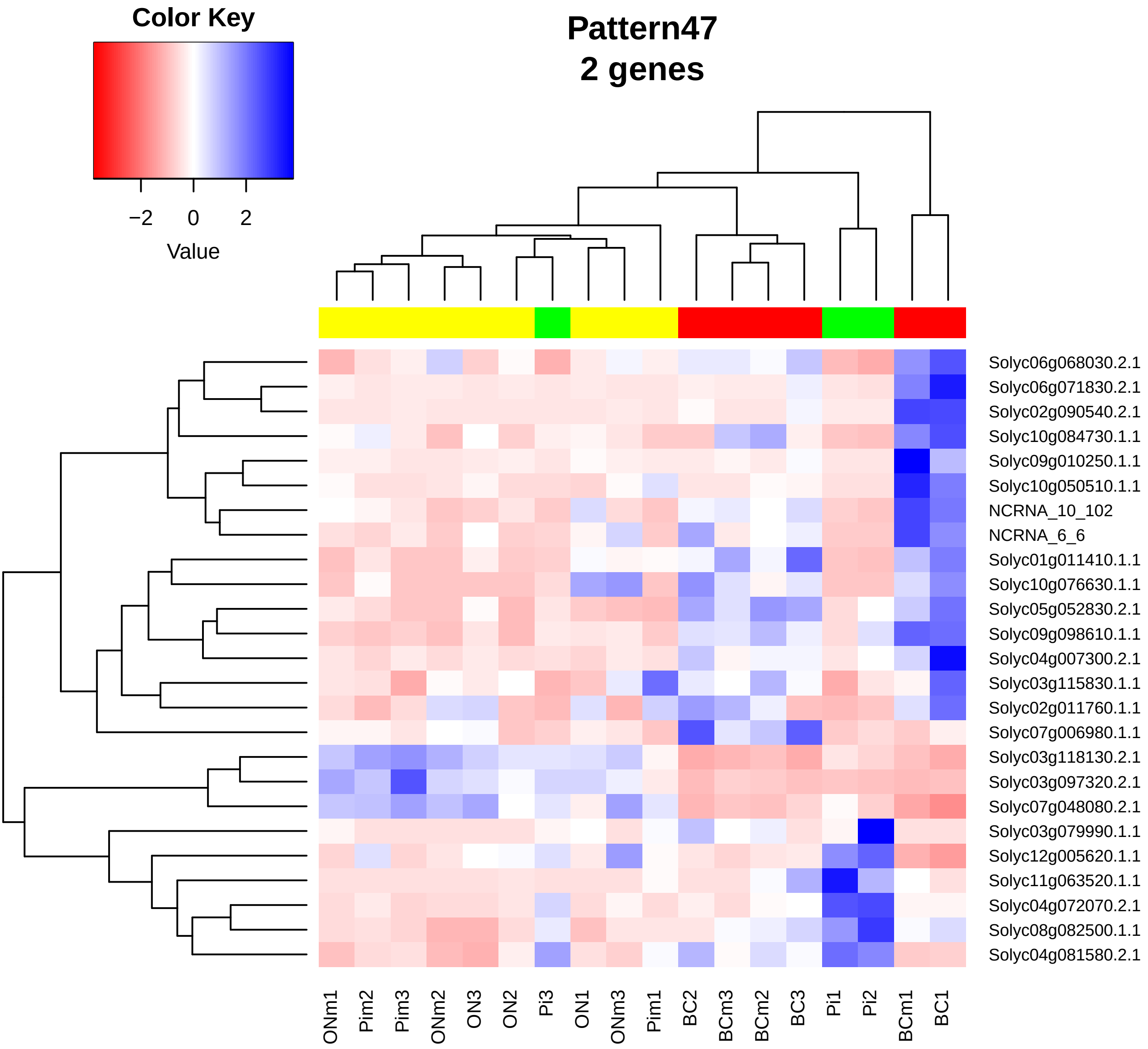

Color Key
−2
0
2
Value
Pattern47
2 genes
Solyc06g068030.2.1
Solyc06g071830.2.1
Solyc02g090540.2.1
Solyc10g084730.1.1
Solyc09g010250.1.1
Solyc10g050510.1.1
NCRNA_10_102
NCRNA_6_6
Solyc01g011410.1.1
Solyc10g076630.1.1
Solyc05g052830.2.1
Solyc09g098610.1.1
Solyc04g007300.2.1
Solyc03g115830.1.1
Solyc02g011760.1.1
Solyc07g006980.1.1
Solyc03g118130.2.1
Solyc03g097320.2.1
Solyc07g048080.2.1
Solyc03g079990.1.1
Solyc12g005620.1.1
Solyc11g063520.1.1
Solyc04g072070.2.1
Solyc08g082500.1.1
Solyc04g081580.2.1
ONm1
Pim2
Pim3
ONm2
ON3
ON2
Pi3
ON1
ONm3
Pim1
BC2
BCm3
BCm2
BC3
Pi1
Pi2
BCm1
BC1

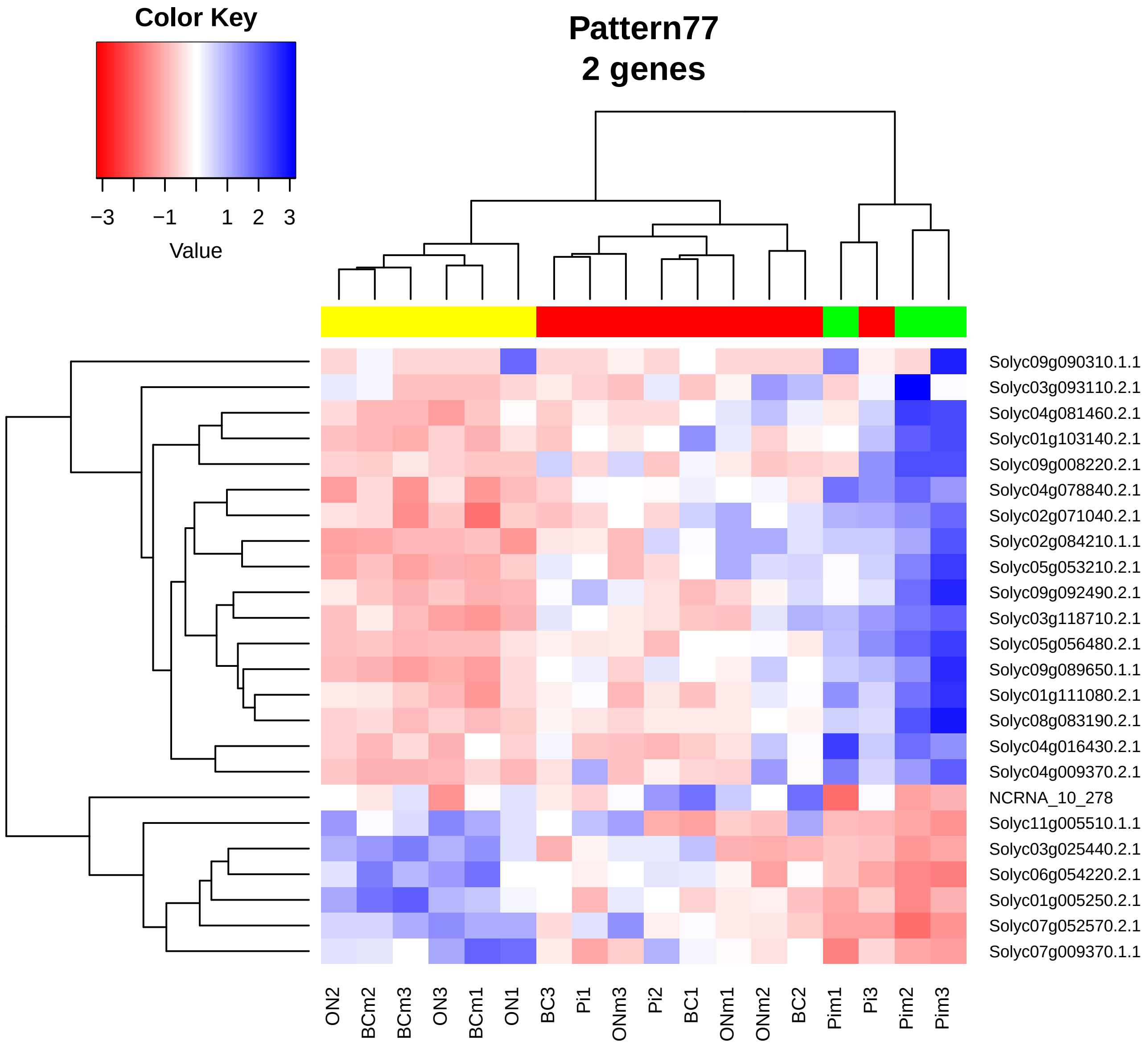
Color Key
−3
−1
1
2
3
Value
Pattern77
2 genes
Solyc09g090310.1.1
Solyc03g093110.2.1
Solyc04g081460.2.1
Solyc01g103140.2.1
Solyc09g008220.2.1
Solyc04g078840.2.1
Solyc02g071040.2.1
Solyc02g084210.1.1
Solyc05g053210.2.1
Solyc09g092490.2.1
Solyc03g118710.2.1
Solyc05g056480.2.1
Solyc09g089650.1.1
Solyc01g111080.2.1
Solyc08g083190.2.1
Solyc04g016430.2.1
Solyc04g009370.2.1
NCRNA_10_278
Solyc11g005510.1.1
Solyc03g025440.2.1
Solyc06g054220.2.1
Solyc01g005250.2.1
Solyc07g052570.2.1
Solyc07g009370.1.1
ON2
BCm2
BCm3
ON3
BCm1
ON1
BC3
Pi1
ONm3
Pi2
BC1
ONm1
ONm2
BC2
Pim1
Pi3
Pim2
Pim3

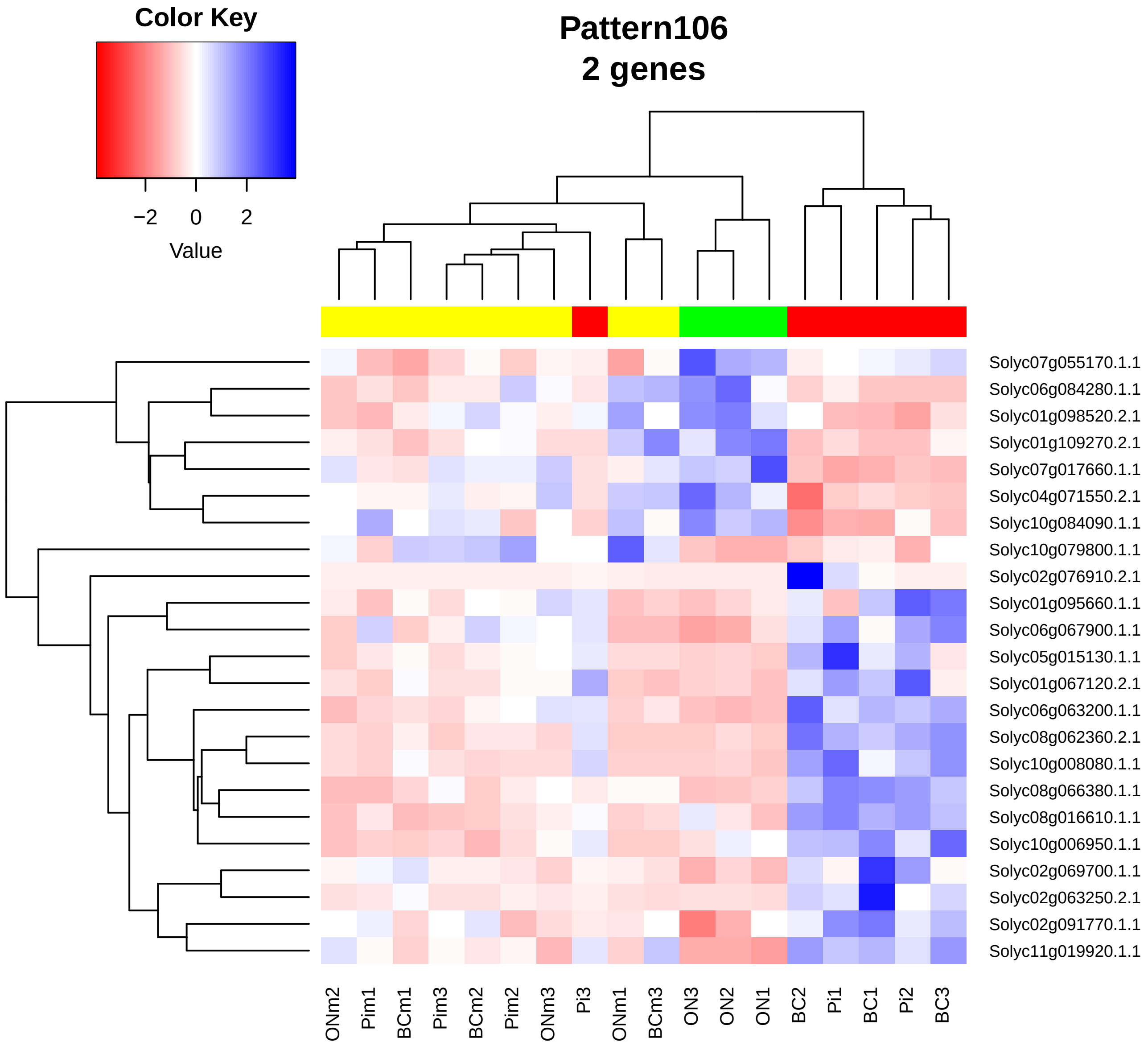
Color Key
−2
0
2
Value
Pattern106
2 genes
Solyc07g055170.1.1
Solyc06g084280.1.1
Solyc01g098520.2.1
Solyc01g109270.2.1
Solyc07g017660.1.1
Solyc04g071550.2.1
Solyc10g084090.1.1
Solyc10g079800.1.1
Solyc02g076910.2.1
Solyc01g095660.1.1
Solyc06g067900.1.1
Solyc05g015130.1.1
Solyc01g067120.2.1
Solyc06g063200.1.1
Solyc08g062360.2.1
Solyc10g008080.1.1
Solyc08g066380.1.1
Solyc08g016610.1.1
Solyc10g006950.1.1
Solyc02g069700.1.1
Solyc02g063250.2.1
Solyc02g091770.1.1
Solyc11g019920.1.1
ONm2
Pim1
BCm1
Pim3
BCm2
Pim2
ONm3
Pi3
ONm1
BCm3
ON3
ON2
ON1
BC2
Pi1
BC1
Pi2
BC3

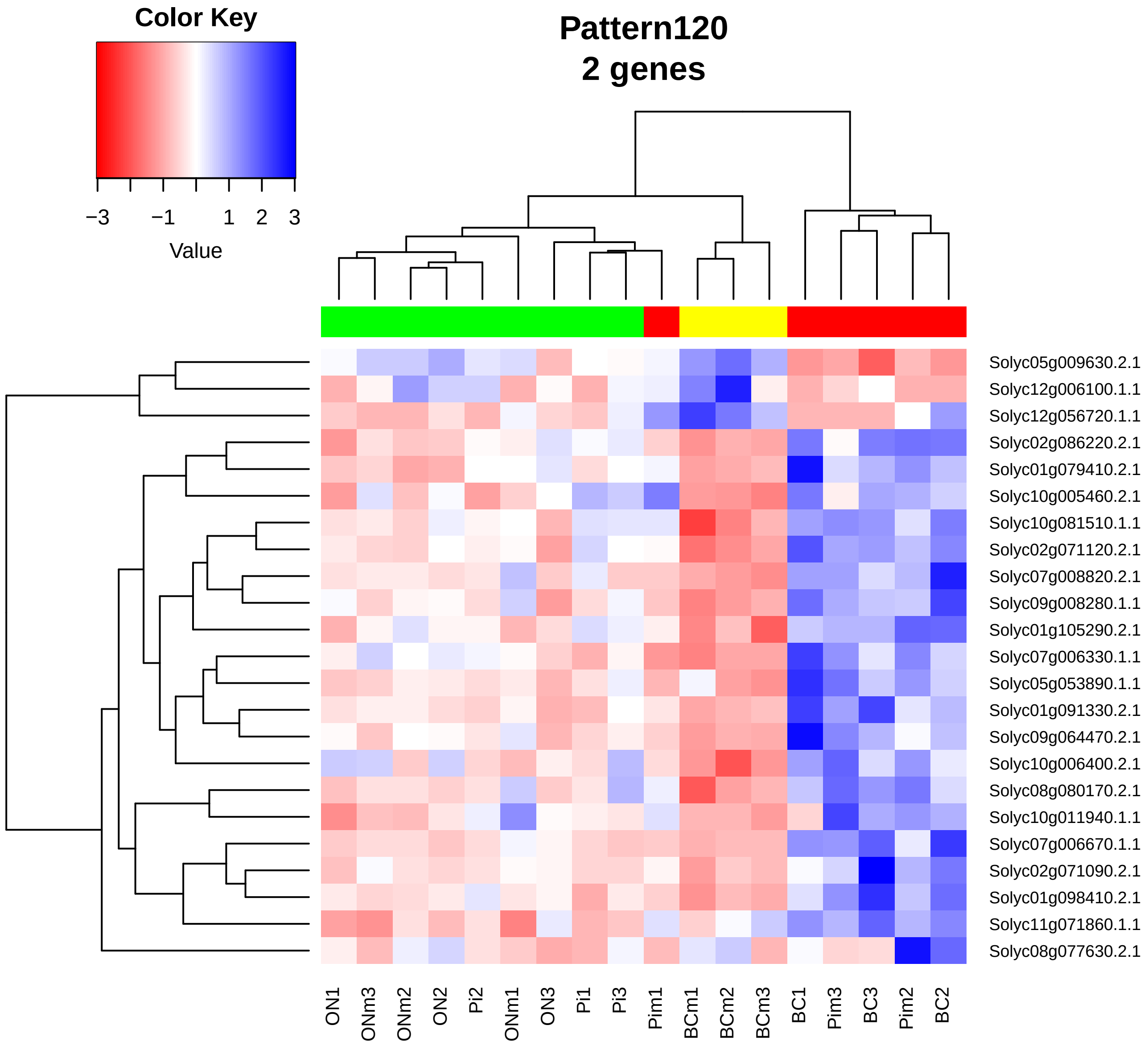
Color Key
−3
−1
1
2
3
Value
Pattern120
2 genes
Solyc05g009630.2.1
Solyc12g006100.1.1
Solyc12g056720.1.1
Solyc02g086220.2.1
Solyc01g079410.2.1
Solyc10g005460.2.1
Solyc10g081510.1.1
Solyc02g071120.2.1
Solyc07g008820.2.1
Solyc09g008280.1.1
Solyc01g105290.2.1
Solyc07g006330.1.1
Solyc05g053890.1.1
Solyc01g091330.2.1
Solyc09g064470.2.1
Solyc10g006400.2.1
Solyc08g080170.2.1
Solyc10g011940.1.1
Solyc07g006670.1.1
Solyc02g071090.2.1
Solyc01g098410.2.1
Solyc11g071860.1.1
Solyc08g077630.2.1
ON1
ONm3
ONm2
ON2
Pi2
ONm1
ON3
Pi1
Pi3
Pim1
BCm1
BCm2
BCm3
BC1
Pim3
BC3
Pim2
BC2

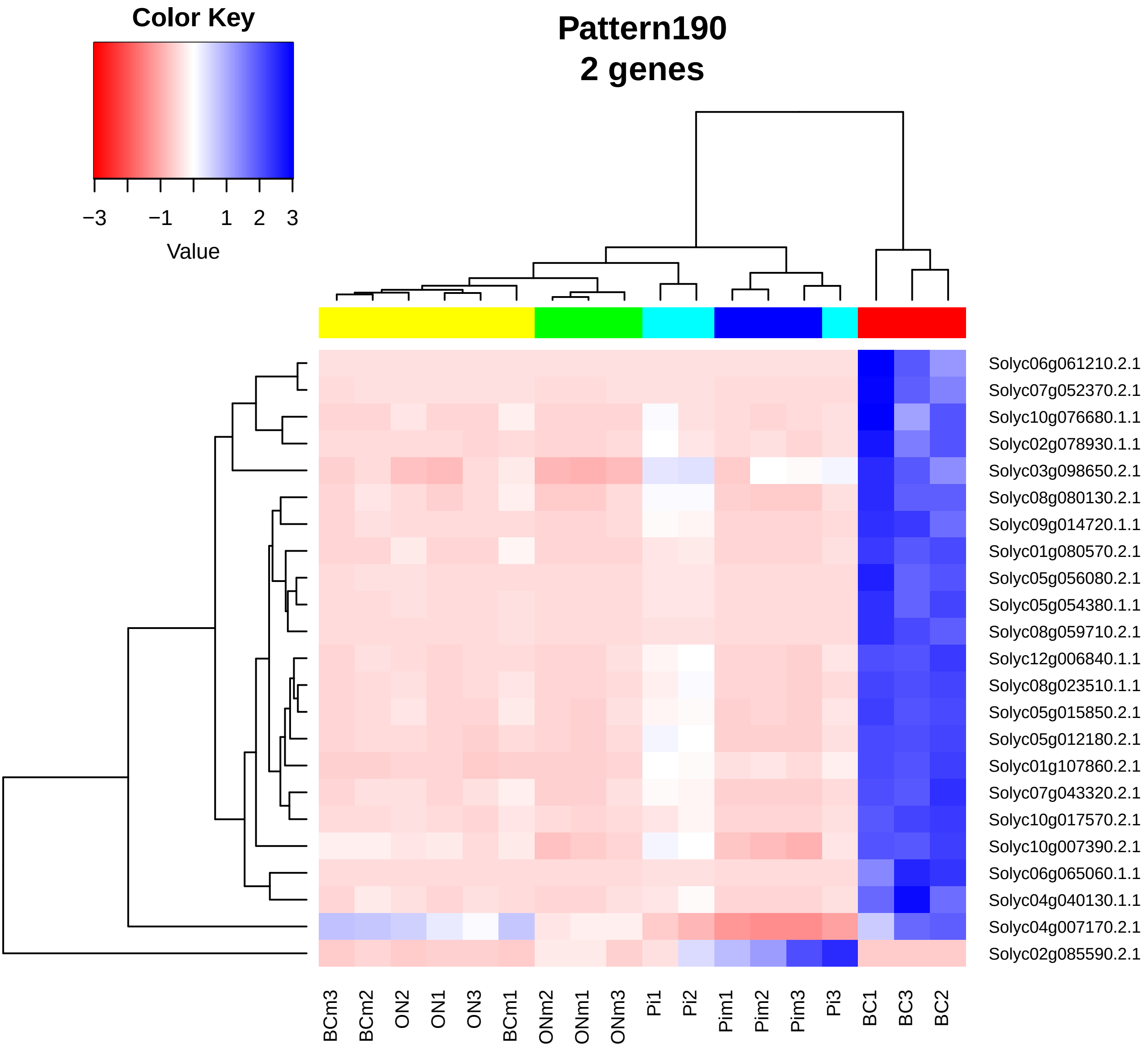
Color Key
−3
−1
1
2
3
Value
Pattern190
2 genes
Solyc06g061210.2.1
Solyc07g052370.2.1
Solyc10g076680.1.1
Solyc02g078930.1.1
Solyc03g098650.2.1
Solyc08g080130.2.1
Solyc09g014720.1.1
Solyc01g080570.2.1
Solyc05g056080.2.1
Solyc05g054380.1.1
Solyc08g059710.2.1
Solyc12g006840.1.1
Solyc08g023510.1.1
Solyc05g015850.2.1
Solyc05g012180.2.1
Solyc01g107860.2.1
Solyc07g043320.2.1
Solyc10g017570.2.1
Solyc10g007390.2.1
Solyc06g065060.1.1
Solyc04g040130.1.1
Solyc04g007170.2.1
Solyc02g085590.2.1
BCm3
BCm2
ON2
ON1
ON3
BCm1
ONm2
ONm1
ONm3
Pi1
Pi2
Pim1
Pim2
Pim3
Pi3
BC1
BC3
BC2

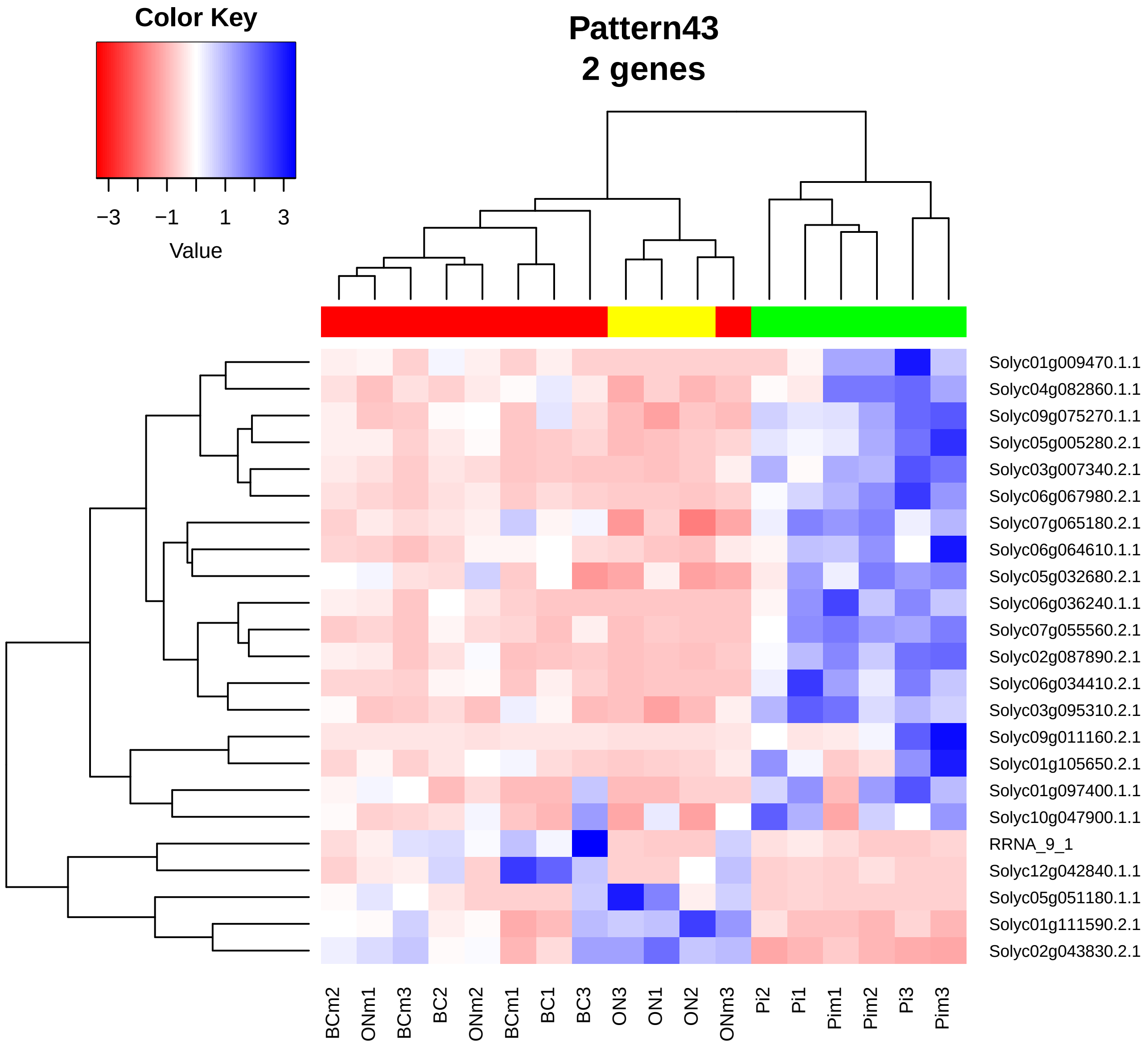

Color Key
−3
−1
1
3
Value
Pattern43
2 genes
Solyc01g009470.1.1
Solyc04g082860.1.1
Solyc09g075270.1.1
Solyc05g005280.2.1
Solyc03g007340.2.1
Solyc06g067980.2.1
Solyc07g065180.2.1
Solyc06g064610.1.1
Solyc05g032680.2.1
Solyc06g036240.1.1
Solyc07g055560.2.1
Solyc02g087890.2.1
Solyc06g034410.2.1
Solyc03g095310.2.1
Solyc09g011160.2.1
Solyc01g105650.2.1
Solyc01g097400.1.1
Solyc10g047900.1.1
RRNA_9_1
Solyc12g042840.1.1
Solyc05g051180.1.1
Solyc01g111590.2.1
Solyc02g043830.2.1
BCm2
ONm1
BCm3
BC2
ONm2
BCm1
BC1
BC3
ON3
ON1
ON2
ONm3
Pi2
Pi1
Pim1
Pim2
Pi3
Pim3

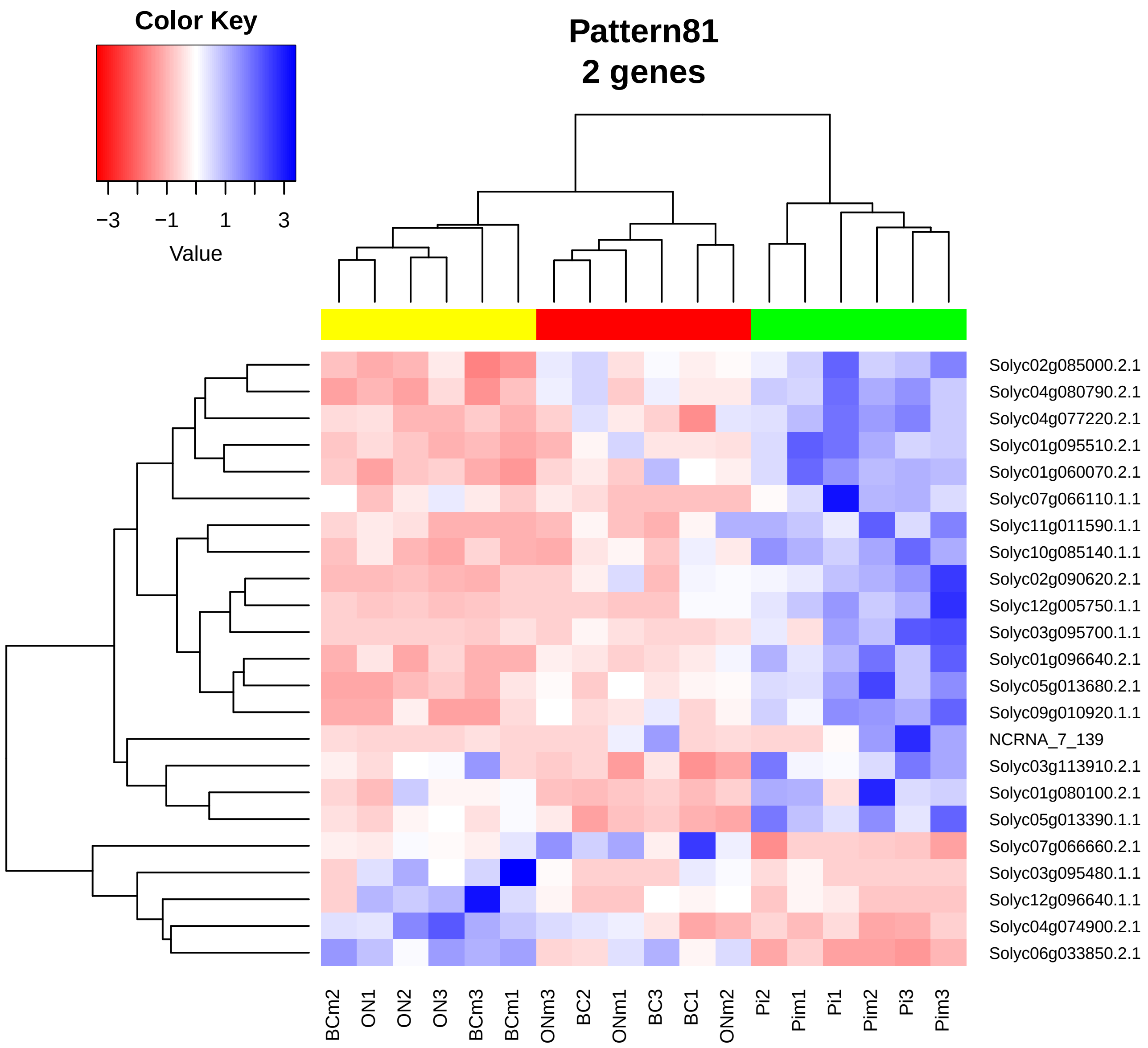

Color Key
−3
−1
1
3
Value
Pattern81
2 genes
Solyc02g085000.2.1
Solyc04g080790.2.1
Solyc04g077220.2.1
Solyc01g095510.2.1
Solyc01g060070.2.1
Solyc07g066110.1.1
Solyc11g011590.1.1
Solyc10g085140.1.1
Solyc02g090620.2.1
Solyc12g005750.1.1
Solyc03g095700.1.1
Solyc01g096640.2.1
Solyc05g013680.2.1
Solyc09g010920.1.1
NCRNA_7_139
Solyc03g113910.2.1
Solyc01g080100.2.1
Solyc05g013390.1.1
Solyc07g066660.2.1
Solyc03g095480.1.1
Solyc12g096640.1.1
Solyc04g074900.2.1
Solyc06g033850.2.1
BCm2
ON1
ON2
ON3
BCm3
BCm1
ONm3
BC2
ONm1
BC3
BC1
ONm2
Pi2
Pim1
Pi1
Pim2
Pi3
Pim3

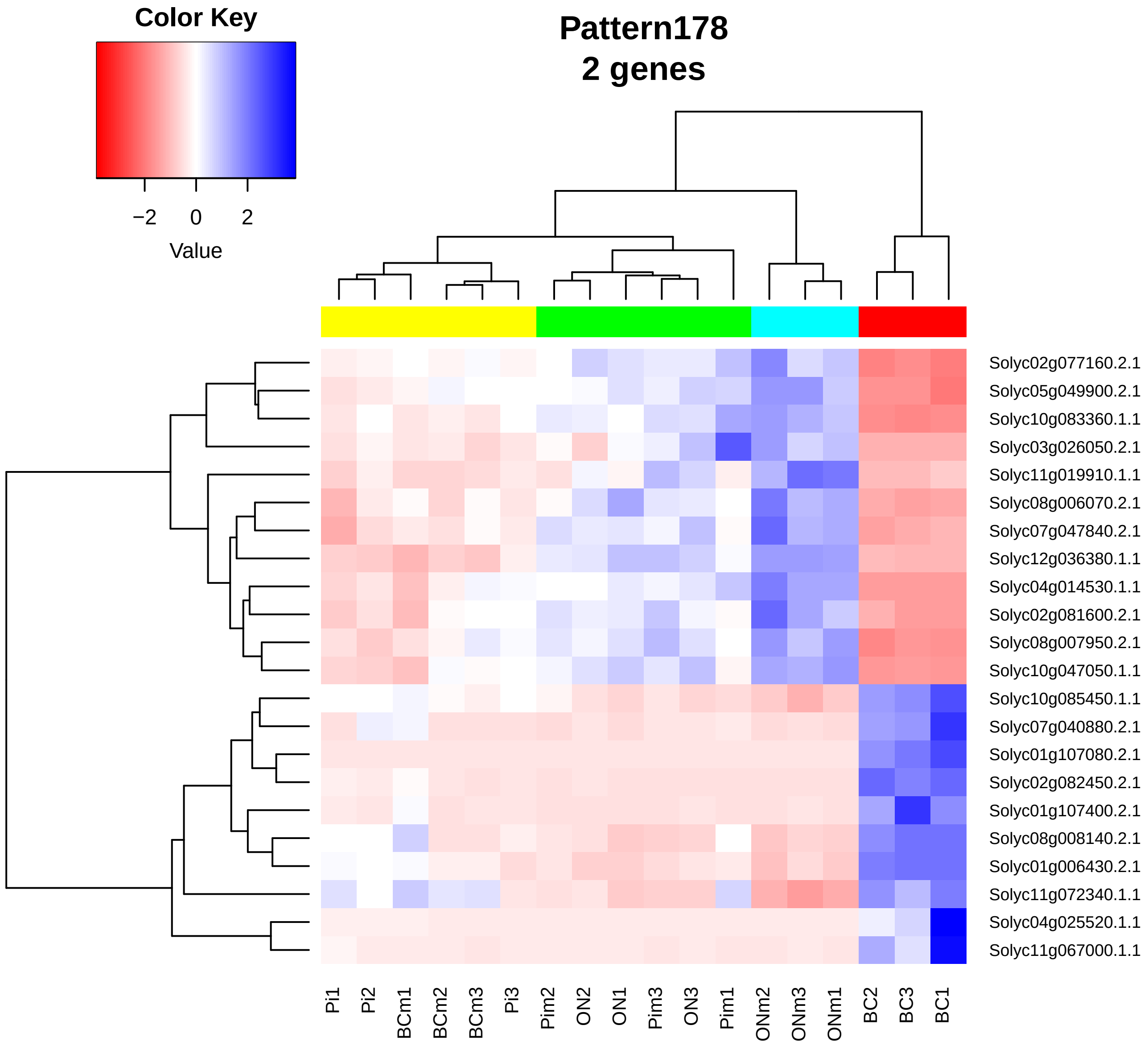

Color Key
−2
0
2
Value
Pattern178
2 genes
Solyc02g077160.2.1
Solyc05g049900.2.1
Solyc10g083360.1.1
Solyc03g026050.2.1
Solyc11g019910.1.1
Solyc08g006070.2.1
Solyc07g047840.2.1
Solyc12g036380.1.1
Solyc04g014530.1.1
Solyc02g081600.2.1
Solyc08g007950.2.1
Solyc10g047050.1.1
Solyc10g085450.1.1
Solyc07g040880.2.1
Solyc01g107080.2.1
Solyc02g082450.2.1
Solyc01g107400.2.1
Solyc08g008140.2.1
Solyc01g006430.2.1
Solyc11g072340.1.1
Solyc04g025520.1.1
Solyc11g067000.1.1
Pi1
Pi2
BCm1
BCm2
BCm3
Pi3
Pim2
ON2
ON1
Pim3
ON3
Pim1
ONm2
ONm3
ONm1
BC2
BC3
BC1

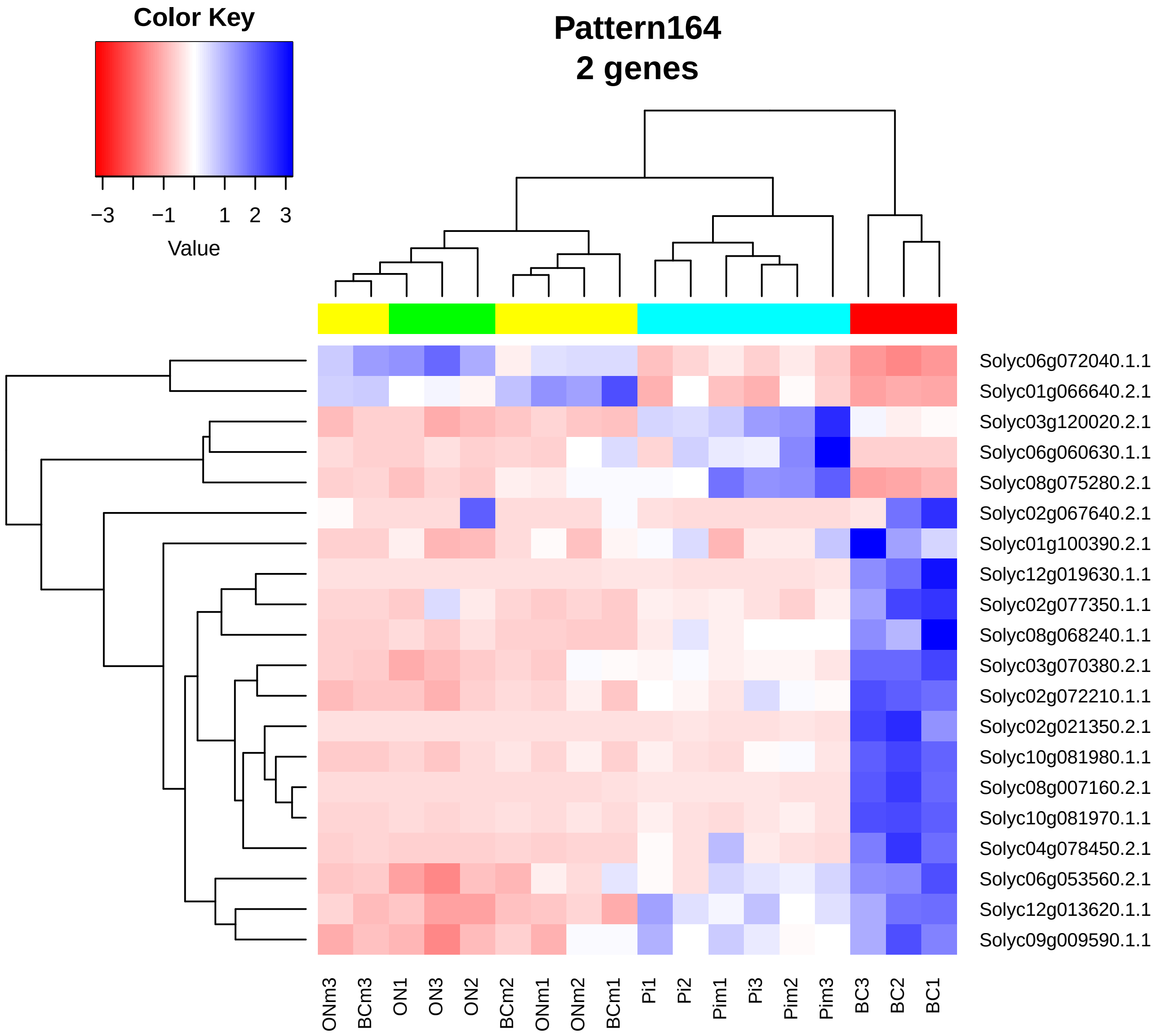

Color Key
−3
−1
1
2
3
Value
Pattern164
2 genes
Solyc06g072040.1.1
Solyc01g066640.2.1
Solyc03g120020.2.1
Solyc06g060630.1.1
Solyc08g075280.2.1
Solyc02g067640.2.1
Solyc01g100390.2.1
Solyc12g019630.1.1
Solyc02g077350.1.1
Solyc08g068240.1.1
Solyc03g070380.2.1
Solyc02g072210.1.1
Solyc02g021350.2.1
Solyc10g081980.1.1
Solyc08g007160.2.1
Solyc10g081970.1.1
Solyc04g078450.2.1
Solyc06g053560.2.1
Solyc12g013620.1.1
Solyc09g009590.1.1
ONm3
BCm3
ON1
ON3
ON2
BCm2
ONm1
ONm2
BCm1
Pi1
Pi2
Pim1
Pi3
Pim2
Pim3
BC3
BC2
BC1

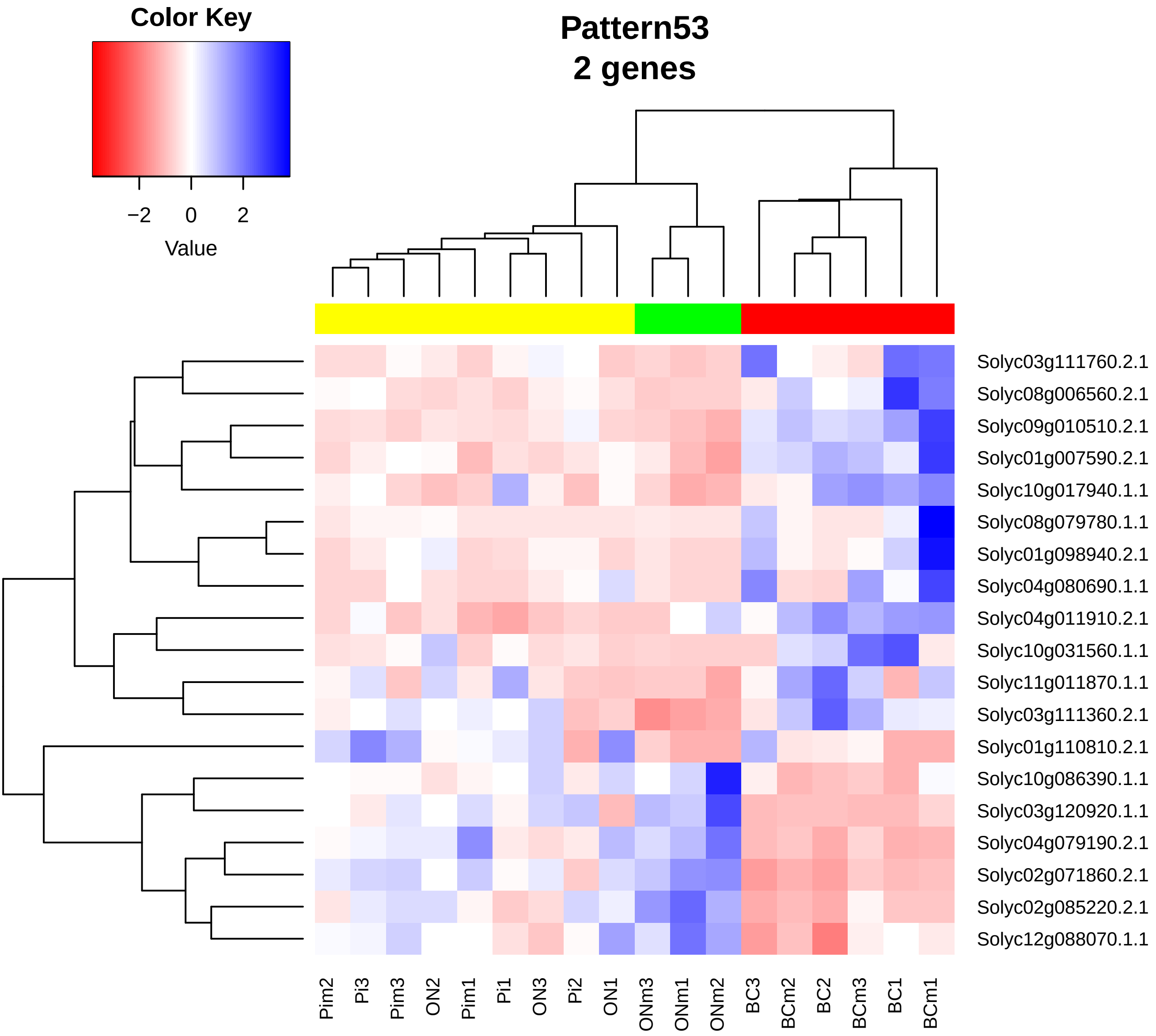
Color Key
−2
0
2
Value
Pattern53
2 genes
Solyc03g111760.2.1
Solyc08g006560.2.1
Solyc09g010510.2.1
Solyc01g007590.2.1
Solyc10g017940.1.1
Solyc08g079780.1.1
Solyc01g098940.2.1
Solyc04g080690.1.1
Solyc04g011910.2.1
Solyc10g031560.1.1
Solyc11g011870.1.1
Solyc03g111360.2.1
Solyc01g110810.2.1
Solyc10g086390.1.1
Solyc03g120920.1.1
Solyc04g079190.2.1
Solyc02g071860.2.1
Solyc02g085220.2.1
Solyc12g088070.1.1
Pim2
Pi3
Pim3
ON2
Pim1
Pi1
ON3
Pi2
ON1
ONm3
ONm1
ONm2
BC3
BCm2
BC2
BCm3
BC1
BCm1

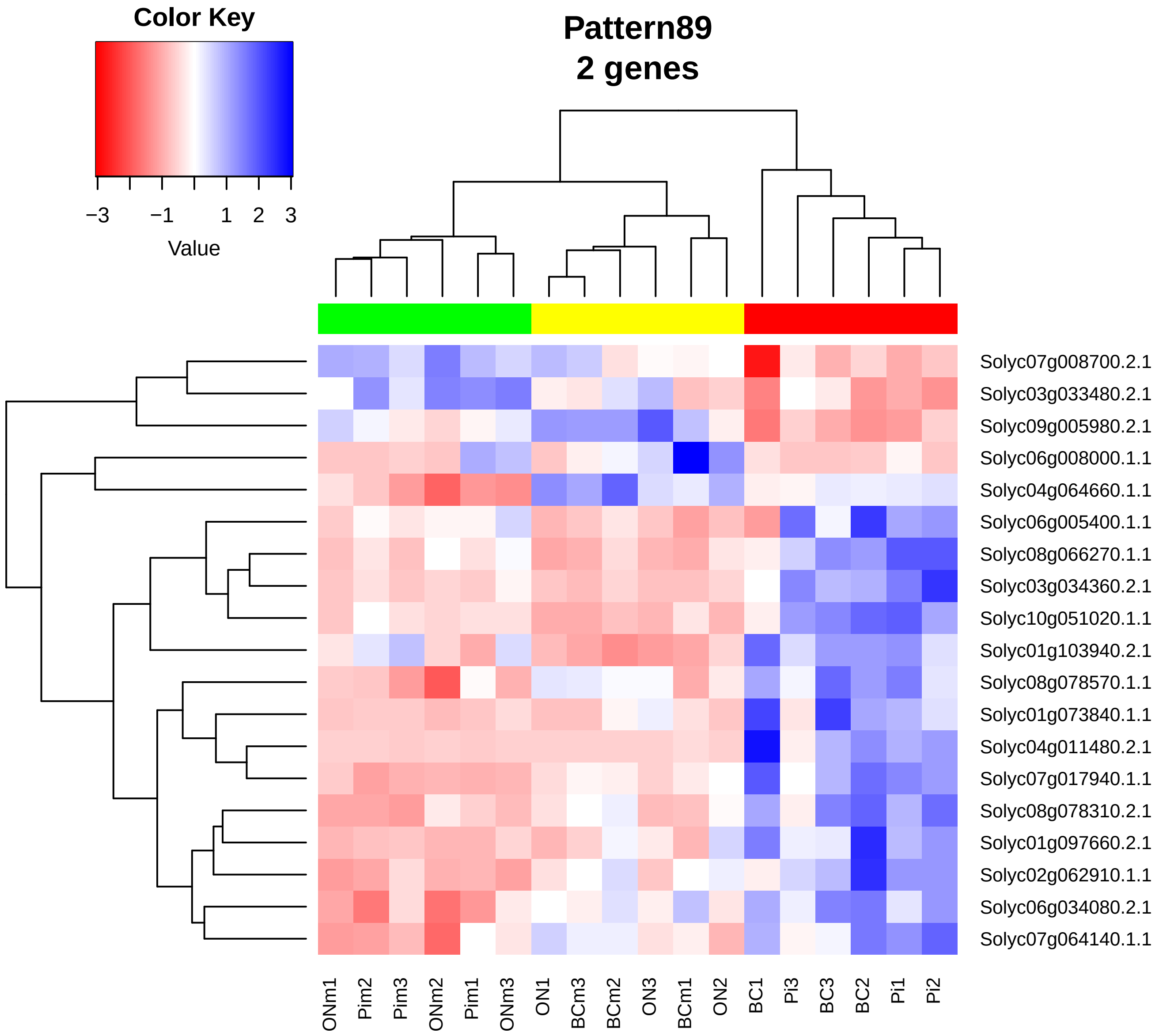
Color Key
−3
−1
1
2
3
Value
Pattern89
2 genes
Solyc07g008700.2.1
Solyc03g033480.2.1
Solyc09g005980.2.1
Solyc06g008000.1.1
Solyc04g064660.1.1
Solyc06g005400.1.1
Solyc08g066270.1.1
Solyc03g034360.2.1
Solyc10g051020.1.1
Solyc01g103940.2.1
Solyc08g078570.1.1
Solyc01g073840.1.1
Solyc04g011480.2.1
Solyc07g017940.1.1
Solyc08g078310.2.1
Solyc01g097660.2.1
Solyc02g062910.1.1
Solyc06g034080.2.1
Solyc07g064140.1.1
ONm1
Pim2
Pim3
ONm2
Pim1
ONm3
ON1
BCm3
BCm2
ON3
BCm1
ON2
BC1
Pi3
BC3
BC2
Pi1
Pi2

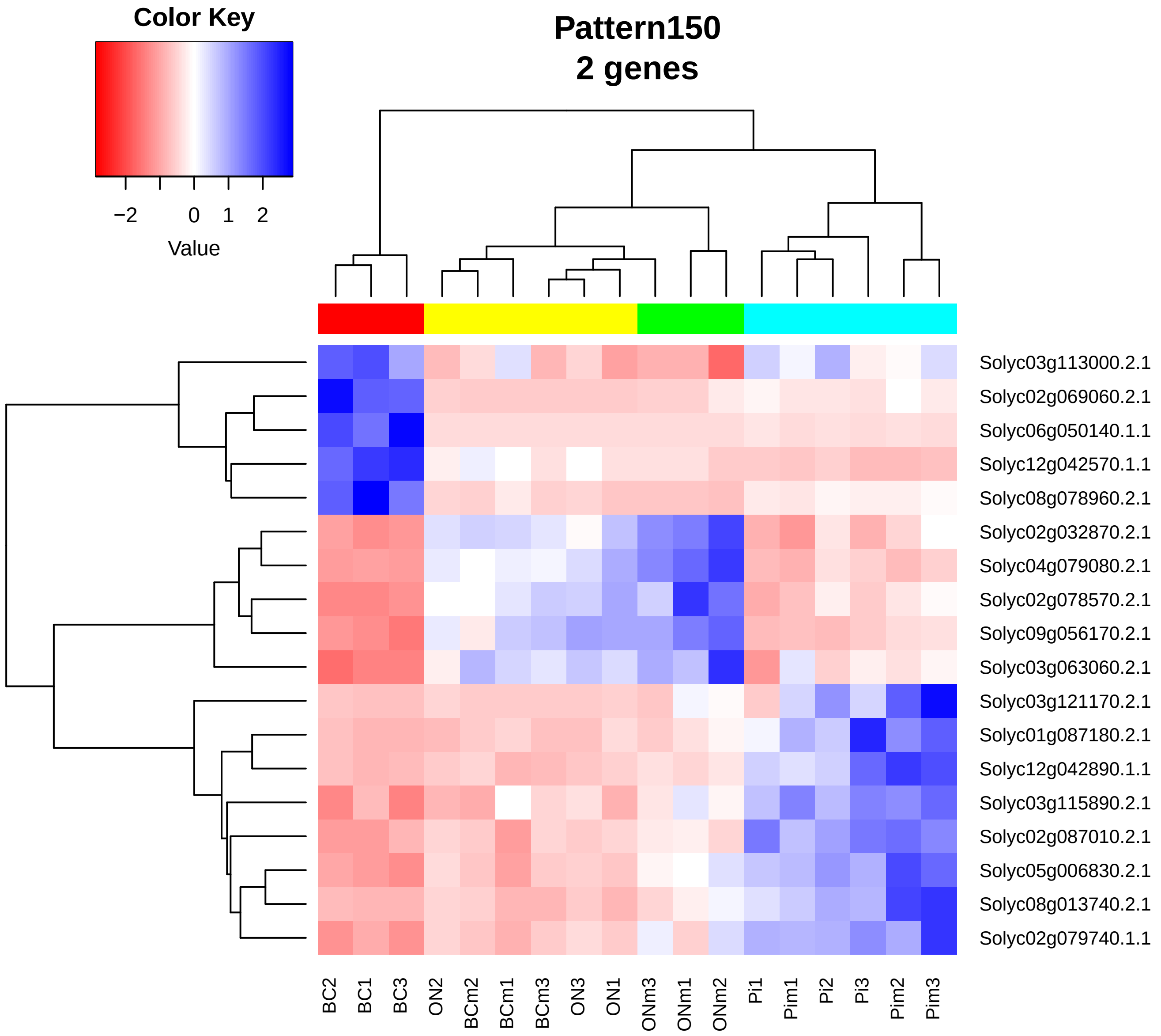

Color Key
−2
0
1
2
Value
Pattern150
2 genes
Solyc03g113000.2.1
Solyc02g069060.2.1
Solyc06g050140.1.1
Solyc12g042570.1.1
Solyc08g078960.2.1
Solyc02g032870.2.1
Solyc04g079080.2.1
Solyc02g078570.2.1
Solyc09g056170.2.1
Solyc03g063060.2.1
Solyc03g121170.2.1
Solyc01g087180.2.1
Solyc12g042890.1.1
Solyc03g115890.2.1
Solyc02g087010.2.1
Solyc05g006830.2.1
Solyc08g013740.2.1
Solyc02g079740.1.1
BC2
BC1
BC3
ON2
BCm2
BCm1
BCm3
ON3
ON1
ONm3
ONm1
ONm2
Pi1
Pim1
Pi2
Pi3
Pim2
Pim3

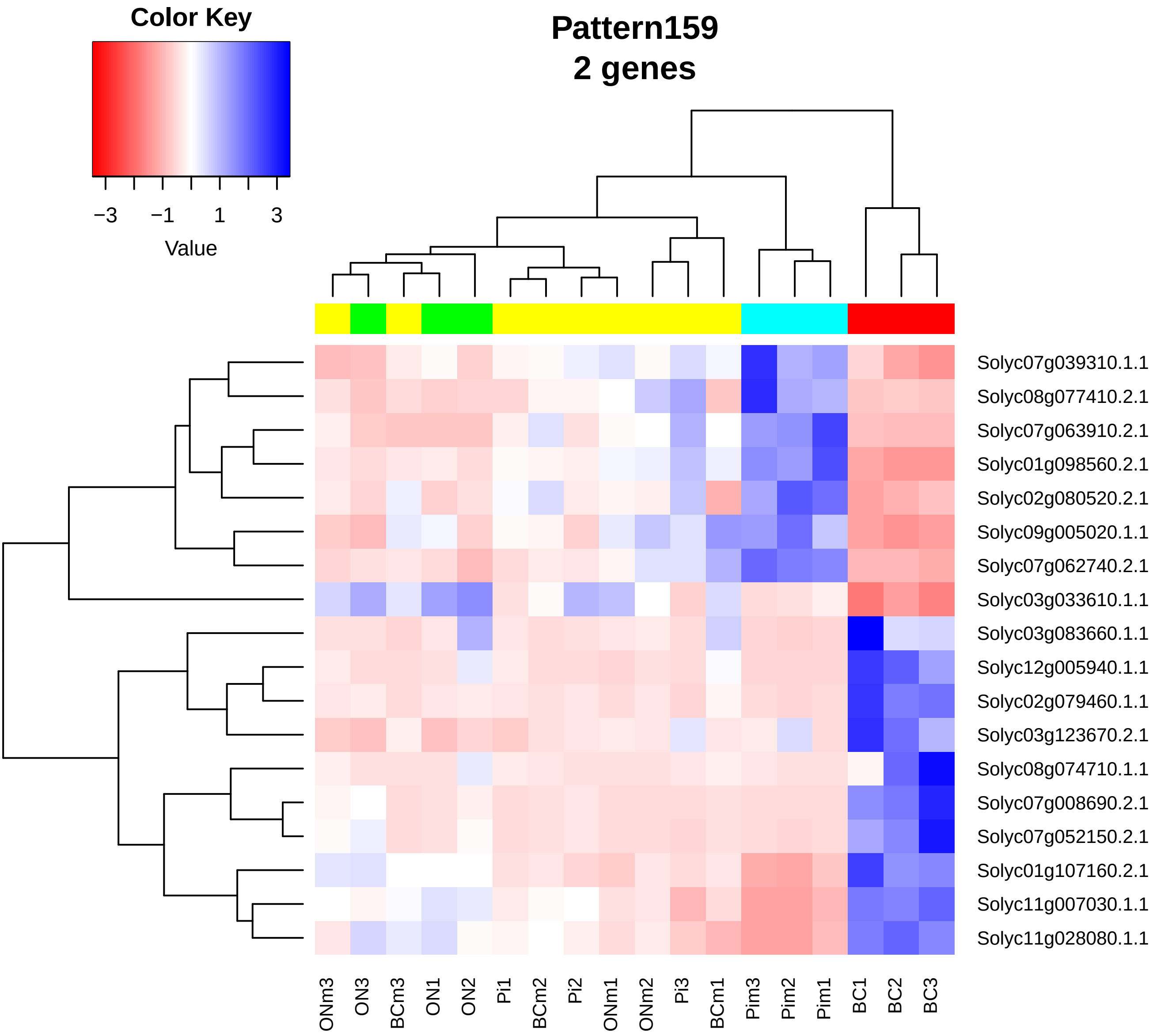
Color Key
−3
−1
1
3
Value
Pattern159
2 genes
Solyc07g039310.1.1
Solyc08g077410.2.1
Solyc07g063910.2.1
Solyc01g098560.2.1
Solyc02g080520.2.1
Solyc09g005020.1.1
Solyc07g062740.2.1
Solyc03g033610.1.1
Solyc03g083660.1.1
Solyc12g005940.1.1
Solyc02g079460.1.1
Solyc03g123670.2.1
Solyc08g074710.1.1
Solyc07g008690.2.1
Solyc07g052150.2.1
Solyc01g107160.2.1
Solyc11g007030.1.1
Solyc11g028080.1.1
ONm3
ON3
BCm3
ON1
ON2
Pi1
BCm2
Pi2
ONm1
ONm2
Pi3
BCm1
Pim3
Pim2
Pim1
BC1
BC2
BC3

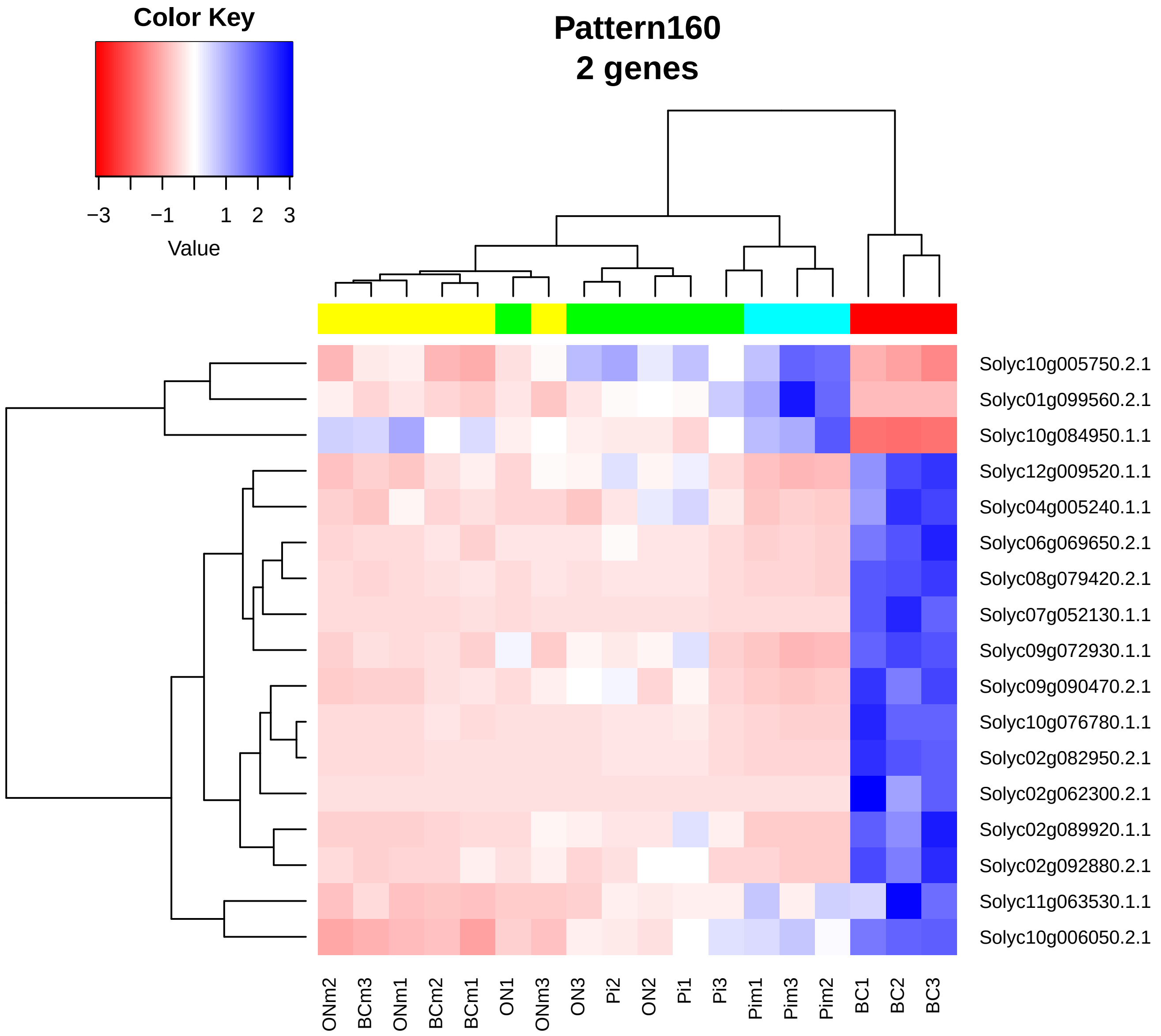
Color Key
−3
−1
1
2
3
Value
Pattern160
2 genes
Solyc10g005750.2.1
Solyc01g099560.2.1
Solyc10g084950.1.1
Solyc12g009520.1.1
Solyc04g005240.1.1
Solyc06g069650.2.1
Solyc08g079420.2.1
Solyc07g052130.1.1
Solyc09g072930.1.1
Solyc09g090470.2.1
Solyc10g076780.1.1
Solyc02g082950.2.1
Solyc02g062300.2.1
Solyc02g089920.1.1
Solyc02g092880.2.1
Solyc11g063530.1.1
Solyc10g006050.2.1
ONm2
BCm3
ONm1
BCm2
BCm1
ON1
ONm3
ON3
Pi2
ON2
Pi1
Pi3
Pim1
Pim3
Pim2
BC1
BC2
BC3

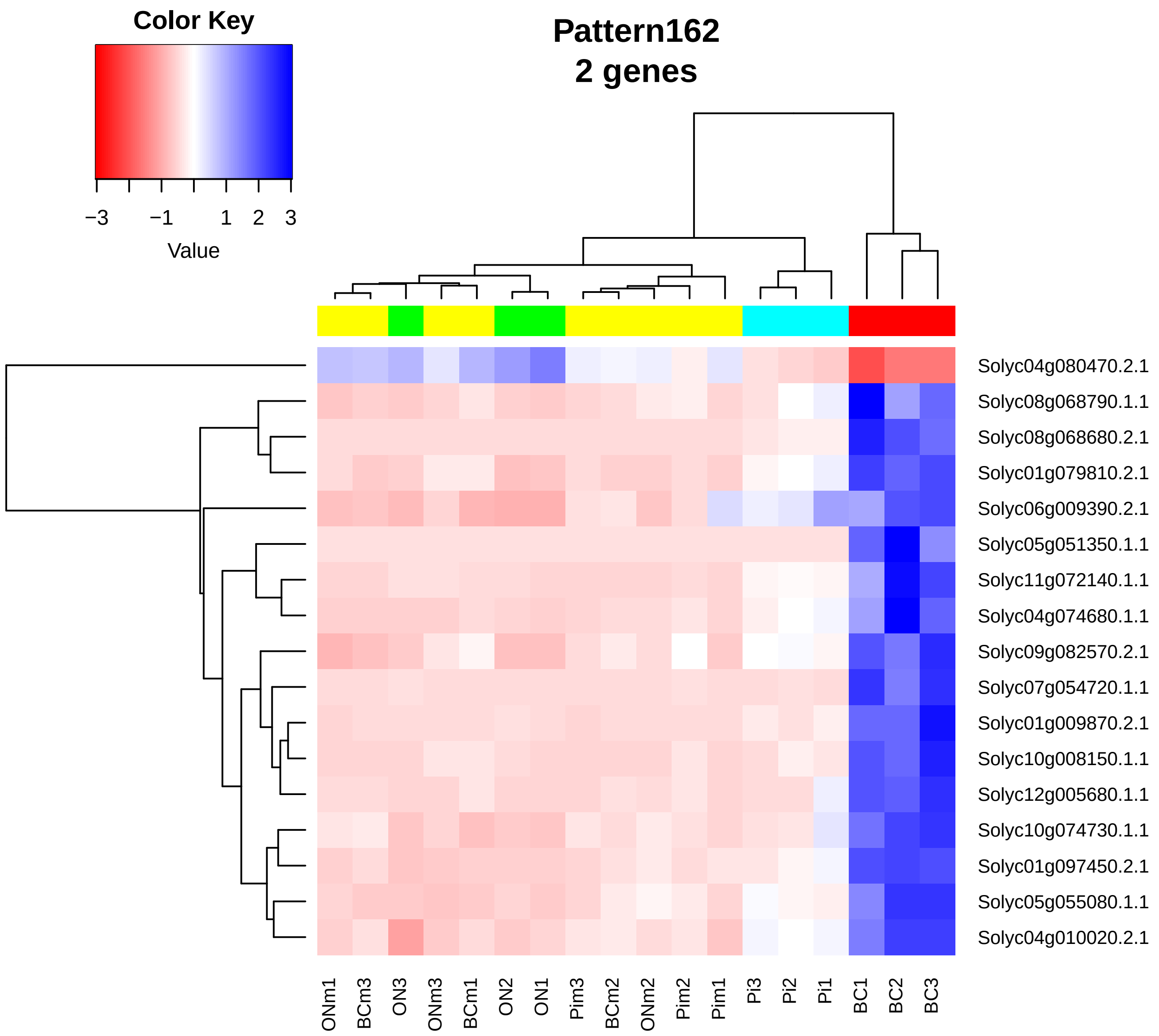
Color Key
−3
−1
1
2
3
Value
Pattern162
2 genes
Solyc04g080470.2.1
Solyc08g068790.1.1
Solyc08g068680.2.1
Solyc01g079810.2.1
Solyc06g009390.2.1
Solyc05g051350.1.1
Solyc11g072140.1.1
Solyc04g074680.1.1
Solyc09g082570.2.1
Solyc07g054720.1.1
Solyc01g009870.2.1
Solyc10g008150.1.1
Solyc12g005680.1.1
Solyc10g074730.1.1
Solyc01g097450.2.1
Solyc05g055080.1.1
Solyc04g010020.2.1
ONm1
BCm3
ON3
ONm3
BCm1
ON2
ON1
Pim3
BCm2
ONm2
Pim2
Pim1
Pi3
Pi2
Pi1
BC1
BC2
BC3

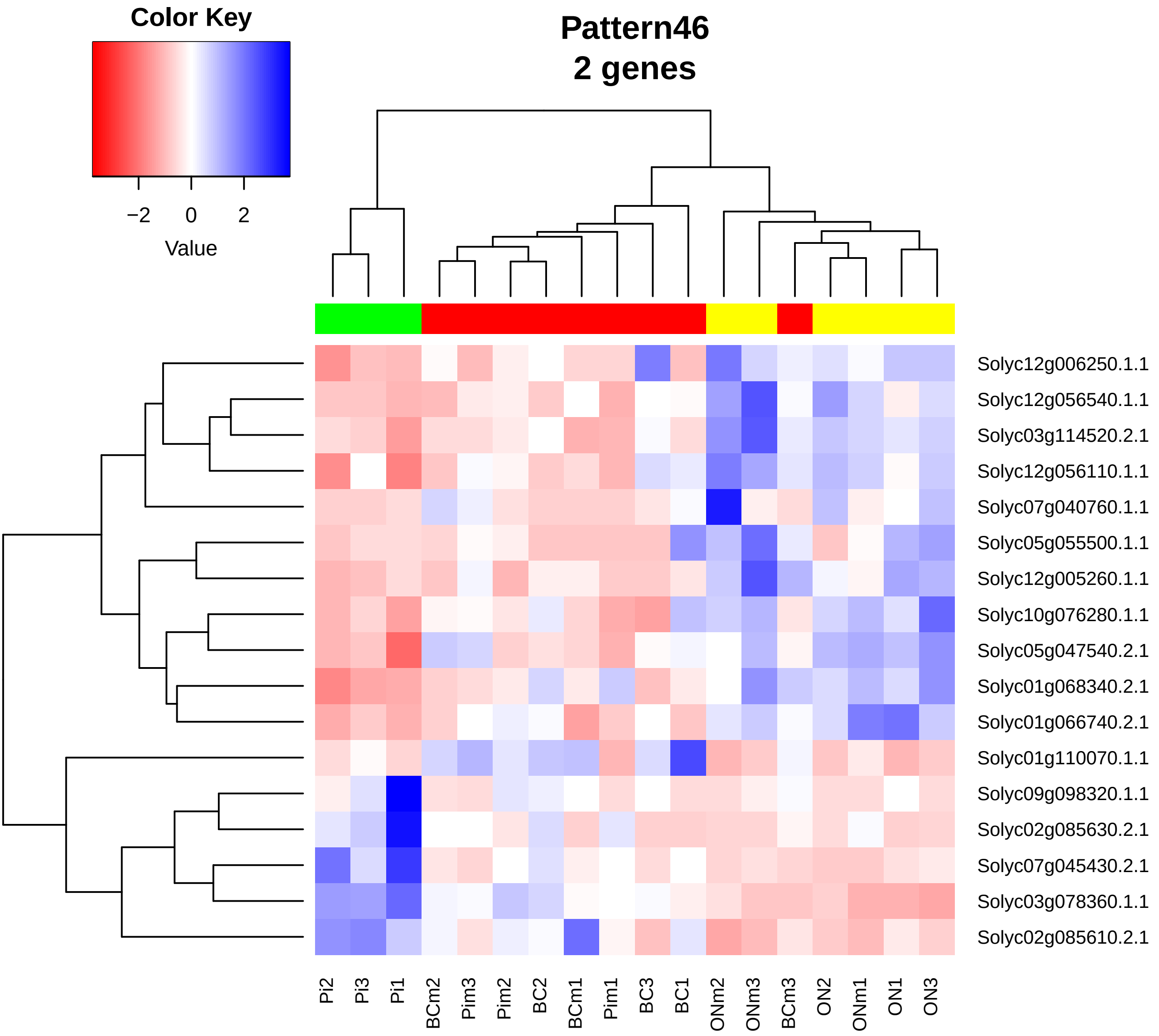

Color Key
−2
0
2
Value
Pattern46
2 genes
Solyc12g006250.1.1
Solyc12g056540.1.1
Solyc03g114520.2.1
Solyc12g056110.1.1
Solyc07g040760.1.1
Solyc05g055500.1.1
Solyc12g005260.1.1
Solyc10g076280.1.1
Solyc05g047540.2.1
Solyc01g068340.2.1
Solyc01g066740.2.1
Solyc01g110070.1.1
Solyc09g098320.1.1
Solyc02g085630.2.1
Solyc07g045430.2.1
Solyc03g078360.1.1
Solyc02g085610.2.1
Pi2
Pi3
Pi1
BCm2
Pim3
Pim2
BC2
BCm1
Pim1
BC3
BC1
ONm2
ONm3
BCm3
ON2
ONm1
ON1
ON3

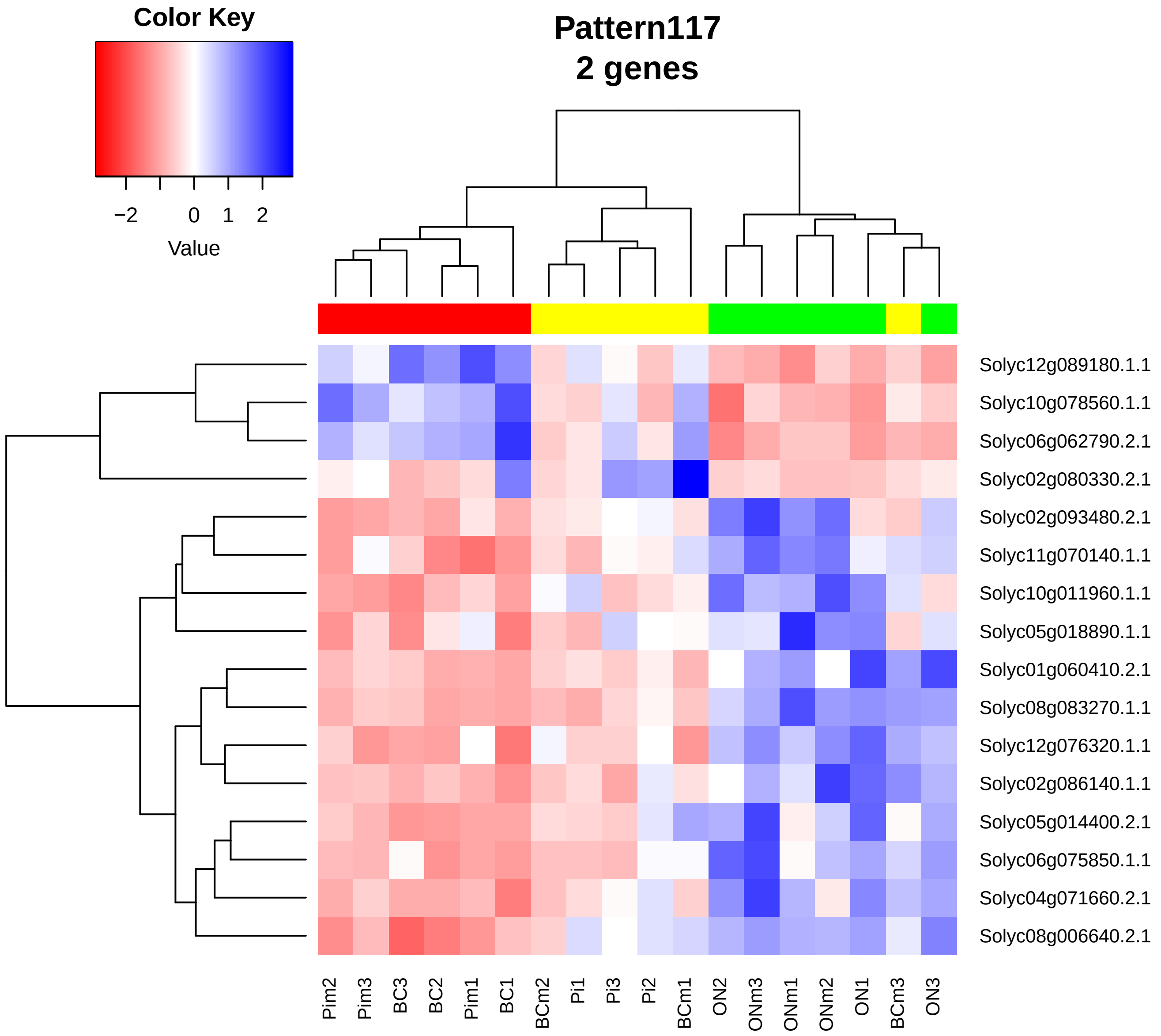

Color Key
−2
0
1
2
Value
Pattern117
2 genes
Solyc12g089180.1.1
Solyc10g078560.1.1
Solyc06g062790.2.1
Solyc02g080330.2.1
Solyc02g093480.2.1
Solyc11g070140.1.1
Solyc10g011960.1.1
Solyc05g018890.1.1
Solyc01g060410.2.1
Solyc08g083270.1.1
Solyc12g076320.1.1
Solyc02g086140.1.1
Solyc05g014400.2.1
Solyc06g075850.1.1
Solyc04g071660.2.1
Solyc08g006640.2.1
Pim2
Pim3
BC3
BC2
Pim1
BC1
BCm2
Pi1
Pi3
Pi2
BCm1
ON2
ONm3
ONm1
ONm2
ON1
BCm3
ON3

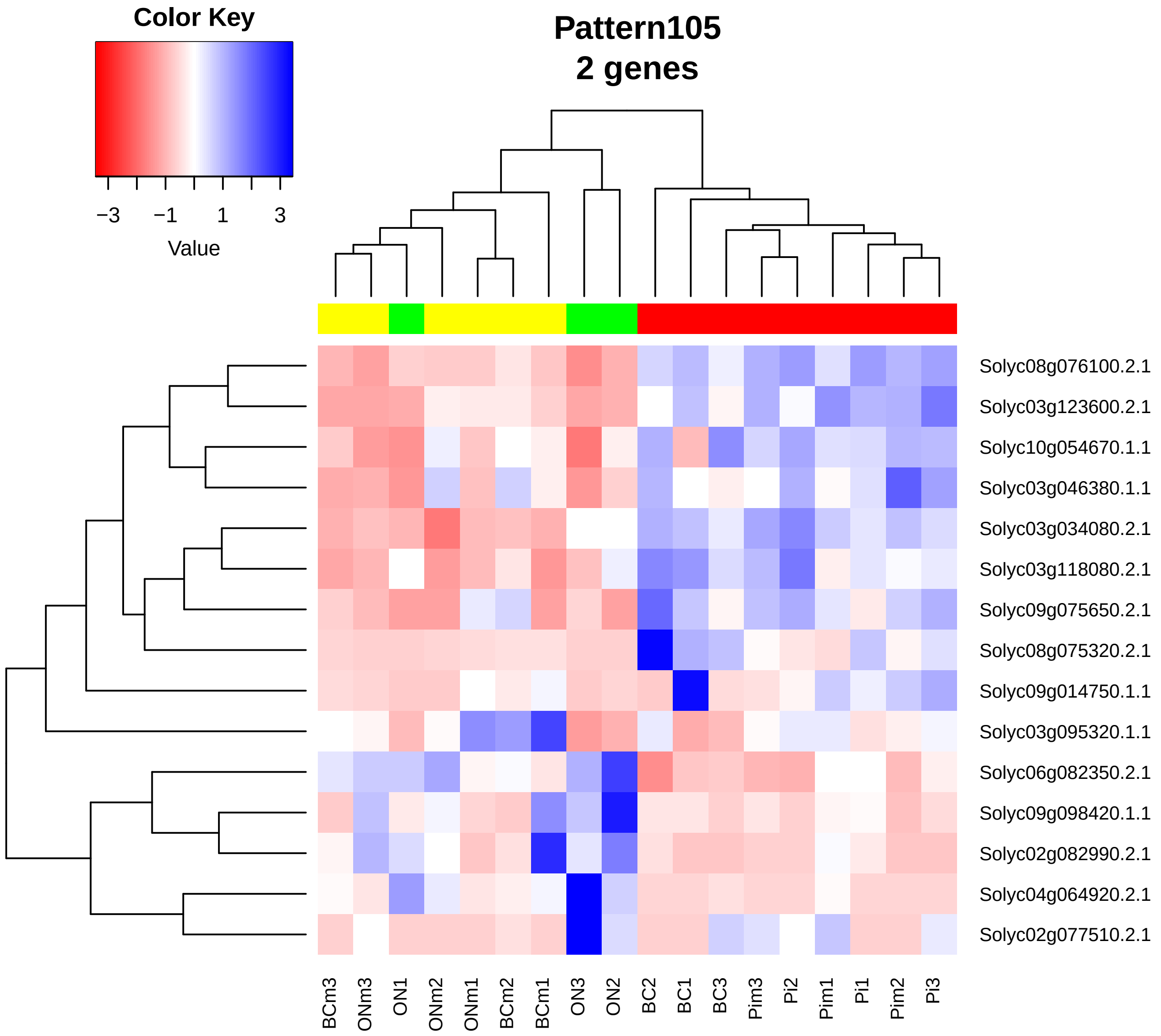

Color Key
−3
−1
1
3
Value
Pattern105
2 genes
Solyc08g076100.2.1
Solyc03g123600.2.1
Solyc10g054670.1.1
Solyc03g046380.1.1
Solyc03g034080.2.1
Solyc03g118080.2.1
Solyc09g075650.2.1
Solyc08g075320.2.1
Solyc09g014750.1.1
Solyc03g095320.1.1
Solyc06g082350.2.1
Solyc09g098420.1.1
Solyc02g082990.2.1
Solyc04g064920.2.1
Solyc02g077510.2.1
BCm3
ONm3
ON1
ONm2
ONm1
BCm2
BCm1
ON3
ON2
BC2
BC1
BC3
Pim3
Pi2
Pim1
Pi1
Pim2
Pi3

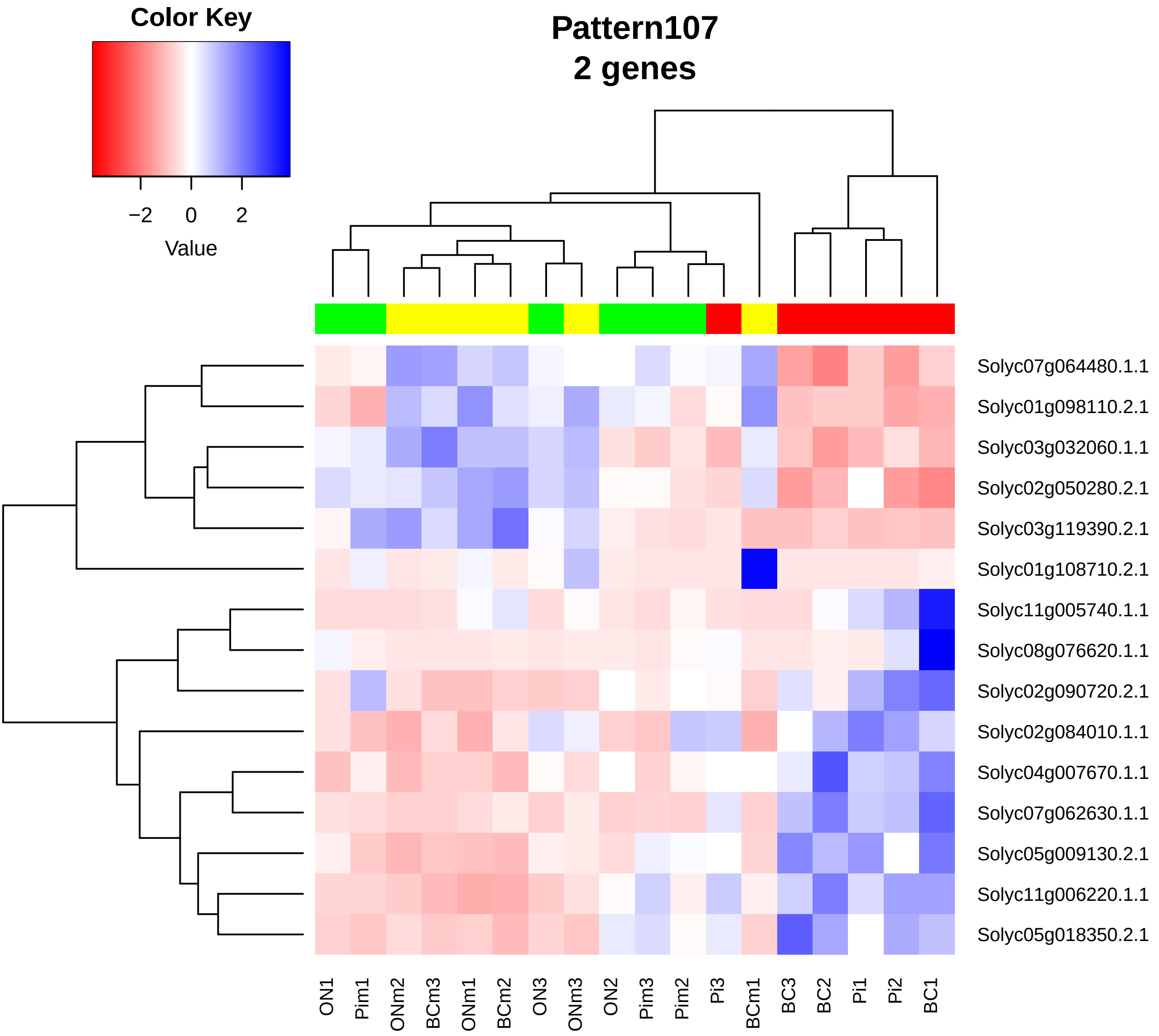
Color Key
−2
0
2
Value
Pattern107
2 genes
Solyc07g064480.1.1
Solyc01g098110.2.1
Solyc03g032060.1.1
Solyc02g050280.2.1
Solyc03g119390.2.1
Solyc01g108710.2.1
Solyc11g005740.1.1
Solyc08g076620.1.1
Solyc02g090720.2.1
Solyc02g084010.1.1
Solyc04g007670.1.1
Solyc07g062630.1.1
Solyc05g009130.2.1
Solyc11g006220.1.1
Solyc05g018350.2.1
ON1
Pim1
ONm2
BCm3
ONm1
BCm2
ON3
ONm3
ON2
Pim3
Pim2
Pi3
BCm1
BC3
BC2
Pi1
Pi2
BC1

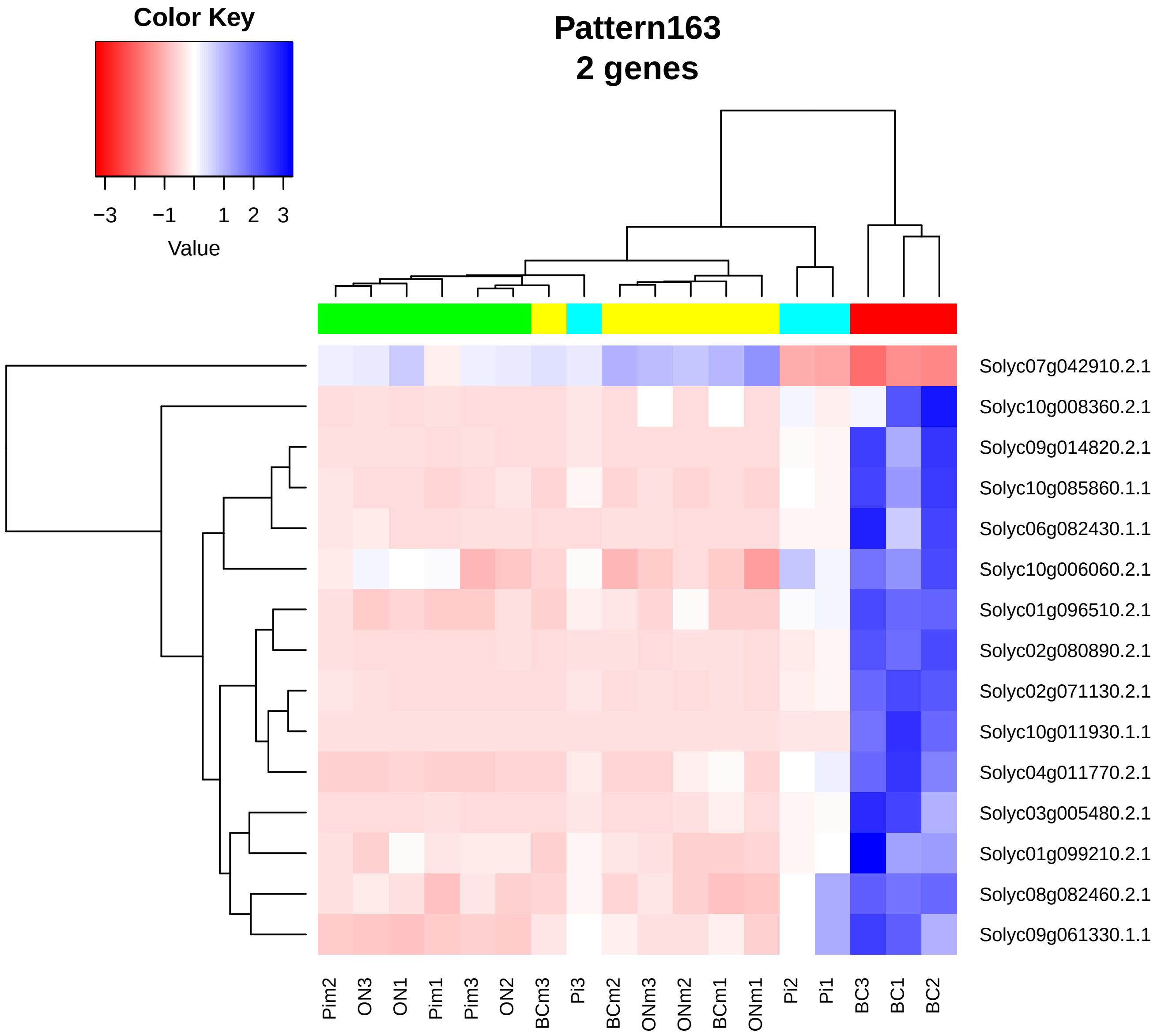
Color Key
−3
−1
1
2
3
Value
Pattern163
2 genes
Solyc07g042910.2.1
Solyc10g008360.2.1
Solyc09g014820.2.1
Solyc10g085860.1.1
Solyc06g082430.1.1
Solyc10g006060.2.1
Solyc01g096510.2.1
Solyc02g080890.2.1
Solyc02g071130.2.1
Solyc10g011930.1.1
Solyc04g011770.2.1
Solyc03g005480.2.1
Solyc01g099210.2.1
Solyc08g082460.2.1
Solyc09g061330.1.1
Pim2
ON3
ON1
Pim1
Pim3
ON2
BCm3
Pi3
BCm2
ONm3
ONm2
BCm1
ONm1
Pi2
Pi1
BC3
BC1
BC2

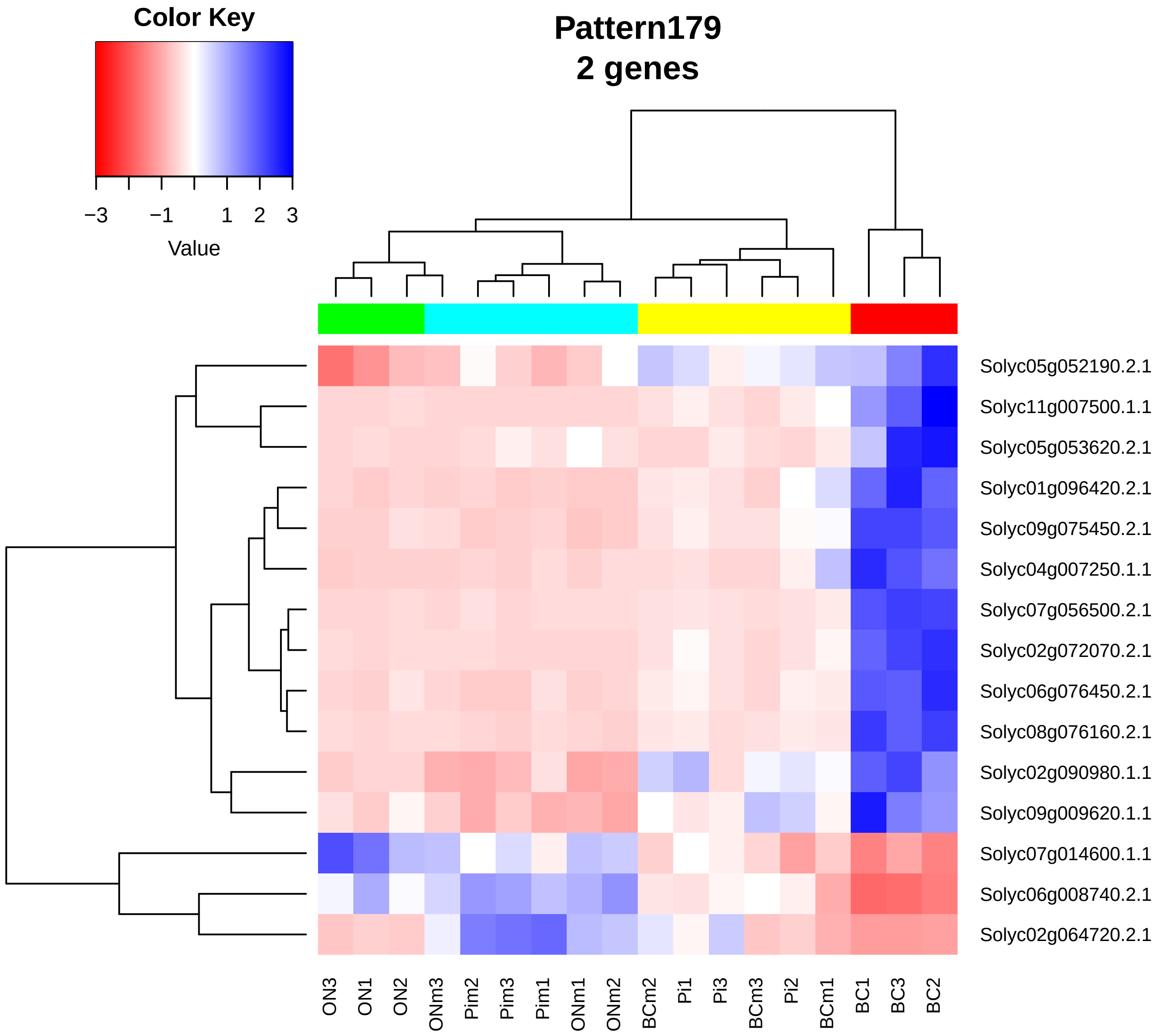
Color Key
−3
−1
1
2
3
Value
Pattern179
2 genes
Solyc05g052190.2.1
Solyc11g007500.1.1
Solyc05g053620.2.1
Solyc01g096420.2.1
Solyc09g075450.2.1
Solyc04g007250.1.1
Solyc07g056500.2.1
Solyc02g072070.2.1
Solyc06g076450.2.1
Solyc08g076160.2.1
Solyc02g090980.1.1
Solyc09g009620.1.1
Solyc07g014600.1.1
Solyc06g008740.2.1
Solyc02g064720.2.1
ON3
ON1
ON2
ONm3
Pim2
Pim3
Pim1
ONm1
ONm2
BCm2
Pi1
Pi3
BCm3
Pi2
BCm1
BC1
BC3
BC2

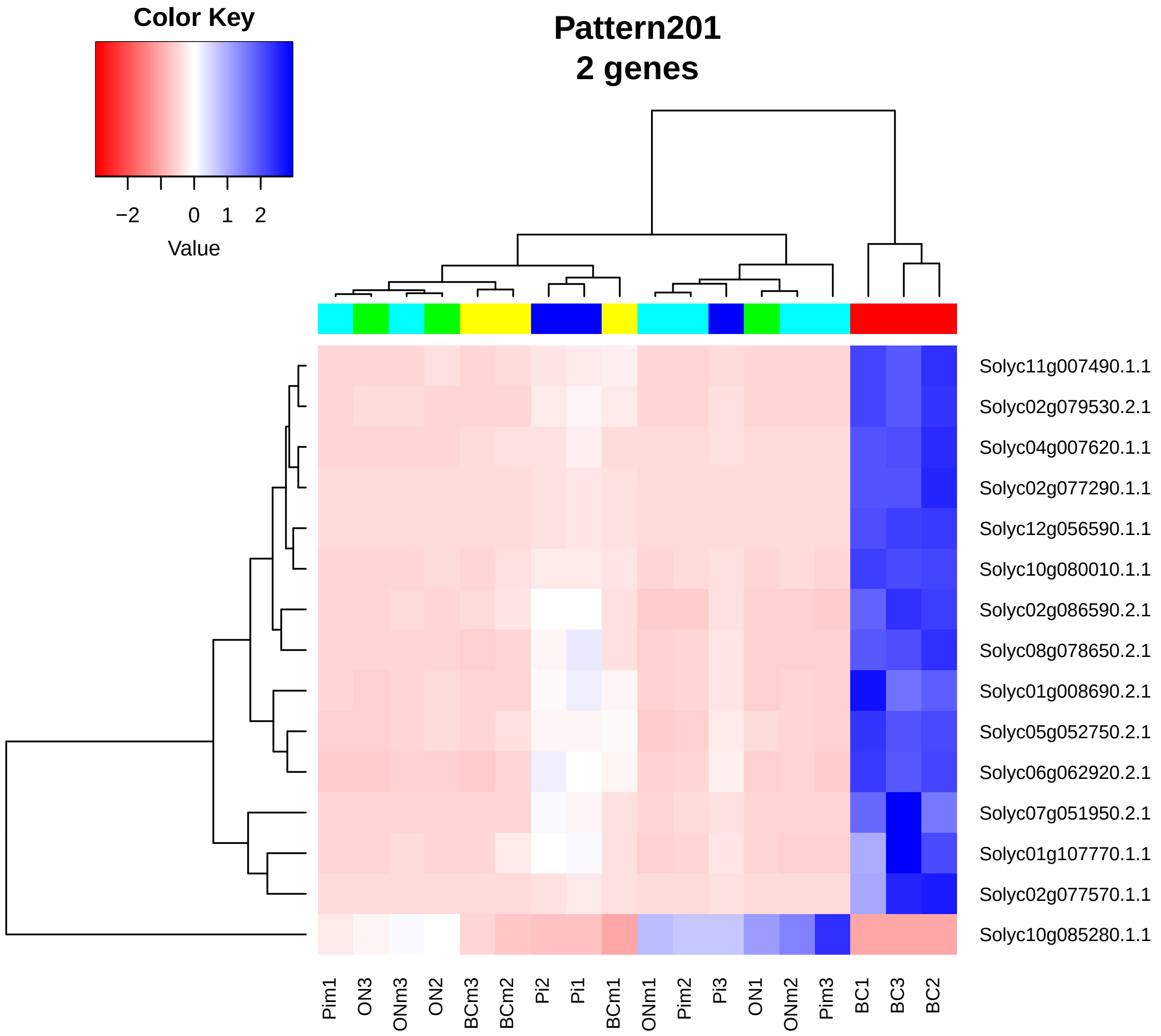

Color Key
−2
0
1
2
Value
Pattern201
2 genes
Solyc11g007490.1.1
Solyc02g079530.2.1
Solyc04g007620.1.1
Solyc02g077290.1.1
Solyc12g056590.1.1
Solyc10g080010.1.1
Solyc02g086590.2.1
Solyc08g078650.2.1
Solyc01g008690.2.1
Solyc05g052750.2.1
Solyc06g062920.2.1
Solyc07g051950.2.1
Solyc01g107770.1.1
Solyc02g077570.1.1
Solyc10g085280.1.1
Pim1
ON3
ONm3
ON2
BCm3
BCm2
Pi2
Pi1
BCm1
ONm1
Pim2
Pi3
ON1
ONm2
Pim3
BC1
BC3
BC2

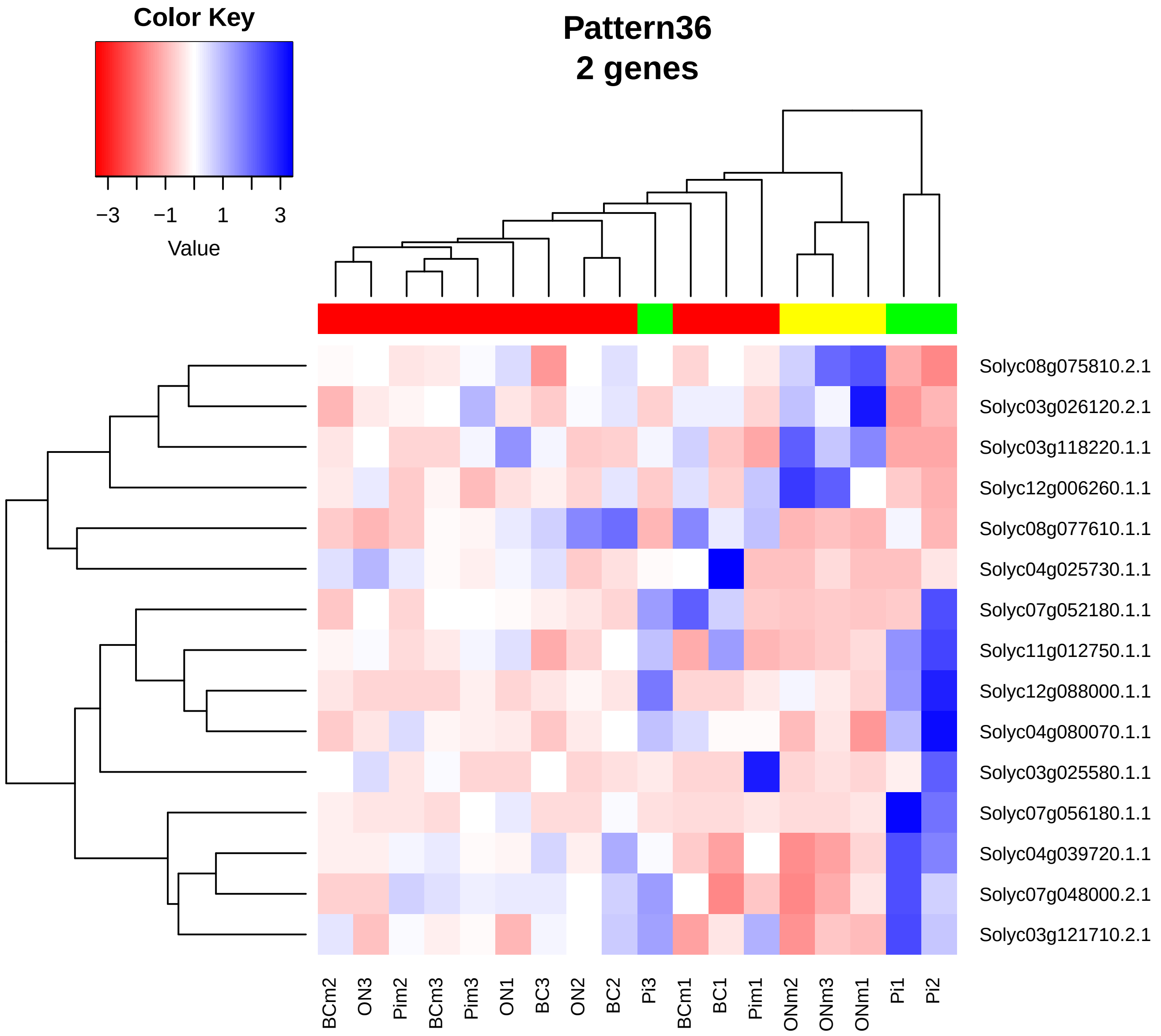
Color Key
−3
−1
1
3
Value
Pattern36
2 genes
Solyc08g075810.2.1
Solyc03g026120.2.1
Solyc03g118220.1.1
Solyc12g006260.1.1
Solyc08g077610.1.1
Solyc04g025730.1.1
Solyc07g052180.1.1
Solyc11g012750.1.1
Solyc12g088000.1.1
Solyc04g080070.1.1
Solyc03g025580.1.1
Solyc07g056180.1.1
Solyc04g039720.1.1
Solyc07g048000.2.1
Solyc03g121710.2.1
BCm2
ON3
Pim2
BCm3
Pim3
ON1
BC3
ON2
BC2
Pi3
BCm1
BC1
Pim1
ONm2
ONm3
ONm1
Pi1
Pi2

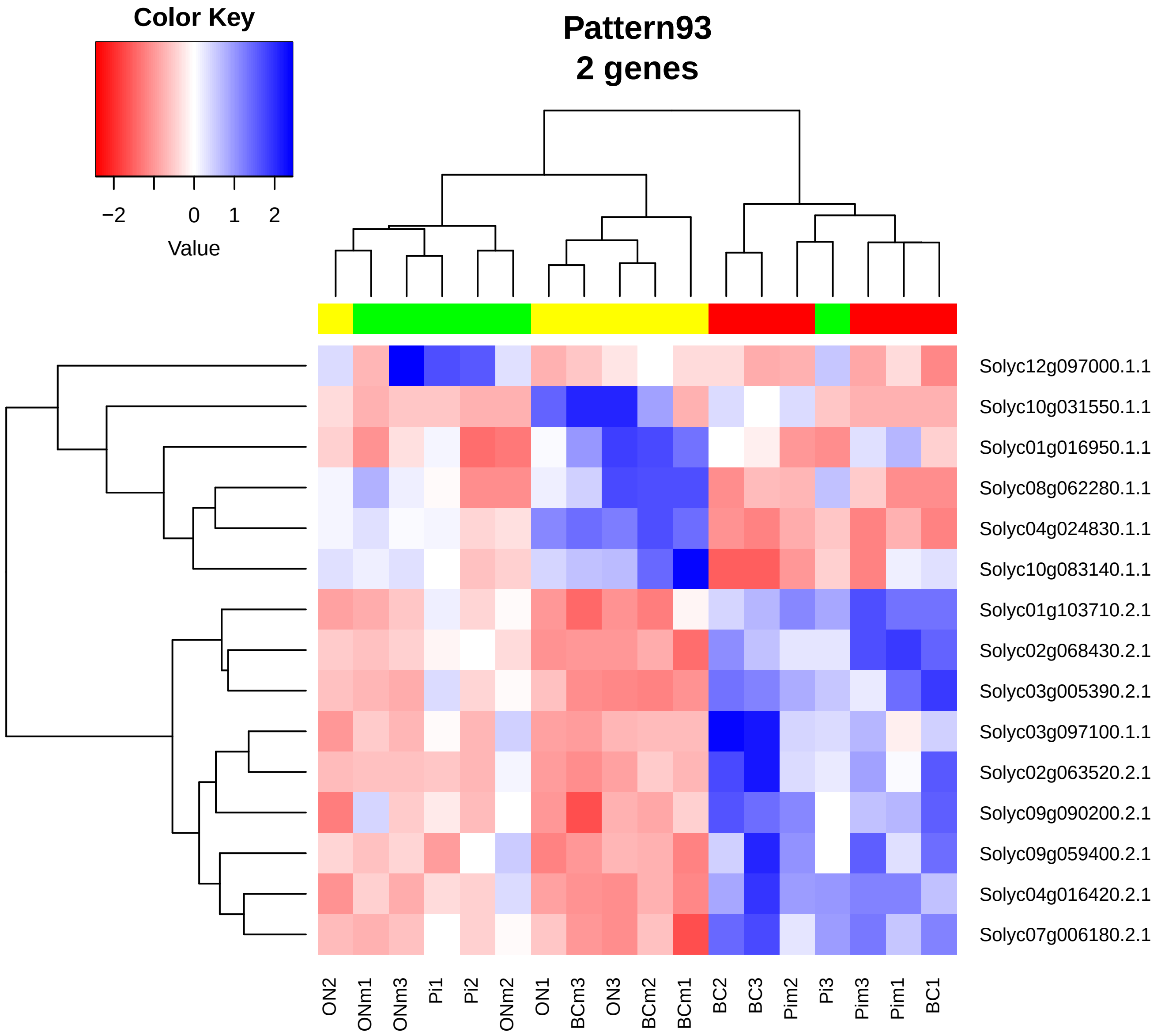

Color Key
−2
0
1
2
Value
Pattern93
2 genes
Solyc12g097000.1.1
Solyc10g031550.1.1
Solyc01g016950.1.1
Solyc08g062280.1.1
Solyc04g024830.1.1
Solyc10g083140.1.1
Solyc01g103710.2.1
Solyc02g068430.2.1
Solyc03g005390.2.1
Solyc03g097100.1.1
Solyc02g063520.2.1
Solyc09g090200.2.1
Solyc09g059400.2.1
Solyc04g016420.2.1
Solyc07g006180.2.1
ON2
ONm1
ONm3
Pi1
Pi2
ONm2
ON1
BCm3
ON3
BCm2
BCm1
BC2
BC3
Pim2
Pi3
Pim3
Pim1
BC1

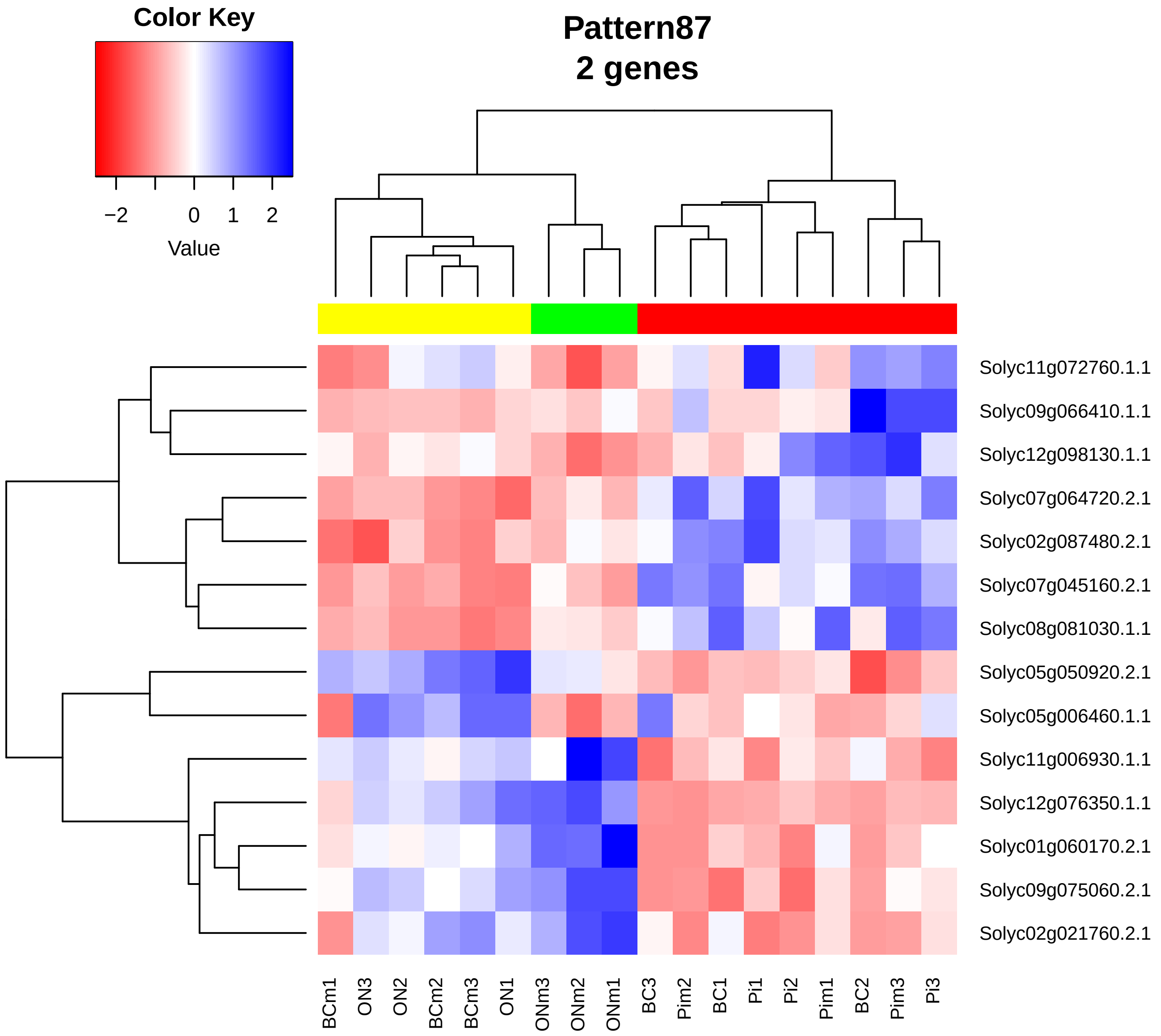
Color Key
−2
0
1
2
Value
Pattern87
2 genes
Solyc11g072760.1.1
Solyc09g066410.1.1
Solyc12g098130.1.1
Solyc07g064720.2.1
Solyc02g087480.2.1
Solyc07g045160.2.1
Solyc08g081030.1.1
Solyc05g050920.2.1
Solyc05g006460.1.1
Solyc11g006930.1.1
Solyc12g076350.1.1
Solyc01g060170.2.1
Solyc09g075060.2.1
Solyc02g021760.2.1
BCm1
ON3
ON2
BCm2
BCm3
ON1
ONm3
ONm2
ONm1
BC3
Pim2
BC1
Pi1
Pi2
Pim1
BC2
Pim3
Pi3

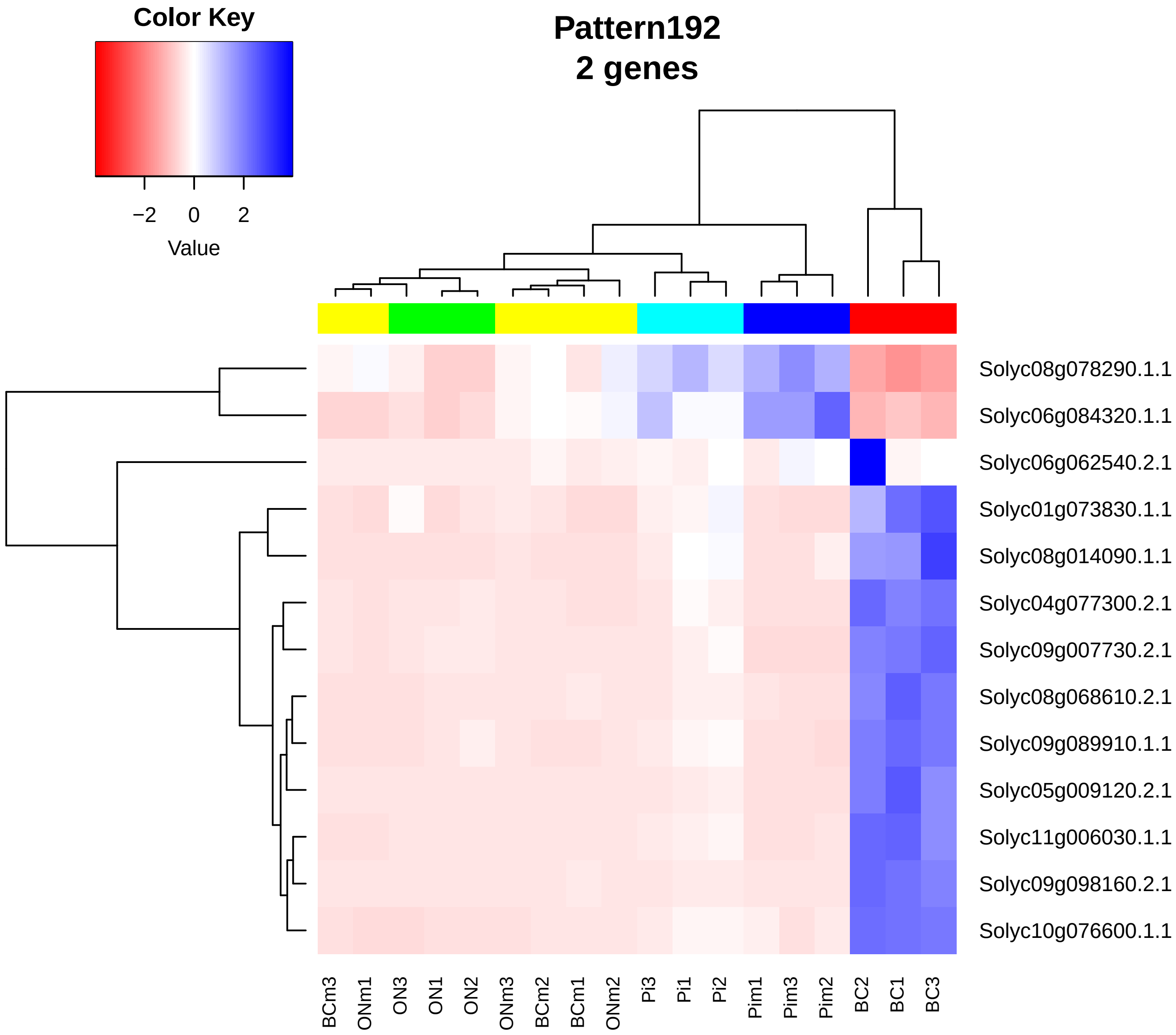
Color Key
−2
0
2
Value
Pattern192
2 genes
Solyc08g078290.1.1
Solyc06g084320.1.1
Solyc06g062540.2.1
Solyc01g073830.1.1
Solyc08g014090.1.1
Solyc04g077300.2.1
Solyc09g007730.2.1
Solyc08g068610.2.1
Solyc09g089910.1.1
Solyc05g009120.2.1
Solyc11g006030.1.1
Solyc09g098160.2.1
Solyc10g076600.1.1
BCm3
ONm1
ON3
ON1
ON2
ONm3
BCm2
BCm1
ONm2
Pi3
Pi1
Pi2
Pim1
Pim3
Pim2
BC2
BC1
BC3

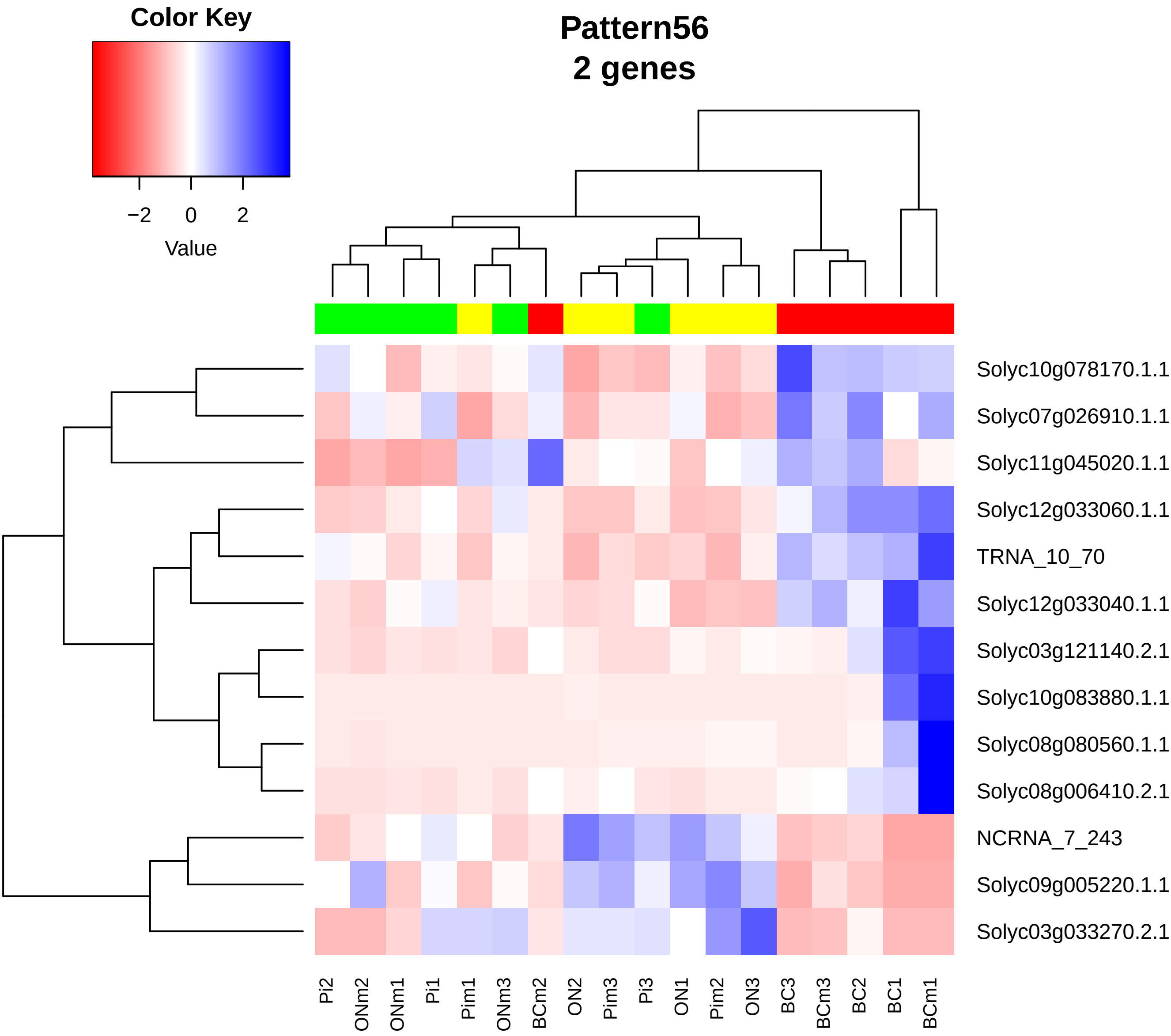

Color Key
−2
0
2
Value
Pattern56
2 genes
Solyc10g078170.1.1
Solyc07g026910.1.1
Solyc11g045020.1.1
Solyc12g033060.1.1
TRNA_10_70
Solyc12g033040.1.1
Solyc03g121140.2.1
Solyc10g083880.1.1
Solyc08g080560.1.1
Solyc08g006410.2.1
NCRNA_7_243
Solyc09g005220.1.1
Solyc03g033270.2.1
Pi2
ONm2
ONm1
Pi1
Pim1
ONm3
BCm2
ON2
Pim3
Pi3
ON1
Pim2
ON3
BC3
BCm3
BC2
BC1
BCm1

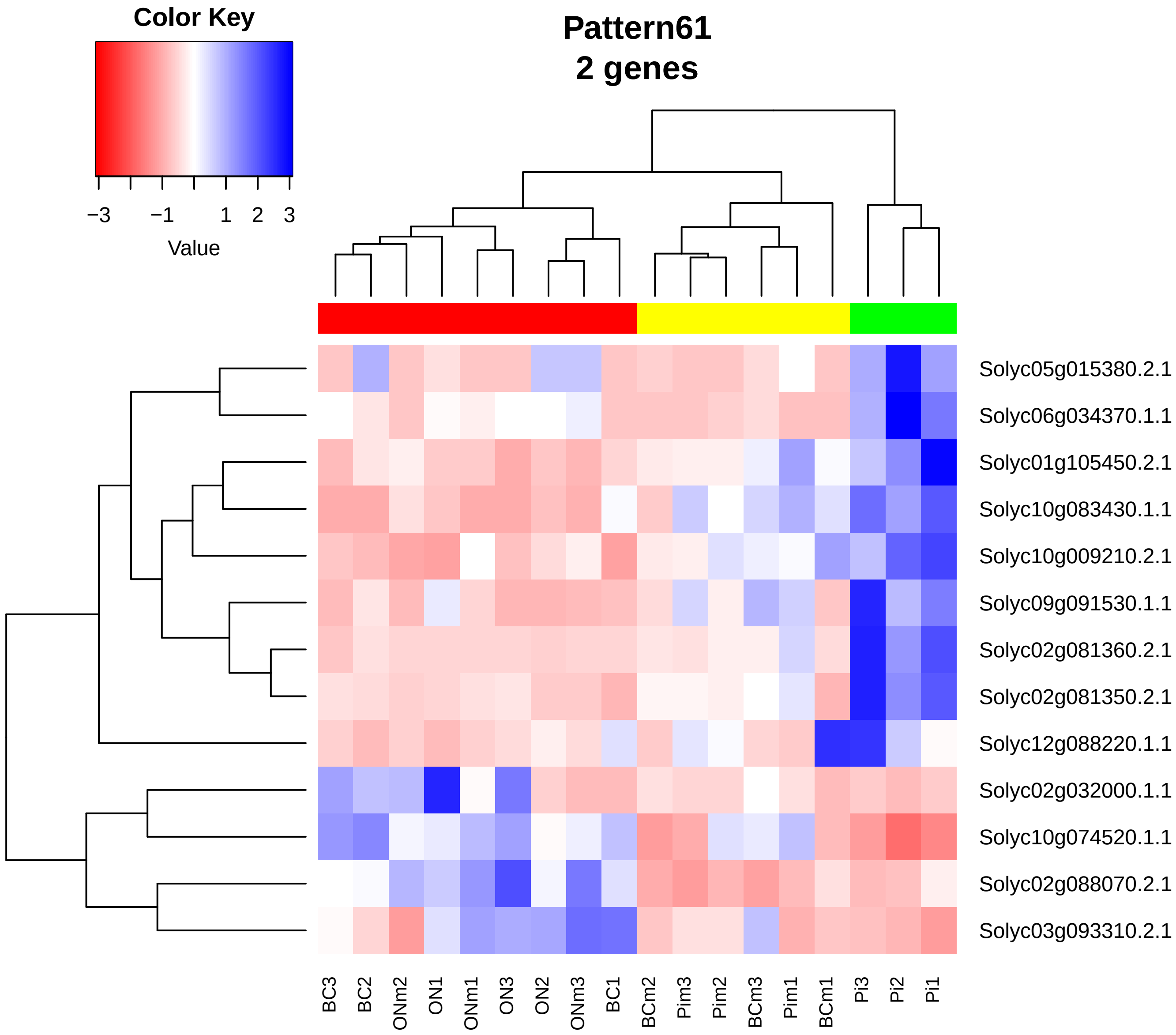

Color Key
−3
−1
1
2
3
Value
Pattern61
2 genes
Solyc05g015380.2.1
Solyc06g034370.1.1
Solyc01g105450.2.1
Solyc10g083430.1.1
Solyc10g009210.2.1
Solyc09g091530.1.1
Solyc02g081360.2.1
Solyc02g081350.2.1
Solyc12g088220.1.1
Solyc02g032000.1.1
Solyc10g074520.1.1
Solyc02g088070.2.1
Solyc03g093310.2.1
BC3
BC2
ONm2
ON1
ONm1
ON3
ON2
ONm3
BC1
BCm2
Pim3
Pim2
BCm3
Pim1
BCm1
Pi3
Pi2
Pi1

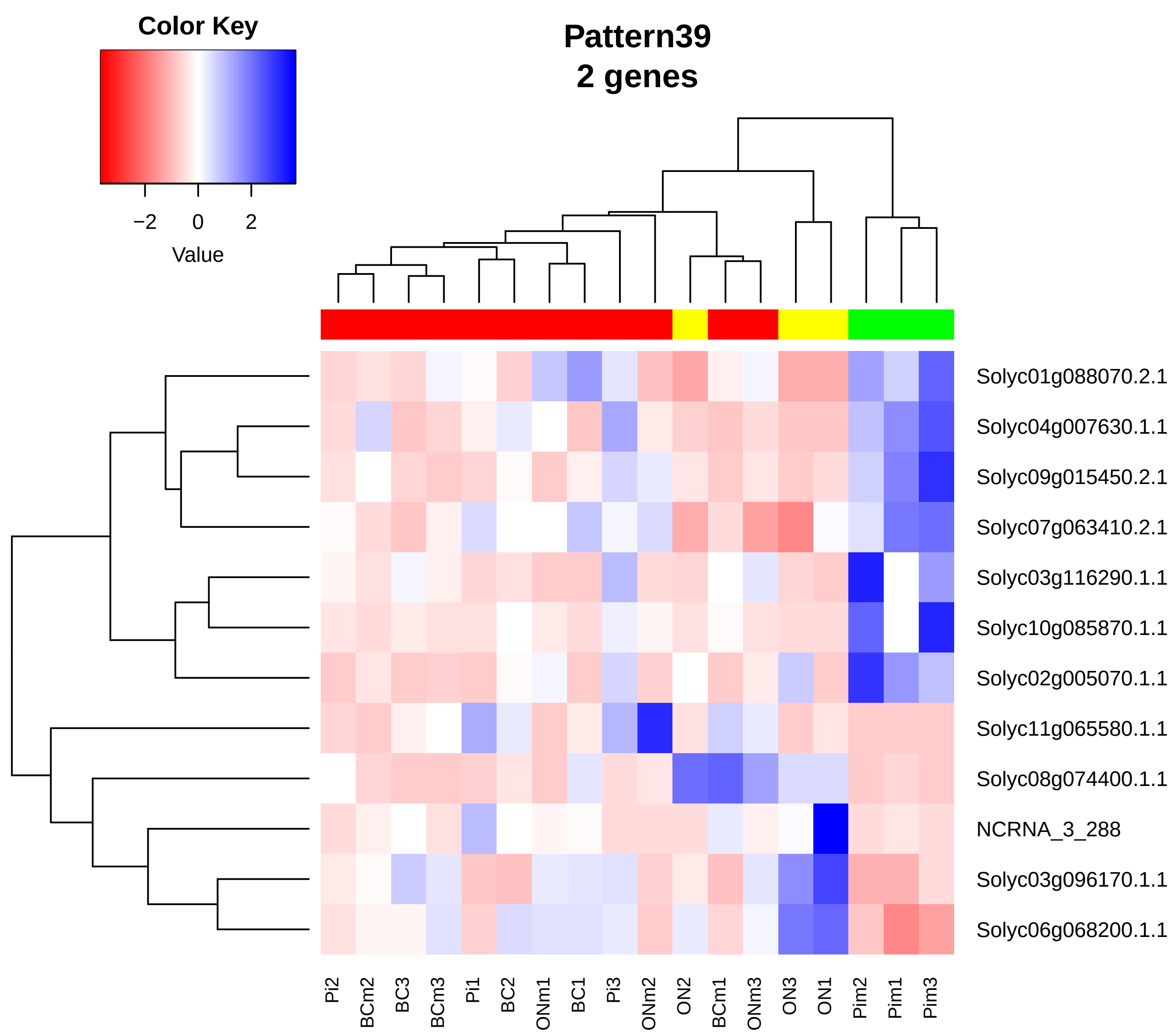

Color Key
−2
0
2
Value
Pattern39
2 genes
Solyc01g088070.2.1
Solyc04g007630.1.1
Solyc09g015450.2.1
Solyc07g063410.2.1
Solyc03g116290.1.1
Solyc10g085870.1.1
Solyc02g005070.1.1
Solyc11g065580.1.1
Solyc08g074400.1.1
NCRNA_3_288
Solyc03g096170.1.1
Solyc06g068200.1.1
Pi2
BCm2
BC3
BCm3
Pi1
BC2
ONm1
BC1
Pi3
ONm2
ON2
BCm1
ONm3
ON3
ON1
Pim2
Pim1
Pim3

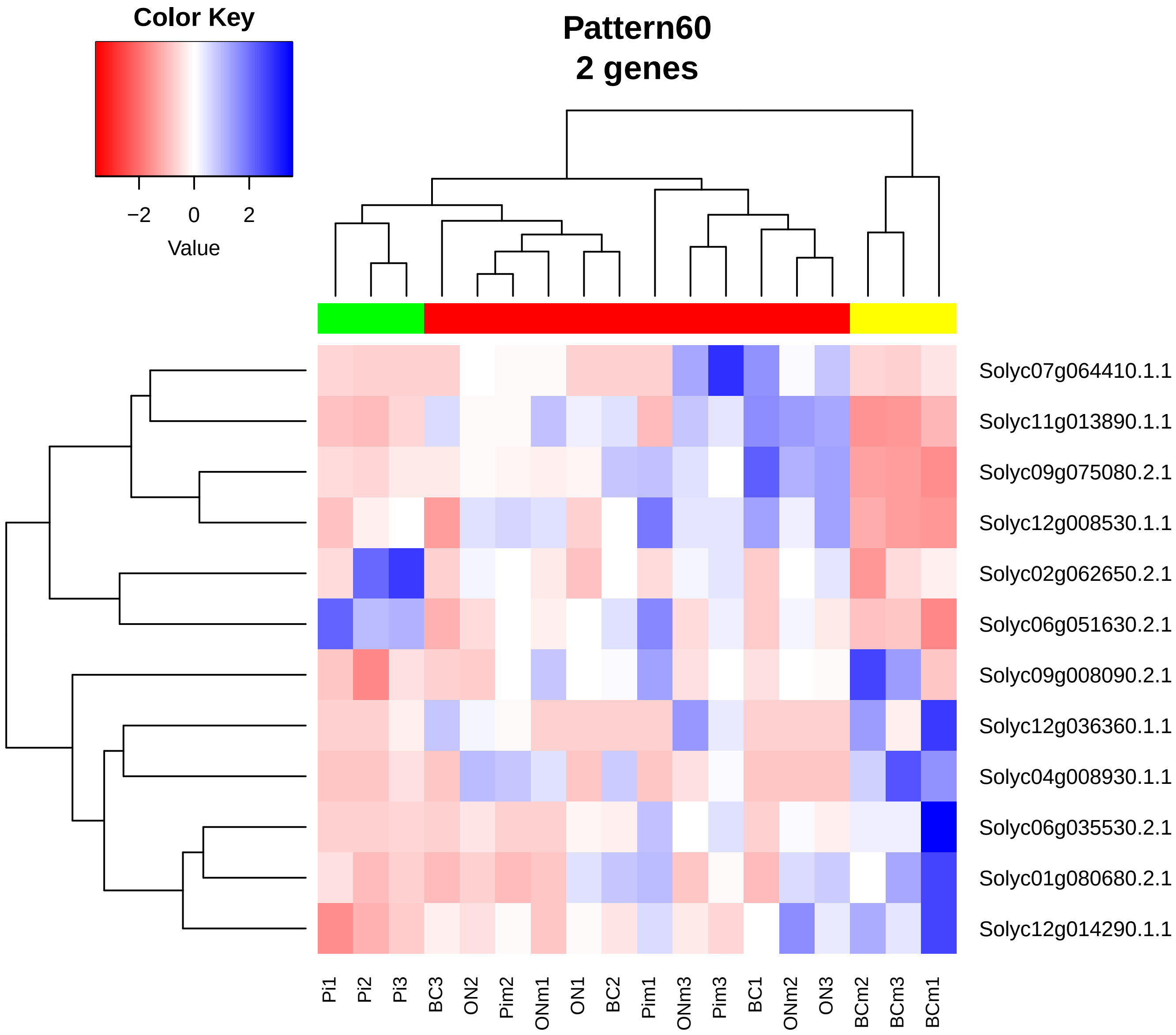
Color Key
−2
0
2
Value
Pattern60
2 genes
Solyc07g064410.1.1
Solyc11g013890.1.1
Solyc09g075080.2.1
Solyc12g008530.1.1
Solyc02g062650.2.1
Solyc06g051630.2.1
Solyc09g008090.2.1
Solyc12g036360.1.1
Solyc04g008930.1.1
Solyc06g035530.2.1
Solyc01g080680.2.1
Solyc12g014290.1.1
Pi1
Pi2
Pi3
BC3
ON2
Pim2
ONm1
ON1
BC2
Pim1
ONm3
Pim3
BC1
ONm2
ON3
BCm2
BCm3
BCm1

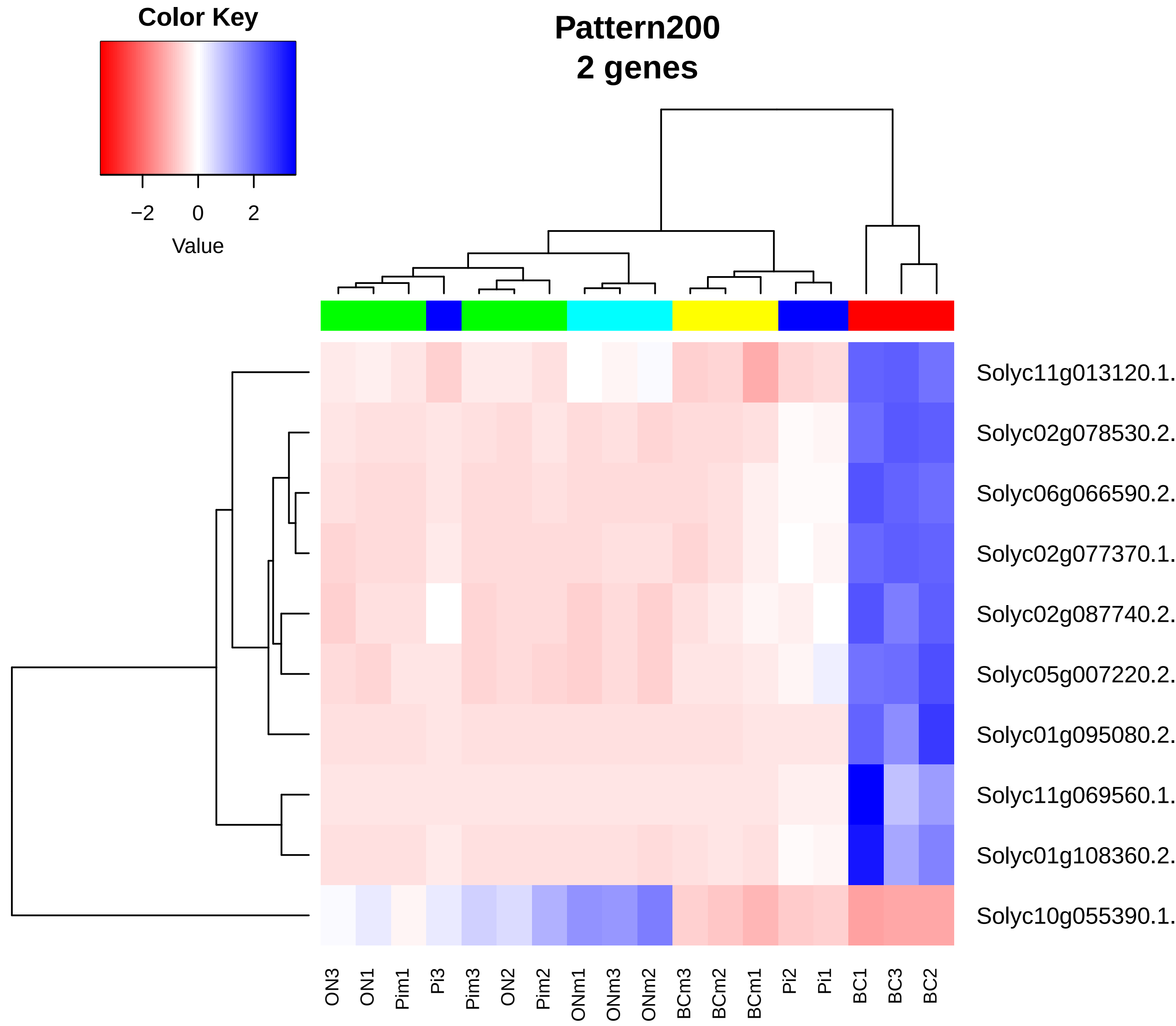
Color Key
−2
0
2
Value
Pattern200
2 genes
Solyc11g013120.1.
Solyc02g078530.2.
Solyc06g066590.2.
Solyc02g077370.1.
Solyc02g087740.2.
Solyc05g007220.2.
Solyc01g095080.2.
Solyc11g069560.1.
Solyc01g108360.2.
Solyc10g055390.1.
ON3
ON1
Pim1
Pi3
Pim3
ON2
Pim2
ONm1
ONm3
ONm2
BCm3
BCm2
BCm1
Pi2
Pi1
BC1
BC3
BC2

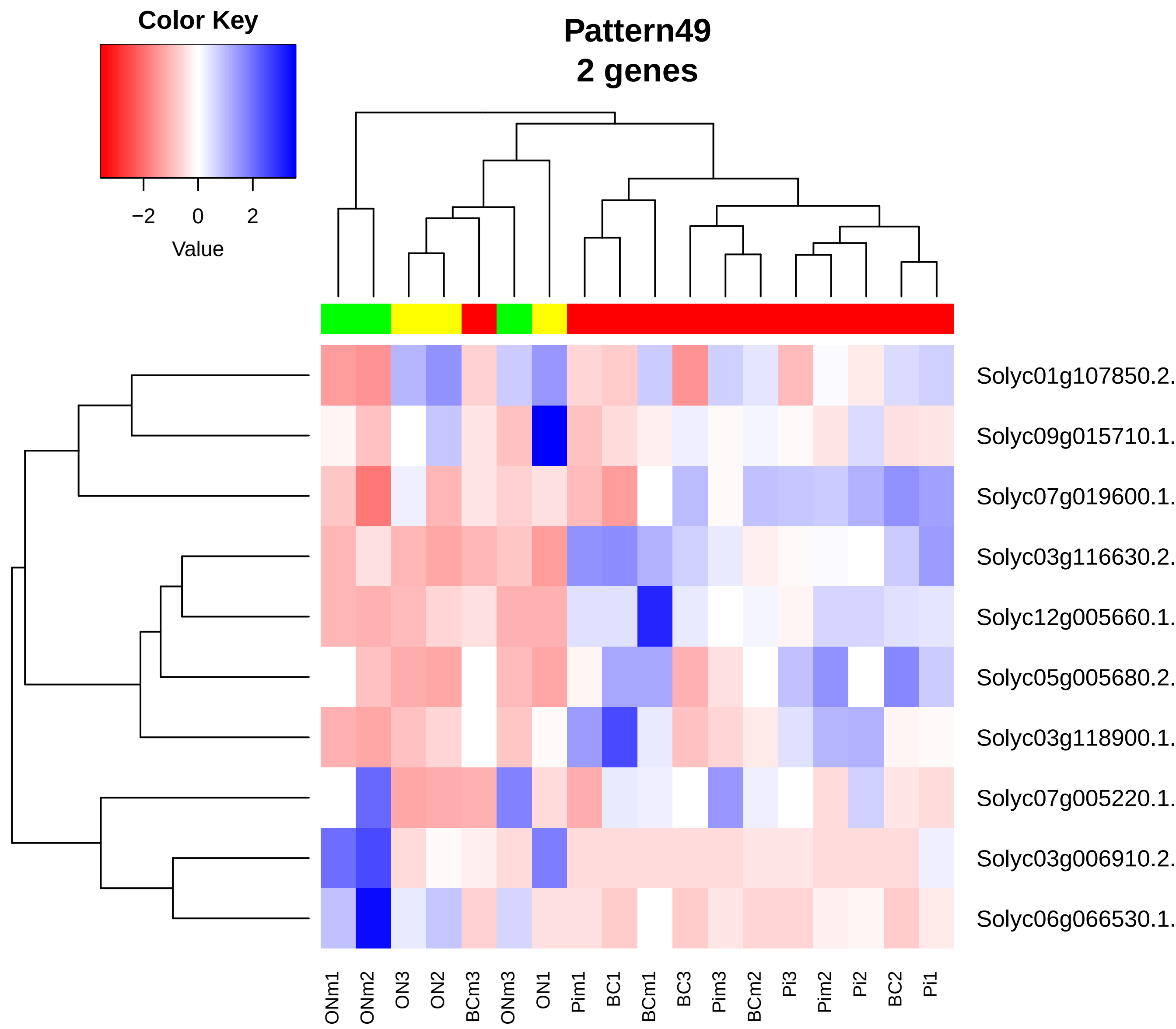

Color Key
−2
0
2
Value
Pattern49
2 genes
Solyc01g107850.2.
Solyc09g015710.1.
Solyc07g019600.1.
Solyc03g116630.2.
Solyc12g005660.1.
Solyc05g005680.2.
Solyc03g118900.1.
Solyc07g005220.1.
Solyc03g006910.2.
Solyc06g066530.1.
ONm1
ONm2
ON3
ON2
BCm3
ONm3
ON1
Pim1
BC1
BCm1
BC3
Pim3
BCm2
Pi3
Pim2
Pi2
BC2
Pi1

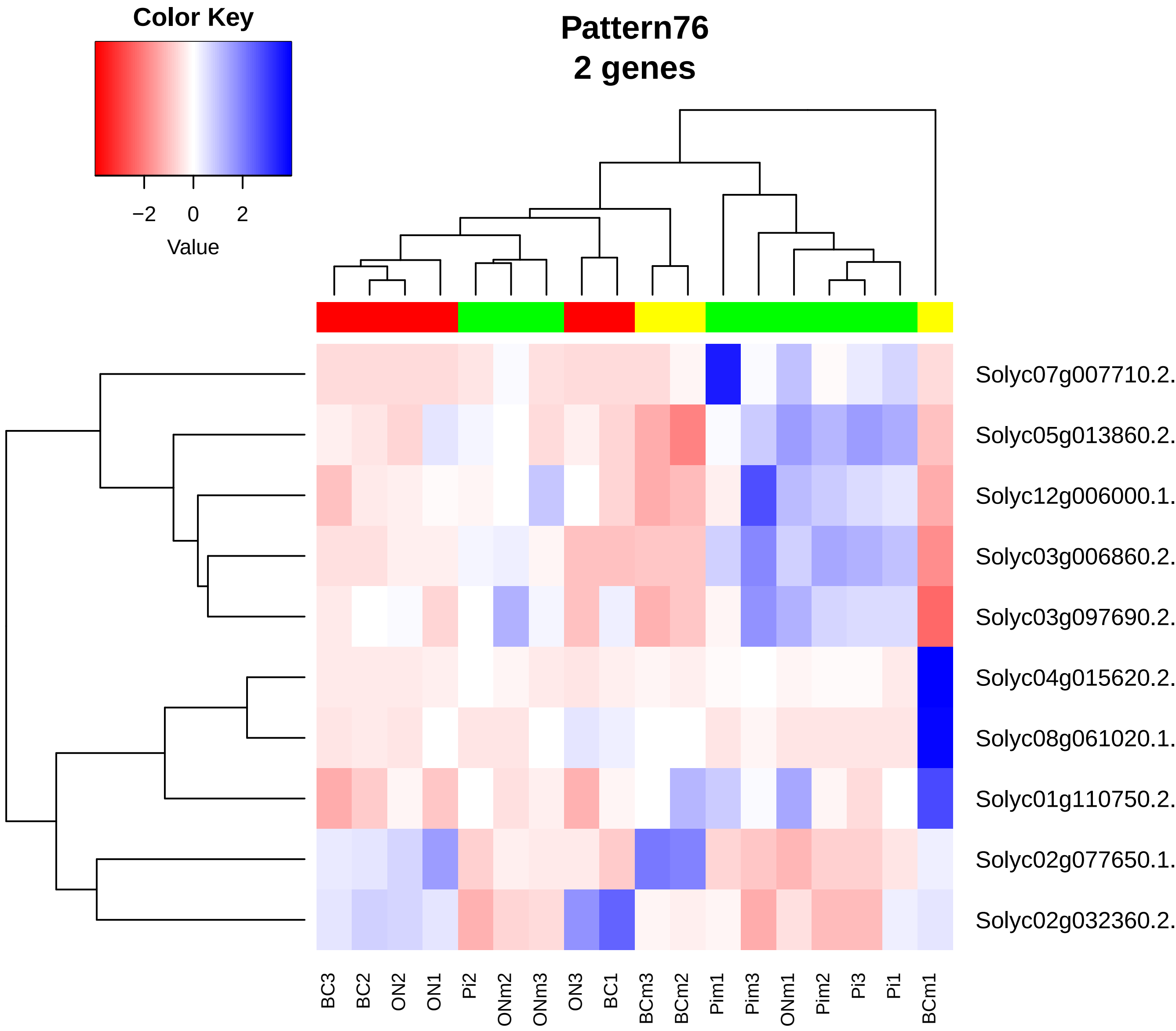
Color Key
−2
0
2
Value
Pattern76
2 genes
Solyc07g007710.2.
Solyc05g013860.2.
Solyc12g006000.1.
Solyc03g006860.2.
Solyc03g097690.2.
Solyc04g015620.2.
Solyc08g061020.1.
Solyc01g110750.2.
Solyc02g077650.1.
Solyc02g032360.2.
BC3
BC2
ON2
ON1
Pi2
ONm2
ONm3
ON3
BC1
BCm3
BCm2
Pim1
Pim3
ONm1
Pim2
Pi3
Pi1
BCm1

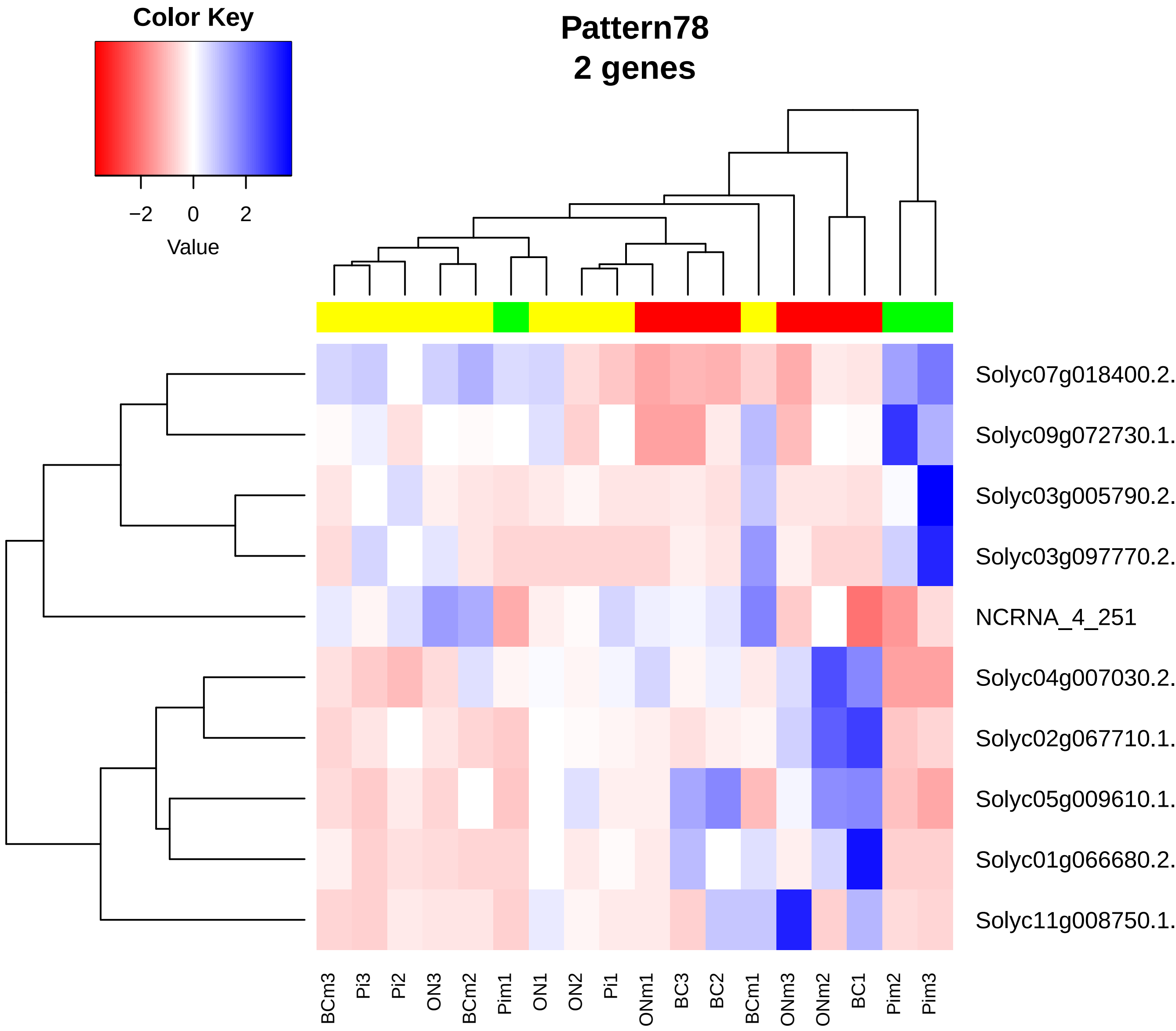
Color Key
−2
0
2
Value
Pattern78
2 genes
Solyc07g018400.2.
Solyc09g072730.1.
Solyc03g005790.2.
Solyc03g097770.2.
NCRNA_4_251
Solyc04g007030.2.
Solyc02g067710.1.
Solyc05g009610.1.
Solyc01g066680.2.
Solyc11g008750.1.
BCm3
Pi3
Pi2
ON3
BCm2
Pim1
ON1
ON2
Pi1
ONm1
BC3
BC2
BCm1
ONm3
ONm2
BC1
Pim2
Pim3

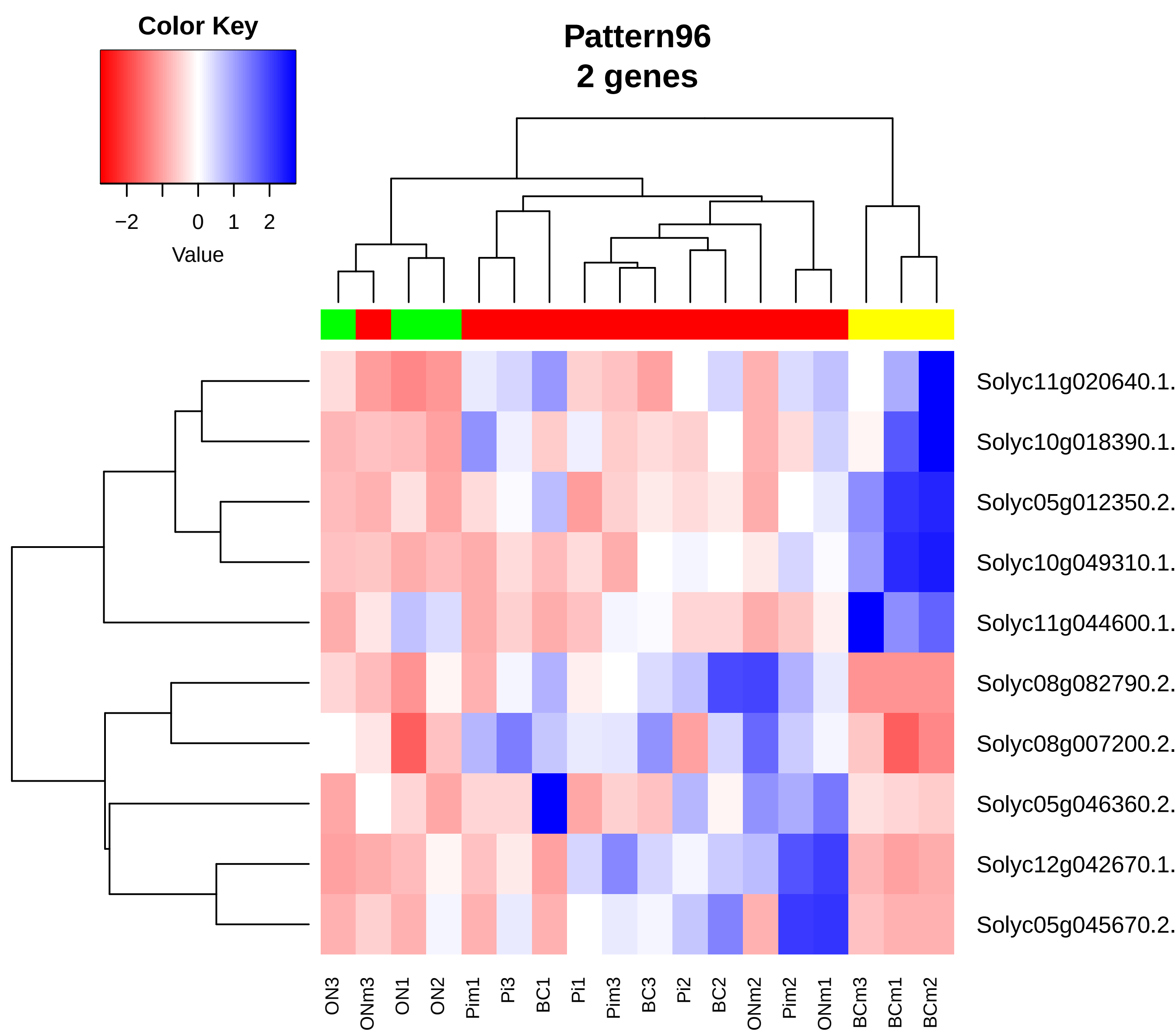
Color Key
−2
0
1
2
Value
Pattern96
2 genes
Solyc11g020640.1.
Solyc10g018390.1.
Solyc05g012350.2.
Solyc10g049310.1.
Solyc11g044600.1.
Solyc08g082790.2.
Solyc08g007200.2.
Solyc05g046360.2.
Solyc12g042670.1.
Solyc05g045670.2.
ON3
ONm3
ON1
ON2
Pim1
Pi3
BC1
Pi1
Pim3
BC3
Pi2
BC2
ONm2
Pim2
ONm1
BCm3
BCm1
BCm2

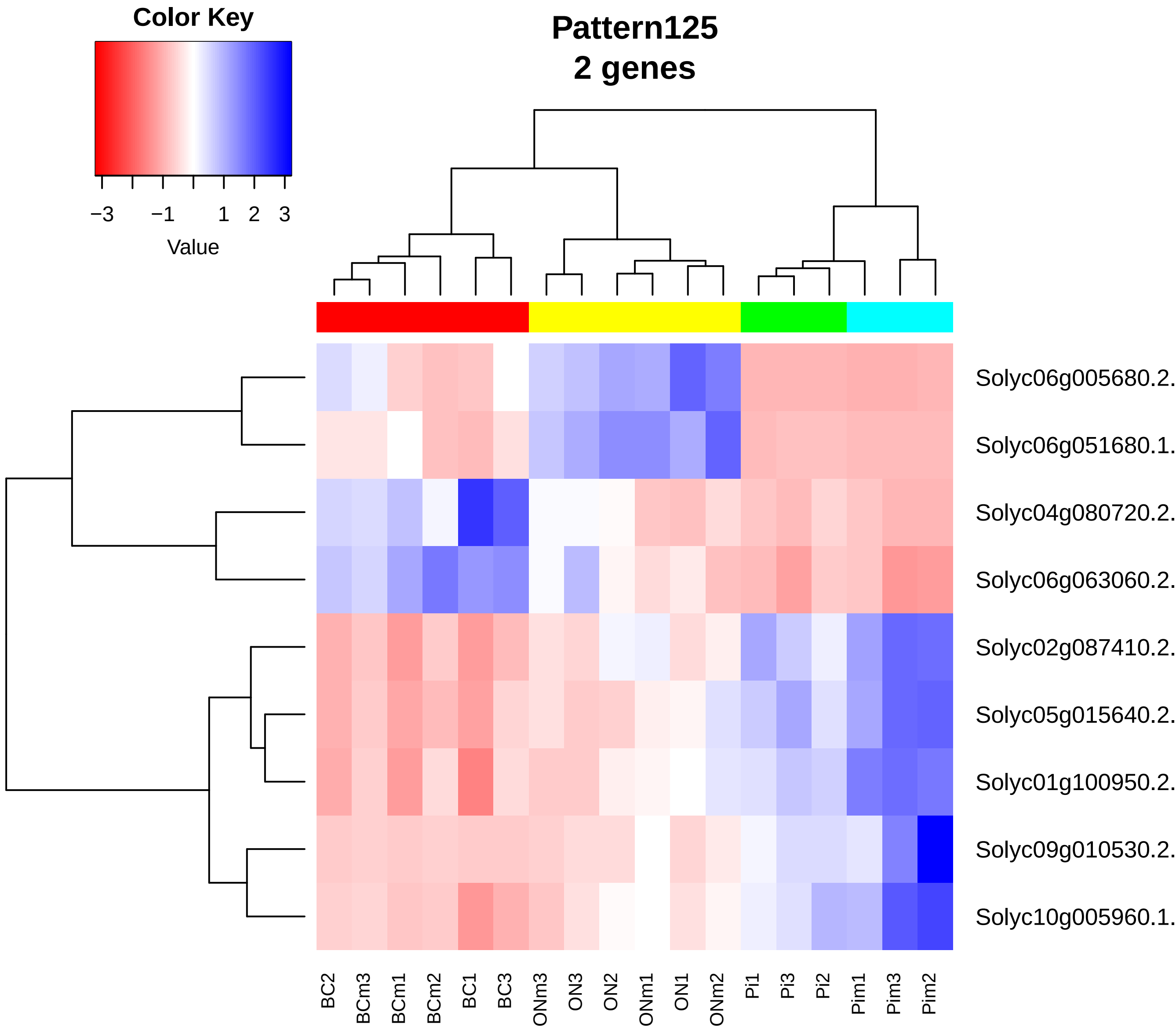
Color Key
−3
−1
1
2
3
Value
Pattern125
2 genes
Solyc06g005680.2.
Solyc06g051680.1.
Solyc04g080720.2.
Solyc06g063060.2.
Solyc02g087410.2.
Solyc05g015640.2.
Solyc01g100950.2.
Solyc09g010530.2.
Solyc10g005960.1.
BC2
BCm3
BCm1
BCm2
BC1
BC3
ONm3
ON3
ON2
ONm1
ON1
ONm2
Pi1
Pi3
Pi2
Pim1
Pim3
Pim2

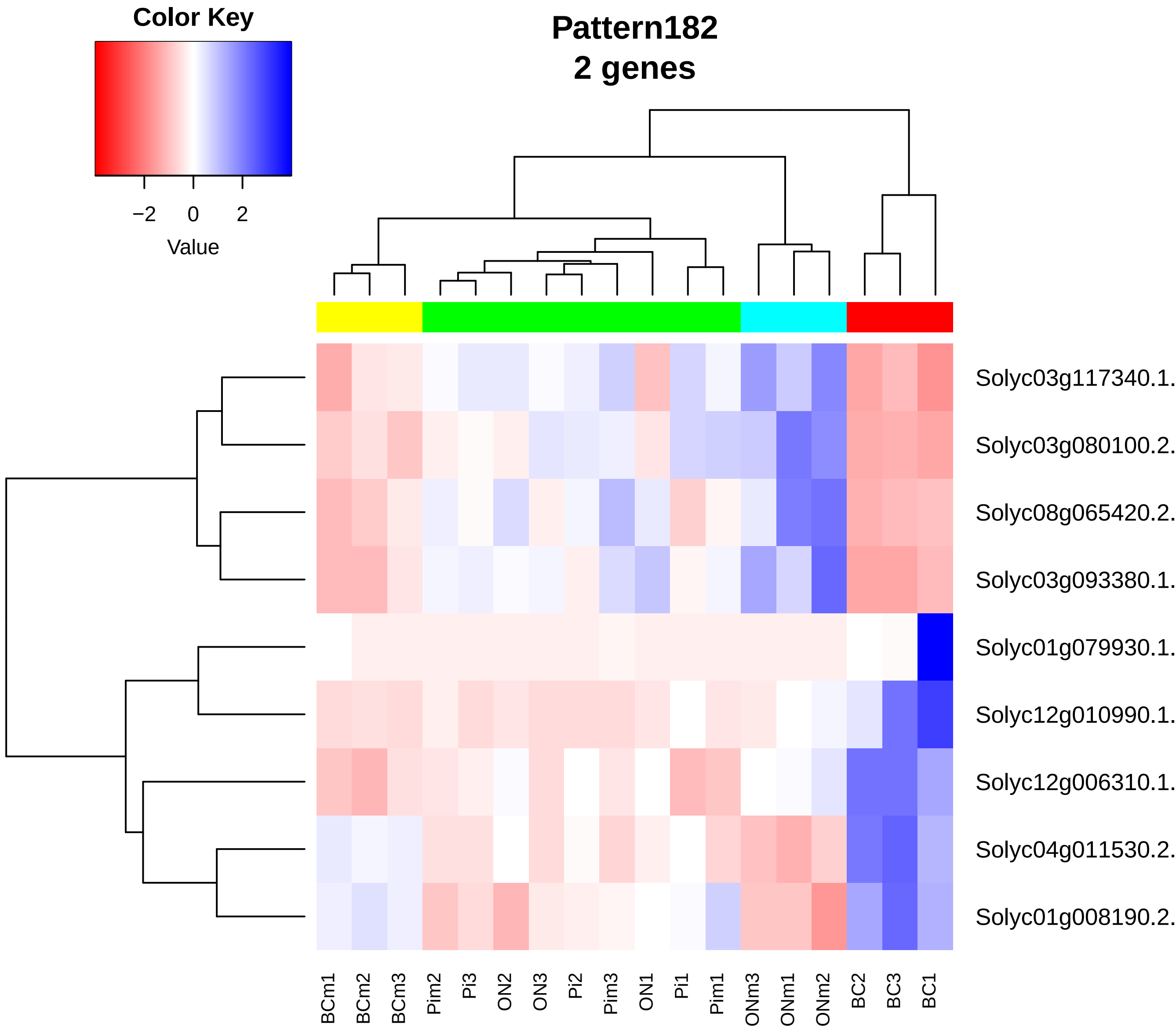

Color Key
−2
0
2
Value
Pattern182
2 genes
Solyc03g117340.1.
Solyc03g080100.2.
Solyc08g065420.2.
Solyc03g093380.1.
Solyc01g079930.1.
Solyc12g010990.1.
Solyc12g006310.1.
Solyc04g011530.2.
Solyc01g008190.2.
BCm1
BCm2
BCm3
Pim2
Pi3
ON2
ON3
Pi2
Pim3
ON1
Pi1
Pim1
ONm3
ONm1
ONm2
BC2
BC3
BC1

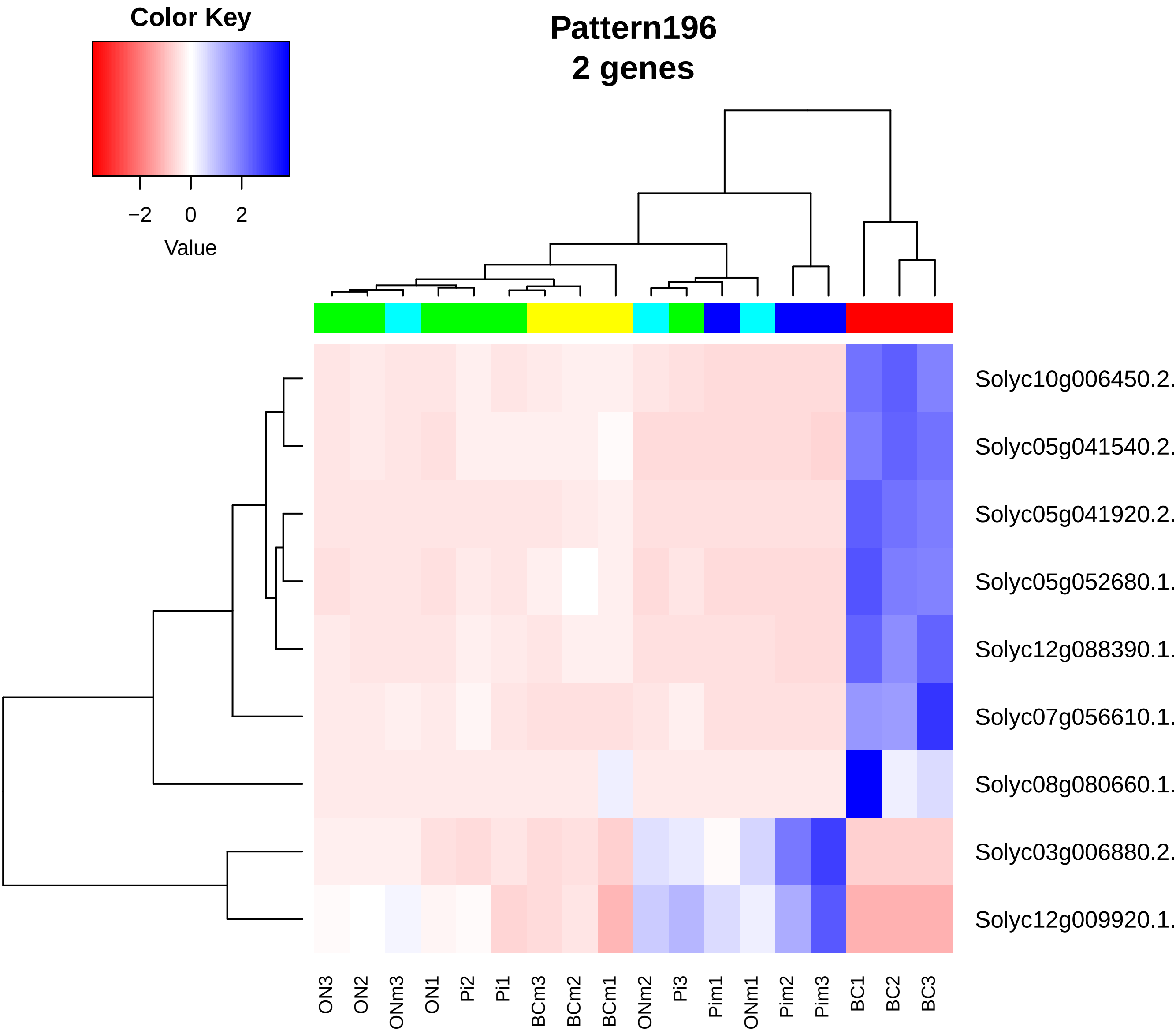
Color Key
−2
0
2
Value
Pattern196
2 genes
Solyc10g006450.2.
Solyc05g041540.2.
Solyc05g041920.2.
Solyc05g052680.1.
Solyc12g088390.1.
Solyc07g056610.1.
Solyc08g080660.1.
Solyc03g006880.2.
Solyc12g009920.1.
ON3
ON2
ONm3
ON1
Pi2
Pi1
BCm3
BCm2
BCm1
ONm2
Pi3
Pim1
ONm1
Pim2
Pim3
BC1
BC2
BC3

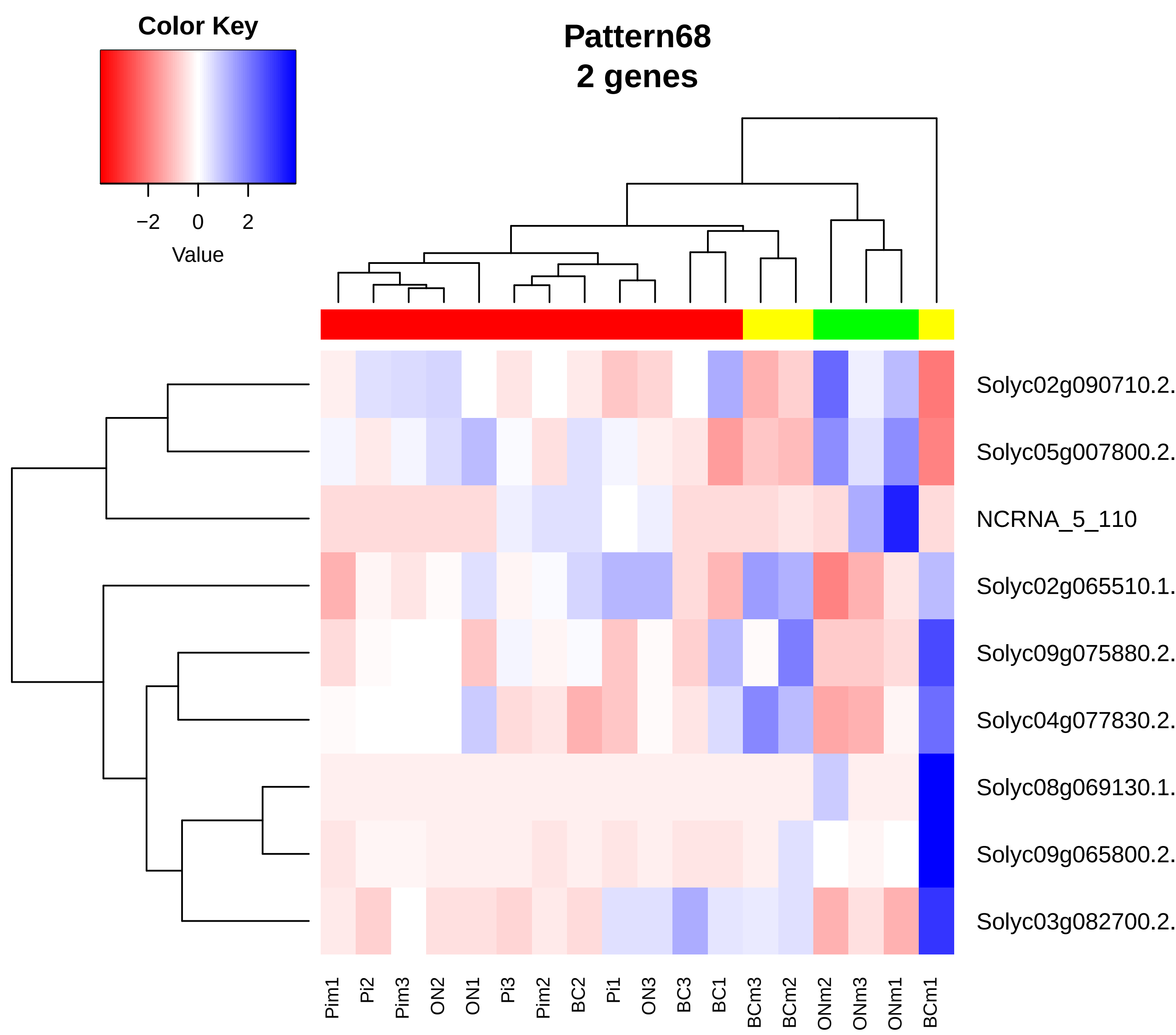
Color Key
−2
0
2
Value
Pattern68
2 genes
Solyc02g090710.2.
Solyc05g007800.2.
NCRNA_5_110
Solyc02g065510.1.
Solyc09g075880.2.
Solyc04g077830.2.
Solyc08g069130.1.
Solyc09g065800.2.
Solyc03g082700.2.
Pim1
Pi2
Pim3
ON2
ON1
Pi3
Pim2
BC2
Pi1
ON3
BC3
BC1
BCm3
BCm2
ONm2
ONm3
ONm1
BCm1

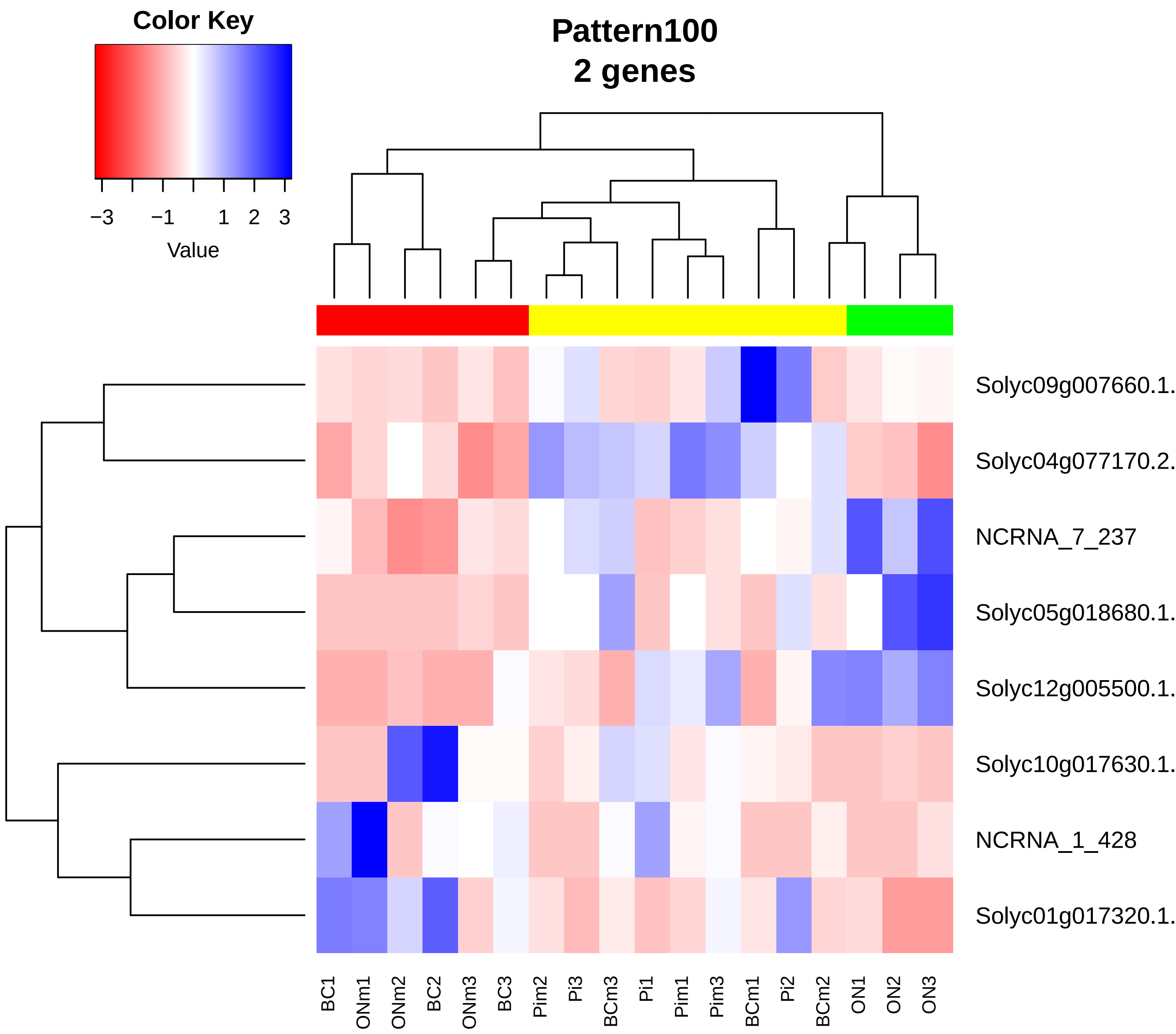

Color Key
−3
−1
1
2
3
Value
Pattern100
2 genes
Solyc09g007660.1.
Solyc04g077170.2.
NCRNA_7_237
Solyc05g018680.1.
Solyc12g005500.1.
Solyc10g017630.1.
NCRNA_1_428
Solyc01g017320.1.
BC1
ONm1
ONm2
BC2
ONm3
BC3
Pim2
Pi3
BCm3
Pi1
Pim1
Pim3
BCm1
Pi2
BCm2
ON1
ON2
ON3

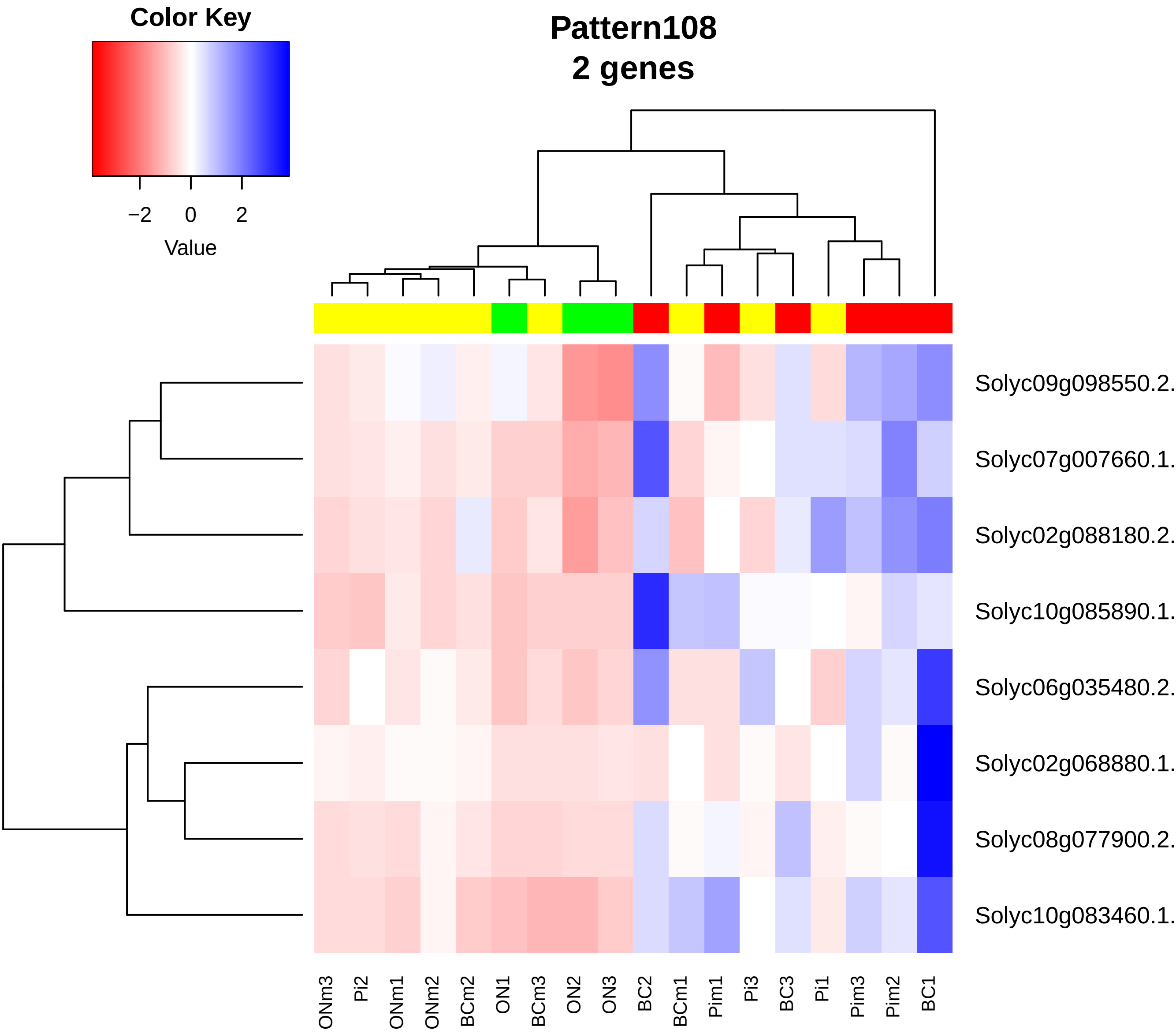
Color Key
−2
0
2
Value
Pattern108
2 genes
Solyc09g098550.2.
Solyc07g007660.1.
Solyc02g088180.2.
Solyc10g085890.1.
Solyc06g035480.2.
Solyc02g068880.1.
Solyc08g077900.2.
Solyc10g083460.1.
ONm3
Pi2
ONm1
ONm2
BCm2
ON1
BCm3
ON2
ON3
BC2
BCm1
Pim1
Pi3
BC3
Pi1
Pim3
Pim2
BC1

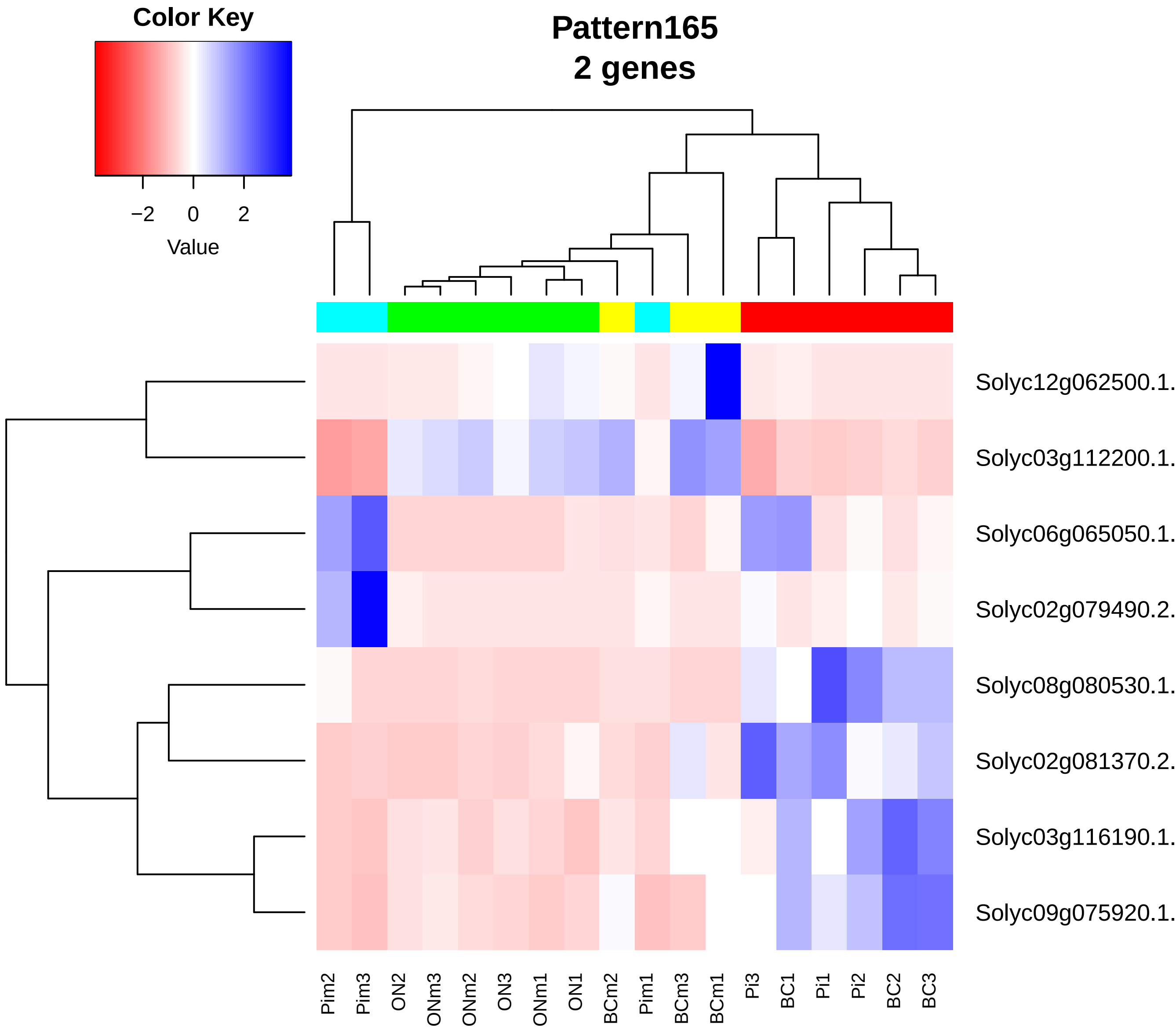
Color Key
−2
0
2
Value
Pattern165
2 genes
Solyc12g062500.1.
Solyc03g112200.1.
Solyc06g065050.1.
Solyc02g079490.2.
Solyc08g080530.1.
Solyc02g081370.2.
Solyc03g116190.1.
Solyc09g075920.1.
Pim2
Pim3
ON2
ONm3
ONm2
ON3
ONm1
ON1
BCm2
Pim1
BCm3
BCm1
Pi3
BC1
Pi1
Pi2
BC2
BC3

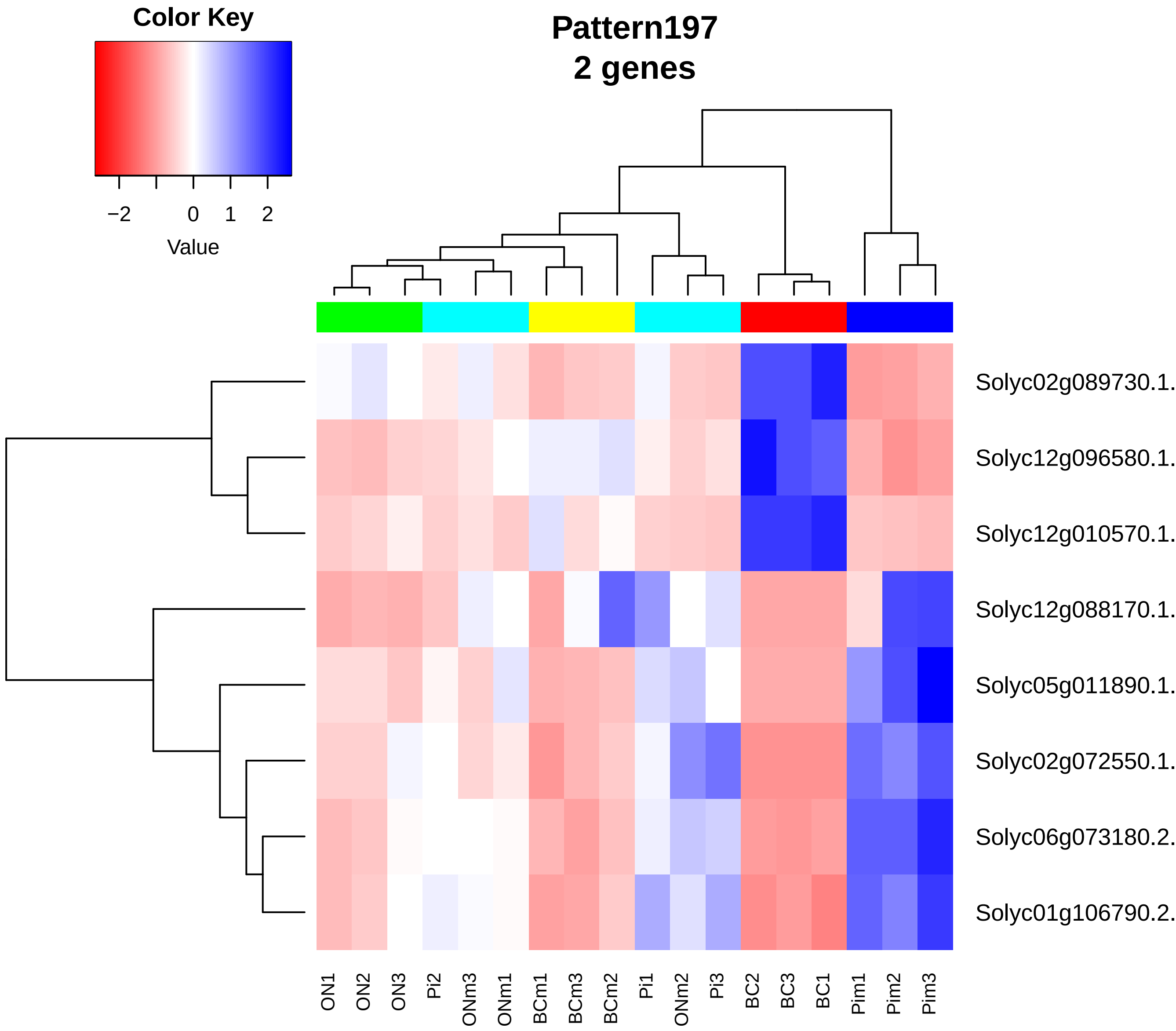
Color Key
−2
0
1
2
Value
Pattern197
2 genes
Solyc02g089730.1.
Solyc12g096580.1.
Solyc12g010570.1.
Solyc12g088170.1.
Solyc05g011890.1.
Solyc02g072550.1.
Solyc06g073180.2.
Solyc01g106790.2.
ON1
ON2
ON3
Pi2
ONm3
ONm1
BCm1
BCm3
BCm2
Pi1
ONm2
Pi3
BC2
BC3
BC1
Pim1
Pim2
Pim3

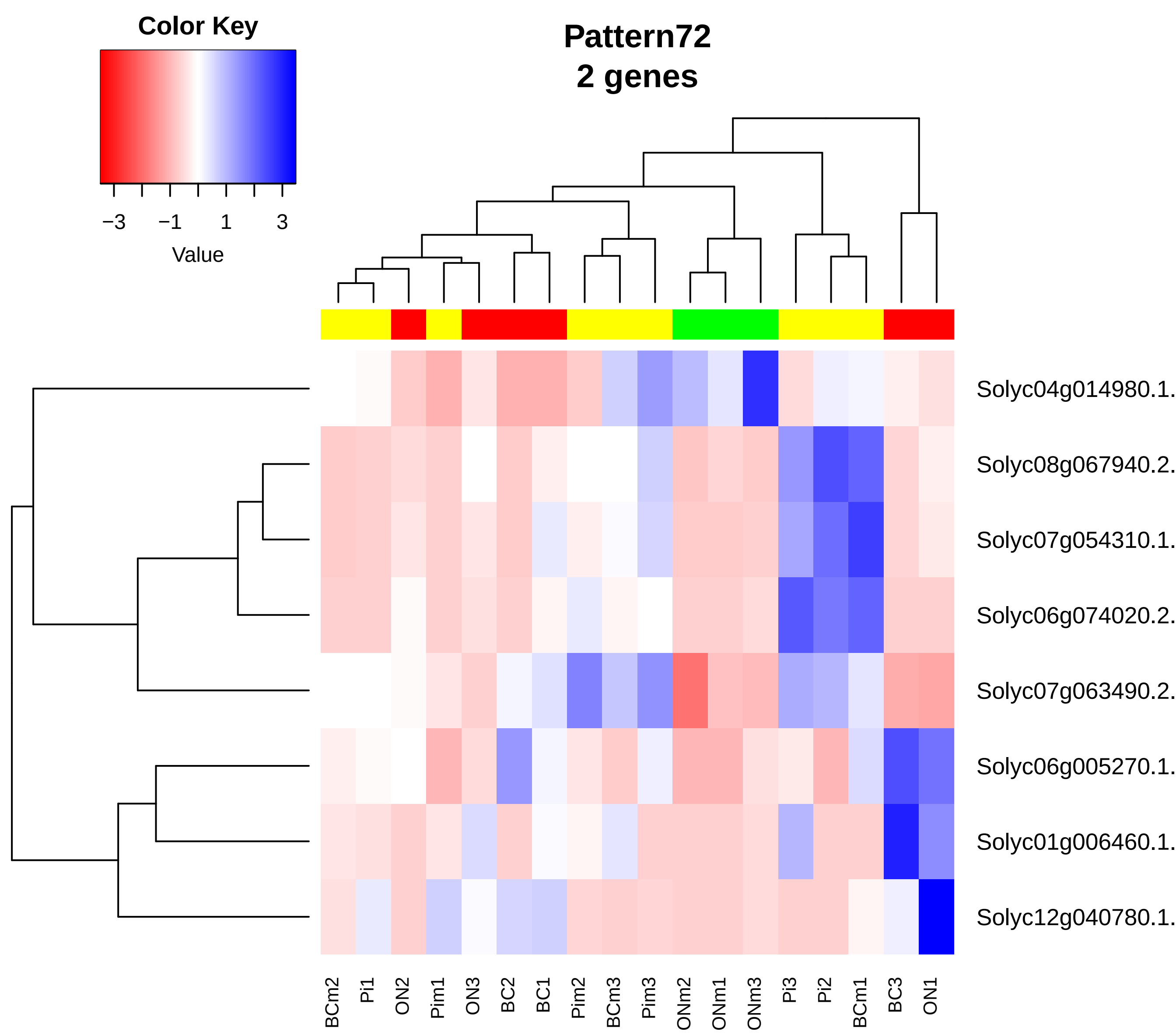
Color Key
−3
−1
1
3
Value
Pattern72
2 genes
Solyc04g014980.1.
Solyc08g067940.2.
Solyc07g054310.1.
Solyc06g074020.2.
Solyc07g063490.2.
Solyc06g005270.1.
Solyc01g006460.1.
Solyc12g040780.1.
BCm2
Pi1
ON2
Pim1
ON3
BC2
BC1
Pim2
BCm3
Pim3
ONm2
ONm1
ONm3
Pi3
Pi2
BCm1
BC3
ON1

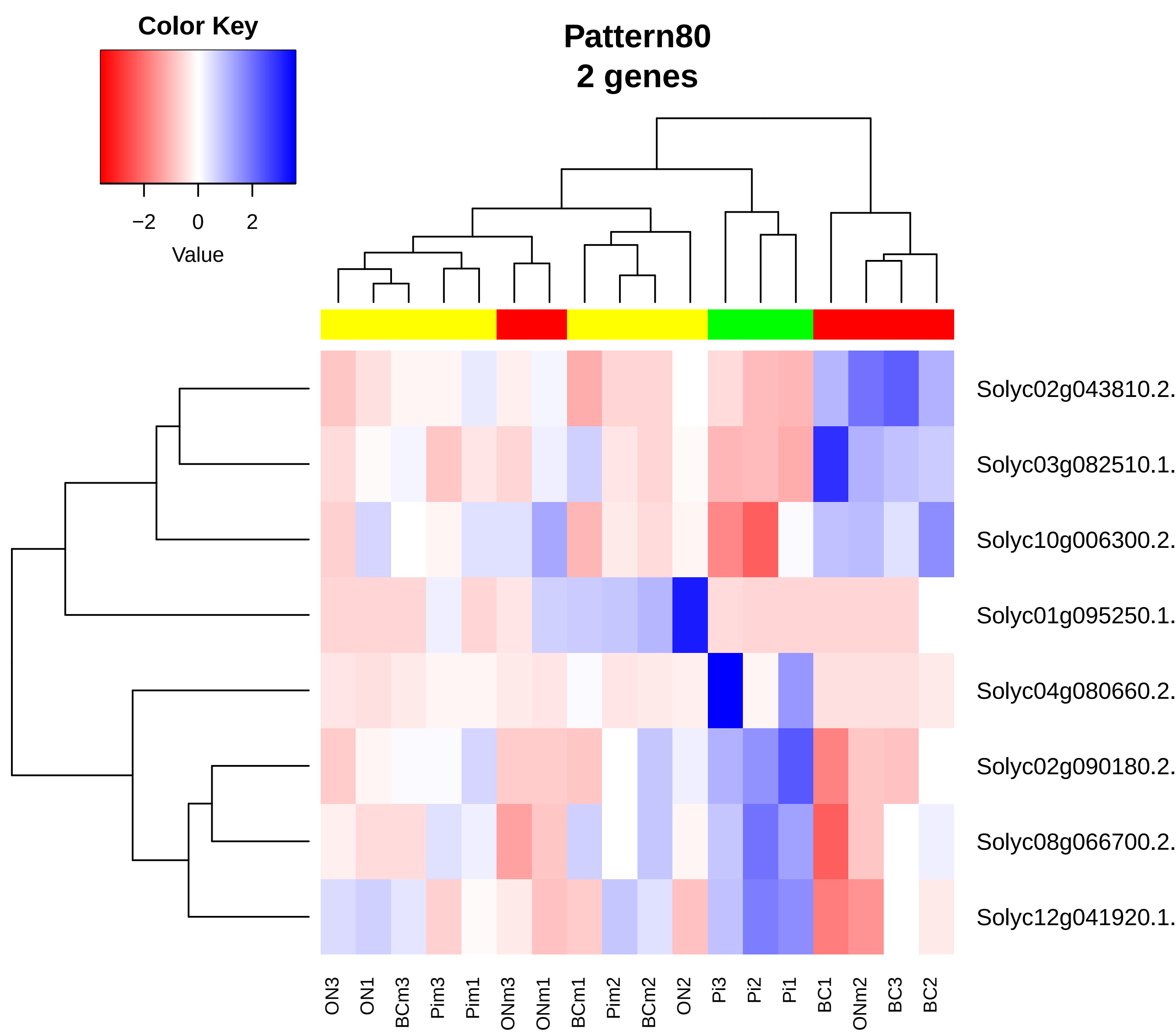

Color Key
−2
0
2
Value
Pattern80
2 genes
Solyc02g043810.2.
Solyc03g082510.1.
Solyc10g006300.2.
Solyc01g095250.1.
Solyc04g080660.2.
Solyc02g090180.2.
Solyc08g066700.2.
Solyc12g041920.1.
ON3
ON1
BCm3
Pim3
Pim1
ONm3
ONm1
BCm1
Pim2
BCm2
ON2
Pi3
Pi2
Pi1
BC1
ONm2
BC3
BC2

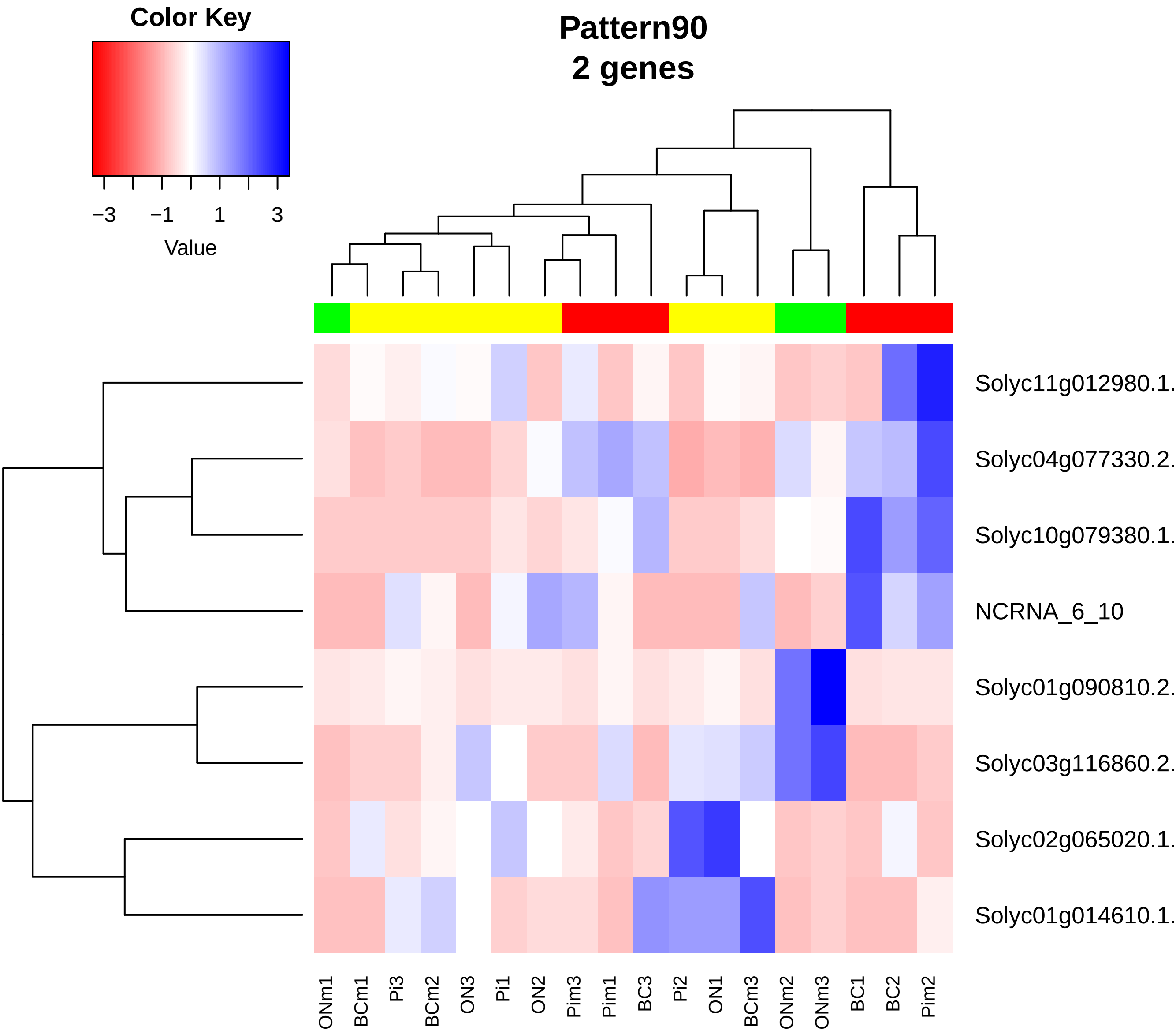
Color Key
−3
−1
1
3
Value
Pattern90
2 genes
Solyc11g012980.1.
Solyc04g077330.2.
Solyc10g079380.1.
NCRNA_6_10
Solyc01g090810.2.
Solyc03g116860.2.
Solyc02g065020.1.
Solyc01g014610.1.
ONm1
BCm1
Pi3
BCm2
ON3
Pi1
ON2
Pim3
Pim1
BC3
Pi2
ON1
BCm3
ONm2
ONm3
BC1
BC2
Pim2

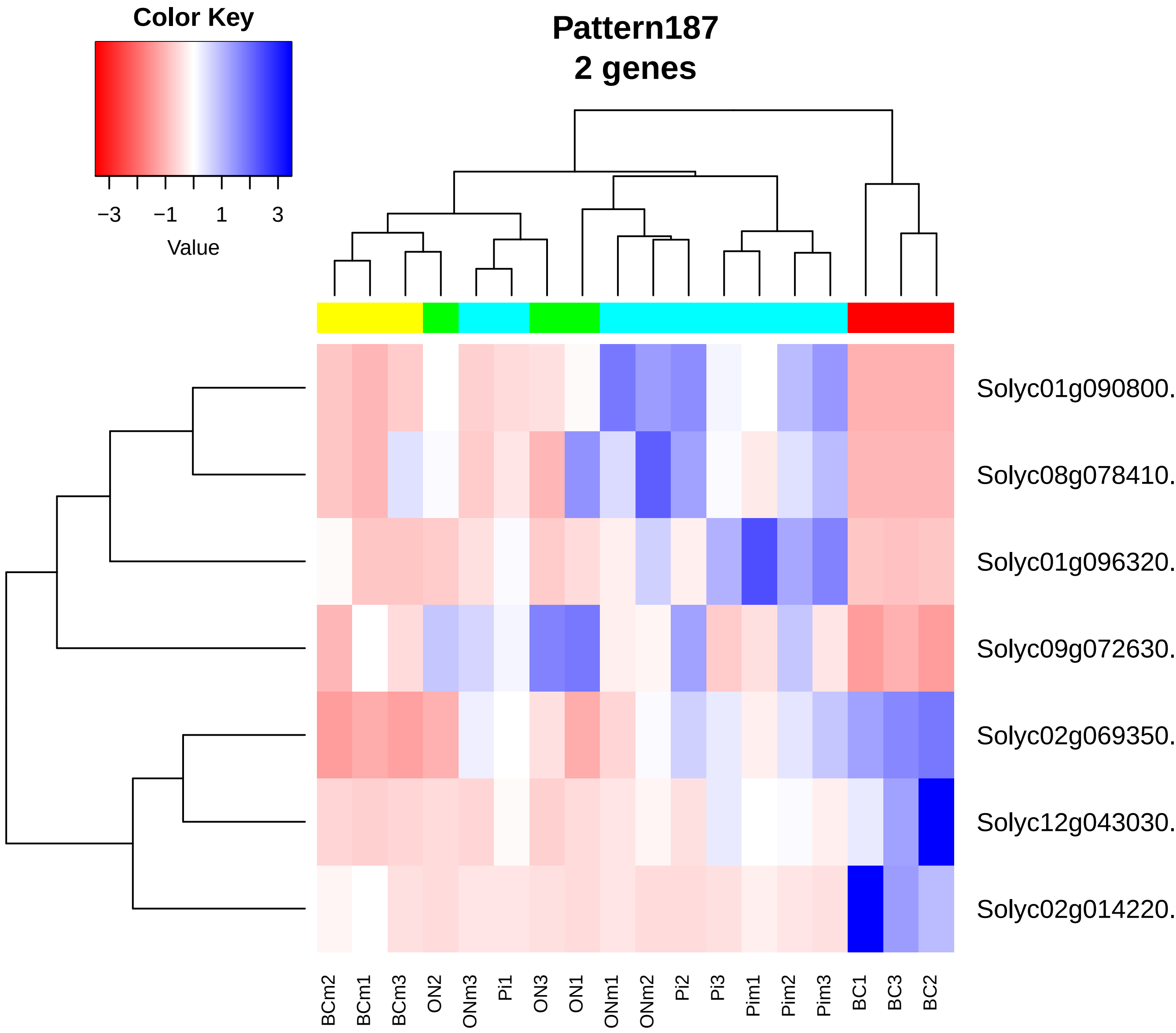
Color Key
−3
−1
1
3
Value
Pattern187
2 genes
Solyc01g090800.
Solyc08g078410.
Solyc01g096320.
Solyc09g072630.
Solyc02g069350.
Solyc12g043030.
Solyc02g014220.
BCm2
BCm1
BCm3
ON2
ONm3
Pi1
ON3
ON1
ONm1
ONm2
Pi2
Pi3
Pim1
Pim2
Pim3
BC1
BC3
BC2

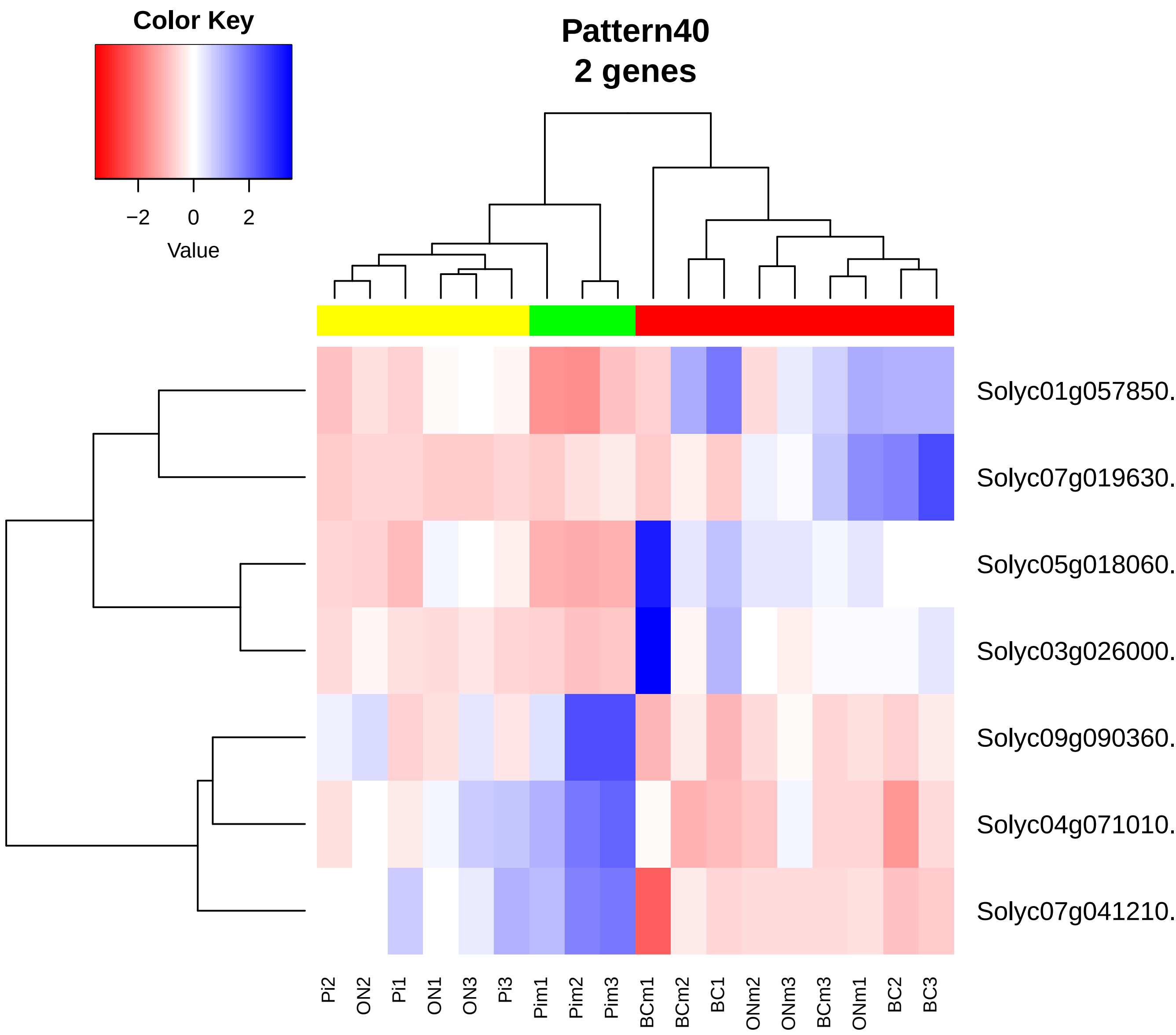

Color Key
−2
0
2
Value
Pattern40
2 genes
Solyc01g057850.
Solyc07g019630.
Solyc05g018060.
Solyc03g026000.
Solyc09g090360.
Solyc04g071010.
Solyc07g041210.
Pi2
ON2
Pi1
ON1
ON3
Pi3
Pim1
Pim2
Pim3
BCm1
BCm2
BC1
ONm2
ONm3
BCm3
ONm1
BC2
BC3

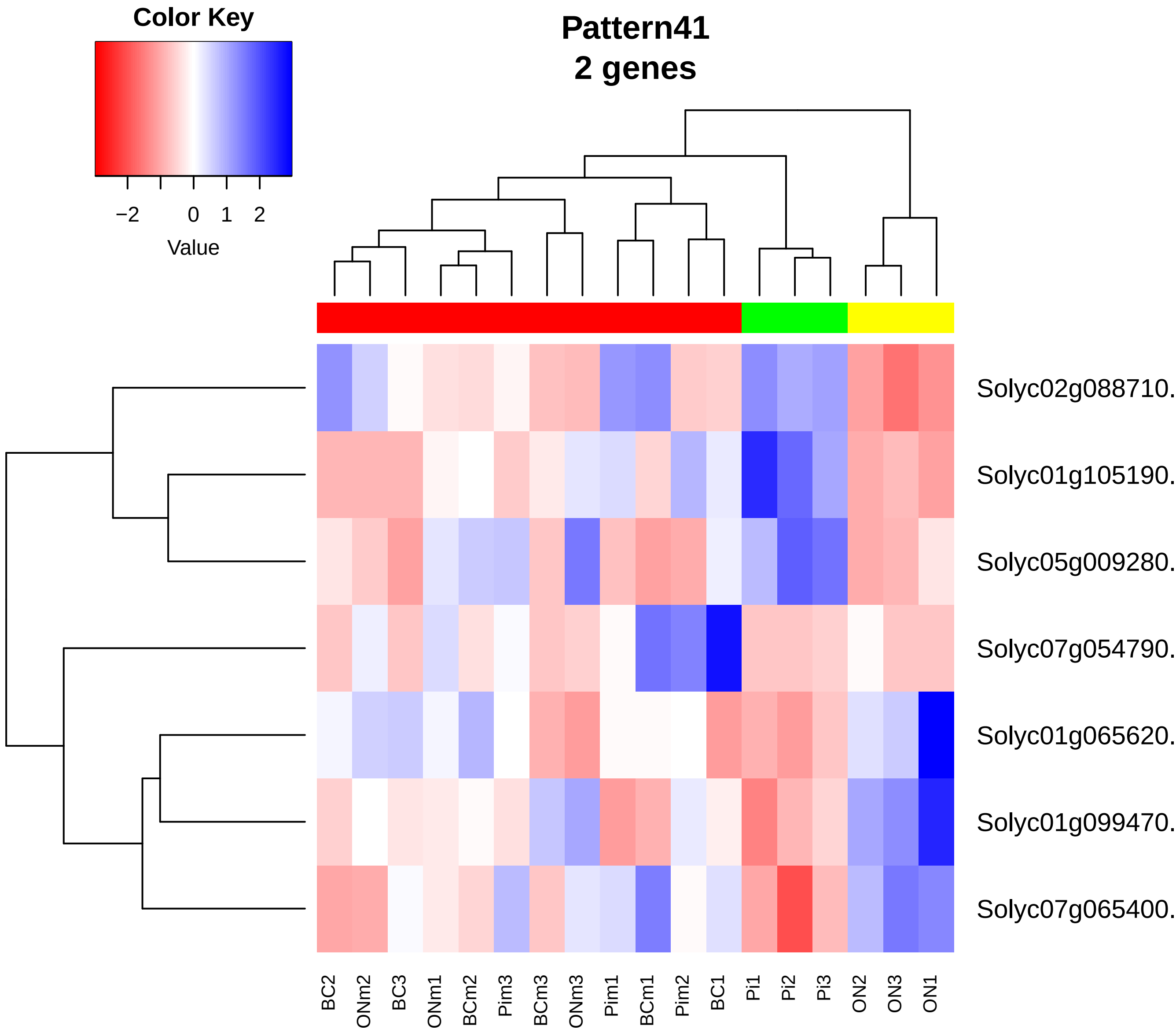
Color Key
−2
0
1
2
Value
Pattern41
2 genes
Solyc02g088710.
Solyc01g105190.
Solyc05g009280.
Solyc07g054790.
Solyc01g065620.
Solyc01g099470.
Solyc07g065400.
BC2
ONm2
BC3
ONm1
BCm2
Pim3
BCm3
ONm3
Pim1
BCm1
Pim2
BC1
Pi1
Pi2
Pi3
ON2
ON3
ON1

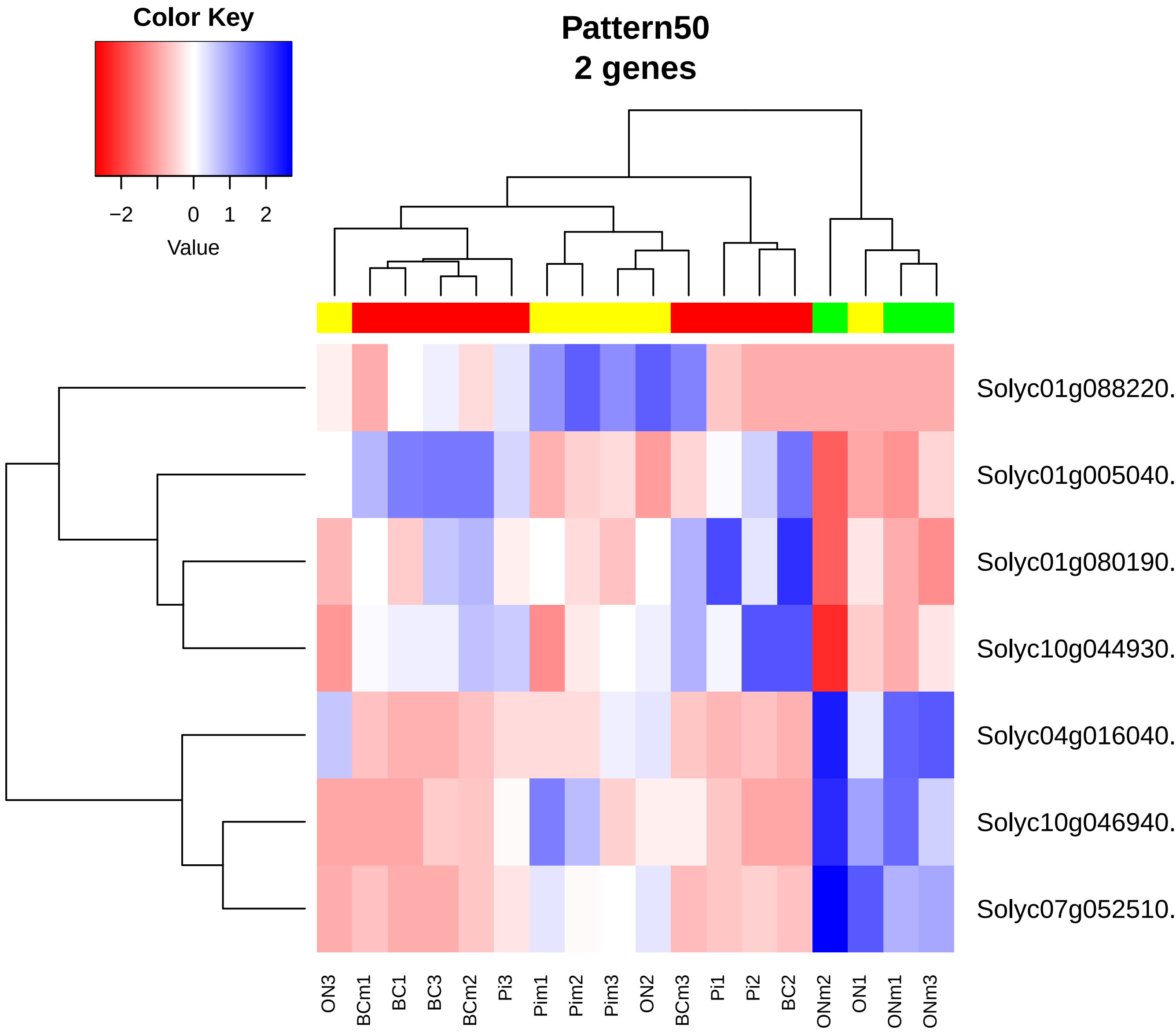
Color Key
−2
0
1
2
Value
Pattern50
2 genes
Solyc01g088220.
Solyc01g005040.
Solyc01g080190.
Solyc10g044930.
Solyc04g016040.
Solyc10g046940.
Solyc07g052510.
ON3
BCm1
BC1
BC3
BCm2
Pi3
Pim1
Pim2
Pim3
ON2
BCm3
Pi1
Pi2
BC2
ONm2
ON1
ONm1
ONm3

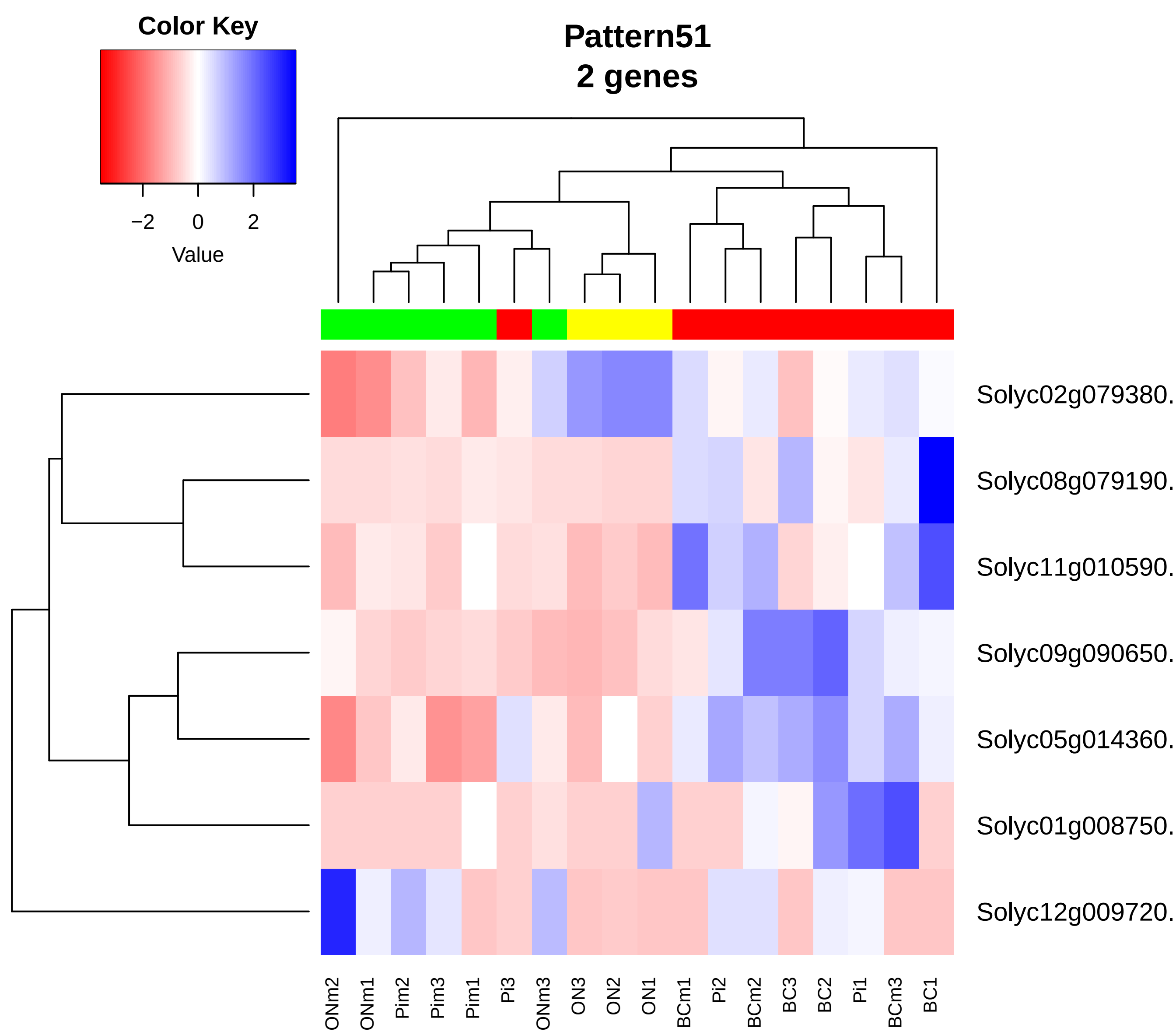
Color Key
−2
0
2
Value
Pattern51
2 genes
Solyc02g079380.
Solyc08g079190.
Solyc11g010590.
Solyc09g090650.
Solyc05g014360.
Solyc01g008750.
Solyc12g009720.
ONm2
ONm1
Pim2
Pim3
Pim1
Pi3
ONm3
ON3
ON2
ON1
BCm1
Pi2
BCm2
BC3
BC2
Pi1
BCm3
BC1

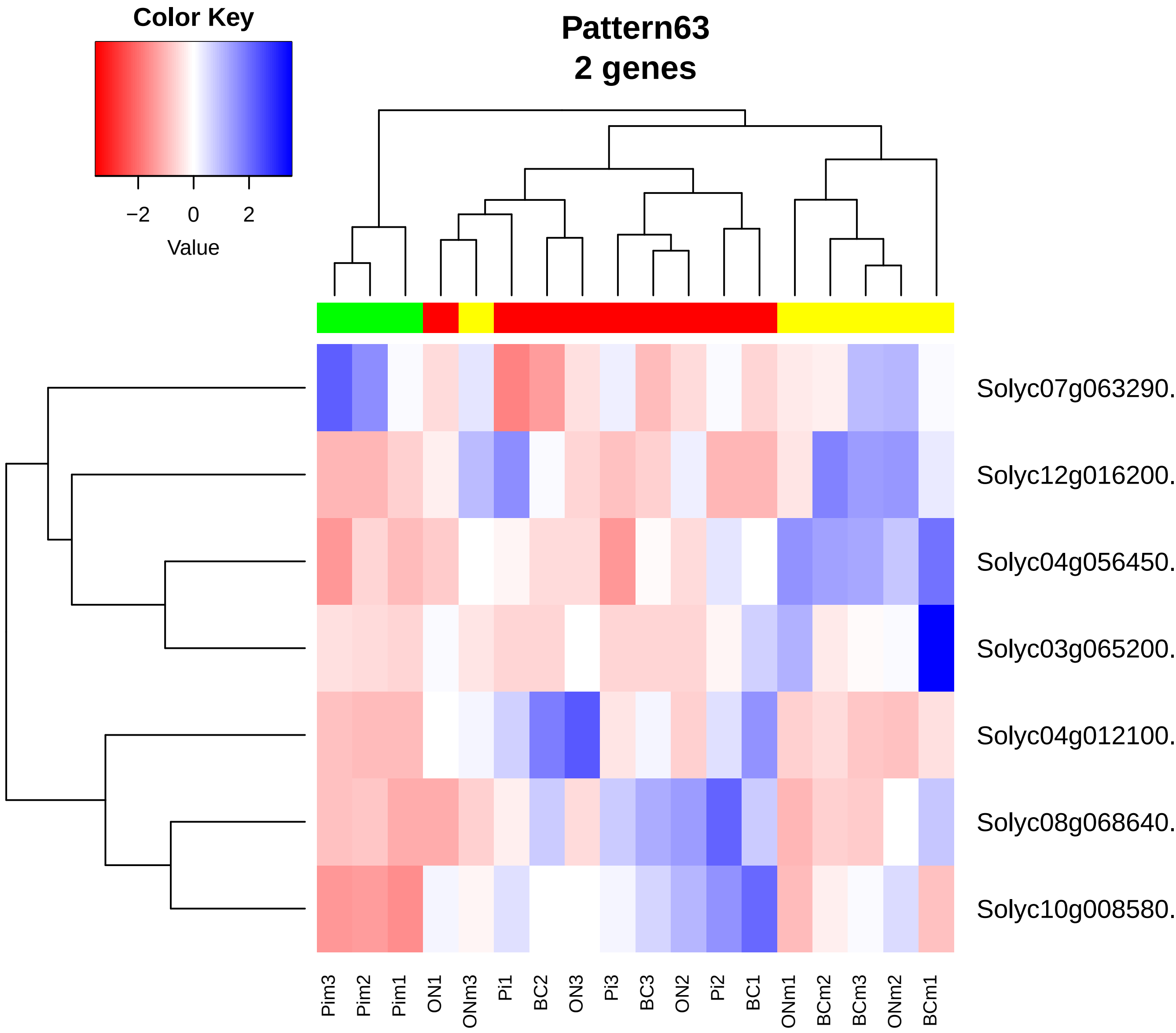
Color Key
−2
0
2
Value
Pattern63
2 genes
Solyc07g063290.
Solyc12g016200.
Solyc04g056450.
Solyc03g065200.
Solyc04g012100.
Solyc08g068640.
Solyc10g008580.
Pim3
Pim2
Pim1
ON1
ONm3
Pi1
BC2
ON3
Pi3
BC3
ON2
Pi2
BC1
ONm1
BCm2
BCm3
ONm2
BCm1

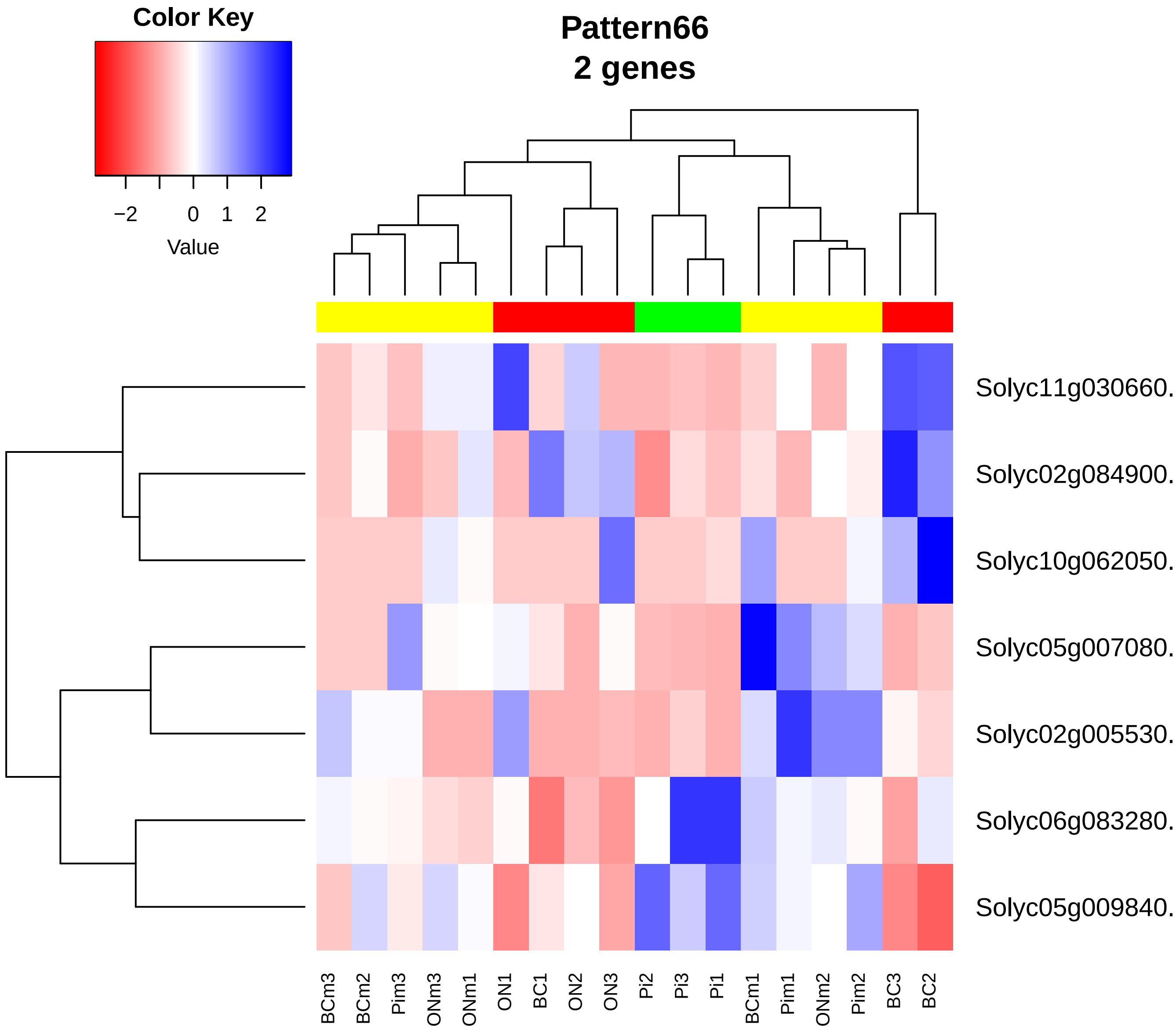
Color Key
−2
0
1
2
Value
Pattern66
2 genes
Solyc11g030660.
Solyc02g084900.
Solyc10g062050.
Solyc05g007080.
Solyc02g005530.
Solyc06g083280.
Solyc05g009840.
BCm3
BCm2
Pim3
ONm3
ONm1
ON1
BC1
ON2
ON3
Pi2
Pi3
Pi1
BCm1
Pim1
ONm2
Pim2
BC3
BC2

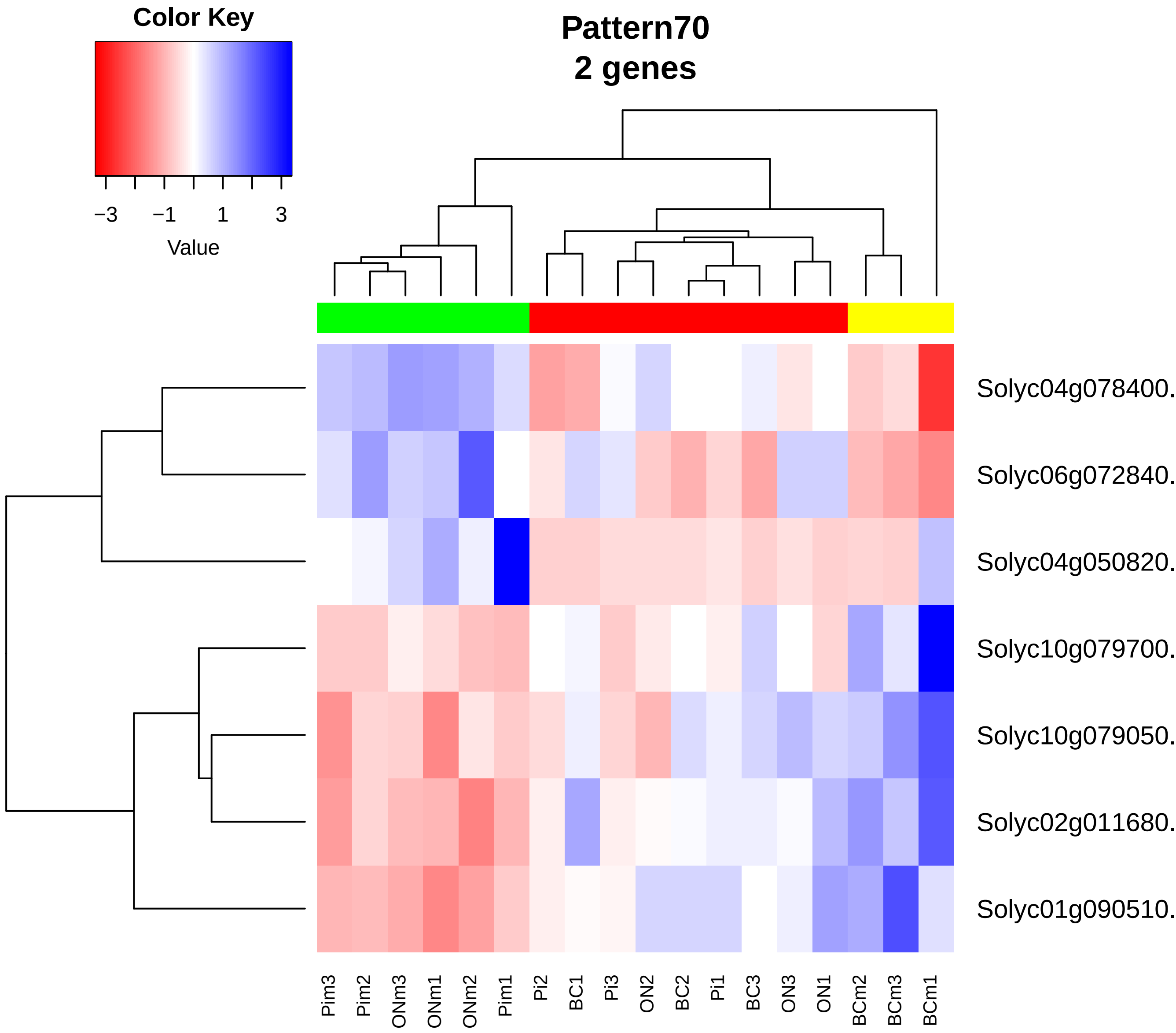
Color Key
−3
−1
1
3
Value
Pattern70
2 genes
Solyc04g078400.
Solyc06g072840.
Solyc04g050820.
Solyc10g079700.
Solyc10g079050.
Solyc02g011680.
Solyc01g090510.
Pim3
Pim2
ONm3
ONm1
ONm2
Pim1
Pi2
BC1
Pi3
ON2
BC2
Pi1
BC3
ON3
ON1
BCm2
BCm3
BCm1

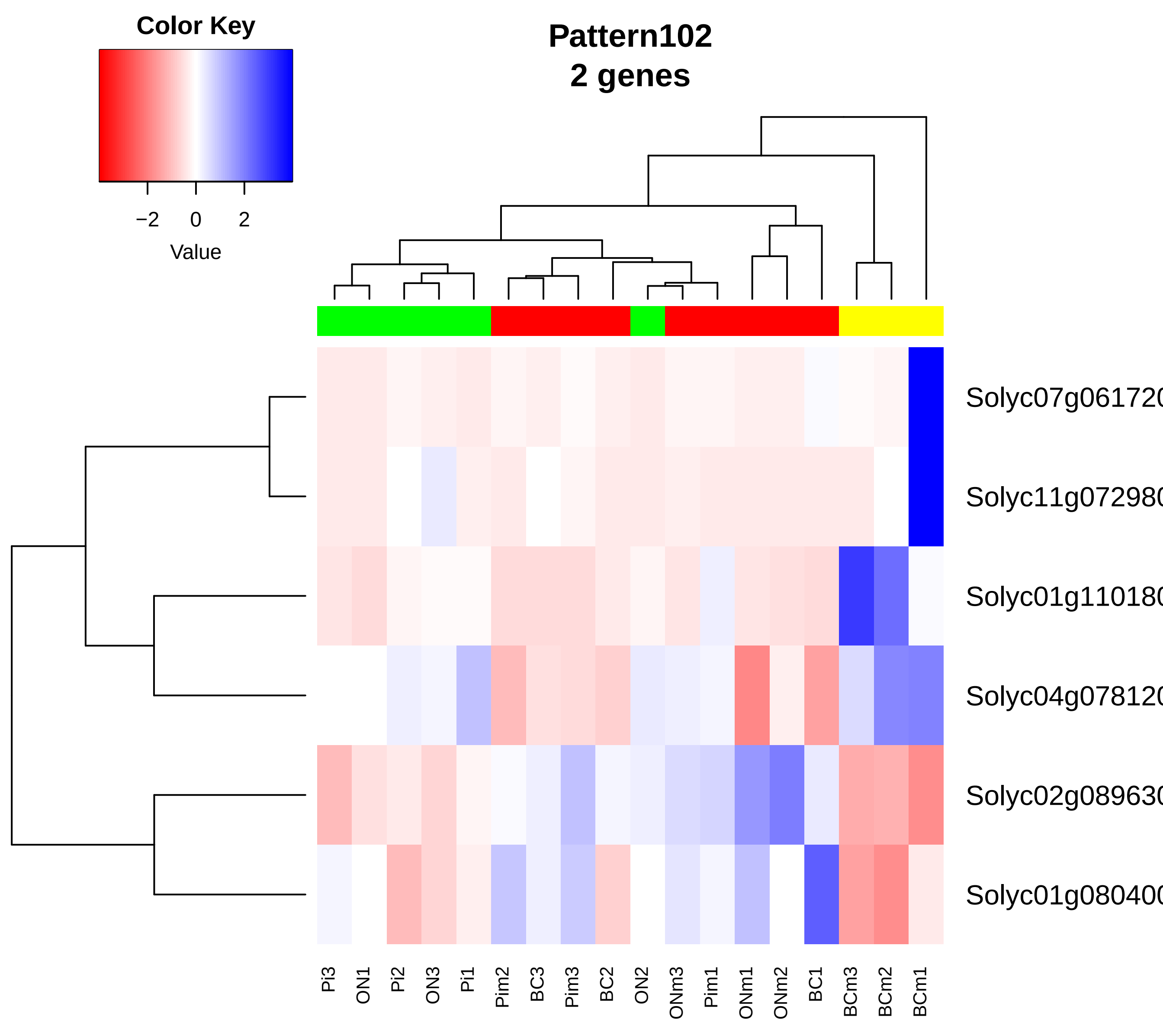
Color Key
−2
0
2
Value
Pattern102
2 genes
Solyc07g06172
Solyc11g07298
Solyc01g11018
Solyc04g07812
Solyc02g08963
Solyc01g08040
Pi3
ON1
Pi2
ON3
Pi1
Pim2
BC3
Pim3
BC2
ON2
ONm3
Pim1
ONm1
ONm2
BC1
BCm3
BCm2
BCm1

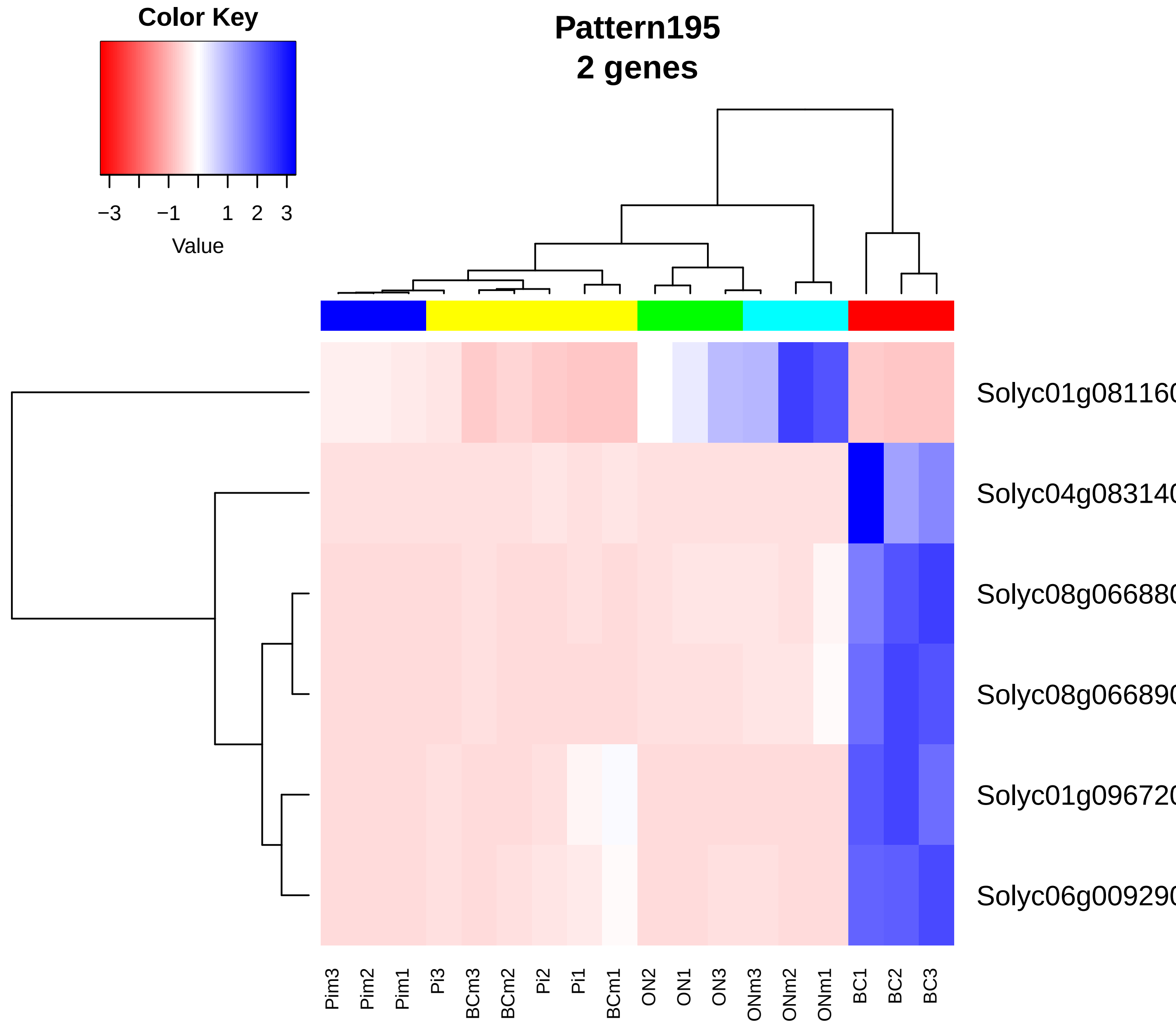
Color Key
−3
−1
1
2
3
Value
Pattern195
2 genes
Solyc01g08116
Solyc04g08314
Solyc08g06688
Solyc08g06689
Solyc01g09672
Solyc06g00929
Pim3
Pim2
Pim1
Pi3
BCm3
BCm2
Pi2
Pi1
BCm1
ON2
ON1
ON3
ONm3
ONm2
ONm1
BC1
BC2
BC3

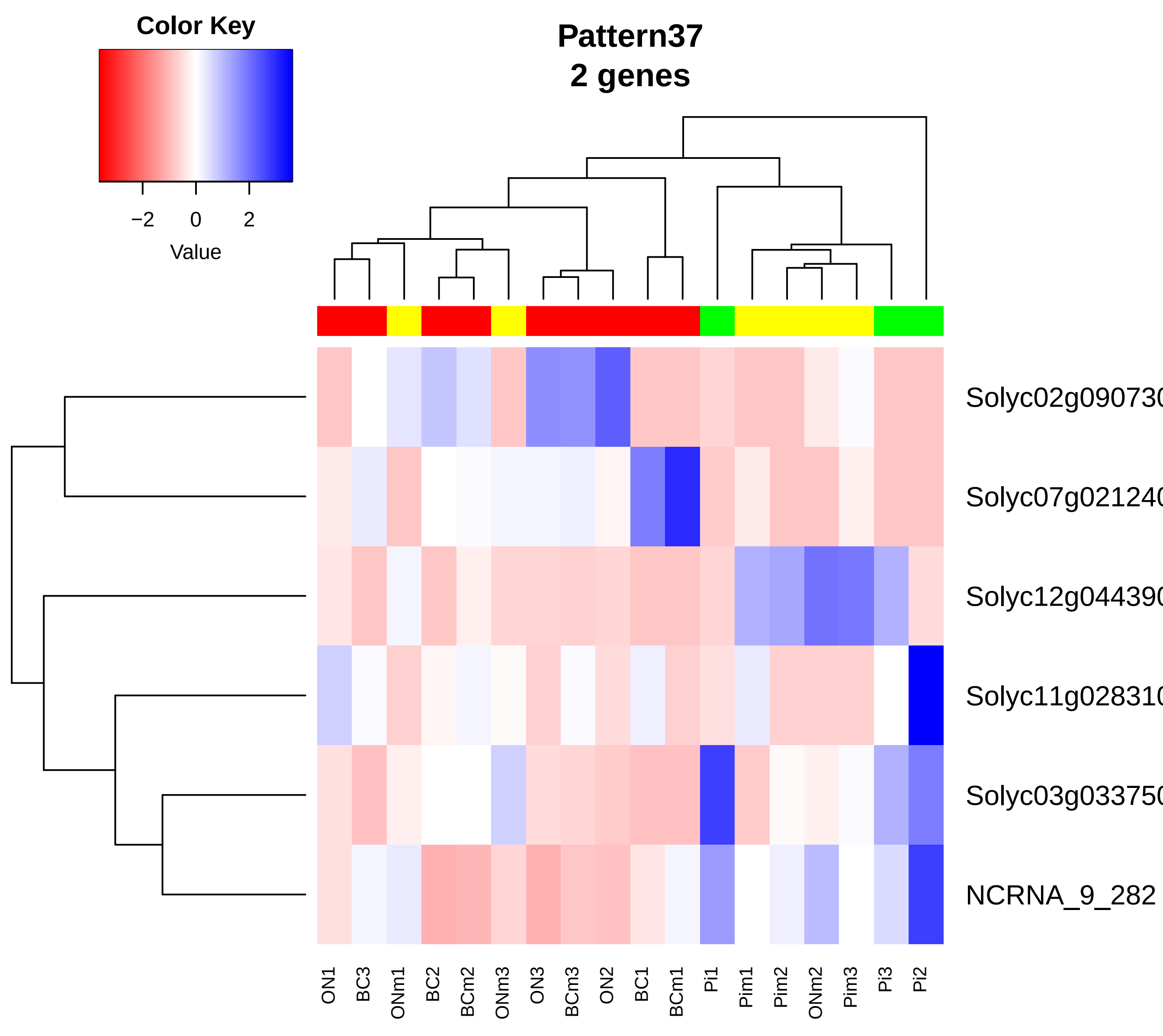

Color Key
−2
0
2
Value
Pattern37
2 genes
Solyc02g09073
Solyc07g02124
Solyc12g04439
Solyc11g02831
Solyc03g03375
NCRNA_9_282
ON1
BC3
ONm1
BC2
BCm2
ONm3
ON3
BCm3
ON2
BC1
BCm1
Pi1
Pim1
Pim2
ONm2
Pim3
Pi3
Pi2

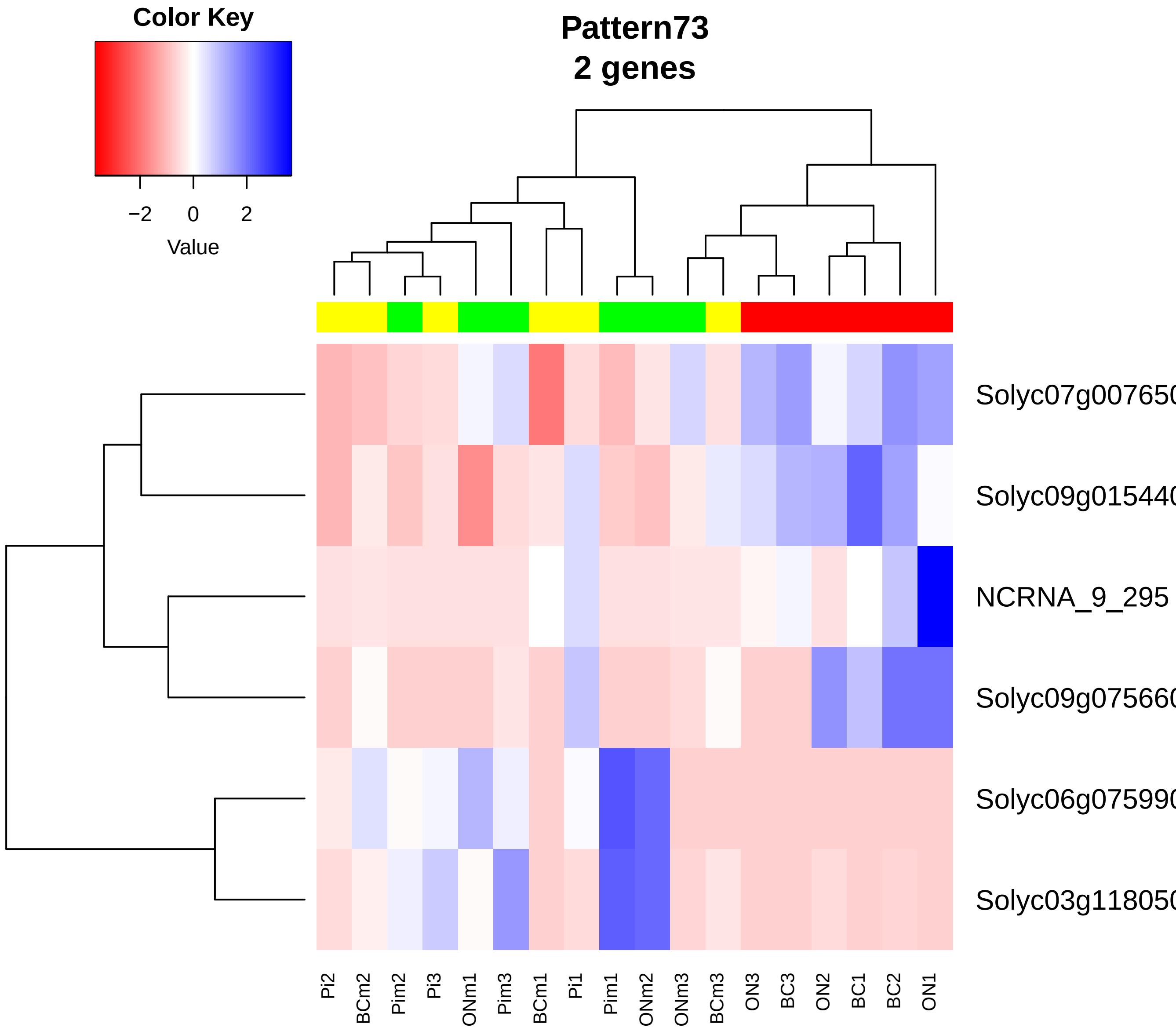
Color Key
−2
0
2
Value
Pattern73
2 genes
Solyc07g00765
Solyc09g01544
NCRNA_9_295
Solyc09g07566
Solyc06g07599
Solyc03g11805
Pi2
BCm2
Pim2
Pi3
ONm1
Pim3
BCm1
Pi1
Pim1
ONm2
ONm3
BCm3
ON3
BC3
ON2
BC1
BC2
ON1

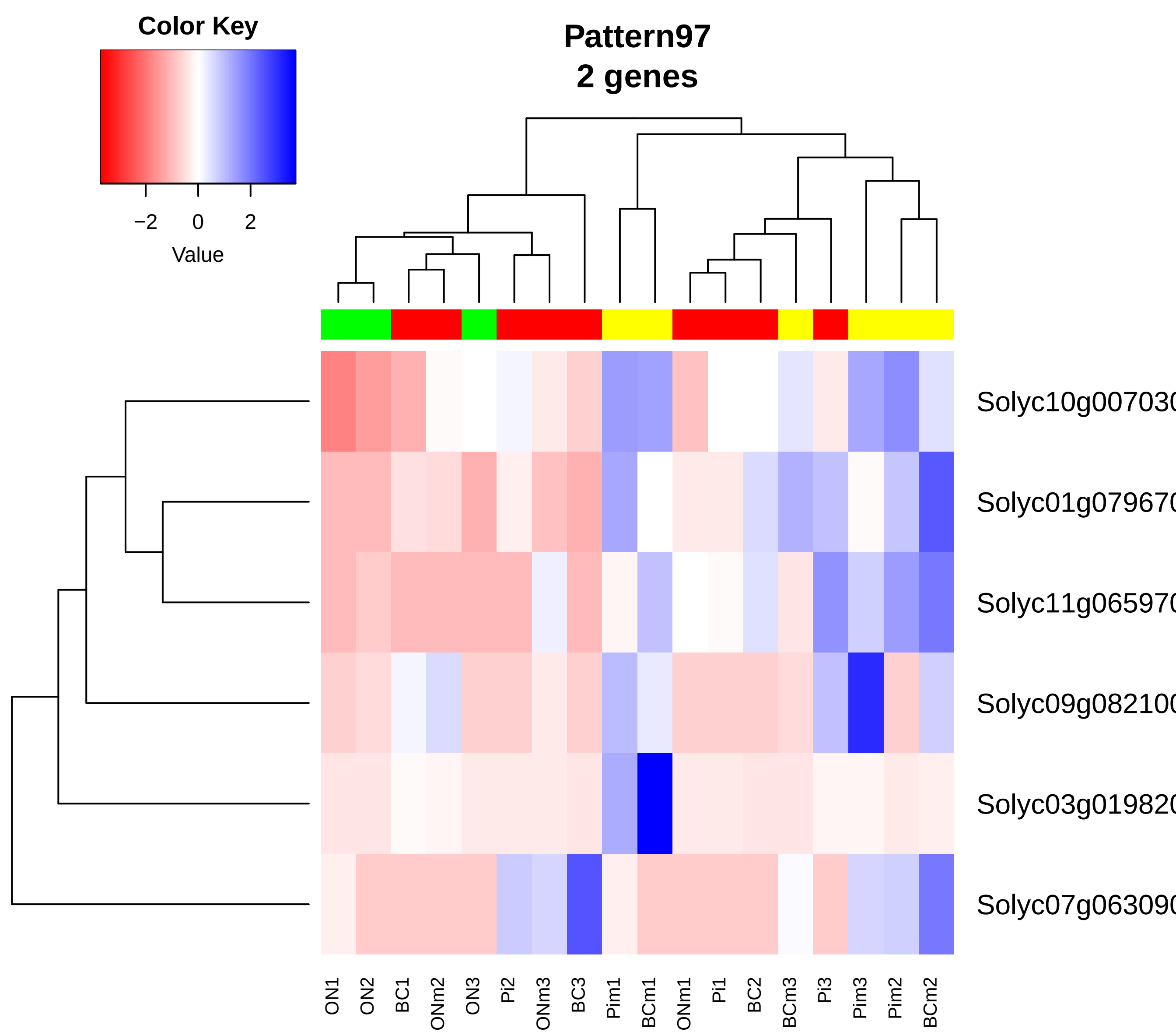
Color Key
−2
0
2
Value
Pattern97
2 genes
Solyc10g00703[illegible]
Solyc01g07967[illegible]
Solyc11g06597[illegible]
Solyc09g08210[illegible]
Solyc03g01982[illegible]
Solyc07g06309[illegible]
ON1
ON2
BC1
ONm2
ON3
Pi2
ONm3
BC3
Pim1
BCm1
ONm1
Pi1
BC2
BCm3
Pi3
Pim3
Pim2
BCm2

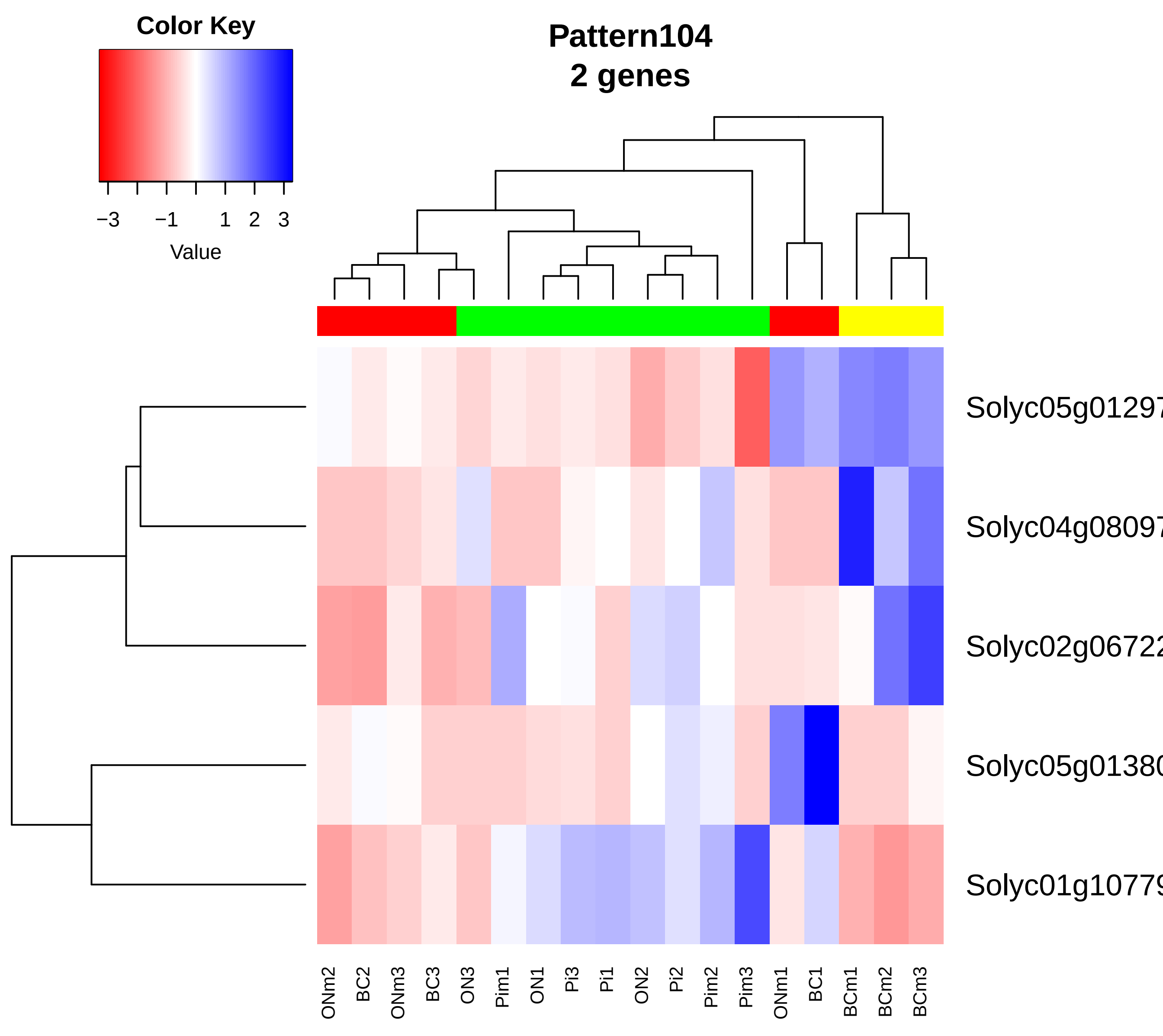

Color Key
−3
−1
1
2
3
Value
Pattern104
2 genes
Solyc05g01297
Solyc04g08097
Solyc02g06722
Solyc05g01380
Solyc01g10779
ONm2
BC2
ONm3
BC3
ON3
Pim1
ON1
Pi3
Pi1
ON2
Pi2
Pim2
Pim3
ONm1
BC1
BCm1
BCm2
BCm3

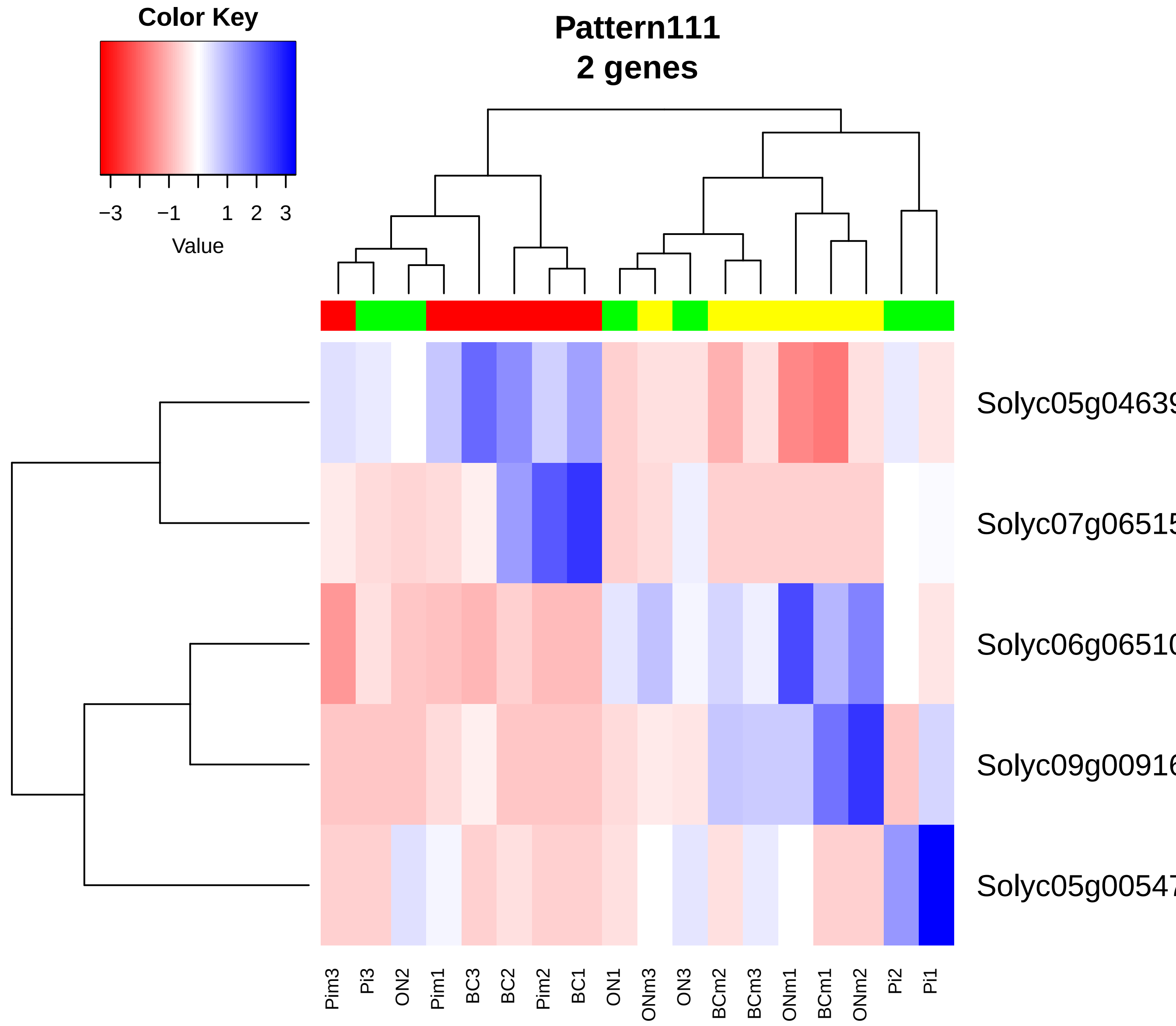
Color Key
−3
−1
1
2
3
Value
Pattern111
2 genes
Solyc05g04639
Solyc07g06515
Solyc06g06510
Solyc09g00916
Solyc05g00547
Pim3
Pi3
ON2
Pim1
BC3
BC2
Pim2
BC1
ON1
ONm3
ON3
BCm2
BCm3
ONm1
BCm1
ONm2
Pi2
Pi1

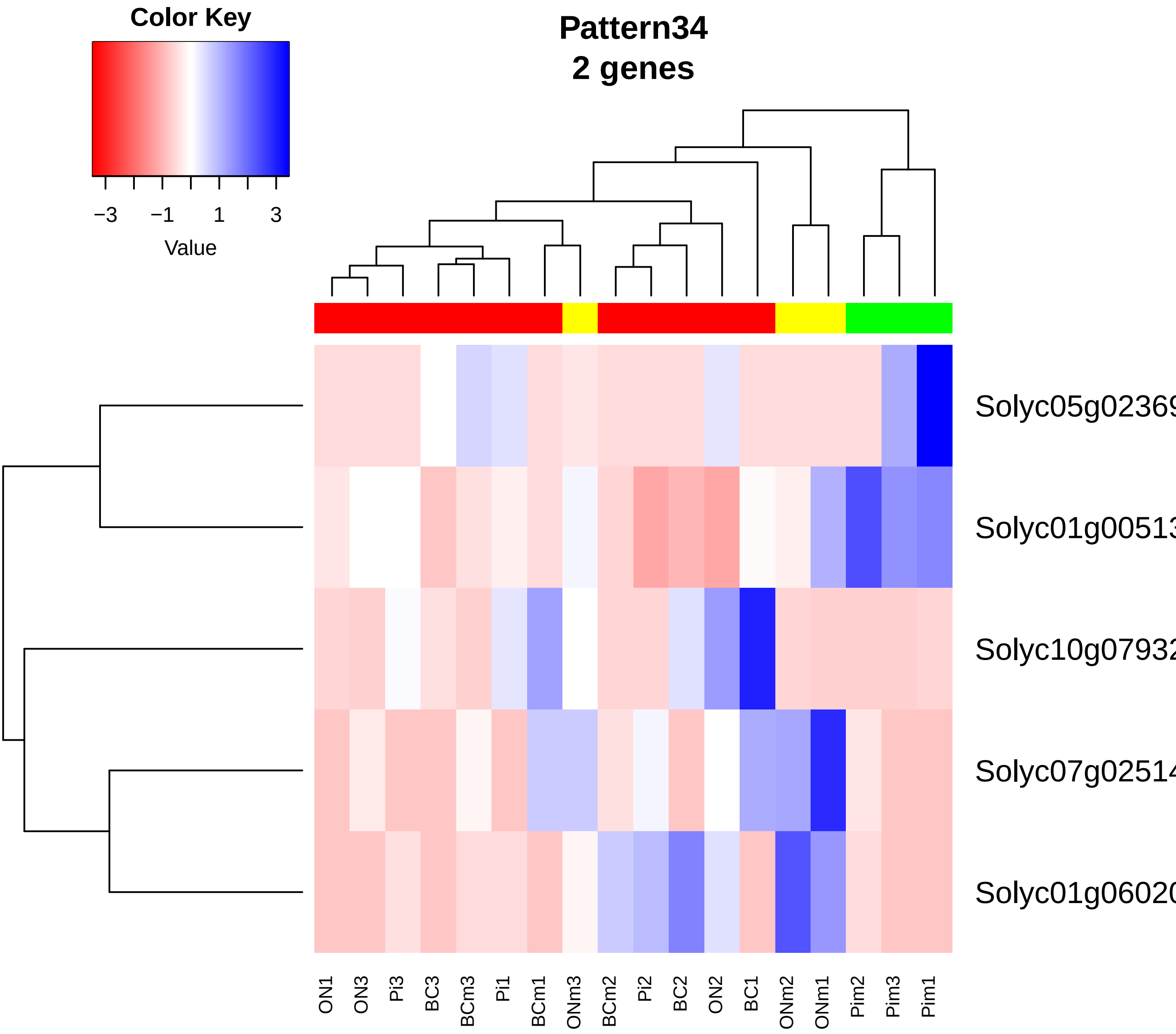
Color Key
−3
−1
1
3
Value
Pattern34
2 genes
Solyc05g02369
Solyc01g00513
Solyc10g07932
Solyc07g02514
Solyc01g06020
ON1
ON3
Pi3
BC3
BCm3
Pi1
BCm1
ONm3
BCm2
Pi2
BC2
ON2
BC1
ONm2
ONm1
Pim2
Pim3
Pim1

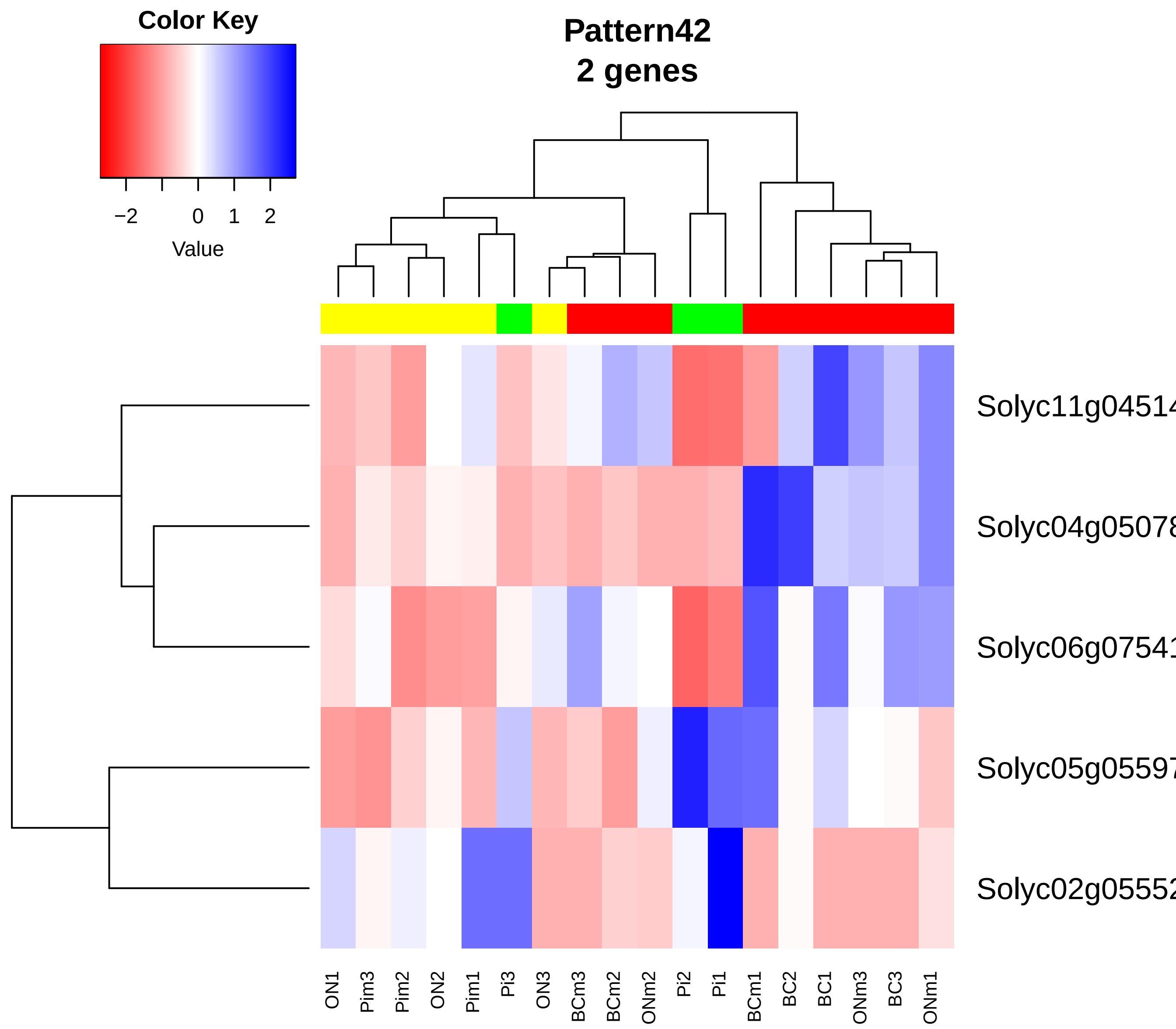

Color Key
−2
0
1
2
Value
Pattern42
2 genes
Solyc11g04514
Solyc04g05078
Solyc06g07541
Solyc05g05597
Solyc02g05552
ON1
Pim3
Pim2
ON2
Pim1
Pi3
ON3
BCm3
BCm2
ONm2
Pi2
Pi1
BCm1
BC2
BC1
ONm3
BC3
ONm1

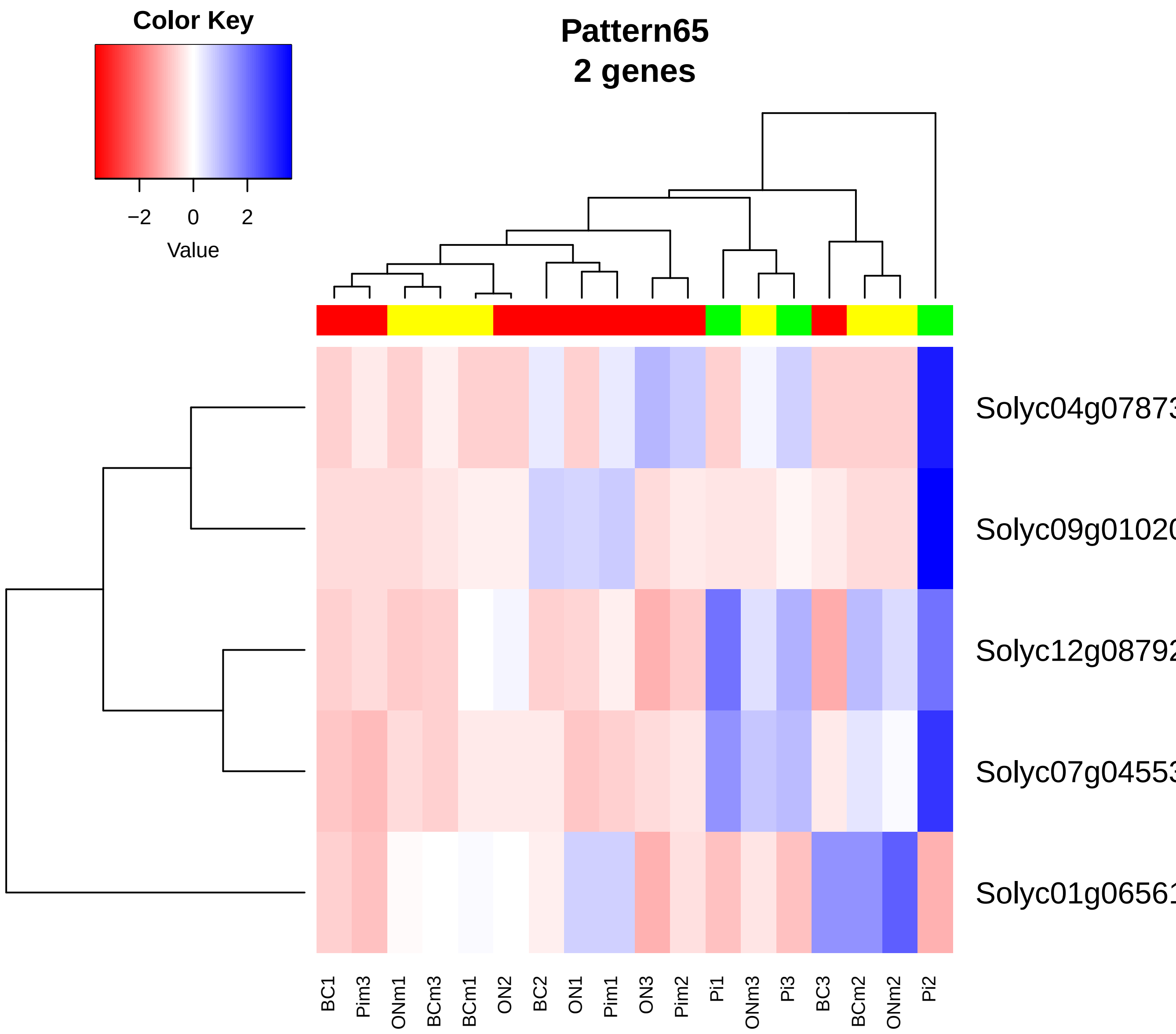
Color Key
−2
0
2
Value
Pattern65
2 genes
Solyc04g07873
Solyc09g01020
Solyc12g08792
Solyc07g04553
Solyc01g06561
BC1
Pim3
ONm1
BCm3
BCm1
ON2
BC2
ON1
Pim1
ON3
Pim2
Pi1
ONm3
Pi3
BC3
BCm2
ONm2
Pi2

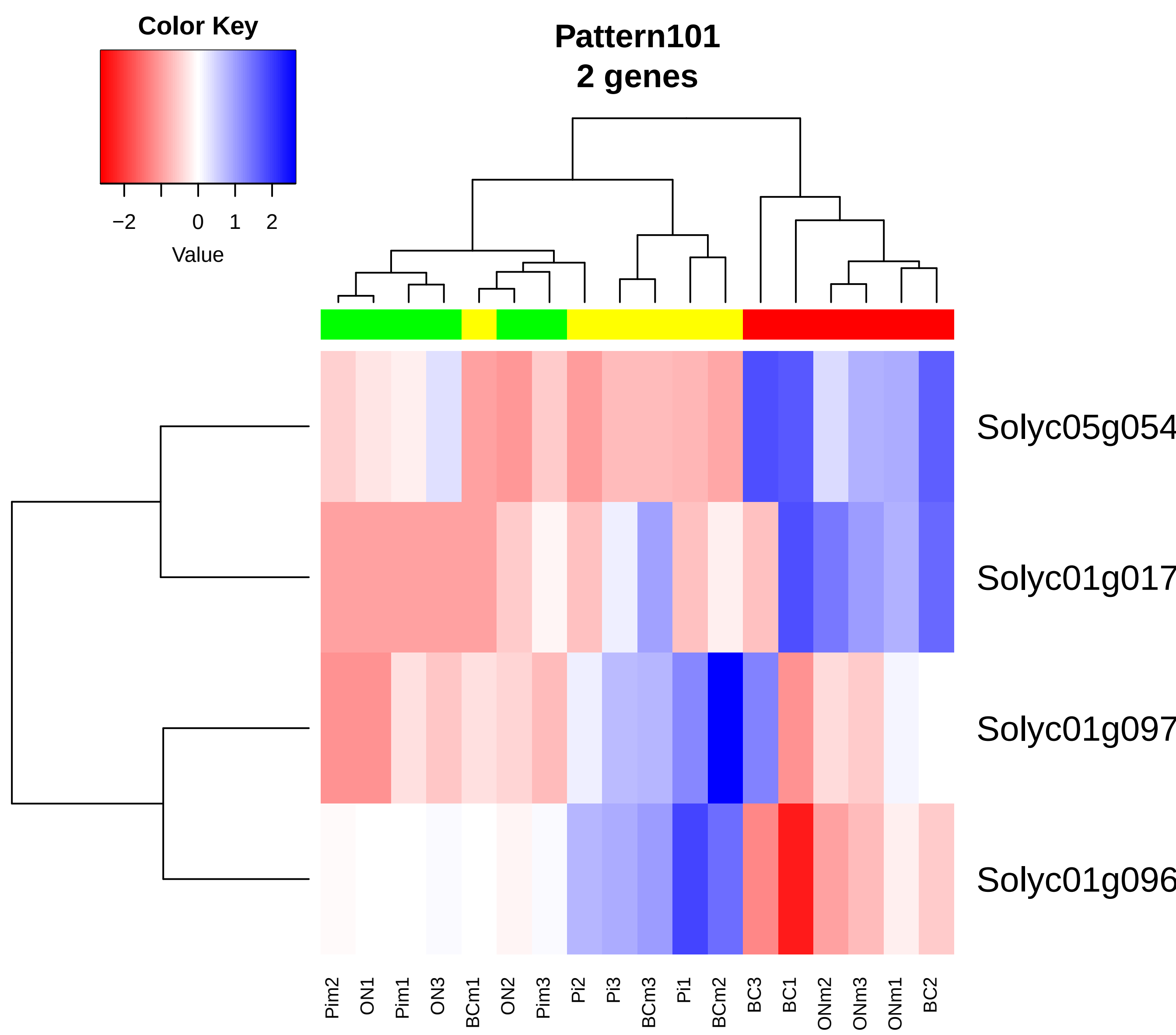

Color Key
−2
0
1
2
Value
Pattern101
2 genes
Solyc05g054
Solyc01g017
Solyc01g097
Solyc01g096
Pim2
ON1
Pim1
ON3
BCm1
ON2
Pim3
Pi2
Pi3
BCm3
Pi1
BCm2
BC3
BC1
ONm2
ONm3
ONm1
BC2

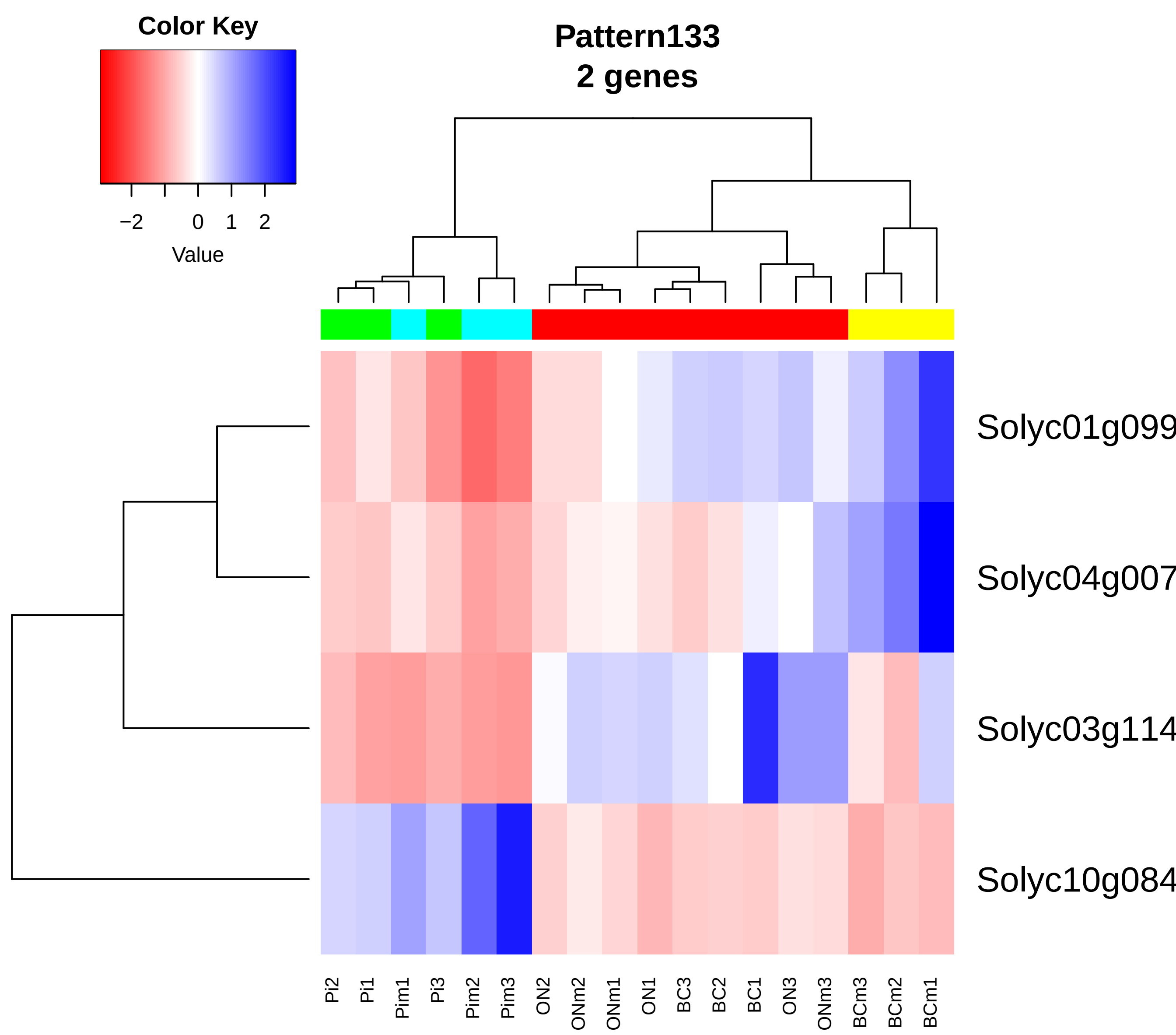
Color Key
−2
0
1
2
Value
Pattern133
2 genes
Solyc01g099
Solyc04g007
Solyc03g114
Solyc10g084
Pi2
Pi1
Pim1
Pi3
Pim2
Pim3
ON2
ONm2
ONm1
ON1
BC3
BC2
BC1
ON3
ONm3
BCm3
BCm2
BCm1

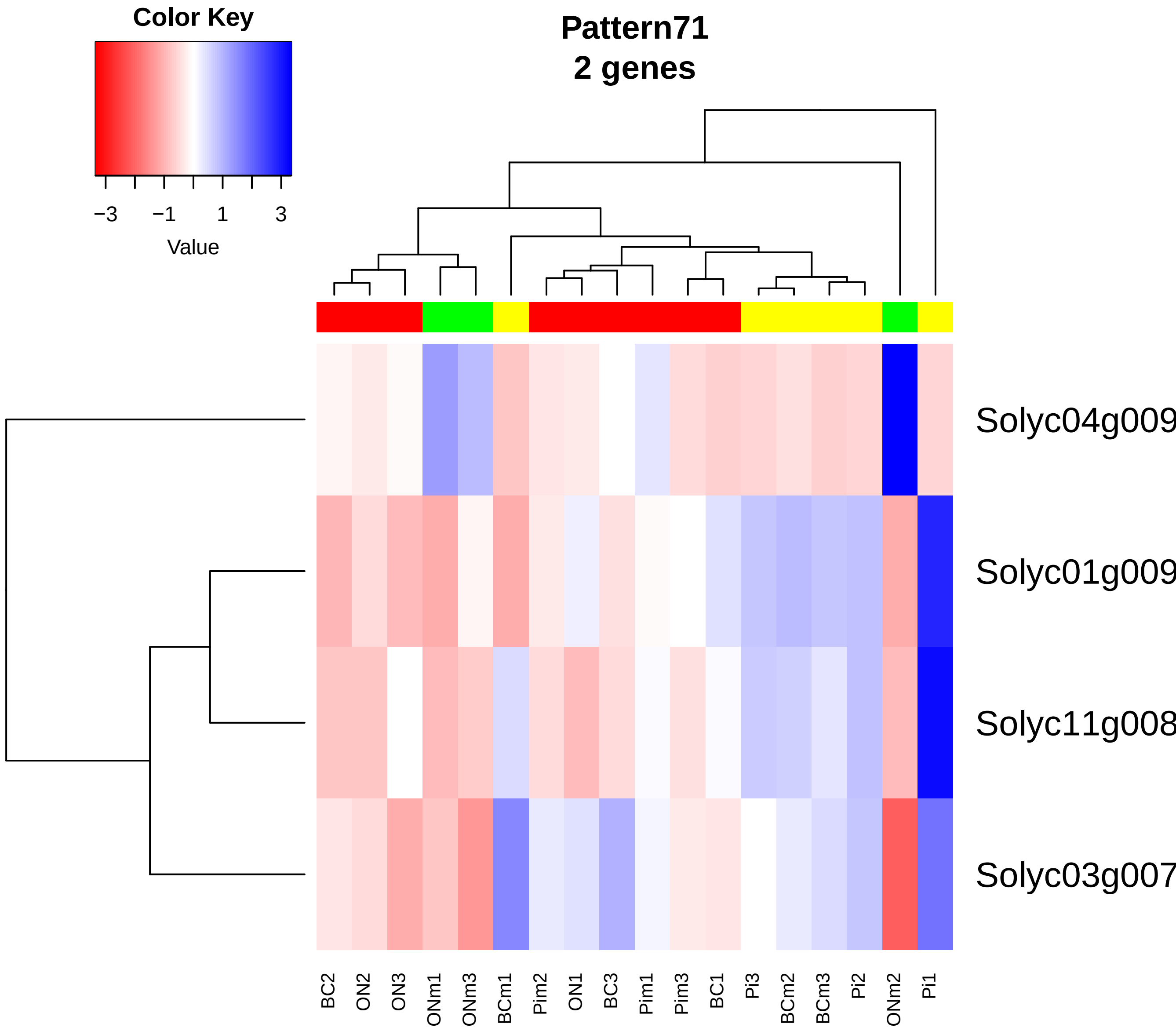
Color Key
−3
−1
1
3
Value
Pattern71
2 genes
Solyc04g009
Solyc01g009
Solyc11g008
Solyc03g007
BC2
ON2
ON3
ONm1
ONm3
BCm1
Pim2
ON1
BC3
Pim1
Pim3
BC1
Pi3
BCm2
BCm3
Pi2
ONm2
Pi1

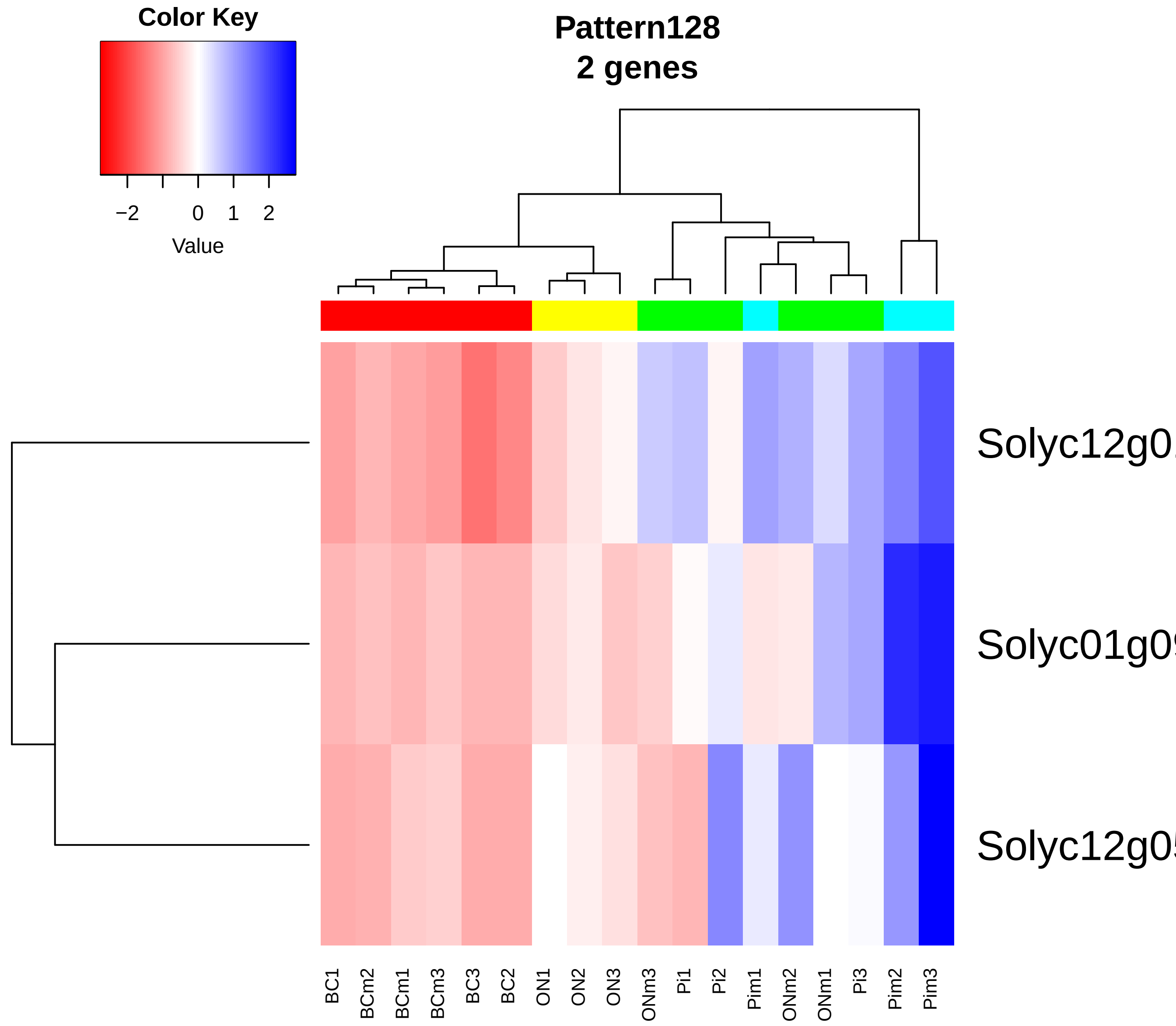
Color Key
−2
0
1
2
Value
Pattern128
2 genes
Solyc12g0
Solyc01g0
Solyc12g0
BC1
BCm2
BCm1
BCm3
BC3
BC2
ON1
ON2
ON3
ONm3
Pi1
Pi2
Pim1
ONm2
ONm1
Pi3
Pim2
Pim3

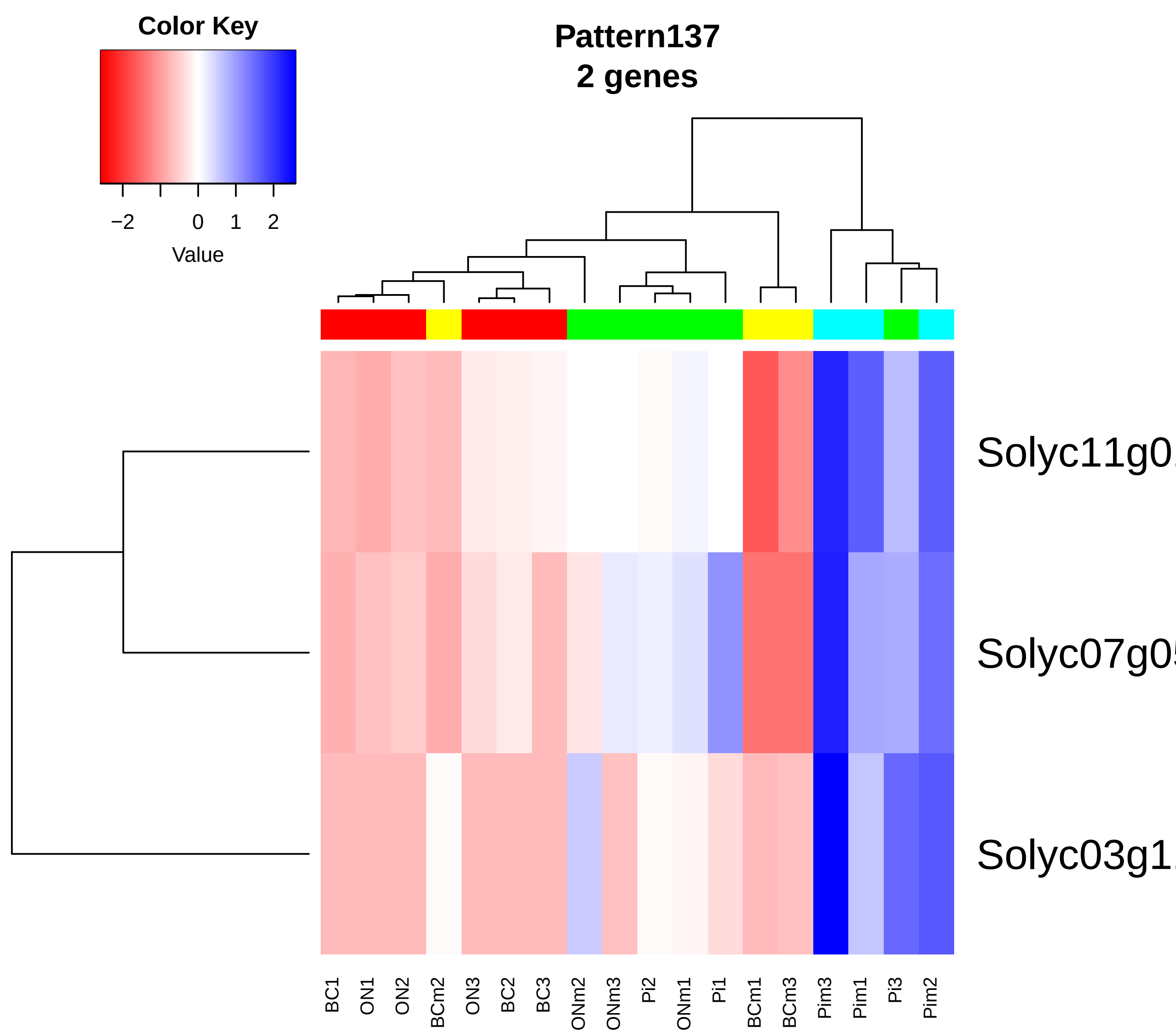

Color Key
−2
0
1
2
Value
Pattern137
2 genes
Solyc11g0
Solyc07g0
Solyc03g1
BC1
ON1
ON2
BCm2
ON3
BC2
BC3
ONm2
ONm3
Pi2
ONm1
Pi1
BCm1
BCm3
Pim3
Pim1
Pi3
Pim2

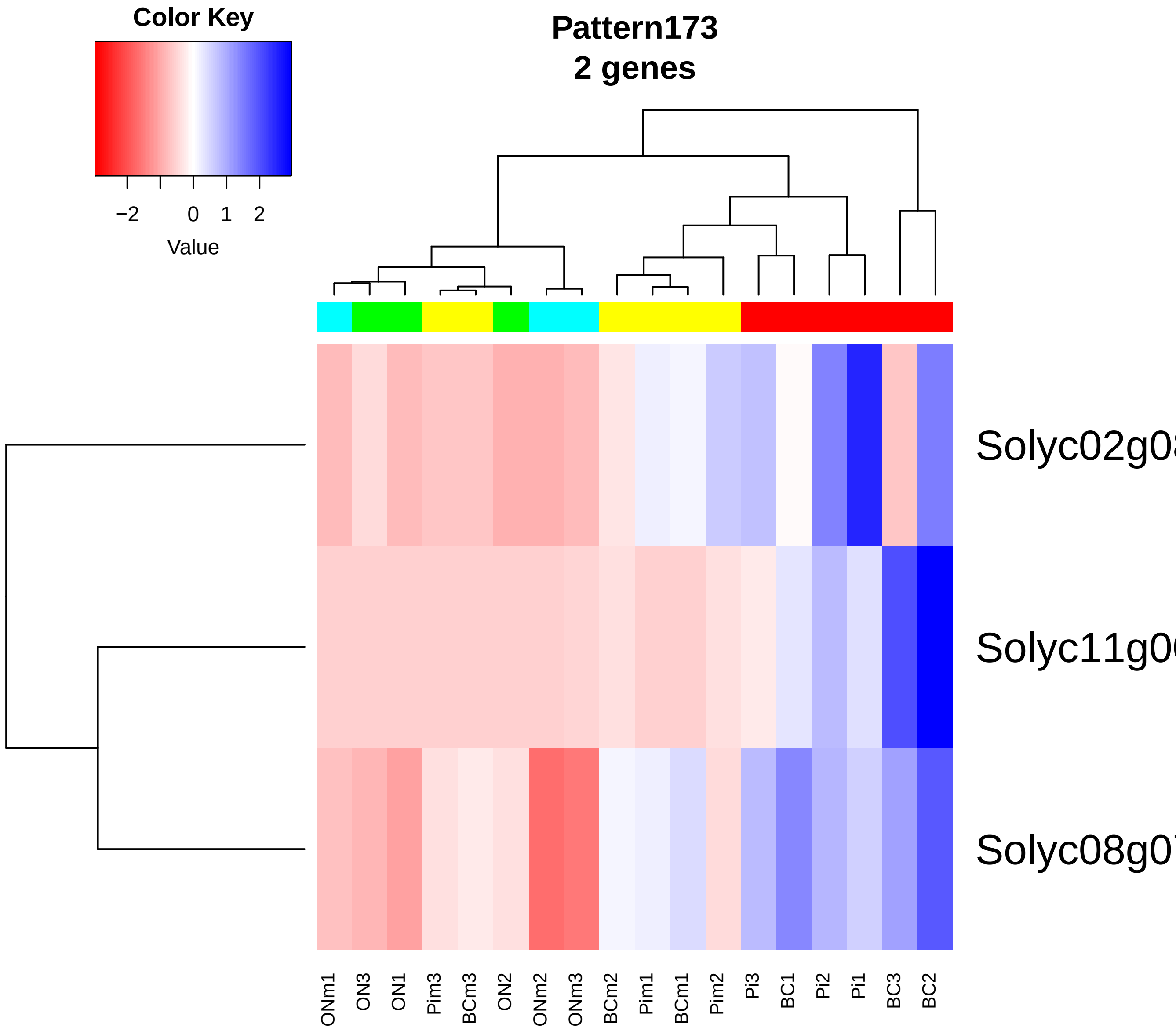
Color Key
−2
0
1
2
Value
Pattern173
2 genes
Solyc02g0
Solyc11g0
Solyc08g0
ONm1
ON3
ON1
Pim3
BCm3
ON2
ONm2
ONm3
BCm2
Pim1
BCm1
Pim2
Pi3
BC1
Pi2
Pi1
BC3
BC2

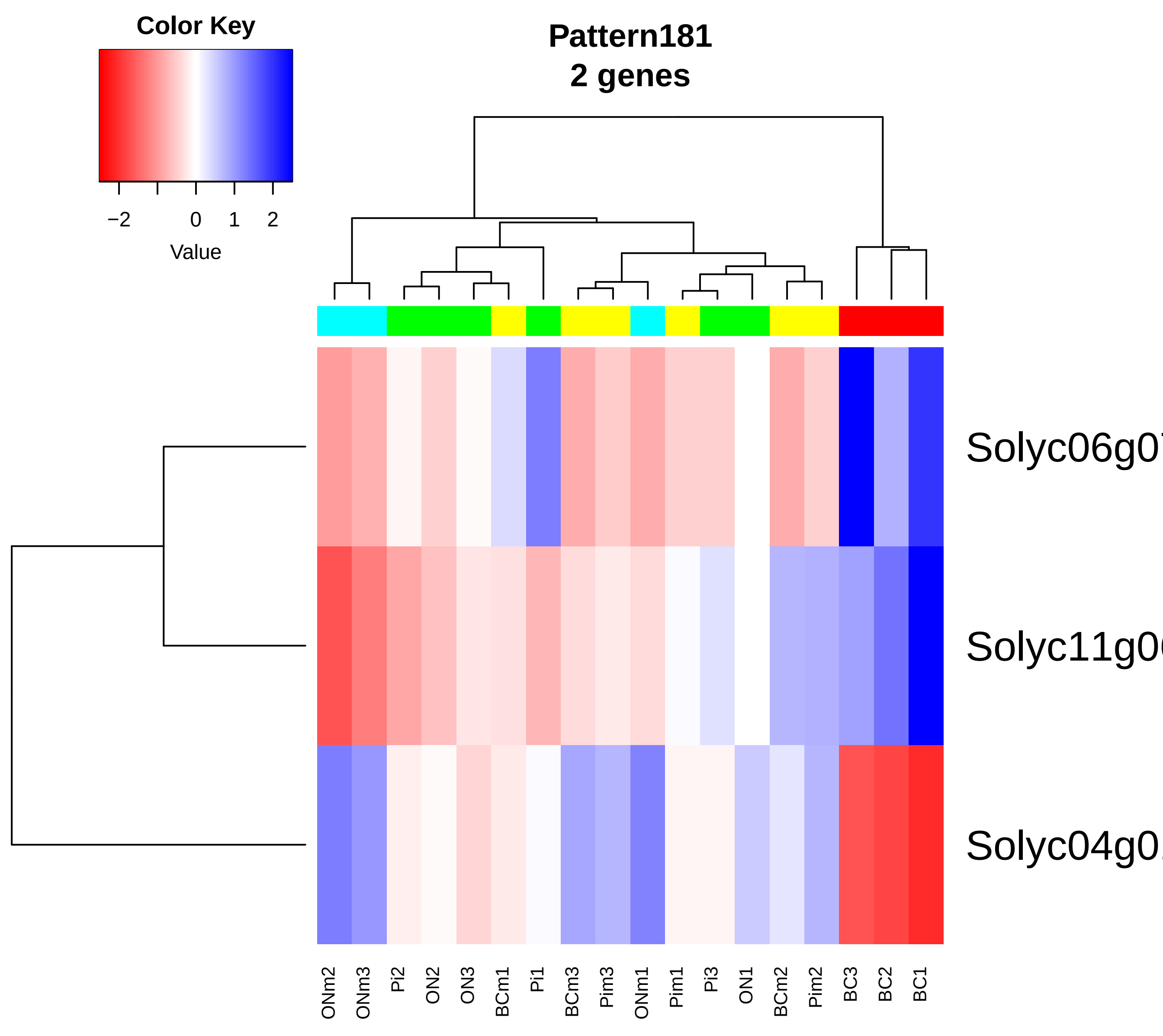
Color Key
−2
0
1
2
Value
Pattern181
2 genes
Solyc06g07
Solyc11g06
Solyc04g0
ONm2
ONm3
Pi2
ON2
ON3
BCm1
Pi1
BCm3
Pim3
ONm1
Pim1
Pi3
ON1
BCm2
Pim2
BC3
BC2
BC1

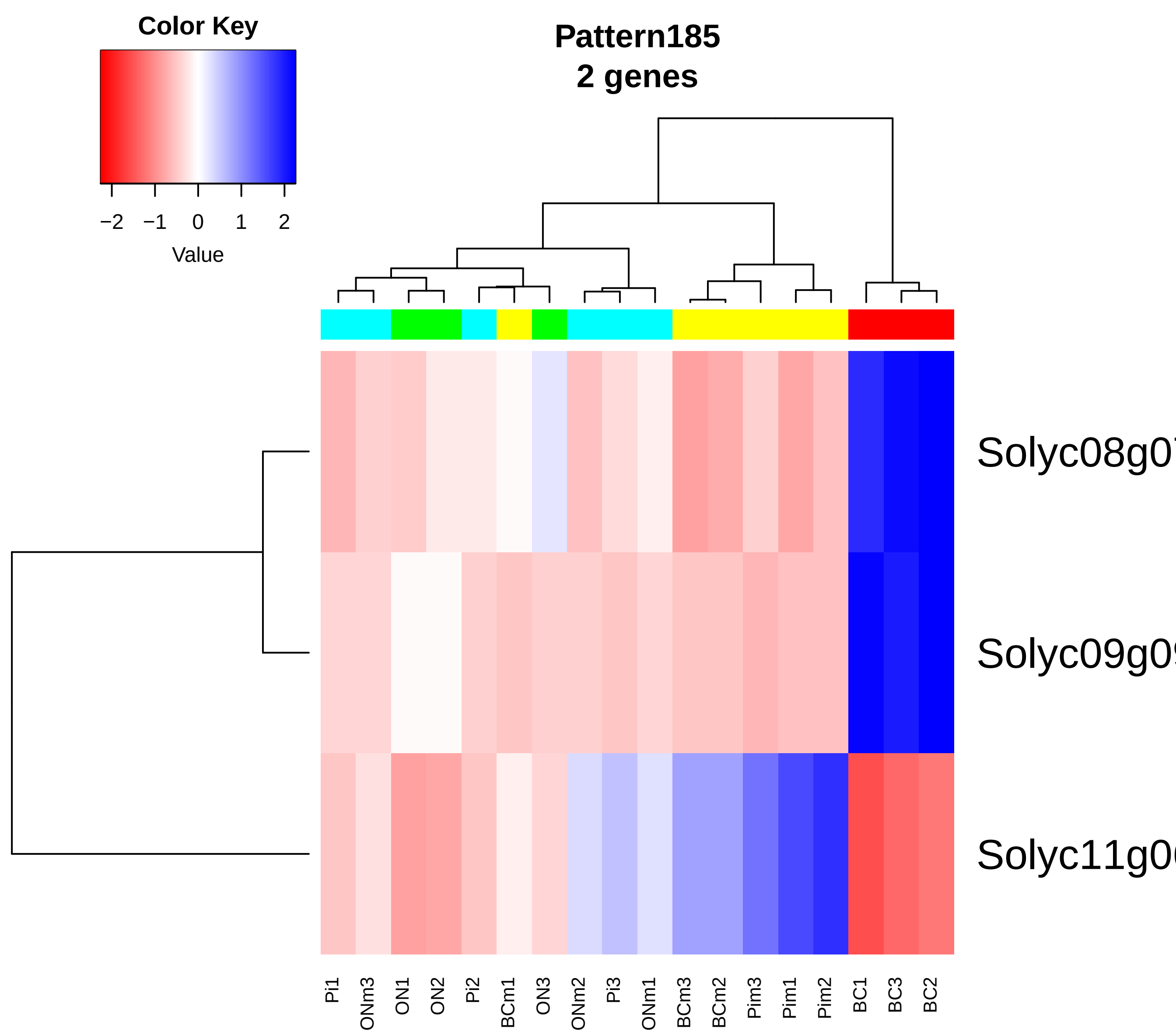

Color Key
−2 −1 0 1 2
Value
Pattern185
2 genes
Solyc08g07
Solyc09g09
Solyc11g06
Pi1
ONm3
ON1
ON2
Pi2
BCm1
ON3
ONm2
Pi3
ONm1
BCm3
BCm2
Pim3
Pim1
Pim2
BC1
BC3
BC2

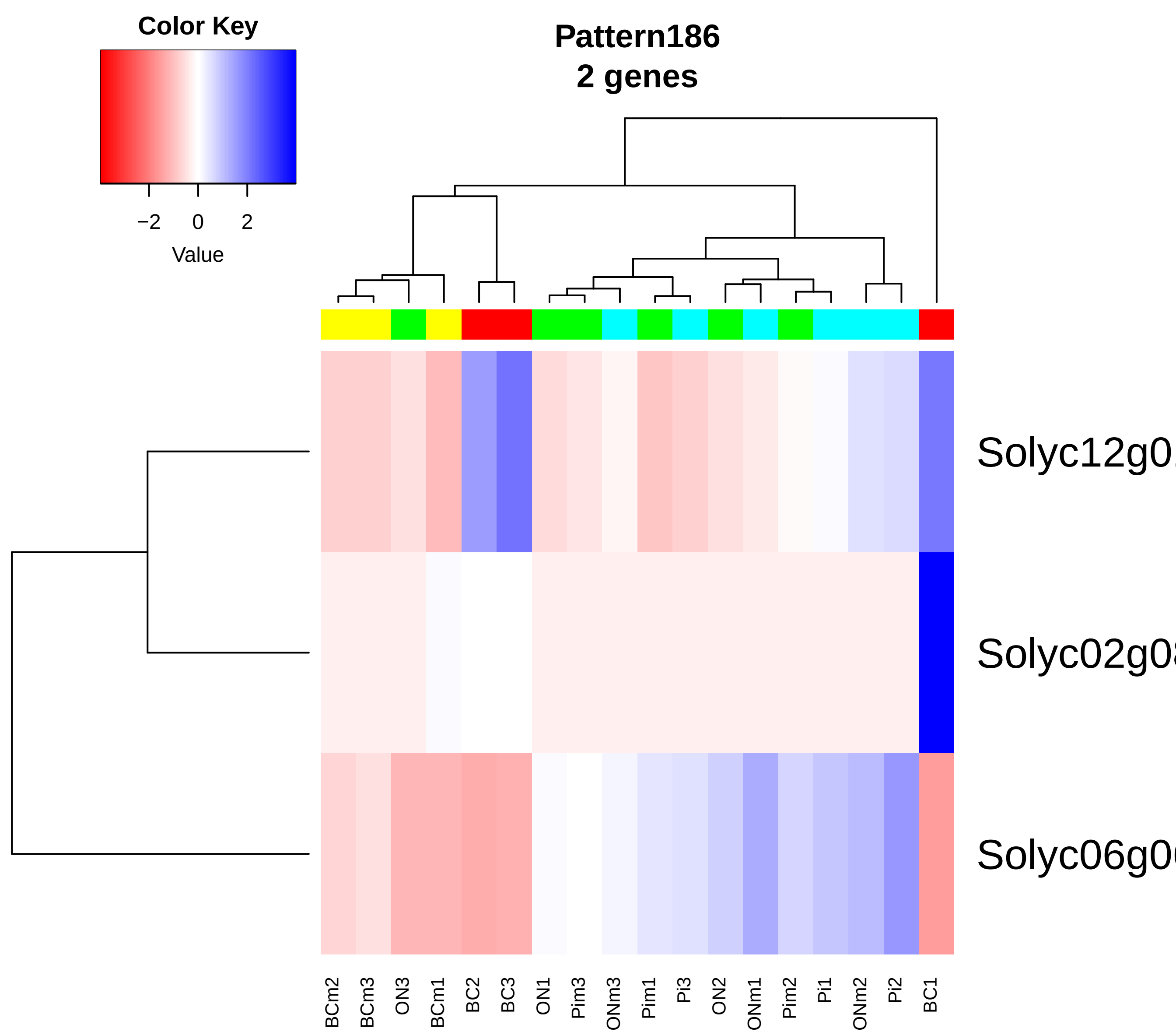

Color Key
−2
0
2
Value
Pattern186
2 genes
Solyc12g0
Solyc02g0
Solyc06g0
BCm2
BCm3
ON3
BCm1
BC2
BC3
ON1
Pim3
ONm3
Pim1
Pi3
ON2
ONm1
Pim2
Pi1
ONm2
Pi2
BC1

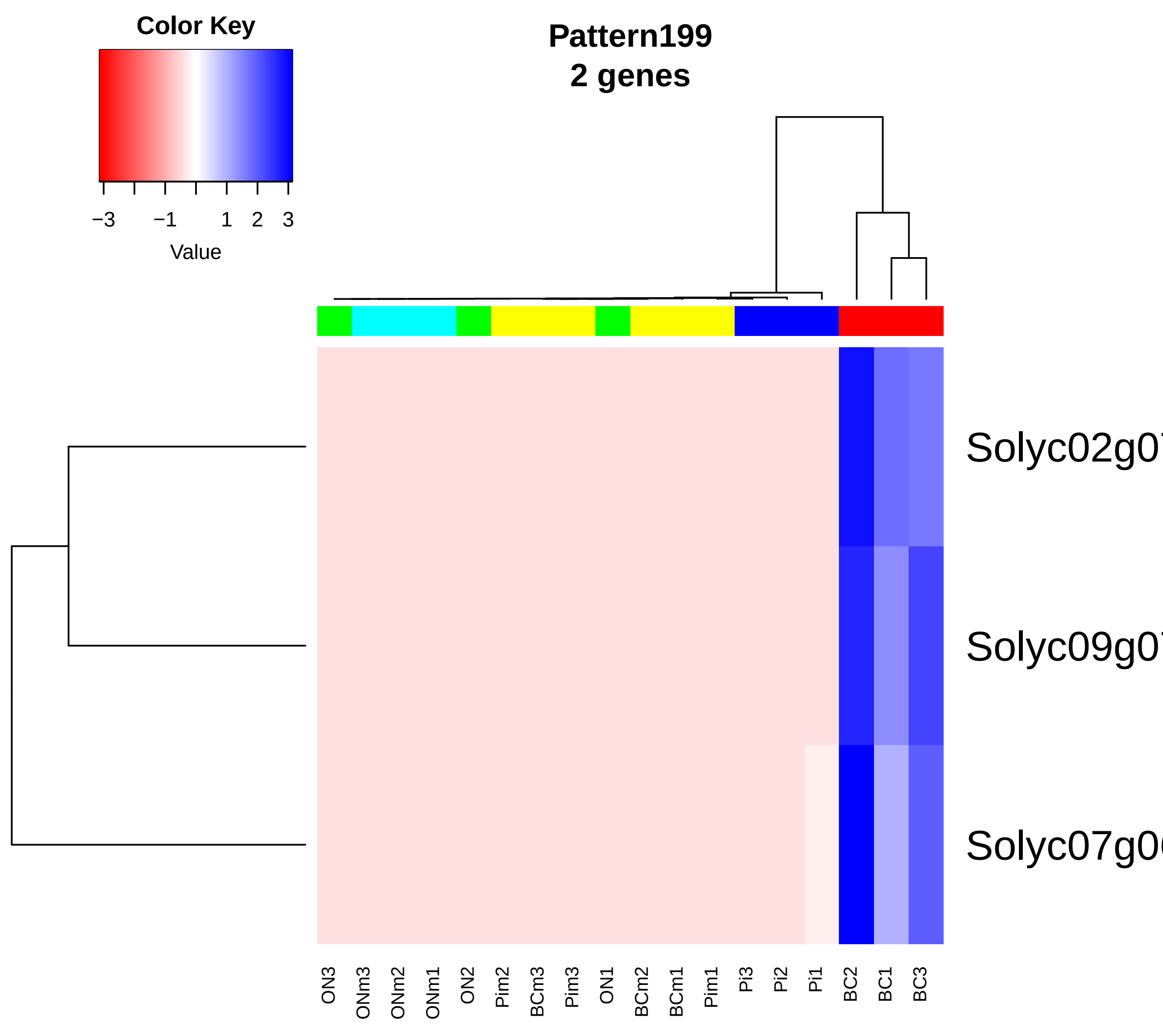
Color Key
−3
−1
1
2
3
Value
Pattern199
2 genes
Solyc02g07
Solyc09g07
Solyc07g0
ON3
ONm3
ONm2
ONm1
ON2
Pim2
BCm3
Pim3
ON1
BCm2
BCm1
Pim1
Pi3
Pi2
Pi1
BC2
BC1
BC3

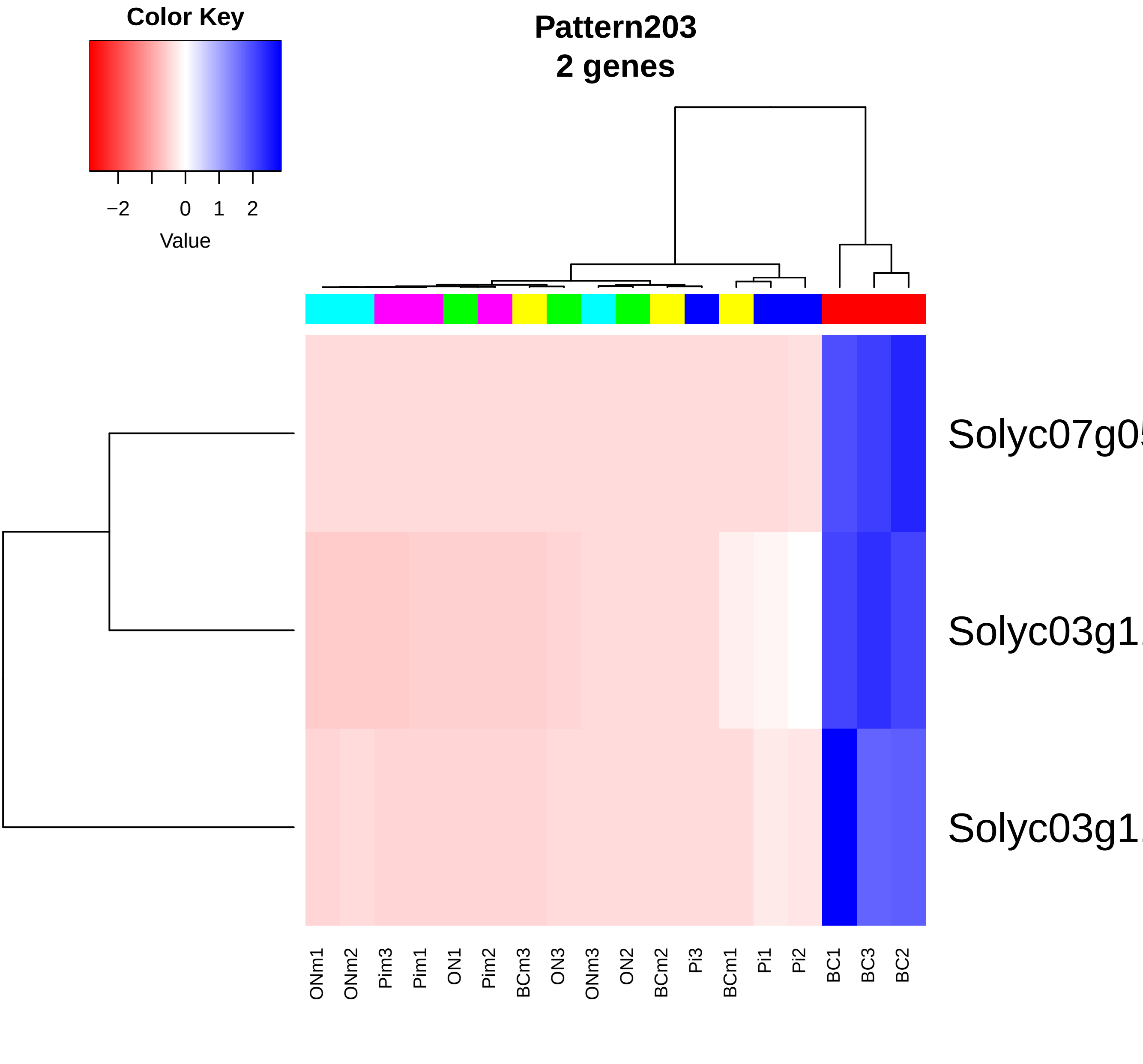

Color Key
−2
0
1
2
Value
Pattern203
2 genes
Solyc07g05
Solyc03g1
Solyc03g1
ONm1
ONm2
Pim3
Pim1
ON1
Pim2
BCm3
ON3
ONm3
ON2
BCm2
Pi3
BCm1
Pi1
Pi2
BC1
BC3
BC2

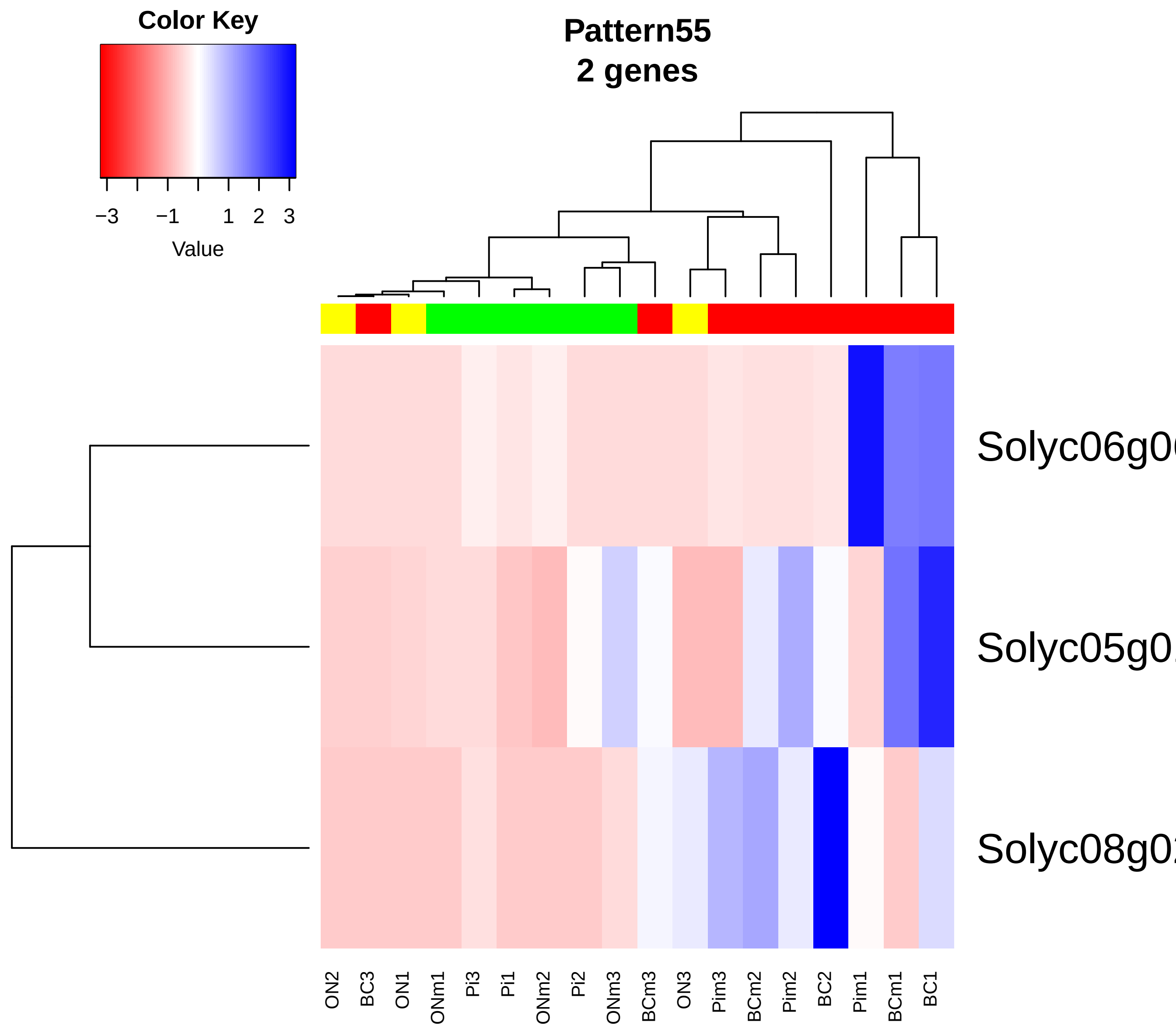

Color Key
−3
−1
1
2
3
Value
Pattern55
2 genes
Solyc06g0
Solyc05g0
Solyc08g0
ON2
BC3
ON1
ONm1
Pi3
Pi1
ONm2
Pi2
ONm3
BCm3
ON3
Pim3
BCm2
Pim2
BC2
Pim1
BCm1
BC1

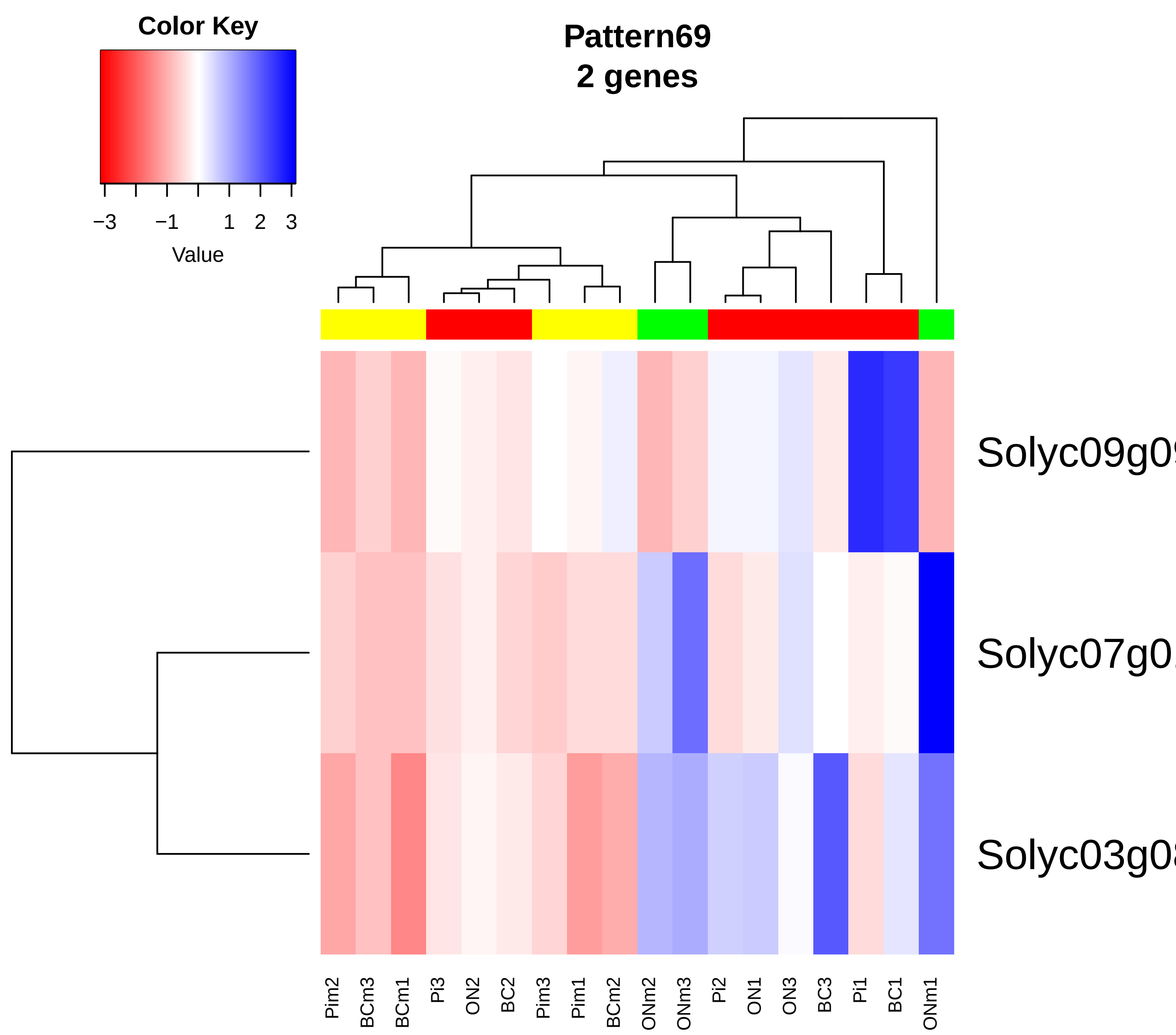
Color Key
−3
−1
1
2
3
Value
Pattern69
2 genes
Solyc09g09
Solyc07g0
Solyc03g0
Pim2
BCm3
BCm1
Pi3
ON2
BC2
Pim3
Pim1
BCm2
ONm2
ONm3
Pi2
ON1
ON3
BC3
Pi1
BC1
ONm1

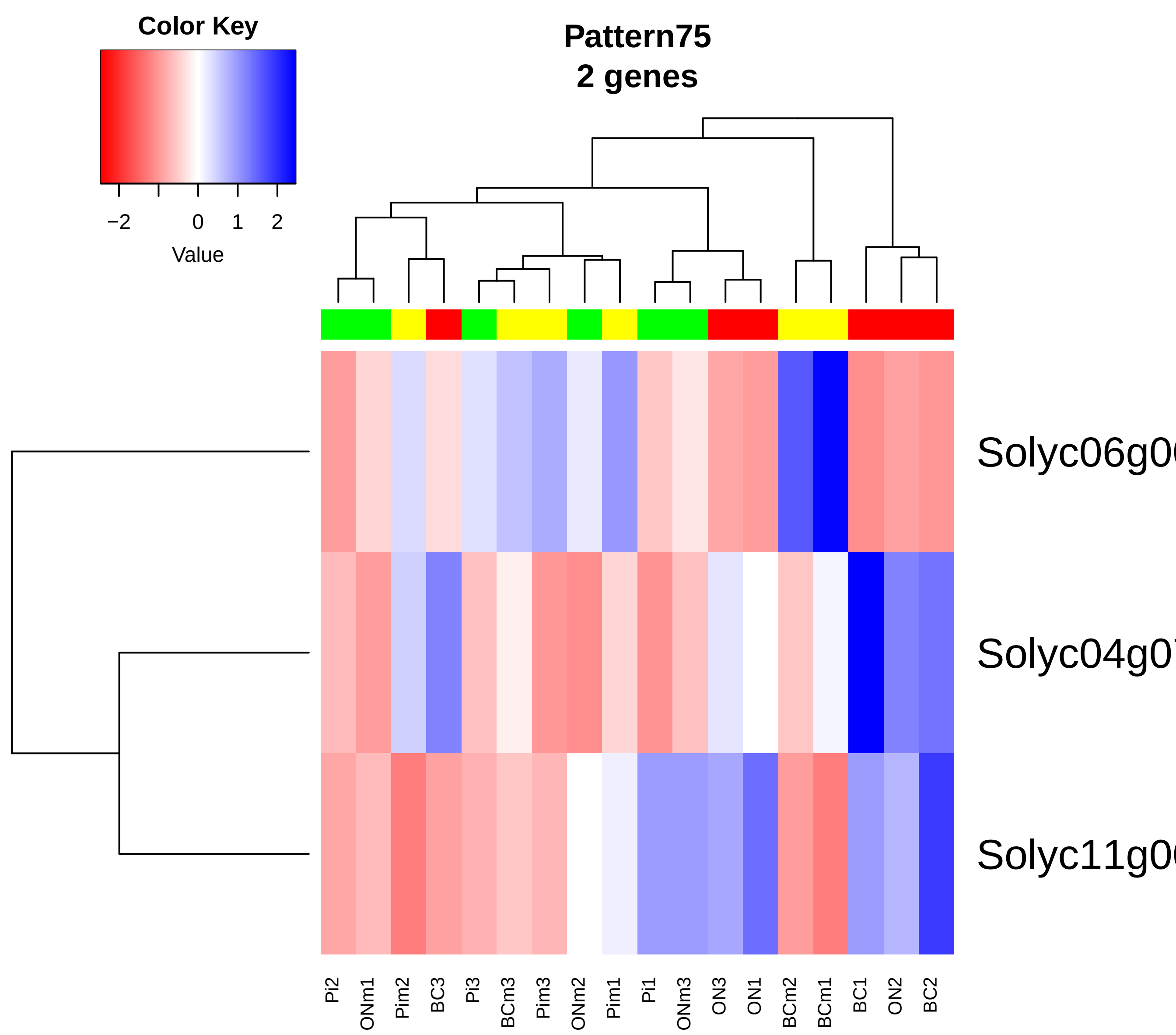
Color Key
−2
0
1
2
Value
Pattern75
2 genes
Solyc06g0
Solyc04g0
Solyc11g0
Pi2
ONm1
Pim2
BC3
Pi3
BCm3
Pim3
ONm2
Pim1
Pi1
ONm3
ON3
ON1
BCm2
BCm1
BC1
ON2
BC2

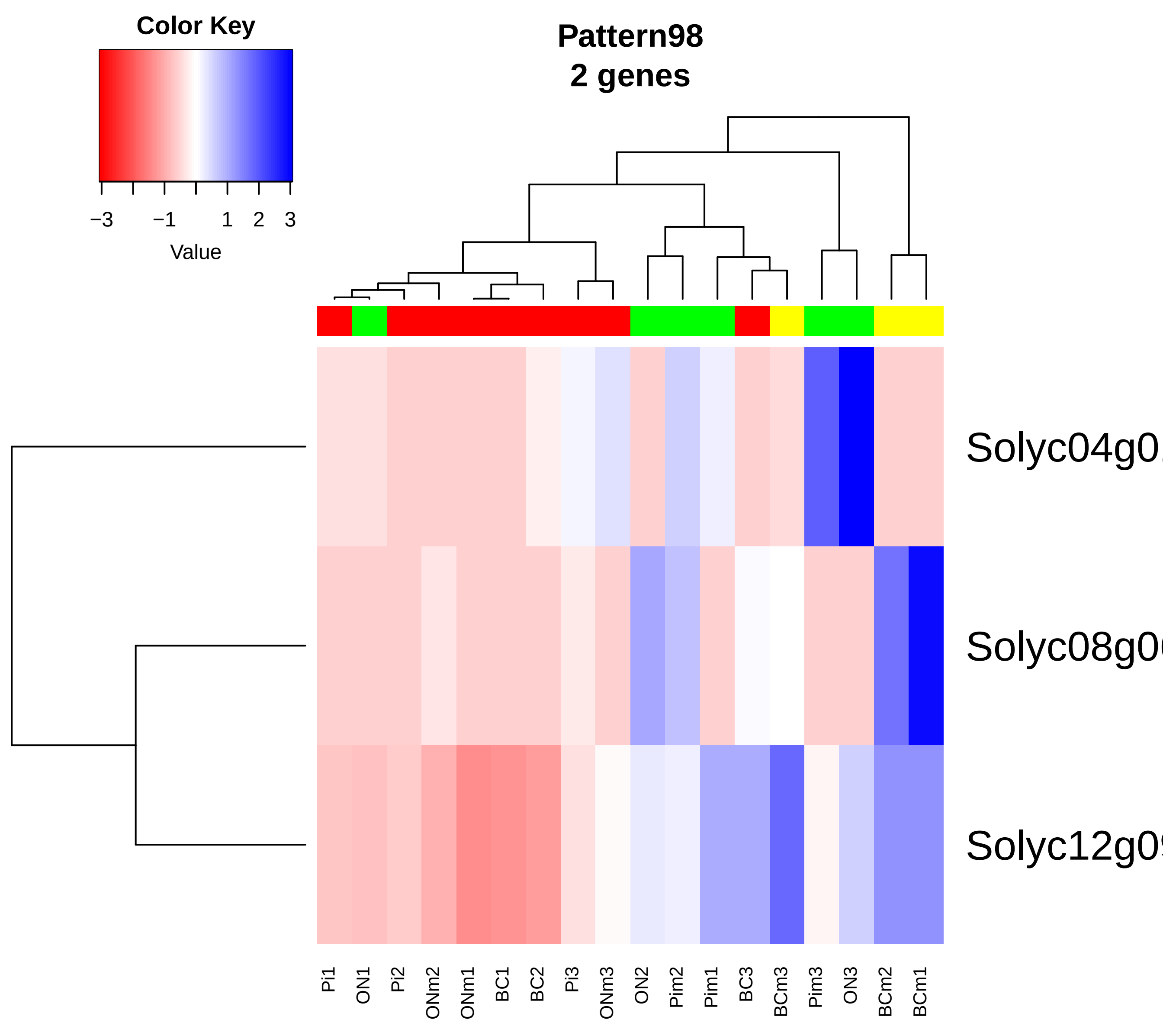

Color Key
−3
−1
1
2
3
Value
Pattern98
2 genes
Solyc04g0
Solyc08g0
Solyc12g0
Pi1
ON1
Pi2
ONm2
ONm1
BC1
BC2
Pi3
ONm3
ON2
Pim2
Pim1
BC3
BCm3
Pim3
ON3
BCm2
BCm1

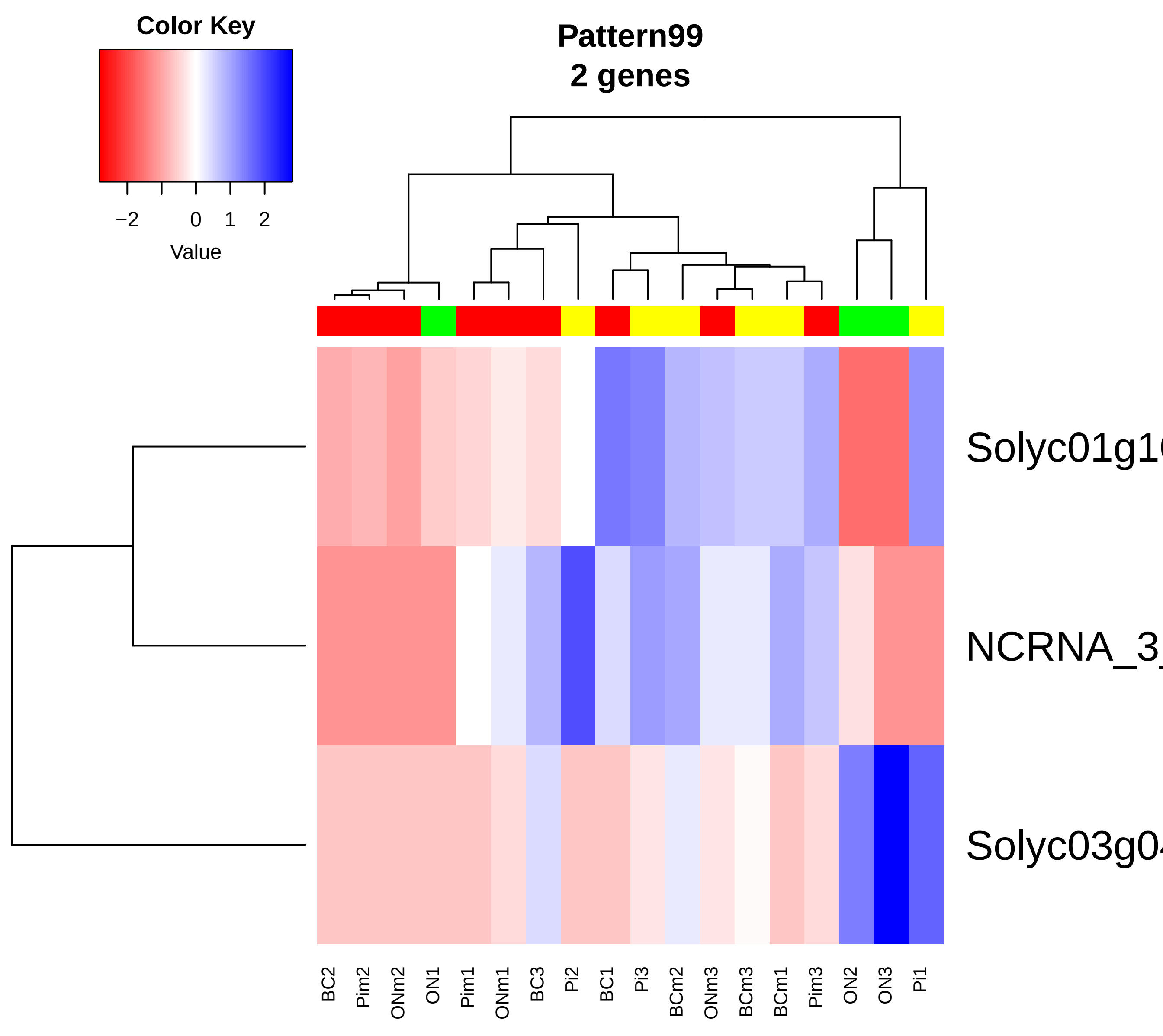
Color Key
−2
0
1
2
Value
Pattern99
2 genes
Solyc01g10
NCRNA_3_
Solyc03g0
BC2
Pim2
ONm2
ON1
Pim1
ONm1
BC3
Pi2
BC1
Pi3
BCm2
ONm3
BCm3
BCm1
Pim3
ON2
ON3
Pi1

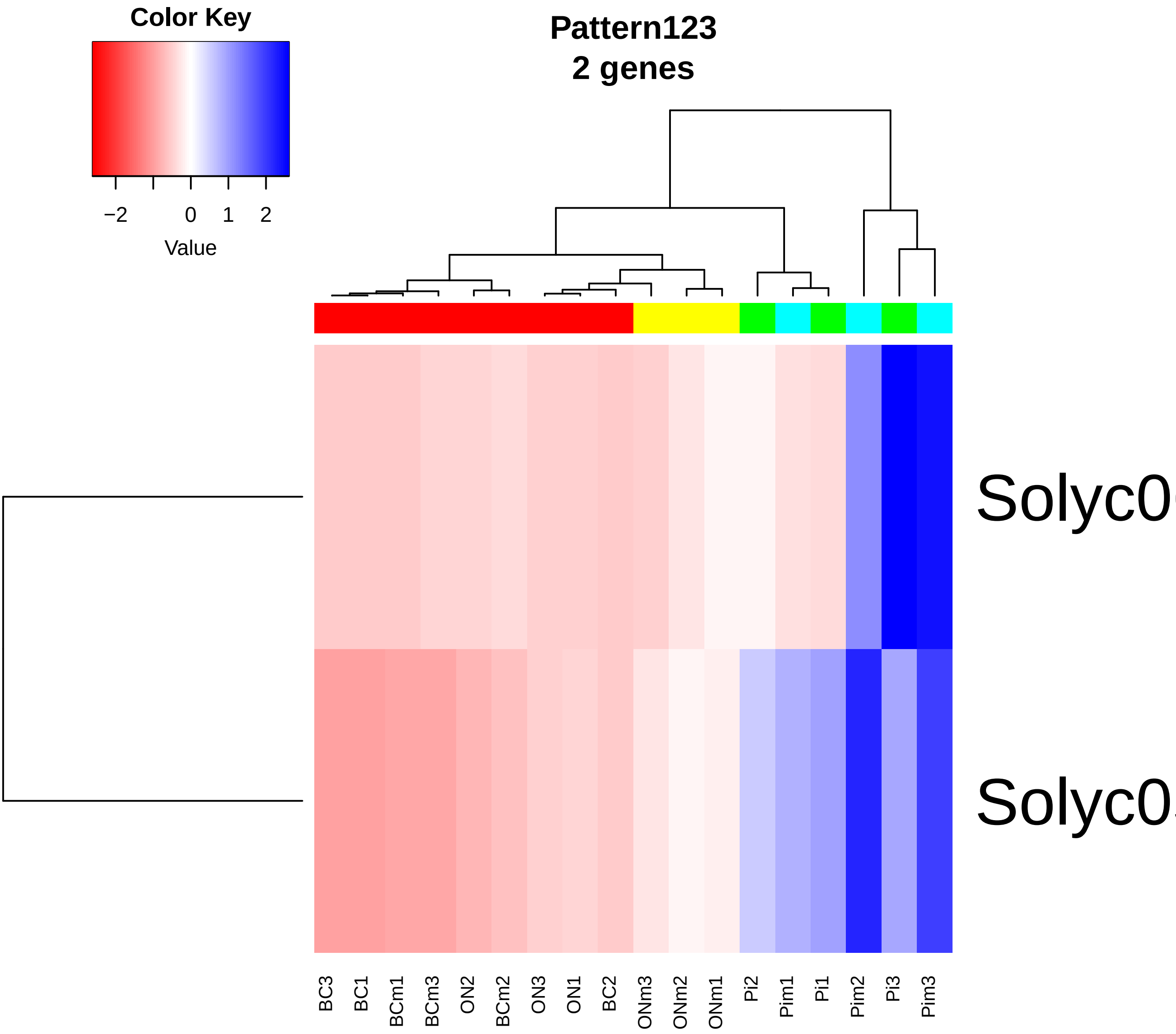
Color Key
−2
0
1
2
Value
Pattern123
2 genes
Solyc0
Solyc0
BC3
BC1
BCm1
BCm3
ON2
BCm2
ON3
ON1
BC2
ONm3
ONm2
ONm1
Pi2
Pim1
Pi1
Pim2
Pi3
Pim3

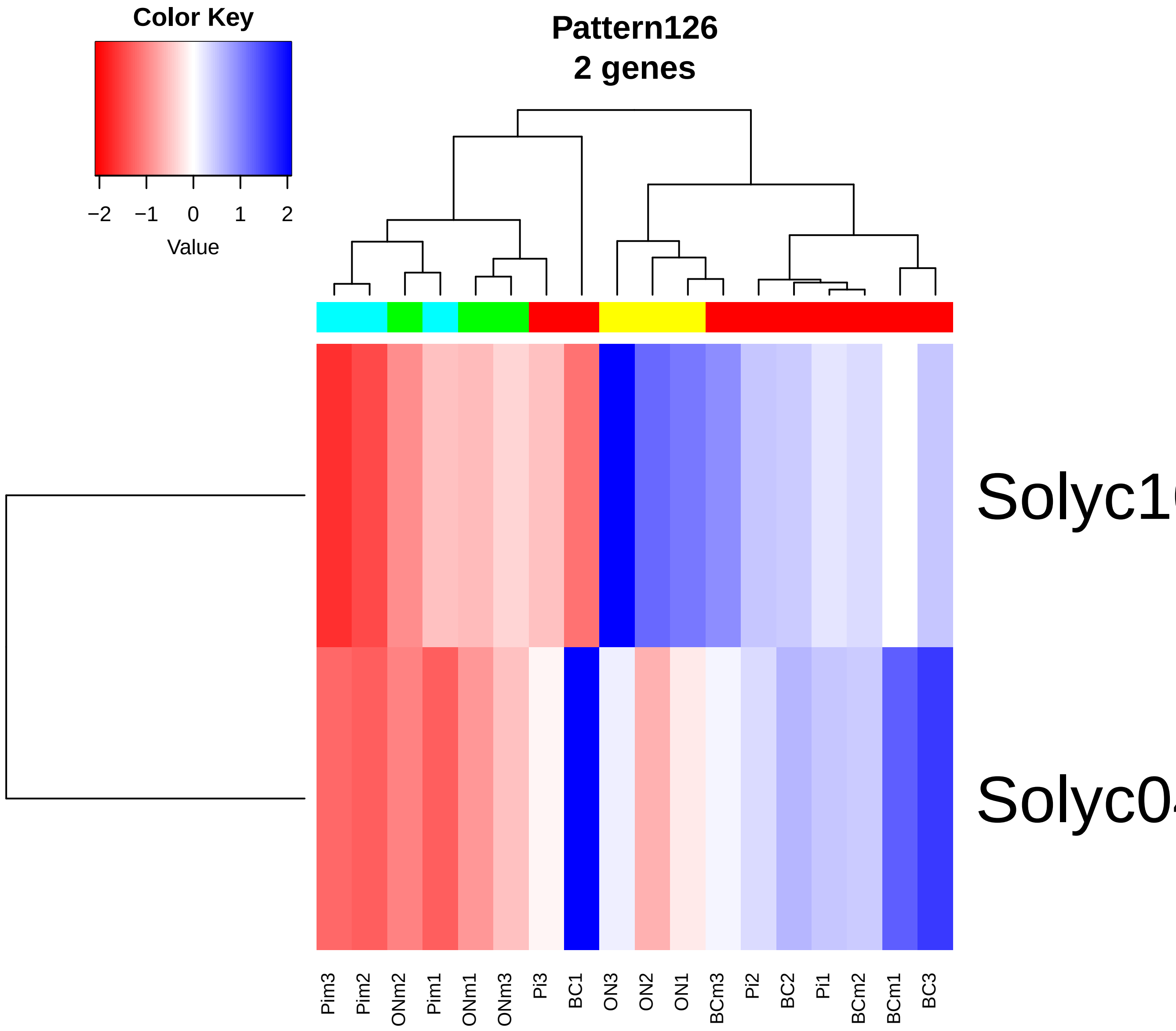
Color Key
−2 −1 0 1 2
Value
Pattern126
2 genes
Solyc1
Solyc0
Pim3
Pim2
ONm2
Pim1
ONm1
ONm3
Pi3
BC1
ON3
ON2
ON1
BCm3
Pi2
BC2
Pi1
BCm2
BCm1
BC3

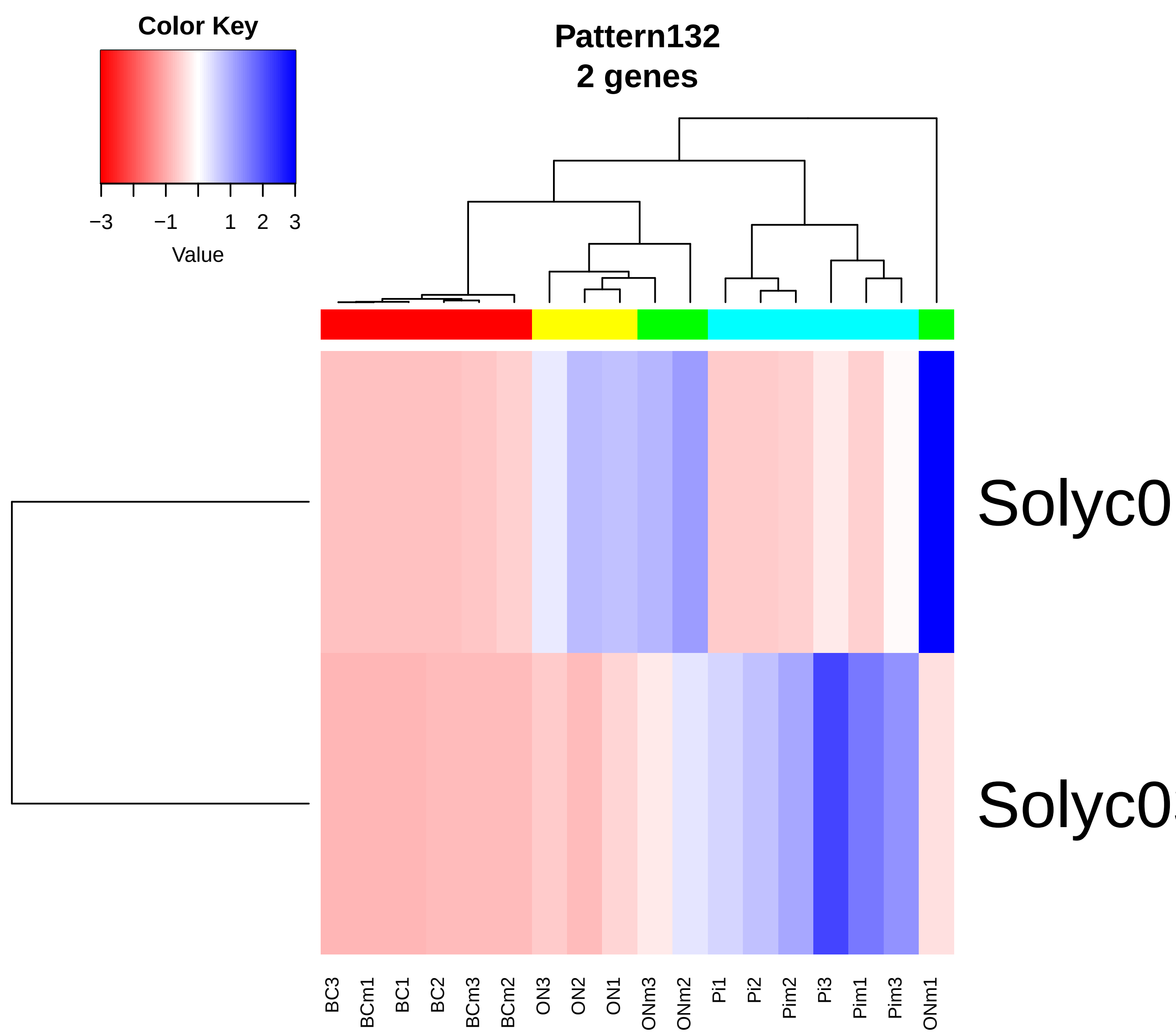

Color Key
−3
−1
1
2
3
Value
Pattern132
2 genes
Solyc0
Solyc0
BC3
BCm1
BC1
BC2
BCm3
BCm2
ON3
ON2
ON1
ONm3
ONm2
Pi1
Pi2
Pim2
Pi3
Pim1
Pim3
ONm1

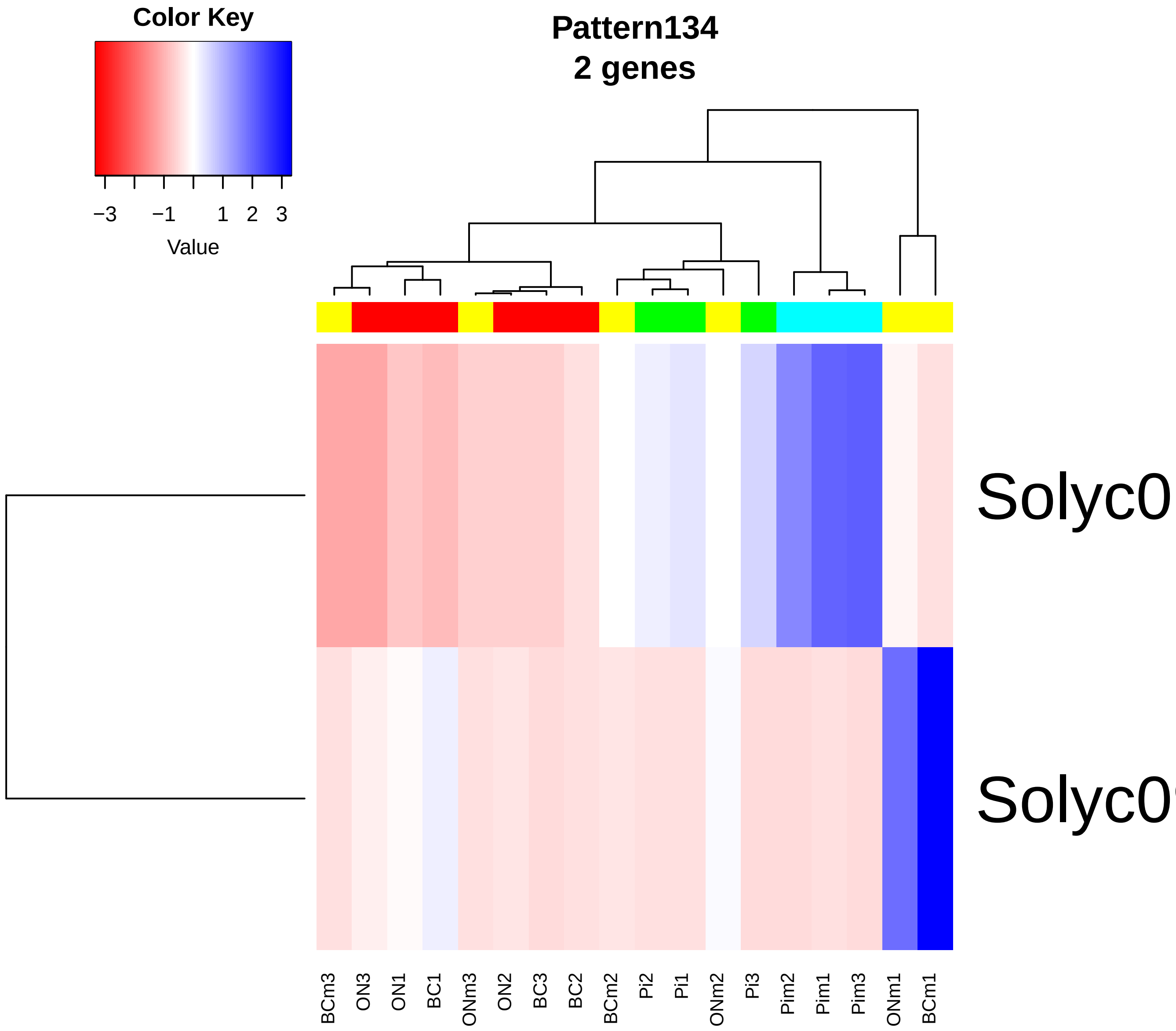

Color Key
−3
−1
1
2
3
Value
Pattern134
2 genes
Solyc0
Solyc0
BCm3
ON3
ON1
BC1
ONm3
ON2
BC3
BC2
BCm2
Pi2
Pi1
ONm2
Pi3
Pim2
Pim1
Pim3
ONm1
BCm1

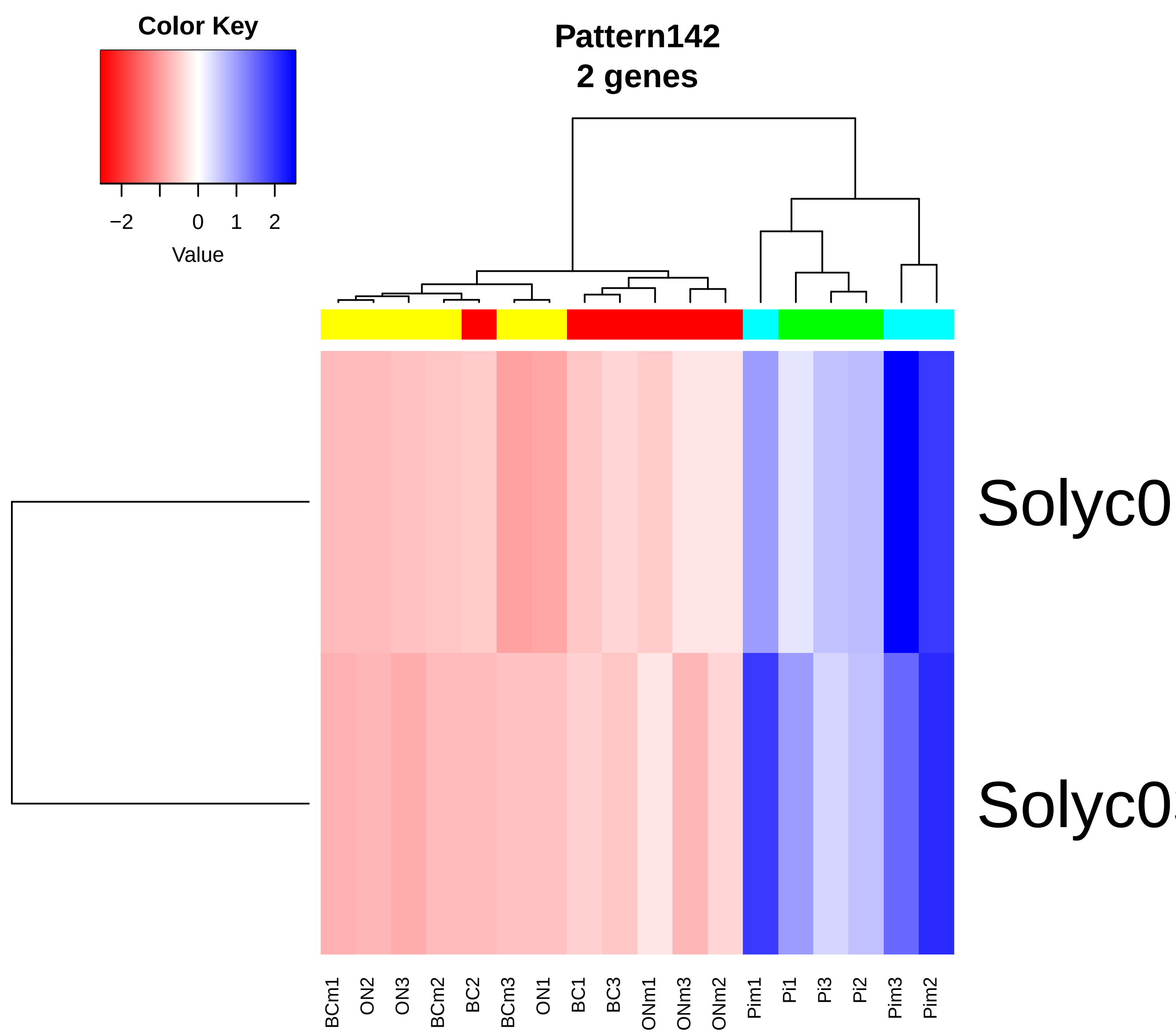
Color Key
−2
0
1
2
Value
Pattern142
2 genes
Solyc0
Solyc0
BCm1
ON2
ON3
BCm2
BC2
BCm3
ON1
BC1
BC3
ONm1
ONm3
ONm2
Pim1
Pi1
Pi3
Pi2
Pim3
Pim2

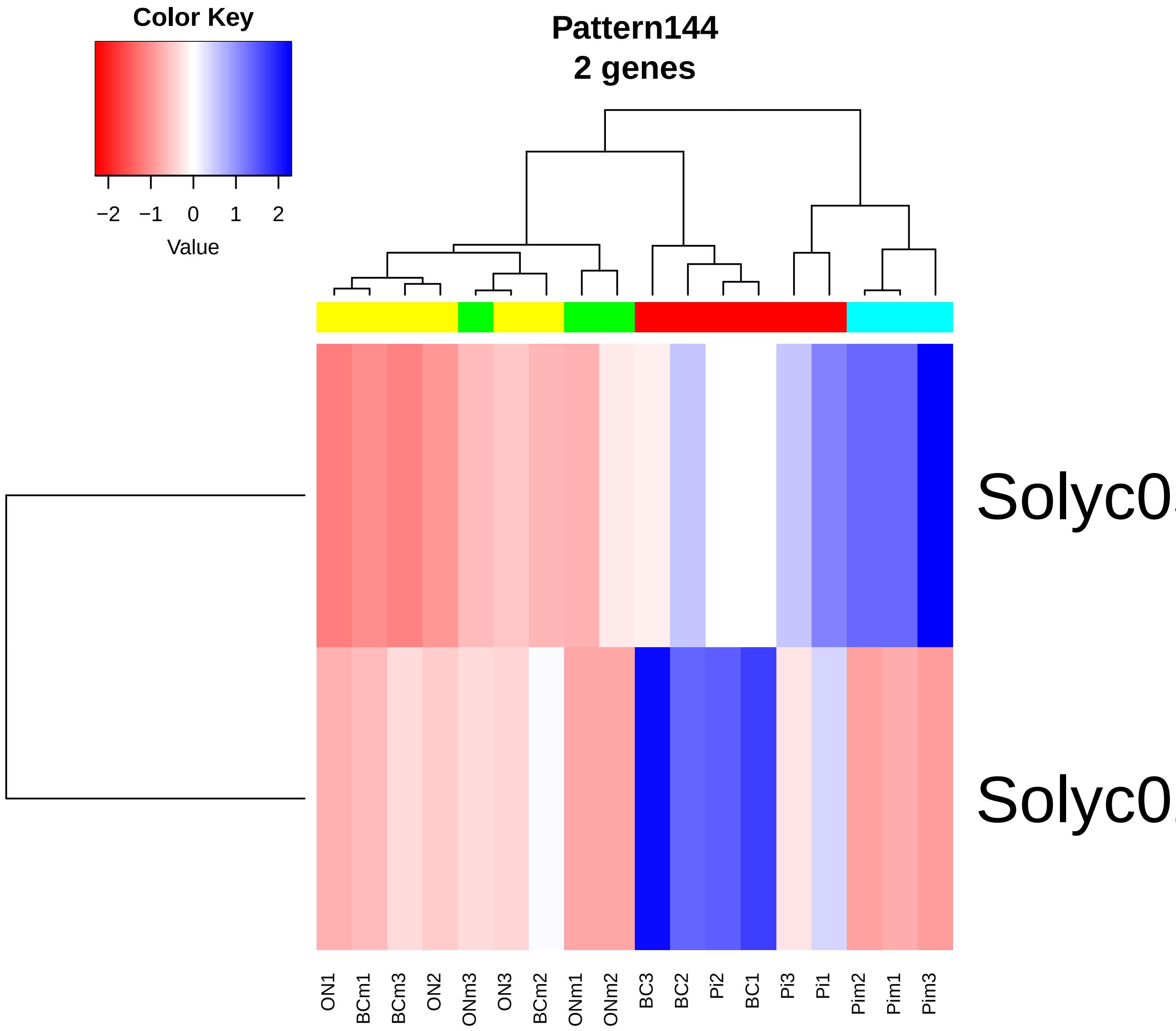

Color Key
−2 −1 0 1 2
Value
Pattern144
2 genes
Solyc0
Solyc0
ON1
BCm1
BCm3
ON2
ONm3
ON3
BCm2
ONm1
ONm2
BC3
BC2
Pi2
BC1
Pi3
Pi1
Pim2
Pim1
Pim3

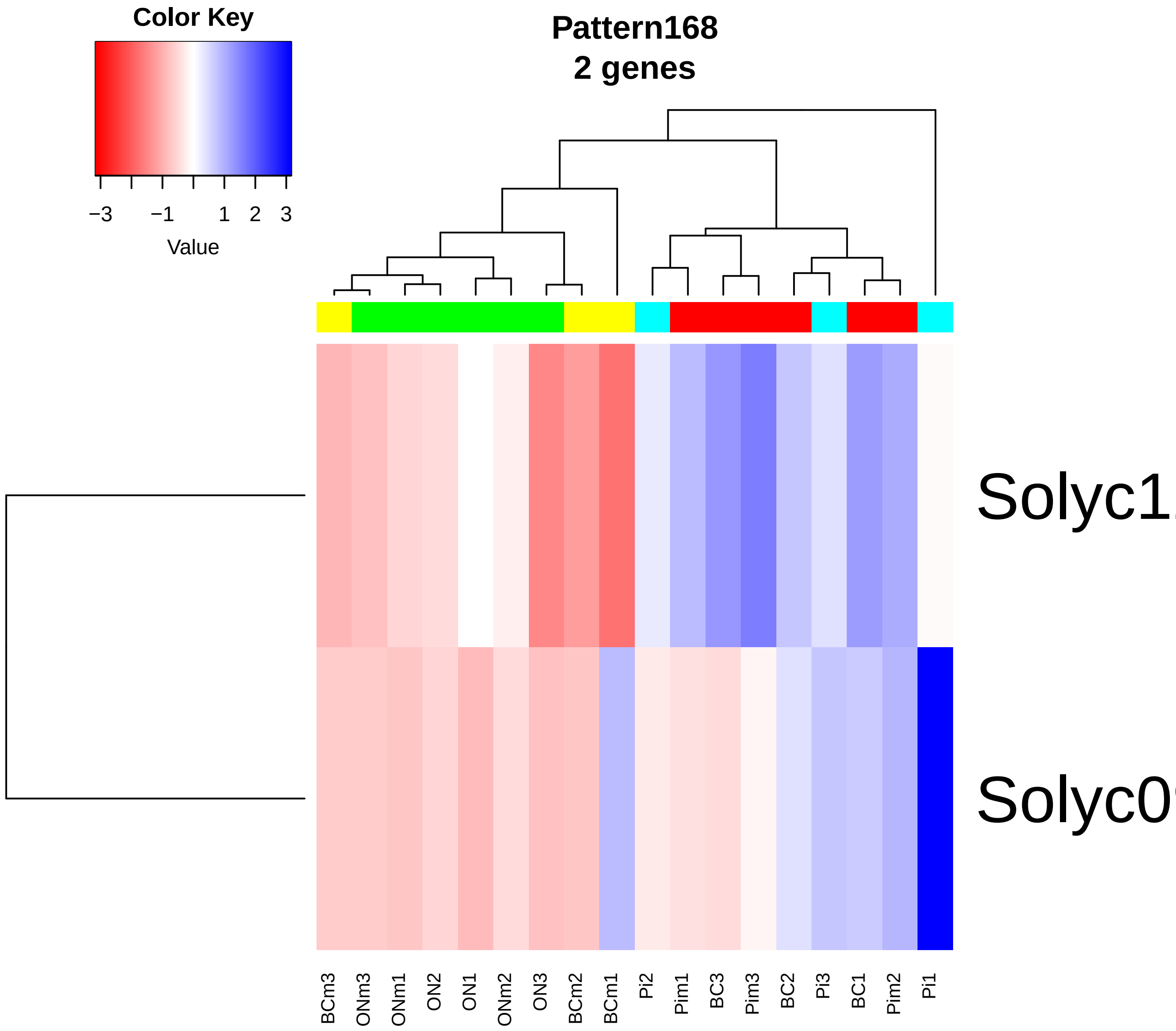

Color Key
−3
−1
1
2
3
Value
Pattern168
2 genes
Solyc1
Solyc0
BCm3
ONm3
ONm1
ON2
ON1
ONm2
ON3
BCm2
BCm1
Pi2
Pim1
BC3
Pim3
BC2
Pi3
BC1
Pim2
Pi1

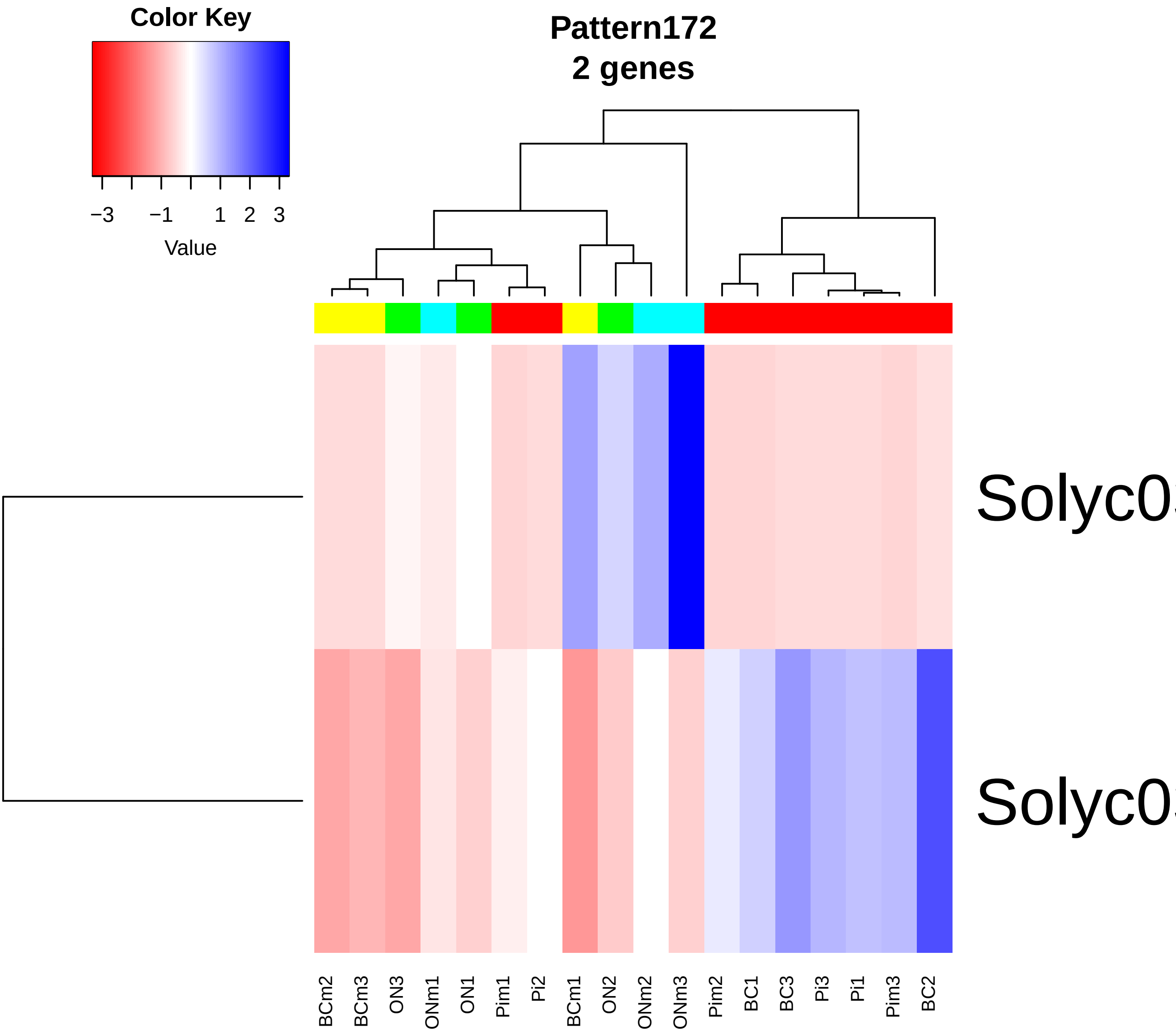
Color Key
−3
−1
1
2
3
Value
Pattern172
2 genes
Solyc0
Solyc0
BCm2
BCm3
ON3
ONm1
ON1
Pim1
Pi2
BCm1
ON2
ONm2
ONm3
Pim2
BC1
BC3
Pi3
Pi1
Pim3
BC2

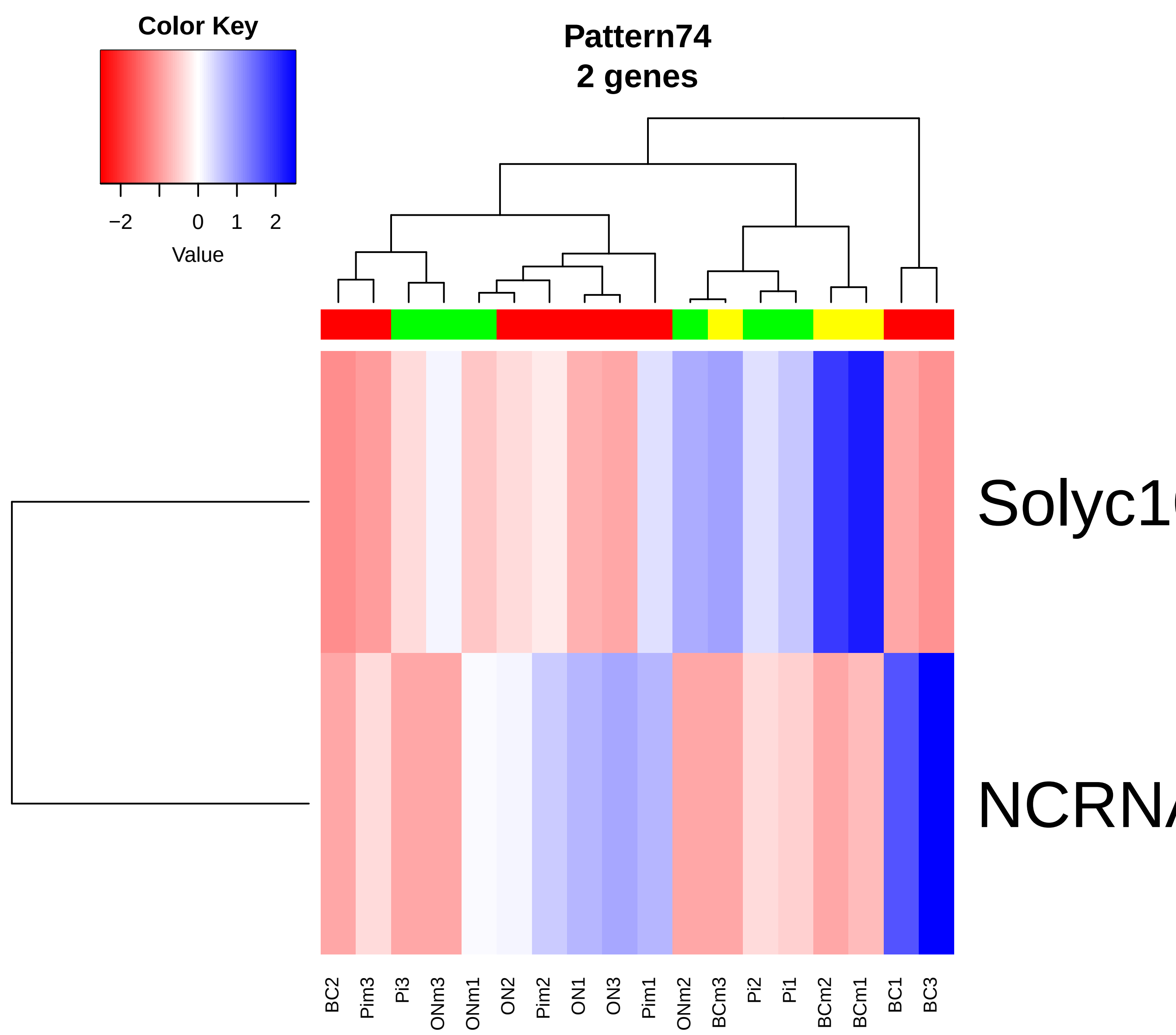
Color Key
−2
0
1
2
Value
Pattern74
2 genes
Solyc1
NCRN
BC2
Pim3
Pi3
ONm3
ONm1
ON2
Pim2
ON1
ON3
Pim1
ONm2
BCm3
Pi2
Pi1
BCm2
BCm1
BC1
BC3

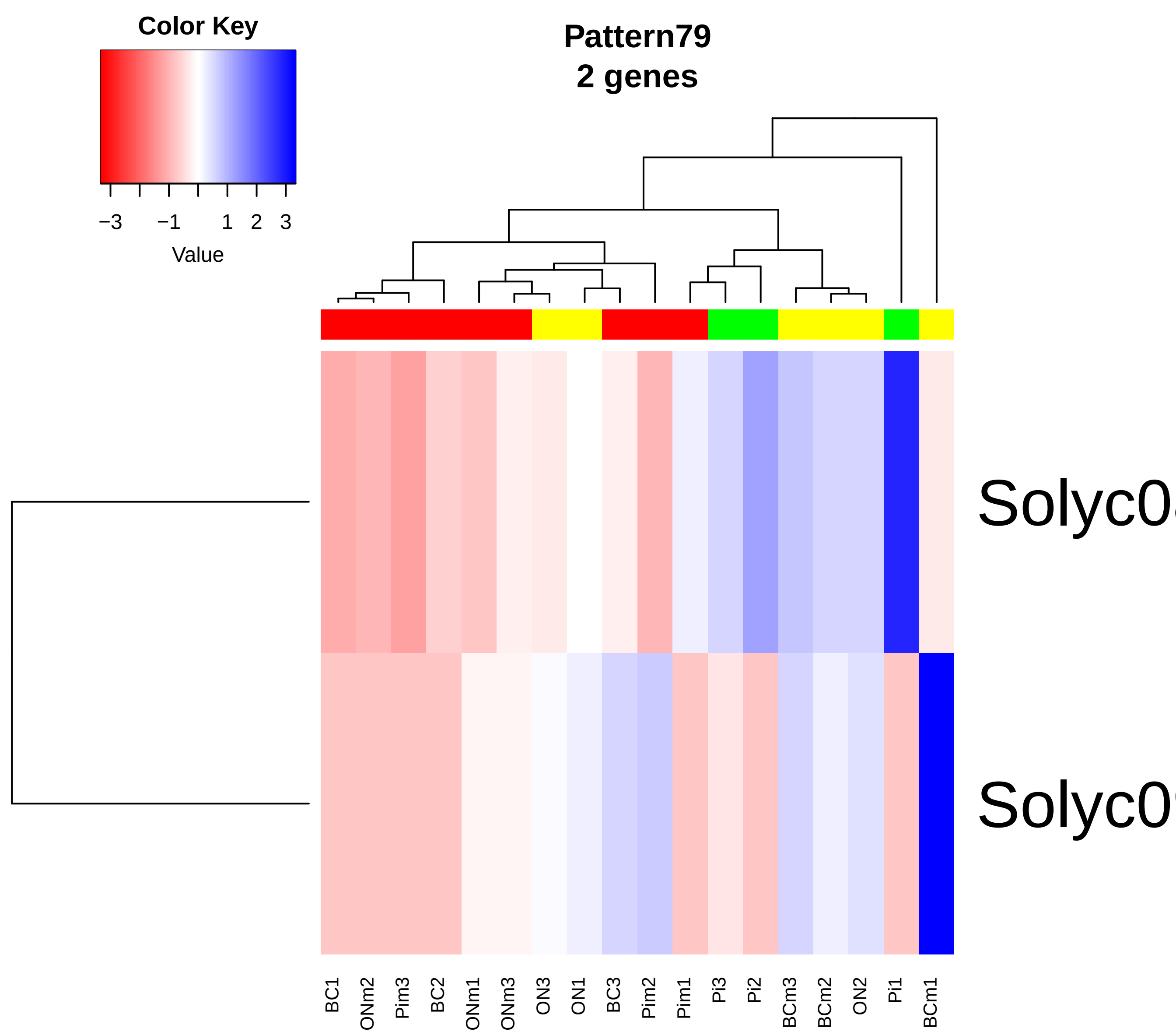

Color Key
−3
−1
1
2
3
Value
Pattern79
2 genes
Solyc0
Solyc0
BC1
ONm2
Pim3
BC2
ONm1
ONm3
ON3
ON1
BC3
Pim2
Pim1
Pi3
Pi2
BCm3
BCm2
ON2
Pi1
BCm1
